# Prion Protein Deficiency Causes Diverse Proteome Shifts in Cell Models That Escape Detection in Brain Tissue

**DOI:** 10.1371/journal.pone.0156779

**Published:** 2016-06-21

**Authors:** Mohadeseh Mehrabian, Dylan Brethour, Declan Williams, Hansen Wang, Hélène Arnould, Benoit Schneider, Gerold Schmitt-Ulms

**Affiliations:** 1 Tanz Centre for Research in Neurodegenerative Diseases, University of Toronto, Toronto, Canada; 2 Departments of Laboratory Medicine & Pathobiology, University of Toronto, Toronto, Canada; 3 French Institute of Health and Medical Research (INSERM), Paris, France, and University Paris Descartes, Paris, France; Van Andel Institute, UNITED STATES

## Abstract

A popular method for studying the function of a given protein is to generate and characterize a suitable model deficient for its expression. For the prion protein (PrP), best known for its role in several invariably fatal neurodegenerative diseases, a natural choice, therefore, would be to undertake such studies with brain samples. We recently documented the surprising observation that PrP deficiency caused a loss or enhancement of NCAM1 polysialylation, dependent on the cell model used. To identify possible causes for this disparity, we set out to systematically investigate the consequence of PrP deficiency on the global proteome in brain tissue and in four distinct cell models. Here we report that PrP deficiency causes robust but surprisingly divergent changes to the global proteomes of cell models but has no discernible impact on the global brain proteome. Amongst >1,500 proteins whose levels were compared in wild-type and PrP-deficient models, members of the MARCKS protein family exhibited pronounced, yet cell model-dependent changes to their steady-state levels. Follow-up experiments revealed that PrP collaborates with members of the MARCKS protein family in its control of NCAM1 polysialylation. We conclude that the physiological function of PrP may be masked in analyses of complex brain samples but its cell-type specific influence on a lipid raft-based NCAM1-related cell biology comes to the fore in investigations of specific cell types.

## Introduction

The prion protein (PrP) is best known for its causative role in several incurable neurodegenerative diseases, including Creutzfeldt-Jakob disease (CJD) in humans, bovine spongiform encephalopathy (BSE) in cattle, scrapie in sheep, and chronic wasting disease (CWD) in cervids [1]. Despite considerable interest in PrP, its cellular role has remained enigmatic, and there is little agreement on molecular players that mediate signals emanating from PrP. The prion gene descended around 500 million years ago from a transcript coding for the ectodomain of an ancestral ZIP zinc transporter [2, 3]. To this day, vertebrate prion proteins exhibit profound sequence similarities to the subbranch of ZIP zinc transporters comprising ZIP5, ZIP6 and ZIP10 [2, 3], and highly similar defects in the execution of a morphogenetic program, known as epithelial-to-mesenchymal transition (EMT) [4], have been reported for zebrafish embryos deficient in ZIP6 [5] or PrP [6]. We recently generated by CRISPR-Cas9 technology PrP knockout clones in the well-established mouse NMuMG EMT model [7] and documented that PrP deficiency leads to an impairment of EMT also in this model [8]. Deep global proteome analyses, conducted to shed light on the mechanism by which PrP may impair EMT, pointed to a PrP-dependent deregulation of neural cell adhesion molecule 1 (NCAM1), a cell adhesion molecule known to directly interact with PrP [9, 10]. Follow-on studies then uncovered that PrP not only stabilizes NCAM1 levels but also controls a signaling pathway that culminates in the polysialylation of NCAM1. This effect of PrP was based on long-range transcriptional control of the *St8sia2* gene, whose polysialyltransferase gene product is responsible for NCAM1 polysialylation in this model. Surprisingly, CRISPR-Cas9-based PrP-deficiency in C2C12 myoblasts (or differentiated myotubes), another mouse cell model popular in cell differentiation studies, did not lead to a similar impairment of NCAM1 polysialylation but caused the opposite effect, namely a strong increase in the levels of polysialylated NCAM1 [8].

To begin to understand how the cellular context of PrP-deficiency might contribute to these differences in phenotypes, we decided to undertake in-depth comparisons of the global proteomes of several PrP-deficient mouse models. We hypothesized that such an approach may not only provide fundamental insights into the consequences of a specific gene knockout in several models but may also reveal candidate proteins that participate in PrP’s control of NCAM1 polysialylation. Here, we describe data that underscore the importance of considering cellular context and differentiation state in interpreting protein-knockout phenotypes. We document that PrP controls steady-state levels of members of the MARCKS and BASP protein families in all cell models we investigated. Moreover, we will show that PrP depletion causes different subsets of members of these protein families to be affected and that the direction of change can be inconsistent across these models. Finally, we will demonstrate that the PrP-dependent stabilization of MARCKSL1 levels contributes to NCAM1-polysialylation during the execution of the morphogenetic EMT program in the NMuMG cell model.

## Materials and Methods

### Antibodies

The antibodies against MARCKS, MARCKSL1, PrP, NCAM1 and beta-actin were purchased from Thermo Scientific, MA, USA (PA1-10021; 1:2500), Bethyl Laboratories, TX, USA (A302-375A; 1:1000), Bertin Pharma, France (A03213; 1:2000), BD Biosciences, ON, Canada (556324; 1:6000) and Cell Signaling Technology, MA, USA (8H10D10; 1:1000), respectively.

### Mouse brain and cell models

All animal protocols were in accordance with the Canadian Council on Animal Care and were approved by the Animal Care and Use Committees at the University of Toronto and the University Health Network. To prevent unnecessary pain or discomfort, animals were monitored on a daily basis. Indicators of failing health, including failure to groom or weight loss exceeding 20% normal body weight, triggered a euthanasia decision. The *Prnp* mouse line was maintained on a FVB/Ncrl background. *Prnp*^*-/-*^ and wild-type mice were obtained by breeding heterozygote *Prnp*^+/-^ mice. At the experimentally indicated ages, or if a decision was made to euthanize an animal, mice were deeply anesthetized by inhalation of isoflurane and sacrificed by cervical dislocation. Animals were housed in groups of three and subjected to artificial light cycles that recapitulated the natural day/night cycles. During the course of the experiment, only one animal was euthanized due to unexplained excessive weight loss and three female mice each of wild-type and *Prnp*^-/-^ genotype were sacrificed at one year of age. Their brains were rapidly dissected and provided the biological source materials for the global proteome analyses.

Neuro-2a neuroblast cells (CCL-131) and C2C12 myoblast cells (CRL-1772) were purchased from American Type Culture Collection (ATCC, VA, USA). NMuMG mouse mammary epithelial cells (CRL-1636) were a kind gift from Dr. Jeffrey Wrana (University of Toronto, ON, Canada). The Neuro2a, C2C12 and NMuMG cells were maintained as described previously [7]. The origins and growth conditions for 1C11 neuroectodermal cells were as previously described [11].

To induce epithelial to mesenchymal transition (EMT), the NMuMG cells were treated with 6.4 ng/mL of TGFB1 (240-B; R&D Systems, USA) for 48h, unless indicated otherwise. The cells were transfected with Silencer Select siRNAs (Thermo Fisher Scientific) against MARCKS (s69470) and MARCKSL1 (s69899) using RNAiMAX Lipofectamine reagent (Thermo Fisher Scientific) 18 h before treatment with TGFB1. When required, cytoplasmic, membrane and nuclear cell fractions were isolated using a kit purchased from Cell Signaling (9038S), which was used according to the manufacturer’s recommendations.

### Generation of PrP-deficiency

The expression of PrP was abolished in N2a, C2C12 and NMuMG cells by CRISPR/Cas9 technology with two previously described guide RNAs, which were targeted to the beginning of the Exon 3 coding sequence. More specifically, the insertion of indels by the cell autonomous non-homologous end joining program led to a frame shift, which generated a premature translation stop codon [7].

The PrP knockdown in 1C11 clones was obtained by stable transfection of PrP-specific shRNAs coded by the pTER plasmid [12], which also comprised an expression cassette for a zeocin resistance selection marker, followed by the selection of positive transfectants in the presence of 1 mg/mL of zeocin over a three week period, as previously described [11]. PrP deficient mice used in this work were derived from a well-characterized mouse line, which had originally been generated by inserting a neo cassette into Exon 3 of the *Prnp* gene [13].

### Sample preparation for global proteome analyses

Mice were euthanized and their brains rapidly dissected from the skull. Brains or cells were homogenized in SDS-containing Lysis Buffer (2% SDS, 62.5 mM HEPES/NaOH, pH 8.0; preheated to 90°C), with the aid of 1.0 mm zirconia beads and a Mini-BeadBeater-8 (Biospec Products Inc., Oklahoma, USA). Following three cycles of 1 minute bead beading, the lysates were further incubated at 90°C to deactivate residual enzymatic activities in the extracts. Protein levels were adjusted by BCA colorimetric assay (Thermo Scientific, Nepean, Ontario, Canada) before sample preparation for global proteome analyses. Protein precipitation, denaturation, reduction, alkylation and digestion were performed as previously described [7]. MS grade trypsin was from Thermo Scientific. Tryptic peptides were covalently modified with the TMTsixplex isobaric label reagent set (Thermo Scientific) according to the protocol supplied by the manufacturer.

### Quantitative mass spectrometry

Following C18 and SCX (zip tip) purification, TMT labeled tryptic digests were separated on an Easy-nLC 1000 HPLC system (Thermo Scientific) and simultaneously analyzed on an Orbitrap Fusion (Thermo Scientific) mass spectrometer. Samples were loaded directly onto the analytical column by the integrated autosampler. Peptide separation was conducted at a flow rate of 300 nL/min over a 240 minute acetonitrile gradient on an Acclaim PepMap RSLC C18 column (75 micrometer internal diameter, 25 cm long, 2 micrometer particle size with 100 Å pores). Mobile phases A and B consisted of 0.1% (v/v) formic acid in water or in acetonitrile containing 5% (v/v) water respectively. In each separation the mobile phase B content of the eluent was brought from 0 to 30% over a 180 minute period and then to 100% over 60 minutes. The column was washed for 20 minutes with 100% mobile phase B after each separation.

All mass spectra were collected in positive ion mode with the nanoflow electrospray ionization source held at a potential of 1850 V, an S-Lens RF value of 60% and an ion transfer tube temperature of 275 degrees Celsius. The data acquisition cycle time was fixed at 3 seconds and consisted of a single 120,000 resolution precursor ion scan from m/z 400 to 2,000 on the Orbitrap mass analyzer followed by MS/MS scans on the linear ion trap and Orbitrap MS^3^ scans from m/z 100 to 500 at 60,000 resolution. Only ions with charge states between 2 and 7 having intensities over 5,000 counts were selected for MS/MS. Dynamic exclusion was applied for 6,000 seconds to precursor ions having been analyzed by MS/MS.

Peptide sequences, post-translational modifications and protein identities were determined from LC-MS data using both the Mascot server (version 2.4.1) and Sequest HT search engines operated in Proteome Discoverer (version 1.4.0.288). All MS/MS data were searched against version 3.87 of the *Mus musculus* IPI database, which contained 26,627,161 amino acid residues within 59,534 protein entries (release date September 27, 2011). False discovery rate (FDR) estimation was conducted using the Proteome Discoverer Percolator algorithm with validation based on q-value. Precursor and product ion mass tolerances were set to 20 ppm and 0.6 Da, respectively. Dynamic modifications considered were asparagine and glutamine deamidation, methionine oxidation, cysteine pyridylethylation and phosphorylation of serine, threonine or tyrosine. The TMT tag (229.163 Da) was set as a fixed modification at lysine and amino-terminal residues. Only tryptic peptides between 400 and 6,000 Da in mass, with up to one missed cleavage site, were considered in the search. Peptide assignments were restricted to sequences of 6 to 144 residues. Peak areas from the six TMT reporter ions in MS^3^ data were used for peptide quantification. Isotopic purity correction factors for each reporter ion were used in accordance with the manufacturer’s recommendation. All mass spectrometry proteomics data generated in this study have been deposited (identifier PXD003398) to the ProteomeXchange Consortium [14] via the PRIDE partner repository [15] and can be accessed through the following link: http://www.ebi.ac.uk/pride/archive/projects/PXD003398.

### Gene ontology analysis

Filtered protein-level data (peptide score ≥ 0.05 FDR estimation cut-off) were converted to prot.xml format in Proteome Discoverer, exported to ProteinCenter (version 3.12.10021 build release 3-12-10021.1) and subjected to ProteinCenter gene ontology (GO) analysis. Outputs from queries that tallied proteins for each model system according to their GO ‘slim’ annotations in the categories ‘Cellular Components’, ‘Biological Processes’ and ‘Molecular functions’ were combined and assembled in the three graphical analyses.

### Cluster analysis

Hierarchical clustering was conducted with Cluster (Version 3.0) and Java TreeView (Version 1.26) open source software, following published instructions [16, 17]. More specifically, prior to clustering, data columns (representing samples depicting the three biological replicates for each of the five mouse models) were centered on their means (protein level data represented in rows were not further adjusted). Subsequently, steady-state protein level data (rows) were clustered using a ‘city-block’ algorithm. The unbiased comparison of samples (columns) was based on a Spearman Rank Correlation, a non-parametric alternative to the Pearson correlation coefficient, which was selected to protect the results from the influence of outliers. Data were visualized in Java TreeView and individual components exported in postscript format for assembly of the figure in Adobe Illustrator.

### Multiple alignment

The amino acid sequence alignment of members of the MARCKS protein family was generated by AlignX, a multiple alignment algorithm embedded in the Vector NTI Advance software suite (Version 11.0; Invitrogen). The alignment was based on the blosum62mt2 score matrix and was computed with gap opening and extension penalties set to 10 and 0.05, respectively.

### SDS-PAGE and western blot

Cells lysates were collected using 1% NP-40, 50 mM Tris (pH 8.0) and 150 mM NaCl, supplemented with Complete Protease Inhibitor Cocktail (11836170001; Roche, ON, Canada). The protein levels were adjusted using the bicinchoninic acid (BCA) assay (Thermo Fisher Scientific) and loaded for SDS-PAGE analyses on to 4–12% Bis-Tris (Thermo Fisher Scientific; NP0322) or 7% Tris-glycine gels (cast in-house). The proteins were subsequently transferred to polyvinylidene fluoride membrane and followed by one hour of blocking and overnight primary antibody incubation at 4°C. The signals were visualized after incubation with the HRP-conjugated anti-mouse (1:5000, 170–6516; BioRad, ON, Canada) or anti-rabbit (1:5000, 170–6515; BioRad) secondary antibodies and the ECL reagent (RPN2106; GE Healthcare) on the LI-COR Odyssey Fc digital imaging system (LI-COR Biosciences, NE, USA).

### RT-PCR methodology

Cell pellets were collected in PBS and later subjected to RNA extraction with the QIAshredder (79654; Qiagen, ON, Canada) and RNeasy Mini kit (74104; Qiagen). 1 μg of RNA was used for cDNA synthesis by the AffinityScript Multiple Temperature cDNA Synthesis Kit (200436; Agilent Technologies, ON, Canada) with both oligo dT and random primers. Subsequently, real-time PCR amplification was performed with the StepOnePlus machine (Thermo Fisher Scientific, MA, USA) using TaqMan gene expression assays (Thermo Fisher Scientific) for mouse ST8sia2 (Mm01311039_m1), Hprt (Mm00446968_m1) and Tfrc (Mm00441941_m1). The results are expressed relative to the control sample.

### Statistics

To evaluate the mass accuracy of peptide-to-spectrum matches (PSMs) from searches against the forward and reversed IPI mouse FASTA database, the mass accuracy of precursor ions was plotted against the relative number of peptide identifications. Calculated precursor ion m/z errors were grouped into ranges of 0.5 parts per million (ppm) within the mass accuracy interval specified in the Proteome Discoverer workflow search parameters (-20 to 20 ppm). The number of PSMs for each precursor ion m/z error range was counted. Whereas the analysis was restricted to PSMs from the forward IPI database search exceeding 0.05 FDR estimation cut-off scores, all PSMs from the reversed IPI database search were included.

To generate the chart which bins proteins on the basis of the number of peptides underlying their identification, protein level search results (based on peptide sequence assignments having scores exceeding a 0.05 FDR cut-off) were exported and processed in Microsoft Excel. Protein and protein group identifications from each sample were counted according to the number of associated peptide sequence assignments using the COUNTIF function.

To compute Pearson correlation coefficients of TMT abundance ratios of proteins as a function of spectral counts, proteins were sorted based on the number of spectral counts underlying their identification and quantitation in biological replicates, grouped in bins of 125 proteins each, and the Pearson correlations for the relative quantitation within these bins were determined. Pearson correlation coefficients were lower when fewer peptides were assigned (the cut-off was set to a minimum of 3 peptides identified and quantified).

## Results

### Experimental design of global proteome analyses of PrP-deficient mouse models

**Fig 1:**
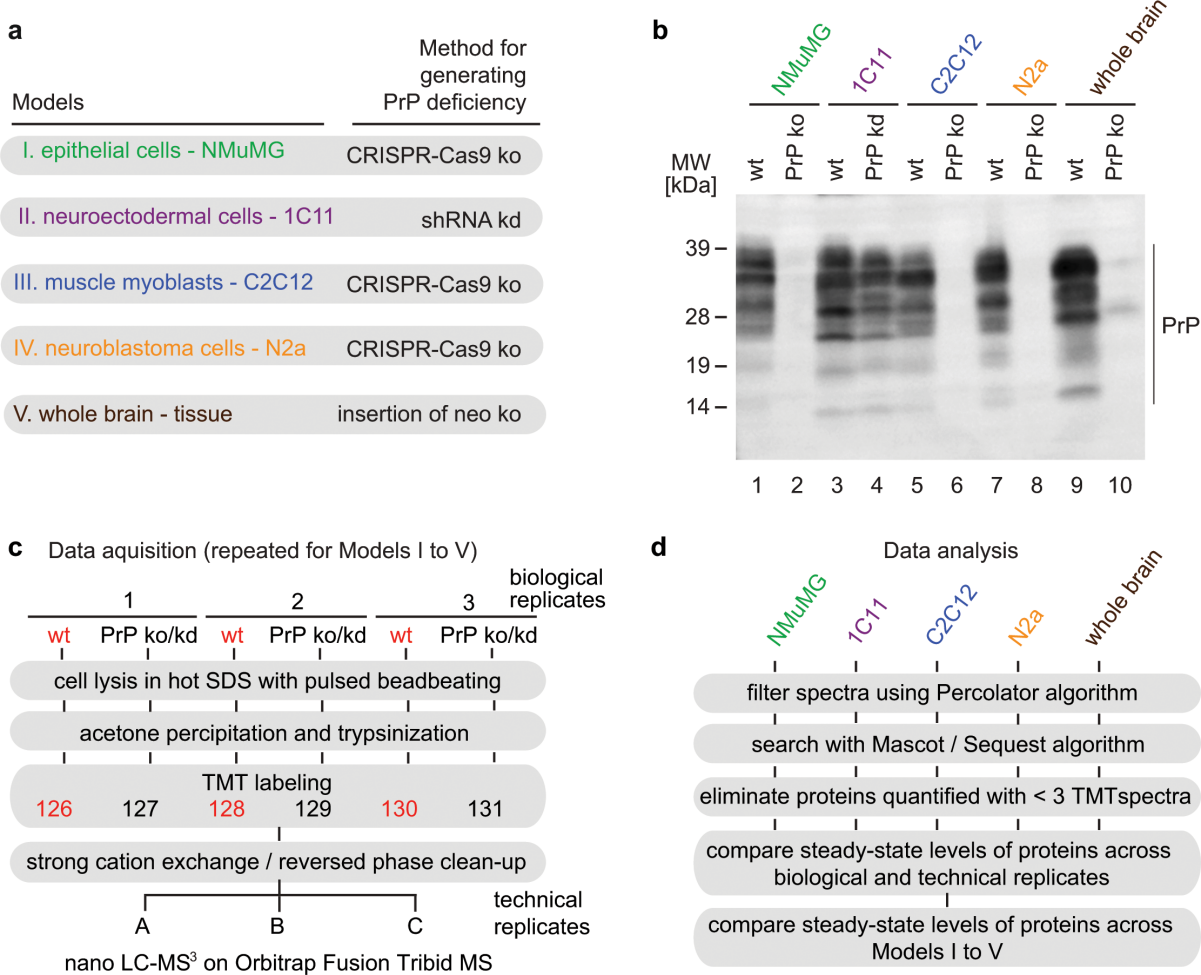
Study design for global proteome comparison of PrP-deficient mouse models. (a) Mouse models used in this study. (b) Western blot analysis of endogenous PrP expression products in wild-type control samples and the PrP-deficiency models derived from them. (c) Flowchart depicting sample preparation steps for generating protein digests, isobaric labeling of peptides and relative quantitative mass spectrometry analyses. (d) Workflow for post-acquisition analyses of global proteome datasets.

The study made use of five previously described PrP-deficient mouse models, four cell models and one whole mouse model (**Fig 1A**). More specifically, mouse brains were obtained from a well-characterized mouse line, which had originally been generated by inserting a neo cassette into Exon 3 of the *Prnp* gene [13]. The PrP-deficient cell models selected for this study were an NMuMG epithelial cell clone, a clone derived from C2C12 muscle myoblasts, and a Neuro2a neuroblastoma cell clone, in which CRISPR-Cas9-based indels in Exon3 led to non-productive PrP expression [7], as well as the 1C11 neuroectodermal cell model exhibiting diminished PrP expression following stable expression of a PrP-specific shRNA [11]. The specific cell clones were selected with a view to cover a broad range of cellular differentiation states and work with models that are popular for PrP-related studies, either because they are known to be susceptible to infection (N2a, C2C12, 1C11, mouse) and/or have retained the ability to (trans)differentiate (C2C12: myoblast to myotube; NMuMG: epithelial to mesenchymal state; 1C11: neuroectodermal to neuronal cells with sprouting neurites). As negative controls served wild-type mice (obtained as littermates of *Prnp*^-/-^ mice by breeding heterozygote *Prnp*^+/-^ mice) and parental cell lines expressing endogenous levels of PrP. Western blot analyses of these models confirmed PrP-deficiency as expected and revealed in wild-type control samples from each model the expression of varying levels of endogenous *Prnp* gene products. The latter migrated during electrophoresis in denaturing sodium dodecyl sulfate (SDS) polyacrylamide gels with the characteristic relative speeds expected for unglycosylated, mono- and di-glycosylated PrP, or were observed as faster migrating bands, presumably indicating the presence of the prominent C1 cleavage products, known to exist in these models (**Fig 1B**) [7, 18, 19].

To generate global proteomics datasets, three biological replicates (cell culture dishes or mouse brains) were collected from the aforementioned cell clones or mice and subjected to rapid lysis in pre-heated SDS, using bead-beating for efficient homogenization and to promote the shearing of DNA. Following adjustment of protein concentrations, the protein content in equal volumes of extracts was acetone precipitated and denatured in 9 M urea. Subsequently, disulfide bonds were reduced and alkylated and protein samples digested with trypsin. To facilitate the direct comparison of steady-state protein levels in subsequent pair-wise comparisons and to avoid run-to-run variance, peptides were labeled with isobaric tandem mass tags (TMT) [20] and combined into one sample. Subsequently, peptide mixtures were separated on a C18 reversed-phase nanoflow column by a four-hour linear gradient of 0–30% acetonitrile in 0.1% formic acid, ionized by electrospray ionization and analyzed in an Orbitrap Fusion Tribrid mass spectrometer (**Fig 1C**). The method for analysis relied on (1) a precursor scan, (2) a tandem MS analysis (MS2) of the most intense precursor ions carrying a minimum of two charges, fragmented by collision-induced dissociation (CID), (3) the concomitant selection and further fragmentation of the ten most intense peptide fragments to yield MS3 spectra documenting on TMT signature ion profiles. The post-acquisition data analyses against the international protein index (IPI) mouse database centered on the Percolator algorithm, which uses a semi-supervised machine learning approach to discriminate confident identifications [21], and was conducted with search engines Mascot and Sequest embedded in the Proteome Discoverer platform. This first analysis was used to determine the composition of the global proteome in each of the models. To limit the influence of outlier MS3 data for the subsequent comparative quantitations, we stipulated that only proteins would be considered in this analysis, whose identifications were based on a minimum of three PSMs in a given dataset. We further required that the corresponding low mass TMT signature ion distributions for relative quantitation had not only been obtained for these PSMs but were collected at a time when no other precursor co-isolated with the precursor ion of interest and were of sufficient intensity to pass the respective Proteome Discoverer stringency filter (**Fig 1D**).

### At similar depths of proteome coverage, global proteomes reflect differences in cellular origins of mouse models studied

**Fig 2:**
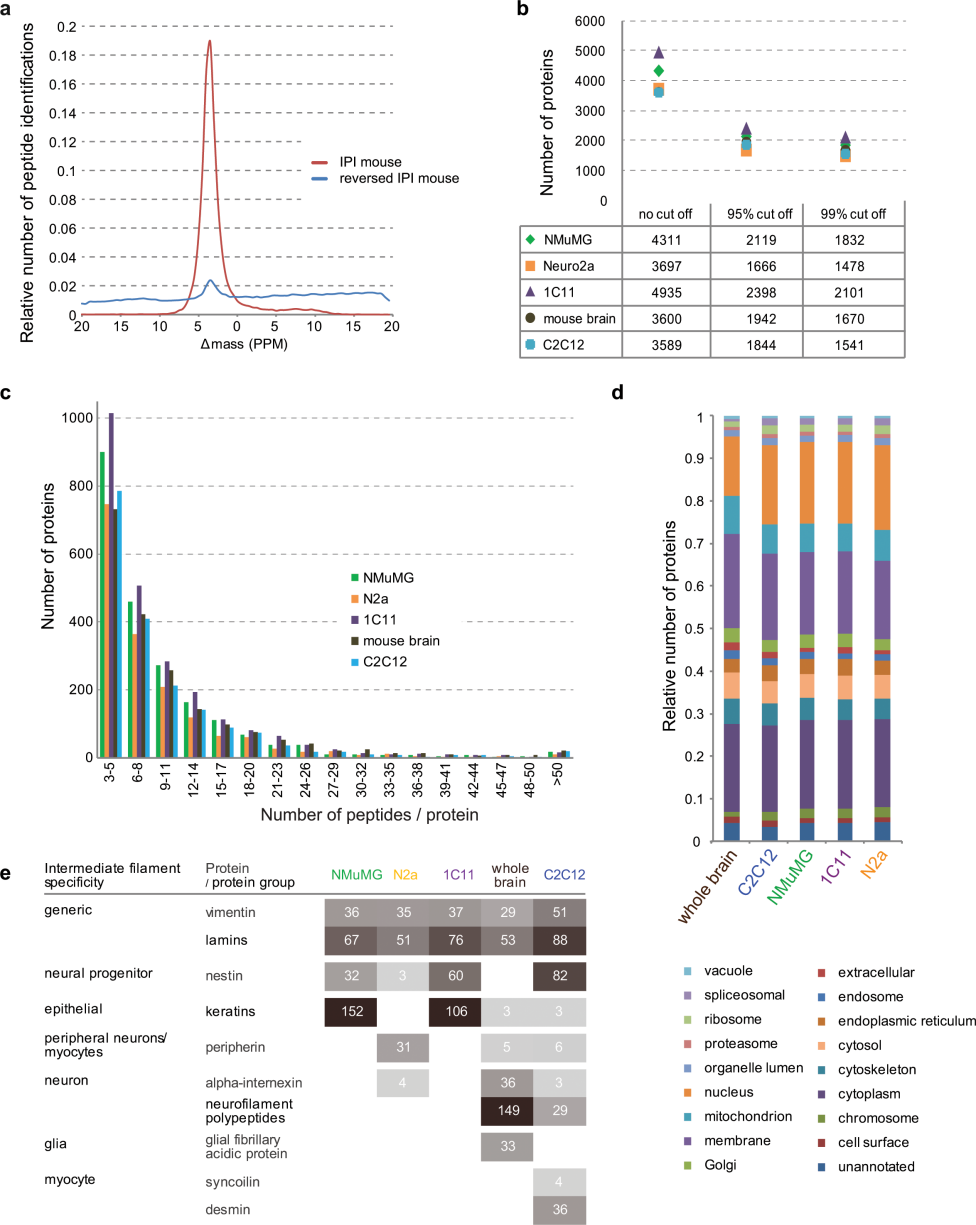
Global proteome datasets exhibit consistent data quality, sequence coverage and gene ontology distributions. (a) Precursor mass error against relative number of peptide identifications for dataset I against target and decoy database. (b) Total number of proteins identified in the five global proteome analyses when increasingly stringent peptide score filters are applied. (c) Chart depicting the binned distributions of peptide assignments per protein (bin size = 3) in the five global proteome datasets. Note that only peptides exceeding 0.95 confidence cut-off score were included in this analysis. (d) Bar diagram comparing ‘Cellular Component’ gene ontology (GO) analyses of global proteome datasets. Note the underrepresentation of proteins identified in mouse brain samples that were assigned to the ‘Nucleus’. (e) Distribution of intermediate filament proteins over the five datasets. The numbers shown represent the number of peptides identified from each protein and correlate directly with the intensity of grey shading.

The global proteome datasets collected exhibited exquisite mass accuracy of PSMs (with mass deviations not exceeding 10 ppm for more than 99% of PSMs) (**Fig 2A**) and led for each of the five PrP-deficiency models to a global proteome coverage of >3,500 proteins, of which > 1,500 proteins in each of the datasets passed >95% confidence thresholds (**Fig 2B**). A more detailed binning of proteins based on the number of peptides, which had been assigned to them, revealed consistent depths of analyses in a direct comparison of all datasets (**Fig 2C**). To gauge the extent to which inherent differences in the biological samples and inconsistent sample handling might have generated variations in the representation of sub-proteomes, a gene ontology (GO) analysis was conducted for each of the five datasets. These analyses, which tallied proteins according to their known GO Cellular Component (CC), Molecular Function (MF) and Biological Process (BP) annotations, established generally consistent numbers of proteins assigned to specific GO categories in all five datasets (**Fig 2D, S1 Fig**). Exceptions to this prevailing trend were an overrepresentation of proteins belonging to the CC ‘mitochondrion’ (434 proteins in the brain versus an average of 345 proteins in the four cell models) and an underrepresentation of proteins known to primarily reside in the CC ‘nucleus’ in the mouse brain-derived global proteome analyses, relative to the respective protein tallies assigned to these CCs in the four cell models (672 proteins in the brain versus an average of 980 proteins in the four cell models). Similarly, there was an overrepresentation of proteins annotated to play a role in the BP ‘transport’ (as well as the corresponding MF ‘transporter activity’) and an underrepresentation of proteins related to the MFs ‘DNA binding’ and ‘RNA binding’ (MF) and the BP ‘cell proliferation’ in mouse brain-derived samples. Although we have not further investigated the underlying causes for these relative shifts in the global proteome of brain-derived samples, it did not escape us that brain tissue might exhibit them because a large subset of its cells are non-proliferating and equipped with neuritic extensions expected to require, relative to non-neuronal cells, additional investments in energy-consuming transport processes.

The inventory of intermediate filaments (IFs) in a given cell is well-known to depend on the cell type and its differentiation state [22]. Because the objective of this study was to identify global proteome differences that may underlie divergent molecular phenotypes in distinct cell models, a positive internal control was sought to test if the methodology applied had led to global proteome datasets that exhibited expected cell type-specific differences. Indeed, when we extracted IF data from the five datasets, we observed that IFs known to be present in a broad range of cell types (e.g., vimentin and lamin) had been detected in all samples, IFs known to be present in epithelial cells were restricted to the NMuMG and 1C11 models (keratins), and, at the other end of the spectrum, muscle-specific IFs (syncoilin and desmin) were only observed in C2C12 myoblasts (**Fig 2E**). Note that because all datasets provided comparable proteome coverage, the relative expression level of a given protein across the five dataset can be inferred by comparing the spectral counts underlying its respective identifications [23]. Taken together, these data established that the coverage achieved in the global proteome analyses and the relative distributions of sub-proteomes across the five datasets were comparable, yet expected cell-type specific differences were preserved.

### Hierarchical clustering reveals surprising disparity in the consequence of PrP-deficiency on global proteomes

We next turned to investigating the specific consequences of PrP-deficiency in the various models, which relied on the TMT-based relative quantitations. A direct comparison of the datasets revealed 4,260 proteins (**S1 Table and S2 Fig**), which were observed to be quantified in at least one of the datasets on the basis of a minimum of three PSMs that were accompanied by informative TMT signature ion profiles. To ensure a meaningful comparison between the models was possible, we initially limited analyses to the subset of 1,559 proteins, whose levels were quantified in all four cell lines (a strict requirement for the quantitation of proteins in the brain dataset was waived for reasons described below). Thus, individual proteins were represented by steady-state abundance profiles that consisted of 12 (cell models only) to 15 (all models) data points, based on the 4 (cell models only) to 5 (all models) x 3 (biological replicates) samples. Because the quantitation measured relative abundances, each data point represented the ratio of steady-state protein levels (in log_2_ space) in wild-type versus PrP-deficient samples of a given protein in one of the five mouse models.

**Fig 3:**
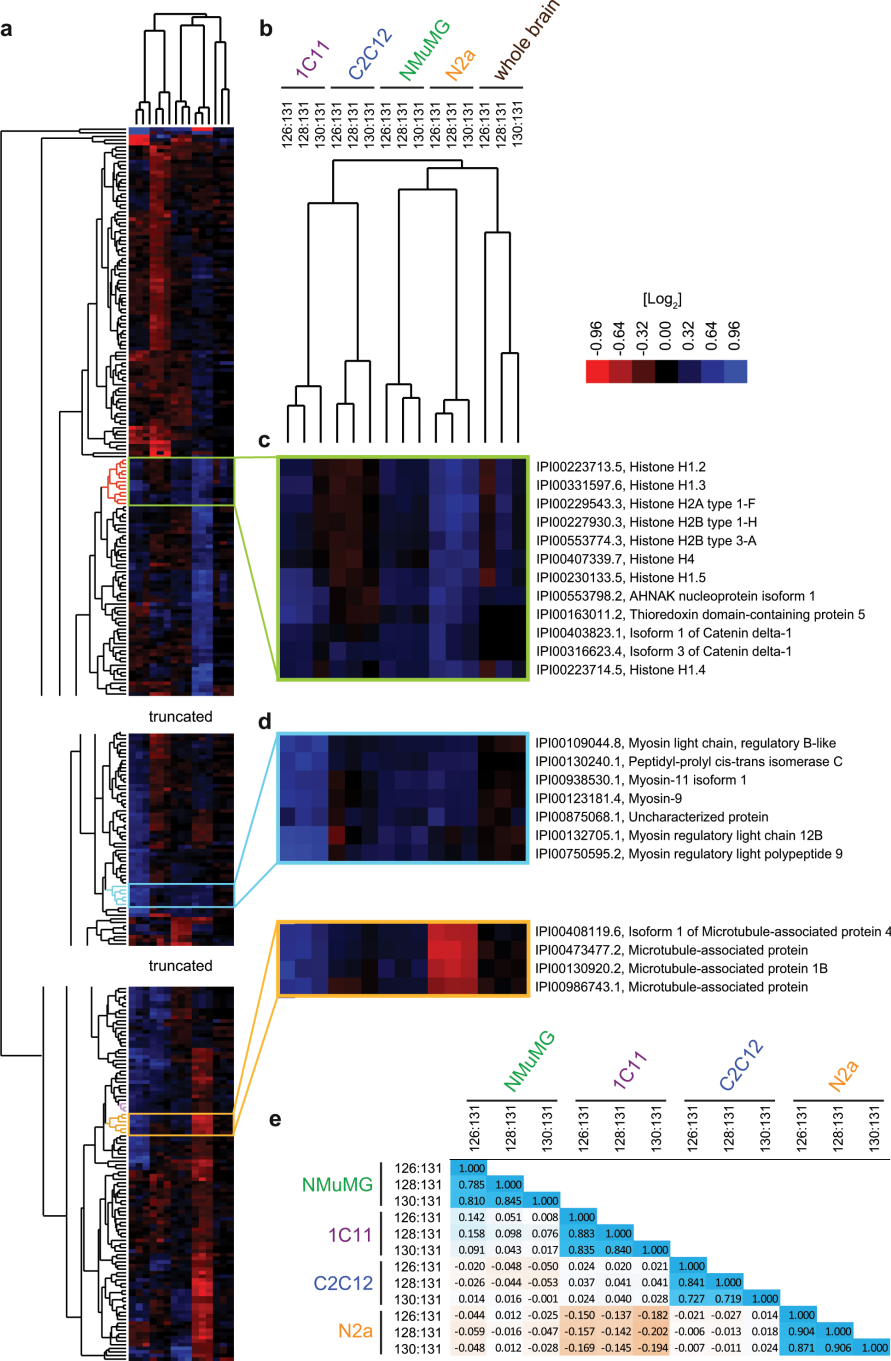
High reproducibility of analyses is contrasted by divergence of sub-proteomes affected by PrP-deficiency in the five models. (a) Hierarchical clustering of proteins (vertical tree) and samples (horizontal tree) of the five global proteome datasets based on TMT-based steady-state protein level abundance ratios of wild-type versus PrP-deficient samples. Note that this analysis was restricted to proteins that were identified and quantified on the basis of a minimum of three TMT signature ion profiles in all four cell models. The heat map color code depicts protein ratios in log_2_ space, with proteins whose levels correlate directly or inversely with PrP levels shown in blue and red, respectively (see legend for details). (b) Tree diagram obtained by hierarchical clustering of protein level ratio profiles in the 15 samples analyzed. The analysis revealed a close relationship of profiles belonging to biological replicates (indicated by short branches connecting them) and relatively low similarities across cell models. (c) Enlarged view of subset of hierarchical cluster depicting similarity in expression profile of members of the histone family across the five mouse models studied. (d) Hierarchical clustering groups members of the protein families of cellular myosins, and mircrotubule associated proteins in small distinct clusters. (e) Pearson correlation analyses of protein abundance ratios reveals a pronounced direct correlation between samples of the same cell model but relatively weak correlations of PrP-dependent changes to the cellular proteome across cell models. Note the weakly negative Pearson coefficients (up to -0.202) in comparisons of samples derived from N2a and 1C11 models, which indicates that PrP-deficiency in these models may manifest in opposite changes in the abundance ratios of a subset of proteins.

Unsupervised hierarchical clustering of the vertical protein level profiles (representing the 15 samples) (**Fig 3A and S2 Fig**) consistently grouped biological replicates of the same model in juxtaposed short sub-branches of the tree diagram, thereby indicating robustness of the methodology (**Fig 3B**). When hierarchical clustering was applied to protein entries based on their abundance ratio profiles, proteins known to represent members of the same protein complexes were observed to group together (e.g., histones, ribosomal subunits or subunits of the proteasome) (**Fig 3C**). A similar co-clustering of proteins was observed for members of protein families (e.g., myosins and microtubule-associated proteins) (**Fig 3D**). Our preliminary investigations into the causes of this clustering of paralogs revealed that sequence similarity (potentially giving rise to a subset of peptide quantitations being ambiguously assigned to more than one paralog) was not the predominant reason, as this behavior was also observed for paralogs, whose identifications did not include any shared tryptic peptides (see Discussion section for a possible interpretation). For the objectives pursued in this work, the clustering of subunits of known protein complexes and paralogs served as an internal control indicating robustness of the analysis method, because a poor reproducibility of the quantitation method would have precluded this outcome. This conclusion was further corroborated by pair-wise Pearson correlation analyses, which returned correlation coefficients close to 1 for all biological replicates (**Fig 3E**). Interestingly, this analysis revealed that the impact of PrP-deficiency on the global proteomes of the respective cell models was not correlated in an obvious way, except for a partial inverse correlation of proteome shifts observed in 1C11 and N2a cells (see below).

Importantly, differences in proteome shifts observed did not appear to have trivial origins related to the methodology that was employed to generate the PrP deficiency. More specifically, distinct proteome shifts were observed even in PrP knockout cells generated with the same CRISPR-Cas9 reagents (i.e., N2a and NMuMG cells), and no differences in steady-state levels between wt and PrP ko cells were observed in a small number of proteins, which we had flagged as possible CRISPR-Cas9 off-target candidates [7].

A closer look at the consequence of PrP-deficiency on the respective global proteomes revealed: (1) PrP-deficiency caused more pronounced shifts to the global proteomes of cells in culture than to the mouse brain. In fact, the brain proteome analysis revealed no protein (with the exception of PrP itself) to have more than a 1.5-fold change in its levels; (2) Steady-state levels of the majority of proteins were not changed in PrP-deficient cells; (3) For most proteins whose steady-state levels were affected by PrP-deficiency, the direction of their level change (up or down) was not consistent across all cell models; (4) With the exception of a small number of proteins, whose steady-state levels were observed to undergo more than twofold changes, effect sizes of PrP-deficiency on steady-state protein levels were small (note that we previously established that the analysis method can detect changes in protein abundance levels as small as 15% when a sufficient number of TMT signature ion profiles accompanying PSMs are available to generate robust statistics) [7].

### PrP-deficiency causes consistent but opposite changes of steady-state levels of MARCKS and its paralog MARCKSL1 in all cell models

**Fig 4:**
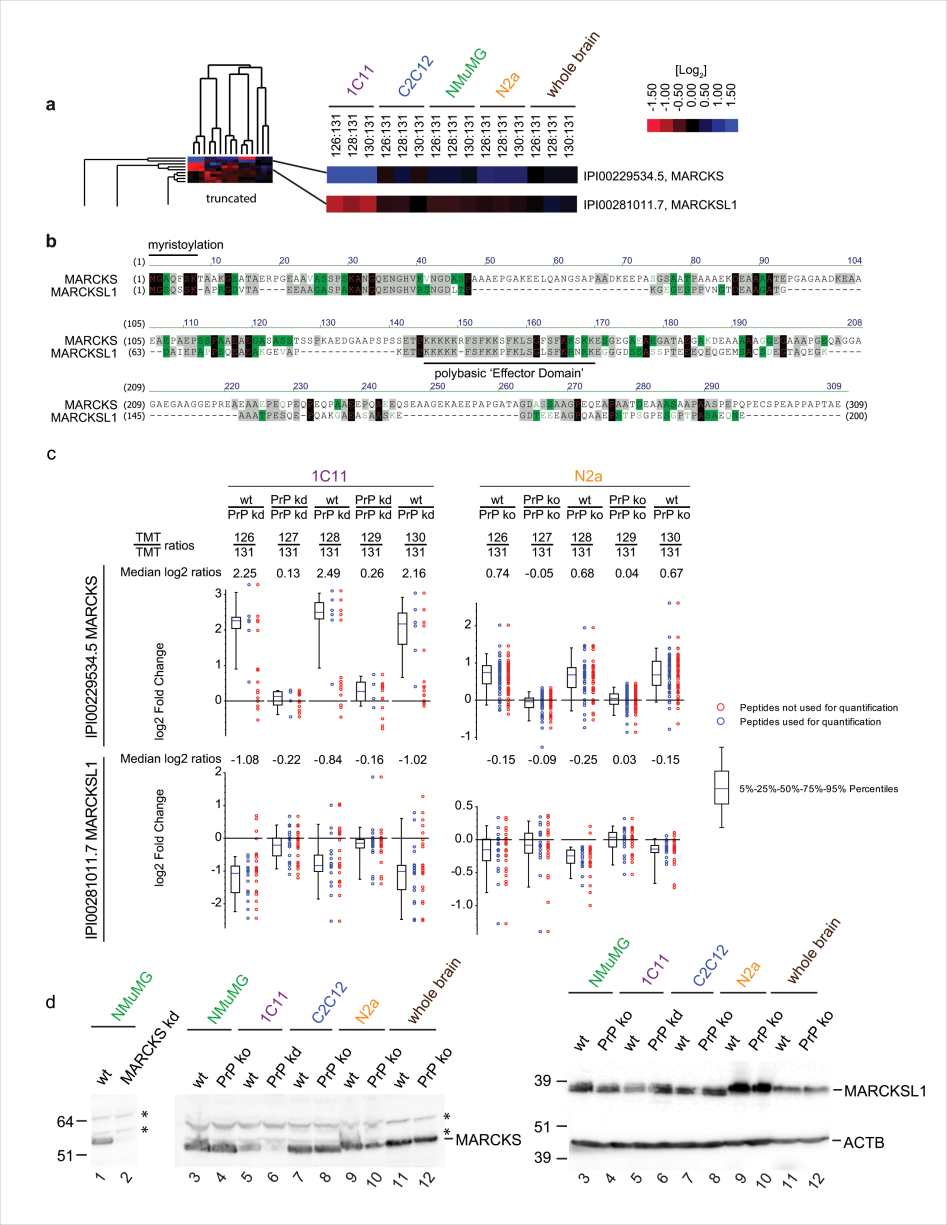
PrP-deficiency causes pronounced and consistent changes to steady-state levels of members of MARCKS protein family. (a) Inverse effect of PrP-deficiency on the relative abundance ratios of MARCKS and MARCKSL1. (b) Sequence alignment of MARCKS and MARCKSL1. (c) Charts depicting the inverse distributions of TMT ratios for peptides assigned to MARCKS and MARCKSL1. Although relative enrichment levels (depicted as log2 ratios) of MARCKS (or MARCKSL1) varied from cell model to cell model, in all cell models tested peptide mixtures obtained from wt cells (labeled with even-numbered TMT reagents) comprised higher (or lower) relative levels of MARCKS (MARCKSL1)-derived peptides than the corresponding peptide mixtures from PrP-deficient cells (labeled with odd-numbered TMT reagents). Note the different ordinate scales on individual plots. The blue dots represent signature ion mass spectra selected for quantitation while the red dots represent those excluded. The largely overlapping distribution of blue and red dots is the consequence of a data processing configuration that relied on two complementary algorithms, Mascot and Sequest, for the assignment of spectra to peptides. To avoid artificial inflation of the number of peptides identified and quantified, the program identifies these occurrences as duplicate hits and rejects one of the replicates when tallying quantified peptides for a given protein. (d) Validation of steady-state expression level changes of MARCKS and MARCKSL1 by Western blot analyses. The even distribution of actin bands across all samples verified successful adjustment of equal protein content by BCA assay. Asterisks designate the presence of a cross-reactive band labeled with the MARCKSL1 antibody that served as an additional internal loading control.

We had anticipated that proteins whose cellular biology is most directly affected by PrP would exhibit the most pronounced and most consistent changes to their abundance levels in PrP-deficient cells. The unsupervised hierarchical clustering drew our attention to a small tree branch comprising MARCKS and MARCKSL1 proteins (**Fig 4A**), two paralogs of the small family of MARCKS proteins sharing 29.1% sequence identity (37% consensus positions), whose steady-state levels were profoundly changed in PrP-deficient cells in several datasets (**Fig 4B**). However, to minimize bias in the selection of proteins-of-interest, two complementary k-means clustering analyses were undertaken: One captured proteins whose log_2_ abundance ratios deviated by ≥ [0.2] in wild-type and PrP-deficient cells in at least nine of the fifteen samples. A total of 66 proteins met this filter criterion, giving rise to 14 clusters (I-XIV) (**S3 Fig**). A second analysis of proteins whose abundance ratios changed in at least one model by log_2_ deviations of ≥ [0.5] returned 74 entries in 13 clusters (I-XIII) (**S4 Fig**). These analyses confirmed the conspicuous effect of PrP-deficiency on MARCKS and MARCKSL1; not only did the TMT signature ion profiles for peptides assigned to these MARCKS family proteins suggest their levels were changed in several of the cell models investigated (**Fig 4C and S3 Fig**) but they also underwent particularly pronounced changes in their relative abundance levels in at least one of the PrP-deficient cell models (**S4 Fig**) (a discriminating characteristic only shared by myosin-9, ferritin light chain 1, microtubule-associated protein 4 and double-stranded RNA-binding protein Staufen homolog 1). Strikingly, however, in 1C11 and N2a cells the direction of change observed for MARCKS correlated directly with the presence of PrP, a result verified by immunoblot analysis, but was opposite for MARCKSL1, whose steady-state levels exhibited an inverse correlation to PrP levels (**Fig 4C** and **4D**).

### The effect of PrP on nerve ending signal hub proteins extends to members of the BASP protein family

In considering possible causes for the disparity of global proteome shifts observed in diverse PrP-deficient cell models, it seemed plausible that PrP’s influence on the global proteome might depend on the cellular differentiation state. For example, the relatively low effect sizes of PrP-deficiency on the global proteomes of NMuMG cells and C2C12 cells (apparent by the relative lack of intensely red or blue colored protein ratio values in columns associated with these cells in **S3 and S4 Figs**) suggested that signaling downstream of PrP that can cause global proteome shifts might be relatively dormant in these cells. In contrast, 1C11 and N2a cells seemed to exhibit relatively stronger proteome shifts in response to PrP-deficiency. To investigate this aspect further, we next waived the requirement of a protein to be quantified in all four cell models, as inclusion of this filter might have masked critical differences between cell models, and asked instead, which of the 4,260 proteins quantified in all datasets underwent the most pronounced changes in their abundance levels in any of the four models (**S5 Fig**). This analysis revealed a total of 56 proteins, whose levels changed more than two-fold on the basis of relative quantitations of a minimum of three unique PSMs. Remarkably, these shortlisted proteins comprised, in addition to the aforementioned MARCKS family proteins, two members of the biochemically and functionally related BASP protein family, brain acid soluble protein 1 and neuromodulin. On the basis of their many conspicuous similarities, members of these protein families have been loosely grouped together and are in the literature referred to as nerve ending signal proteins [24] or GMCs, an acronym formed by the initials of GAP43, MARCKS and CAP23 (BASP1) [25]. GAP43, a protein also known by its alternate names neuromodulin or BASP2, was in this study only detected in C2C12 and N2a cells, with its steady-state levels changing in opposite directions in response to PrP-deficiency i.e., it correlated directly with PrP levels in the C2C12 model and was inversely correlated in N2a cells. Similarly, BASP1 levels underwent two-fold changes in both the 1C11 and N2a cell models and the directionality of this change was again opposite for the two models and inversely correlated to PrP levels in the N2a cell model.

**Fig 5:**
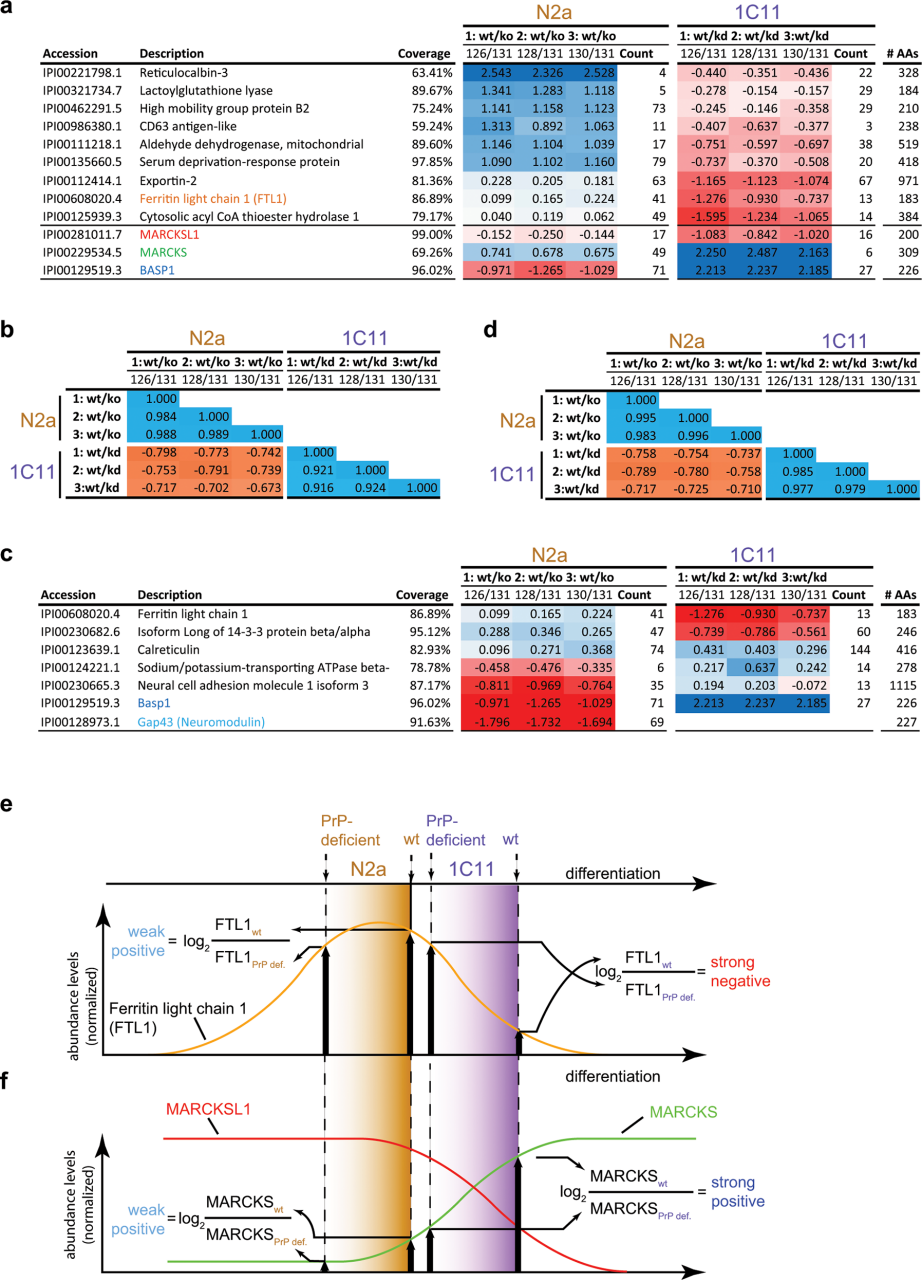
Proteins most affected by PrP-deficiency in N2a and 1C11 cells undergo largely opposite changes in their abundance levels and include MARCKS and BASP signal hub proteins as well as known PrP candidate interactors. (a) Subset of proteins whose steady-state levels changed more than two-fold in PrP deficient N2a and 1C11 cells. Relative protein levels are depicted in log2 space and only robustly quantified protein entries are shown (i.e., ≥ 10 TMT-based quantitations in one model). This short list of proteins comprises three signal hub proteins, i.e., MARCKS, MARCKSL1 and BASP1. (b) Pearson correlation analyses of protein abundance ratios of entries shown in panel ‘a’. (c) The steady-state abundance levels of several previously reported PrP candidate interactors in the N2a cell model are affected by PrP-deficiency [26]. Among them are BASP1 and GAP43, whose levels correlated inversely with levels of PrP (color coded in blue and turquoise font, respectively). (d) Pearson correlation analyses of protein abundance ratio of entries shown in panel ‘b’. (e) Explanatory model consistent with data observed for proteins whose abundance level ratios underwent the most pronounced changes in both cell types (see panel ‘a’ in S6 Fig and text for additional explanations). Abundance level ratios observed for Ferritin light chain 1 (FTL1) in N2a and 1C11 cells serve to explain the model (highlighted in orange color in panels ‘a’ and ‘e’). (f) Extension of model depicted in panel ‘e’ proposing scenario consistent with inverse relationship of PrP-deficiency on MARCKS and MARCKSL1 proteins in N2a and 1C11 cells.

Interestingly, BASP1 was not alone in this regard but the subset of 12 proteins whose steady-state levels had changed in N2a and/or 1C11 more than two-fold and were quantified most robustly (≥ 10 TMT signature ion profiles in at least one of the two models) seemed to exhibit an inverse correlation in their steady-state levels in PrP-deficient cells (**Fig 5A**), supported by a Pearson correlation coefficient of < -0.65 in all pair-wise comparisons of abundance levels ratios (**Fig 5B**). These data raised the question if this inverse correlation of protein abundance levels extends to proteins in the immediate molecular environment of PrP or might reflect downstream events removed from PrP’s direct influence. To address this question, we turned toward a previously reported PrP interactome dataset generated in the N2a cell model [26] and investigated how the relative abundance levels of candidate PrP interactors were affected in the N2a and 1C11 model in response to PrP deficiency. This analysis revealed that candidate PrP interactors were significantly overrepresented amongst the proteins most affected by PrP-deficiency in their steady-state levels (**S3–S5 Figs**), corresponding to a probability of 1.50E-03 (Chi-square = 10.07) if this overlap was due to chance alone. Moreover, the subset of candidate PrP interactors shortlisted in this manner (**Fig 5C**) also shared the aforementioned opposite trend of their abundance levels in the two cell models (with the exception of the protein calreticulin, known to bind to PrP in the endoplasmic reticulum), as evidenced by a relatively high negative Pearson correlation coefficient (**Fig 5D**). This was a puzzling finding that provoked a search for a plausible explanation. It was then noticed that the relationship in abundance levels for these proteins was not, in fact, simply inverse (note, for example, that protein entries whose abundance ratios triggered a dark blue shading in N2a cells did not give rise to a dark red shading of their abundance ratios in the 1C11 cell model, as would be expected for a truly inverse relationship) but manifested in some other way that also generated a highly negative correlation coefficient. A scatter plot analysis of steady-state protein level ratios confirmed that although abundance ratios of proteins in N2a and 1C11 models fell into different quadrants (as expected for an inverse correlation), a strict inverse relationship was incompatible with data observed, as seen by the poor fit of data to the expected trend line for inverse correlations (**S6 Fig,** panel **‘a’**). A much better fit of the same data was observed to trendlines that assumed a reciprocal relationship of abundance ratios of these proteins in response to PrP deficiency in N2a and 1C11 models.

Were opposite trends of PrP deficiency on the proteome perhaps merely a result of differences in steady-state protein levels in the different cell models? To address this question, we analyzed the degree to which the Pearson correlation coefficient for the quantitation among biologically identical samples depended on the spectral counts assigned to proteins. Significantly, this analysis revealed that Pearson correlation coefficients never fell below 0.5 or were negative, as would be expected if limitations in the methodology applied could lead to an erroneous assignment of opposite trends of PrP deficiency on the proteome in cases when such opposite trends do not exist in reality (**S6 Fig,** panel **‘b’**).

In considering possible cell biological scenarios, we noticed that the data can be reconciled in a straightforward model (**Fig 5E**). The model assumes that on account of intrinsic differences in the cellular biology of N2a and 1C11, a differentiation program PrP contributes to might manifest to different degrees in these cells (the term ‘differentiation’ refers in this context to the distinct steady-state characteristics of the cell models compared, which reflects their different origins and lineages, and does not designate the active execution of a differentiation program). Removal of PrP generates phenotypes in which the execution of this differentiation program is less advanced than in the respective wild-type cells. The model further assumes that the steady-state levels of any protein which contributes to this program may at some point during its execution increase, then reach a maximum, and eventually decline. The observed scenario, whereby in two cell models the effect of PrP-deficiency can cause a reciprocal relationship of steady-state protein levels (panel **‘a’** in **S6 Fig**), can be explained if the apex of steady-state levels of a given protein maps to a point along the progression of the differentiation program, in which its completion has more advanced relative to one but not the other cell model. Abundance level ratios observed for Ferritin light chain 1 (FTL1) in N2a and 1C11 cells in this work may serve to explain the model (highlighted in orange color in panels ‘**a**’ and ‘**e**’ in **Fig 5**and panel ‘**a**’ in **S6 Fig)**. In contrast, proteins which undergo equidirectional changes in their abundance level ratios in both cell models (like MARCKS and MARCKSL1), would be expected to exhibit a continuous decline or increase in their abundance levels within the interval of differentiation that is demarked by the respective proteome homeostasis state present in the two cell models (**Fig 5F**).

### MARCKSL1 is a downstream partner in PrP-dependent NCAM1 polysialylation

**Fig 6:**
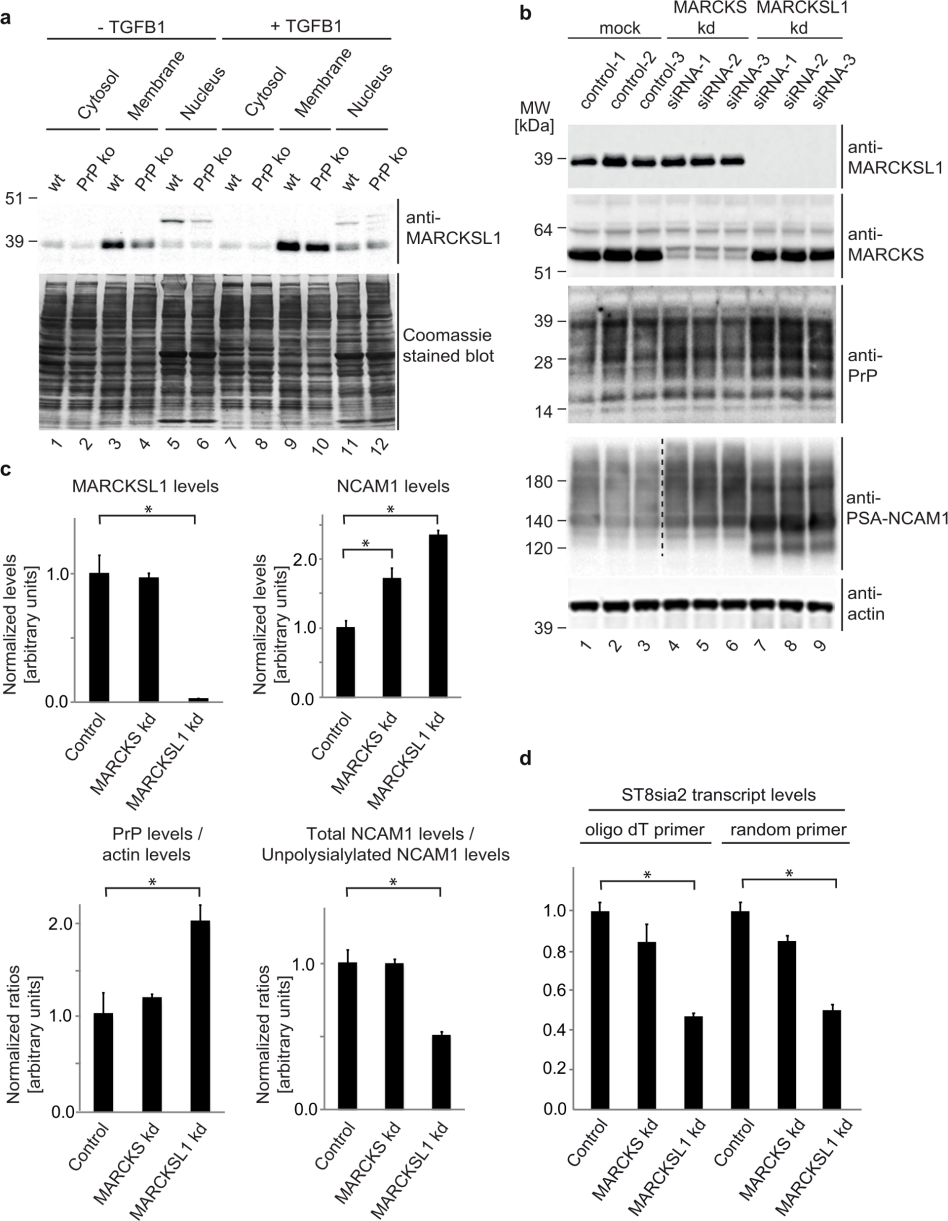
MARCKSL1 contributes to cellular signaling that regulates NCAM1 polysialylation. (a) PrP-deficient NMuMG cells undergoing EMT exhibit reduced levels of membrane-associated MARCKSL1. Western blot analysis of cellular fractions from wild-type (wt) or PrP knockout (ko) NMuMG cells before or after exposure to TGFB1 for 11 h. (b) TGFB1-treated NMuMG cells deficient for MARCKSL1 exhibit a significant upregulation of steady-state protein levels of PrP and NCAM1 but fail to promote NCAM1 polysialylation. (c) Graphs depicting intensity quantitations of protein bands shown in subpanel ‘b’. Asterisks indicate significant protein level (MARCKSL1 and NCAM1) or protein ratio differences (PrP / actin ratios and total NCAM1 / non-polysialylated NCAM1) in direct comparisons (p-value < 0.05). (d) MARCKSL1 deficiency impairs ST8SIA2 transcription following 48 h TGFB1 treatment of NMuMG cells. RT-PCR-based relative quantitation of ST8SIA2 transcript levels (based on oligo dT or random primers) in TGFB1-treated NMuMG cells deficient for MARCKS or MACKSL1. Data were normalized relative to vehicle-treated control cells.

**Fig 7:**
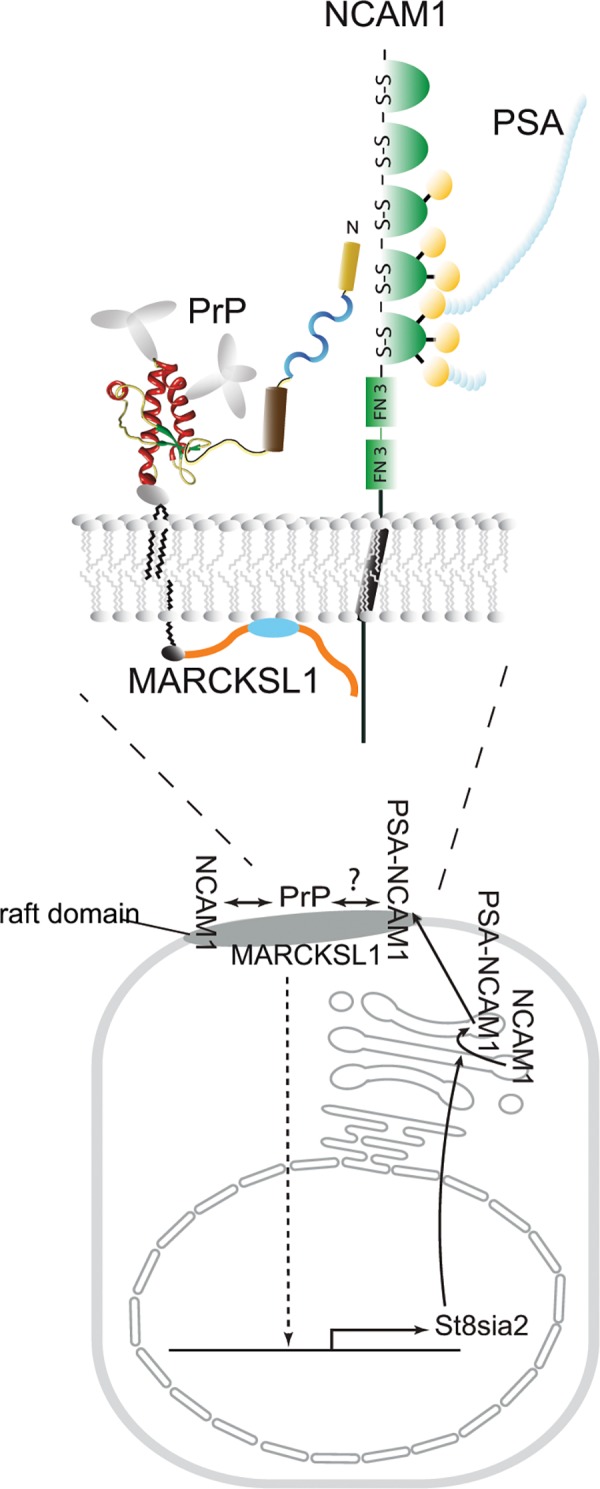
Cartoon depicting MARCKSL1 in a PrP-dependent signaling loop that controls NCAM1 polysialylation in NMuMG cells undergoing EMT. Note that evidence for PrP controlling *St8sia2* gene expression had been previously established [8].

In light of the profound effects of PrP-deficiency on the abundance levels of MARCKS and MARCKSL1, we next addressed if these proteins play a role in the previously established PrP-dependent signaling that controls NCAM1 polysialylation [8]. This phenotype can be observed in NMuMG cells, induced to undergo epithelial-to-mesenchymal transition (EMT), upon TGFB1-addition to the cell culture medium. Because the global proteome analyses presented in this manuscript had been undertaken with undifferentiated NMuMG cells, we first explored if PrP-deficiency leads to changes in the levels of MARCKS family proteins also when NMuMG cells are induced to undergo EMT. Indeed, whereas in this paradigm only a minor change in MARCKS levels was observed in a comparison of TGFB1-treated wild-type versus PrP knockout cells (not shown), levels of cytoplasmic and membrane-bound MARCKSL1 were reduced by more than 50% (**Fig 6A**). To investigate if the reduction in MARCKSL1 levels might mediate the NCAM1 polysialylation deficiency observed in PrP-deficient cells, NMuMG cells were transfected with siRNAs targeting the transcripts coding for MARCKSL1 or MARCKS (serving as a negative control in this paradigm) and protein levels of PrP and levels of NCAM1 polysialylation were compared (**Fig 6B**). Western blotting confirmed the intended knockdown of protein levels of MARCKSL1 (96% reduction) and MARCKS (78% reduction). Interestingly, MARCKSL1 knockdown was observed to lead to a significant increase in protein levels of both NCAM1 and PrP (**Fig 6B**). More strikingly, although these key components of a signaling loop that promotes NCAM1 polysialylation were increased, the MARCKSL1 knockdown did not promote NCAM1 polysialylation but caused its inhibition, apparent by the appearance of the three predominant non-polysialylated NCAM1 isoforms of 120 kDa, 140 kDa and 180 kDa (**Fig 6B** and **6C**). To investigate whether this effect of MARCKSL1 on NCAM1 polysialyation was mediated by a direct effect of MARKCKSL1 on the molecular environment that hosts this post-translational modification or involved reduced transcription of ST8SIA2, the enzyme responsible for the transfer of polysialic acid onto the NCAM1 acceptor glycans, we next undertook an RT-PCR analysis of ST8SIA2 transcript levels in NMuMG cells that were transfected with MARCKSL1 siRNA and induced to convert to a mesenchymal phenotype by 48 h exposure to TGFB1. As negative controls served NMuMG cells tansfected with MARCKS siRNAs or vehicle reagents (**Fig 6D**). This analysis revealed that, akin to the previously reported reduction of ST8SIA2 transcript levels in PrP deficient NMuMG cells [8], the knockdown of MARCKSL1 led to a pronounced reduction in ST8SIA2 transcript levels. Taken together, these data identified MARCKSL1 as a downstream partner in PrP-dependent signaling that contributes to NCAM1 polysialylation in this EMT paradigm (**Fig 7**).

## Discussion

To our knowledge, the current study represents the first investigation to compare side-by-side in several models the consequences of a specific protein deficiency on the global proteome. It made use of a previously optimized workflow that incorporated the tagging of peptides with isobaric reagents to facilitate the direct comparison of samples in a high-end mass spectrometer. The analysis of three biological and three technical replicates from wild-type and PrP-deficient mouse models generated a repository that combines data from >150 hrs of tandem MS analysis time. Post-acquisition investigations of these datasets revealed a profound contribution of the cellular differentiation state to proteome shifts observed. We document that the deficiency of a single protein can affect abundance levels of sub-proteomes in opposite directions in different cell types. The study illustrated that quantitative global proteome analyses can be a powerful complement to target-specific interactome analyses. More specifically, (i) it provided evidence that previously known PrP interactors were significantly enriched amongst the proteins whose abundance levels changed most profoundly in response to PrP-deficiency, and (ii) it provided powerful testament to the conclusion that this type of analysis can reveal non-obvious connections to proteins that contribute to signaling emanating from a protein-of-interest.

### General comments on global proteomics datasets

Several observations we documented in this manuscript stood out and merit commentary:

1. Cluster analyses undertaken in this work tended to group proteins together, which either collaborate as subunits in the assembly of a given protein complex or represent paralogs of each other. While, for the purpose of this work, these observations were primarily used to validate the reproducibility of the methodology, their appearance is interesting and warrants a short explanation: The co-clustering of proteins, which contribute to the assembly of known protein complexes, validated the expectation that proteins known to contribute to the assembly of a given protein complex are frequently co-regulated and stabilized at similar steady-state levels in a given cell; there is ample precedent in the literature to recognize the widespread occurrence of this phenomenon, which constitutes a most economical solution from the perspective of the cellular energy household. The co-clustering of paralogs is easier explained by assuming that they may often retain throughout their separate evolution similarities in aspects of their cellular biology that govern their steady-state levels (e.g., transcriptional regulation, mRNA stability, ribosomal translation, protein interactions, cellular targeting, molecular function, degradation and clearance). Needless to say, we are also aware of many instances where this link between steady-state protein levels of paralogs is broken, for instance, when paralogs have diverged to fulfill mutually exclusive functions and/or exhibit non-overlapping expression patterns. In fact, a relevant case in point represents the expression of PrP and Sho paralogs in the mouse brain [27].
2. A commonly raised request during peer-review is that the biology of a particular protein is investigated in the most relevant biological paradigm. With regard to PrP, this line of thought would lead to the conclusion that the molecular consequences of PrP-deficiency ought to be analyzed in mouse brain tissue. Data presented in this study temper enthusiasm for whole brain global proteome analyses, because PrP-deficiency failed to generate prominent differences in steady-state protein levels in this tissue, thereby extending data from an earlier study, which compared the levels of the most abundant ~350 proteins in wild-type and *Prnp*^-/-^ mice by two-dimensional difference gel electrophoresis [28]. Although it is advisable to proceed with caution in extrapolating general conclusions from specific observations, it seems probable that this outcome is a mere reflection of the relative complexity of the biological source material when conducting tissue-based versus cell-based analyses. A thought experiment of mixing the cellular extracts of the four mouse cell models investigated in this study illustrates this point: because PrP-deficiency affects steady-state levels of a large number of proteins in opposite ways in the individual cell models we investigated, it would be predicted that the pooling of cellular extracts from different cell types would have canceled out and thereby masked effects of PrP knockout on steady-state levels of these proteins in a specific cell type, a phenomenon predicted to be further exacerbated in whole brain analyses.
3. Perhaps the most striking observation in this work was the surprising disparity in the consequences of PrP-deficiency on the global proteomes of distinct cell models. It might be tempting to ascribe these differences to technical issues with the cell models used. However, three of the cell models investigated (NMuMG, C2C12 and N2a) were not only generated by applying an identical CRISRP-Cas9 strategy (which generated indels causing frameshifts in Exon 3) but also were grown in identical cell culture media. Although we cannot formally rule out the possibility that off-target effects of the CRISPR-Cas9 target sequences contributed to this disparity at some level, this explanation is not likely to account for the majority of differences observed. Note that with a view to gauge the influence of off-target effects on the cellular proteome, a previous in-depth global proteome analysis of the same NMuMG clones utilized in this work had been undertaken. In this previous study, the consequences of CRISPR-Cas9-based PrP knockout versus shRNA-based PrP knockdown were compared side-by-side, leading to the conclusion that the method of PrP-deficiency applied had only a minor effect on steady-state protein levels observed [7]. Based on these insights, we propose that the disparity in the effects of PrP-deficiency were largely caused by differences in the differentiation state of the mouse cell models investigated. When considering alternative roles of PrP, for example, a housekeeping function versus a role in a specialized cellular program, the divergent response of the cellular proteomes we observed can be more easily reconciled with the latter scenario. This is because a deficiency in a housekeeping protein would tend to lead to a molecular phenotype that is shared across cell models regardless of their differentiation state. In contrast, interference with the expression of proteins that contribute to specific cellular programs (which may be dormant or active to different degrees in different cell models) would be expected to exhibit effects on a given cellular proteome that reflect the involvement of the specific cellular program in the respective differentiation state of the cell.

### Influence of PrP on steady-state levels of MARCKS family proteins

This study uncovered a profound influence of PrP on steady-state levels of members of the MARCKS and BASP protein families. Members of these families of proteins do not exhibit significant sequence identity but share many physicochemical and functional characteristics [29, 30]. MARCKS was discovered in 1992 when it was shown that it represents a Ca^2+^-dependent protein kinase C (PKC) phosphorylation substrate [31], a characteristic shared by other members of the MARCKS and BASP protein families. All of these proteins lack virtually any secondary structure and are equipped with an N-terminal acylation acceptor site that can be reversibly modified (i.e., by myristoylation for MARCKS, MARCKSL1 and BASP1 and by palmitoylation for GAP43), as well as an internal phosphorylation site domain, that comprises the ‘effector domain’ (ED) (**Fig 4B**). The ED is recognizable by a more or less pronounced cluster of basic residues and represents the domain through which these signaling hub proteins interact in a mutually exclusive manner with PI(4,5)P_2_ [32], the negatively-charged phospholipid bilayer, Ca^2+^/calmodulin or actin. Thus, all of these proteins appear to influence cell fate decisions by binding or detaching from other factors or the membrane in response to their N-terminal acylation and local accumulation of PI(4,5)P_2_, Ca/calmodulin or active PKC [31]. The outcomes of perturbations to this intricate balance of interactions can be dramatic as evidenced by the observation that mice deficient for MARCKS, MACKSL1, BASP1 or GAP43 are nonviable [30, 33–35].

Several characteristics of members of the MARCKS protein family, which are either known from the literature or observed in this work, strengthen their candidacy as molecules that might collaborate in signals that emanate from PrP: (1) MARCKS, MARCKSL1, BASP1 and GAP43 were amongst a small number of proteins, whose levels were not only most dramatically altered in at least one of the cell model studied (**S1 Table and S4 Fig**) but were also most consistently affected by PrP-deficiency in at least three cell models (**S3 Fig**). Interestingly, the effect of PrP deficiency on the MARCKS and MARCKSL1 paralogs tended to be opposite in nature. Thus, whereas PrP deficiency caused a reduction in steady-state protein levels of MARCKS in the N2a and 1C11 cell models, levels of MARCKSL1 were increased in the absence of PrP in these paradigms. The effect size of the PrP-dependent changes observed for individual MARCKS proteins would be predictive of profound effects on cell biology. Note, for instance, that heterozygote *Marcksl1* deficiency is sufficient to generate a low penetrant, yet severe neural tube defect in mice [36], and a mere two-fold overexpression of MARCKS reduced noradrenaline release from neuroblastoma cells by 50%, possibly by promoting actin crosslinking, thereby restricting the movement of dense-cored vesicles carrying this neurotransmitter [37]. (2) A previous PrP interactome study reported the affinity capture of BASP1 and GAP43 together with PrP (**Fig 5B**) [26]. (3) Like PrP, members of the MARCKS protein family are known to localize to raft domains. However, whereas PrP is inserted with its glycosylphosphatidylinositol (GPI)-anchor into the outer leaflet of the plasma membrane, MARCKS family proteins are inserted with their N-terminal fatty acyl group into the inner face of the membrane and further bind to this leaflet through electrostatic interactions of their ED. (4) There is an abundance of data which tie PrP and MARCKS family proteins to cellular programs that affect motility [38], neurite outgrowth and the organization of cellular junctions [39]. (5) The knockout of MARCKSL1 has been shown to cause partially penetrant neural tube closure defects [35, 36], a phenotype independently described for mice lacking normal expression of both PrP and its paralog Sho [40]. We can currently only speculate on how PrP might regulate levels of members of the MARCKS protein family. Because PrP and MARCKS family proteins share residence in raft domains, the impairment of PrP expression may destabilize a subset of these specialized membrane domains, thereby indirectly reducing the stability of MARCKS family proteins. Alternatively, based on the precedent of PrP controlling the transcription of the *St8sia2* gene [8], it also seems plausible that a transcriptional mechanism could underlie the influence of PrP on members of the MARCKS protein family. Regardless of the underlying mechanism, altered MARCKS family protein levels would be expected to influence the local accumulation of PI(4,5)P_2_, Ca/calmodulin or active PKC, thereby potentially compromising the aforementioned cellular programs these signaling molecules contribute to.

### MARCKS family proteins influence biology of PrP-dependent NCAM1 polysialylation and cation-selective channels

A recent report provided evidence for an interaction of MARCKS and BASP1 with polysialylated NCAM1 [41]. Remarkably, the authors concluded that this interaction involves a direct intramembrane interaction of the negatively charged polysialic acid attached to the NCAM1 ectodomain and the positively charged EDs of the respective intracellular MARCKS family members. The functional significance of this interaction, if further corroborated, is not yet understood. One possible explanation could be that it serves the cell to coordinate the assembly of the inner and outer faces of the respective raft domains. Because homopolymers of sialic acid attached to NCAM1 are considerably longer than the ED, there also is a possibility that this interaction might concentrate MARCKS family members, thereby inducing their oligomerization. Indeed, independent data by others suggest that at least BASP1 can be readily induced to adopt quaternary ring-like structures [42] that may convey cation-selective channel properties to the membrane. This observation built on previous data, which established that MARCKS family proteins are quick to acquire SDS-stable oligomeric assemblies. We had previously documented that PrP controls the transcription of the polysialyltransferase ST8SIA2, which in turn polysialylates NCAM1 [8]. Here, we observed that MARCKSL1 similarly influences ST8SIA2 transcription. Taken together, this places PrP at the helm of a signaling loop that not only modulates well-established roles of polysialic acids but also organizes the intracellular face of the signaling hub it is a component of. Intriguingly, the aforementioned scenario also represents yet another alternative mechanism by which PrP may influence cation transport, in addition to widely discussed models that see PrP itself form cation-selective channels [43] or facilitate cation import through molecular interactions with zinc channels [44, 45].

## Conclusions

The current study uncovered a surprising divergence in the consequences of PrP-deficiency in several mouse models, which are routinely used in the PrP research field. The data are consistent with a previously proposed role of PrP in a celI morphogenesis program, whose components are to varying degrees expressed in the specific differentiation states manifested in these models. The data emphasize the usefulness of cell-based over tissue-based analysis for studying signaling downstream of PrP and should be a warning to exercise caution when passing judgment on the veracity of non-overlapping molecular phenotypes generated in different cell models. Careful comparative studies that dissect PrP’s molecular interactions and the signals emanating from them in several cell models may be the way forward to define its molecular function.

## Supporting Information

S1 Fig Relative distributions of proteins identified in the five global proteome datasets across GO ‘Molecular Functions’ and ‘Biological Process’ categories. (PDF)

S2 Fig Hierarchical clustering of proteins quantified on the basis of a minimum of three TMT signature ion distributions in all cell models. (PDF)

S3 Fig Proteins quantified in all cell models and exhibiting PrP-dependent changes in their abundance ratios of > [0.2] (log_2_) in more than nine samples. (PDF)

S4 Fig Proteins quantified in all cell models and exhibiting PrP-dependent changes in their abundance ratios of > [0.5] (log_2_) in more than three samples. (PDF)

S5 Fig Proteins exhibiting PrP-dependent changes in their abundance ratios of > [1.0] (log_2_) in three samples. (PDF)

S6 Fig Graphical evidence that abundance level ratios for proteins undergoing robust ≥ two-fold changes in response to PrP-deficiency are neither simply inverted in N2a and 1C11 cell models nor a consequence of mere differences in protein abundance levels. (a) Comparison of abundance fold change for 13 proteins observed in N2a and 1C11 preparations. The proteins shown are the best known binding partners of PrP and/or changed the most in their abundance with PrP expression. Each protein is represented by five data points with a single x-coordinate corresponding to its median Log2 126/131 fold change in N2a cells. The median Log2 reporter ion ratios 126/131, 128/131 and 130/131 for 1C11 as well as the median Log2 reporter ion ratios 128/131 and 130/131 for N2a constitute the five y-coordinates for each protein. The dashed black vertical line transects all five values for ferritin light chain 1. The dashed pink diagonal line traversing quadrants II and IV shows the trend in reporter ion ratios which would be observed if the abundance profiles of the 12 proteins in 1C11 and N2a had an inverse relationship. The relative abundance of these proteins in wild-type and PrP deficient versions of the two cell models (represented by TMT ratios) follows a reciprocal function as plotted in Quadrants II and IV. From left to right: neural cell adhesion molecule 1, sodium/potassium-transporting ATPase subunit beta-3, secernin-1, cytosolic acyl coenzyme A thioester hydrolase isoform 1, ferritin light chain 1, exportin-2, isoform Long of 14-3-3 protein beta/alpha, serum deprivation-response protein, high mobility group protein B2, aldehyde dehydrogenase, CD63 antigen-like, lactoylglutathione lyase, reticulocalbin-3. (b) Proteins quantified in NMuMG cells were sorted by the number of quantifications per protein and secondly by the number of spectral counts per protein (both indicators of relative protein abundance), then segregated in bins of 125 proteins. Pearson correlation coefficients for median protein reporter ion ratios from all biological replicates were calculated for each bin.
(PDF)

S1 Table Abundance level ratios of proteins detected in the five mouse models on the basis of a minimum of three TMT reporter ion ratios. (PDF)

## Abbreviations

CID: collision-induced dissociation
ED: effector domain
EMT: epithelial-to-mesenchymal transition
IPI: international protein index
FTL1: ferritin light chain 1
GO: gene ontology
kd: knockdown
ko: knockout
FDR: false discovery rate
HMM: high molecular mass
IF: intermediate filaments
MARCKS: myristoylated alanine-rich C kinase substrate
MS/MS: tandem mass spectrometry
NCAM: neural cell adhesion molecule
ppm: parts per million
PKC: protein kinase C
PrP: prion protein
PSM: peptide-to-spectrum matches
TMT: tandem mass tag
wt: wild-type
