# Supplementary figures and images for: Prion Protein Deficiency Causes Diverse Proteome Shifts in Cell Models That Escape Detection in Brain Tissue
Source: PLoS One. 2016 Jun 21;11(6):e0156779. doi: 10.1371/journal.pone.0156779 (PMC4915660; doi:10.1371/journal.pone.0156779)

S1 Figure

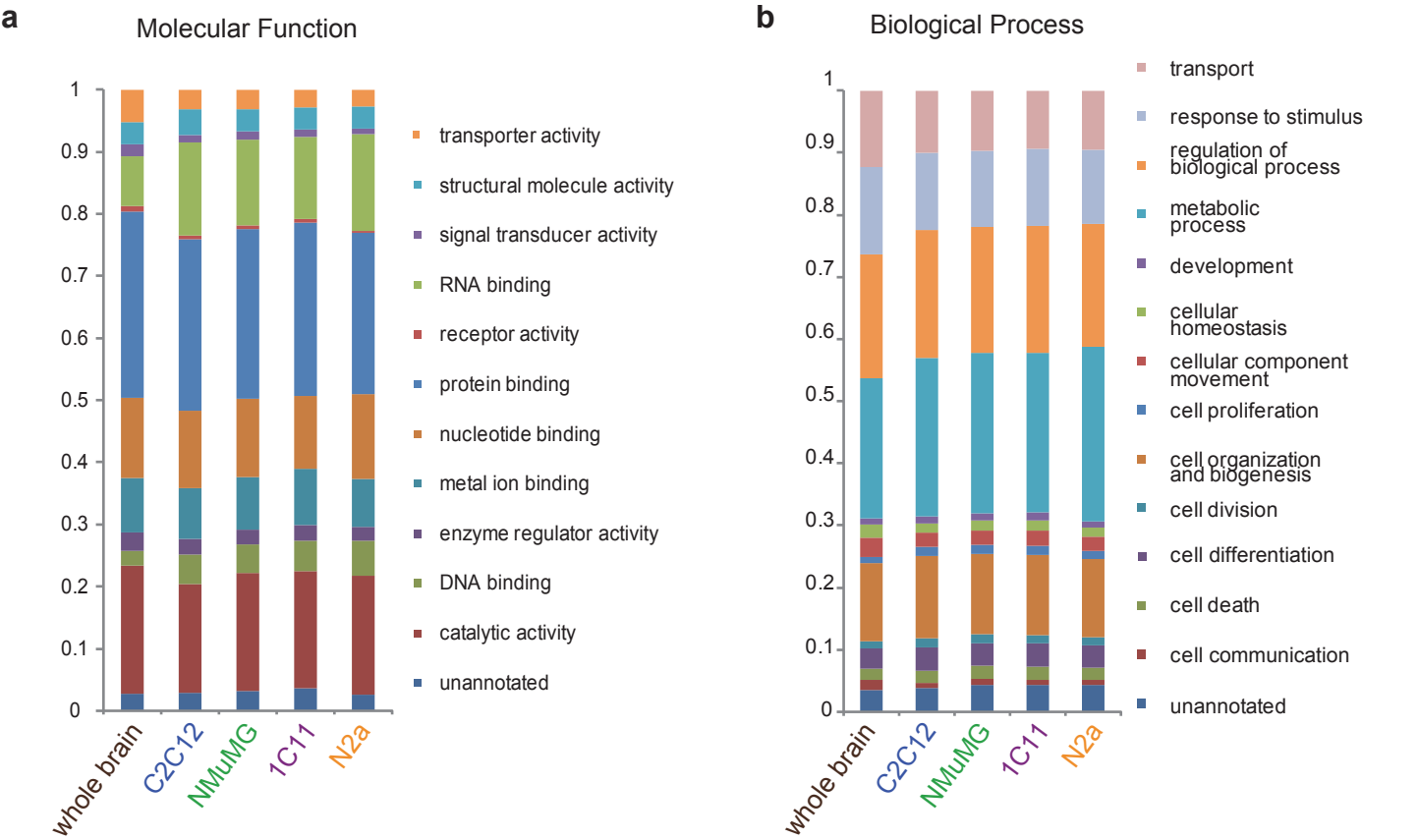

Supplement: S1 Fig — (PDF) [file pone.0156779.s001.pdf]

## S6 Figure

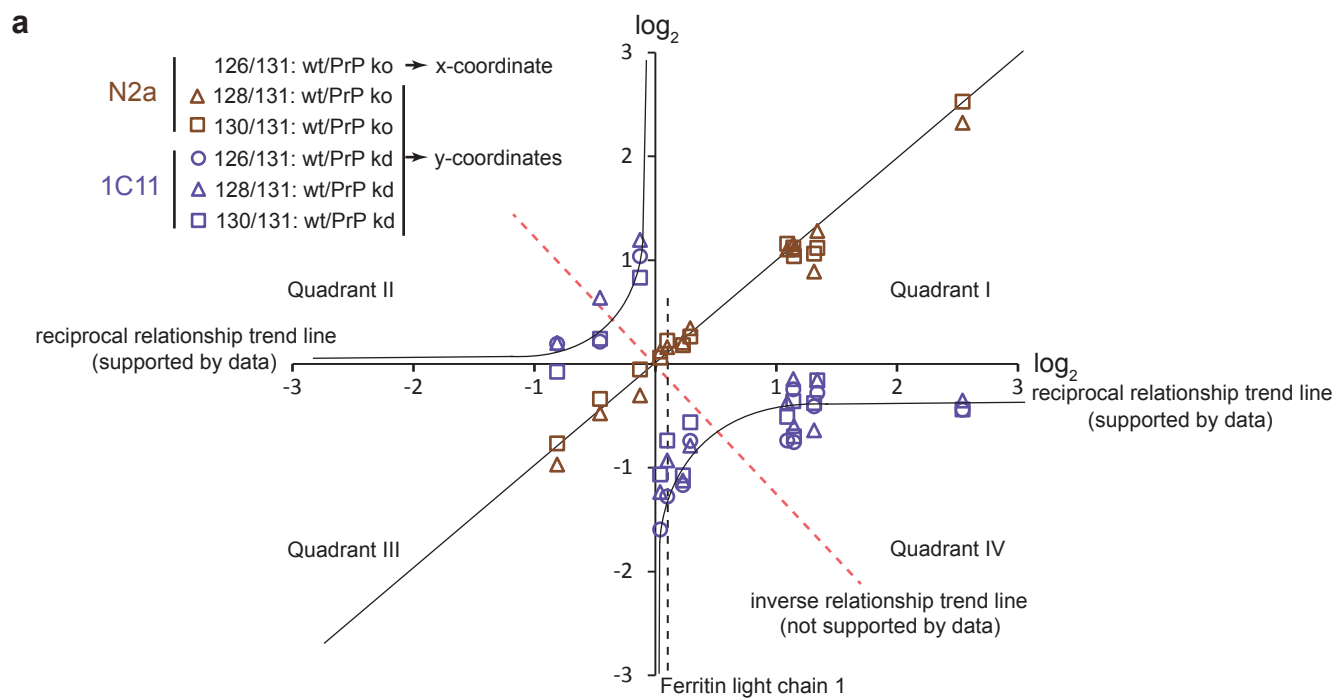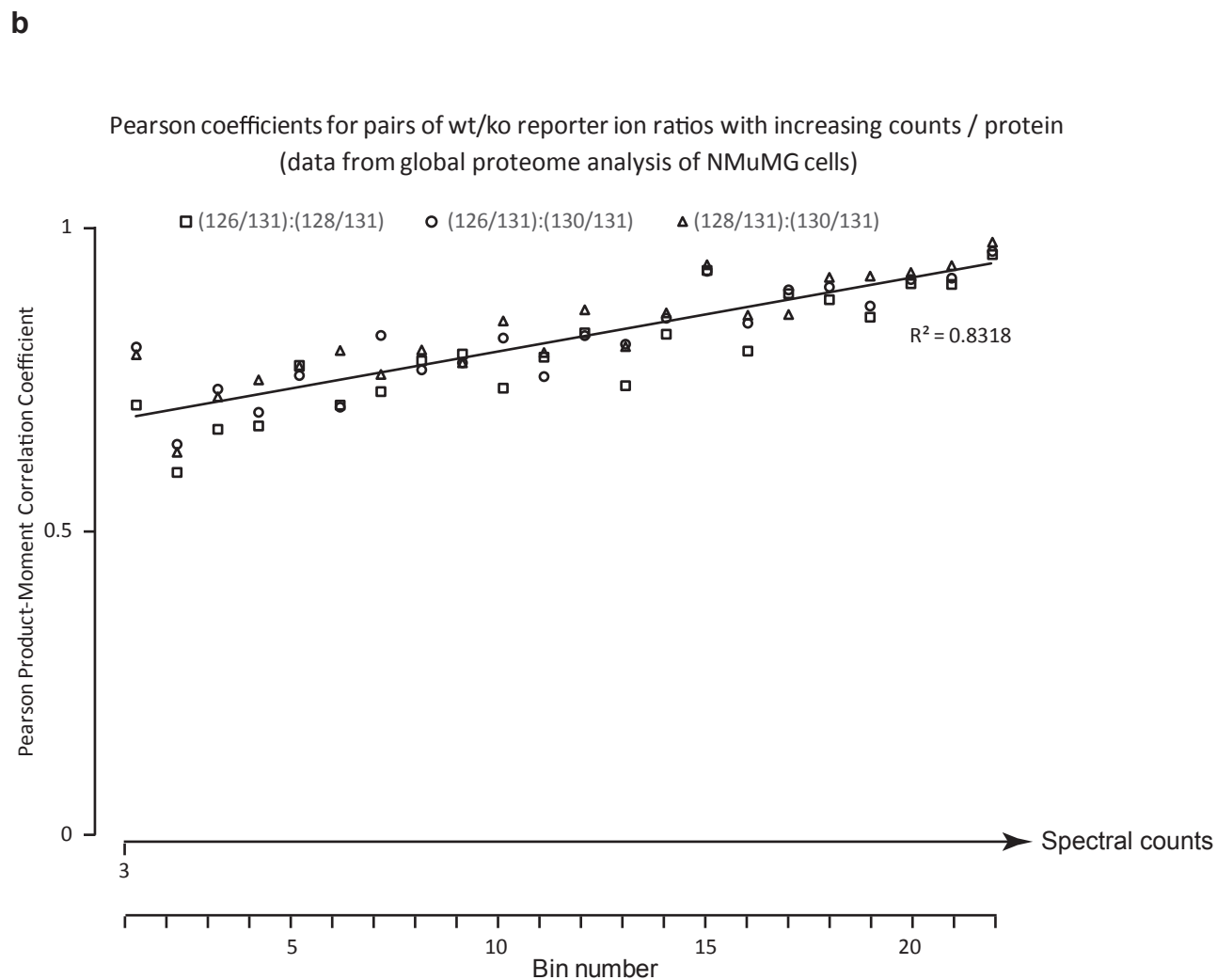

Supplement: S6 Fig — (a) Comparison of abundance fold change for 13 proteins observed in N2a and 1C11 preparations. The proteins shown are the best known binding partners of PrP and/or changed the most in their abundance with PrP expression. Each protein is represented by five data points with a single x-coordinate corresponding to its median Log2 126/131 fold change in N2a cells. The median Log2 reporter ion ratios 126/131, 128/131 and 130/131 for 1C11 as well as the median Log2 reporter ion ratios 128/131 and 130/131 for N2a constitute the five y-coordinates for each protein. The dashed black vertical line transects all five values for ferritin light chain 1. The dashed pink diagonal line traversing quadrants II and IV shows the trend in reporter ion ratios which would be observed if the abundance profiles of the 12 proteins in 1C11 and N2a had an inverse relationship. The relative abundance of these proteins in wild-type and PrP deficient versions of the two cell models (represented by TMT ratios) follows a reciprocal function as plotted in Quadrants II and IV. From left to right: neural cell adhesion molecule 1, sodium/potassium-transporting ATPase subunit beta-3, secernin-1, cytosolic acyl coenzyme A thioester hydrolase isoform 1, ferritin light chain 1, exportin-2, isoform Long of 14-3-3 protein beta/alpha, serum deprivation-response protein, high mobility group protein B2, aldehyde dehydrogenase, CD63 antigen-like, lactoylglutathione lyase, reticulocalbin-3. (b) Proteins quantified in NMuMG cells were sorted by the number of quantifications per protein and secondly by the number of spectral counts per protein (both indicators of relative protein abundance), then segregated in bins of 125 proteins. Pearson correlation coefficients for median protein reporter ion ratios from all biological replicates were calculated for each bin. (PDF) [file pone.0156779.s006.pdf]
