# Supplementary material for: Prion Protein Deficiency Causes Diverse Proteome Shifts in Cell Models That Escape Detection in Brain Tissue
Source: PLoS One. 2016 Jun 21;11(6):e0156779. doi: 10.1371/journal.pone.0156779 (PMC4915660; doi:10.1371/journal.pone.0156779)
Supplement: S2 Fig — (PDF) [file pone.0156779.s002.pdf]

S2 Figure

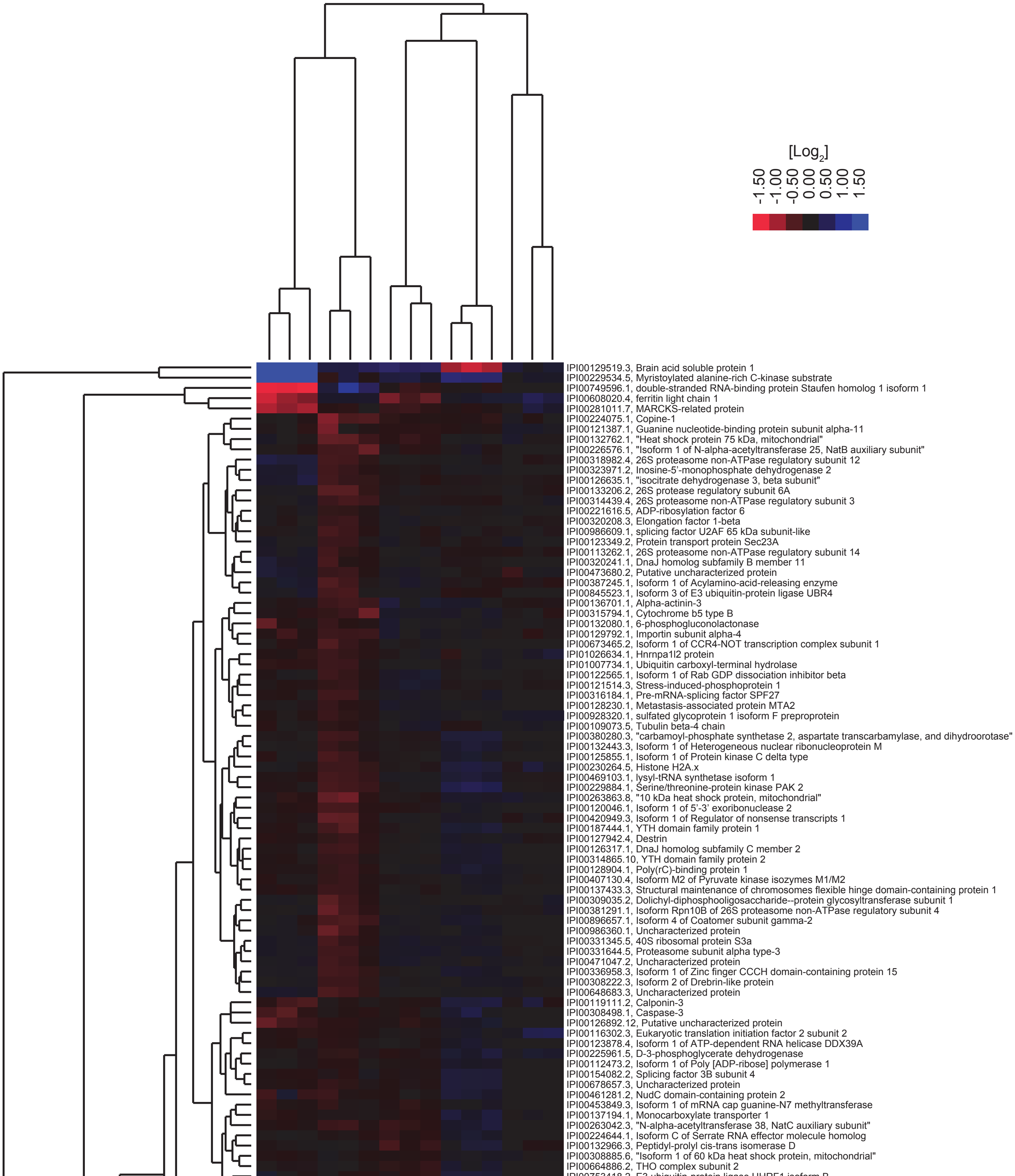
[Log2]
-1.50
-1.00
-0.50
0.00
0.50
1.00
1.50
IPI00129519.3, Brain acid soluble protein 1
IPI00229534.5, Myristoylated alanine-rich C-kinase substrate
IPI00749596.1, double-stranded RNA-binding protein Staufen homolog 1 isoform 1
IPI00608020.4, ferritin light chain 1
IPI00281011.7, MARCKS-related protein
IPI00224075.1, Copine-1
IPI00121387.1, Guanine nucleotide-binding protein subunit alpha-11
IPI00132762.1, "Heat shock protein 75 kDa, mitochondrial"
IPI00226576.1, "Isoform 1 of N-alpha-acetyltransferase 25, NatB auxiliary subunit"
IPI00318982.4, 26S proteasome non-ATPase regulatory subunit 12
IPI00323971.2, Inosine-5’-monophosphate dehydrogenase 2
IPI00126635.1, "isocitrate dehydrogenase 3, beta subunit"
IPI00133206.2, 26S protease regulatory subunit 6A
IPI00314439.4, 26S proteasome non-ATPase regulatory subunit 3
IPI00221616.5, ADP-ribosylation factor 6
IPI00320208.3, Elongation factor 1-beta
IPI00986609.1, splicing factor U2AF 65 kDa subunit-like
IPI00123349.2, Protein transport protein Sec23A
IPI00113262.1, 26S proteasome non-ATPase regulatory subunit 14
IPI00320241.1, DnaJ homolog subfamily B member 11
IPI00473680.2, Putative uncharacterized protein
IPI00387245.1, Isoform 1 of Acylamino-acid-releasing enzyme
IPI00845523.1, Isoform 3 of E3 ubiquitin-protein ligase UBR4
IPI00136701.1, Alpha-actinin-3
IPI00315794.1, Cytochrome b5 type B
IPI00132080.1, 6-phosphogluconolactonase
IPI00129792.1, Importin subunit alpha-4
IPI00673465.2, Isoform 1 of CCR4-NOT transcription complex subunit 1
IPI01026634.1, Hnrnpa1l2 protein
IPI01007734.1, Ubiquitin carboxyl-terminal hydrolase
IPI00122565.1, Isoform 1 of Rab GDP dissociation inhibitor beta
IPI00121514.3, Stress-induced-phosphoprotein 1
IPI00316184.1, Pre-mRNA-splicing factor SPF27
IPI00128230.1, Metastasis-associated protein MTA2
IPI00928320.1, sulfated glycoprotein 1 isoform F preproprotein
IPI00109073.5, Tubulin beta-4 chain
IPI00380280.3, "carbamoyl-phosphate synthetase 2, aspartate transcarbamylase, and dihydroorotase"
IPI00132443.3, Isoform 1 of Heterogeneous nuclear ribonucleoprotein M
IPI00125855.1, Isoform 1 of Protein kinase C delta type
IPI00230264.5, Histone H2A.x
IPI00469103.1, lysyl-tRNA synthetase isoform 1
IPI00229884.1, Serine/threonine-protein kinase PAK 2
IPI00263863.8, "10 kDa heat shock protein, mitochondrial"
IPI00120046.1, Isoform 1 of 5’-3’ exoribonuclease 2
IPI00420949.3, Isoform 1 of Regulator of nonsense transcripts 1
IPI00187444.1, YTH domain family protein 1
IPI00127942.4, Destrin
IPI00126317.1, DnaJ homolog subfamily C member 2
IPI00314865.10, YTH domain family protein 2
IPI00128904.1, Poly(rC)-binding protein 1
IPI00407130.4, Isoform M2 of Pyruvate kinase isozymes M1/M2
IPI00137433.3, Structural maintenance of chromosomes flexible hinge domain-containing protein 1
IPI00309035.2, Dolichyl-diphosphooligosaccharide--protein glycosyltransferase subunit 1
IPI00381291.1, Isoform Rpn10B of 26S proteasome non-ATPase regulatory subunit 4
IPI00896657.1, Isoform 4 of Coatomer subunit gamma-2
IPI00986360.1, Uncharacterized protein
IPI00331345.5, 40S ribosomal protein S3a
IPI00331644.5, Proteasome subunit alpha type-3
IPI00471047.2, Uncharacterized protein
IPI00336958.3, Isoform 1 of Zinc finger CCCH domain-containing protein 15
IPI00308222.3, Isoform 2 of Drebrin-like protein
IPI00648683.3, Uncharacterized protein
IPI00119111.2, Calponin-3
IPI00308498.1, Caspase-3
IPI00126892.12, Putative uncharacterized protein
IPI00116302.3, Eukaryotic translation initiation factor 2 subunit 2
IPI00123878.4, Isoform 1 of ATP-dependent RNA helicase DDX39A
IPI00225961.5, D-3-phosphoglycerate dehydrogenase
IPI00112473.2, Isoform 1 of Poly [ADP-ribose] polymerase 1
IPI00154082.2, Splicing factor 3B subunit 4
IPI00678657.3, Uncharacterized protein
IPI00461281.2, NudC domain-containing protein 2
IPI00453849.3, Isoform 1 of mRNA cap guanine-N7 methyltransferase
IPI00137194.1, Monocarboxylate transporter 1
IPI00263042.3, "N-alpha-acetyltransferase 38, NatC auxiliary subunit"
IPI00224644.1, Isoform C of Serrate RNA effector molecule homolog
IPI00132966.3, Peptidyl-prolyl cis-trans isomerase D
IPI00308885.6, "Isoform 1 of 60 kDa heat shock protein, mitochondrial"
IPI00664886.2, THO complex subunit 2

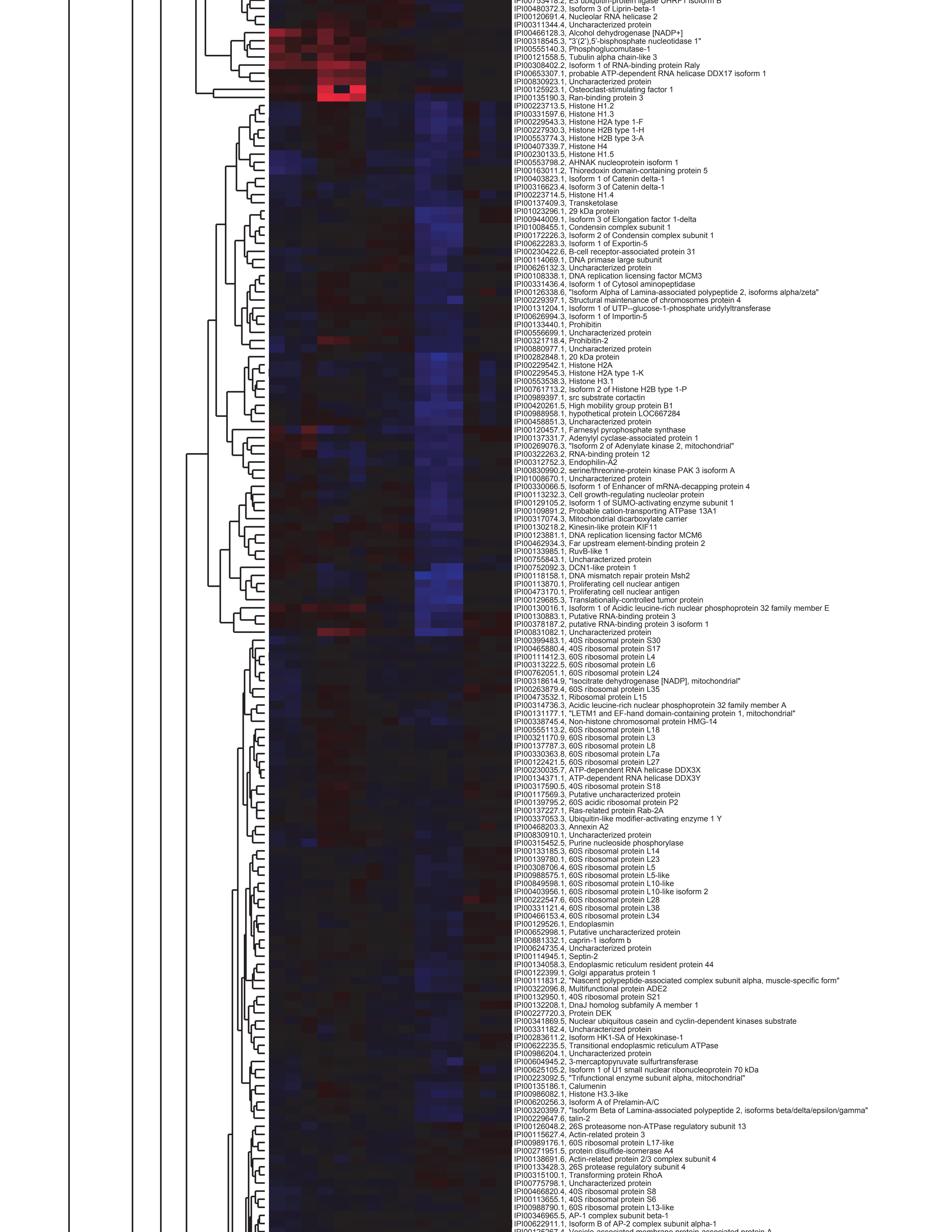
IPI00480372.3, Isoform 3 of Liprin-beta-1
IPI00120691.4, Nucleolar RNA helicase 2
IPI00311344.4, Uncharacterized protein
IPI00466128.3, Alcohol dehydrogenase [NADP+]
IPI00318545.3, "3'(2'),5'-bisphosphate nucleotidase 1"
IPI00555140.3, Phosphoglucomutase-1
IPI00121558.5, Tubulin alpha chain-like 3
IPI00308402.2, Isoform 1 of RNA-binding protein Raly
IPI00653307.1, probable ATP-dependent RNA helicase DDX17 isoform 1
IPI00830923.1, Uncharacterized protein
IPI00125923.1, Osteoclast-stimulating factor 1
IPI00135190.3, Ran-binding protein 3
IPI00223713.5, Histone H1.2
IPI00331597.6, Histone H1.3
IPI00229543.3, Histone H2A type 1-F
IPI00227930.3, Histone H2B type 1-H
IPI00553774.3, Histone H2B type 3-A
IPI00407339.7, Histone H4
IPI00230133.5, Histone H1.5
IPI00553798.2, AHNAK nucleoprotein isoform 1
IPI00163011.2, Thioredoxin domain-containing protein 5
IPI00403823.1, Isoform 1 of Catenin delta-1
IPI00316623.4, Isoform 3 of Catenin delta-1
IPI00223714.5, Histone H1.4
IPI00137409.3, Transketolase
IPI01023296.1, 29 kDa protein
IPI00944009.1, Isoform 3 of Elongation factor 1-delta
IPI01008455.1, Condensin complex subunit 1
IPI00172226.3, Isoform 2 of Condensin complex subunit 1
IPI00622283.3, Isoform 1 of Exportin-5
IPI00230422.6, B-cell receptor-associated protein 31
IPI00114069.1, DNA primase large subunit
IPI00626132.3, Uncharacterized protein
IPI00108338.1, DNA replication licensing factor MCM3
IPI00331436.4, Isoform 1 of Cytosol aminopeptidase
IPI00126338.6, "Isoform Alpha of Lamina-associated polypeptide 2, isoforms alpha/zeta"
IPI00229397.1, Structural maintenance of chromosomes protein 4
IPI00131204.1, Isoform 1 of UTP--glucose-1-phosphate uridylyltransferase
IPI00626994.3, Isoform 1 of Importin-5
IPI00133440.1, Prohibitin
IPI00556699.1, Uncharacterized protein
IPI00321718.4, Prohibitin-2
IPI00880977.1, Uncharacterized protein
IPI00282848.1, 20 kDa protein
IPI00229542.1, Histone H2A
IPI00229545.3, Histone H2A type 1-K
IPI00553538.3, Histone H3.1
IPI00761713.2, Isoform 2 of Histone H2B type 1-P
IPI00989397.1, src substrate cortactin
IPI00420261.5, High mobility group protein B1
IPI00988958.1, hypothetical protein LOC667284
IPI00458851.3, Uncharacterized protein
IPI00120457.1, Farnesyl pyrophosphate synthase
IPI00137331.7, Adenylyl cyclase-associated protein 1
IPI00269076.3, "Isoform 2 of Adenylate kinase 2, mitochondrial"
IPI00322263.2, RNA-binding protein 12
IPI00312752.3, Endophilin-A2
IPI00830990.2, serine/threonine-protein kinase PAK 3 isoform A
IPI01008670.1, Uncharacterized protein
IPI00330066.5, Isoform 1 of Enhancer of mRNA-decapping protein 4
IPI00113232.3, Cell growth-regulating nucleolar protein
IPI00129105.2, Isoform 1 of SUMO-activating enzyme subunit 1
IPI00109891.2, Probable cation-transporting ATPase 13A1
IPI00317074.3, Mitochondrial dicarboxylate carrier
IPI00130218.2, Kinesin-like protein KIF11
IPI00123881.1, DNA replication licensing factor MCM6
IPI00462934.3, Far upstream element-binding protein 2
IPI00133985.1, RuvB-like 1
IPI00755843.1, Uncharacterized protein
IPI00752092.3, DCN1-like protein 1
IPI00118158.1, DNA mismatch repair protein Msh2
IPI00113870.1, Proliferating cell nuclear antigen
IPI00473170.1, Proliferating cell nuclear antigen
IPI00129685.3, Translationally-controlled tumor protein
IPI00130016.1, Isoform 1 of Acidic leucine-rich nuclear phosphoprotein 32 family member E
IPI00130883.1, Putative RNA-binding protein 3
IPI00378187.2, putative RNA-binding protein 3 isoform 1
IPI00831082.1, Uncharacterized protein
IPI00399483.1, 40S ribosomal protein S30
IPI00465880.4, 40S ribosomal protein S17
IPI00111412.3, 60S ribosomal protein L4
IPI00313222.5, 60S ribosomal protein L6
IPI00762051.1, 60S ribosomal protein L24
IPI00318614.9, "Isocitrate dehydrogenase [NADP], mitochondrial"
IPI00263879.4, 60S ribosomal protein L35
IPI00473532.1, Ribosomal protein L15
IPI00314736.3, Acidic leucine-rich nuclear phosphoprotein 32 family member A
IPI00131177.1, "LETM1 and EF-hand domain-containing protein 1, mitochondrial"
IPI00338745.4, Non-histone chromosomal protein HMG-14
IPI00555113.2, 60S ribosomal protein L18
IPI00321170.9, 60S ribosomal protein L3
IPI00137787.3, 60S ribosomal protein L8
IPI00330363.8, 60S ribosomal protein L7a
IPI00122421.5, 60S ribosomal protein L27
IPI00230035.7, ATP-dependent RNA helicase DDX3X
IPI00134371.1, ATP-dependent RNA helicase DDX3Y
IPI00317590.5, 40S ribosomal protein S18
IPI00117569.3, Putative uncharacterized protein
IPI00139795.2, 60S acidic ribosomal protein P2
IPI00137227.1, Ras-related protein Rab-2A
IPI00337053.3, Ubiquitin-like modifier-activating enzyme 1 Y
IPI00468203.3, Annexin A2
IPI00830910.1, Uncharacterized protein
IPI00315452.5, Purine nucleoside phosphorylase
IPI00133185.3, 60S ribosomal protein L14
IPI00139780.1, 60S ribosomal protein L23
IPI00308706.4, 60S ribosomal protein L5
IPI00988575.1, 60S ribosomal protein L5-like
IPI00849598.1, 60S ribosomal protein L10-like
IPI00403956.1, 60S ribosomal protein L10-like isoform 2
IPI00222547.6, 60S ribosomal protein L28
IPI00331121.4, 60S ribosomal protein L38
IPI00466153.4, 60S ribosomal protein L34
IPI00129526.1, Endoplasmin
IPI00652998.1, Putative uncharacterized protein
IPI00881332.1, caprin-1 isoform b
IPI00624735.4, Uncharacterized protein
IPI00114945.1, Septin-2
IPI00134058.3, Endoplasmic reticulum resident protein 44
IPI00122399.1, Golgi apparatus protein 1
IPI00111831.2, "Nascent polypeptide-associated complex subunit alpha, muscle-specific form"
IPI00322096.8, Multifunctional protein ADE2
IPI00132950.1, 40S ribosomal protein S21
IPI00132208.1, DnaJ homolog subfamily A member 1
IPI00227720.3, Protein DEK
IPI00341869.5, Nuclear ubiquitous casein and cyclin-dependent kinases substrate
IPI00331182.4, Uncharacterized protein
IPI00283611.2, Isoform HK1-SA of Hexokinase-1
IPI00622235.5, Transitional endoplasmic reticulum ATPase
IPI00986204.1, Uncharacterized protein
IPI00604945.2, 3-mercaptopyruvate sulfurtransferase
IPI00625105.2, Isoform 1 of U1 small nuclear ribonucleoprotein 70 kDa
IPI00223092.5, "Trifunctional enzyme subunit alpha, mitochondrial"
IPI00135186.1, Calumenin
IPI00986082.1, Histone H3.3-like
IPI00620256.3, Isoform A of Prelamin-A/C
IPI00320399.7, "Isoform Beta of Lamina-associated polypeptide 2, isoforms beta/delta/epsilon/gamma"
IPI00229647.6, talin-2
IPI00126048.2, 26S proteasome non-ATPase regulatory subunit 13
IPI00115627.4, Actin-related protein 3
IPI00989176.1, 60S ribosomal protein L17-like
IPI00271951.5, protein disulfide-isomerase A4
IPI00138691.6, Actin-related protein 2/3 complex subunit 4
IPI00133428.3, 26S protease regulatory subunit 4
IPI00315100.1, Transforming protein RhoA
IPI00775798.1, Uncharacterized protein
IPI00466820.4, 40S ribosomal protein S8
IPI00113655.1, 40S ribosomal protein S6
IPI00988790.1, 60S ribosomal protein L13-like
IPI00346965.5, AP-1 complex subunit beta-1
IPI00622911.1, Isoform B of AP-2 complex subunit alpha-1

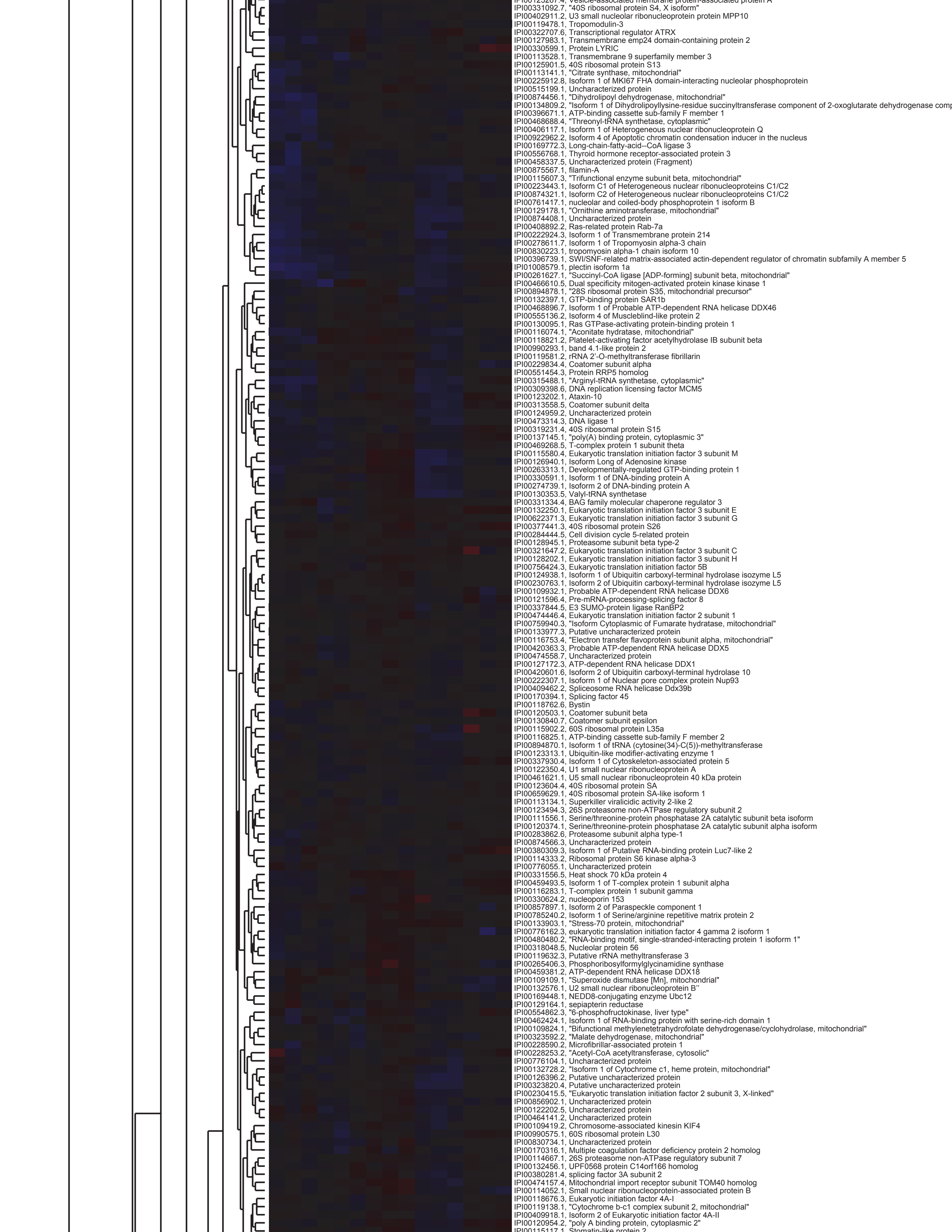
IPI00123287.4, Vesicle-associated membrane protein-associated protein A
IPI00331092.7, "40S ribosomal protein S4, X isoform"
IPI00402911.2, U3 small nucleolar ribonucleoprotein protein MPP10
IPI00119478.1, Tropomodulin-3
IPI00322707.6, Transcriptional regulator ATRX
IPI00127983.1, Transmembrane emp24 domain-containing protein 2
IPI00330599.1, Protein LYRIC
IPI00113528.1, Transmembrane 9 superfamily member 3
IPI00125901.5, 40S ribosomal protein S13
IPI00113141.1, "Citrate synthase, mitochondrial"
IPI00225912.8, Isoform 1 of MKI67 FHA domain-interacting nucleolar phosphoprotein
IPI00515199.1, Uncharacterized protein
IPI00874456.1, "Dihydrolipoyl dehydrogenase, mitochondrial"
IPI00134809.2, "Isoform 1 of Dihydrolipoyllysine-residue succinyltransferase component of 2-oxoglutarate dehydrogenase comp
IPI00396671.1, ATP-binding cassette sub-family F member 1
IPI00468688.4, "Threonyl-tRNA synthetase, cytoplasmic"
IPI00406117.1, Isoform 1 of Heterogeneous nuclear ribonucleoprotein Q
IPI00922962.2, Isoform 4 of Apoptotic chromatin condensation inducer in the nucleus
IPI00169772.3, Long-chain-fatty-acid--CoA ligase 3
IPI00556768.1, Thyroid hormone receptor-associated protein 3
IPI00458337.5, Uncharacterized protein (Fragment)
IPI00875567.1, filamin-A
IPI00115607.3, "Trifunctional enzyme subunit beta, mitochondrial"
IPI00223443.1, Isoform C1 of Heterogeneous nuclear ribonucleoproteins C1/C2
IPI00874321.1, Isoform C2 of Heterogeneous nuclear ribonucleoproteins C1/C2
IPI00761417.1, nucleolar and coiled-body phosphoprotein 1 isoform B
IPI00129178.1, "Ornithine aminotransferase, mitochondrial"
IPI00874408.1, Uncharacterized protein
IPI00408892.2, Ras-related protein Rab-7a
IPI00222924.3, Isoform 1 of Transmembrane protein 214
IPI00278611.7, Isoform 1 of Tropomyosin alpha-3 chain
IPI00830223.1, tropomyosin alpha-1 chain isoform 10
IPI00396739.1, SWI/SNF-related matrix-associated actin-dependent regulator of chromatin subfamily A member 5
IPI01008579.1, plectin isoform 1a
IPI00261627.1, "Succinyl-CoA ligase [ADP-forming] subunit beta, mitochondrial"
IPI00466610.5, Dual specificity mitogen-activated protein kinase kinase 1
IPI00894878.1, "28S ribosomal protein S35, mitochondrial precursor"
IPI00132397.1, GTP-binding protein SAR1b
IPI00468896.7, Isoform 1 of Probable ATP-dependent RNA helicase DDX46
IPI00555136.2, Isoform 4 of Muscleblind-like protein 2
IPI00130095.1, Ras GTPase-activating protein-binding protein 1
IPI00116074.1, "Aconitate hydratase, mitochondrial"
IPI00118821.2, Platelet-activating factor acetylhydrolase IB subunit beta
IPI00990293.1, band 4.1-like protein 2
IPI00119581.2, rRNA 2'-O-methyltransferase fibrillarin
IPI00229834.4, Coatomer subunit alpha
IPI00551454.3, Protein RRP5 homolog
IPI00315488.1, "Arginyl-tRNA synthetase, cytoplasmic"
IPI00309398.6, DNA replication licensing factor MCM5
IPI00123202.1, Ataxin-10
IPI00313558.5, Coatomer subunit delta
IPI00124959.2, Uncharacterized protein
IPI00473314.3, DNA ligase 1
IPI00319231.4, 40S ribosomal protein S15
IPI00137145.1, "poly(A) binding protein, cytoplasmic 3"
IPI00469268.5, T-complex protein 1 subunit theta
IPI00115580.4, Eukaryotic translation initiation factor 3 subunit M
IPI00126940.1, Isoform Long of Adenosine kinase
IPI00263313.1, Developmentally-regulated GTP-binding protein 1
IPI00330591.1, Isoform 1 of DNA-binding protein A
IPI00274739.1, Isoform 2 of DNA-binding protein A
IPI00130353.5, Valyl-tRNA synthetase
IPI00331334.4, BAG family molecular chaperone regulator 3
IPI00132250.1, Eukaryotic translation initiation factor 3 subunit E
IPI00622371.3, Eukaryotic translation initiation factor 3 subunit G
IPI00377441.3, 40S ribosomal protein S26
IPI00284444.5, Cell division cycle 5-related protein
IPI00128945.1, Proteasome subunit beta type-2
IPI00321647.2, Eukaryotic translation initiation factor 3 subunit C
IPI00128202.1, Eukaryotic translation initiation factor 3 subunit H
IPI00756424.3, Eukaryotic translation initiation factor 5B
IPI00124938.1, Isoform 1 of Ubiquitin carboxyl-terminal hydrolase isozyme L5
IPI00230763.1, Isoform 2 of Ubiquitin carboxyl-terminal hydrolase isozyme L5
IPI00109932.1, Probable ATP-dependent RNA helicase DDX6
IPI00121596.4, Pre-mRNA-processing-splicing factor 8
IPI00337844.5, E3 SUMO-protein ligase RanBP2
IPI00474446.4, Eukaryotic translation initiation factor 2 subunit 1
IPI00759940.3, "Isoform Cytoplasmic of Fumarate hydratase, mitochondrial"
IPI00133977.3, Putative uncharacterized protein
IPI00116753.4, "Electron transfer flavoprotein subunit alpha, mitochondrial"
IPI00420363.3, Probable ATP-dependent RNA helicase DDX5
IPI00474558.7, Uncharacterized protein
IPI00127172.3, ATP-dependent RNA helicase DDX1
IPI00420601.6, Isoform 2 of Ubiquitin carboxyl-terminal hydrolase 10
IPI00222307.1, Isoform 1 of Nuclear pore complex protein Nup93
IPI00409462.2, Spliceosome RNA helicase Ddx39b
IPI00170394.1, Splicing factor 45
IPI00118762.6, Bystin
IPI00120503.1, Coatomer subunit beta
IPI00130840.7, Coatomer subunit epsilon
IPI00115902.2, 60S ribosomal protein L35a
IPI00116825.1, ATP-binding cassette sub-family F member 2
IPI00894870.1, Isoform 1 of tRNA (cytosine(34)-C(5))-methyltransferase
IPI00123313.1, Ubiquitin-like modifier-activating enzyme 1
IPI00337930.4, Isoform 1 of Cytoskeleton-associated protein 5
IPI00122350.4, U1 small nuclear ribonucleoprotein A
IPI00461621.1, U5 small nuclear ribonucleoprotein 40 kDa protein
IPI00123604.4, 40S ribosomal protein SA
IPI00659629.1, 40S ribosomal protein SA-like isoform 1
IPI00113134.1, Superkiller viralicidic activity 2-like 2
IPI00123494.3, 26S proteasome non-ATPase regulatory subunit 2
IPI00111556.1, Serine/threonine-protein phosphatase 2A catalytic subunit beta isoform
IPI00120374.1, Serine/threonine-protein phosphatase 2A catalytic subunit alpha isoform
IPI00283862.6, Proteasome subunit alpha type-1
IPI00874566.3, Uncharacterized protein
IPI00380309.3, Isoform 1 of Putative RNA-binding protein Luc7-like 2
IPI00114333.2, Ribosomal protein S6 kinase alpha-3
IPI00776055.1, Uncharacterized protein
IPI00331556.5, Heat shock 70 kDa protein 4
IPI00459493.5, Isoform 1 of T-complex protein 1 subunit alpha
IPI00116283.1, T-complex protein 1 subunit gamma
IPI00330624.2, nucleoporin 153
IPI00857897.1, Isoform 2 of Paraspeckle component 1
IPI00785240.2, Isoform 1 of Serine/arginine repetitive matrix protein 2
IPI00133903.1, "Stress-70 protein, mitochondrial"
IPI00776162.3, eukaryotic translation initiation factor 4 gamma 2 isoform 1
IPI00480480.2, "RNA-binding motif, single-stranded-interacting protein 1 isoform 1"
IPI00318048.5, Nucleolar protein 56
IPI00119632.3, Putative rRNA methyltransferase 3
IPI00265406.3, Phosphoribosylformylglycinamidine synthase
IPI00459381.2, ATP-dependent RNA helicase DDX18
IPI00109109.1, "Superoxide dismutase [Mn], mitochondrial"
IPI00132576.1, U2 small nuclear ribonucleoprotein B''
IPI00169448.1, NEDD8-conjugating enzyme Ubc12
IPI00129164.1, sepiapterin reductase
IPI00554862.3, "6-phosphofructokinase, liver type"
IPI00462424.1, Isoform 1 of RNA-binding protein with serine-rich domain 1
IPI00109824.1, "Bifunctional methylenetetrahydrofolate dehydrogenase/cyclohydrolase, mitochondrial"
IPI00323592.2, "Malate dehydrogenase, mitochondrial"
IPI00228590.2, Microfibrillar-associated protein 1
IPI00228253.2, "Acetyl-CoA acetyltransferase, cytosolic"
IPI00776104.1, Uncharacterized protein
IPI00132728.2, "Isoform 1 of Cytochrome c1, heme protein, mitochondrial"
IPI00126396.2, Putative uncharacterized protein
IPI00323820.4, Putative uncharacterized protein
IPI00230415.5, "Eukaryotic translation initiation factor 2 subunit 3, X-linked"
IPI00856902.1, Uncharacterized protein
IPI00122202.5, Uncharacterized protein
IPI00464141.2, Uncharacterized protein
IPI00109419.2, Chromosome-associated kinesin KIF4
IPI00990575.1, 60S ribosomal protein L30
IPI00830734.1, Uncharacterized protein
IPI00170316.1, Multiple coagulation factor deficiency protein 2 homolog
IPI00114667.1, 26S proteasome non-ATPase regulatory subunit 7
IPI00132456.1, UPF0568 protein C14orf166 homolog
IPI00380281.4, splicing factor 3A subunit 2
IPI00474157.4, Mitochondrial import receptor subunit TOM40 homolog
IPI00114052.1, Small nuclear ribonucleoprotein-associated protein B
IPI00118676.3, Eukaryotic initiation factor 4A-I
IPI00119138.1, "Cytochrome b-c1 complex subunit 2, mitochondrial"
IPI00409918.1, Isoform 2 of Eukaryotic initiation factor 4A-II
IPI00120954.2, "poly A binding protein, cytoplasmic 2"
IPI00115117.1, Stomatin-like protein 2

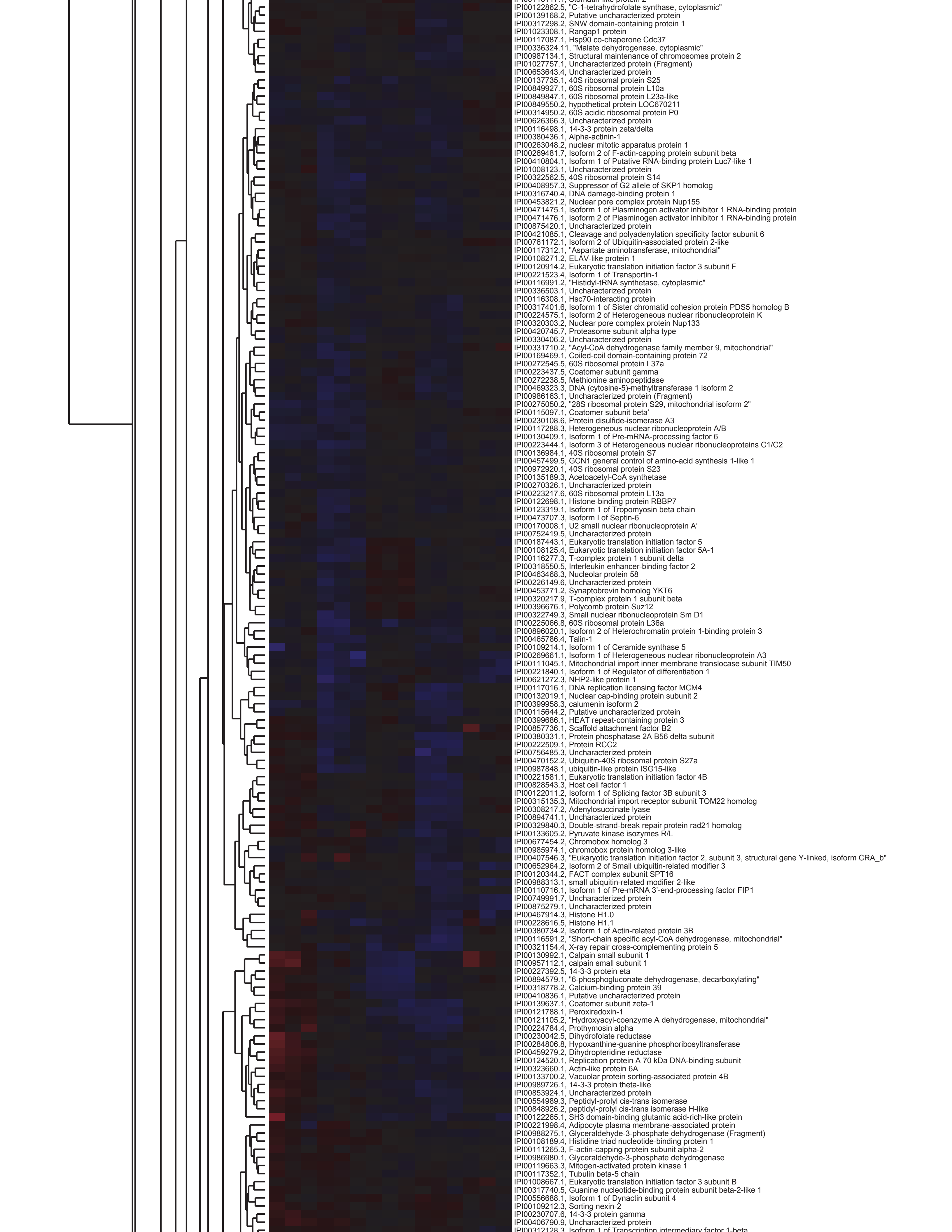
IPI00122862.5, "C-1-tetrahydrofolate synthase, cytoplasmic"
IPI00139168.2, Putative uncharacterized protein
IPI00317298.2, SNW domain-containing protein 1
IPI01023308.1, Rangap1 protein
IPI00117087.1, Hsp90 co-chaperone Cdc37
IPI00336324.11, "Malate dehydrogenase, cytoplasmic"
IPI00987134.1, Structural maintenance of chromosomes protein 2
IPI01027757.1, Uncharacterized protein (Fragment)
IPI00653643.4, Uncharacterized protein
IPI00137735.1, 40S ribosomal protein S25
IPI00849927.1, 60S ribosomal protein L10a
IPI00849847.1, 60S ribosomal protein L23a-like
IPI00849550.2, hypothetical protein LOC670211
IPI00314950.2, 60S acidic ribosomal protein P0
IPI00626366.3, Uncharacterized protein
IPI00116498.1, 14-3-3 protein zeta/delta
IPI00380436.1, Alpha-actinin-1
IPI00263048.2, nuclear mitotic apparatus protein 1
IPI00269481.7, Isoform 2 of F-actin-capping protein subunit beta
IPI00410804.1, Isoform 1 of Putative RNA-binding protein Luc7-like 1
IPI01008123.1, Uncharacterized protein
IPI00322562.5, 40S ribosomal protein S14
IPI00408957.3, Suppressor of G2 allele of SKP1 homolog
IPI00316740.4, DNA damage-binding protein 1
IPI00453821.2, Nuclear pore complex protein Nup155
IPI00471475.1, Isoform 1 of Plasminogen activator inhibitor 1 RNA-binding protein
IPI00471476.1, Isoform 2 of Plasminogen activator inhibitor 1 RNA-binding protein
IPI00875420.1, Uncharacterized protein
IPI00421085.1, Cleavage and polyadenylation specificity factor subunit 6
IPI00761172.1, Isoform 2 of Ubiquitin-associated protein 2-like
IPI00117312.1, "Aspartate aminotransferase, mitochondrial"
IPI00108271.2, ELAV-like protein 1
IPI00120914.2, Eukaryotic translation initiation factor 3 subunit F
IPI00221523.4, Isoform 1 of Transportin-1
IPI00116991.2, "Histidyl-tRNA synthetase, cytoplasmic"
IPI00336503.1, Uncharacterized protein
IPI00116308.1, Hsc70-interacting protein
IPI00317401.6, Isoform 1 of Sister chromatid cohesion protein PDS5 homolog B
IPI00224575.1, Isoform 2 of Heterogeneous nuclear ribonucleoprotein K
IPI00320303.2, Nuclear pore complex protein Nup133
IPI00420745.7, Proteasome subunit alpha type
IPI00330406.2, Uncharacterized protein
IPI00331710.2, "Acyl-CoA dehydrogenase family member 9, mitochondrial"
IPI00169469.1, Coiled-coil domain-containing protein 72
IPI00272545.5, 60S ribosomal protein L37a
IPI00223437.5, Coatomer subunit gamma
IPI00272238.5, Methionine aminopeptidase
IPI00469323.3, DNA (cytosine-5)-methyltransferase 1 isoform 2
IPI00986163.1, Uncharacterized protein (Fragment)
IPI00275050.2, "28S ribosomal protein S29, mitochondrial isoform 2"
IPI00115097.1, Coatomer subunit beta'
IPI00230108.6, Protein disulfide-isomerase A3
IPI00117288.3, Heterogeneous nuclear ribonucleoprotein A/B
IPI00130409.1, Isoform 1 of Pre-mRNA-processing factor 6
IPI00223444.1, Isoform 3 of Heterogeneous nuclear ribonucleoproteins C1/C2
IPI00136984.1, 40S ribosomal protein S7
IPI00457499.5, GCN1 general control of amino-acid synthesis 1-like 1
IPI00972920.1, 40S ribosomal protein S23
IPI00135189.3, Acetoacetyl-CoA synthetase
IPI00270326.1, Uncharacterized protein
IPI00223217.6, 60S ribosomal protein L13a
IPI00122698.1, Histone-binding protein RBBP7
IPI00123319.1, Isoform 1 of Tropomyosin beta chain
IPI00473707.3, Isoform I of Septin-6
IPI00170008.1, U2 small nuclear ribonucleoprotein A'
IPI00752419.5, Uncharacterized protein
IPI00187443.1, Eukaryotic translation initiation factor 5
IPI00108125.4, Eukaryotic translation initiation factor 5A-1
IPI00116277.3, T-complex protein 1 subunit delta
IPI00318550.5, Interleukin enhancer-binding factor 2
IPI00463468.3, Nucleolar protein 58
IPI00226149.6, Uncharacterized protein
IPI00453771.2, Synaptobrevin homolog YKT6
IPI00320217.9, T-complex protein 1 subunit beta
IPI00396676.1, Polycomb protein Suz12
IPI00322749.3, Small nuclear ribonucleoprotein Sm D1
IPI00225066.8, 60S ribosomal protein L36a
IPI00896020.1, Isoform 2 of Heterochromatin protein 1-binding protein 3
IPI00465786.4, Talin-1
IPI00109214.1, Isoform 1 of Ceramide synthase 5
IPI00269661.1, Isoform 1 of Heterogeneous nuclear ribonucleoprotein A3
IPI00111045.1, Mitochondrial import inner membrane translocase subunit TIM50
IPI00221840.1, Isoform 1 of Regulator of differentiation 1
IPI00621272.3, NHP2-like protein 1
IPI00117016.1, DNA replication licensing factor MCM4
IPI00132019.1, Nuclear cap-binding protein subunit 2
IPI00399958.3, calumenin isoform 2
IPI00115644.2, Putative uncharacterized protein
IPI00399686.1, HEAT repeat-containing protein 3
IPI00857736.1, Scaffold attachment factor B2
IPI00380331.1, Protein phosphatase 2A B56 delta subunit
IPI00222509.1, Protein RCC2
IPI00756485.3, Uncharacterized protein
IPI00470152.2, Ubiquitin-40S ribosomal protein S27a
IPI00987848.1, ubiquitin-like protein ISG15-like
IPI00221581.1, Eukaryotic translation initiation factor 4B
IPI00828543.3, Host cell factor 1
IPI00122011.2, Isoform 1 of Splicing factor 3B subunit 3
IPI00315135.3, Mitochondrial import receptor subunit TOM22 homolog
IPI00308217.2, Adenylosuccinate lyase
IPI00894741.1, Uncharacterized protein
IPI00329840.3, Double-strand-break repair protein rad21 homolog
IPI00133605.2, Pyruvate kinase isozymes R/L
IPI00677454.2, Chromobox homolog 3
IPI00985974.1, chromobox protein homolog 3-like
IPI00407546.3, "Eukaryotic translation initiation factor 2, subunit 3, structural gene Y-linked, isoform CRA_b"
IPI00652964.2, Isoform 2 of Small ubiquitin-related modifier 3
IPI00120344.2, FACT complex subunit SPT16
IPI00988313.1, small ubiquitin-related modifier 2-like
IPI00110716.1, Isoform 1 of Pre-mRNA 3'-end-processing factor FIP1
IPI00749991.7, Uncharacterized protein
IPI00875279.1, Uncharacterized protein
IPI00467914.3, Histone H1.0
IPI00228616.5, Histone H1.1
IPI00380734.2, Isoform 1 of Actin-related protein 3B
IPI00116591.2, "Short-chain specific acyl-CoA dehydrogenase, mitochondrial"
IPI00321154.4, X-ray repair cross-complementing protein 5
IPI00130992.1, Calpain small subunit 1
IPI00957112.1, calpain small subunit 1
IPI00227392.5, 14-3-3 protein eta
IPI00894579.1, "6-phosphogluconate dehydrogenase, decarboxylating"
IPI00318778.2, Calcium-binding protein 39
IPI00410836.1, Putative uncharacterized protein
IPI00139637.1, Coatomer subunit zeta-1
IPI00121788.1, Peroxiredoxin-1
IPI00121105.2, "Hydroxyacyl-coenzyme A dehydrogenase, mitochondrial"
IPI00224784.4, Prothymosin alpha
IPI00230042.5, Dihydrofolate reductase
IPI00284806.8, Hypoxanthine-guanine phosphoribosyltransferase
IPI00459279.2, Dihydropteridine reductase
IPI00124520.1, Replication protein A 70 kDa DNA-binding subunit
IPI00323660.1, Actin-like protein 6A
IPI00133700.2, Vacuolar protein sorting-associated protein 4B
IPI00989726.1, 14-3-3 protein theta-like
IPI00853924.1, Uncharacterized protein
IPI00554989.3, Peptidyl-prolyl cis-trans isomerase
IPI00848926.2, peptidyl-prolyl cis-trans isomerase H-like
IPI00122265.1, SH3 domain-binding glutamic acid-rich-like protein
IPI00221998.4, Adipocyte plasma membrane-associated protein
IPI00988275.1, Glyceraldehyde-3-phosphate dehydrogenase (Fragment)
IPI00108189.4, Histidine triad nucleotide-binding protein 1
IPI00111265.3, F-actin-capping protein subunit alpha-2
IPI00986980.1, Glyceraldehyde-3-phosphate dehydrogenase
IPI00119663.3, Mitogen-activated protein kinase 1
IPI00117352.1, Tubulin beta-5 chain
IPI01008667.1, Eukaryotic translation initiation factor 3 subunit B
IPI00317740.5, Guanine nucleotide-binding protein subunit beta-2-like 1
IPI00556688.1, Isoform 1 of Dynactin subunit 4
IPI00109212.3, Sorting nexin-2
IPI00230707.6, 14-3-3 protein gamma
IPI00406790.9, Uncharacterized protein

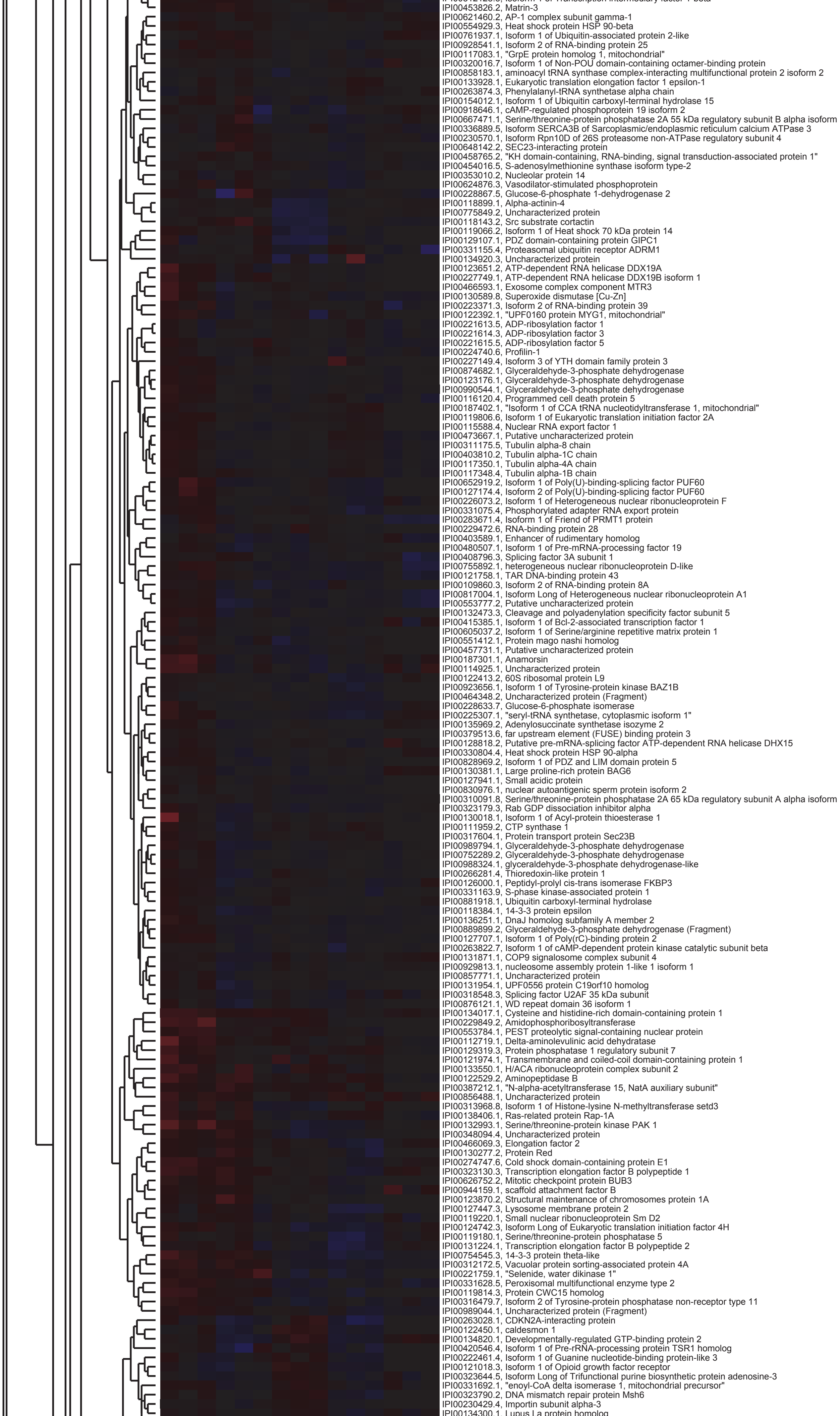
IPI00453826.2, Matrin-3
IPI00621460.2, AP-1 complex subunit gamma-1
IPI00554929.3, Heat shock protein HSP 90-beta
IPI00761937.1, Isoform 1 of Ubiquitin-associated protein 2-like
IPI00928541.1, Isoform 2 of RNA-binding protein 25
IPI00117083.1, "GrpE protein homolog 1, mitochondrial"
IPI00320016.7, Isoform 1 of Non-POU domain-containing octamer-binding protein
IPI00858183.1, aminoacyl tRNA synthase complex-interacting multifunctional protein 2 isoform 2
IPI00133928.1, Eukaryotic translation elongation factor 1 epsilon-1
IPI00263874.3, Phenylalanyl-tRNA synthetase alpha chain
IPI00154012.1, Isoform 1 of Ubiquitin carboxyl-terminal hydrolase 15
IPI00918646.1, cAMP-regulated phosphoprotein 19 isoform 2
IPI00667471.1, Serine/threonine-protein phosphatase 2A 55 kDa regulatory subunit B alpha isoform
IPI00336889.5, Isoform SERCA3B of Sarcoplasmic/endoplasmic reticulum calcium ATPase 3
IPI00230570.1, Isoform Rpn10D of 26S proteasome non-ATPase regulatory subunit 4
IPI00648142.2, SEC23-interacting protein
IPI00458765.2, "KH domain-containing, RNA-binding, signal transduction-associated protein 1"
IPI00454016.5, S-adenosylmethionine synthase isoform type-2
IPI00353010.2, Nucleolar protein 14
IPI00624876.3, Vasodilator-stimulated phosphoprotein
IPI00228867.5, Glucose-6-phosphate 1-dehydrogenase 2
IPI00118899.1, Alpha-actinin-4
IPI00775849.2, Uncharacterized protein
IPI00118143.2, Src substrate cortactin
IPI00119066.2, Isoform 1 of Heat shock 70 kDa protein 14
IPI00129107.1, PDZ domain-containing protein GIPC1
IPI00331155.4, Proteasomal ubiquitin receptor ADRM1
IPI00134920.3, Uncharacterized protein
IPI00123651.2, ATP-dependent RNA helicase DDX19A
IPI00227749.1, ATP-dependent RNA helicase DDX19B isoform 1
IPI00466593.1, Exosome complex component MTR3
IPI00130589.8, Superoxide dismutase [Cu-Zn]
IPI00223371.3, Isoform 2 of RNA-binding protein 39
IPI00122392.1, "UPF0160 protein MYG1, mitochondrial"
IPI00221613.5, ADP-ribosylation factor 1
IPI00221614.3, ADP-ribosylation factor 3
IPI00221615.5, ADP-ribosylation factor 5
IPI00224740.6, Profilin-1
IPI00227149.4, Isoform 3 of YTH domain family protein 3
IPI00874682.1, Glyceraldehyde-3-phosphate dehydrogenase
IPI00123176.1, Glyceraldehyde-3-phosphate dehydrogenase
IPI00990544.1, Glyceraldehyde-3-phosphate dehydrogenase
IPI00116120.4, Programmed cell death protein 5
IPI00187402.1, "Isoform 1 of CCA tRNA nucleotidyltransferase 1, mitochondrial"
IPI00119806.6, Isoform 1 of Eukaryotic translation initiation factor 2A
IPI00115588.4, Nuclear RNA export factor 1
IPI00473667.1, Putative uncharacterized protein
IPI00311175.5, Tubulin alpha-8 chain
IPI00403810.2, Tubulin alpha-1C chain
IPI00117350.1, Tubulin alpha-4A chain
IPI00117348.4, Tubulin alpha-1B chain
IPI00652919.2, Isoform 1 of Poly(U)-binding-splicing factor PUF60
IPI00127174.4, Isoform 2 of Poly(U)-binding-splicing factor PUF60
IPI00226073.2, Isoform 1 of Heterogeneous nuclear ribonucleoprotein F
IPI00331075.4, Phosphorylated adapter RNA export protein
IPI00283671.4, Isoform 1 of Friend of PRMT1 protein
IPI00229472.6, RNA-binding protein 28
IPI00403589.1, Enhancer of rudimentary homolog
IPI00480507.1, Isoform 1 of Pre-mRNA-processing factor 19
IPI00408796.3, Splicing factor 3A subunit 1
IPI00755892.1, heterogeneous nuclear ribonucleoprotein D-like
IPI00121758.1, TAR DNA-binding protein 43
IPI00109860.3, Isoform 2 of RNA-binding protein 8A
IPI00817004.1, Isoform Long of Heterogeneous nuclear ribonucleoprotein A1
IPI00553777.2, Putative uncharacterized protein
IPI00132473.3, Cleavage and polyadenylation specificity factor subunit 5
IPI00415385.1, Isoform 1 of Bcl-2-associated transcription factor 1
IPI00605037.2, Isoform 1 of Serine/arginine repetitive matrix protein 1
IPI00551412.1, Protein mago nashi homolog
IPI00457731.1, Putative uncharacterized protein
IPI00187301.1, Anamorsin
IPI00114925.1, Uncharacterized protein
IPI00122413.2, 60S ribosomal protein L9
IPI00923656.1, Isoform 1 of Tyrosine-protein kinase BAZ1B
IPI00464348.2, Uncharacterized protein (Fragment)
IPI00228633.7, Glucose-6-phosphate isomerase
IPI00225307.1, "seryl-tRNA synthetase, cytoplasmic isoform 1"
IPI00135969.2, Adenylosuccinate synthetase isozyme 2
IPI00379513.6, far upstream element (FUSE) binding protein 3
IPI00128818.2, Putative pre-mRNA-splicing factor ATP-dependent RNA helicase DHX15
IPI00330804.4, Heat shock protein HSP 90-alpha
IPI00828969.2, Isoform 1 of PDZ and LIM domain protein 5
IPI00130381.1, Large proline-rich protein BAG6
IPI00127941.1, Small acidic protein
IPI00830976.1, nuclear autoantigenic sperm protein isoform 2
IPI00310091.8, Serine/threonine-protein phosphatase 2A 65 kDa regulatory subunit A alpha isoform
IPI00323179.3, Rab GDP dissociation inhibitor alpha
IPI00130018.1, Isoform 1 of Acyl-protein thioesterase 1
IPI00111959.2, CTP synthase 1
IPI00317604.1, Protein transport protein Sec23B
IPI00989794.1, Glyceraldehyde-3-phosphate dehydrogenase
IPI00752289.2, Glyceraldehyde-3-phosphate dehydrogenase
IPI00988324.1, glyceraldehyde-3-phosphate dehydrogenase-like
IPI00266281.4, Thioredoxin-like protein 1
IPI00126000.1, Peptidyl-prolyl cis-trans isomerase FKBP3
IPI00331163.9, S-phase kinase-associated protein 1
IPI00881918.1, Ubiquitin carboxyl-terminal hydrolase
IPI00118384.1, 14-3-3 protein epsilon
IPI00136251.1, DnaJ homolog subfamily A member 2
IPI00889899.2, Glyceraldehyde-3-phosphate dehydrogenase (Fragment)
IPI00127707.1, Isoform 1 of Poly(rC)-binding protein 2
IPI00263822.7, Isoform 1 of cAMP-dependent protein kinase catalytic subunit beta
IPI00131871.1, COP9 signalosome complex subunit 4
IPI00929813.1, nucleosome assembly protein 1-like 1 isoform 1
IPI00857771.1, Uncharacterized protein
IPI00131954.1, UPF0556 protein C19orf10 homolog
IPI00318548.3, Splicing factor U2AF 35 kDa subunit
IPI00876121.1, WD repeat domain 36 isoform 1
IPI00134017.1, Cysteine and histidine-rich domain-containing protein 1
IPI00229849.2, Amidophosphoribosyltransferase
IPI00553784.1, PEST proteolytic signal-containing nuclear protein
IPI00112719.1, Delta-aminolevulinic acid dehydratase
IPI00129319.3, Protein phosphatase 1 regulatory subunit 7
IPI00121974.1, Transmembrane and coiled-coil domain-containing protein 1
IPI00133550.1, H/ACA ribonucleoprotein complex subunit 2
IPI00122529.2, Aminopeptidase B
IPI00387212.1, "N-alpha-acetyltransferase 15, NatA auxiliary subunit"
IPI00856488.1, Uncharacterized protein
IPI00313968.8, Isoform 1 of Histone-lysine N-methyltransferase setd3
IPI00138406.1, Ras-related protein Rap-1A
IPI00132993.1, Serine/threonine-protein kinase PAK 1
IPI00348094.4, Uncharacterized protein
IPI00466069.3, Elongation factor 2
IPI00130277.2, Protein Red
IPI00274747.6, Cold shock domain-containing protein E1
IPI00323130.3, Transcription elongation factor B polypeptide 1
IPI00626752.2, Mitotic checkpoint protein BUB3
IPI00944159.1, scaffold attachment factor B
IPI00123870.2, Structural maintenance of chromosomes protein 1A
IPI00127447.3, Lysosome membrane protein 2
IPI00119220.1, Small nuclear ribonucleoprotein Sm D2
IPI00124742.3, Isoform Long of Eukaryotic translation initiation factor 4H
IPI00119180.1, Serine/threonine-protein phosphatase 5
IPI00131224.1, Transcription elongation factor B polypeptide 2
IPI00754545.3, 14-3-3 protein theta-like
IPI00312172.5, Vacuolar protein sorting-associated protein 4A
IPI00221759.1, "Selenide, water dikinase 1"
IPI00331628.5, Peroxisomal multifunctional enzyme type 2
IPI00119814.3, Protein CWC15 homolog
IPI00316479.7, Isoform 2 of Tyrosine-protein phosphatase non-receptor type 11
IPI00989044.1, Uncharacterized protein (Fragment)
IPI00263028.1, CDKN2A-interacting protein
IPI00122450.1, caldesmon 1
IPI00134820.1, Developmentally-regulated GTP-binding protein 2
IPI00420546.4, Isoform 1 of Pre-rRNA-processing protein TSR1 homolog
IPI00222461.4, Isoform 1 of Guanine nucleotide-binding protein-like 3
IPI00121018.3, Isoform 1 of Opioid growth factor receptor
IPI00323644.5, Isoform Long of Trifunctional purine biosynthetic protein adenosine-3
IPI00331692.1, "enoyl-CoA delta isomerase 1, mitochondrial precursor"
IPI00323790.2, DNA mismatch repair protein Msh6
IPI00230429.4, Importin subunit alpha-3
IPI00134300.1, Lupus La protein homolog

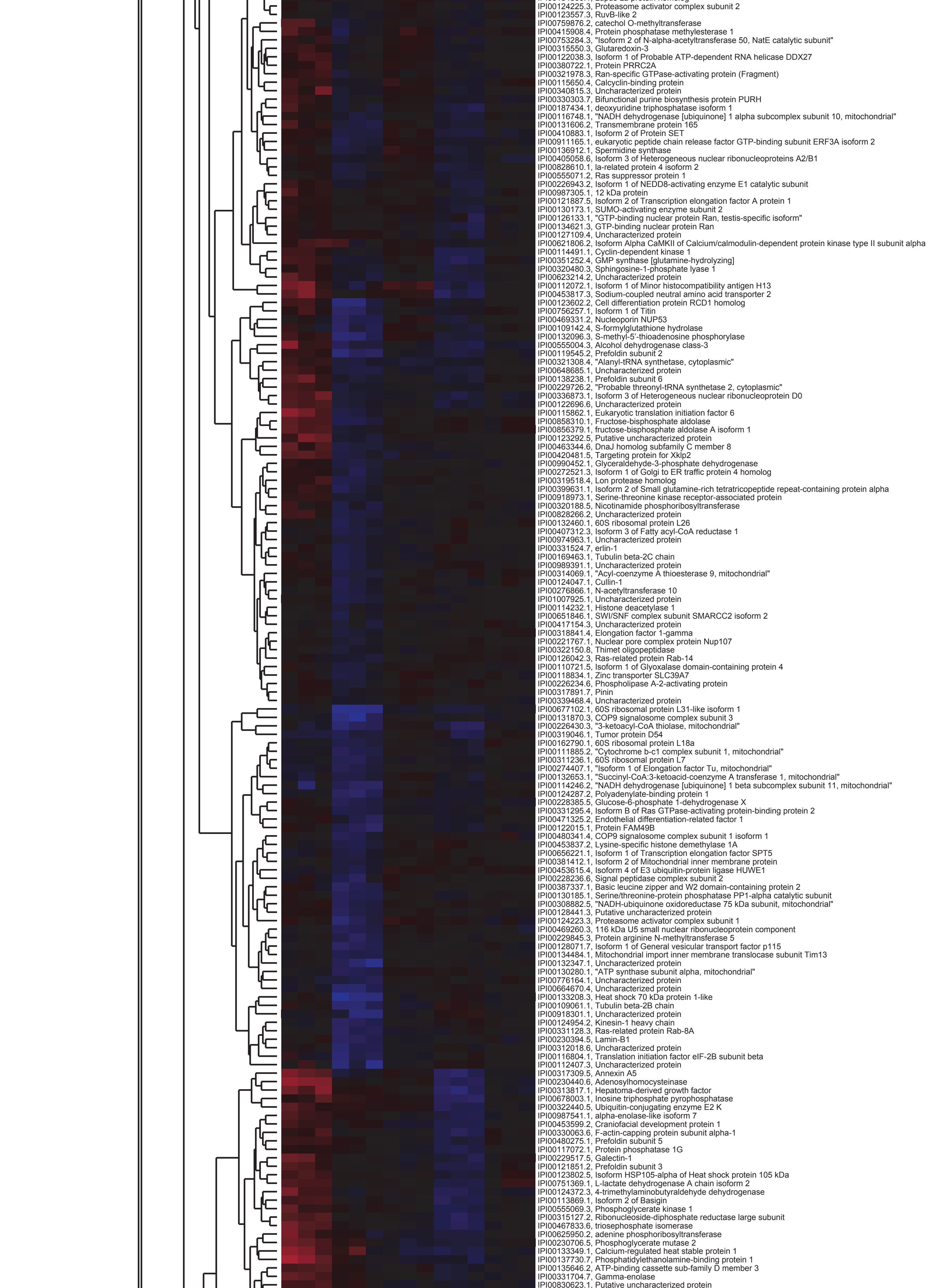
IPI00124225.3, Proteasome activator complex subunit 2
IPI00123557.3, RuvB-like 2
IPI00759876.2, catechol O-methyltransferase
IPI00415908.4, Protein phosphatase methylesterase 1
IPI00753284.3, "Isoform 2 of N-alpha-acetyltransferase 50, NatE catalytic subunit"
IPI00315550.3, Glutaredoxin-3
IPI00122038.3, Isoform 1 of Probable ATP-dependent RNA helicase DDX27
IPI00380722.1, Protein PRRC2A
IPI00321978.3, Ran-specific GTPase-activating protein (Fragment)
IPI00115650.4, Calcyclin-binding protein
IPI00340815.3, Uncharacterized protein
IPI00330303.7, Bifunctional purine biosynthesis protein PURH
IPI00187434.1, deoxyuridine triphosphatase isoform 1
IPI00116748.1, "NADH dehydrogenase [ubiquinone] 1 alpha subcomplex subunit 10, mitochondrial"
IPI00131606.2, Transmembrane protein 165
IPI00410883.1, Isoform 2 of Protein SET
IPI00911165.1, eukaryotic peptide chain release factor GTP-binding subunit ERF3A isoform 2
IPI00136912.1, Spermidine synthase
IPI00405058.6, Isoform 3 of Heterogeneous nuclear ribonucleoproteins A2/B1
IPI00828610.1, la-related protein 4 isoform 2
IPI00555071.2, Ras suppressor protein 1
IPI00226943.2, Isoform 1 of NEDD8-activating enzyme E1 catalytic subunit
IPI00987305.1, 12 kDa protein
IPI00121887.5, Isoform 2 of Transcription elongation factor A protein 1
IPI00130173.1, SUMO-activating enzyme subunit 2
IPI00126133.1, "GTP-binding nuclear protein Ran, testis-specific isoform"
IPI00134621.3, GTP-binding nuclear protein Ran
IPI00127109.4, Uncharacterized protein
IPI00621806.2, Isoform Alpha CaMKII of Calcium/calmodulin-dependent protein kinase type II subunit alpha
IPI00114491.1, Cyclin-dependent kinase 1
IPI00351252.4, GMP synthase [glutamine-hydrolyzing]
IPI00320480.3, Sphingosine-1-phosphate lyase 1
IPI00623214.2, Uncharacterized protein
IPI00112072.1, Isoform 1 of Minor histocompatibility antigen H13
IPI00453817.3, Sodium-coupled neutral amino acid transporter 2
IPI00123602.2, Cell differentiation protein RCD1 homolog
IPI00756257.1, Isoform 1 of Titin
IPI00469331.2, Nucleoporin NUP53
IPI00109142.4, S-formylglutathione hydrolase
IPI00132096.3, S-methyl-5'-thioadenosine phosphorylase
IPI00555004.3, Alcohol dehydrogenase class-3
IPI00119545.2, Prefoldin subunit 2
IPI00321308.4, "Alanyl-tRNA synthetase, cytoplasmic"
IPI00648685.1, Uncharacterized protein
IPI00138238.1, Prefoldin subunit 6
IPI00229726.2, "Probable threonyl-tRNA synthetase 2, cytoplasmic"
IPI00336873.1, Isoform 3 of Heterogeneous nuclear ribonucleoprotein D0
IPI00122696.6, Uncharacterized protein
IPI00115862.1, Eukaryotic translation initiation factor 6
IPI00858310.1, Fructose-bisphosphate aldolase
IPI00856379.1, fructose-bisphosphate aldolase A isoform 1
IPI00123292.5, Putative uncharacterized protein
IPI00463344.6, DnaJ homolog subfamily C member 8
IPI00420481.5, Targeting protein for Xklp2
IPI00990452.1, Glyceraldehyde-3-phosphate dehydrogenase
IPI00272521.3, Isoform 1 of Golgi to ER traffic protein 4 homolog
IPI00319518.4, Lon protease homolog
IPI00399631.1, Isoform 2 of Small glutamine-rich tetratricopeptide repeat-containing protein alpha
IPI00918973.1, Serine-threonine kinase receptor-associated protein
IPI00320188.5, Nicotinamide phosphoribosyltransferase
IPI00828266.2, Uncharacterized protein
IPI00132460.1, 60S ribosomal protein L26
IPI00407312.3, Isoform 3 of Fatty acyl-CoA reductase 1
IPI00974963.1, Uncharacterized protein
IPI00331524.7, erlin-1
IPI00169463.1, Tubulin beta-2C chain
IPI00989391.1, Uncharacterized protein
IPI00314069.1, "Acyl-coenzyme A thioesterase 9, mitochondrial"
IPI00124047.1, Cullin-1
IPI00276866.1, N-acetyltransferase 10
IPI01007925.1, Uncharacterized protein
IPI00114232.1, Histone deacetylase 1
IPI00651846.1, SWI/SNF complex subunit SMARCC2 isoform 2
IPI00417154.3, Uncharacterized protein
IPI00318841.4, Elongation factor 1-gamma
IPI00221767.1, Nuclear pore complex protein Nup107
IPI00322150.8, Thimet oligopeptidase
IPI00126042.3, Ras-related protein Rab-14
IPI00110721.5, Isoform 1 of Glyoxalase domain-containing protein 4
IPI00118834.1, Zinc transporter SLC39A7
IPI00226234.6, Phospholipase A-2-activating protein
IPI00317891.7, Pinin
IPI00339468.4, Uncharacterized protein
IPI00677102.1, 60S ribosomal protein L31-like isoform 1
IPI00131870.3, COP9 signalosome complex subunit 3
IPI00226430.3, "3-ketoacyl-CoA thiolase, mitochondrial"
IPI00319046.1, Tumor protein D54
IPI00162790.1, 60S ribosomal protein L18a
IPI00111885.2, "Cytochrome b-c1 complex subunit 1, mitochondrial"
IPI00311236.1, 60S ribosomal protein L7
IPI00274407.1, "Isoform 1 of Elongation factor Tu, mitochondrial"
IPI00132653.1, "Succinyl-CoA:3-ketoacid-coenzyme A transferase 1, mitochondrial"
IPI00114246.2, "NADH dehydrogenase [ubiquinone] 1 beta subcomplex subunit 11, mitochondrial"
IPI00124287.2, Polyadenylate-binding protein 1
IPI00228385.5, Glucose-6-phosphate 1-dehydrogenase X
IPI00331295.4, Isoform B of Ras GTPase-activating protein-binding protein 2
IPI00471325.2, Endothelial differentiation-related factor 1
IPI00122015.1, Protein FAM49B
IPI00480341.4, COP9 signalosome complex subunit 1 isoform 1
IPI00453837.2, Lysine-specific histone demethylase 1A
IPI00656221.1, Isoform 1 of Transcription elongation factor SPT5
IPI00381412.1, Isoform 2 of Mitochondrial inner membrane protein
IPI00453615.4, Isoform 4 of E3 ubiquitin-protein ligase HUWE1
IPI00228236.6, Signal peptidase complex subunit 2
IPI00387337.1, Basic leucine zipper and W2 domain-containing protein 2
IPI00130185.1, Serine/threonine-protein phosphatase PP1-alpha catalytic subunit
IPI00308882.5, "NADH-ubiquinone oxidoreductase 75 kDa subunit, mitochondrial"
IPI00128441.3, Putative uncharacterized protein
IPI00124223.3, Proteasome activator complex subunit 1
IPI00469260.3, 116 kDa U5 small nuclear ribonucleoprotein component
IPI00229845.3, Protein arginine N-methyltransferase 5
IPI00128071.7, Isoform 1 of General vesicular transport factor p115
IPI00134484.1, Mitochondrial import inner membrane translocase subunit Tim13
IPI00132347.1, Uncharacterized protein
IPI00130280.1, "ATP synthase subunit alpha, mitochondrial"
IPI00776164.1, Uncharacterized protein
IPI00664670.4, Uncharacterized protein
IPI00133208.3, Heat shock 70 kDa protein 1-like
IPI00109061.1, Tubulin beta-2B chain
IPI00918301.1, Uncharacterized protein
IPI00124954.2, Kinesin-1 heavy chain
IPI00331128.3, Ras-related protein Rab-8A
IPI00230394.5, Lamin-B1
IPI00312018.6, Uncharacterized protein
IPI00116804.1, Translation initiation factor eIF-2B subunit beta
IPI00112407.3, Uncharacterized protein
IPI00317309.5, Annexin A5
IPI00230440.6, Adenosylhomocysteinase
IPI00313817.1, Hepatoma-derived growth factor
IPI00678003.1, Inosine triphosphate pyrophosphatase
IPI00322440.5, Ubiquitin-conjugating enzyme E2 K
IPI00987541.1, alpha-enolase-like isoform 7
IPI00453599.2, Craniofacial development protein 1
IPI00330063.6, F-actin-capping protein subunit alpha-1
IPI00480275.1, Prefoldin subunit 5
IPI00117072.1, Protein phosphatase 1G
IPI00229517.5, Galectin-1
IPI00121851.2, Prefoldin subunit 3
IPI00123802.5, Isoform HSP105-alpha of Heat shock protein 105 kDa
IPI00751369.1, L-lactate dehydrogenase A chain isoform 2
IPI00124372.3, 4-trimethylaminobutyraldehyde dehydrogenase
IPI00113869.1, Isoform 2 of Basigin
IPI00555069.3, Phosphoglycerate kinase 1
IPI00315127.2, Ribonucleoside-diphosphate reductase large subunit
IPI00467833.6, triosephosphate isomerase
IPI00625950.2, adenine phosphoribosyltransferase
IPI00230706.5, Phosphoglycerate mutase 2
IPI00133349.1, Calcium-regulated heat stable protein 1
IPI00137730.7, Phosphatidylethanolamine-binding protein 1
IPI00135646.2, ATP-binding cassette sub-family D member 3
IPI00331704.7, Gamma-enolase
IPI00830623.1, Putative uncharacterized protein

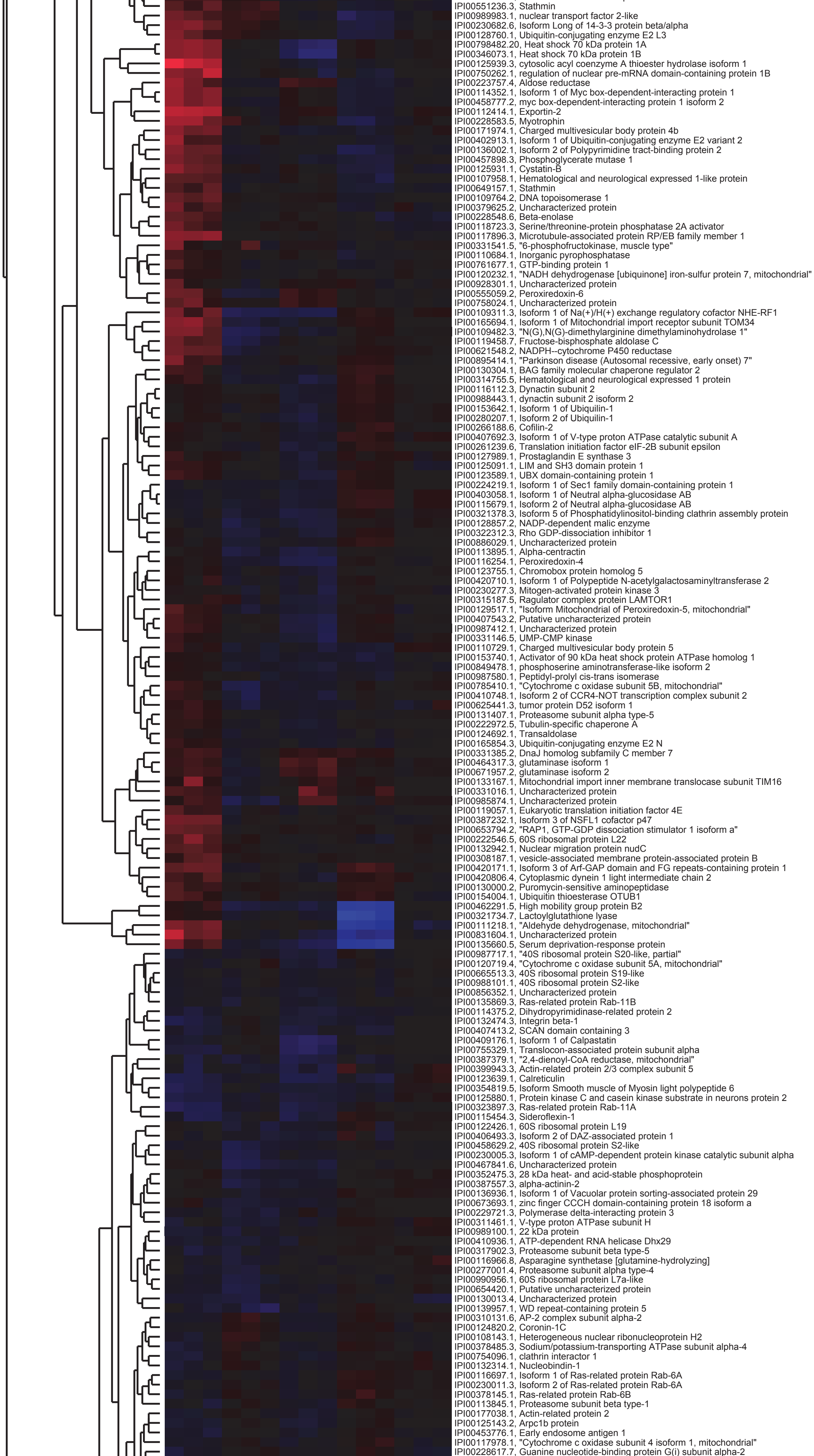

IPI00551236.3, Stathmin
IPI00989983.1, nuclear transport factor 2-like
IPI00230682.6, Isoform Long of 14-3-3 protein beta/alpha
IPI00128760.1, Ubiquitin-conjugating enzyme E2 L3
IPI00798482.20, Heat shock 70 kDa protein 1A
IPI00346073.1, Heat shock 70 kDa protein 1B
IPI00125939.3, cytosolic acyl coenzyme A thioester hydrolase isoform 1
IPI00750262.1, regulation of nuclear pre-mRNA domain-containing protein 1B
IPI00223757.4, Aldose reductase
IPI00114352.1, Isoform 1 of Myc box-dependent-interacting protein 1
IPI00458777.2, myc box-dependent-interacting protein 1 isoform 2
IPI00112414.1, Exportin-2
IPI00228583.5, Myotrophin
IPI00171974.1, Charged multivesicular body protein 4b
IPI00402913.1, Isoform 1 of Ubiquitin-conjugating enzyme E2 variant 2
IPI00136002.1, Isoform 2 of Polypyrimidine tract-binding protein 2
IPI00457898.3, Phosphoglycerate mutase 1
IPI00125931.1, Cystatin-B
IPI00107958.1, Hematological and neurological expressed 1-like protein
IPI00649157.1, Stathmin
IPI00109764.2, DNA topoisomerase 1
IPI00379625.2, Uncharacterized protein
IPI00228548.6, Beta-enolase
IPI00118723.3, Serine/threonine-protein phosphatase 2A activator
IPI00117896.3, Microtubule-associated protein RP/EB family member 1
IPI00331541.5, "6-phosphofructokinase, muscle type"
IPI00110684.1, Inorganic pyrophosphatase
IPI00761677.1, GTP-binding protein 1
IPI00120232.1, "NADH dehydrogenase [ubiquinone] iron-sulfur protein 7, mitochondrial"
IPI00928301.1, Uncharacterized protein
IPI00555059.2, Peroxiredoxin-6
IPI00758024.1, Uncharacterized protein
IPI00109311.3, Isoform 1 of Na(+)/H(+) exchange regulatory cofactor NHE-RF1
IPI00165694.1, Isoform 1 of Mitochondrial import receptor subunit TOM34
IPI00109482.3, "N(G),N(G)-dimethylarginine dimethylaminohydrolase 1"
IPI00119458.7, Fructose-bisphosphate aldolase C
IPI00621548.2, NADPH--cytochrome P450 reductase
IPI00895414.1, "Parkinson disease (Autosomal recessive, early onset) 7"
IPI00130304.1, BAG family molecular chaperone regulator 2
IPI00314755.5, Hematological and neurological expressed 1 protein
IPI00116112.3, Dynactin subunit 2
IPI00988443.1, dynactin subunit 2 isoform 2
IPI00153642.1, Isoform 1 of Ubiquilin-1
IPI00280207.1, Isoform 2 of Ubiquilin-1
IPI00266188.6, Cofilin-2
IPI00407692.3, Isoform 1 of V-type proton ATPase catalytic subunit A
IPI00261239.6, Translation initiation factor eIF-2B subunit epsilon
IPI00127989.1, Prostaglandin E synthase 3
IPI00125091.1, LIM and SH3 domain protein 1
IPI00123589.1, UBX domain-containing protein 1
IPI00224219.1, Isoform 1 of Sec1 family domain-containing protein 1
IPI00403058.1, Isoform 1 of Neutral alpha-glucosidase AB
IPI00115679.1, Isoform 2 of Neutral alpha-glucosidase AB
IPI00321378.3, Isoform 5 of Phosphatidylinositol-binding clathrin assembly protein
IPI00128857.2, NADP-dependent malic enzyme
IPI00322312.3, Rho GDP-dissociation inhibitor 1
IPI00886029.1, Uncharacterized protein
IPI00113895.1, Alpha-centractin
IPI00116254.1, Peroxiredoxin-4
IPI00123755.1, Chromobox protein homolog 5
IPI00420710.1, Isoform 1 of Polypeptide N-acetylgalactosaminyltransferase 2
IPI00230277.3, Mitogen-activated protein kinase 3
IPI00315187.5, Ragulator complex protein LAMTOR1
IPI00129517.1, "Isoform Mitochondrial of Peroxiredoxin-5, mitochondrial"
IPI00407543.2, Putative uncharacterized protein
IPI00987412.1, Uncharacterized protein
IPI00331146.5, UMP-CMP kinase
IPI00110729.1, Charged multivesicular body protein 5
IPI00153740.1, Activator of 90 kDa heat shock protein ATPase homolog 1
IPI00849478.1, phosphoserine aminotransferase-like isoform 2
IPI00987580.1, Peptidyl-prolyl cis-trans isomerase
IPI00785410.1, "Cytochrome c oxidase subunit 5B, mitochondrial"
IPI00410748.1, Isoform 2 of CCR4-NOT transcription complex subunit 2
IPI00625441.3, tumor protein D52 isoform 1
IPI00131407.1, Proteasome subunit alpha type-5
IPI00222972.5, Tubulin-specific chaperone A
IPI00124692.1, Transaldolase
IPI00165854.3, Ubiquitin-conjugating enzyme E2 N
IPI00331385.2, DnaJ homolog subfamily C member 7
IPI00464317.3, glutaminase isoform 1
IPI00671957.2, glutaminase isoform 2
IPI00133167.1, Mitochondrial import inner membrane translocase subunit TIM16
IPI00331016.1, Uncharacterized protein
IPI00985874.1, Uncharacterized protein
IPI00119057.1, Eukaryotic translation initiation factor 4E
IPI00387232.1, Isoform 3 of NSFL1 cofactor p47
IPI00653794.2, "RAP1, GTP-GDP dissociation stimulator 1 isoform a"
IPI00222546.5, 60S ribosomal protein L22
IPI00132942.1, Nuclear migration protein nudC
IPI00308187.1, vesicle-associated membrane protein-associated protein B
IPI00420171.1, Isoform 3 of Arf-GAP domain and FG repeats-containing protein 1
IPI00420806.4, Cytoplasmic dynein 1 light intermediate chain 2
IPI00130000.2, Puromycin-sensitive aminopeptidase
IPI00154004.1, Ubiquitin thioesterase OTUB1
IPI00462291.5, High mobility group protein B2
IPI00321734.7, Lactoylglutathione lyase
IPI00111218.1, "Aldehyde dehydrogenase, mitochondrial"
IPI00831604.1, Uncharacterized protein
IPI00135660.5, Serum deprivation-response protein
IPI00987717.1, "40S ribosomal protein S20-like, partial"
IPI00120719.4, "Cytochrome c oxidase subunit 5A, mitochondrial"
IPI00665513.3, 40S ribosomal protein S19-like
IPI00988101.1, 40S ribosomal protein S2-like
IPI00856352.1, Uncharacterized protein
IPI00135869.3, Ras-related protein Rab-11B
IPI00114375.2, Dihydropyrimidinase-related protein 2
IPI00132474.3, Integrin beta-1
IPI00407413.2, SCAN domain containing 3
IPI00409176.1, Isoform 1 of Calpastatin
IPI00755329.1, Translocon-associated protein subunit alpha
IPI00387379.1, "2,4-dienoyl-CoA reductase, mitochondrial"
IPI00399943.3, Actin-related protein 2/3 complex subunit 5
IPI00123639.1, Calreticulin
IPI00354819.5, Isoform Smooth muscle of Myosin light polypeptide 6
IPI00125880.1, Protein kinase C and casein kinase substrate in neurons protein 2
IPI00323897.3, Ras-related protein Rab-11A
IPI00115454.3, Sideroflexin-1
IPI00122426.1, 60S ribosomal protein L19
IPI00406493.3, Isoform 2 of DAZ-associated protein 1
IPI00458629.2, 40S ribosomal protein S2-like
IPI00230005.3, Isoform 1 of cAMP-dependent protein kinase catalytic subunit alpha
IPI00467841.6, Uncharacterized protein
IPI00352475.3, 28 kDa heat- and acid-stable phosphoprotein
IPI00387557.3, alpha-actinin-2
IPI00136936.1, Isoform 1 of Vacuolar protein sorting-associated protein 29
IPI00673693.1, zinc finger CCCH domain-containing protein 18 isoform a
IPI00229721.3, Polymerase delta-interacting protein 3
IPI00311461.1, V-type proton ATPase subunit H
IPI00989100.1, 22 kDa protein
IPI00410936.1, ATP-dependent RNA helicase Dhx29
IPI00317902.3, Proteasome subunit beta type-5
IPI00116966.8, Asparagine synthetase [glutamine-hydrolyzing]
IPI00277001.4, Proteasome subunit alpha type-4
IPI00990956.1, 60S ribosomal protein L7a-like
IPI00654420.1, Putative uncharacterized protein
IPI00130013.4, Uncharacterized protein
IPI00139957.1, WD repeat-containing protein 5
IPI00310131.6, AP-2 complex subunit alpha-2
IPI00124820.2, Coronin-1C
IPI00108143.1, Heterogeneous nuclear ribonucleoprotein H2
IPI00378485.3, Sodium/potassium-transporting ATPase subunit alpha-4
IPI00754096.1, clathrin interactor 1
IPI00132314.1, Nucleobindin-1
IPI00116697.1, Isoform 1 of Ras-related protein Rab-6A
IPI00230011.3, Isoform 2 of Ras-related protein Rab-6A
IPI00378145.1, Ras-related protein Rab-6B
IPI00113845.1, Proteasome subunit beta type-1
IPI00177038.1, Actin-related protein 2
IPI00125143.2, Arpc1b protein
IPI00453776.1, Early endosome antigen 1
IPI00117978.1, "Cytochrome c oxidase subunit 4 isoform 1, mitochondrial"
IPI00228617.7, Guanine nucleotide-binding protein G(i) subunit alpha-2

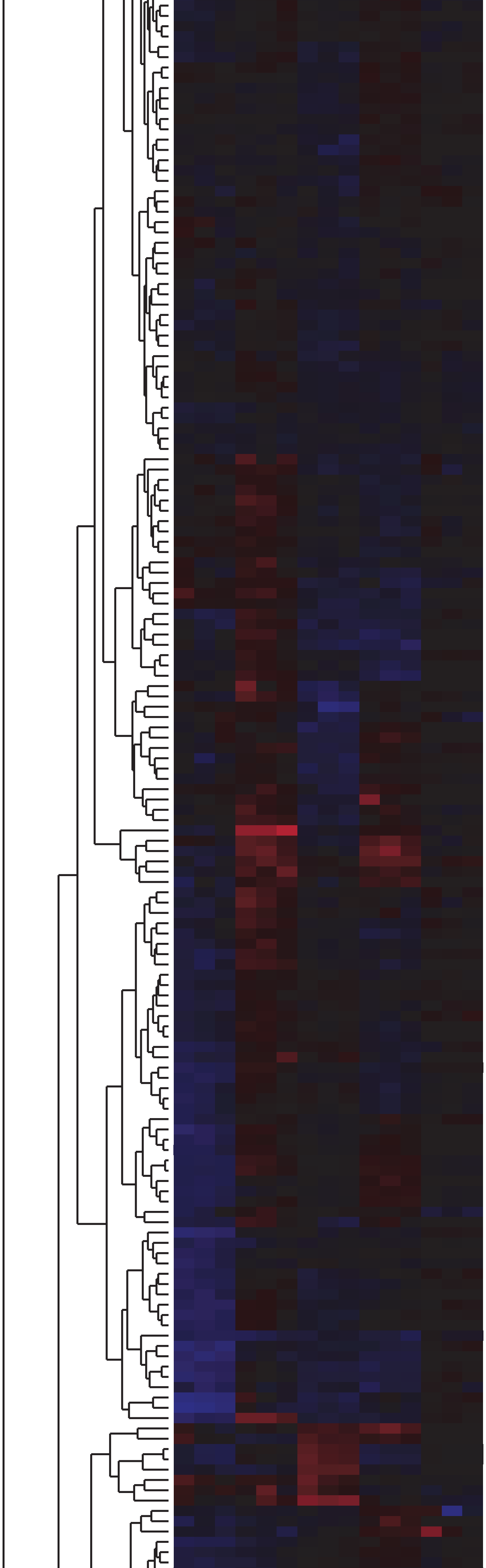
IPI01027722.1, 10 kDa protein
IPI00329839.7, Isoform 1 of ATP-dependent RNA helicase DDX42
IPI00224626.3, septin-7
IPI00123129.1, Staphylococcal nuclease domain-containing protein 1
IPI00116356.5, AP-2 complex subunit mu
IPI00316133.7, signal recognition particle 72
IPI00126083.3, EH domain-containing protein 1
IPI00653573.1, Putative uncharacterized protein (Fragment)
IPI00473415.1, 40S ribosomal protein S12-like
IPI00856500.2, dynactin subunit 1 isoform 3
IPI00989933.2, ras GTPase-activating-like protein IQGAP1
IPI00131845.1, Proteasome subunit alpha type-6
IPI00133706.1, Ras-related protein Rab-1B
IPI00136207.6, DNA-directed RNA polymerase II subunit RPB1
IPI00112597.11, Ubiquitin conjugation factor E4 B
IPI00317710.1, Isoform 1 of Heat shock 70 kDa protein 4L
IPI00224729.1, Uncharacterized protein
IPI00127408.3, Uncharacterized protein
IPI00624501.2, ATPase Asna1
IPI00135977.3, Chloride intracellular channel protein 4
IPI00648806.2, Isoform 2 of Protein FAM114A2
IPI00132352.2, Isoform 1 of UPF0368 protein Cxorf26 homolog
IPI00116877.5, transportin-2
IPI00661414.2, Actin-related protein 2/3 complex subunit 2
IPI00129512.3, Proteasome subunit beta type-4
IPI00315879.1, Cytosolic non-specific dipeptidase
IPI00230351.1, "Succinate dehydrogenase [ubiquinone] flavoprotein subunit, mitochondrial"
IPI00990885.1, Glyceraldehyde-3-phosphate dehydrogenase
IPI00119714.2, "Upstream binding transcription factor, RNA polymerase I, isoform CRA_b"
IPI00128867.3, Transcriptional activator protein Pur-beta
IPI00110588.4, Moesin
IPI00421223.3, Tropomyosin alpha-4 chain
IPI00123762.2, regulator of chromosome condensation isoform 1
IPI00955744.1, ribonuclease inhibitor isoform b
IPI00955082.1, core histone macro-H2A.1 isoform 2
IPI00471441.1, Ptms protein
IPI00230044.5, Isoform 2 of Tropomyosin alpha-3 chain
IPI00881945.1, Uncharacterized protein
IPI00459570.1, Uncharacterized protein
IPI00663587.1, Heterogeneous nuclear ribonucleoprotein G-like 1
IPI00124979.2, "RNA binding motif protein, X-linked"
IPI00626139.2, Putative uncharacterized protein
IPI00119239.2, Proteasome subunit beta type-6
IPI01023315.1, Uncharacterized protein (Fragment)
IPI00119959.1, Barrier-to-autointegration factor
IPI00555055.3, Histone H2A.V
IPI00112448.1, 40S ribosomal protein S10
IPI00457533.2, Ubiquitin-associated protein 2
IPI00874728.1, Isoform 2 of Tropomyosin beta chain
IPI00395163.1, Isoform 6 of General transcription factor II-I
IPI00458583.3, Heterogeneous nuclear ribonucleoprotein U
IPI00126191.5, Isoform B2 of Lamin-B2
IPI00124245.1, Isoform A of Ras GTPase-activating protein-binding protein 2
IPI00120886.3, Nuclease-sensitive element-binding protein 1
IPI00131176.1, Cytochrome c oxidase subunit 2
IPI00117910.3, Peroxiredoxin-2
IPI00109389.1, IST1 homolog
IPI00130344.3, Chloride intracellular channel protein 1
IPI00314748.7, WD repeat-containing protein 1
IPI00330862.5, Ezrin
IPI00132217.1, Mitochondrial fission 1 protein
IPI00228879.7, High mobility group protein B3
IPI00319270.2, Perilipin-3
IPI00938457.1, Isoform 1 of Protein transport protein Sec31A
IPI00123278.1, Pyrroline-5-carboxylate reductase 2
IPI00272381.5, Putative uncharacterized protein
IPI00153607.1, Deoxynucleotidyltransferase terminal-interacting protein 2
IPI00989752.1, Putative uncharacterized protein (Fragment)
IPI00470003.3, Alpha-parvin
IPI00132539.3, Transcription factor BTF3 homolog 4
IPI00123012.1, Isoform 1 of Zinc finger protein 330
IPI00755161.2, oxysterol-binding protein 1
IPI00404438.1, Isoform 6 of Phosphatidylinositol-binding clathrin assembly protein
IPI00135231.2, Isocitrate dehydrogenase
IPI00120193.1, Isoform 1 of Cell division control protein 42 homolog
IPI01027807.1, Uncharacterized protein
IPI00117178.1, AH receptor-interacting protein
IPI00132340.1, Serine/arginine-rich splicing factor 9
IPI00230680.7, Uncharacterized protein
IPI00986276.1, Uncharacterized protein
IPI00753928.1, kinesin light chain 2
IPI00116134.2, Dipeptidyl peptidase 3
IPI00124282.1, Ras-related protein Ral-A
IPI00117252.1, Glycylpeptide N-tetradecanoyltransferase 2
IPI00656325.2, Vesicle-fusing ATPase
IPI00121341.1, Thioredoxin-related transmembrane protein 1
IPI00420726.3, 40S ribosomal protein S9
IPI00990363.1, 60S ribosomal protein L21-like
IPI00222767.1, "Pyruvate dehydrogenase protein X component, mitochondrial"
IPI00849793.1, 60S ribosomal protein L12
IPI00402981.2, Isoform 2 of 40S ribosomal protein S24
IPI00986371.1, 60S ribosomal protein L27a-like
IPI00113223.2, Fatty acid synthase
IPI00132424.4, X-ray repair cross-complementing protein 6
IPI00118963.1, "39S ribosomal protein L12, mitochondrial"
IPI00134599.1, 40S ribosomal protein S3
IPI00119618.1, Calnexin
IPI00122548.4, Voltage-dependent anion-selective channel protein 3
IPI00378120.2, "Glutaredoxin-related protein 5, mitochondrial"
IPI00759913.1, Isoform 11 of Kinectin
IPI00760003.2, kinectin
IPI00122549.1, Isoform Pl-VDAC1 of Voltage-dependent anion-selective channel protein 1
IPI00853964.2, Uncharacterized protein
IPI00626385.4, plectin isoform 12alpha
IPI00132042.1, "Pyruvate dehydrogenase E1 component subunit beta, mitochondrial"
IPI00830604.1, tropomyosin alpha-1 chain isoform 2
IPI00831330.1, tropomyosin alpha-1 chain isoform 6
IPI00944126.1, tropomyosin alpha-1 chain isoform 8
IPI00319992.1, 78 kDa glucose-regulated protein
IPI00848443.1, Isoform 2 of Acetyl-CoA carboxylase 1
IPI00129350.2, Isoform Long of Delta-1-pyrroline-5-carboxylate synthase
IPI00221398.2, Isoform Short of Delta-1-pyrroline-5-carboxylate synthase
IPI00319830.7, "Isoform 1 of Spectrin beta chain, brain 1"
IPI00121892.9, "Isoform 2 of Spectrin beta chain, brain 1"
IPI00788337.1, "Isoform 2 of Presequence protease, mitochondrial"
IPI00753815.3, "Isoform 1 of Spectrin alpha chain, brain"
IPI00968403.1, "spectrin alpha chain, brain isoform 3"
IPI00123223.2, Murinoglobulin-1
IPI00222977.2, Signal recognition particle 68 kDa protein
IPI00320459.2, Epiplakin
IPI00154054.1, "Acetyl-CoA acetyltransferase, mitochondrial"
IPI00663627.2, Filamin-B
IPI00337893.2, "Pyruvate dehydrogenase E1 component subunit alpha, somatic form, mitochondrial"
IPI00466570.4, Isoform 1 of Transmembrane emp24 domain-containing protein 10
IPI00135686.2, Peptidyl-prolyl cis-trans isomerase B
IPI00775791.1, Uncharacterized protein
IPI00121079.3, Isoform 1 of NADH-cytochrome b5 reductase 3
IPI00757312.1, Uncharacterized protein
IPI00969919.1, Uncharacterized protein
IPI00114209.1, "Glutamate dehydrogenase 1, mitochondrial"
IPI00109044.8, "myosin light chain, regulatory B-like"
IPI00130240.1, Peptidyl-prolyl cis-trans isomerase C
IPI00938530.1, myosin-11 isoform 1
IPI00123181.4, Myosin-9
IPI00875068.1, Uncharacterized protein
IPI00132705.1, Myosin regulatory light chain 12B
IPI00750595.2, Myosin regulatory light polypeptide 9
IPI00900442.1, Uncharacterized protein
IPI00316509.2, Epoxide hydrolase 1
IPI00265025.5, galactokinase
IPI00554894.3, Annexin A6
IPI00310240.4, annexin A6 isoform b
IPI00122223.1, DNA topoisomerase 2-alpha
IPI00128209.3, Isoform 1 of Adenylate kinase isoenzyme 1
IPI00762316.4, Isoform 1 of Guanine nucleotide-binding protein-like 1
IPI00109611.1, Protein FAM162A
IPI00222412.3, CAAX prenyl protease 1 homolog
IPI00453692.4, Isoform 1 of Nestin
IPI00122353.1, Isoform 2 of Glucosidase 2 subunit beta
IPI00127841.3, ADP/ATP translocase 2
IPI00115564.5, ADP/ATP translocase 1
IPI00169916.11, Clathrin heavy chain 1

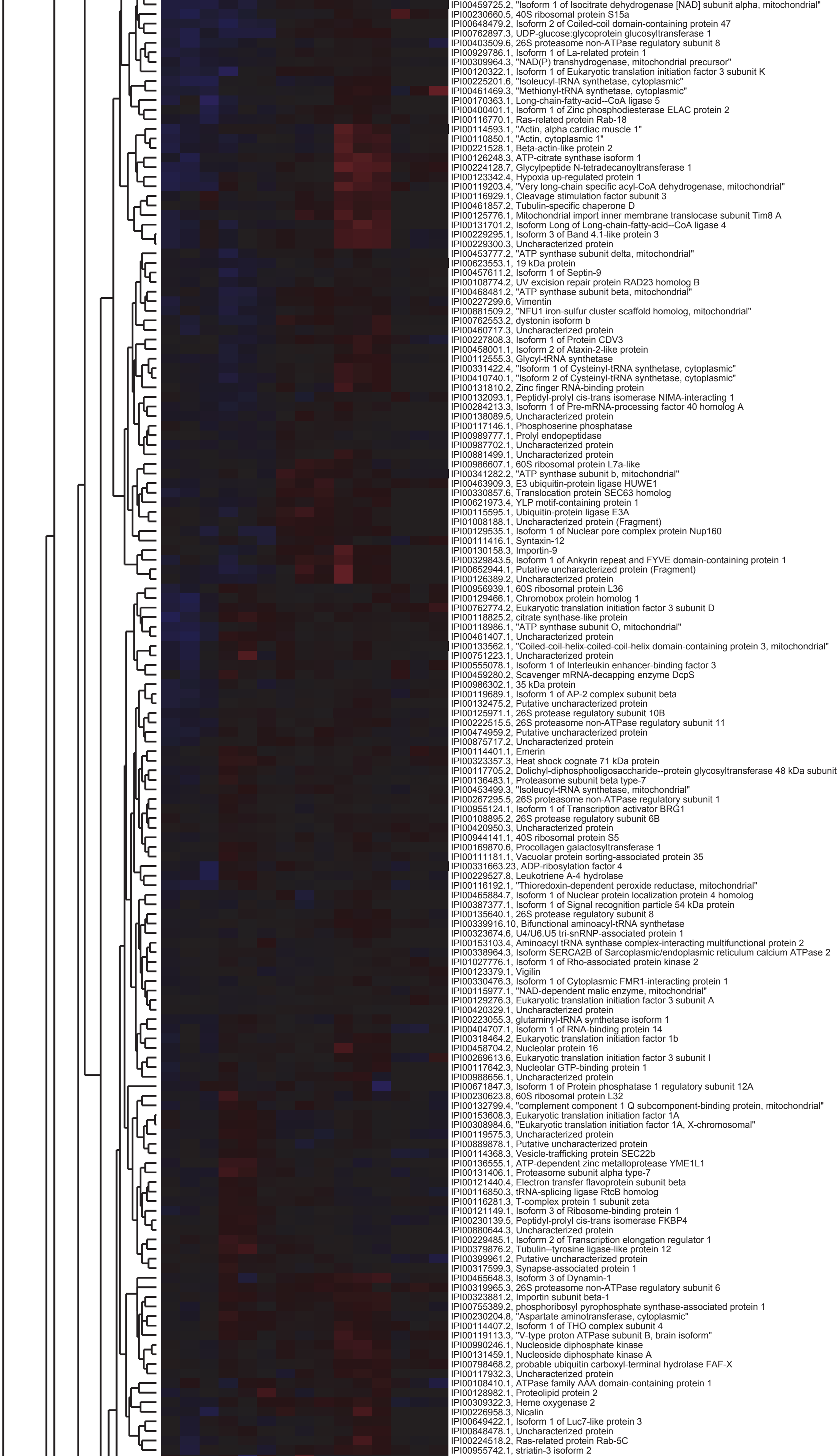
IPI00459725.2, "Isoform 1 of Isocitrate dehydrogenase [NAD] subunit alpha, mitochondrial"
IPI00230660.5, 40S ribosomal protein S15a
IPI00648479.2, Isoform 2 of Coiled-coil domain-containing protein 47
IPI00762897.3, UDP-glucose:glycoprotein glucosyltransferase 1
IPI00403509.6, 26S proteasome non-ATPase regulatory subunit 8
IPI00929786.1, Isoform 1 of La-related protein 1
IPI00309964.3, "NAD(P) transhydrogenase, mitochondrial precursor"
IPI00120322.1, Isoform 1 of Eukaryotic translation initiation factor 3 subunit K
IPI00225201.6, "Isoleucyl-tRNA synthetase, cytoplasmic"
IPI00461469.3, "Methionyl-tRNA synthetase, cytoplasmic"
IPI00170363.1, Long-chain-fatty-acid--CoA ligase 5
IPI00400401.1, Isoform 1 of Zinc phosphodiesterase ELAC protein 2
IPI00116770.1, Ras-related protein Rab-18
IPI00114593.1, "Actin, alpha cardiac muscle 1"
IPI00110850.1, "Actin, cytoplasmic 1"
IPI00221528.1, Beta-actin-like protein 2
IPI00126248.3, ATP-citrate synthase isoform 1
IPI00224128.7, Glycylpeptide N-tetradecanoyltransferase 1
IPI00123342.4, Hypoxia up-regulated protein 1
IPI00119203.4, "Very long-chain specific acyl-CoA dehydrogenase, mitochondrial"
IPI00116929.1, Cleavage stimulation factor subunit 3
IPI00461857.2, Tubulin-specific chaperone D
IPI00125776.1, Mitochondrial import inner membrane translocase subunit Tim8 A
IPI00131701.2, Isoform Long of Long-chain-fatty-acid--CoA ligase 4
IPI00229295.1, Isoform 3 of Band 4.1-like protein 3
IPI00229300.3, Uncharacterized protein
IPI00453777.2, "ATP synthase subunit delta, mitochondrial"
IPI00623553.1, 19 kDa protein
IPI00457611.2, Isoform 1 of Septin-9
IPI00108774.2, UV excision repair protein RAD23 homolog B
IPI00468481.2, "ATP synthase subunit beta, mitochondrial"
IPI00227299.6, Vimentin
IPI00881509.2, "NFU1 iron-sulfur cluster scaffold homolog, mitochondrial"
IPI00762553.2, dystonin isoform b
IPI00460717.3, Uncharacterized protein
IPI00227808.3, Isoform 1 of Protein CDV3
IPI00458001.1, Isoform 2 of Ataxin-2-like protein
IPI00112555.3, Glycyl-tRNA synthetase
IPI00331422.4, "Isoform 1 of Cysteinyl-tRNA synthetase, cytoplasmic"
IPI00410740.1, "Isoform 2 of Cysteinyl-tRNA synthetase, cytoplasmic"
IPI00131810.2, Zinc finger RNA-binding protein
IPI00132093.1, Peptidyl-prolyl cis-trans isomerase NIMA-interacting 1
IPI00284213.3, Isoform 1 of Pre-mRNA-processing factor 40 homolog A
IPI00138089.5, Uncharacterized protein
IPI00117146.1, Phosphoserine phosphatase
IPI00989777.1, Prolyl endopeptidase
IPI00987702.1, Uncharacterized protein
IPI00881499.1, Uncharacterized protein
IPI00986607.1, 60S ribosomal protein L7a-like
IPI00341282.2, "ATP synthase subunit b, mitochondrial"
IPI00463909.3, E3 ubiquitin-protein ligase HUWE1
IPI00330857.6, Translocation protein SEC63 homolog
IPI00621973.4, YLP motif-containing protein 1
IPI00115595.1, Ubiquitin-protein ligase E3A
IPI01008188.1, Uncharacterized protein (Fragment)
IPI00129535.1, Isoform 1 of Nuclear pore complex protein Nup160
IPI00111416.1, Syntaxin-12
IPI00130158.3, Importin-9
IPI00329843.5, Isoform 1 of Ankyrin repeat and FYVE domain-containing protein 1
IPI00652944.1, Putative uncharacterized protein (Fragment)
IPI00126389.2, Uncharacterized protein
IPI00956939.1, 60S ribosomal protein L36
IPI00129466.1, Chromobox protein homolog 1
IPI00762774.2, Eukaryotic translation initiation factor 3 subunit D
IPI00118825.2, citrate synthase-like protein
IPI00118986.1, "ATP synthase subunit O, mitochondrial"
IPI00461407.1, Uncharacterized protein
IPI00133562.1, "Coiled-coil-helix-coiled-coil-helix domain-containing protein 3, mitochondrial"
IPI00751223.1, Uncharacterized protein
IPI00555078.1, Isoform 1 of Interleukin enhancer-binding factor 3
IPI00459280.2, Scavenger mRNA-decapping enzyme DcpS
IPI00986302.1, 35 kDa protein
IPI00119689.1, Isoform 1 of AP-2 complex subunit beta
IPI00132475.2, Putative uncharacterized protein
IPI00125971.1, 26S protease regulatory subunit 10B
IPI00222515.5, 26S proteasome non-ATPase regulatory subunit 11
IPI00474959.2, Putative uncharacterized protein
IPI00875717.2, Uncharacterized protein
IPI00114401.1, Emerin
IPI00323357.3, Heat shock cognate 71 kDa protein
IPI00117705.2, Dolichyl-diphosphooligosaccharide--protein glycosyltransferase 48 kDa subunit
IPI00136483.1, Proteasome subunit beta type-7
IPI00453499.3, "Isoleucyl-tRNA synthetase, mitochondrial"
IPI00267295.5, 26S proteasome non-ATPase regulatory subunit 1
IPI00955124.1, Isoform 1 of Transcription activator BRG1
IPI00108895.2, 26S protease regulatory subunit 6B
IPI00420950.3, Uncharacterized protein
IPI00944141.1, 40S ribosomal protein S5
IPI00169870.6, Procollagen galactosyltransferase 1
IPI00111181.1, Vacuolar protein sorting-associated protein 35
IPI00331663.23, ADP-ribosylation factor 4
IPI00229527.8, Leukotriene A-4 hydrolase
IPI00116192.1, "Thioredoxin-dependent peroxide reductase, mitochondrial"
IPI00465884.7, Isoform 1 of Nuclear protein localization protein 4 homolog
IPI00387377.1, Isoform 1 of Signal recognition particle 54 kDa protein
IPI00135640.1, 26S protease regulatory subunit 8
IPI00339916.10, Bifunctional aminoacyl-tRNA synthetase
IPI00323674.6, U4/U6.U5 tri-snRNP-associated protein 1
IPI00153103.4, Aminoacyl tRNA synthase complex-interacting multifunctional protein 2
IPI00338964.3, Isoform SERCA2B of Sarcoplasmic/endoplasmic reticulum calcium ATPase 2
IPI01027776.1, Isoform 1 of Rho-associated protein kinase 2
IPI00123379.1, Vigilin
IPI00330476.3, Isoform 1 of Cytoplasmic FMR1-interacting protein 1
IPI00115977.1, "NAD-dependent malic enzyme, mitochondrial"
IPI00129276.3, Eukaryotic translation initiation factor 3 subunit A
IPI00420329.1, Uncharacterized protein
IPI00223055.3, glutaminyl-tRNA synthetase isoform 1
IPI00404707.1, Isoform 1 of RNA-binding protein 14
IPI00318464.2, Eukaryotic translation initiation factor 1b
IPI00458704.2, Nucleolar protein 16
IPI00269613.6, Eukaryotic translation initiation factor 3 subunit I
IPI00117642.3, Nucleolar GTP-binding protein 1
IPI00988656.1, Uncharacterized protein
IPI00671847.3, Isoform 1 of Protein phosphatase 1 regulatory subunit 12A
IPI00230623.8, 60S ribosomal protein L32
IPI00132799.4, "complement component 1 Q subcomponent-binding protein, mitochondrial"
IPI00153608.3, Eukaryotic translation initiation factor 1A
IPI00308984.6, "Eukaryotic translation initiation factor 1A, X-chromosomal"
IPI00119575.3, Uncharacterized protein
IPI00889878.1, Putative uncharacterized protein
IPI00114368.3, Vesicle-trafficking protein SEC22b
IPI00136555.1, ATP-dependent zinc metalloprotease YME1L1
IPI00131406.1, Proteasome subunit alpha type-7
IPI00121440.4, Electron transfer flavoprotein subunit beta
IPI00116850.3, tRNA-splicing ligase RtcB homolog
IPI00116281.3, T-complex protein 1 subunit zeta
IPI00121149.1, Isoform 3 of Ribosome-binding protein 1
IPI00230139.5, Peptidyl-prolyl cis-trans isomerase FKBP4
IPI00880644.3, Uncharacterized protein
IPI00229485.1, Isoform 2 of Transcription elongation regulator 1
IPI00379876.2, Tubulin--tyrosine ligase-like protein 12
IPI00399961.2, Putative uncharacterized protein
IPI00317599.3, Synapse-associated protein 1
IPI00465648.3, Isoform 3 of Dynamin-1
IPI00319965.3, 26S proteasome non-ATPase regulatory subunit 6
IPI00323881.2, Importin subunit beta-1
IPI00755389.2, phosphoribosyl pyrophosphate synthase-associated protein 1
IPI00230204.8, "Aspartate aminotransferase, cytoplasmic"
IPI00114407.2, Isoform 1 of THO complex subunit 4
IPI00119113.3, "V-type proton ATPase subunit B, brain isoform"
IPI00990246.1, Nucleoside diphosphate kinase
IPI00131459.1, Nucleoside diphosphate kinase A
IPI00798468.2, probable ubiquitin carboxyl-terminal hydrolase FAF-X
IPI00117932.3, Uncharacterized protein
IPI00108410.1, ATPase family AAA domain-containing protein 1
IPI00128982.1, Proteolipid protein 2
IPI00309322.3, Heme oxygenase 2
IPI00226958.3, Nicalin
IPI00649422.1, Isoform 1 of Luc7-like protein 3
IPI00848478.1, Uncharacterized protein
IPI00224518.2, Ras-related protein Rab-5C
IPI00955742.1, striatin-3 isoform 2

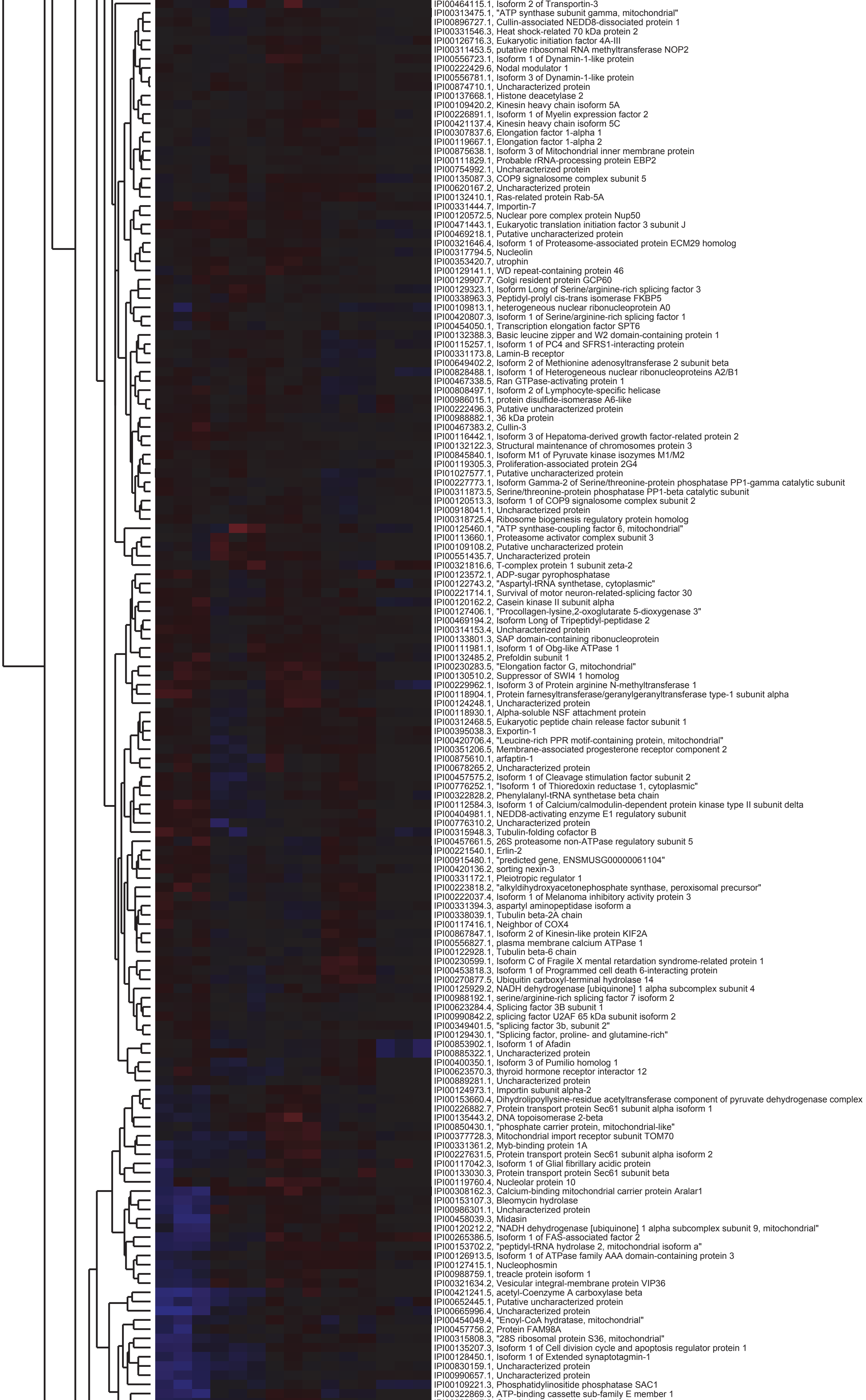
IPI00464115.1, Isoform 2 of Transportin-3
IPI00313475.1, "ATP synthase subunit gamma, mitochondrial"
IPI00896727.1, Cullin-associated NEDD8-dissociated protein 1
IPI00331546.3, Heat shock-related 70 kDa protein 2
IPI00126716.3, Eukaryotic initiation factor 4A-III
IPI00311453.5, putative ribosomal RNA methyltransferase NOP2
IPI00556723.1, Isoform 1 of Dynamin-1-like protein
IPI00222429.6, Nodal modulator 1
IPI00556781.1, Isoform 3 of Dynamin-1-like protein
IPI00874710.1, Uncharacterized protein
IPI00137668.1, Histone deacetylase 2
IPI00109420.2, Kinesin heavy chain isoform 5A
IPI00226891.1, Isoform 1 of Myelin expression factor 2
IPI00421137.4, Kinesin heavy chain isoform 5C
IPI00307837.6, Elongation factor 1-alpha 1
IPI00119667.1, Elongation factor 1-alpha 2
IPI00875638.1, Isoform 3 of Mitochondrial inner membrane protein
IPI00111829.1, Probable rRNA-processing protein EBP2
IPI00754992.1, Uncharacterized protein
IPI00135087.3, COP9 signalosome complex subunit 5
IPI00620167.2, Uncharacterized protein
IPI00132410.1, Ras-related protein Rab-5A
IPI00331444.7, Importin-7
IPI00120572.5, Nuclear pore complex protein Nup50
IPI00471443.1, Eukaryotic translation initiation factor 3 subunit J
IPI00469218.1, Putative uncharacterized protein
IPI00321646.4, Isoform 1 of Proteasome-associated protein ECM29 homolog
IPI00317794.5, Nucleolin
IPI00353420.7, utrophin
IPI00129141.1, WD repeat-containing protein 46
IPI00129907.7, Golgi resident protein GCP60
IPI00129323.1, Isoform Long of Serine/arginine-rich splicing factor 3
IPI00338963.3, Peptidyl-prolyl cis-trans isomerase FKBP5
IPI00109813.1, heterogeneous nuclear ribonucleoprotein A0
IPI00420807.3, Isoform 1 of Serine/arginine-rich splicing factor 1
IPI00454050.1, Transcription elongation factor SPT6
IPI00132388.3, Basic leucine zipper and W2 domain-containing protein 1
IPI00115257.1, Isoform 1 of PC4 and SFRS1-interacting protein
IPI00331173.8, Lamin-B receptor
IPI00649402.2, Isoform 2 of Methionine adenosyltransferase 2 subunit beta
IPI00828488.1, Isoform 1 of Heterogeneous nuclear ribonucleoproteins A2/B1
IPI00467338.5, Ran GTPase-activating protein 1
IPI00808497.1, Isoform 2 of Lymphocyte-specific helicase
IPI00986015.1, protein disulfide-isomerase A6-like
IPI00222496.3, Putative uncharacterized protein
IPI00988882.1, 36 kDa protein
IPI00467383.2, Cullin-3
IPI00116442.1, Isoform 3 of Hepatoma-derived growth factor-related protein 2
IPI00132122.3, Structural maintenance of chromosomes protein 3
IPI00845840.1, Isoform M1 of Pyruvate kinase isozymes M1/M2
IPI00119305.3, Proliferation-associated protein 2G4
IPI01027577.1, Putative uncharacterized protein
IPI00227773.1, Isoform Gamma-2 of Serine/threonine-protein phosphatase PP1-gamma catalytic subunit
IPI00311873.5, Serine/threonine-protein phosphatase PP1-beta catalytic subunit
IPI00120513.3, Isoform 1 of COP9 signalosome complex subunit 2
IPI00918041.1, Uncharacterized protein
IPI00318725.4, Ribosome biogenesis regulatory protein homolog
IPI00125460.1, "ATP synthase-coupling factor 6, mitochondrial"
IPI00113660.1, Proteasome activator complex subunit 3
IPI00109108.2, Putative uncharacterized protein
IPI00551435.7, Uncharacterized protein
IPI00321816.6, T-complex protein 1 subunit zeta-2
IPI00123572.1, ADP-sugar pyrophosphatase
IPI00122743.2, "Aspartyl-tRNA synthetase, cytoplasmic"
IPI00221714.1, Survival of motor neuron-related-splicing factor 30
IPI00120162.2, Casein kinase II subunit alpha
IPI00127406.1, "Procollagen-lysine,2-oxoglutarate 5-dioxygenase 3"
IPI00469194.2, Isoform Long of Tripeptidyl-peptidase 2
IPI00314153.4, Uncharacterized protein
IPI00133801.3, SAP domain-containing ribonucleoprotein
IPI00111981.1, Isoform 1 of Obg-like ATPase 1
IPI00132485.2, Prefoldin subunit 1
IPI00230283.5, "Elongation factor G, mitochondrial"
IPI00130510.2, Suppressor of SWI4 1 homolog
IPI00229962.1, Isoform 3 of Protein arginine N-methyltransferase 1
IPI00118904.1, Protein farnesyltransferase/geranylgeranyltransferase type-1 subunit alpha
IPI00124248.1, Uncharacterized protein
IPI00118930.1, Alpha-soluble NSF attachment protein
IPI00312468.5, Eukaryotic peptide chain release factor subunit 1
IPI00395038.3, Exportin-1
IPI00420706.4, "Leucine-rich PPR motif-containing protein, mitochondrial"
IPI00351206.5, Membrane-associated progesterone receptor component 2
IPI00875610.1, arfaptin-1
IPI00678265.2, Uncharacterized protein
IPI00457575.2, Isoform 1 of Cleavage stimulation factor subunit 2
IPI00776252.1, "Isoform 1 of Thioredoxin reductase 1, cytoplasmic"
IPI00322828.2, Phenylalanyl-tRNA synthetase beta chain
IPI00112584.3, Isoform 1 of Calcium/calmodulin-dependent protein kinase type II subunit delta
IPI00404981.1, NEDD8-activating enzyme E1 regulatory subunit
IPI00776310.2, Uncharacterized protein
IPI00315948.3, Tubulin-folding cofactor B
IPI00457661.5, 26S proteasome non-ATPase regulatory subunit 5
IPI00221540.1, Erlin-2
IPI00915480.1, "predicted gene, ENSMUSG00000061104"
IPI00420136.2, sorting nexin-3
IPI00331172.1, Pleiotropic regulator 1
IPI00223818.2, "alkyldihydroxyacetonephosphate synthase, peroxisomal precursor"
IPI00222037.4, Isoform 1 of Melanoma inhibitory activity protein 3
IPI00331394.3, aspartyl aminopeptidase isoform a
IPI00338039.1, Tubulin beta-2A chain
IPI00117416.1, Neighbor of COX4
IPI00867847.1, Isoform 2 of Kinesin-like protein KIF2A
IPI00556827.1, plasma membrane calcium ATPase 1
IPI00122928.1, Tubulin beta-6 chain
IPI00230599.1, Isoform C of Fragile X mental retardation syndrome-related protein 1
IPI00453818.3, Isoform 1 of Programmed cell death 6-interacting protein
IPI00270877.5, Ubiquitin carboxyl-terminal hydrolase 14
IPI00125929.2, NADH dehydrogenase [ubiquinone] 1 alpha subcomplex subunit 4
IPI00988192.1, serine/arginine-rich splicing factor 7 isoform 2
IPI00623284.4, Splicing factor 3B subunit 1
IPI00990842.2, splicing factor U2AF 65 kDa subunit isoform 2
IPI00349401.5, "splicing factor 3b, subunit 2"
IPI00129430.1, "Splicing factor, proline- and glutamine-rich"
IPI00853902.1, Isoform 1 of Afadin
IPI00885322.1, Uncharacterized protein
IPI00400350.1, Isoform 3 of Pumilio homolog 1
IPI00623570.3, thyroid hormone receptor interactor 12
IPI00889281.1, Uncharacterized protein
IPI00124973.1, Importin subunit alpha-2
IPI00153660.4, Dihydrolipoyllysine-residue acetyltransferase component of pyruvate dehydrogenase complex
IPI00226882.7, Protein transport protein Sec61 subunit alpha isoform 1
IPI00135443.2, DNA topoisomerase 2-beta
IPI00850430.1, "phosphate carrier protein, mitochondrial-like"
IPI00377728.3, Mitochondrial import receptor subunit TOM70
IPI00331361.2, Myb-binding protein 1A
IPI00227631.5, Protein transport protein Sec61 subunit alpha isoform 2
IPI00117042.3, Isoform 1 of Glial fibrillary acidic protein
IPI00133030.3, Protein transport protein Sec61 subunit beta
IPI00119760.4, Nucleolar protein 10
IPI00308162.3, Calcium-binding mitochondrial carrier protein Aralar1
IPI00153107.3, Bleomycin hydrolase
IPI00986301.1, Uncharacterized protein
IPI00458039.3, Midasin
IPI00120212.2, "NADH dehydrogenase [ubiquinone] 1 alpha subcomplex subunit 9, mitochondrial"
IPI00265386.5, Isoform 1 of FAS-associated factor 2
IPI00153702.2, "peptidyl-tRNA hydrolase 2, mitochondrial isoform a"
IPI00126913.5, Isoform 1 of ATPase family AAA domain-containing protein 3
IPI00127415.1, Nucleophosmin
IPI00988759.1, treacle protein isoform 1
IPI00321634.2, Vesicular integral-membrane protein VIP36
IPI00421241.5, acetyl-Coenzyme A carboxylase beta
IPI00652445.1, Putative uncharacterized protein
IPI00665996.4, Uncharacterized protein
IPI00454049.4, "Enoyl-CoA hydratase, mitochondrial"
IPI00457756.2, Protein FAM98A
IPI00315808.3, "28S ribosomal protein S36, mitochondrial"
IPI00135207.3, Isoform 1 of Cell division cycle and apoptosis regulator protein 1
IPI00128450.1, Isoform 1 of Extended synaptotagmin-1
IPI00830159.1, Uncharacterized protein
IPI00990657.1, Uncharacterized protein
IPI00109221.3, Phosphatidylinositide phosphatase SAC1
IPI00322869.3, ATP-binding cassette sub-family E member 1

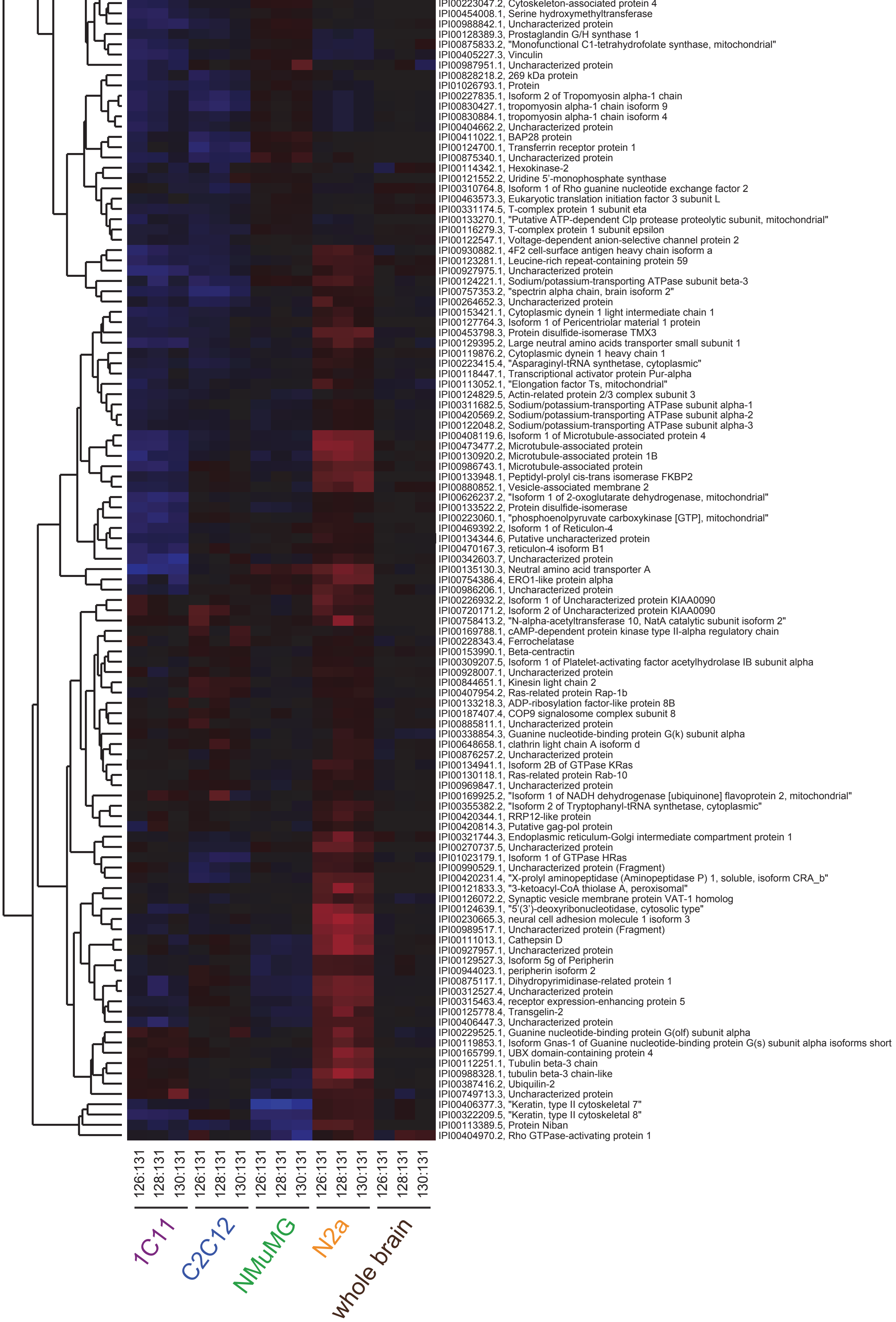
IPI00223047.2, Cytoskeleton-associated protein 4
IPI00454008.1, Serine hydroxymethyltransferase
IPI00988842.1, Uncharacterized protein
IPI00128389.3, Prostaglandin G/H synthase 1
IPI00875833.2, "Monofunctional C1-tetrahydrofolate synthase, mitochondrial"
IPI00405227.3, Vinculin
IPI00987951.1, Uncharacterized protein
IPI00828218.2, 269 kDa protein
IPI01026793.1, Protein
IPI00227835.1, Isoform 2 of Tropomyosin alpha-1 chain
IPI00830427.1, tropomyosin alpha-1 chain isoform 9
IPI00830884.1, tropomyosin alpha-1 chain isoform 4
IPI00404662.2, Uncharacterized protein
IPI00411022.1, BAP28 protein
IPI00124700.1, Transferrin receptor protein 1
IPI00875340.1, Uncharacterized protein
IPI00114342.1, Hexokinase-2
IPI00121552.2, Uridine 5'-monophosphate synthase
IPI00310764.8, Isoform 1 of Rho guanine nucleotide exchange factor 2
IPI00463573.3, Eukaryotic translation initiation factor 3 subunit L
IPI00331174.5, T-complex protein 1 subunit eta
IPI00133270.1, "Putative ATP-dependent Clp protease proteolytic subunit, mitochondrial"
IPI00116279.3, T-complex protein 1 subunit epsilon
IPI00122547.1, Voltage-dependent anion-selective channel protein 2
IPI00930882.1, 4F2 cell-surface antigen heavy chain isoform a
IPI00123281.1, Leucine-rich repeat-containing protein 59
IPI00927975.1, Uncharacterized protein
IPI00124221.1, Sodium/potassium-transporting ATPase subunit beta-3
IPI00757353.2, "spectrin alpha chain, brain isoform 2"
IPI00264652.3, Uncharacterized protein
IPI00153421.1, Cytoplasmic dynein 1 light intermediate chain 1
IPI00127764.3, Isoform 1 of Pericentriolar material 1 protein
IPI00453798.3, Protein disulfide-isomerase TMX3
IPI00129395.2, Large neutral amino acids transporter small subunit 1
IPI00119876.2, Cytoplasmic dynein 1 heavy chain 1
IPI00223415.4, "Asparaginyl-tRNA synthetase, cytoplasmic"
IPI00118447.1, Transcriptional activator protein Pur-alpha
IPI00113052.1, "Elongation factor Ts, mitochondrial"
IPI00124829.5, Actin-related protein 2/3 complex subunit 3
IPI00311682.5, Sodium/potassium-transporting ATPase subunit alpha-1
IPI00420569.2, Sodium/potassium-transporting ATPase subunit alpha-2
IPI00122048.2, Sodium/potassium-transporting ATPase subunit alpha-3
IPI00408119.6, Isoform 1 of Microtubule-associated protein 4
IPI00473477.2, Microtubule-associated protein
IPI00130920.2, Microtubule-associated protein 1B
IPI00986743.1, Microtubule-associated protein
IPI00133948.1, Peptidyl-prolyl cis-trans isomerase FKBP2
IPI00880852.1, Vesicle-associated membrane 2
IPI00626237.2, "Isoform 1 of 2-oxoglutarate dehydrogenase, mitochondrial"
IPI00133522.2, Protein disulfide-isomerase
IPI00223060.1, "phosphoenolpyruvate carboxykinase [GTP], mitochondrial"
IPI00469392.2, Isoform 1 of Reticulon-4
IPI00134344.6, Putative uncharacterized protein
IPI00470167.3, reticulon-4 isoform B1
IPI00342603.7, Uncharacterized protein
IPI00135130.3, Neutral amino acid transporter A
IPI00754386.4, ERO1-like protein alpha
IPI00986206.1, Uncharacterized protein
IPI00226932.2, Isoform 1 of Uncharacterized protein KIAA0090
IPI00720171.2, Isoform 2 of Uncharacterized protein KIAA0090
IPI00758413.2, "N-alpha-acetyltransferase 10, NatA catalytic subunit isoform 2"
IPI00169788.1, cAMP-dependent protein kinase type II-alpha regulatory chain
IPI00228343.4, Ferrochelatase
IPI00153990.1, Beta-centractin
IPI00309207.5, Isoform 1 of Platelet-activating factor acetylhydrolase IB subunit alpha
IPI00928007.1, Uncharacterized protein
IPI00844651.1, Kinesin light chain 2
IPI00407954.2, Ras-related protein Rap-1b
IPI00133218.3, ADP-ribosylation factor-like protein 8B
IPI00187407.4, COP9 signalosome complex subunit 8
IPI00885811.1, Uncharacterized protein
IPI00338854.3, Guanine nucleotide-binding protein G(k) subunit alpha
IPI00648658.1, clathrin light chain A isoform d
IPI00876257.2, Uncharacterized protein
IPI00134941.1, Isoform 2B of GTPase KRas
IPI00130118.1, Ras-related protein Rab-10
IPI00969847.1, Uncharacterized protein
IPI00169925.2, "Isoform 1 of NADH dehydrogenase [ubiquinone] flavoprotein 2, mitochondrial"
IPI00355382.2, "Isoform 2 of Tryptophanyl-tRNA synthetase, cytoplasmic"
IPI00420344.1, RRP12-like protein
IPI00420814.3, Putative gag-pol protein
IPI00321744.3, Endoplasmic reticulum-Golgi intermediate compartment protein 1
IPI00270737.5, Uncharacterized protein
IPI01023179.1, Isoform 1 of GTPase HRas
IPI00990529.1, Uncharacterized protein (Fragment)
IPI00420231.4, "X-prolyl aminopeptidase (Aminopeptidase P) 1, soluble, isoform CRA_b"
IPI00121833.3, "3-ketoacyl-CoA thiolase A, peroxisomal"
IPI00126072.2, Synaptic vesicle membrane protein VAT-1 homolog
IPI00124639.1, "5'(3')-deoxyribonucleotidase, cytosolic type"
IPI00230665.3, neural cell adhesion molecule 1 isoform 3
IPI00989517.1, Uncharacterized protein (Fragment)
IPI00111013.1, Cathepsin D
IPI00927957.1, Uncharacterized protein
IPI00129527.3, Isoform 5g of Peripherin
IPI00944023.1, peripherin isoform 2
IPI00875117.1, Dihydropyrimidinase-related protein 1
IPI00312527.4, Uncharacterized protein
IPI00315463.4, receptor expression-enhancing protein 5
IPI00125778.4, Transgelin-2
IPI00406447.3, Uncharacterized protein
IPI00229525.1, Guanine nucleotide-binding protein G(olf) subunit alpha
IPI00119853.1, Isoform Gnas-1 of Guanine nucleotide-binding protein G(s) subunit alpha isoforms short
IPI00165799.1, UBX domain-containing protein 4
IPI00112251.1, Tubulin beta-3 chain
IPI00988328.1, tubulin beta-3 chain-like
IPI00387416.2, Ubiquilin-2
IPI00749713.3, Uncharacterized protein
IPI00406377.3, "Keratin, type II cytoskeletal 7"
IPI00322209.5, "Keratin, type II cytoskeletal 8"
IPI00113389.5, Protein Niban
IPI00404970.2, Rho GTPase-activating protein 1
126:131
128:131
130:131
126:131
128:131
130:131
126:131
128:131
130:131
126:131
128:131
130:131
126:131
128:131
130:131
1C11
C2C12
NMuMG
N2a
whole brain
