# Supplementary material for: Prion Protein Deficiency Causes Diverse Proteome Shifts in Cell Models That Escape Detection in Brain Tissue
Source: PLoS One. 2016 Jun 21;11(6):e0156779. doi: 10.1371/journal.pone.0156779 (PMC4915660; doi:10.1371/journal.pone.0156779)

S3 Figure

Proteins detected in all samples exhibiting PrP-dependent log2 level changes of > [0.2] in more than 9 samples

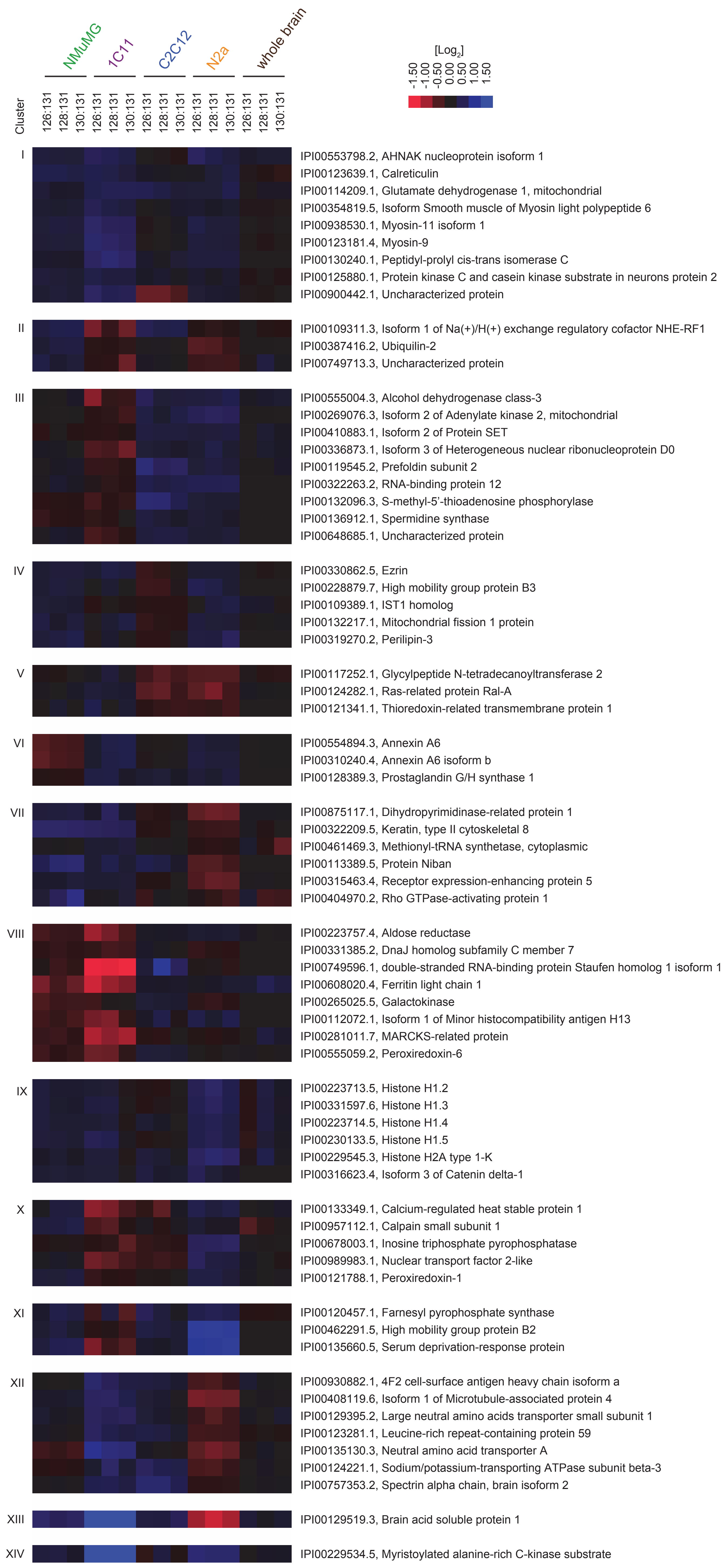

Supplement: S3 Fig — (PDF) [file pone.0156779.s003.pdf]
