# Supplementary material for: Prion Protein Deficiency Causes Diverse Proteome Shifts in Cell Models That Escape Detection in Brain Tissue
Source: PLoS One. 2016 Jun 21;11(6):e0156779. doi: 10.1371/journal.pone.0156779 (PMC4915660; doi:10.1371/journal.pone.0156779)

S5 Figure

Proteins exhibiting PrP-dependent log2 level changes of > [1.0] in at least 3 samples (including proteins not detected in all samples)

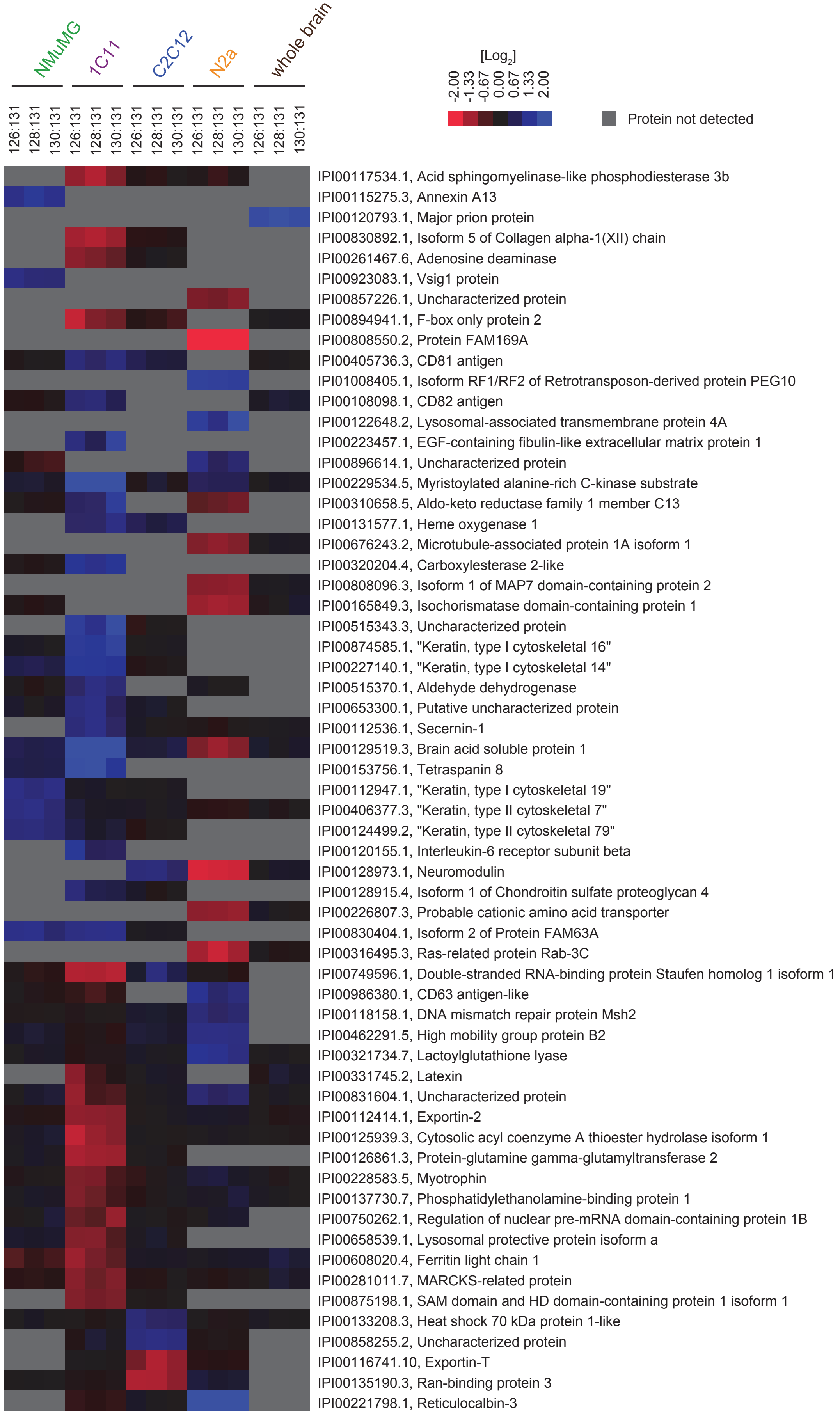

Supplement: S5 Fig — (PDF) [file pone.0156779.s005.pdf]
