# Supplementary material for: Prion Protein Deficiency Causes Diverse Proteome Shifts in Cell Models That Escape Detection in Brain Tissue
Source: PLoS One. 2016 Jun 21;11(6):e0156779. doi: 10.1371/journal.pone.0156779 (PMC4915660; doi:10.1371/journal.pone.0156779)
Supplement: S1 Table — (PDF) [file pone.0156779.s007.pdf]

S1 Table: Relative steady-state abundance levels of proteins detected in the five mouse models (only entries quantified on the basis of >3 TMT reporter ion ratios)

epithelial cells - NMuMG | neuroectodermal cells - 1C11 | muscle myoblasts - C2C12 | neuroblastoma cells - N2a | whole brain - tissue

Accession	Description	Coverage	# Proteins	# Unique Peptides	# Peptides	# PSMs	126/131 (wt/ko)	127/131 (ko/ko)	128/131 (wt/ko)	129/131 (ko/ko)	130/131 (wt/ko)	Count
IPI00263863.8	10 kDa heat shock protein, mitochondrial	97.06%	3	9	22	410	0.000	0.066	-0.091	0.040	-0.155	31
IPI01027722.1	10 kDa protein	100.00%	2	1	19	148	0.013	-0.092	0.057	0.010	-0.006	15
IPI00856284.1	102 kDa protein	69.51%	3	2	93	390	0.019	-0.223	0.166	-0.063	0.052	11
IPI00469260.3	116 kDa U5 small nuclear ribonucleoprotein component	86.10%	4	31	89	651	-0.097	0.041	-0.072	-0.001	-0.139	57
IPI00987091.1	12 kDa protein	30.39%	1	1	3	5						
IPI00987305.1	12 kDa protein	92.38%	6	12	24	300	-0.009	-0.010	-0.082	-0.007	-0.089	17
IPI00118757.1	14 kDa phosphohistidine phosphatase	83.87%	1	7	16	74	-0.056	-0.089	0.275	-0.154	-0.394	7
IPI00118384.1	14-3-3 protein epsilon	94.90%	2	11	40	1450	0.004	-0.059	0.013	-0.019	-0.003	117
IPI00227392.5	14-3-3 protein eta	80.89%	4	14	34	762	0.372	-0.009	0.424	0.029	0.454	45
IPI00230707.6	14-3-3 protein gamma	97.57%	2	15	40	822	0.041	-0.059	0.024	0.019	0.054	44
IPI00754545.3	14-3-3 protein theta-like	97.49%	2	1	53	1090	0.087	-0.086	0.134	-0.028	0.128	81
IPI00989726.1	14-3-3 protein theta-like	80.57%	1	1	44	946	0.132	-0.092	0.157	-0.034	0.162	85
IPI00116498.1	14-3-3 protein zeta/delta	100.00%	5	14	52	1514	0.128	-0.099	0.207	-0.014	0.190	108
IPI00112641.2	15 kDa selenoprotein	95.68%	2	3	12	34						
IPI00459443.5	182 kDa tankyrase-1-binding protein	68.37%	1	32	143	890	0.202	0.006	0.183	0.013	0.247	66
IPI00623553.1	19 kDa protein	95.00%	4	14	24	532	-0.010	0.120	0.040	0.061	-0.099	32
IPI00894804.1	19 kDa protein	53.89%	3	6	12	138	0.031	0.062	-0.077	-0.023	0.026	7
IPI00173168.1	1-acyl-sn-glycerol-3-phosphate acyltransferase delta	49.74%	1	3	18	44						
IPI00469338.4	1-acyl-sn-glycerol-3-phosphate acyltransferase gamma	46.54%	1	5	26	88						
IPI00311203.2	1-phosphatidylinositol-4,5-bisphosphate phosphodiesterase beta-3	64.51%	8	7	95	247	0.048	-0.020	0.207	-0.057	0.223	9
IPI00321515.2	1-phosphatidylinositol-4,5-bisphosphate phosphodiesterase delta-1	60.85%	1	6	50	118						
IPI00387379.1	2,4-dienoyl-CoA reductase, mitochondrial	95.22%	1	10	41	219	0.444	0.026	0.356	0.075	0.297	4
IPI00420284.3	2',5'-phosphodiesterase 12	69.08%	1	3	50	116	0.179	0.295	0.302	0.135	0.287	3
IPI00282848.1	20 kDa protein	87.29%	6	1	32	629	0.241	0.200	0.152	0.115	0.120	25
IPI01026590.1	20 kDa protein	78.69%	5	2	17	47						
IPI00990261.1	21 kDa protein	75.96%	2	3	16	55						
IPI01026824.1	212 kDa protein	66.56%	18	9	125	312						
IPI01027567.1	213 kDa protein	58.43%	1	2	126	272	-0.106	0.110	-0.292	-0.199	-0.022	3
IPI00987312.1	22 kDa protein	70.98%	2	2	20	161						
IPI00989100.1	22 kDa protein	99.01%	11	8	37	591	0.068	0.010	0.089	0.034	0.038	41
IPI01027072.1	24 kDa protein	79.06%	3	1	29	105	0.104	-0.063	0.143	0.019	0.141	3
IPI01027449.1	26 kDa protein	95.44%	1	1	43	146						
IPI00828218.2	269 kDa protein	46.59%	9	25	147	613	-0.146	-0.050	-0.119	-0.180	-0.180	30
IPI00125971.1	26S protease regulatory subunit 10B	92.54%	1	20	57	488	-0.034	-0.022	-0.026	0.011	0.014	37
IPI00133428.3	26S protease regulatory subunit 4	83.41%	1	23	55	592	0.007	-0.062	0.083	0.000	0.057	46
IPI00133206.2	26S protease regulatory subunit 6A	97.29%	4	25	63	806	-0.106	-0.068	0.037	0.014	-0.068	54
IPI00108895.2	26S protease regulatory subunit 6B	95.93%	1	23	48	661	-0.017	-0.028	-0.009	0.004	-0.041	47
IPI00135640.1	26S protease regulatory subunit 8	89.90%	3	18	56	462	0.013	0.000	0.037	0.040	-0.022	33
IPI00267295.5	26S proteasome non-ATPase regulatory subunit 1	87.30%	1	35	99	947	-0.031	-0.037	0.073	-0.037	-0.020	51
IPI00222515.5	26S proteasome non-ATPase regulatory subunit 11	91.23%	11	16	60	483	0.000	-0.028	-0.072	0.010	-0.048	43
IPI00318982.4	26S proteasome non-ATPase regulatory subunit 12	91.89%	5	18	68	510	-0.001	-0.057	-0.031	-0.007	-0.044	37
IPI00126048.2	26S proteasome non-ATPase regulatory subunit 13	99.20%	6	22	57	419	0.094	0.021	0.044	0.007	0.076	32
IPI00113262.1	26S proteasome non-ATPase regulatory subunit 14	86.45%	2	11	33	346	-0.012	-0.089	-0.004	-0.056	0.070	38
IPI00123494.3	26S proteasome non-ATPase regulatory subunit 2	93.50%	2	12	89	970	-0.082	0.019	-0.028	0.040	-0.057	76
IPI00314439.4	26S proteasome non-ATPase regulatory subunit 3	90.00%	2	24	80	579	-0.089	-0.080	-0.080	-0.017	-0.109	48
IPI00457661.5	26S proteasome non-ATPase regulatory subunit 5	97.42%	2	13	61	291	-0.014	0.009	0.197	0.003	0.147	10
IPI00319965.3	26S proteasome non-ATPase regulatory subunit 6	79.18%	1	16	48	235	-0.181	-0.041	-0.198	0.087	-0.269	14
IPI00114667.1	26S proteasome non-ATPase regulatory subunit 7	90.03%	1	12	33	287	-0.102	-0.089	-0.103	0.014	-0.095	20
IPI00403509.6	26S proteasome non-ATPase regulatory subunit 8	86.12%	1	13	39	518	-0.025	-0.035	0.112	0.043	-0.013	33
IPI00352475.3	28 kDa heat- and acid-stable phosphoprotein	96.13%	1	16	35	706	0.174	-0.017	0.181	0.003	0.229	65
IPI00120620.2	28S ribosomal protein S11, mitochondrial	86.39%	2	2	20	35	-0.138	-0.026	-0.283	-0.108	-0.198	3
IPI00321858.1	28S ribosomal protein S15, mitochondrial	87.98%	1	4	46	170	-0.120	-0.255	-0.188	0.079	0.255	3
IPI00132147.1	28S ribosomal protein S16, mitochondrial	78.52%	1	2	12	21	-0.013	0.044	-0.167	0.052	-0.112	3
IPI00132448.1	28S ribosomal protein S17, mitochondrial	79.17%	2	1	14	44	0.010	0.123	-0.088	-0.062	-0.180	3
IPI00110918.1	28S ribosomal protein S22, mitochondrial	76.60%	1	8	33	146	-0.306	-0.158	-0.186	-0.086	-0.211	3
IPI00284934.1	28S ribosomal protein S23, mitochondrial	80.23%	6	8	21	81	0.107	0.054	-0.054	-0.034	-0.100	9
IPI00315671.1	28S ribosomal protein S25, mitochondrial	77.78%	1	3	16	65	-0.119	-0.071	-0.290	0.048	-0.375	6
IPI00108194.1	28S ribosomal protein S26, mitochondrial	78.00%	1	5	27	152						
IPI00275050.2	28S ribosomal protein S29, mitochondrial isoform 2	84.09%	9	7	45	149	0.106	0.257	-0.039	0.123	-0.068	3
IPI00132504.1	28S ribosomal protein S30, mitochondrial	87.10%	1	6	41	122						
IPI00124828.1	28S ribosomal protein S31, mitochondrial	71.61%	1	7	33	84	-0.080	-0.139	-0.131	-0.109	-0.103	3
IPI00120709.1	28S ribosomal protein S34, mitochondrial	73.39%	1	6	25	76						
IPI00894878.1	28S ribosomal protein S35, mitochondrial precursor	91.56%	2	6	43	169	-0.086	0.140	-0.080	0.215	0.306	7
IPI00315808.3	28S ribosomal protein S36, mitochondrial	89.22%	3	5	9	83	0.072	0.024	-0.141	0.045	-0.022	3
IPI00330688.3	28S ribosomal protein S7, mitochondrial	79.75%	1	4	31	94	0.080	0.160	-0.109	-0.059	-0.080	6
IPI00336292.3	28S ribosomal protein S9, mitochondrial	76.41%	4	5	41	139						
IPI01023296.1	29 kDa protein	93.00%	4	2	32	690	-0.045	0.026	-0.047	0.000	-0.050	61
IPI01027089.1	29 kDa protein	43.97%	3	1	8	55						
IPI00136655.1	2-amino-3-ketobutyrate coenzyme A ligase, mitochondrial	62.50%	2	3	31	102						
IPI00331555.2	2-oxoisovalerate dehydrogenase subunit alpha, mitochondria	80.27%	1	6	38	106						
IPI00318545.3	3'(2'),5'-bisphosphate nucleotidase 1	90.26%	3	9	39	259	-0.116	-0.141	0.075	-0.047	-0.082	15
IPI01026945.1	34 kDa protein	72.41%	8	9	23	124	-0.608	0.106	-0.385	-0.178	-0.333	3
IPI00330329.3	3'-5' exoribonuclease 1	67.83%	1	6	37	79						
IPI00986302.1	35 kDa protein	76.28%	2	13	39	454	0.084	0.092	0.026	0.030	-0.029	27
IPI00988882.1	36 kDa protein	74.93%	6	5	34	737	-0.045	-0.003	0.038	-0.009	0.055	24
IPI00857911.1	38 kDa protein	60.00%	3	4	29	140						
IPI01027420.1	394 kDa protein	60.29%	8	1	226	586	-0.095	0.034	-0.023	-0.034	0.030	3
IPI00118237.2	39S ribosomal protein L1, mitochondrial	86.61%	2	7	39	143	-0.109	0.069	0.026	0.059	0.023	5
IPI00132412.3	39S ribosomal protein L10, mitochondrial	67.18%	2	5	17	60						
IPI00118963.1	39S ribosomal protein L12, mitochondrial	90.05%	1	6	27	317	-0.035	0.126	0.006	0.079	-0.069	24
IPI00133778.1	39S ribosomal protein L14, mitochondrial	88.28%	1	5	21	107	0.095	0.091	-0.039	-0.112	-0.059	8
IPI00118230.1	39S ribosomal protein L16, mitochondrial	94.82%	2	4	33	125	-0.003	0.250	-0.199	-0.050	-0.346	3
IPI00225318.3	39S ribosomal protein L22, mitochondrial	72.82%	1	3	21	90	-0.170	-0.171	-0.293	0.036	-0.175	5
IPI00118235.1	39S ribosomal protein L3, mitochondrial	75.86%	3	3	34	84						
IPI00162942.1	39S ribosomal protein L37, mitochondrial	66.67%	1	7	43	184	0.128	0.114	0.183	0.103	0.112	8
IPI00121359.4	39S ribosomal protein L4, mitochondrial	67.35%	1	3	23	89						
IPI00378520.4	39S ribosomal protein L41, mitochondrial	91.11%	2	5	17	45	-0.450	-0.053	-0.464	-0.025	-0.286	4
IPI00132895.1	39S ribosomal protein L45, mitochondrial	80.39%	3	4	31	140						
IPI00133750.1	39S ribosomal protein L53, mitochondrial	72.88%	1	4	14	62	-0.229	-0.034	-0.304	-0.059	-0.255	5
IPI00137471.3	3-beta-hydroxysteroid-Delta(8),Delta(7)-isomerase	33.91%	1	2	5	10						
IPI00322145.10	3-hydroxyacyl-CoA dehydratase 3	66.57%	1	7	26	158	0.247	-0.022	0.084	0.163	0.156	3
IPI00116222.1	3-hydroxyisobutyrate dehydrogenase, mitochondria	87.46%	2	8	29	262	0.029	0.153	0.059	0.072	-0.010	6
IPI00154047.1	3-hydroxyisobutyryl-CoA hydrolase, mitochondrial	95.58%	2	8	48	185						
IPI00121833.3	3-ketoacyl-CoA thiolase A, peroxisomal	98.11%	3	16	39	378	0.050	0.062	0.045	0.013	0.041	55
IPI00226430.3	3-ketoacyl-CoA thiolase, mitochondrial	97.98%	1	15	46	472	-0.059	0.017	-0.109	-0.013	-0.097	31
IPI00604945.2	3-mercaptopyruvate sulfurtransferase	63.64%	2	10	21	145	0.057	0.020	0.006	-0.085	0.024	8
IPI00112448.1	40S ribosomal protein S10	87.27%	2	14	25	394	0.041	-0.023	0.004	-0.003	0.052	39
IPI00473415.1	40S ribosomal protein S12-like	86.36%	10	7	16	219	0.169	0.088	0.193	-0.004	0.079	13
IPI00125901.5	40S ribosomal protein S13	86.09%	5	12	31	431	-0.001	-0.031	0.045	0.007	-0.044	33
IPI00322562.5	40S ribosomal protein S14	92.05%	3	5	25	374	0.119	-0.026	0.080	-0.044	-0.023	30
IPI00319231.4	40S ribosomal protein S15	76.55%	9	7	14	367	-0.012	-0.057	0.172	0.017	-0.062	48
IPI00230660.5	40S ribosomal protein S15a	98.46%	9	8	30	254	0.045	0.055	0.072	0.038	0.037	15
IPI00459875.4	40S ribosomal protein S15a-like	80.00%	2	1	32	137						
IPI00465880.4	40S ribosomal protein S17	99.26%	4	10	25	650	0.052	-0.100	0.110	-0.085	0.187	47
IPI00317590.5	40S ribosomal protein S18	90.79%	9	12	33	466	0.141	0.100	0.092	-0.019	0.091	26
IPI00665513.3	40S ribosomal protein S19-like	94.48%	2	4	33	577	0.171	0.041	0.139	0.041	0.076	57
IPI00987717.1	40S ribosomal protein S20-like, partial	95.65%	2	6	20	235	0.264	-0.189	0.223	-0.085	0.330	13
IPI00132950.1	40S ribosomal protein S21	97.59%	2	7	16	165	0.132	0.006	0.058	-0.019	0.134	14
IPI00972920.1	40S ribosomal protein S23	85.31%	5	7	23	351	0.068	0.096	0.095	0.036	0.069	34
IPI00131934.4	40S ribosomal protein S24-like	65.41%	1	1	15	37						
IPI00137735.1	40S ribosomal protein S25	77.60%	4	8	22	248	0.126	0.038	0.275	0.023	0.112	8
IPI00377441.3	40S ribosomal protein S26	92.17%	2	3	18	96	-0.012	0.179	-0.125	0.072	-0.236	3
IPI00124709.3	40S ribosomal protein S27-like	85.71%	4	2	12	103	-0.133	-0.045	-0.048	-0.019	-0.006	7
IPI00458629.2	40S ribosomal protein S2-like	98.98%	3	2	51	604	0.176	0.051	0.139	0.024	0.087	30
IPI00988101.1	40S ribosomal protein S2-like	95.56%	2	3	45	615	0.171	0.044	0.144	0.024	0.098	41
IPI00134599.1	40S ribosomal protein S3	95.06%	2	19	39	719	-0.028	-0.032	-0.095	0.010	-0.091	59
IPI00399483.1	40S ribosomal protein S30	83.05%	2	3	15	140	0.189	0.161	0.176	0.087	0.143	14
IPI00331345.5	40S ribosomal protein S3a	92.42%	3	23	48	685	0.112	0.029	0.170	0.087	0.103	55
IPI00331092.7	40S ribosomal protein S4, X isoform	90.87%	2	11	45	818	0.099	0.088	0.085	0.045	0.051	54
IPI00944141.1	40S ribosomal protein S5	85.78%	3	11	35	397	0.004	-0.108	-0.128	-0.102	0.034	23
IPI00113655.1	40S ribosomal protein S6	76.71%	5	15	45	500	0.170	0.045	0.087	0.058	0.068	45
IPI00136984.1	40S ribosomal protein S7	79.38%	2	12	22	615	0.070	0.003	0.021	0.009	0.045	46
IPI00466820.4	40S ribosomal protein S8	78.37%	9	11	28	381	0.045	-0.023	0.102	0.009	0.036	27
IPI00420726.3	40S ribosomal protein S9	79.90%	6	19	35	432	0.069	0.017	-0.026	-0.006	-0.044	46
IPI00123604.4	40S ribosomal protein SA	74.92%	4	5	28	611	0.016	0.043	0.079	0.044	0.003	41
IPI00659629.1	40S ribosomal protein SA-like isoform 1	72.88%	3	1	31	500	0.029	0.095	0.111	0.059	0.006	33
IPI01026927.1	45 kDa protein	66.16%	19	1	27	73						
IPI00775848.1	48 kDa protein	75.18%	13	2	30	85						
IPI00930882.1	4F2 cell-surface antigen heavy chain isoform a	74.69%	2	27	51	892	0.023	0.061	-0.032	-0.017	0.014	47
IPI00124372.3	4-trimethylaminobutyraldehyde dehydrogenase	85.71%	1	15	46	304	0.034	-0.065	-0.057	-0.086	-0.007	11
IPI00124639.1	5'(3')-deoxyribonucleotidase, cytosolic type	92.50%	2	9	19	117	0.047	-0.233	0.043	0.048	-0.032	8
IPI00137706.1	55 kDa erythrocyte membrane protein	92.49%	7	9	45	212	-0.071	-0.038	-0.155	0.000	0.013	10
IPI00556823.3	5'-AMP-activated protein kinase catalytic subunit alpha-1	70.66%	2	6	40	77	0.149	0.141	-0.218	-0.136	0.055	3
IPI00223185.5	5'-AMP-activated protein kinase subunit beta-1	80.37%	1	4	19	126	0.198	-0.158	-0.160	0.069	-0.035	3
IPI00119930.2	5'-AMP-activated protein kinase subunit gamma-1	79.70%	3	5	20	56	-0.038	0.048	-0.111	0.187	-0.218	5
IPI00648312.3	5'-nucleotidase domain-containing protein 3	84.43%	2	5	53	143						
IPI00169473.1	5-oxoprolinase	65.61%	2	2	71	188						
IPI00116360.3	60 kDa SS-A/Ro ribonucleoprotein	89.41%	1	3	51	155	-0.017	-0.083	-0.257	-0.050	-0.252	3
IPI00314950.2	60S acidic ribosomal protein P0	81.39%	3	2	38	772	0.130	0.011	0.075	0.014	0.044	58
IPI00113377.1	60S acidic ribosomal protein P1	96.49%	5	2	7	237						
IPI00139795.2	60S acidic ribosomal protein P2	96.52%	2	11	16	484	0.194	0.073	0.048	-0.006	0.122	24
IPI00849927.1	60S ribosomal protein L10a	84.33%	4	14	31	460	0.064	0.027	0.170	0.036	0.076	40
IPI00849598.1	60S ribosomal protein L10-like	82.24%	7	3	35	384	0.065	0.021	0.052	-0.004	0.019	22
IPI00403956.1	60S ribosomal protein L10-like isoform 2	85.05%	2	2	38	319	0.108	0.041	0.099	0.026	0.033	11
IPI00989506.1	60S ribosomal protein L11-like	75.84%	1	1	25	98	-0.390	-0.269	-0.074	-0.047	0.198	3
IPI00849793.1	60S ribosomal protein L12	90.91%	10	11	30	565	0.096	0.016	0.057	-0.001	0.055	44
IPI00223217.6	60S ribosomal protein L13a	85.71%	5	13	39	378	0.019	-0.026	0.050	0.021	0.040	23
IPI00988790.1	60S ribosomal protein L13-like	89.57%	7	11	37	432	0.188	0.024	0.040	-0.013	0.062	24
IPI00133185.3	60S ribosomal protein L14	81.57%	4	14	37	423	0.119	0.010	0.167	0.066	0.083	26
IPI00989676.1	60S ribosomal protein L15-like	76.88%	1	1	27	179	-0.045	-0.038	0.009	0.119	-0.044	13
IPI00989176.1	60S ribosomal protein L17-like	86.63%	6	12	30	585	0.114	0.098	0.165	0.029	0.130	33
IPI00555113.2	60S ribosomal protein L18	78.19%	5	9	36	380	0.069	0.072	0.034	0.003	-0.003	11
IPI00162790.1	60S ribosomal protein L18a	90.34%	5	10	34	319	-0.025	0.017	-0.053	-0.013	-0.006	25
IPI00122426.1	60S ribosomal protein L19	80.10%	4	11	38	451	0.160	0.047	0.162	0.006	0.111	32
IPI00990363.1	60S ribosomal protein L21-like	91.88%	17	8	29	489	0.083	0.047	0.135	0.038	-0.047	30
IPI00222546.5	60S ribosomal protein L22	80.47%	3	6	13	235	0.061	0.075	0.048	0.038	-0.031	14
IPI00139780.1	60S ribosomal protein L23	90.00%	4	9	24	372	0.096	-0.014	0.192	-0.044	0.087	25
IPI00849847.1	60S ribosomal protein L23a-like	71.79%	18	13	26	431	0.080	0.044	0.138	0.017	0.064	46
IPI00762051.1	60S ribosomal protein L24	87.26%	4	13	33	450	0.138	0.017	0.205	0.029	0.126	27
IPI00132460.1	60S ribosomal protein L26	90.34%	6	10	28	303	0.070	0.014	0.166	-0.003	0.085	28
IPI00122421.5	60S ribosomal protein L27	78.68%	6	9	18	351	0.079	0.089	0.057	0.036	0.001	26
IPI00986371.1	60S ribosomal protein L27a-like	79.14%	11	7	20	254	0.052	0.040	0.123	0.034	0.061	20
IPI00222547.6	60S ribosomal protein L28	89.78%	3	9	31	457	0.100	0.044	0.169	0.024	0.036	28
IPI00321170.9	60S ribosomal protein L3	88.83%	4	26	70	1549	0.069	0.017	0.091	0.020	0.077	90
IPI00990575.1	60S ribosomal protein L30	95.65%	9	10	23	315	-0.042	-0.041	0.007	-0.023	-0.053	14
IPI00677102.1	60S ribosomal protein L31-like isoform 1	79.03%	11	11	23	261	0.126	0.062	0.204	0.108	0.095	18
IPI00230623.8	60S ribosomal protein L32	91.11%	6	10	28	323	0.037	0.036	0.023	0.013	-0.102	20
IPI00466153.4	60S ribosomal protein L34	92.31%	5	5	31	277	0.076	0.094	0.016	0.047	-0.003	14
IPI00263879.4	60S ribosomal protein L35	73.98%	4	4	23	250	0.123	0.030	0.135	0.050	0.116	17
IPI00115902.2	60S ribosomal protein L35a	92.73%	9	8	25	324	0.054	0.068	-0.025	0.003	-0.031	25
IPI00956939.1	60S ribosomal protein L36	88.57%	12	6	25	232	0.017	-0.003	0.038	0.045	0.016	10
IPI00225066.8	60S ribosomal protein L36a	86.79%	1	4	18	157	0.143	0.004	0.391	0.154	0.154	6
IPI00453924.4	60S ribosomal protein L37	92.78%	3	6	20	105	0.080	0.103	0.134	-0.028	0.087	10
IPI00272545.5	60S ribosomal protein L37a	91.09%	1	1	20	123	-0.032	0.011	-0.026	-0.016	-0.085	12
IPI00331121.4	60S ribosomal protein L38	72.86%	5	6	12	173	0.001	-0.010	0.112	-0.019	0.013	18
IPI00111412.3	60S ribosomal protein L4	85.44%	1	32	74	1260	0.115	0.040	0.131	0.001	0.132	95
IPI00308706.4	60S ribosomal protein L5	88.55%	3	8	54	859	-0.154	-0.079	0.110	0.040	0.020	54
IPI00988575.1	60S ribosomal protein L5-like	87.90%	1	1	54	652	-0.158	-0.116	0.167	0.069	0.024	40
IPI00313222.5	60S ribosomal protein L6	89.86%	5	24	53	948	0.083	0.037	0.160	0.047	0.021	87
IPI00311236.1	60S ribosomal protein L7	90.74%	4	15	59	934	0.052	0.047	0.131	0.048	0.031	68
IPI00330363.8	60S ribosomal protein L7a	86.47%	15	10	46	855	0.075	0.044	0.026	-0.007	0.034	69
IPI00986607.1	60S ribosomal protein L7a-like	86.47%	3	1	39	616	-0.085	0.006	-0.317	-0.085	-0.285	23
IPI00990956.1	60S ribosomal protein L7a-like	80.22%	3	2	37	365	0.100	0.048	0.041	0.019	0.069	8
IPI00111695.1	60S ribosomal protein L7-like 1	83.74%	2	6	39	147						
IPI00137787.3	60S ribosomal protein L8	78.99%	1	15	38	451	0.081	0.000	0.158	0.057	0.064	23
IPI00122413.2	60S ribosomal protein L9	94.79%	6	11	29	573	0.052	0.068	0.124	0.058	-0.020	45
IPI01016108.1	69 kDa protein	59.67%	5	4	46	152						
IPI00554862.3	6-phosphofructokinase, liver type	85.64%	1	20	79	553	-0.184	-0.123	-0.131	-0.037	-0.120	25
IPI00331541.5	6-phosphofructokinase, muscle type	69.62%	1	25	67	535	-0.108	-0.069	-0.116	-0.072	-0.120	31
IPI00894579.1	6-phosphogluconate dehydrogenase, decarboxylating	87.37%	7	23	64	535	0.360	-0.086	0.373	0.003	0.417	19
IPI00132080.1	6-phosphogluconolactonase	96.50%	4	5	24	226	0.200	-0.188	0.013	-0.102	0.068	9
IPI00113584.2	76 kDa protein	72.44%	5	4	79	504						
IPI00319992.1	78 kDa glucose-regulated protein	85.80%	1	47	85	2177	0.075	0.059	0.103	0.041	0.057	165
IPI00130988.1	7-dehydrocholesterol reductase	28.87%	2	2	10	47						
IPI00322456.2	7SK snRNA methylphosphate capping enzyme	68.47%	1	2	36	81	-0.154	-0.023	-0.062	-0.013	-0.091	3
IPI00130339.3	Abhydrolase domain-containing protein 16A	43.55%	2	6	36	85						
IPI00380956.1	Abl interactor 2	56.73%	3	4	30	114						
IPI00135189.3	Acetoacetyl-CoA synthetase	72.02%	1	15	64	286	0.073	-0.068	0.001	-0.071	0.098	15
IPI00228253.2	Acetyl-CoA acetyltransferase, cytosolic	97.23%	5	10	39	298	0.096	-0.025	0.003	0.102	0.019	13
IPI00154054.1	Acetyl-CoA acetyltransferase, mitochondrial	82.78%	1	22	43	795	0.135	0.024	0.085	-0.026	0.043	39
IPI00421241.5	acetyl-Coenzyme A carboxylase beta	77.61%	2	1	182	567	-0.077	-0.034	0.034	0.043	-0.329	7
IPI00319858.1	Acetyl-coenzyme A transporter 1	74.91%	3	5	24	60	-0.139	-0.042	-0.077	-0.076	-0.007	5
IPI00125266.1	Acid ceramidase	54.57%	2	7	24	77						
IPI00117534.1	Acid sphingomyelinase-like phosphodiesterase 3b	63.38%	1	5	24	106						
IPI00314736.3	Acidic leucine-rich nuclear phosphoprotein 32 family member A	64.37%	5	10	30	248	0.070	-0.048	0.030	0.038	0.123	13
IPI00116074.1	Aconitate hydratase, mitochondrial	81.41%	1	42	88	1877	-0.108	0.051	-0.130	0.029	-0.147	121
IPI00114593.1	Actin, alpha cardiac muscle 1	98.14%	12	10	47	1750	0.080	0.030	0.165	0.020	0.094	85
IPI00110850.1	Actin, cytoplasmic 1	95.20%	18	11	42	2612	-0.007	-0.009	0.131	0.017	0.038	146
IPI00172197.2	Actin-binding protein anillin	79.66%	1	18	114	551	-0.388	0.010	-0.288	-0.047	-0.381	24
IPI00323660.1	Actin-like protein 6A	80.42%	4	12	40	232	0.099	-0.060	0.174	0.079	0.084	8
IPI00137206.2	Actin-related protein 10	82.01%	1	9	32	137	0.310	0.144	0.120	0.021	0.128	14
IPI00177038.1	Actin-related protein 2	78.43%	2	14	44	375	0.128	-0.079	0.236	0.027	0.181	28
IPI00127987.1	Actin-related protein 2/3 complex subunit 1A	63.78%	2	10	27	90						
IPI00661414.2	Actin-related protein 2/3 complex subunit 2	82.00%	2	16	41	307	0.118	0.061	0.038	0.037	0.083	29
IPI00124829.5	Actin-related protein 2/3 complex subunit 3	98.88%	4	9	28	179	0.073	-0.016	0.228	0.023	0.267	11
IPI00138691.6	Actin-related protein 2/3 complex subunit 4	89.88%	3	9	26	164	0.152	0.037	0.208	-0.053	0.156	10
IPI00399943.3	Actin-related protein 2/3 complex subunit 5	94.04%	3	9	21	187	0.380	-0.028	0.180	0.014	0.376	8
IPI00111117.5	Actin-related protein 2/3 complex subunit 5-like protein	69.93%	1	4	17	37						
IPI00115627.4	Actin-related protein 3	89.47%	1	16	44	459	0.126	-0.026	0.175	0.051	0.160	39
IPI00225633.3	Activated RNA polymerase II transcriptional coactivator p15	92.91%	1	8	24	146	-0.038	-0.082	-0.150	0.001	0.126	11
IPI00553687.6	activating signal cointegrator 1 complex subunit 3 isoform 2	53.50%	1	8	124	260	-0.063	0.057	-0.265	0.023	-0.039	5
IPI00153740.1	Activator of 90 kDa heat shock protein ATPase homolog 1	92.31%	1	17	44	405	0.124	0.038	0.189	-0.023	0.156	22
IPI00672180.1	activity-dependent neuroprotector homeobox protein	72.02%	1	14	106	460	-0.010	-0.274	0.095	0.112	-0.194	4
IPI00331710.2	Acyl-CoA dehydrogenase family member 9, mitochondrial	99.52%	3	17	87	398	0.001	-0.025	0.007	0.047	0.007	6
IPI00117142.2	Acyl-CoA desaturase 2	52.23%	5	5	21	99	0.009	0.180	-0.025	0.124	-0.134	12
IPI00122633.3	Acyl-CoA synthetase family member 2, mitochondrial	78.05%	2	10	59	245	-0.122	0.179	-0.097	-0.017	-0.082	5
IPI00857953.2	acyl-CoA-binding domain-containing protein 5 isoform a	43.81%	4	1	23	87	0.034	0.136	0.290	-0.026	0.059	3
IPI00128588.1	Acyl-coenzyme A thioesterase 11	68.69%	3	3	42	118						
IPI00132958.1	Acyl-coenzyme A thioesterase 13	85.00%	1	4	18	104	0.094	-0.106	-0.020	0.215	-0.112	5
IPI00136683.2	Acyl-coenzyme A thioesterase 2, mitochondrial	63.13%	10	6	29	126	0.116	-0.039	0.123	0.027	0.289	8
IPI00314069.1	Acyl-coenzyme A thioesterase 9, mitochondrial	84.51%	2	19	65	343	-0.001	0.068	-0.019	0.081	-0.094	16
IPI00880375.1	Acylphosphatase	92.45%	2	4	17	81						
IPI00881291.1	Acylphosphatase	95.54%	2	4	17	148	-0.162	0.095	0.055	0.128	0.088	9
IPI00123518.1	Acyl-protein thioesterase 2	83.55%	1	8	25	104						
IPI00225533.1	Adaptin ear-binding coat-associated protein 1	64.73%	1	6	24	127						
IPI00133798.1	Adaptin ear-binding coat-associated protein 2	51.13%	3	3	16	107	0.111	-0.209	0.130	-0.050	0.157	8
IPI00625950.2	adenine phosphoribosyltransferase	96.11%	2	14	20	152	0.064	-0.144	0.197	-0.006	0.152	16
IPI00261467.6	Adenosine deaminase	52.84%	1	7	17	65						
IPI00230440.6	Adenosylhomocysteinase	94.44%	2	21	49	448	-0.088	-0.044	-0.051	0.055	-0.127	29
IPI00313750.3	Adenylate cyclase type 9	51.37%	2	3	79	149						
IPI00125035.1	Adenylate kinase isoenzyme 4, mitochondrial	92.38%	4	6	21	75						
IPI00308217.2	Adenylosuccinate lyase	93.39%	4	19	61	479	-0.186	-0.114	-0.091	-0.085	-0.125	32
IPI00135969.2	Adenylosuccinate synthetase isozyme 2	82.02%	1	18	43	389	-0.012	-0.128	-0.063	-0.004	0.048	13
IPI00137331.7	Adenylyl cyclase-associated protein 1	77.22%	2	26	56	1031	0.108	-0.034	0.160	0.004	0.172	103

IPI00112001.1	Adenylyl cyclase-associated protein 2	73.95%	2	10	49	181
IPI00221998.4	Adipocyte plasma membrane-associated protein	84.82%	1	7	44	187	0.069
IPI00115564.5	ADP/ATP translocase 1	98.32%	1	11	54	765	0.009
IPI00127841.3	ADP/ATP translocase 2	97.65%	1	8	47	794	0.036
IPI00221613.5	ADP-ribosylation factor 1	94.48%	5	1	21	515	-0.065
IPI00221614.3	ADP-ribosylation factor 3	93.92%	5	1	21	533	-0.065
IPI00331663.23	ADP-ribosylation factor 4	86.11%	7	4	22	360	-0.176
IPI00221615.5	ADP-ribosylation factor 5	68.33%	4	4	19	374	-0.056
IPI00221616.5	ADP-ribosylation factor 6	94.29%	5	6	16	163	0.083
IPI00807733.2	ADP-ribosylation factor GTPase-activating protein 1 isoform d	95.01%	4	19	38	344	0.021
IPI00111931.2	ADP-ribosylation factor GTPase-activating protein 3	80.34%	2	9	64	212	0.273
IPI00137087.5	ADP-ribosylation factor guanine nucleotide-exchange factor 2	71.32%	2	7	158	445	-0.086
IPI00153201.1	ADP-ribosylation factor-binding protein GGA1	57.64%	1	5	39	227	
IPI00278498.10	ADP-ribosylation factor-like protein 1	83.98%	3	6	16	139	-0.080
IPI00315504.1	ADP-ribosylation factor-like protein 2	78.26%	1	5	16	85	0.116
IPI00124787.3	ADP-ribosylation factor-like protein 3	84.07%	1	10	22	197	0.029
IPI00123519.6	ADP-ribosylation factor-like protein 6-interacting protein 1	38.42%	1	4	12	75	
IPI00133218.3	ADP-ribosylation factor-like protein 8B	90.86%	1	2	25	139	-0.007
IPI00123572.1	ADP-sugar pyrophosphatase	66.97%	2	10	17	104	-0.111
IPI00135939.1	Adrenodoxin, mitochondrial	60.64%	2	4	15	57	
IPI00331490.6	Aflatoxin B1 aldehyde reductase member 2	76.29%	1	6	35	127	
IPI00117178.1	AH receptor-interacting protein	78.79%	2	9	29	103	0.094
IPI00553798.2	AHNAK nucleoprotein isoform 1	93.81%	6	255	484	6969	0.317
IPI00321739.1	A-kinase anchor protein 8	69.14%	1	11	49	251	
IPI00311509.1	Aladin	66.85%	1	7	36	178	-0.032
IPI00154045.3	Alanine aminotransferase 1	75.60%	1	3	45	161	-0.085
IPI00265352.1	Alanine aminotransferase 2	93.87%	2	13	61	254	
IPI00321308.4	Alanyl-tRNA synthetase, cytoplasmic	85.33%	1	45	104	1267	0.193
IPI00470086.1	Alanyl-tRNA synthetase, mitochondrial	61.63%	1	3	64	224	
IPI00466128.3	Alcohol dehydrogenase [NADP+]	76.62%	2	15	42	245	-0.035
IPI00134435.2	Alcohol dehydrogenase class 4 mu/sigma chain	68.98%	1	4	24	77	0.020
IPI00555004.3	Alcohol dehydrogenase class-3	83.69%	1	12	39	254	0.024
IPI00515370.1	Aldehyde dehydrogenase	77.63%	4	6	56	238	0.061
IPI00890112.2	Aldehyde dehydrogenase	69.60%	2	3	38	84	-0.860
IPI00153317.3	Aldehyde dehydrogenase family 1 member L1	83.59%	3	16	82	336	
IPI00169472.4	Aldehyde dehydrogenase family 1 member L2, mitochondria	88.52%	2	34	94	623	
IPI00458392.4	Aldehyde dehydrogenase family 16 member A1	35.16%	3	5	40	110	0.157
IPI00111218.1	Aldehyde dehydrogenase, mitochondria	89.60%	5	23	57	626	0.031
IPI00310658.5	Aldo-keto reductase family 1 member C13	74.61%	3	7	29	93	-0.022
IPI00223757.4	Aldose reductase	81.01%	5	15	38	374	-0.442
IPI00224181.7	Aldose reductase-related protein 1	66.14%	4	1	26	129	-0.092
IPI00273096.7	Aldose reductase-related protein 2	77.85%	4	5	27	134	-0.068
IPI00223818.2	alkyldihydroxyacetonephosphate synthase, peroxisomal precursor	85.10%	2	13	72	334	0.083
IPI00458418.2	Allograft inflammatory factor 1-like	88.67%	1	7	20	93	
IPI00153664.3	Alpha N-terminal protein methyltransferase 1A	87.89%	3	3	20	95	
IPI00121575.1	Alpha-1,3/1,6-mannosyltransferase ALG2	67.47%	2	7	34	81	
IPI00122285.3	Alpha-1,6-mannosyl-glycoprotein 2-beta-N-acetylglucosaminyltransferase	64.48%	1	4	37	168	
IPI00123920.2	Alpha-1-antitrypsin 1-3	54.85%	1	3	24	120	
IPI00751974.1	alpha-1-syntrophin	74.95%	2	3	40	95	
IPI00469307.2	Alpha-2-macroglobulin receptor-associated protein	77.78%	2	12	44	195	-0.109
IPI00380436.1	Alpha-actinin-1	95.40%	3	37	112	2137	0.183
IPI00387557.3	alpha-actinin-2	73.04%	3	5	90	680	0.223
IPI00136701.1	Alpha-actinin-3	91.00%	1	19	97	691	0.299
IPI00118899.1	Alpha-actinin-4	92.11%	4	24	117	2109	0.290
IPI00113895.1	Alpha-centractin	82.45%	2	6	43	351	0.317
IPI00138274.1	Alpha-crystallin B chain	56.57%	2	7	11	69	
IPI00987541.1	alpha-enolase-like isoform 7	97.95%	11	29	65	3037	-0.013
IPI00135965.2	Alpha-internexin	81.94%	2	30	66	471	
IPI00127450.1	Alpha-mannosidase 2C1	66.89%	4	4	58	191	
IPI00470003.3	Alpha-parvin	69.62%	2	7	40	184	0.179
IPI01008566.1	alpha-protein kinase 1	69.86%	2	2	89	359	-0.010
IPI00118930.1	Alpha-soluble NSF attachment protein	82.37%	1	13	34	275	-0.091
IPI00229849.2	Amidophosphoribosyltransferase	71.68%	1	10	45	159	-0.106
IPI00169711.2	Amine oxidase [flavin-containing] A	78.14%	4	15	47	278	-0.071
IPI00226140.6	Amine oxidase [flavin-containing] B	81.73%	3	11	46	182	
IPI00153103.4	Aminoacyl tRNA synthase complex-interacting multifunctional protein 2	100.00%	1	1	42	567	0.077
IPI00858183.1	aminoacyl tRNA synthase complex-interacting multifunctional protein 2 isof	100.00%	1	1	40	498	0.250
IPI00122529.2	Aminopeptidase B	75.23%	2	11	48	191	-0.146
**IPI00319509.5**	**Aminopeptidase N**	**71.95%**	**2**	**7**	**66**	**255**	
IPI00400180.1	Amphiphysin	54.66%	2	19	42	225	
IPI00226052.4	amyotrophic lateral sclerosis 2 (juvenile) chromosome region, candidate 11	67.50%	2	3	47	171	
IPI00120917.1	AN1-type zinc finger protein 6	52.91%	1	2	15	77	0.273
IPI00187301.1	Anamorsin	88.35%	4	7	23	351	0.119
IPI00127493.1	Anaphase-promoting complex subunit 4	65.92%	1	3	63	276	
IPI00115426.1	Anaphase-promoting complex subunit CDC26	84.71%	1	2	9	20	0.116
IPI00453820.2	Ankycorbin	89.89%	1	15	128	570	0.017
IPI00475362.2	ankyrin 3, epithelial isoform b	79.70%	12	14	177	548	
IPI01016174.1	ankyrin repeat and KH domain-containing protein 1	52.51%	4	2	150	392	-0.057
IPI00123306.2	Ankyrin repeat, SAM and basic leucine zipper domain-containing protein 1	57.05%	2	3	37	72	
IPI00230395.5	Annexin A1	95.38%	2	29	57	989	0.358
IPI00124264.2	Annexin A11	50.89%	3	11	39	178	-0.267
IPI00115275.3	Annexin A13	80.76%	8	7	33	137	1.197
IPI00468203.3	Annexin A2	98.53%	4	31	56	953	-0.017
IPI00132722.9	Annexin A3	100.00%	2	25	62	488	0.054
IPI00353727.8	Annexin A4	91.85%	4	20	47	386	0.166
IPI00317309.5	Annexin A5	95.61%	1	27	48	691	-0.219
IPI00554894.3	Annexin A6	93.76%	2	3	97	1474	-0.535
IPI00310240.4	annexin A6 isoform b	93.70%	2	3	97	1398	-0.565
IPI00114017.2	Annexin A7	58.96%	3	12	45	252	-0.074
IPI00132756.2	Annexin A8	96.33%	2	16	48	240	-0.211
IPI00850544.2	anoctamin-1	62.34%	4	7	69	170	0.161
IPI00346965.5	AP-1 complex subunit beta-1	79.53%	7	1	103	1210	0.011
IPI00621460.2	AP-1 complex subunit gamma-1	74.09%	2	16	96	481	-0.042
IPI00119680.3	AP-1 complex subunit mu-1	52.72%	4	10	26	97	
IPI00118026.1	AP-1 complex subunit sigma-1A	82.28%	4	3	17	60	0.151
IPI00310131.6	AP-2 complex subunit alpha-2	81.02%	1	31	110	1054	0.152
IPI00116356.5	AP-2 complex subunit mu	91.95%	2	24	66	605	0.245
IPI00118022.1	AP-2 complex subunit sigma	64.08%	2	8	16	116	-0.283
IPI00130444.2	AP-3 complex subunit beta-1	74.84%	2	13	118	466	-0.065
IPI00420426.1	AP-3 complex subunit beta-2	77.36%	2	24	113	453	
IPI00117811.3	AP-3 complex subunit delta-1	74.56%	1	18	96	439	
IPI00311387.1	AP-3 complex subunit mu-1	68.18%	2	1	30	131	-0.085
IPI00124286.2	AP-3 complex subunit mu-2	82.30%	1	2	36	88	
IPI00877236.1	apolipoprotein A-I preproprotein	66.29%	2	11	37	153	
IPI00170307.1	Apolipoprotein A-I-binding protein	70.21%	1	4	21	166	
IPI00377351.3	Apolipoprotein A-IV	77.97%	1	4	48	192	
IPI00323571.1	Apolipoprotein E	68.17%	5	10	39	173	
IPI00121576.2	Apolipoprotein O	90.57%	3	6	18	107	-0.103
IPI00120684.2	Apoptosis regulator BAX	72.92%	2	6	22	123	0.034
IPI00129577.1	Apoptosis-inducing factor 1, mitochondrial	76.63%	2	19	64	517	-0.017
IPI00470095.1	ARF GTPase-activating protein GIT1	65.32%	5	9	69	179	
IPI00875610.1	arfaptin-1	90.88%	3	2	46	197	-0.331
IPI00349032.2	ArfGAP with SH# domain, ankyrin repeat and PH domain1	75.76%	7	5	108	317	
IPI00310880.4	arginine/serine-rich splicing factor 6	76.40%	1	9	49	183	-0.047
IPI00314788.5	Argininosuccinate lyase	84.91%	4	7	46	151	
IPI00134746.5	Argininosuccinate synthase	89.32%	1	14	51	248	
IPI00315488.1	Arginyl-tRNA synthetase, cytoplasmic	86.82%	1	40	94	736	-0.076
IPI00131814.1	Aromatic-L-amino-acid decarboxylase	53.33%	1	3	31	78	
IPI00125143.2	Arpc1b protein	80.85%	4	8	41	335	0.245
IPI00515140.2	Arsenite methyltransferase	52.66%	1	2	19	64	
IPI00116966.8	Asparagine synthetase [glutamine-hydrolyzing]	85.92%	4	23	54	591	0.029
IPI00223415.4	Asparaginyl-tRNA synthetase, cytoplasmic	94.28%	2	28	67	687	0.115
IPI00230204.8	Aspartate aminotransferase, cytoplasmic	87.65%	2	25	50	644	-0.109
IPI00117312.1	Aspartate aminotransferase, mitochondrial	80.70%	1	30	56	917	0.072
IPI00331394.3	aspartyl aminopeptidase isoform a	74.53%	3	9	47	354	0.058
IPI00122743.2	Aspartyl-tRNA synthetase, cytoplasmic	87.23%	2	27	63	483	-0.178
IPI00123202.1	Ataxin-10	89.47%	3	19	55	359	-0.184
IPI00117229.3	ataxin-2	63.06%	5	14	100	462	-0.071
IPI00221754.3	Atlastin-1	63.62%	2	14	44	174	
IPI00323406.2	ATP synthase mitochondrial F1 complex assembly factor 1 precursor	74.43%	1	2	22	38	
IPI00130280.1	ATP synthase subunit alpha, mitochondrial	87.88%	4	40	89	1687	-0.149
IPI00341282.2	ATP synthase subunit b, mitochondrial	96.09%	1	11	32	287	-0.357
IPI00468481.2	ATP synthase subunit beta, mitochondrial	99.43%	1	33	61	1932	-0.136
IPI00453777.2	ATP synthase subunit delta, mitochondrial	95.24%	2	4	15	205	-0.041
IPI00111770.7	ATP synthase subunit e, mitochondrial	100.00%	2	8	14	116	-0.297
IPI00230241.6	ATP synthase subunit epsilon, mitochondrial	98.08%	1	3	11	48	-0.077
IPI00271986.6	ATP synthase subunit f, mitochondrial	71.59%	2	3	6	37	
IPI00313475.1	ATP synthase subunit gamma, mitochondrial	80.54%	7	17	45	536	-0.079
IPI00118986.1	ATP synthase subunit O, mitochondrial	95.31%	3	3	37	486	-0.136
IPI00125460.1	ATP synthase-coupling factor 6, mitochondrial	95.37%	3	6	19	375	-0.047
IPI00624501.2	ATPase Asna1	81.32%	1	13	28	270	0.000
IPI00108410.1	ATPase family AAA domain-containing protein 1	91.14%	1	10	40	214	-0.044
IPI00135252.2	ATPase family AAA domain-containing protein 2	69.06%	2	10	135	381	-0.293
IPI00120094.1	ATP-binding cassette sub-family B member 6, mitochondrial	57.13%	1	2	46	158	
IPI00672663.3	ATP-binding cassette sub-family B member 7, mitochondrial	69.68%	1	6	63	272	
IPI00459383.2	ATP-binding cassette sub-family B member 8, mitochondrial	73.50%	2	3	57	175	
IPI00135646.2	ATP-binding cassette sub-family D member 3	77.69%	3	10	58	323	-0.017
IPI00322869.3	ATP-binding cassette sub-family E member 1	78.96%	1	21	62	575	-0.257
IPI00396671.1	ATP-binding cassette sub-family F member 1	85.90%	2	28	86	823	-0.012
IPI00116825.1	ATP-binding cassette sub-family F member 2	75.32%	2	24	73	357	0.003
IPI00111686.6	ATP-binding cassette, sub-family C (CFTR/MRP), member 4 isoform 2	62.64%	2	7	112	290	-0.047
IPI00126248.3	ATP-citrate synthase isoform 1	88.28%	3	46	120	907	-0.037
IPI00119808.2	ATP-dependent Clp protease ATP-binding subunit clpX-like, mitochondria	88.01%	3	13	84	318	0.079
IPI00127172.3	ATP-dependent RNA helicase DDX1	80.27%	1	30	80	481	-0.016
IPI00459381.2	ATP-dependent RNA helicase DDX18	84.55%	2	23	73	462	-0.276
IPI00123651.2	ATP-dependent RNA helicase DDX19A	97.28%	3	2	70	378	-0.048
IPI00227749.1	ATP-dependent RNA helicase DDX19B isoform 1	92.51%	3	1	69	400	-0.013
IPI00113576.2	ATP-dependent RNA helicase DDX24 isoform 1	80.40%	2	8	97	335	
IPI00230035.7	ATP-dependent RNA helicase DDX3X	86.10%	2	10	83	870	0.081
IPI00134371.1	ATP-dependent RNA helicase DDX3Y	85.56%	1	1	79	696	0.085
IPI00117771.1	ATP-dependent RNA helicase DDX50	59.13%	4	6	63	172	
IPI00875042.1	ATP-dependent RNA helicase DDX54	81.69%	2	5	107	336	
IPI00410936.1	ATP-dependent RNA helicase Dhx29	80.37%	2	15	147	582	0.236
IPI00136555.1	ATP-dependent zinc metalloprotease YME1L1	83.22%	1	13	83	322	-0.083
IPI00124792.1	ATP-sensitive inward rectifier potassium channel 10	51.98%	2	3	27	57	
IPI00134095.3	baculoviral IAP repeat-containing 6	68.17%	1	36	315	1255	-0.022
IPI00130304.1	BAG family molecular chaperone regulator 2	90.48%	4	8	29	128	0.144
IPI00331334.4	BAG family molecular chaperone regulator 3	93.24%	2	26	63	453	-0.085
IPI00229727.3	BAG family molecular chaperone regulator 5	76.51%	2	5	43	93	
IPI00990293.1	band 4.1-like protein 2	88.26%	7	35	124	784	-0.082
IPI00411022.1	BAP28 protein	73.82%	1	21	191	879	-0.216
IPI00119959.1	Barrier-to-autointegration factor	94.38%	1	4	12	114	0.111
IPI00113824.1	Basement membrane-specific heparan sulfate proteoglycan core protein	64.28%	1	1	184	503	
IPI00515360.8	basement membrane-specific heparan sulfate proteoglycan core protein	66.78%	3	6	224	636	
IPI00132388.3	Basic leucine zipper and W2 domain-containing protein 1	91.65%	2	21	49	457	-0.149
IPI00387337.1	Basic leucine zipper and W2 domain-containing protein 2	89.02%	3	9	50	299	-0.170
IPI00970649.1	basophilic leukemia expressed protein BLES03	39.60%	2	2	15	43	0.337
IPI00119980.1	B-cell receptor-associated protein 29	64.17%	1	5	25	111	-0.035
IPI00230422.6	B-cell receptor-associated protein 31	81.22%	4	13	43	401	-0.051
IPI00119986.1	Bcl2 antagonist of cell death	70.59%	4	1	18	73	-0.039
IPI00309183.3	bcl-2 homologous antagonist/killer	86.12%	4	5	10	115	
IPI00321499.2	Bcl-2-like protein 13	47.24%	1	9	17	124	0.141
IPI00221528.1	Beta-actin-like protein 2	91.49%	1	3	45	1160	0.065
IPI00622716.4	Beta-catenin-like protein 1	64.48%	1	10	43	115	-0.175
IPI00153990.1	Beta-centractin	77.93%	3	3	38	328	0.079
IPI00228548.6	Beta-enolase	85.94%	6	13	50	855	-0.133
IPI00115530.1	Beta-hexosaminidase subunit beta	60.07%	1	3	38	100	
IPI00116221.2	Beta-lactamase-like protein 2	41.67%	1	3	16	57	0.151
IPI00268377.7	Beta-parvin	67.95%	1	7	32	119	-0.349
IPI00311515.2	Beta-soluble NSF attachment protein	92.62%	2	17	39	298	-0.091
IPI00131614.1	Beta-synuclein	59.40%	1	3	16	114	
IPI00308086.2	BH3-interacting domain death agonist	74.87%	1	3	12	32	0.091
IPI00395150.2	Bicaudal D-related protein 2	70.02%	1	2	60	156	
IPI00754489.4	Bifunctional 3'-phosphoadenosine 5'-phosphosulfate synthase 2	73.11%	2	9	46	110	
IPI00339916.10	Bifunctional aminoacyl-tRNA synthetase	80.69%	3	73	180	1846	-0.020
IPI00322610.5	Bifunctional coenzyme A synthase	69.09%	1	8	31	96	0.009
IPI00109824.1	Bifunctional methylenetetrahydrofolate dehydrogenase/cyclohydrolase, mi	92.57%	2	16	39	220	-0.025
IPI00330303.7	Bifunctional purine biosynthesis protein PURH	91.72%	1	31	73	772	-0.141
IPI00331262.2	bifunctional UDP-N-acetylglucosamine 2-epimerase/N-acetylmannosamine	60.96%	8	4	52	99	
IPI00123194.1	Biglycan	76.96%	1	4	34	73	
IPI00622875.3	Bile salt export pump	64.19%	1	2	81	192	
IPI00330627.5	Biliverdin reductase A	72.20%	4	8	26	112	0.044
IPI00753321.2	biorientation of chromosomes in cell division 1-like	63.29%	1	9	264	804	0.239
IPI00135345.3	Bis(5'-nucleosyl)-tetraphosphatase [asymmetrical]	60.54%	1	2	8	20	
IPI00153107.3	Bleomycin hydrolase	84.18%	4	7	50	264	-0.165
**IPI00111953.1**	**BolA-like protein 1**	**74.45%**	**1**	**3**	**16**	**46**	-0.267
IPI00129519.3	Brain acid soluble protein 1	96.02%	1	21	25	960	0.635
IPI00131896.1	Brain protein 44	85.83%	2	4	13	81	
IPI00124292.1	Brain protein 44-like protein	73.39%	2	3	10	33	
IPI00321643.4	Brain-specific angiogenesis inhibitor 1-associated protein 2-like protein 1	81.13%	1	12	66	221	0.131
IPI00895538.1	Branched-chain-amino-acid aminotransferase	62.18%	11	12	33	186	
IPI00122442.1	Branched-chain-amino-acid aminotransferase, mitochondrial	65.65%	4	5	34	141	
IPI00133648.2	BRCA2 and CDKN1A-interacting protein	60.13%	1	3	16	30	
IPI00380817.7	Breakpoint cluster region protein	70.87%	2	3	116	533	-0.240
IPI00330860.3	Breast carcinoma-amplified sequence 1 homolog	74.57%	3	15	63	269	
IPI00626782.2	brefeldin A-inhibited guanine nucleotide-exchange protein 1	52.82%	2	4	114	229	-0.165
IPI00623371.3	Brevican core protein	52.32%	2	18	46	178	
IPI00226771.4	BRI3-binding protein	72.33%	1	4	24	110	
IPI00408556.1	BRO1 domain-containing protein BROX	68.86%	3	6	38	119	-0.258
IPI00990135.1	bromodomain adjacent to zinc finger domain protein 1A-like	63.58%	5	9	149	461	0.214
IPI01027135.1	bromodomain adjacent to zinc finger domain protein 2A	45.47%	6	3	119	339	
IPI00133099.1	Bromodomain-containing protein 7	71.89%	1	4	49	122	
IPI00119130.1	BTB/POZ domain-containing protein 17	55.86%	2	3	25	63	
IPI00421206.1	BTB/POZ domain-containing protein KCTD12	78.59%	2	5	26	55	
IPI00675985.1	BTB/POZ domain-containing protein KCTD16	62.06%	1	3	29	79	
IPI00118762.6	Bystin	83.94%	1	7	50	154	0.068
IPI00122862.5	C-1-tetrahydrofolate synthase, cytoplasmic	84.17%	3	41	107	723	-0.103
IPI00222412.3	CAAX prenyl protease 1 homolog	62.95%	2	10	36	208	0.034
IPI00318626.1	Cadherin-1	40.95%	1	12	32	254	0.320
IPI00123746.2	Cadherin-13	57.70%	2	5	42	95	
IPI00323134.2	Cadherin-2	55.52%	2	11	52	287	0.193
IPI00123942.2	Cadherin-6	78.48%	3	3	53	131	0.504
IPI00331066.6	Calbindin	77.01%	1	15	28	174	
IPI00229604.4	calcium homeostasis endoplasmic reticulum protein	44.88%	1	10	55	191	-0.012
IPI00118900.1	Calcium/calmodulin-dependent 3',5'-cyclic nucleotide phosphodiesterase 1I	60.19%	3	8	37	98	
IPI00626535.3	Calcium/calmodulin-dependent protein kinase kinase 1	62.77%	2	2	37	108	
IPI00130439.1	Calcium/calmodulin-dependent protein kinase type 1	46.79%	2	3	18	147	
IPI00877328.1	calcium/calmodulin-dependent protein kinase type IV	67.59%	3	11	35	72	
IPI00308162.3	Calcium-binding mitochondrial carrier protein Aralar1	88.77%	1	32	67	694	-0.208
IPI00468924.5	Calcium-binding mitochondrial carrier protein SCaMC-1	81.47%	1	20	50	341	0.147
IPI00283600.2	Calcium-binding mitochondrial carrier protein SCaMC-3	75.59%	3	3	52	181	
IPI00318778.2	Calcium-binding protein 39	91.20%	4	14	50	248	0.336
IPI00229680.5	Calcium-binding protein p22	92.31%	5	6	27	102	-0.074
IPI00133349.1	Calcium-regulated heat stable protein 1	69.59%	2	3	15	67	0.054
IPI00115650.4	Calcyclin-binding protein	88.21%	1	14	32	365	-0.344
IPI00122450.1	caldesmon 1	87.92%	7	4	88	1313	-0.306
IPI00119618.1	Calnexin	66.67%	2	41	55	1354	-0.051
IPI00130992.1	Calpain small subunit 1	96.65%	2	2	23	178	0.310
IPI00957112.1	calpain small subunit 1	96.64%	3	1	23	195	0.310
IPI00626909.1	Calpain-1 catalytic subunit	78.12%	1	19	67	276	0.227
IPI00308938.6	Calpain-2 catalytic subunit	81.14%	2	27	63	447	0.229
IPI00132758.4	Calpain-6	59.44%	1	6	42	106	
IPI00116649.1	Calponin-2	96.07%	2	13	42	496	-0.224

IPI00119111.2	Calponin-3	97.58%	3	14	44	803	-0.128	-0.149	-0.181	0.000	-0.158	32	[illegible]
IPI00123639.1	Calreticulin	82.93%	1	30	40	1272	0.421	0.019	0.417	0.047	0.366	103	[illegible]
IPI00119346.1	Calretinin	74.17%	2	16	29	145							[illegible]
IPI00135186.1	Calumenin	86.67%	7	3	30	517	0.058	0.124	0.127	0.066	0.024	47	[illegible]
IPI00399958.3	calumenin isoform 2	94.29%	5	4	30	540	-0.026	0.084	0.079	0.019	-0.020	48	[illegible]
IPI00122486.3	CaM kinase-like vesicle-associated protein	67.97%	1	18	44	173							[illegible]
IPI00169788.1	cAMP-dependent protein kinase type II-alpha regulatory chain	79.85%	2	14	39	227	0.003	-0.170	-0.095	-0.025	-0.080	12	[illegible]
IPI00224570.3	cAMP-dependent protein kinase type II-beta regulatory subunit	81.73%	1	10	42	170	0.014	-0.109	-0.231	-0.165	-0.108	3	[illegible]
IPI00918646.1	cAMP-regulated phosphoprotein 19 isoform 2	91.03%	5	3	23	95	0.014	-0.241	0.167	-0.083	0.062	4	[illegible]
IPI00132339.1	Cancer-related nucleoside-triphosphatase homolog	85.26%	4	3	20	70							[illegible]
IPI00881332.1	caprin-1 isoform b	60.17%	5	22	54	805	0.026	0.088	0.036	0.066	0.024	61	[illegible]
IPI00459652.3	Cap-specific mRNA (nucleoside-2'-O-)-methyltransferase 1	69.77%	2	2	76	154							[illegible]
IPI00380280.3	carbamoyl-phosphate synthetase 2, aspartate transcarbamylase, and dihyd	81.57%	5	54	184	1125	-0.085	-0.060	-0.112	0.007	-0.120	73	[illegible]
IPI00112032.2	carbohydrate kinase domain-containing protein isoform 1	96.44%	3	9	42	162							[illegible]
IPI00798521.2	carbonic anhydrase 1	52.49%	2	4	12	40							[illegible]
IPI00121534.11	Carbonic anhydrase 2	80.00%	1	11	15	224							[illegible]
IPI00377496.5	Carbonic anhydrase-related protein	42.27%	1	5	10	46							[illegible]
IPI00314191.6	Carbonyl reductase [NADPH] 1	95.31%	4	14	42	300							[illegible]
IPI00169998.2	carbonyl reductase 3	80.51%	1	3	25	106							[illegible]
IPI00320204.4	carboxylesterase 2-like	56.25%	5	8	25	106	-0.114	-0.038	-0.188	-0.060	-0.116	9	[illegible]
IPI00119152.1	Carboxypeptidase E	71.85%	1	9	34	138							[illegible]
IPI00338309.5	Cardiac Ca2+ release channel	72.44%	4	6	427	1514							[illegible]
IPI00330094.4	Carnitine O-palmitoyltransferase 1, liver isoform	74.39%	1	4	64	161	0.031	-0.099	0.169	-0.100	0.179	5	[illegible]
IPI00111370.2	Cartilage-associated protein	70.25%	1	7	26	65							[illegible]
IPI00120162.2	Casein kinase II subunit alpha	75.70%	4	12	37	209	-0.245	-0.053	-0.099	-0.141	-0.079	11	[illegible]
IPI00118795.1	Casein kinase II subunit alpha'	79.71%	2	8	38	120	0.161	0.185	0.051	0.122	0.306	3	[illegible]
IPI00126762.1	Casein kinase II subunit beta	60.00%	5	3	16	123							[illegible]
IPI00308498.1	Caspase-3	91.70%	3	10	43	162	-0.128	-0.048	-0.228	-0.122	-0.041	9	[illegible]
IPI00131533.1	Caspase-8	80.00%	1	6	55	158	-0.004	0.059	0.003	-0.057	-0.088	5	[illegible]
IPI00312058.6	Catalase	82.73%	2	18	53	311	0.323	0.010	0.455	0.009	0.344	17	[illegible]
IPI00759876.2	catechol O-methyltransferase	85.28%	3	7	21	160	-0.131	-0.170	-0.056	-0.089	-0.094	9	[illegible]
IPI00222125.1	Catechol O-methyltransferase domain-containing protein 1	85.88%	1	4	25	75							[illegible]
IPI00112963.1	Catenin alpha-1	88.96%	1	35	120	1144	0.315	0.077	0.333	0.036	0.318	95	[illegible]
IPI00125899.1	Catenin beta-1	90.65%	6	26	82	748	0.229	-0.022	0.161	-0.022	0.220	52	[illegible]
IPI00228632.2	catenin delta-2	78.17%	3	11	115	420							[illegible]
IPI00113517.1	Cathepsin B	67.26%	1	11	26	203	0.140	-0.103	-0.020	0.029	0.068	9	[illegible]
IPI00111013.1	Cathepsin D	91.71%	4	6	40	407	0.280	-0.026	0.366	0.073	0.290	24	[illegible]
IPI00128154.1	Cathepsin L1	88.02%	2	8	28	169	0.703	0.095	0.901	0.198	0.576	20	[illegible]
IPI00986833.1	Cathepsin Z	53.27%	2	6	15	82							[illegible]
IPI00108844.1	Cation-dependent mannose-6-phosphate receptor	68.71%	2	7	29	158							[illegible]
IPI00308971.1	Cation-independent mannose-6-phosphate receptor	74.47%	1	8	184	580	0.282	-0.188	0.199	-0.117	0.203	9	[illegible]
IPI00125832.1	Caveolin-2	54.94%	3	4	7	59	0.061	0.140	0.344	0.000	0.160	9	[illegible]
IPI00221845.1	Caytaxin	28.23%	2	4	8	10							[illegible]
IPI00108330.2	CB1 cannabinoid receptor-interacting protein 1	97.56%	2	7	18	81							[illegible]
IPI00169667.1	CCR4-NOT transcription complex subunit 3	57.79%	2	6	42	121	-0.292	0.044	0.072	-0.130	0.062	8	[illegible]
IPI00266426.4	CD151 antigen	92.49%	1	3	18	77	0.154	0.219	0.075	0.044	0.016	3	[illegible]
IPI00268688.4	CD2-associated protein	69.39%	1	19	63	289	0.234	-0.060	0.152	-0.020	0.169	45	[illegible]
IPI00986380.1	CD63 antigen-like	59.24%	2	3	18	117	0.009	0.336	-0.160	0.224	-0.245	4	[illegible]
IPI00405736.3	CD81 antigen	53.39%	2	3	12	293	-0.127	-0.276	0.010	-0.105	-0.007	16	[illegible]
IPI00108098.1	CD82 antigen	46.99%	2	4	13	81	-0.236	-0.068	-0.267	-0.150	-0.112	3	[illegible]
IPI00221921.5	CD9 antigen	29.20%	1	3	6	19							[illegible]
IPI00471127.1	Cdc42 effector protein 1	68.70%	1	7	30	140	0.156	0.037	0.160	0.017	-0.006	7	[illegible]
IPI00124906.1	Cdc42 effector protein 4	48.71%	3	3	28	78							[illegible]
IPI00221894.2	CDC42 small effector protein 1	56.25%	1	1	3	17	0.968	0.257	0.423	0.152	0.776	3	[illegible]
IPI00128346.1	CDGSH iron-sulfur domain-containing protein 1	83.33%	1	5	14	187							[illegible]
IPI00132350.1	CDGSH iron-sulfur domain-containing protein 2	69.63%	2	5	16	54	-0.203	-0.238	-0.248	-0.381	-0.346	3	[illegible]
IPI00117025.1	CDK5 regulatory subunit-associated protein 3	64.41%	4	3	42	157							[illegible]
IPI00263028.1	CDKN2A-interacting protein	85.61%	1	6	60	333	-0.009	-0.057	-0.250	0.041	-0.318	5	[illegible]
IPI00348717.6	cDNA sequence BC005561	61.49%	2	1	127	295							[illegible]
IPI00162949.1	CDP-diacylglycerol--inositol 3-phosphatidyltransferase	67.61%	1	5	13	33							[illegible]
IPI00353932.6	Cdr2l protein	60.94%	1	1	38	73							[illegible]
IPI00118020.1	Cell adhesion molecule 3	57.32%	4	10	19	89							[illegible]
IPI00153840.2	Cell adhesion molecule 4	49.23%	1	6	18	109							[illegible]
IPI00387318.3	Cell cycle control protein 50A	44.51%	2	4	17	56							[illegible]
IPI00122826.1	Cell cycle exit and neuronal differentiation protein 1	71.14%	1	10	15	301							[illegible]
IPI00123602.2	Cell differentiation protein RCD1 homolog	92.64%	1	5	38	182	-0.010	0.223	-0.176	-0.025	-0.150	7	[illegible]
IPI00284444.5	Cell division cycle 5-related protein	85.41%	3	24	81	452	-0.037	-0.042	-0.130	-0.072	-0.120	39	[illegible]
IPI00113232.3	Cell growth-regulating nucleolar protein	65.21%	5	16	44	355	-0.047	0.148	-0.059	0.031	-0.120	31	[illegible]
**IPI00133687.4**	**Cellular retinoic acid-binding protein 2**	**96.38%**	**2**	**6**	**21**	**79**							[illegible]
IPI00406306.1	Cellular tumor antigen p53	57.80%	3	8	27	101							[illegible]
IPI00378768.5	centaurin, alpha 1	67.91%	2	5	26	41							[illegible]
IPI00128741.1	Centrin-2	71.51%	4	2	17	81							[illegible]
IPI00620180.3	Centromere/kinetochore protein zw10 homolog	79.97%	1	9	69	222							[illegible]
IPI00896626.2	centrosome-associated protein CEP250 isoform 2	80.48%	5	3	301	1153							[illegible]
IPI00170255.2	Ceramide kinase	58.57%	2	1	33	70							[illegible]
IPI00605187.4	ceruloplasmin isoform a	49.54%	4	6	57	159	0.219	-0.077	0.106	0.023	0.149	16	[illegible]
IPI00623742.2	cGMP-dependent 3',5'-cyclic phosphodiesterase	58.84%	5	19	50	178							[illegible]
IPI00122318.3	Charged multivesicular body protein 1a	64.29%	1	3	26	55							[illegible]
IPI00222386.3	Charged multivesicular body protein 2b	67.14%	1	4	33	139	0.446	-0.092	0.490	-0.102	0.352	7	[illegible]
IPI00347275.3	Charged multivesicular body protein 3	87.95%	1	3	29	66	0.072	0.115	0.152	0.106	0.098	4	[illegible]
IPI00171974.1	Charged multivesicular body protein 4b	70.09%	1	10	26	294	0.170	0.001	0.214	0.043	0.154	33	[illegible]
IPI00110729.1	Charged multivesicular body protein 5	71.69%	1	4	22	191	0.065	0.030	0.114	0.019	0.305	4	[illegible]
IPI00110956.6	Charged multivesicular body protein 6	67.00%	2	3	23	101							[illegible]
IPI00467127.5	chitinase domain-containing protein 1 isoform 1	46.46%	3	3	23	58							[illegible]
IPI00649035.1	chloride channel CLIC-like protein 1 isoform 1 precursor	63.60%	3	6	33	86							[illegible]
IPI00130344.3	Chloride intracellular channel protein 1	97.51%	4	14	30	510	0.165	-0.109	0.223	-0.013	0.249	48	[illegible]
IPI00135977.3	Chloride intracellular channel protein 4	98.81%	5	12	31	421	0.027	-0.063	0.144	-0.001	0.083	45	[illegible]
IPI00126085.1	Cholesterol 24-hydroxylase	72.60%	1	4	40	91							[illegible]
IPI00132770.1	Chromatin assembly factor 1 subunit B	70.28%	1	7	51	188	0.016	0.139	-0.125	0.079	-0.147	5	[illegible]
IPI00677454.2	Chromobox homolog 3	87.43%	7	3	23	349	0.116	0.108	0.167	0.072	0.059	28	[illegible]
IPI00129466.1	Chromobox protein homolog 1	76.76%	3	9	16	215	0.020	0.041	0.014	-0.006	-0.042	20	[illegible]
IPI00985974.1	chromobox protein homolog 3-like	85.79%	8	1	22	245	0.114	0.138	0.120	0.065	0.044	21	[illegible]
IPI00123755.1	Chromobox protein homolog 5	77.49%	1	11	26	203	0.131	-0.017	0.204	0.073	0.301	18	[illegible]
IPI00845761.2	chromodomain helicase DNA binding protein 2	61.14%	1	1	136	387	-0.041	-0.117	-0.041	-0.076	-0.157	3	[illegible]
IPI00457724.1	chromodomain helicase DNA binding protein 6	59.94%	3	3	183	534							[illegible]
IPI00107999.2	Chromodomain-helicase-DNA-binding protein 1	62.54%	3	4	148	466	-0.383	-0.262	-0.175	-0.022	-0.586	6	[illegible]
IPI00109419.2	Chromosome-associated kinesin KIF4	86.19%	3	18	157	735	-0.155	0.114	-0.076	0.124	0.059	12	[illegible]
IPI00331601.4	Cirhin	75.07%	1	5	58	182							[illegible]
IPI00153903.6	Citrate lyase subunit beta-like protein, mitochondrial	85.50%	2	5	32	79							[illegible]
IPI00113141.1	Citrate synthase, mitochondrial	83.84%	1	14	54	1017	-0.044	0.020	0.004	0.033	-0.037	44	[illegible]
IPI00118825.2	citrate synthase-like protein	72.32%	1	1	48	565	-0.141	-0.038	-0.091	0.044	-0.009	17	[illegible]
IPI00987448.1	C-Jun-amino-terminal kinase-interacting protein 4 isoform 6	73.33%	16	13	120	518	-0.062	-0.013	-0.042	0.092	-0.026	8	[illegible]
IPI00169916.11	Clathrin heavy chain 1	86.63%	3	101	181	3832	-0.014	-0.022	-0.045	0.004	-0.026	247	[illegible]
IPI00754096.1	clathrin interactor 1	41.73%	5	13	37	256	0.103	0.034	0.210	0.016	0.176	14	[illegible]
IPI00648658.1	clathrin light chain A isoform d	48.15%	4	1	22	302	0.026	0.064	0.019	0.079	0.107	26	[illegible]
IPI00131933.1	Claudin-2	43.04%	1	4	10	35	0.312	0.034	0.175	0.014	0.180	11	[illegible]
IPI00944213.1	cleavage and polyadenylation specificity factor subunit 1 isoform 1	62.21%	2	10	103	319							[illegible]
IPI00314302.3	Cleavage and polyadenylation specificity factor subunit 2	66.88%	1	5	60	169							[illegible]
IPI00135099.2	Cleavage and polyadenylation specificity factor subunit 3	69.15%	2	6	49	168	-0.167	0.048	-0.097	0.111	-0.059	6	[illegible]
IPI00132473.3	Cleavage and polyadenylation specificity factor subunit 5	78.41%	1	13	26	278	0.128	0.043	-0.028	0.000	0.029	13	[illegible]
IPI00421085.1	Cleavage and polyadenylation specificity factor subunit 6	46.64%	1	11	39	277	0.126	0.128	0.000	0.135	0.047	20	[illegible]
IPI00467932.4	Cleavage stimulation factor subunit 2 tau variant	67.72%	1	2	39	147							[illegible]
IPI00116929.1	Cleavage stimulation factor subunit 3	68.48%	2	7	56	153	0.020	0.077	-0.168	0.054	-0.069	6	[illegible]
IPI00121627.3	Cleft lip and palate transmembrane protein 1 homolog	60.99%	1	14	39	262	0.065	0.099	0.014	-0.010	-0.100	18	[illegible]
IPI00880482.1	CLIP-associating protein 1 isoform 2	91.42%	3	21	196	845	0.106	-0.028	-0.069	-0.109	-0.068	14	[illegible]
IPI00407863.6	CLIP-associating protein 2 c	84.61%	9	18	169	653							[illegible]
IPI00885921.2	Clk2-Scamp3 protein	49.09%	5	1	52	206	0.249	0.095	0.261	-0.094	0.263	4	[illegible]
IPI00320420.3	Clusterin	53.57%	6	8	29	74							[illegible]
IPI00132575.3	Coactosin-like protein	92.96%	1	9	20	152	0.026	-0.213	0.165	-0.034	0.136	15	[illegible]
IPI00229834.4	Coatomer subunit alpha	85.13%	5	33	132	801	0.033	-0.034	-0.082	-0.044	-0.063	54	[illegible]
IPI00120503.1	Coatomer subunit beta	83.53%	3	38	103	729	0.085	0.009	0.029	0.000	0.047	56	[illegible]
IPI00115097.1	Coatomer subunit beta'	90.61%	1	30	86	571	-0.001	-0.031	-0.010	0.021	-0.006	34	[illegible]
IPI00313558.5	Coatomer subunit delta	76.32%	1	18	68	448	-0.037	-0.023	-0.071	-0.035	-0.116	27	[illegible]
IPI00130840.7	Coatomer subunit epsilon	95.78%	4	10	31	157	0.070	-0.088	0.116	0.111	0.024	16	[illegible]
IPI00223437.5	Coatomer subunit gamma	89.13%	4	26	87	587	-0.009	-0.047	-0.141	-0.031	-0.149	40	[illegible]
IPI00139637.1	Coatomer subunit zeta-1	95.48%	2	3	15	82	0.050	-0.175	0.219	-0.079	0.418	8	[illegible]
IPI00119397.1	Coatomer subunit zeta-2	37.56%	1	3	7	12							[illegible]
IPI00266188.6	Cofilin-2	95.78%	1	9	26	317	0.139	-0.066	0.238	0.021	0.195	16	[illegible]
IPI00321848.6	Coiled-coil and C2 domain-containing protein 1A	67.02%	3	3	82	240							[illegible]
IPI00649691.3	Coiled-coil and C2 domain-containing protein 1B	73.58%	2	6	79	300	0.058	-0.054	0.276	0.027	0.288	11	[illegible]
IPI00226249.1	Coiled-coil domain-containing protein 104	82.80%	2	4	33	139							[illegible]
IPI00319221.3	Coiled-coil domain-containing protein 12	66.87%	1	4	12	35							[illegible]
IPI00458723.2	Coiled-coil domain-containing protein 124	85.71%	1	4	34	85							[illegible]
IPI00153742.3	Coiled-coil domain-containing protein 25	59.13%	1	1	21	56	-0.300	-0.082	0.014	-0.123	0.355	3	[illegible]
IPI00135208.1	Coiled-coil domain-containing protein 56	89.81%	2	2	12	56							[illegible]
IPI00169469.1	Coiled-coil domain-containing protein 72	85.94%	5	3	12	58	0.003	-0.066	0.158	-0.053	-0.114	5	[illegible]
IPI00402914.2	Coiled-coil domain-containing protein 86	54.23%	1	3	30	85							[illegible]
IPI00831329.1	coiled-coil domain-containing protein 93 isoform c	70.22%	2	3	67	228	0.055	-0.034	-0.029	-0.042	0.004	5	[illegible]
IPI00133391.1	Coiled-coil domain-containing protein 96	71.75%	1	1	59	194							[illegible]
IPI00133562.1	Coiled-coil-helix-coiled-coil-helix domain-containing protein 3, mitochondria	86.78%	4	1	34	311	-0.193	0.054	-0.042	0.027	-0.080	14	[illegible]
IPI00274747.6	Cold shock domain-containing protein E1	78.32%	3	28	96	598	-0.026	0.003	-0.057	0.036	-0.047	54	[illegible]
IPI00121073.1	Cold-inducible RNA-binding protein	93.60%	3	6	26	86							[illegible]
IPI00129571.3	Collagen alpha-1(III) chain	60.59%	2	14	79	232							[illegible]
IPI00128689.2	Collagen alpha-1(V) chain	68.88%	2	11	108	457							[illegible]
IPI00339885.2	Collagen alpha-1(VI) chain	64.20%	3	11	64	232	-0.063	0.134	-0.136	-0.139	0.295	3	[illegible]
IPI00126387.3	Collagen alpha-1(XIX) chain	42.34%	2	1	40	99	0.348	0.220	-0.119	-0.060	0.083	3	[illegible]
IPI00222188.4	Collagen alpha-2(I) chain	91.25%	3	33	107	447							[illegible]
IPI00121120.3	Collagen alpha-2(V) chain	68.20%	2	12	91	273							[illegible]
IPI00621027.2	Collagen alpha-2(VI) chain	63.64%	2	4	77	186							[illegible]
IPI00113477.5	collagen, type IV, alpha 5	54.11%	4	2	57	128							[illegible]
IPI00227640.1	COMM domain-containing protein 3	85.13%	1	3	13	32	0.344	-0.009	0.446	0.100	0.089	3	[illegible]
IPI00132799.4	complement component 1 Q subcomponent-binding protein, mitochondria	90.68%	2	10	26	362	0.019	-0.014	-0.201	0.094	-0.103	26	[illegible]
IPI00132278.1	Complexin-1	94.03%	2	5	20	144							[illegible]
IPI00111501.1	Complexin-2	75.37%	1	4	15	104							[illegible]
IPI01008455.1	Condensin complex subunit 1	85.83%	1	2	132	592	-0.063	0.021	-0.116	-0.050	-0.186	18	[illegible]
IPI00224053.3	Condensin complex subunit 2	66.62%	2	15	56	220	-0.012	-0.091	-0.020	-0.020	-0.175	14	[illegible]
IPI00830478.1	Constitutive coactivator of PPAR-gamma-like protein 1	75.54%	1	20	98	416	0.132	-0.019	0.072	-0.037	0.246	20	[illegible]
IPI00123058.1	Contactin-1	74.41%	1	32	80	474							[illegible]
IPI00119970.4	Contactin-2	53.94%	1	3	62	150							[illegible]
IPI00338983.4	Contactin-associated protein 1	48.45%	1	19	61	181							[illegible]
IPI01008124.1	contactin-associated protein-like 2 isoform a	71.62%	2	11	87	285							[illegible]
IPI00480341.4	COP9 signalosome complex subunit 1 isoform 1	76.62%	3	13	61	313	-0.100	-0.019	-0.039	-0.016	-0.042	15	[illegible]
IPI00131870.3	COP9 signalosome complex subunit 3	100.00%	3	7	45	430	-0.051	0.037	0.037	0.073	0.027	14	[illegible]
IPI00131871.1	COP9 signalosome complex subunit 4	99.01%	4	20	59	406	-0.141	0.068	0.041	0.064	-0.080	22	[illegible]
IPI00135087.3	COP9 signalosome complex subunit 5	88.92%	1	10	31	249	-0.250	-0.142	-0.152	-0.041	-0.139	19	[illegible]
IPI00453769.2	COP9 signalosome complex subunit 7b	62.12%	1	3	21	112							[illegible]
IPI00187407.4	COP9 signalosome complex subunit 8	84.21%	1	6	16	159	0.040	0.108	-0.142	0.058	0.052	14	[illegible]
IPI00224075.1	Copine-1	65.67%	9	8	43	127	-0.293	-0.152	-0.199	-0.035	-0.181	5	[illegible]
IPI00170387.1	Copine-5	52.45%	7	3	31	82							[illegible]
IPI00129451.1	Copine-6	74.33%	2	10	44	159							[illegible]
IPI00117379.3	Copper transport protein ATOX1	100.00%	1	4	10	58							[illegible]
IPI00400301.2	Coproporphyrinogen-III oxidase, mitochondria	81.26%	1	13	46	192	-0.180	-0.051	-0.295	0.092	-0.162	5	[illegible]
IPI00955082.1	core histone macro-H2A.1 isoform 2	89.22%	4	12	38	325	0.249	-0.009	0.256	0.077	0.107	27	[illegible]
IPI00652934.3	Core histone macro-H2A.2	78.23%	3	6	44	205	0.102	-0.071	0.103	0.080	0.265	12	[illegible]
IPI00323600.4	Coronin-1A	64.21%	3	12	39	127	0.114	-0.085	0.234	-0.037	0.214	4	[illegible]
IPI00124819.1	Coronin-1B	73.14%	2	14	42	291	0.061	-0.158	0.148	-0.102	0.165	31	[illegible]
IPI00124820.2	Coronin-1C	79.11%	4	19	50	507	0.183	0.111	0.176	0.057	0.152	34	[illegible]
IPI00115949.1	Cox7a2l protein	96.46%	3	5	12	99							[illegible]
IPI00453599.2	Craniofacial development protein 1	69.83%	1	7	28	180	0.190	0.027	0.197	0.089	0.187	16	[illegible]
IPI00136703.1	Creatine kinase B-type	81.89%	1	21	40	557							[illegible]
IPI00127596.1	Creatine kinase M-type	54.86%	1	1	27	69							[illegible]
IPI00128296.1	Creatine kinase U-type, mitochondrial	88.52%	6	17	50	314							[illegible]
IPI00463549.3	CREB-binding protein	53.01%	2	2	117	294							[illegible]
IPI00113362.2	Crk-like protein	60.40%	1	9	23	183	-0.068	-0.041	0.189	-0.004	-0.062	8	[illegible]
IPI00132376.3	Crooked neck-like protein 1	67.25%	3	9	64	145							[illegible]
IPI00986703.1	C-terminal-binding protein 1 isoform 1	41.14%	5	4	32	171	0.091	-0.019	0.183	-0.013	0.132	5	[illegible]
IPI00111959.2	CTP synthase 1	81.05%	1	20	63	397	-0.111	-0.047	-0.028	0.013	-0.062	33	[illegible]
IPI00124047.1	Cullin-1	89.56%	4	23	100	462	0.013	-0.028	-0.146	-0.022	-0.099	12	[illegible]
IPI00467383.2	Cullin-3	92.45%	9	16	110	462	-0.127	-0.059	-0.074	-0.080	-0.007	17	[illegible]
IPI00321407.6	Cullin-4A	71.54%	3	3	71	198	-0.163	0.048	-0.065	0.050	-0.150	9	[illegible]
IPI00988369.1	Cullin-5	74.23%	2	1	90	244	-0.085	-0.009	0.024	0.092	0.011	4	[illegible]
IPI00331507.9	cullin-5 isoform 1	75.09%	2	1	102	287	-0.085	-0.009	0.024	0.092	0.011	4	[illegible]
IPI00896727.1	Cullin-associated NEDD8-dissociated protein 1	92.52%	2	52	139	1260	-0.144	-0.053	-0.100	-0.014	-0.130	49	[illegible]
IPI00420577.3	Cullin-associated NEDD8-dissociated protein 2	60.16%	1	2	90	300							[illegible]
IPI00947597.1	cutaneous T-cell lymphoma-associated antigen 5 homolog isoform 2	79.27%	4	6	89	301	0.016	0.044	0.029	-0.038	-0.136	3	[illegible]
IPI00114491.1	Cyclin-dependent kinase 1	90.24%	45	14	34	337	-0.191	-0.111	-0.097	0.052	-0.157	32	[illegible]
IPI00111168.1	Cyclin-dependent kinase 18	51.22%	45	6	29	84	-0.034	0.051	0.112	0.010	0.141	3	[illegible]
IPI00128326.1	Cyclin-dependent kinase 4	72.94%	47	4	21	73	-0.340	-0.100	-0.642	-0.196	-0.432	6	[illegible]
IPI00309262.3	Cyclin-dependent kinase 5	70.55%	42	6	20	87							[illegible]
IPI00128463.1	Cyclin-dependent kinase 6	67.79%	42	4	26	93	-0.238	-0.062	-0.171	-0.167	-0.106	6	[illegible]
IPI00113698.1	Cyclin-dependent kinases regulatory subunit 1	94.94%	4	2	9	24							[illegible]
IPI00660051.1	cyclin-dependent kinases regulatory subunit 2-like	41.77%	5	2	4	15							[illegible]
IPI00122344.1	Cystathionine gamma-lyase	95.48%	1	12	47	268							[illegible]
IPI00125931.1	Cystatin-B	92.86%	1	3	13	285	-0.181	-0.198	0.023	0.024	0.043	16	[illegible]
IPI00123744.1	Cystatin-C	71.43%	2	4	14	67							[illegible]
**IPI00123891.3**	**Cysteine and glycine-rich protein 1**	**91.71%**	**1**	**8**	**27**	**216**	**0.154**	**-0.063**	**0.219**	**0.011**	**0.192**	**11**	[illegible]
IPI00470178.7	Cysteine and glycine-rich protein 2	78.24%	2	4	13	56	0.834	-0.066	0.815	0.065	1.014	3	[illegible]
IPI00134017.1	Cysteine and histidine-rich domain-containing protein 1	88.22%	1	8	35	141	-0.218	-0.131	-0.221	-0.069	-0.173	12	[illegible]
IPI00311072.2	cysteine desulfurase, mitochondrial	83.01%	4	7	45	122							[illegible]
IPI00129015.1	Cysteine-rich with EGF-like domain protein 1	80.48%	1	3	26	72							[illegible]
IPI00111286.1	Cysteine-rich with EGF-like domain protein 2	64.29%	1	3	22	88	0.469	-0.007	0.402	-0.088	0.222	5	[illegible]
IPI00230113.5	Cytochrome b5	91.79%	3	6	14	119	0.569	0.044	0.632	0.094	0.430	19	[illegible]
IPI00315794.1	Cytochrome b5 type B	95.89%	2	7	13	230	0.154	0.081	0.107	0.079	0.098	17	[illegible]
IPI00111885.2	Cytochrome b-c1 complex subunit 1, mitochondrial	72.50%	2	19	49	872	-0.079	-0.001	-0.076	-0.056	-0.076	22	[illegible]
IPI00119138.1	Cytochrome b-c1 complex subunit 2, mitochondrial	79.25%	1	25	48	757	-0.054	0.066	-0.111	0.033	-0.108	26	[illegible]
IPI00129516.1	Cytochrome b-c1 complex subunit 6, mitochondrial	92.13%	1	3	11	55							[illegible]
IPI00224210.5	Cytochrome b-c1 complex subunit 8	100.00%	2	6	13	104							[illegible]
IPI00153381.1	Cytochrome b-c1 complex subunit 9	53.13%	2	3	6	42							[illegible]
IPI00133240.1	Cytochrome b-c1 complex subunit Rieske, mitochondrial	76.64%	1	11	22	173	-0.031	0.055	-0.031	0.045	0.000	8	[illegible]
IPI00130376.1	Cytochrome c oxidase subunit 1	9.73%	1	1	3	5							[illegible]
IPI00131176.1	Cytochrome c oxidase subunit 2	78.41%	1	4	11	236	0.195	0.023	0.124	0.119	0.183	19	[illegible]
IPI00117978.1	Cytochrome c oxidase subunit 4 isoform 1, mitochondrial	92.31%	2	11	22	325	0.192	0.069	0.052	0.100	0.095	22	[illegible]
IPI00120719.4	Cytochrome c oxidase subunit 5A, mitochondrial	89.04%	1	9	21	214	0.228	-0.092	0.193	-0.009	0.309	10	[illegible]
IPI00785410.1	Cytochrome c oxidase subunit 5B, mitochondrial	80.47%	3	8	20	231	0.198	0.085	0.147	0.084	0.149	12	[illegible]
IPI00225390.5	Cytochrome c oxidase subunit 6B1	86.05%	1	6	12	149							[illegible]
IPI00131771.3	Cytochrome c oxidase subunit 6C	98.68%	2	8	14	124	0.080	0.052	0.103	-0.023	0.100	6	[illegible]
IPI00114377.1	Cytochrome c oxidase subunit 7A2, mitochondrial	84.34%	1	3	11	134	0.167	0.276	0.308	0.178	0.034	14	[illegible]
IPI00111255.1	Cytochrome c oxidase subunit 7C, mitochondrial	53.97%	4	2	10	74	0.099	0.228	0.185	0.033	0.059	7	[illegible]
IPI00309285.5	cytochrome c-type heme lyase	69.85%	3	6	24	137							[illegible]
IPI00222582.2	Cytochrome P450 20A1	73.38%	2	10	41	163	-0.216	0.089	-0.105	0.092	-0.309	4	[illegible]
IPI00123619.7	cytochrome P450, family 2, subfamily d, polypeptide 22	55.00%	5	4	26	95							[illegible]
IPI00875325.1	Cytoplasmic aconitate hydratase	71.32%	2	11	66	190	0.076	-0.155	0.052	-0.023	0.115	12	[illegible]
IPI00119876.2	Cytoplasmic dynein 1 heavy chain 1	86.93%	4	221	544	4982	0.095	-0.035	0.115	-0.032	0.158	247	[illegible]
IPI00153421.1	Cytoplasmic dynein 1 light intermediate chain 1	89.29%	2	18	58	343	0.169	-0.003	-0.028	0.006	0.149	25	[illegible]
IPI00420806.4	Cytoplasmic dynein 1 light intermediate chain 2	71.95%	1	10	45	331	0.070	-0.006	0.122	-0.060	0.259	25	[illegible]
IPI00405625.9	Cytoplasmic FMR1-interacting protein 2	80.85%	2	14	135	529							[illegible]
IPI00470092.2	Cytoskeleton-associated protein 2	76.81%	1	5	83	261							[illegible]
IPI00223047.2	Cytoskeleton-associated protein 4	91.65%	6	37	79	1007	-0.243	0.030	-0.264	0.021	-0.274	50	[illegible]
IPI00649681.2	cytosolic 5'-nucleotidase III-like protein isoform 1	84.00%	4	3	29	112							[illegible]
IPI00125939.3	cytosolic acyl coenzyme A thioester hydrolase isoform 1	79.17%	4	17	48	463	0.116	-0.032	0.223	-0.047	0.317	33	[illegible]
IPI00126924.1	Cytosolic Fe-S cluster assembly factor NUBP1	52.50%	1	1	14	42	-0.208	-0.079	-0.128	-0.163	-0.175	5	[illegible]
IPI00126925.1	Cytosolic Fe-S cluster assembly factor NUBP2	36.36%	1	2	11	21							[illegible]
IPI00315879.1	Cytosolic non-specific dipeptidase	82.32%	2	21	46	319	0.038	-0.152	0.027	-0.080	0.195	28	[illegible]
IPI00111169.1	Cytosolic phospholipase A2	63.37%	2	10	54	149							[illegible]
IPI00944120.1	cytosolic purine 5'-nucleotidase isoform 2	80.68%	3	13	50	154	-0.032	-0.175	0.013	-0.063	-0.035	16	[illegible]
IPI00222675.3	D19Bwg1357e protein	79.32%	3	15	81	352	-0.144	0.061	0.037	0.000	-0.117	19	[illegible]
IPI00225961.5	D-3-phosphoglycerate dehydrogenase	66.23%	3	25	52	787	-0.236	-0.045	-0.163	0.007	-0.120	32	[illegible]
IPI00330754.2	D-beta-hydroxybutyrate dehydrogenase, mitochondrial	76.68%	4	9	32	146							[illegible]
IPI00170084.1	DCC-interacting protein 13-alpha	72.28%	2	7	52	180							[illegible]
IPI00752092.3	DCN1-like protein 1	60.23%	6	3	18	74	0.026	-0.063	0.126	0.163	0.087	4	[illegible]
IPI00135700.1	dCTP pyrophosphatase 1	57.06%	1	2	10	29							[illegible]
IPI00129701.7	DDB1- and CUL4-associated factor 13	67.19%	1	1	39	66	-0.362	-0.083	-0.128	-0.059	-0.216	4	[illegible]
IPI00230034.5	D-dopachrome decarboxylase	99.15%	3	7	13	63							[illegible]

IPI00330679.2	DDRGK domain-containing protein 1	67.30%	1	3	27	84	-0.014	0.058	0.144	0.009	0.112	3
IPI00128975.3	Death-associated protein 1	95.10%	1	5	13	83	0.120	-0.103	-0.094	-0.056	0.126	15
IPI00117846.1	Death-associated protein kinase 3	51.12%	1	3	36	69	0.165	0.055	0.187	0.034	0.158	3
IPI00331549.1	Dehydrogenase/reductase SDR family member 1	79.55%	1	7	30	178	0.167	0.055	0.194	0.178	0.293	3
IPI00110659.2	Dehydrogenase/reductase SDR family member 7	79.88%	1	6	31	129	0.112	0.195	0.051	-0.069	0.075	4
IPI00130804.1	Delta(3,5)-Delta(2,4)-dienoyl-CoA isomerase, mitochondrial	92.35%	2	6	38	121						
IPI00112719.1	Delta-aminolevulinic acid dehydratase	70.00%	1	9	26	251	-0.199	-0.170	-0.100	-0.095	0.052	27
IPI00125328.3	Dematin	63.21%	7	7	32	137						
IPI00330057.4	Density-regulated protein	92.93%	2	6	21	74	0.106	0.023	0.300	0.047	-0.020	6
IPI00114647.10	Deoxyhypusine synthase	87.26%	2	6	31	101						
IPI00153607.1	Deoxynucleotidyltransferase terminal-interacting protein 2	80.08%	1	10	83	372	0.420	0.218	0.490	0.394	0.249	7
IPI00187434.1	deoxyuridine triphosphatase isoform 1	92.50%	2	10	27	184	-0.111	-0.171	-0.091	0.041	-0.102	23
IPI00886236.1	Dephospho-CoA kinase domain-containing protein	90.91%	3	8	28	117	-0.494	-0.032	-0.419	0.013	-0.624	5
IPI00130102.4	Desmin	96.38%	4	29	81	1003	-0.019	-0.257	-0.086	-0.150	0.059	20
IPI00553419.3	Desmoplakin	85.15%	2	32	400	1577	0.352	-0.028	0.257	0.017	0.325	44
IPI00127942.4	Destrin	93.94%	1	14	26	366	-0.041	-0.131	-0.045	-0.057	0.021	29
IPI00263313.1	Developmentally-regulated GTP-binding protein 1	73.02%	2	11	31	185	-0.165	0.027	-0.167	-0.016	-0.141	18
IPI00134820.1	Developmentally-regulated GTP-binding protein 2	95.60%	1	6	45	122	-0.160	-0.128	-0.329	0.138	-0.253	4
IPI00894963.1	Diacylglycerol kinase epsilon	51.06%	4	3	28	67						
IPI00330317.2	Dickkopf-related protein 1	90.81%	2	1	19	60						
IPI00230042.5	Dihydrofolate reductase	87.70%	1	6	24	117	-0.019	-0.165	0.187	0.061	0.080	8
IPI00874456.1	Dihydrolipoyl dehydrogenase, mitochondria	76.23%	2	19	46	704	0.017	0.077	-0.006	0.024	-0.004	28
IPI00153660.4	Dihydrolipoyllysine-residue acetyltransferase component of pyruvate dehyc	76.01%	1	19	60	461	-0.100	-0.038	-0.234	-0.032	-0.180	23
IPI00459279.2	Dihydropteridine reductase	100.00%	4	10	36	321	0.087	0.050	0.134	-0.050	0.154	10
IPI00875117.1	Dihydropyrimidinase-related protein 1	83.74%	1	1	54	783	0.301	-0.091	0.317	-0.111	0.355	16
IPI00114375.2	Dihydropyrimidinase-related protein 2	93.53%	1	28	66	1655	0.209	-0.105	0.256	-0.060	0.292	93
IPI00624192.3	Dihydropyrimidinase-related protein 5	91.31%	2	12	56	252						
IPI00880742.1	dipeptidyl aminopeptidase-like protein 6 isoform 3	67.17%	4	10	60	203						
IPI00116134.2	Dipeptidyl peptidase 3	85.64%	2	23	68	362	0.169	-0.083	0.225	-0.094	0.208	39
IPI00119880.1	Diphosphoinositol polyphosphate phosphohydrolase 1	80.36%	2	3	9	21						
IPI00854941.1	Diphosphoinositol polyphosphate phosphohydrolase 2	28.49%	1	2	4	18	-0.260	-0.128	-0.407	-0.295	-0.315	4
IPI00319950.4	Diphosphomevalonate decarboxylase	71.82%	1	4	38	225						
IPI00124761.1	Discoidin domain-containing receptor 2	40.52%	1	6	43	150						
IPI00131881.2	Disintegrin and metalloproteinase domain-containing protein 1C	67.02%	3	6	53	105						
IPI00626485.2	Disintegrin and metalloproteinase domain-containing protein 9	60.47%	2	5	51	170						
IPI00136350.3	Disks large homolog 3	61.01%	5	9	53	229						
IPI00649420.2	disks large-associated protein 5 isoform 1	83.79%	5	6	96	311	-0.392	-0.057	-0.204	-0.128	-0.344	5
IPI00469323.3	DNA (cytosine-5)-methyltransferase 1 isoform 2	77.58%	4	42	163	755	-0.071	0.031	-0.105	-0.038	-0.094	37
IPI00316740.4	DNA damage-binding protein 1	77.98%	3	39	93	697	-0.025	-0.045	-0.054	0.043	-0.034	51
IPI00473314.3	DNA ligase 1	78.00%	4	23	93	423	-0.051	-0.053	-0.012	-0.025	0.003	16
IPI00118158.1	DNA mismatch repair protein Msh2	68.77%	2	16	80	499	-0.094	-0.097	-0.083	0.036	-0.045	11
IPI00323790.2	DNA mismatch repair protein Msh6	75.41%	1	22	121	503	-0.180	-0.014	-0.191	-0.106	-0.253	18
IPI00114051.1	DNA polymerase alpha catalytic subunit	75.84%	2	12	123	374	-0.500	-0.243	-0.436	-0.402	-0.651	5
IPI00170123.3	DNA polymerase beta	71.64%	1	5	35	114						
IPI00323143.2	DNA polymerase delta catalytic subunit	57.29%	2	3	77	257	-0.269	-0.141	-0.094	0.052	-0.262	3
IPI00114069.1	DNA primase large subunit	75.84%	2	5	51	145	-0.099	-0.292	-0.278	0.023	-0.065	3
IPI00322337.5	DNA primase small subunit	65.47%	1	3	39	70						
IPI00551367.2	DNA repair protein RAD50	87.04%	4	20	201	712	-0.054	-0.105	-0.056	-0.048	-0.139	8
IPI00118139.2	DNA repair protein XRCC1	47.54%	1	2	29	49						
IPI00108338.1	DNA replication licensing factor MCM3	92.73%	1	43	111	1248	-0.003	0.023	0.004	0.009	-0.044	94
IPI00117016.1	DNA replication licensing factor MCM4	87.35%	2	30	102	682	-0.128	-0.097	-0.144	-0.037	-0.045	36
IPI00309398.6	DNA replication licensing factor MCM5	90.19%	2	31	111	619	-0.062	-0.026	0.034	0.000	0.017	21
IPI00123881.1	DNA replication licensing factor MCM6	91.11%	4	38	115	718	-0.100	-0.092	-0.074	0.031	-0.163	37
IPI00109764.2	DNA topoisomerase 1	82.27%	2	28	109	492	-0.102	0.076	-0.108	0.001	-0.136	29
IPI00122223.1	DNA topoisomerase 2-alpha	83.51%	2	56	192	1656	-0.535	0.055	-0.517	0.021	-0.527	95
IPI00135443.2	DNA topoisomerase 2-beta	80.40%	8	21	205	962	-0.346	0.037	-0.517	-0.009	-0.123	25
IPI00313726.2	DNA-directed RNA polymerase I subunit RPA1	75.60%	3	5	150	594						
IPI00169700.4	DNA-directed RNA polymerase I subunit RPA34	69.67%	1	3	27	133						
IPI00136207.6	DNA-directed RNA polymerase II subunit RPB1	71.02%	2	22	173	673	0.180	0.184	0.166	0.130	0.436	12
IPI00320034.6	DNA-directed RNA polymerase II subunit RPB2	76.32%	1	15	112	392	-0.262	-0.269	-0.180	-0.201	-0.196	17
IPI00875500.1	DNA-directed RNA polymerase II subunit RPB3	77.82%	3	6	25	142	0.120	0.070	0.138	0.184	0.080	4
IPI00268215.1	DNA-directed RNA polymerase II subunit RPB4	80.99%	2	2	12	38						
IPI00337955.1	DNA-directed RNA polymerases I, II, and III subunit RPABC1	70.00%	2	3	20	116	-0.045	-0.041	-0.165	-0.068	0.026	3
IPI00124284.3	DNA-directed RNA polymerases I, II, and III subunit RPABC3	88.00%	1	5	12	47	-0.176	0.038	-0.193	-0.077	-0.224	4
IPI00458074.4	DnaJ (Hsp40) homolog, subfamily C, member 13	75.66%	5	12	174	582	-0.267	-0.089	0.100	0.231	0.104	3
IPI00132208.1	DnaJ homolog subfamily A member 1	92.44%	6	19	47	560	0.153	-0.054	0.144	-0.035	0.167	41
IPI00136251.1	DnaJ homolog subfamily A member 2	91.02%	1	18	53	535	-0.042	-0.042	0.048	-0.006	-0.053	28
IPI00125454.1	DnaJ homolog subfamily A member 4	68.26%	2	2	41	122						
IPI00136253.3	DnaJ homolog subfamily B member 1	71.76%	2	13	40	258						
IPI00320241.1	DnaJ homolog subfamily B member 11	79.89%	1	13	36	307	-0.025	0.001	0.041	-0.028	-0.016	17
IPI00223482.2	dnaJ homolog subfamily B member 2 isoform 3	43.52%	3	1	17	46						
IPI00110760.1	DnaJ homolog subfamily B member 4	77.15%	1	11	34	157						
IPI00844664.2	DnaJ homolog subfamily C member 10	60.53%	2	13	60	293	-0.103	-0.072	-0.186	-0.023	-0.154	20
IPI00336247.4	DnaJ homolog subfamily C member 16	68.52%	1	3	62	237						
IPI00126317.1	DnaJ homolog subfamily C member 2	75.36%	5	13	70	270	-0.034	-0.004	0.017	-0.117	0.009	9
IPI00459033.2	DnaJ homolog subfamily C member 3	55.36%	1	11	33	133	0.050	-0.041	0.068	-0.026	0.099	9
IPI00331385.2	DnaJ homolog subfamily C member 7	80.77%	3	18	59	354	-0.278	-0.089	-0.338	-0.059	-0.257	21
IPI00463344.6	DnaJ homolog subfamily C member 8	81.42%	6	11	27	157	0.094	0.034	0.160	0.084	0.130	10
IPI00128268.2	DnaJ homolog subfamily C member 9	77.61%	2	7	29	128						
IPI00117705.2	Dolichyl-diphosphooligosaccharide--protein glycosyltransferase 48 kDa subu	84.58%	3	13	30	250	0.061	0.037	-0.117	-0.054	-0.074	19
IPI00309035.2	Dolichyl-diphosphooligosaccharide--protein glycosyltransferase subunit 1	85.03%	1	31	69	636	0.023	0.047	0.050	0.017	-0.037	38
IPI00316469.4	Dolichyl-diphosphooligosaccharide--protein glycosyltransferase subunit STT	57.47%	1	7	41	138	0.421	0.158	0.352	0.079	0.212	7
IPI00329840.3	Double-strand-break repair protein rad21 homolog	68.19%	1	13	50	277	0.091	0.006	-0.071	-0.163	0.110	11
IPI00749596.1	double-stranded RNA-binding protein Staufen homolog 1 isoform 1	88.48%	5	10	52	252	-0.097	-0.086	-0.385	-0.198	-0.295	3
IPI00466610.5	Dual specificity mitogen-activated protein kinase kinase 1	70.99%	1	8	32	343	-0.006	-0.065	-0.105	-0.051	0.026	19
IPI00112335.1	Dual specificity mitogen-activated protein kinase kinase 4	83.12%	2	7	37	129	0.156	-0.041	0.506	0.393	0.448	3
IPI00137439.1	Dual specificity mitogen-activated protein kinase kinase 6	78.14%	2	1	32	91	-0.095	-0.099	-0.071	-0.123	-0.240	3
IPI00380332.4	Dual specificity protein phosphatase	74.34%	5	7	35	153						
IPI00110990.1	Dual specificity protein phosphatase 3	68.11%	2	3	12	119	-0.044	-0.041	-0.092	-0.297	0.034	13
IPI00856500.2	dynactin subunit 1 isoform 3	84.67%	10	43	145	940	0.203	0.030	0.204	-0.082	0.091	34
IPI00116112.3	Dynactin subunit 2	93.03%	2	1	50	620	0.233	0.020	0.160	-0.044	0.163	31
IPI00988443.1	dynactin subunit 2 isoform 2	93.07%	2	1	50	616	0.231	0.024	0.180	0.009	0.162	30
IPI00331339.3	Dynactin subunit 3	68.28%	2	6	20	61						
IPI01008216.1	dynein heavy chain 12, axonemal	68.43%	4	5	324	1106						
IPI00128278.2	Dynein heavy chain 5, axonemal	78.40%	1	5	445	1604						
IPI00121623.1	Dynein light chain 1, cytoplasmic	92.13%	1	2	9	141	-0.226	0.029	-0.120	0.052	-0.383	4
IPI00132734.1	Dynein light chain 2, cytoplasmic	87.64%	1	1	11	59	-0.396	0.209	-0.281	0.058	-0.550	3
IPI00315225.7	Dynein light chain roadblock-type 1	95.83%	2	4	15	133	0.040	-0.131	0.179	-0.057	0.144	10
IPI00127431.1	Dynein light chain Tctex-type 1	100.00%	3	2	10	72	0.312	-0.069	0.434	0.001	0.383	3
IPI00622122.5	dynein, axonemal, heavy chain 11	68.98%	1	4	347	1110						
IPI00762553.2	dystonin isoform b	78.52%	13	22	761	3056	-0.292	-0.069	-0.158	0.001	-0.211	30
IPI00122273.1	Dystroglycan	53.86%	1	4	46	118	-0.080	-0.034	0.179	-0.012	-0.038	3
IPI00474450.2	Dystrophin	70.88%	5	5	337	1092						
IPI00337844.5	E3 SUMO-protein ligase RanBP2	79.36%	1	68	304	1621	-0.091	0.040	-0.041	-0.031	-0.106	50
IPI00122289.4	E3 ubiquitin/ISG15 ligase TRIM25	84.54%	5	1	62	199	0.091	0.006	0.217	0.019	0.175	10
IPI00130236.4	E3 ubiquitin-protein ligase ARIH1	65.23%	2	6	46	186	-0.180	-0.246	-0.281	-0.144	-0.267	4
IPI00130237.1	E3 ubiquitin-protein ligase ARIH2	51.63%	1	2	28	118	-0.170	-0.082	-0.444	-0.260	-0.304	5
IPI00621791.3	E3 ubiquitin-protein ligase BRE1B	83.32%	1	6	124	417						
IPI00762434.1	E3 ubiquitin-protein ligase HECTD1	62.87%	2	5	182	370	0.048	0.120	-0.241	-0.014	-0.112	4
IPI00463909.3	E3 ubiquitin-protein ligase HUWE1	74.55%	6	4	371	1641	-0.288	0.023	-0.176	-0.100	-0.340	23
IPI00347336.7	E3 ubiquitin-protein ligase listerin	63.95%	2	5	113	313	0.013	0.165	-0.224	0.229	-0.038	3
IPI00462445.2	E3 ubiquitin-protein ligase NEDD4	73.39%	3	30	70	637	-0.117	-0.076	-0.048	-0.086	-0.041	68
IPI00133880.2	E3 ubiquitin-protein ligase RING2	79.46%	4	6	37	121	-0.019	0.115	-0.119	-0.034	-0.103	4
IPI00321005.5	E3 ubiquitin-protein ligase TRIM32	48.85%	3	2	34	107						
IPI01008056.1	E3 ubiquitin-protein ligase TRIM33 isoform 1	66.14%	7	7	84	222						
IPI00831567.2	E3 ubiquitin-protein ligase UBR5 isoform 1	75.73%	2	9	261	931	0.075	-0.072	0.138	0.026	0.073	6
IPI00753418.2	E3 ubiquitin-protein ligase UHRF1 isoform B	81.27%	3	15	84	408	-0.117	0.147	-0.265	0.058	-0.326	10
IPI00453776.1	Early endosome antigen 1	87.17%	2	33	225	1345	0.127	-0.042	0.217	-0.020	0.059	25
IPI01027439.1	echinoderm microtubule-associated protein-like 4 1	68.12%	5	4	78	236						
IPI00331234.4	Ectoderm-neural cortex protein 1	74.02%	1	3	43	96						
IPI00308077.6	Ectonucleotide pyrophosphatase/phosphodiesterase family member 6	78.18%	2	4	35	72						
IPI00453906.2	EF-hand domain-containing family member A1	56.71%	1	4	32	97						
IPI00659608.4	EF-hand domain-containing family member A2	36.14%	3	2	23	44						
IPI00137288.1	EF-hand domain-containing protein D1	67.50%	1	3	25	77						
IPI00896707.1	EG331392 protein	76.62%	2	1	58	211	-0.141	0.204	-0.085	-0.086	-0.162	8
IPI00223457.1	EGF-containing fibulin-like extracellular matrix protein 1	71.60%	1	2	36	135						
IPI00118018.5	EH domain-binding protein 1-like protein 1 isoform a	70.55%	8	9	140	420						
IPI00126083.3	EH domain-containing protein 1	88.76%	2	4	66	597	0.149	0.016	0.145	-0.004	0.169	41
IPI00402968.1	EH domain-containing protein 2	90.42%	2	23	57	402	-0.032	-0.083	-0.009	-0.128	-0.007	29
IPI00108271.2	ELAV-like protein 1	78.83%	1	12	41	552	0.017	0.026	0.027	0.023	-0.066	27
IPI00121676.1	ELAV-like protein 2	79.72%	8	4	45	321						
IPI00857092.1	electrogenic sodium bicarbonate cotransporter 1 isoform b	55.48%	3	1	52	180						
IPI00116753.4	Electron transfer flavoprotein subunit alpha, mitochondrial	96.70%	1	18	39	650	-0.057	0.062	-0.080	0.036	-0.042	58
IPI00121440.4	Electron transfer flavoprotein subunit beta	93.33%	1	17	36	384	-0.050	0.038	-0.038	0.057	-0.047	20
IPI00121322.2	Electron transfer flavoprotein-ubiquinone oxidoreductase, mitochondria	73.86%	2	13	57	248	-0.006	0.124	-0.017	-0.039	-0.102	3
IPI01027796.1	ELKS/Rab6-interacting/CAST family member 1 isoform 1	87.95%	6	14	170	724						
IPI00307837.6	Elongation factor 1-alpha 1	92.21%	5	19	54	2704	-0.042	-0.080	-0.173	-0.044	-0.071	158
IPI00119667.1	Elongation factor 1-alpha 2	96.76%	1	14	57	977	-0.069	-0.038	-0.171	-0.047	-0.136	59
IPI00320208.3	Elongation factor 1-beta	96.44%	3	17	28	543	0.055	0.072	0.108	0.033	-0.042	45
IPI00318841.4	Elongation factor 1-gamma	94.74%	1	33	50	893	0.013	0.011	0.040	0.051	-0.056	84
IPI00466069.3	Elongation factor 2	91.84%	3	59	121	2282	-0.146	-0.109	-0.050	-0.001	-0.065	178
IPI00230283.5	Elongation factor G, mitochondrial	87.75%	2	17	93	415	-0.257	-0.035	-0.362	0.024	-0.281	8
IPI00113052.1	Elongation factor Ts, mitochondrial	90.43%	3	11	29	220	0.070	0.185	0.043	0.185	0.059	4
IPI00453532.2	Elongation factor Tu GTP-binding domain-containing protein 1	70.54%	1	4	110	339	0.223	0.220	0.003	0.052	0.157	5
IPI00124382.1	Elongation of very long chain fatty acids protein 1	60.22%	2	2	15	127						
IPI00380780.7	Elongator complex protein 1	58.44%	1	6	86	204						
IPI00308066.1	Elongator complex protein 4	85.31%	2	2	51	169						
IPI00129968.2	Embigin	58.18%	1	7	32	119						
IPI00114401.1	Emerin	81.08%	4	11	19	254	-0.044	0.120	-0.014	0.059	-0.062	18
IPI00312752.3	Endophilin-A2	61.41%	1	8	35	254	0.253	0.070	0.218	-0.041	0.141	11
IPI00312771.7	Endophilin-A3	74.06%	1	3	33	80						
IPI00318595.2	Endoplasmic reticulum aminopeptidase 1	71.94%	2	8	71	209						
IPI00124093.2	Endoplasmic reticulum mannosyl-oligosaccharide 1,2-alpha-mannosidase	54.10%	1	2	30	69						
IPI00118832.1	Endoplasmic reticulum resident protein 29	94.27%	1	9	31	228						
IPI00134058.3	Endoplasmic reticulum resident protein 44	83.00%	1	9	38	164	0.064	0.158	0.122	0.034	-0.092	8
IPI00321744.3	Endoplasmic reticulum-Golgi intermediate compartment protein 1	68.97%	2	7	17	119	-0.050	0.138	0.045	0.176	-0.039	6
IPI00129526.1	Endoplasmin	81.42%	2	10	97	2015	0.019	0.076	0.034	0.013	-0.059	126
IPI00471325.2	Endothelial differentiation-related factor 1	87.84%	2	8	25	153	0.009	0.011	0.024	0.007	-0.016	12
IPI00403589.1	Enhancer of rudimentary homolog	96.15%	3	6	16	288	0.079	0.000	0.166	0.095	-0.088	7
IPI00310578.3	Enhancer of yellow 2 transcription factor homolog	82.18%	1	1	14	47						
IPI00331692.1	enoyl-CoA delta isomerase 1, mitochondrial precursor	92.04%	2	11	40	300	-0.147	0.037	-0.252	-0.020	-0.184	17
IPI00454049.4	Enoyl-CoA hydratase, mitochondrial	78.97%	3	13	37	329	-0.236	-0.041	-0.059	-0.014	-0.123	14
IPI00989373.1	Env polyprotein	55.16%	9	2	34	140	-0.252	0.134	-0.533	0.128	-0.624	3
IPI00420148.2	Envelope polyprotein	84.03%	9	4	43	207	-0.116	0.054	-0.466	-0.022	-0.344	7
IPI00129220.2	Ephrin type-A receptor 2	71.95%	8	9	87	270	0.026	0.178	0.170	0.096	0.010	12
IPI00121190.1	Epidermal growth factor receptor	55.87%	7	4	67	143	0.318	0.052	0.240	-0.079	-0.069	3
IPI00129186.1	Epididymal secretory protein E1	83.22%	1	7	15	111	0.158	0.081	0.290	0.059	0.204	3
IPI00320459.2	Epiplakin	91.28%	1	9	281	1163	-0.019	0.027	0.184	0.000	0.064	24
IPI00124614.1	EPM2A-interacting protein 1	51.32%	1	3	43	111						
IPI00316509.2	Epoxide hydrolase 1	85.49%	4	21	46	289	-0.366	0.038	-0.413	0.010	-0.329	44
IPI00115317.1	ER lumen protein retaining receptor 1	46.23%	5	2	11	54	-0.181	-0.050	-0.165	0.013	-0.245	3
IPI00331524.7	erlin-1	82.47%	3	5	45	237	0.044	-0.103	0.174	0.252	0.068	7
IPI00221540.1	Erlin-2	85.88%	1	9	47	245	0.059	-0.103	0.119	0.151	0.172	11
IPI00624843.3	Ermin	66.55%	1	9	23	76						
IPI00754386.4	ERO1-like protein alpha	81.90%	1	12	41	221	-0.152	0.020	-0.272	-0.012	-0.184	16
IPI00323748.2	Erythrocyte band 7 integral membrane protein	84.51%	3	8	34	246	-0.116	-0.019	0.076	0.160	-0.229	13
IPI00133284.1	ES1 protein homolog, mitochondrial	91.35%	2	10	27	166	0.263	-0.023	0.274	0.016	0.195	6
IPI00169521.4	ESF1 homolog	48.64%	1	4	58	146						
IPI00118676.3	Eukaryotic initiation factor 4A-I	97.04%	2	15	51	1676	-0.074	-0.083	-0.095	0.026	-0.077	122
IPI00126716.3	Eukaryotic initiation factor 4A-III	98.54%	4	17	60	709	-0.206	-0.103	-0.116	0.024	-0.165	43
IPI00911165.1	eukaryotic peptide chain release factor GTP-binding subunit ERF3A isoform	65.51%	5	19	57	428	-0.211	-0.085	-0.111	-0.023	-0.134	33
IPI00312468.5	Eukaryotic peptide chain release factor subunit 1	90.16%	1	20	52	455	-0.221	-0.031	-0.176	-0.004	-0.139	30
IPI00133928.1	Eukaryotic translation elongation factor 1 epsilon-1	93.10%	1	7	24	163	0.036	0.094	0.069	-0.154	0.077	9
IPI00153608.3	Eukaryotic translation initiation factor 1A	76.39%	14	1	19	266	0.135	0.026	0.127	-0.010	0.096	24
IPI00987898.1	eukaryotic translation initiation factor 1A	76.39%	10	1	16	114	0.092	0.003	0.251	0.020	0.156	14
IPI00308984.6	Eukaryotic translation initiation factor 1A, X-chromosomal	86.81%	10	3	21	270	0.122	0.014	0.124	-0.020	0.089	25
IPI00318464.2	Eukaryotic translation initiation factor 1b	85.84%	8	6	11	110	-0.056	0.019	-0.017	0.033	-0.047	9
IPI00474446.4	Eukaryotic translation initiation factor 2 subunit 1	94.29%	1	18	46	573	-0.072	0.075	-0.051	0.066	-0.201	36
IPI00116302.3	Eukaryotic translation initiation factor 2 subunit 2	91.84%	3	19	43	487	-0.044	0.014	-0.083	0.014	-0.114	49
IPI00230415.5	Eukaryotic translation initiation factor 2 subunit 3, X-linked	77.12%	4	16	48	520	-0.069	-0.019	-0.103	-0.032	-0.117	49
IPI00407546.3	Eukaryotic translation initiation factor 2, subunit 3, structural gene Y-linked,	95.05%	1	2	13	137	-0.020	0.081	-0.004	-0.012	-0.041	12
IPI00648960.2	eukaryotic translation initiation factor 2B, subunit 3 gamma isoform 2	74.16%	3	4	39	155						
IPI00129276.3	Eukaryotic translation initiation factor 3 subunit A	80.65%	5	66	200	1806	0.031	0.021	0.013	0.003	-0.017	125
IPI01008667.1	Eukaryotic translation initiation factor 3 subunit B	87.42%	2	35	75	999	-0.086	0.006	0.024	-0.013	-0.051	64
IPI00321647.2	Eukaryotic translation initiation factor 3 subunit C	75.74%	2	27	100	806	0.003	0.009	-0.072	0.024	-0.039	88
IPI00762774.2	Eukaryotic translation initiation factor 3 subunit D	87.77%	1	22	70	684	-0.041	0.041	-0.065	-0.100	-0.112	33
IPI00132250.1	Eukaryotic translation initiation factor 3 subunit E	91.69%	1	21	55	626	-0.089	0.026	-0.128	0.001	-0.112	44
IPI00120914.2	Eukaryotic translation initiation factor 3 subunit F	55.40%	1	13	21	508	0.044	0.020	0.000	-0.009	0.024	19
IPI00622371.3	Eukaryotic translation initiation factor 3 subunit G	79.69%	1	18	39	365	-0.072	-0.028	-0.083	-0.026	-0.147	32
IPI00128202.1	Eukaryotic translation initiation factor 3 subunit H	84.94%	2	16	48	469	-0.092	0.016	-0.158	0.010	-0.130	43
IPI00269613.6	Eukaryotic translation initiation factor 3 subunit I	85.23%	3	15	31	370	0.010	0.087	-0.017	0.044	-0.010	27
IPI00471443.1	Eukaryotic translation initiation factor 3 subunit J	88.97%	2	14	34	339	-0.134	0.011	-0.138	-0.019	-0.186	27
IPI00463573.3	Eukaryotic translation initiation factor 3 subunit L	72.70%	1	23	56	561	-0.056	-0.009	-0.160	0.066	-0.142	42
IPI00115580.4	Eukaryotic translation initiation factor 3 subunit M	97.86%	3	16	47	269	-0.255	0.070	-0.181	0.052	-0.209	20
IPI00776162.3	eukaryotic translation initiation factor 4 gamma 2 isoform 1	78.48%	5	36	102	897	-0.136	-0.085	-0.123	-0.020	-0.038	47
IPI00221581.1	Eukaryotic translation initiation factor 4B	70.21%	2	27	72	799	-0.032	-0.007	0.021	0.001	-0.003	67
IPI00119057.1	Eukaryotic translation initiation factor 4E	80.65%	3	8	25	124	0.166	-0.047	-0.032	0.083	0.054	8
IPI00222983.1	Eukaryotic translation initiation factor 4E type 2	55.51%	7	1	15	22	-0.044	-0.149	0.072	-0.133	-0.072	4
IPI00318938.6	Eukaryotic translation initiation factor 4E-binding protein 1	94.87%	1	5	13	85						
IPI00187443.1	Eukaryotic translation initiation factor 5	78.09%	4	15	53	279	-0.162	0.001	-0.191	0.043	-0.194	31
IPI00108125.4	Eukaryotic translation initiation factor 5A-1	100.00%	3	8	21	478	-0.224	-0.077	-0.117	-0.116	-0.167	44
IPI00756424.3	Eukaryotic translation initiation factor 5B	75.90%	1	35	117	783	-0.020	0.037	-0.111	-0.032	-0.054	46
IPI00115862.1	Eukaryotic translation initiation factor 6	100.00%	3	6	20	184	-0.032	0.034	-0.057	-0.045	-0.017	7
IPI00114279.1	Excitatory amino acid transporter 1	61.14%	2	16	38	300						
IPI00131666.1	Excitatory amino acid transporter 4	40.82%	1	1	25	82						
IPI00129959.2	Exocyst complex component 4	82.67%	4	6	100	353	-0.065	0.011	-0.056	0.026	-0.342	8
IPI00876027.2	Exocyst complex component 5	73.59%	3	3	64	161						
IPI00377299.1	Exocyst complex component 8	77.79%	1	5	75	192	-0.108	-0.051	-0.152	-0.042	-0.120	5
IPI00466593.1	Exosome complex component MTR3	69.23%	1	7	22	116	-0.130	-0.007	-0.065	0.016	-0.023	13
IPI00121437.1	Exosome complex component RRP4	85.67%	2	2	30	106	-0.051	0.091	-0.114	-0.108	-0.085	6
IPI00396812.3	Exosome complex component RRP40	85.40%	2	3	27	88	0.189	-0.068	0.001	0.240	-0.167	4
IPI00310857.4	Exosome complex component RRP41	77.14%	1	4	20	62						
IPI00109639.6	Exosome complex component RRP43	60.14%	3	4	16	73	0.302	0.220	-0.119	0.151	-0.231	3
IPI00119442.1	Exosome complex component RRP45	71.69%	2	5	36	166						
IPI00133532.1	Exosome complex component RRP46	53.19%	1	2	11	33	0.027	-0.023	-0.122	0.029	0.103	3
IPI00407764.6	Exosome complex exonuclease RRP44	80.38%	2	20	103	495	-0.004	0.126	0.038	0.041	0.143	18
IPI00342382.3	Exosome component 10	79.14%	6	8	85	315	0.106	0.336	0.285	0.170	-0.060	3
IPI00395038.3	Exportin-1	78.06%	2	40	99	972	-0.178	-0.001	-0.160	0.006	-0.144	48
IPI00112414.1	Exportin-2	81.36%	6	37	102	1108	-0.168	-0.026	-0.246	-0.037	-0.257	65
IPI00331063.7	Exportin-4	72.55%	2	6	84	259	-0.066	0.079	-0.196	0.075	-0.028	6
IPI00116741.10	Exportin-T	68.12%	1	7	62	209						
IPI00330862.5	Ezrin	84.30%	1	28	96	1250	0.245	-0.023	0.294	-0.001	0.294	146
IPI00120344.2	FACT complex subunit SPT16	77.84%	1	30	131	810	0.050	0.073	0.020	0.050	0.007	44
IPI00330063.6	F-actin-capping protein subunit alpha-1	88.46%	5	11	28	433	0.033	-0.019	0.051	0.085	0.013	41
IPI00111265.3	F-actin-capping protein subunit alpha-2	86.01%	2	7	29	427	0.004	0.001	-0.079	0.001	-0.032	37
IPI00986569.1	Fam3c protein (Fragment)	71.06%	5	5	26	64	0.051	0.004	-0.119	0.091	-0.009	7
IPI00379513.6	far upstream element (FUSE) binding protein 3	87.83%	6	12	63	413	-0.048	0.004	0.001	-0.042	-0.048	19
IPI00462934.3	Far upstream element-binding protein 2	67.25%	3	32	73	1249	-0.012	0.058	-0.034	-0.012	-0.082	91
IPI00120457.1	Farnesyl pyrophosphate synthase	92.63%	1	13	34	355	0.194	0.047	0.405	-0.068	0.345	20
IPI00108791.2	FAS-associated factor 1	68.41%	1	9	54	262	0.004	-0.139	-0.020	0.064	-0.003	12
IPI00353563.4	Fascin	94.52%	5	28	60	876						
IPI00623114.6	FAT tumor suppressor homolog 1	61.96%	2	5	280	790	0.141	-0.031	0.072	-0.071	0.228	4
IPI00129362.1	Fatty acid desaturase 2	46.85%	1	9	25	149	0.245	0.079	0.072	-0.003	0.103	13
IPI00113223.2	Fatty acid synthase	78.71%	3	103	222	2589	0.058	-0.028	0.115	-0.009	0.075	171
IPI00227585.6	Fatty acid-binding protein, brain	67.42%	2	3	11	37						
IPI00114162.3	Fatty acid-binding protein, epidermal	88.89%	2	7	17	233	-0.085	-0.068	-0.048	0.031	-0.037	14
IPI00230124.5	Fatty acid-binding protein, heart	76.69%	1	4	18	89						
IPI00153176.2	F-box only protein 2	55.56%	1	1	12	59						
IPI00894941.1	F-box only protein 2	55.56%	1	1	12	67						
IPI00120982.4	F-box only protein 22	60.20%	3	2	22	62	-0.017	-0.023	0.170	0.040	0.286	4
IPI00108960.3	F-box/LRR-repeat protein 16	43.22%	1	3	20	45						
IPI00223056.3	F-box-like/WD repeat-containing protein TBL1X	71.92%	1	5	30	115						
IPI00308283.1	F-box-like/WD repeat-containing protein TBL1XR1	77.04%	1	2	29	89						

IPI00276222.1	Fermitin family homolog 2	80.88%	4	16	52	217	[illegible]
IPI00356904.7	FERMRhoGEF (Arhgef) and pleckstrin domain protein 1	73.66%	2	13	96	336	[illegible]
IPI00322418.3	Ferric-chelate reductase 1	67.40%	1	4	33	97	[illegible]
IPI00230145.5	Ferritin heavy chain	82.42%	1	9	23	191	[illegible]
IPI00608020.4	ferritin light chain 1	86.89%	4	8	24	407	[illegible]
IPI00228343.4	Ferrochelatase	90.76%	2	9	49	242	[illegible]
IPI00223046.6	FH1/FH2 domain-containing protein 1	78.61%	2	9	96	301	[illegible]
IPI00279079.1	Fibrinogen beta chain	74.22%	1	5	40	92	[illegible]
IPI00990997.1	Fibrinogen gamma chain	75.00%	2	3	24	57	[illegible]
IPI00885793.1	fibrinogen, alpha polypeptide isoform 1	48.92%	2	8	59	155	[illegible]
IPI00229601.6	Fibronectin type III domain-containing protein 3B	54.43%	2	5	70	183	[illegible]
IPI00750014.6	Fibrous sheath-interacting protein 2	76.18%	5	5	669	2703	[illegible]
IPI00830803.1	fibulin-2 isoform b	61.58%	3	6	51	162	[illegible]
IPI00331030.3	Fidgetin-like protein 1	72.47%	1	1	60	257	[illegible]
IPI00875567.1	filamin-A	87.00%	8	123	260	4624	[illegible]
IPI00663627.2	Filamin-B	84.59%	1	121	267	3486	[illegible]
IPI00113996.7	Flavin reductase	95.63%	3	4	21	104	[illegible]
IPI00117181.1	Flotillin-1	81.54%	4	8	46	176	[illegible]
IPI00124707.2	Follistatin-related protein 1	66.01%	1	5	23	64	[illegible]
IPI00656285.2	Forkhead box protein K1	51.60%	2	1	35	127	[illegible]
IPI00338178.3	Fos-related antigen 2	63.19%	1	2	22	73	[illegible]
IPI00776276.1	four and a half LIM domains protein 1 isoform 1	79.26%	4	2	33	146	[illegible]
IPI00858310.1	Fructose-bisphosphate aldolase	96.20%	3	2	33	1215	[illegible]
IPI00856379.1	fructose-bisphosphate aldolase A isoform 1	97.85%	5	10	51	1783	[illegible]
IPI00119458.7	Fructose-bisphosphate aldolase C	88.15%	2	18	44	754	[illegible]
IPI00310035.4	Fumarylacetoacetase	28.64%	2	3	13	36	[illegible]
IPI00653743.1	fumarylacetoacetate hydrolase domain-containing protein 1	76.65%	2	3	16	38	[illegible]
IPI00121218.5	Fumarylacetoacetate hydrolase domain-containing protein 2A	64.22%	1	7	25	74	[illegible]
IPI00466200.3	FUN14 domain-containing protein 2	76.16%	1	2	14	35	[illegible]
IPI00309815.6	G patch domain and KOW motifs-containing protein	60.45%	2	3	40	101	[illegible]
IPI00138232.4	G protein-regulated inducer of neurite outgrowth 1	74.79%	1	26	96	332	[illegible]
IPI00109561.1	G1/S-specific cyclin-D1	68.47%	2	4	22	63	[illegible]
IPI00118948.4	GA-binding protein alpha chain	79.96%	2	6	41	171	[illegible]
IPI00675491.2	Gag	71.46%	3	6	79	478	[illegible]
IPI00464344.2	Gag protein	66.80%	1	1	15	73	[illegible]
IPI00466581.4	Gag protein	79.01%	1	3	65	417	[illegible]
IPI00108206.1	Gag-Pol polyprotein	72.49%	3	9	154	649	[illegible]
IPI00265025.5	galactokinase	79.08%	1	12	29	201	[illegible]
IPI00229517.5	Galectin-1	95.56%	1	11	15	813	[illegible]
IPI00315771.1	Galectin-2	92.31%	1	8	15	93	[illegible]
IPI00929878.1	galectin-9 isoform 1	71.39%	3	4	24	113	[illegible]
IPI00124501.1	Galectin-related protein A	81.40%	2	5	14	71	[illegible]
IPI00113772.1	Gamma-aminobutyric acid receptor subunit alpha-1	63.96%	3	1	37	143	[illegible]
IPI00110598.1	Gamma-aminobutyric acid receptor subunit alpha-2	70.95%	4	1	39	193	[illegible]
IPI00119283.1	Gamma-aminobutyric acid receptor subunit beta-1	50.63%	2	1	26	66	[illegible]
IPI00130546.1	Gamma-aminobutyric acid receptor subunit beta-3	60.68%	3	1	26	50	[illegible]
IPI00850638.1	gamma-aminobutyric acid receptor-associated protein-like 2-like	90.60%	3	4	13	47	[illegible]
IPI00409336.4	Gamma-aminobutyric acid type B receptor subunit 2	54.89%	1	4	55	243	[illegible]
IPI00331704.7	Gamma-enolase	96.31%	6	16	57	873	[illegible]
IPI00109870.1	Gamma-soluble NSF attachment protein	79.17%	2	17	34	294	[illegible]
IPI00121787.1	Gamma-tubulin complex component 2	82.54%	2	5	93	321	[illegible]
IPI00396839.1	Gamma-tubulin complex component 3	69.39%	9	8	80	196	[illegible]
IPI00119095.3	Ganglioside GM2 activator	23.32%	1	3	5	23	[illegible]
IPI00620728.2	ganglioside-induced differentiation-associated protein 1-like 1	78.20%	3	5	24	68	[illegible]
IPI00380273.1	Gap junction protein	61.98%	2	8	30	224	[illegible]
IPI00111986.1	Gasdermin-D	77.21%	1	11	39	189	[illegible]
IPI00457499.5	GCN1 general control of amino-acid synthesis 1-like 1	83.11%	4	67	303	1548	[illegible]
IPI00894776.2	GDH/6PGL endoplasmic bifunctional protein	51.08%	6	7	47	141	[illegible]
IPI00128336.3	GDP-fucose protein O-fucosyltransferase 2	64.10%	1	2	22	55	[illegible]
IPI00133690.2	GDP-L-fucose synthase	80.69%	1	7	22	104	[illegible]
IPI00954312.1	gem-associated protein 5 isoform 3	67.55%	5	8	106	261	[illegible]
IPI00169775.6	gemin 4	56.05%	1	5	61	236	[illegible]
IPI00132417.1	General transcription factor IIE subunit 1	50.00%	3	7	31	125	[illegible]
IPI00331112.4	General transcription factor IIE subunit 2	80.48%	3	5	43	114	[illegible]
IPI00153986.3	General transcription factor IIF subunit 1	76.38%	2	7	51	181	[illegible]
IPI00135812.2	General transcription factor IIF subunit 2	69.08%	1	5	14	33	[illegible]
IPI00225670.2	Gephyrin	73.34%	2	12	64	250	[illegible]
IPI00467495.4	Glia maturation factor, beta	82.52%	6	4	13	124	[illegible]
**IPI00379245.2**	**Glucosamine-6-phosphate isomerase 1**	**56.75%**	**3**	**3**	**18**	**51**	[illegible]
IPI00228059.4	Glucose 1,6-bisphosphate synthase	85.67%	3	13	69	224	[illegible]
IPI00228867.5	Glucose-6-phosphate 1-dehydrogenase 2	65.89%	1	1	43	257	[illegible]
IPI00228385.5	Glucose-6-phosphate 1-dehydrogenase X	79.42%	2	15	53	509	[illegible]
IPI00228633.7	Glucose-6-phosphate isomerase	83.87%	4	37	72	1217	[illegible]
IPI00318496.1	Glutamate decarboxylase 1	49.92%	4	8	37	130	[illegible]
IPI00318522.3	Glutamate decarboxylase 2	57.95%	2	11	36	131	[illegible]
IPI00114209.1	Glutamate dehydrogenase 1, mitochondrial	88.89%	3	32	74	881	[illegible]
IPI00407939.1	glutamate receptor 1 isoform 2 precursor	61.08%	3	4	60	161	[illegible]
IPI01023161.1	glutamate receptor 4 isoform 2 precursor	57.54%	3	2	58	155	[illegible]
IPI00123534.1	Glutamate receptor delta-2 subunit	63.75%	2	6	69	215	[illegible]
IPI00125658.5	Glutamate--cysteine ligase catalytic subunit	80.53%	1	14	49	158	[illegible]
IPI00464317.3	glutaminase isoform 1	92.88%	4	4	83	752	[illegible]
IPI00671957.2	glutaminase isoform 2	96.35%	4	2	75	698	[illegible]
IPI00132593.4	Glutaminase liver isoform, mitochondrial	61.13%	1	1	45	142	[illegible]
IPI00626790.3	Glutamine synthetase	80.16%	3	19	36	328	[illegible]
IPI00225526.2	Glutamine-rich protein 1	41.57%	1	3	48	135	[illegible]
IPI00223055.3	glutaminyl-tRNA synthetase isoform 1	78.32%	4	18	82	274	[illegible]
IPI00331528.9	Glutaredoxin-1	87.85%	1	4	13	63	[illegible]
IPI00315550.3	Glutaredoxin-3	94.66%	1	15	36	278	[illegible]
IPI00378120.2	Glutaredoxin-related protein 5, mitochondrial	92.76%	6	4	14	122	[illegible]
IPI00120233.2	Glutaryl-CoA dehydrogenase, mitochondrial	67.12%	4	5	38	88	[illegible]
IPI00463220.2	Glutathione peroxidase	60.59%	1	1	13	56	[illegible]
IPI00319652.2	Glutathione peroxidase 1	73.63%	1	6	14	55	[illegible]
IPI00116055.6	Glutathione S-transferase A2	86.94%	8	5	28	111	[illegible]
IPI00121051.3	Glutathione S-transferase kappa 1	62.39%	1	3	19	58	[illegible]
IPI00230212.5	Glutathione S-transferase Mu 1	94.50%	9	9	35	322	[illegible]
IPI00228820.5	Glutathione S-transferase Mu 2	83.03%	6	5	24	112	[illegible]
IPI00338855.3	Glutathione S-transferase Mu 3	77.06%	3	1	28	164	[illegible]
IPI00114380.1	Glutathione S-transferase Mu 5	84.82%	5	2	25	129	[illegible]
IPI00114285.1	Glutathione S-transferase omega-1	97.08%	3	16	33	257	[illegible]
IPI00127691.1	Glutathione synthetase	66.03%	3	6	43	124	[illegible]
IPI00123176.1	Glyceraldehyde-3-phosphate dehydrogenase	97.94%	5	2	45	1196	[illegible]
IPI00752289.2	Glyceraldehyde-3-phosphate dehydrogenase	98.50%	5	2	40	1086	[illegible]
IPI00874682.1	Glyceraldehyde-3-phosphate dehydrogenase	98.50%	8	1	46	1653	[illegible]
IPI00986980.1	Glyceraldehyde-3-phosphate dehydrogenase	95.48%	5	2	40	1350	[illegible]
IPI00989794.1	Glyceraldehyde-3-phosphate dehydrogenase	95.82%	3	2	38	827	[illegible]
IPI00990452.1	Glyceraldehyde-3-phosphate dehydrogenase	91.29%	5	1	38	1614	[illegible]
IPI00990544.1	Glyceraldehyde-3-phosphate dehydrogenase	97.31%	5	2	41	1268	[illegible]
IPI00990885.1	Glyceraldehyde-3-phosphate dehydrogenase	73.73%	1	1	28	219	[illegible]
IPI00889899.2	Glyceraldehyde-3-phosphate dehydrogenase (Fragment)	88.06%	3	2	36	394	[illegible]
IPI00988275.1	Glyceraldehyde-3-phosphate dehydrogenase (Fragment)	93.69%	3	3	23	695	[illegible]
IPI00988324.1	glyceraldehyde-3-phosphate dehydrogenase-like	97.30%	5	1	46	1605	[illegible]
IPI00123975.1	Glycerol kinase 2	58.66%	1	1	36	126	[illegible]
IPI00453663.1	glycerol kinase-like 1	44.44%	1	2	21	79	[illegible]
IPI00230185.5	Glycerol-3-phosphate dehydrogenase [NAD+], cytoplasmic	81.66%	2	14	36	132	[illegible]
IPI00134420.1	Glycerophosphodiester phosphodiesterase domain-containing protein 1	73.89%	1	4	25	89	[illegible]
IPI00112129.1	Glycine amidinotransferase, mitochondrial	79.20%	1	15	35	249	[illegible]
IPI00453724.1	Glycine cleavage system H protein, mitochondrial	68.24%	2	1	12	63	[illegible]
IPI00130127.2	Glycogen [starch] synthase, muscle	61.52%	2	5	48	135	[illegible]
IPI00229796.3	Glycogen phosphorylase, brain form	92.41%	1	30	103	590	[illegible]
IPI00319525.4	Glycogen phosphorylase, liver form	84.00%	2	2	86	350	[illegible]
IPI00225275.5	Glycogen phosphorylase, muscle form	85.15%	2	19	103	441	[illegible]
IPI00648141.1	Glycogen synthase kinase-3 alpha	71.63%	9	4	35	121	[illegible]
IPI00264062.6	Glycogenin-1	45.65%	2	6	18	102	[illegible]
IPI00229718.10	Glycolipid transfer protein	86.12%	3	7	18	97	[illegible]
IPI00224128.7	Glycylpeptide N-tetradecanoyltransferase 1	77.02%	1	12	52	581	[illegible]
IPI00117252.1	Glycylpeptide N-tetradecanoyltransferase 2	79.96%	3	3	50	394	[illegible]
IPI00112555.3	Glycyl-tRNA synthetase	92.18%	2	30	93	849	[illegible]
IPI00130530.1	Glyoxylate reductase/hydroxypyruvate reductase	73.48%	3	7	31	116	[illegible]
IPI00137336.1	Glypican-1	90.66%	1	19	57	294	[illegible]
IPI00121566.1	GMP reductase 1	41.74%	1	1	12	29	[illegible]
IPI00116248.2	GMP reductase 2	68.10%	1	1	22	40	[illegible]
IPI00351252.4	GMP synthase [glutamine-hydrolyzing]	88.31%	1	31	72	504	[illegible]
IPI00122399.1	Golgi apparatus protein 1	77.53%	4	30	139	637	[illegible]
IPI00929857.2	golgi autoantigen, golgin subfamily b, macrogolgin 1	82.17%	2	21	385	1552	[illegible]
IPI00128905.1	Golgi membrane protein 1	75.32%	1	4	39	130	[illegible]
IPI00129907.7	Golgi resident protein GCP60	57.14%	4	10	31	487	[illegible]
IPI00416279.3	Golgi-associated plant pathogenesis-related protein 1	98.05%	2	5	24	166	[illegible]
IPI00755997.2	Golgin subfamily A member 2 isoform a	78.27%	7	14	116	530	[illegible]
IPI00125681.4	GPI transamidase component PIG-S	64.32%	2	2	36	132	[illegible]
IPI00222723.1	GPI-anchor transamidase isoform 2	44.57%	4	2	14	33	[illegible]
IPI00124640.4	granulins	53.65%	1	2	27	55	[illegible]
IPI00453582.2	G-rich sequence factor 1	79.54%	2	6	37	95	[illegible]
IPI00377455.3	GRIP and coiled-coil domain-containing protein 2	84.11%	8	11	209	655	[illegible]
IPI00267855.4	GRIP1-associated protein 1	81.89%	5	5	95	310	[illegible]
IPI00126721.3	Growth hormone-inducible transmembrane protein	52.89%	1	3	20	63	[illegible]
IPI00117083.1	GrpE protein homolog 1, mitochondrial	81.57%	1	13	29	255	[illegible]
IPI00114691.1	GTP cyclohydrolase 1	74.27%	1	2	21	71	[illegible]
IPI00221769.5	GTP:AMP phosphotransferase, mitochondrial	99.56%	2	11	35	294	[illegible]
IPI00134621.3	GTP-binding nuclear protein Ran	97.69%	1	5	23	594	[illegible]
IPI00126133.1	GTP-binding nuclear protein Ran, testis-specific isoform	81.02%	1	1	14	198	[illegible]
IPI00761677.1	GTP-binding protein 1	84.28%	2	11	69	350	[illegible]
IPI00169475.6	GTP-binding protein 10	76.50%	6	3	33	126	[illegible]
IPI00130567.1	GTP-binding protein Di-Ras1	74.24%	2	3	19	108	[illegible]
IPI00126551.1	GTP-binding protein Di-Ras2	93.47%	1	6	25	91	[illegible]
IPI00177205.1	GTP-binding protein Rheb	59.78%	1	5	13	73	[illegible]
IPI00132397.1	GTP-binding protein SAR1b	92.42%	1	4	18	170	[illegible]
IPI00469987.3	Guanine deaminase	72.25%	3	14	36	162	[illegible]
IPI00466436.4	guanine nucleotide exchange factor DBS isoform 1	64.34%	19	1	73	136	[illegible]
IPI00467152.4	Guanine nucleotide-binding protein G(i) subunit alpha-1	68.36%	5	5	37	267	[illegible]
IPI00228617.7	Guanine nucleotide-binding protein G(i) subunit alpha-2	79.44%	6	11	40	415	[illegible]
IPI00227838.4	Guanine nucleotide-binding protein G(I)/G(S)/G(O) subunit gamma-12	87.50%	1	5	15	111	[illegible]
IPI00125602.1	Guanine nucleotide-binding protein G(I)/G(S)/G(O) subunit gamma-13	80.60%	1	3	7	24	[illegible]
IPI00230194.5	Guanine nucleotide-binding protein G(I)/G(S)/G(O) subunit gamma-2	87.32%	1	4	7	58	[illegible]
IPI00129268.1	Guanine nucleotide-binding protein G(I)/G(S)/G(O) subunit gamma-3	90.67%	1	5	8	54	[illegible]
IPI00115544.1	Guanine nucleotide-binding protein G(I)/G(S)/G(O) subunit gamma-4	80.00%	1	3	8	38	[illegible]
IPI00120716.3	Guanine nucleotide-binding protein G(I)/G(S)/G(T) subunit beta-1	96.76%	1	8	34	411	[illegible]
IPI00116938.1	Guanine nucleotide-binding protein G(I)/G(S)/G(T) subunit beta-3	65.88%	1	1	21	78	[illegible]
IPI00338854.3	Guanine nucleotide-binding protein G(k) subunit alpha	83.05%	5	5	45	361	[illegible]
IPI00229525.1	Guanine nucleotide-binding protein G(olf) subunit alpha	85.30%	5	3	40	156	[illegible]
IPI00228618.5	Guanine nucleotide-binding protein G(q) subunit alpha	80.22%	1	7	40	205	[illegible]
IPI00330646.4	Guanine nucleotide-binding protein G(t) subunit alpha-1	74.00%	4	1	35	136	[illegible]
IPI00347770.3	Guanine nucleotide-binding protein G(t) subunit alpha-3	82.49%	4	2	37	233	[illegible]
IPI00230193.7	Guanine nucleotide-binding protein G(z) subunit alpha	92.11%	1	12	34	143	[illegible]
IPI00121387.1	Guanine nucleotide-binding protein subunit alpha-11	79.11%	2	6	37	186	[illegible]
IPI00230191.5	Guanine nucleotide-binding protein subunit alpha-12	81.53%	4	1	49	220	[illegible]
IPI00118569.1	Guanine nucleotide-binding protein subunit alpha-13	91.25%	4	12	43	229	[illegible]
IPI00317740.5	Guanine nucleotide-binding protein subunit beta-2-like 1	100.00%	1	21	42	744	[illegible]
IPI00108818.3	Guanine nucleotide-binding protein-like 3-like protein	77.47%	1	2	60	135	[illegible]
IPI00132959.1	Guanosine-3',5'-bis(diphosphate) 3'-pyrophosphohydrolase MESH1	42.46%	1	2	11	54	[illegible]
IPI00118880.5	Guanylate cyclase soluble subunit beta-1	55.48%	4	6	39	77	[illegible]
IPI00929789.1	guanylate kinase isoform 2	88.41%	3	2	17	44	[illegible]
IPI00133550.1	H/ACA ribonucleoprotein complex subunit 2	92.81%	1	3	15	89	[illegible]
IPI00133008.1	H/ACA ribonucleoprotein complex subunit 3	73.44%	1	4	8	28	[illegible]
IPI00110805.1	H-2 class I histocompatibility antigen, D-D alpha chain	67.67%	29	1	34	126	[illegible]
IPI00126300.1	H-2 class I histocompatibility antigen, D-K alpha chain	65.75%	22	1	32	157	[illegible]
IPI00110641.1	HAI-2 related small protein	35.09%	1	2	4	30	[illegible]
IPI00129964.1	HCLS1-associated protein X-1	73.21%	2	4	23	80	[illegible]
IPI00130235.4	HCLS1-binding protein 3	70.89%	1	5	29	51	[illegible]
IPI00137694.5	HD domain-containing protein 2	61.81%	1	4	15	61	[illegible]
IPI00323705.2	Hdac6 protein	48.70%	3	11	54	192	[illegible]
IPI00399686.1	HEAT repeat-containing protein 3	73.64%	2	10	54	307	[illegible]
IPI00798482.20	Heat shock 70 kDa protein 1A	87.36%	2	1	69	950	[illegible]
IPI00346073.1	Heat shock 70 kDa protein 1B	87.23%	2	1	68	942	[illegible]
IPI00133208.3	Heat shock 70 kDa protein 1-like	80.81%	1	2	73	968	[illegible]
IPI00331556.5	Heat shock 70 kDa protein 4	95.96%	1	53	119	2648	[illegible]
IPI00323357.3	Heat shock cognate 71 kDa protein	97.68%	4	39	96	4002	[illegible]
IPI00133387.1	Heat shock factor-binding protein 1	98.68%	1	3	14	98	[illegible]
IPI00132762.1	Heat shock protein 75 kDa, mitochondrial	90.37%	2	29	100	837	[illegible]
IPI00330804.4	Heat shock protein HSP 90-alpha	94.95%	6	36	103	2373	[illegible]
IPI00554929.3	Heat shock protein HSP 90-beta	88.54%	5	35	102	3042	[illegible]
IPI00331546.3	Heat shock-related 70 kDa protein 2	91.94%	1	8	77	1406	[illegible]
IPI00314755.5	Hematological and neurological expressed 1 protein	99.35%	1	8	18	282	[illegible]
IPI00107958.1	Hematological and neurological expressed 1-like protein	100.00%	2	6	28	166	[illegible]
IPI00131577.1	Heme oxygenase 1	90.66%	2	9	25	133	[illegible]
IPI00309322.3	Heme oxygenase 2	89.21%	4	12	37	252	[illegible]
**IPI00135085.4**	**Heme-binding protein 1**	**96.32%**	**2**	**12**	**25**	**187**	[illegible]
IPI00132747.5	hepatitis B virus X-interacting protein homolog	64.83%	1	1	7	23	[illegible]
IPI00471176.2	Hepatocyte cell adhesion molecule	60.53%	1	6	41	134	[illegible]
IPI00929837.1	hepatocyte growth factor-regulated tyrosine kinase substrate isoform 1	68.71%	4	11	64	234	[illegible]
IPI00119671.2	Hepatocyte nuclear factor 3-beta	42.92%	3	1	16	58	[illegible]
IPI00313817.1	Hepatoma-derived growth factor	86.92%	3	15	28	444	[illegible]
IPI00117288.3	Heterogeneous nuclear ribonucleoprotein A/B	69.82%	3	19	37	686	[illegible]
IPI00109813.1	heterogeneous nuclear ribonucleoprotein A0	97.05%	1	11	35	408	[illegible]
IPI00755892.1	heterogeneous nuclear ribonucleoprotein D-like	67.38%	3	9	48	402	[illegible]
IPI00663587.1	Heterogeneous nuclear ribonucleoprotein G-like 1	91.75%	4	7	66	837	[illegible]
IPI00108143.1	Heterogeneous nuclear ribonucleoprotein H2	75.72%	2	7	42	586	[illegible]
IPI00816884.1	heterogeneous nuclear ribonucleoprotein H3	95.09%	3	10	34	264	[illegible]
IPI00458583.3	Heterogeneous nuclear ribonucleoprotein U	74.13%	2	38	101	1598	[illegible]
IPI00114342.1	Hexokinase-2	84.19%	3	19	105	410	[illegible]
IPI00121634.1	High affinity cationic amino acid transporter 1	73.47%	3	5	35	231	[illegible]
IPI00311626.5	High mobility group nucleosome-binding domain-containing protein 5	65.52%	1	11	32	96	[illegible]
IPI00420261.5	High mobility group protein B1	80.00%	10	1	30	699	[illegible]
IPI00462291.5	High mobility group protein B2	75.24%	1	18	35	420	[illegible]
IPI00228879.7	High mobility group protein B3	79.50%	3	7	27	182	[illegible]
IPI00331612.3	High mobility group protein HMGI-C	68.52%	2	6	15	148	[illegible]
IPI00230449.7	Hippocalcin-like protein 1	83.42%	2	5	21	233	[illegible]
IPI00222813.1	HIRA-interacting protein 3	81.20%	2	3	67	207	[illegible]
IPI00126826.1	Histamine N-methyltransferase	59.66%	1	2	21	45	[illegible]
IPI00108189.4	Histidine triad nucleotide-binding protein 1	95.24%	2	8	16	148	[illegible]
IPI00133034.3	Histidine triad nucleotide-binding protein 2, mitochondria	96.93%	1	3	20	93	[illegible]
IPI00131903.1	Histidine triad nucleotide-binding protein 3	20.00%	1	3	3	4	[illegible]
IPI00116991.2	Histidyl-tRNA synthetase, cytoplasmic	82.12%	1	16	71	383	[illegible]
IPI00461822.6	Histone acetyltransferase p300	54.66%	2	1	120	358	[illegible]
IPI00114232.1	Histone deacetylase 1	76.14%	2	11	44	352	[illegible]
IPI00137668.1	Histone deacetylase 2	80.74%	3	6	39	344	[illegible]
IPI00135456.4	histone deacetylase 3	50.23%	2	4	16	42	[illegible]
IPI00467914.3	Histone H1.0	80.93%	2	12	37	423	[illegible]
IPI00228616.5	Histone H1.1	95.77%	1	11	44	454	[illegible]
IPI00223713.5	Histone H1.2	88.21%	2	7	50	1048	[illegible]
IPI00331597.6	Histone H1.3	89.59%	2	3	45	1226	[illegible]
IPI00223714.5	Histone H1.4	91.32%	2	7	49	1154	[illegible]
IPI00230133.5	Histone H1.5	90.58%	1	17	50	1086	[illegible]
IPI00229542.1	Histone H2A	89.92%	1	2	21	501	[illegible]
IPI00229543.3	Histone H2A type 1-F	91.54%	1	1	23	586	[illegible]
IPI00229545.3	Histone H2A type 1-K	91.54%	1	1	24	591	[illegible]
IPI00555055.3	Histone H2A.V	86.05%	6	4	15	225	[illegible]
IPI00230264.5	Histone H2A.x	92.31%	1	9	23	606	[illegible]
IPI00227930.3	Histone H2B type 1-H	85.71%	11	2	28	1027	[illegible]
IPI00553774.3	Histone H2B type 3-A	83.33%	7	3	25	829	[illegible]
IPI00553538.3	Histone H3.1	88.24%	5	1	25	542	[illegible]
IPI00986082.1	Histone H3.3-like	86.76%	20	3	24	479	[illegible]
IPI00407339.7	Histone H4	87.38%	1	11	17	579	[illegible]
IPI00122698.1	Histone-binding protein RBBP7	60.71%	5	6	23	210	[illegible]

IPI00331236.4	Histone-lysine N-methyltransferase SETD7	35.25%	1	4	10	22																									0.141	0.075	-0.050	-0.208	0.010	6	366	40.5	4.65
IPI01026634.1	Hnrnpa1l2 protein	95.27%	1	1	31	278	-0.114	-0.091	0.019	0.036	0.092	6	-0.176	-0.028	-0.139	-0.019	-0.300	11	0.163	0.127	0.123	-0.013	0.124	16	-0.297	-0.189	-0.009	-0.150	0.092	6	0.017	0.247	0.138	0.140	0.268	4	275	29.1	9.26
IPI00129604.1	Homeobox protein Rhox5	75.24%	2	3	16	34																			-0.228	0.031	0.124	-0.089	0.087	4							210	23.0	6.25
IPI00828543.3	Host cell factor 1	49.68%	3	22	107	817	0.019	0.040	0.066	0.050	0.036	16	-0.057	-0.060	-0.117	-0.041	-0.203	25	-0.010	0.025	0.135	-0.017	0.134	5	0.256	-0.060	0.303	-0.017	0.352	16							2045	210.3	7.18
IPI00474317.5	host cell factor 2	70.22%	1	1	51	154							1.021	-0.226	1.329	-0.189	-0.010	3																			722	79.4	8.35
IPI00116308.1	Hsc70-interacting protein	56.06%	4	19	40	598	-0.045	-0.050	0.036	-0.059	-0.017	59	-0.062	-0.102	-0.127	-0.171	-0.094	23	-0.423	-0.122	-0.351	0.070	-0.224	55	0.118	0.004	0.165	-0.042	0.292	49	0.034	-0.004	0.103	-0.004	0.066	34	371	41.6	5.26
IPI00120257.1	Hsp70-binding protein 1	76.47%	1	6	33	165	0.210	0.124	0.345	0.070	0.160	4													0.392	0.203	0.350	-0.085	0.289	6							357	39.1	5.36
IPI00117087.1	Hsp90 co-chaperone Cdc37	72.03%	1	14	35	343	0.030	-0.026	0.043	-0.007	0.076	26	0.040	-0.108	-0.025	-0.103	0.007	18	-0.290	-0.143	-0.234	0.114	-0.130	25	0.165	-0.105	0.128	-0.071	0.267	25	-0.060	-0.031	-0.029	-0.062	-0.079	20	379	44.6	5.34
IPI00131995.1	Hyaluronan and proteoglycan link protein 1	75.56%	1	12	28	129																									0.054	0.006	0.088	0.038	0.023	41	356	40.5	7.80
IPI00113410.1	Hyaluronan and proteoglycan link protein 2	62.46%	1	8	23	82																									0.076	0.219	0.151	0.156	0.099	20	341	37.9	9.09
IPI00229184.3	Hyaluronan and proteoglycan link protein 4	62.50%	1	3	24	56																									0.036	-0.204	-0.041	-0.065	-0.255	6	400	42.8	8.85
IPI00121105.2	Hydroxyacyl-coenzyme A dehydrogenase, mitochondria	95.54%	2	12	37	373	0.180	-0.054	0.091	-0.050	0.252	15	-0.368	0.021	-0.127	0.222	-0.313	20	0.185	0.106	0.275	0.064	0.092	13	0.288	-0.017	0.374	-0.094	0.431	3	-0.037	-0.031	-0.051	-0.028	-0.019	10	314	34.4	8.65
IPI00127625.2	Hydroxymethylglutaryl-CoA lyase, mitochondrial	82.46%	1	5	40	144							-0.513	0.094	-0.290	0.080	-0.490	6													0.485	-0.095	-0.120	0.479	-0.482	3	325	34.2	8.41
IPI00331707.1	Hydroxymethylglutaryl-CoA synthase, cytoplasmic	87.50%	3	10	44	215	0.064	-0.097	0.238	-0.012	0.358	13	-0.308	0.424	0.194	0.472	-0.189	13	-0.484	-0.146	-0.617	-0.028	-0.370	9													520	57.5	5.99
IPI00117214.3	Hydroxysteroid dehydrogenase-like protein 2	72.86%	3	6	44	180																									-0.326	0.283	-0.136	-0.076	0.111	4	490	54.2	6.74
IPI00989858.1	hypothetical protein LOC100504864	60.83%	2	1	142	486																									0.294	0.167	0.138	0.230	0.141	5	1514	179.4	8.72
IPI00222228.5	hypothetical protein LOC239673	76.77%	2	1	70	283	0.700	0.181	0.785	0.091	0.742	7	0.543	-0.001	0.225	-0.016	0.188	10	0.143	0.093	-0.009	-0.082	-0.069	7							0.075	0.072	0.017	-0.056	0.131	10	538	58.2	8.25
IPI00605376.1	hypothetical protein LOC627788	74.16%	1	1	8	183	-0.057	0.098	-0.028	0.003	-0.216	7	0.020	-0.047	0.062	-0.306	-0.016	7	-0.216	-0.074	-0.245	0.111	0.065	10													89	10.3	7.88
IPI00988780.1	hypothetical protein LOC638791	86.96%	1	1	21	141	-0.175	0.170	-0.309	0.070	-0.170	6													0.393	-0.109	0.348	-0.193	0.314	7							184	20.1	4.92
IPI00988958.1	hypothetical protein LOC667284	65.59%	3	1	46	396	0.072	-0.057	0.169	-0.019	0.220	21	0.013	0.128	0.103	0.091	-0.139	8	0.222	-0.072	0.314	0.328	0.352	17	0.740	0.158	0.683	0.089	0.560	18	-0.088	0.026	0.225	0.143	0.104	17	433	49.4	6.13
IPI00849550.2	hypothetical protein LOC670211	82.51%	27	7	38	371	0.161	0.084	0.181	0.024	0.128	21	0.212	0.077	0.214	0.116	0.040	23	0.070	0.111	0.087	-0.001	0.014	31	0.135	0.089	0.156	0.050	0.151	20	-0.198	0.116	-0.053	0.020	-0.147	14	263	28.5	10.84
IPI00221580.1	hypothetical protein LOC67892	64.56%	1	2	5	19							-0.150	0.017	0.145	-0.194	0.143	8																			79	9.3	8.16
IPI00954636.1	hypothetical protein LOC67894 isoform 2	64.57%	3	2	33	82	0.007	0.098	0.116	0.128	0.290	4																									350	39.7	6.47
IPI00672962.4	hypothetical protein LOC70715	80.82%	1	1	37	143													0.144	-0.090	-0.050	0.246	0.118	3							0.247	0.110	0.026	0.222	0.344	5	391	42.5	9.95
IPI00454053.7	hypothetical protein LOC72097	68.34%	2	5	106	373																									0.181	0.313	0.001	0.245	0.213	5	1175	125.8	7.37
IPI00347394.7	hypothetical protein LOC74148	71.84%	3	19	105	443	-0.181	0.080	-0.191	0.047	-0.193	29	0.247	-0.208	0.265	0.236	0.417	4	-0.091	-0.052	-0.189	-0.097	-0.183	6													1353	151.7	6.02
IPI00761429.2	hypothetical protein LOC75964 isoform 1	70.56%	2	6	104	273	-0.383	0.151	0.024	-0.102	-0.134	3																									1437	160.8	7.01
IPI00284806.8	Hypoxanthine-guanine phosphoribosyltransferase	86.70%	1	10	26	259	0.043	-0.138	0.120	-0.095	0.179	14	-0.542	-0.112	-0.373	-0.368	-0.176	15	-0.162	-0.122	-0.260	-0.048	-0.158	5	0.075	0.098	0.099	-0.047	0.045	11	0.070	0.178	0.107	0.050	0.047	39	218	24.6	6.68
IPI00123342.4	Hypoxia up-regulated protein 1	89.19%	3	36	108	1359	-0.041	0.016	-0.029	0.007	-0.009	70	0.386	0.038	0.332	0.134	0.245	65	0.091	0.016	0.073	-0.039	0.048	34	-0.355	-0.035	-0.486	0.009	-0.357	149	0.033	0.043	-0.154	-0.271	-0.105	16	999	111.1	5.19
IPI00116996.1	IgE-binding protein	86.54%	4	2	66	520							-0.306	-0.155	-0.304	0.016	-0.042	3							-0.375	-0.035	-0.513	-0.060	-0.228	134							557	62.7	9.31
IPI00475246.1	Igh protein	41.68%	3	2	16	45																									0.166	-0.062	0.207	0.258	0.184	4	463	51.0	7.03
IPI00321348.3	Immunoglobulin superfamily member 8	70.54%	3	14	46	193							-0.218	-0.311	-0.020	0.010	0.188	3													0.085	0.193	0.106	0.122	0.172	43	611	65.0	7.99
IPI00311329.1	Importin subunit alpha-1	57.43%	4	2	34	132	-0.071	-0.076	-0.186	-0.079	-0.170	3	-0.060	0.007	0.124	-0.133	0.166	12	-0.074	0.126	0.233	0.152	-0.141	5													538	60.1	5.01
IPI00124973.1	Importin subunit alpha-2	84.69%	6	12	49	610	-0.206	0.107	-0.198	0.059	-0.285	44	0.247	-0.102	0.061	-0.288	0.273	31	0.207	-0.013	0.250	0.107	0.274	33	0.064	0.076	0.006	0.041	0.115	38							529	57.9	5.68
IPI00230429.4	Importin subunit alpha-3	54.70%	2	7	30	206	-0.127	-0.039	-0.189	-0.022	-0.158	21	-0.041	0.004	-0.041	-0.019	-0.105	13	0.085	-0.010	0.091	0.021	0.007	8	0.076	0.120	0.085	0.027	0.016	10							521	57.7	4.94
IPI00129792.1	Importin subunit alpha-4	62.38%	2	8	30	239	0.195	0.073	-0.001	0.051	0.112	5	-0.160	0.026	-0.409	-0.114	-0.176	13	-0.048	0.019	0.029	0.054	-0.059	15	-0.026	-0.155	0.084	0.041	-0.048	7	-0.031	-0.253	-0.278	-0.306	-0.080	4	521	57.9	4.94
IPI00649329.3	Importin subunit alpha-7	75.93%	4	5	41	216	-0.051	0.079	-0.106	0.037	-0.068	8	-0.173	-0.089	-0.108	-0.183	0.083	14	-0.286	-0.025	-0.204	0.030	-0.117	8													536	59.9	4.94
IPI00323881.2	Importin subunit beta-1	76.60%	1	36	75	1211	-0.183	0.027	-0.134	0.013	-0.150	93	0.041	0.020	-0.056	-0.044	0.048	52	0.026	-0.039	0.023	0.040	0.041	85	-0.283	0.156	-0.286	0.037	-0.304	88	0.006	-0.025	-0.082	-0.114	-0.016	50	876	97.1	4.78
IPI00128880.3	Importin-4	62.29%	3	12	77	310	0.179	-0.082	0.088	-0.006	0.152	8	-0.091	-0.278	-0.199	-0.265	0.219	4																			1082	119.2	5.03
IPI00331444.7	Importin-7	72.64%	1	24	76	660	-0.144	-0.063	-0.184	0.000	-0.134	38	-0.109	0.003	-0.320	-0.034	-0.037	29	-0.295	-0.109	-0.188	0.099	-0.139	22	0.010	-0.091	-0.012	-0.080	0.034	38	-0.099	-0.006	-0.092	-0.154	-0.108	22	1038	119.4	4.82
IPI00130158.3	Importin-9	72.72%	6	22	76	436	-0.209	-0.128	-0.214	-0.004	-0.117	22	-0.054	0.033	0.009	0.108	0.009	23	-0.318	-0.208	-0.370	-0.059	-0.219	7	-0.500	-0.127	-0.209	0.038	-0.211	8							1041	116.0	4.81
IPI00396687.3	inactive dipeptidyl peptidase 10	70.75%	2	8	68	169																									0.128	0.027	-0.009	-0.059	0.061	17	800	91.1	6.48
IPI00225301.1	Inactive hydroxysteroid dehydrogenase-like protein 1	47.88%	1	2	17	47																									-0.029	0.033	-0.006	-0.260	0.128	5	330	36.8	8.48
IPI00128145.6	Inactive phospholipase C-like protein 1	60.86%	11	5	79	191																															1096	122.6	5.64
IPI00322431.9	Inactive phospholipase C-like protein 2	60.37%	1	5	83	229																									0.030	-0.004	0.143	0.429	-0.029	4	1128	125.7	6.92
IPI00122958.1	Induced myeloid leukemia cell differentiation protein Mcl-1 homolog	78.25%	2	5	29	65	-0.023	0.070	0.004	-0.066	-0.120	3	0.330	0.240	0.299	-0.139	0.200	3																			331	35.2	6.20
IPI00110684.1	Inorganic pyrophosphatase	94.46%	2	19	33	365	-0.142	-0.136	-0.004	0.003	-0.048	22	-0.434	-0.029	-0.288	-0.147	-0.204	39	-0.519	-0.216	-0.357	0.031	-0.231	16	0.263	0.077	0.068	-0.028	0.085	24	0.070	0.059	0.007	-0.131	-0.017	33	289	32.6	5.60
IPI00678003.1	Inosine triphosphate pyrophosphatase	92.93%	2	6	19	156	-0.163	-0.313	-0.092	-0.204	-0.119	6	-0.213	-0.383	-0.286	-0.170	-0.548	8	-0.591	-0.296	-0.246	-0.013	-0.326	3	0.545	-0.103	0.564	0.050	0.607	4	0.038	-0.063	-0.039	-0.157	0.004	21	198	21.9	5.87
IPI00970385.1	Inosine-5'-monophosphate dehydrogenase	95.95%	5	7	56	241							-0.069	0.001	-0.023	-0.160	0.009	10							0.087	0.212	0.238	0.139	0.364	3							568	60.8	7.59
IPI00323971.2	Inosine-5'-monophosphate dehydrogenase 2	83.27%	2	22	57	562	-0.127	0.003	-0.089	-0.007	-0.136	81	0.189	-0.051	0.158	-0.059	0.275	53	-0.383	-0.125	-0.383	-0.026	-0.288	17	0.051	-0.016	0.037	0.033	0.066	29							514	55.8	7.28
IPI00137400.2	Inositol 1,4,5-trisphosphate receptor type 3	78.35%	2	37	270	1267	-0.014	0.021	-0.025	0.003	0.027	39	0.835	0.139	0.871	0.234	0.682	28	0.141	0.067	0.092	-0.083	0.061	27							-0.425	-0.130	0.100	0.073	-0.150	7	2670	304.1	6.54
IPI00625248.3	inositol monophosphatase 1	68.95%	4	9	30	194	-0.053	-0.103	0.019	-0.041	0.094	21	-0.474	-0.038	-0.193	-0.260	-0.097	6	-0.798	-0.203	-0.383	0.166	-0.293	6							0.089	0.104	0.064	0.099	0.076	21	277	30.4	5.19
IPI00126255.1	Inositol monophosphatase 2	41.38%	1	2	16	23																			0.245	-0.142	0.157	-0.071	0.199	4							290	31.7	6.23
IPI00228719.1	Inositol monophosphatase 3	74.72%	1	8	33	89	-0.051	0.052	0.122	-0.048	0.087	3							0.045	0.093	0.065	-0.041	-0.130	4													356	38.6	6.47
IPI00114801.2	Inositol polyphosphate 1-phosphatase	84.09%	3	4	34	163																			-0.318	0.092	-0.360	-0.134	-0.472	3	0.163	-0.326	0.262	0.285	0.435	4	396	43.3	5.01
IPI00119886.1	Inositol-3-phosphate synthase 1	75.22%	1	6	41	220							-0.525	0.106	-0.556	0.176	-0.377	6	-0.472	-0.177	-0.390	0.207	-0.167	4	0.010	0.079	0.017	0.065	-0.074	14	-0.062	0.059	0.058	0.115	0.096	3	557	60.9	6.42
IPI00153136.1	Inositol-trisphosphate 3-kinase A	54.47%	1	5	32	71																									0.019	-0.133	-0.054	0.239	0.205	5	459	50.9	7.72
IPI00379844.5	Insulin receptor substrate 2	65.56%	1	3	85	189																			-0.180	-0.188	-0.208	-0.068	-0.198	4							1321	136.7	8.69
IPI00131056.1	Insulin-like growth factor 2 mRNA-binding protein 1	79.72%	1	15	68	416	-0.138	0.017	-0.286	0.011	-0.286	23	-0.240	-0.056	-0.130	-0.085	-0.171	32																			577	63.4	9.20
IPI00331315.9	Insulin-like growth factor 2 mRNA-binding protein 3	63.04%	1	4	53	173	-0.302	-0.120	-0.318	-0.003	-0.281	3							-0.091	0.103	-0.226	-0.162	-0.315	3	0.377	0.239	-0.007	0.077	0.300	4							579	63.5	8.87
IPI00130758.1	Integral membrane protein 2B	88.35%	1	5	22	82	-0.038	-0.269	-0.219	-0.216	-0.133	3							-0.425	0.090	-0.434	-0.255	-0.621	5													266	30.2	5.30
IPI00877221.2	integrator complex subunit 1	74.93%	1	4	180	630	-0.086	-0.001	-0.191	0.010	-0.279	5																									2222	247.8	6.34
IPI00115976.2	Integrin alpha-5	54.70%	1	13	72	265							0.268	0.088	0.158	0.158	0.094	9	0.138	0.109	0.064	0.003	-0.123	25													1053	115.0	5.95
IPI00132474.3	Integrin beta-1	85.84%	2	20	91	747	0.244	0.141	0.251	0.068	0.230	24	0.448	0.040	0.348	0.054	0.240	46	0.200	0.062	0.130	0.016	0.070	71	0.153	-0.044	0.208	-0.023	0.171	32							798	88.2	5.94
IPI00116668.2	Integrin-linked protein kinase	67.26%	2	14	41	208	0.062	-0.103	0.064	-0.056	0.017	12	-0.186	0.029	-0.189	-0.099	-0.213	21	-0.219	-0.175	-0.163	-0.028	-0.056	16													452	51.3	8.07
IPI00119033.1	Intercellular adhesion molecule 5	51.69%	2	8	46	173																									0.153	-0.003	-0.139	-0.138	-0.144	11	917	96.8	6.16
IPI00387397.3	interferon alpha responsive protein isoform a	31.30%	1	2	5	15																															131	15.3	7.31
IPI00357145.5	interferon regulatory factor 2 binding protein 2	78.07%	1	5	45	150	0.010	-0.089	-0.057	-0.209	-0.074	7																									570	59.3	8.69
**IPI00469941.2**	**Interferon regulatory factor 2-binding protein-like**	**68.26%**	**1**	**4**	**55**	**224**							0.065	-0.272	0.126	-0.044	0.076	**4**	0.024	0.016	0.126	0.122	-0.136	**4**													**775**	**80.5**	**8.24**
IPI01008307.1	interferon-activable protein 204	76.41%	6	4	62	260	-0.260	-0.123	-0.300	-0.071	-0.362	3							-0.127	-0.034	-0.213	-0.176	-0.133	6													619	69.4	8.69
IPI00133243.1	Interferon-induced transmembrane protein 3	67.15%	1	2	7	31							0.516	-0.357	0.822	0.327	0.783	3																			137	14.9	7.40
IPI00318550.5	Interleukin enhancer-binding factor 2	83.59%	1	13	32	358	-0.189	0.021	-0.056	0.031	-0.203	17	0.052	0.009	0.038	0.080	-0.010	16	0.100	0.068	0.170	-0.045	0.143	20	0.057	0.059	0.139	0.027	0.070	26	-0.076	0.033	0.075	-0.032	-0.010	12	390	43.0	5.26
IPI00752375.1	Interleukin-1 receptor antagonist protein isoform 3	60.49%	4	2	9	25	0.112	-0.262	0.187	-0.388	0.110	3																									162	18.3	6.01
IPI00120155.1	Interleukin-6 receptor subunit beta	63.90%	1	2	59	174							1.434	0.309	0.727	0.481	0.760	4																			917	102.4	5.48
IPI00123374.3	intracellular hyaluronan-binding protein 4	64.56%	2	3	32	78																									0.029	-0.044	0.135	0.003	0.087	3	412	45.9	6.84
IPI01008430.1	inverted formin-2	66.88%	4	20	96	435	-0.258	0.007	-0.208	-0.071	-0.203	18	0.394	-0.191	0.596	0.258	0.193	12	0.392	0.012	0.347	0.011	0.162	14													1271	138.3	5.22
IPI00761443.2	IQ motif and SEC7 domain-containing protein 1 isoform b	66.88%	3	10	103	340																									0.055	-0.013	0.003	0.038	-0.139	21	1099	122.6	8.47
IPI00124412.7	IQ motif containing GTPase activating protein 3	68.63%	3	9	147	476	-0.180	0.001	-0.318	0.006	-0.020	6							-0.051	-0.016	0.033	0.209	-0.168	3													1632	185.2	7.52
IPI00274222.2	Isobutyryl-CoA dehydrogenase, mitochondrial	91.53%	1	9	44	104																									-0.047	0.139	0.236	-0.218	0.245	7	413	45.0	8.13
IPI00165849.3	Isochorismatase domain-containing protein 1	92.59%	1	5	28	135	-0.125	-0.144	-0.271	-0.062	-0.150	8													-1.245	-0.390	-1.347	-0.041	-1.272	5	-0.154	0.333	0.004	0.116	0.275	9	297	32.0	7.39
IPI00757372.2	Isochorismatase domain-containing protein 2A, mitochondrial	79.61%	1	4	19	72																									-0.120	0.157	0.181	0.072	0.118	10	206	22.4	8.02
IPI00135231.2	Isocitrate dehydrogenase	87.68%	3	27	57	499	0.276	-0.138	0.276	-0.044	0.409	43	0.007	0.199	0.457	0.210	0.166	13	-0.208	-0.200	-0.127	-0.076	-0.068	18	-0.258	-0.199	-0.155	-0.122	-0.095	20	-0.053	-0.020	-0.037	-0.139	-0.045	53	422	47.5	6.93
IPI00318614.9	Isocitrate dehydrogenase [NADP], mitochondrial	94.03%	4	26	59	683	0.138	0.017	0.151	0.016	0.138	32	0.253	0.179	0.343	0.262	0.075	73	0.058	0.063	0.064	-0.007	-0.127	44	0.099	-0.044	0.115	-0.071	0.203	29	-0.044	0.083	-0.089	0.065	0.064	20	452	50.9	8.69
IPI00126635.1	isocitrate dehydrogenase 3, beta subunit	79.17%	1	15	44	385	-0.149	0.037	-0.100	0.043	-0.214	22	0.175	-0.108	0.188	-0.047	0.358	12	0.166	0.115	0.141	-0.056	0.013	18	-0.092	-0.044	-0.116	-0.029	-0.071	27	-0.047	-0.130	-0.106	-0.204	-0.133	51	384	42.2	8.60
IPI00230618.3	Isoform 1 of 1-phosphatidylinositol-3-phosphate 5-kinase	72.44%	3	4	176	495	-0.069	-0.285	-0.130	0.099	-0.094	3																									2097	236.7	6.68
IPI00118193.1	Isoform 1 of 28S ribosomal protein S18b, mitochondrial	81.50%	5	3	20	51	0.021	-0.059	0.006	-0.069	-0.001	3																									254	28.7	8.35
IPI00626237.2	Isoform 1 of 2-oxoglutarate dehydrogenase, mitochondrial	76.93%	6	36	86	909	0.099	0.059	0.080	-0.042	0.115	56	0.607	0.040	0.719	0.116	0.605	32	0.185	0.065	0.085	0.011	-0.016	48	-0.193	-0.034	-0.218	0.013	-0.186	74	0.020	-0.053	-0.014	-0.056	-0.077	94	1023	116.4	6.83
IPI00798614.1	Isoform 1 of 39S ribosomal protein L15, mitochondrial	70.51%	2	4	32	72							0.062	-0.076	0.085	0.006	0.204	6							-0.193	0.136	0.286	-0.019	0.358	3							295	33.5	10.07
IPI00133965.2	Isoform 1 of 39S ribosomal protein L21, mitochondrial	65.07%	2	4	17	53	-0.057	0.116	-0.147	-0.083	-0.004	5							-0.059	0.087	-0.035	-0.191	-0.189	3													209	23.4	9.99
IPI00607969.2	Isoform 1 of 39S ribosomal protein L44, mitochondrial	63.06%	2	3	24	60	-0.317	0.006	-0.269	-0.020	-0.229	3																									333	37.5	8.51
IPI00117754.1	Isoform 1 of 45 kDa calcium-binding protein	56.23%	2	4	16	47							-0.004	-0.223	-0.047	0.007	-0.038	6																			361	42.0	4.96
IPI00407499.1	Isoform 1 of 4-aminobutyrate aminotransferase, mitochondrial	92.80%	9	26	54	400																									0.016	0.120	0.091	0.064	0.059	130	500	56.4	8.09
IPI00120046.1	Isoform 1 of 5'-3' exoribonuclease 2	65.83%	2	19	71	467	-0.223	0.003	-0.157	0.026	-0.119	21	-0.131	0.131	-0.199	0.010	-0.246	25	0.165	0.052	0.192	0.041	-0.020	14	0.050	-0.100	0.139	-0.116	0.051	22							951	108.6	7.59
IPI00308885.6	Isoform 1 of 60 kDa heat shock protein, mitochondrial	92.84%	4	47	74	3023	-0.141	0.057	-0.183	0.023	-0.216	229	-0.160	-0.017	-0.201	-0.032	-0.133	161	0.023	0.106	-0.037	-0.044	-0.165	188	0.089	-0.016	0.068	-0.039	0.114	259	0.102	-0.042	-0.048	-0.141	0.003	155	573	60.9	6.18
IPI00881976.1	Isoform 1 of 60S ribosomal export protein NMD3	79.13%	1	4	42	108							-0.478	-0.193	-0.474	-0.320	-0.415	3																			503	57.6	7.17
IPI00225209.1	Isoform 1 of Acetolactate synthase-like protein	63.13%	3	6	45	140	0.213	-0.019	-0.072	-0.013	0.106	5																									632	68.1	8.69
IPI00130016.1	Isoform 1 of Acidic leucine-rich nuclear phosphoprotein 32 family member I	66.15%	7	9	24	264	0.139	-0.246	0.174	-0.039	0.127	22	-0.366	0.163	-0.184	0.205	-0.407	24	-0.173	-0.169	-0.219	0.154	-0.065	9	0.679	0.047	0.639	-0.029	0.595	32							260	29.6	3.88
IPI00467530.3	Isoform 1 of Actin-binding LIM protein 1	62.02%	18	9	73	302	-0.038	0.089	-0.026	-0.001	0.033	7	0.007	0.072	-0.253	0.088	0.027	5	-0.150	0.113	-0.146	-0.056	-0.213	4							-0.492	-0.417	0.055	0.041	-0.488	4	861	96.7	8.63
IPI00380734.2	Isoform 1 of Actin-related protein 3B	68.18%	2	4	36	293	0.112	-0.012	0.175	0.092	0.115	17	0.088	-0.026	-0.080	-0.146	0.119	8	-0.083	-0.030	-0.042	0.104	0.073	8	0.104	0.178	0.383	0.190	0.357	5	-0.025	-0.180	-0.154	-0.189	-0.003	13	418	47.5	6.02
IPI00135240.2	Isoform 1 of Activating signal cointegrator 1	76.94%	2	1	52	137	0.098	-0.053	-0.318	0.065	0.167	3																									581	66.2	7.55
IPI00273023.5	Isoform 1 of Activator of 90 kDa heat shock protein ATPase homolog 2	72.21%	5	1	36	94													-0.056	-0.168	-0.149	0.054	-0.079	3	-0.336	-0.010	-0.102	-0.004	-0.077	4							331	37.6	6.44
IPI00395178.1	Isoform 1 of Active breakpoint cluster region-related protein	71.13%	5	10	88	333	-0.240	-0.032	-0.144	-0.003	-0.123	3							-0.218	-0.050	0.051	0.116	-0.180	3							0.030	0.103	0.023	0.000	-0.014	25	859	97.6	6.58
IPI00387245.1	Isoform 1 of Acylamino-acid-releasing enzyme	71.17%	2	14	60	295	0.072	-0.007	0.179	0.128	0.095	10	0.062	-0.016	0.214	0.122	0.185	10							-0.116	0.158	-0.071	-0.023	-0.180	7	-0.248	0.013	-0.117	-0.181	-0.245	6	732	81.5	5.53
IPI00132083.2	Isoform 1 of Acyl-CoA-binding domain-containing protein 6	55.32%	1	2	10	31																			-0.186	0.007	-0.269	0.033	0.132	5							282	30.9	5.11
IPI00406375.1	Isoform 1 of Acyl-coenzyme A thioesterase 6	60.14%	8	1	28	71																			-0.031	-0.152	-0.023	-0.059	0.092	3	0.328	0.123	-0.029	0.140	0.208	4	419	46.7	7.02
IPI00113606.2	Isoform 1 of Acylglycerol kinase, mitochondrial	64.61%	2	9	27	126							0.163	-0.255	-0.162	-0.141	0.301	3							0.087	-0.085	0.139	-0.017	0.070	3	-0.094	0.066	0.006	-0.032	0.062	22	421	46.9	8.40
IPI00130018.1	Isoform 1 of Acyl-protein thioesterase 1	95.22%	5	6	18	195	-0.069	0.048	0.026	-0.019	-0.057	7	-0.621	-0.191	-0.127	-0.180	-0.048	10							-0.001	-0.048	0.031	0.011	-0.047	4	-0.012	0.189	0.038	-0.079	-0.013	10	230	24.7	6.62
IPI00128209.3	Isoform 1 of Adenylate kinase isoenzyme 1	92.78%	3	14	29	218	-0.589	-0.022	-0.377	-0.051	-0.295	6	-0.476	-0.138	-0.295	-0.066	-0.035	8	-0.606	-0.294	-0.533	-0.013	-0.313	9	0.009	0.139	-0.034	-0.013	0.019	8	0.047	0.040	0.047	0.006	0.026	49	194	21.5	5.81
IPI00116072.7	Isoform 1 of Adenylate kinase isoenzyme 5	69.93%	2	4	51	185																									-0.091	-0.269	-0.198	-0.286	-0.203	8	562	63.3	5.29
IPI00123190.1	Isoform 1 of Adenylosuccinate synthetase isozyme 1	76.81%	2	10	49	253																									-0.194	0.116	0.041	0.169	-0.044	21	457	50.2	8.43
IPI00853902.1	Isoform 1 of Afadin	79.17%	5	1	176	851	0.029	0.044	0.102	0.040	0.088	34	-0.050	0.020	-0.029	0.009	-0.173	48							-0.117	0.045	-0.076	-0.051	-0.203	14	0.463	-0.051	0.246	0.050	0.551	3	1805	204.4	6.24
IPI00468514.1	Isoform 1 of AFG3-like protein 1	79.09%	2	1	72	240																									-0.017	-0.091	-0.163	-0.105	-0.112	5	789	87.0	8.85
IPI00123709.1	Isoform 1 of A-kinase anchor protein 12	85.51%	2	26	133	931							-0.260	-0.050	-0.288	-0.010	-0.245	35	0.353	-0.064	0.349	0.007	0.534	25	-0.279	0.033	-0.364	0.062	-0.281	134	-0.050	0.079	0.085	0.220	0.183	6	1684	180.6	4.44
IPI00136000.1	Isoform 1 of Alpha-adducin	75.65%	8	21	60	481	0.124	-0.003	0.273	-0.041	0.321	18	0.265	0.073	0.373	0.270	0.058	19	0.388	-0.086	0.458	0.320	0.338	4							-0.023	-0.017	-0.023	-0.069	-0.012	67	735	80.6	5.90
IPI00230084.5	Isoform 1 of Alpha-aminoadipic semialdehyde dehydrogenase	66.05%	6	11	40	325	-0.173	-0.204	-0.178	-0.155	-0.134	3	-0.069	0.205	0.124	0.048	0.055	13													-0.068	0.034	-0.045	-0.053	-0.065	21	539	58.8	7.47
IPI00409045.2	Isoform 1 of Alpha-endosulfine	87.60%	3	7	20	142							-0.358	-0.092	-0.248	-0.068	-0.257	11	-0.063	-0.034	0.070	0.310	-0.034	5	0.310	-0.068	-0.097	-0.007	-0.136	3	0.132	-0.032	0.004	0.062	0.069	15	121	13.3	7.24
IPI00342749.3	Isoform 1 of Alpha-taxilin	86.10%	8	9	62	239	0.148	0.034	0.003	-0.076	0.327	6	0.115	-0.127	0.160	0.057	0.190	6	-0.299	-0.029	-0.068	-0.066	-0.130	5													554	62.3	6.74
IPI00844650.1	Isoform 1 of Alpha-type platelet-derived growth factor receptor	62.99%	9	1	83	286																									-0.199	-0.116	-0.155	-0.128	-0.022	4	1089	122.6	5.15
IPI00387261.4	Isoform 1 of Amyloid beta A4 precursor protein-binding family B member 1	50.42%	2	3	39	85	0.027	0.006	-0.048	0.128	0.122	3																									710	77.3	5.00
IPI00329843.5	Isoform 1 of Ankyrin repeat and FYVE domain-containing protein 1	79.21%	2	14	103	510	-0.144	-0.142	-0.313	-0.039	-0.281	5	0.240	-0.209	-0.062	-0.257	0.052	15	0.322	0.115	0.152	0.144	0.156	3	-0.448	0.403	-0.201	-0.001	-0.163	3							1169	128.6	5.91
IPI00465535.1	Isoform 1 of Ankyrin repeat domain-containing protein 17	62.74%	5	11	161	655	0.076	0.091	-0.065	-0.082	0.076	8	0.166	0.315	-0.272	-0.111	-0.109	4																			2603	274.0	6.52
IPI00468236.3	Isoform 1 of Anoctamin-6	59.71%	2	11	57	185	0.640	0.192	0.373	-0.041	0.354	4	0.011	0.219	-0.257	-0.026	0.238	6	0.027	0.033	0.001	-0.038	0.007	15													911	106.2	6.76
IPI00119689.1	Isoform 1 of AP-2 complex subunit beta	76.20%	8	31	106	1651	0.073	-0.013	-0.023	0.011	0.016	77	0.274	0.037	0.251	0.061	0.157	76	0.080	-0.013	0.054	-0.017	0.031	50	-0.062	0.087	-0.109	0.038	-0.105	79	0.179	-0.088	-0.092	-0.086	-0.066	203	937	104.5	5.38
IPI00349020.6	Isoform 1 of Arf-GAP with GTPase, ANK repeat and PH domain-containing p	66.86%	2	8	109	260																									-0.003	-0.099	-0.042	-0.062	-0.068	12	1186	124.4	9.89
IPI00128319.1	Isoform 1 of Arf-GAP with GTPase, ANK repeat and PH domain-containing p	72.53%	4	4	73	234																									0.271	-0.165	0.296	0.496	0.696	7	910	97.9	7.75
IPI00652831.2	Isoform 1 of Arginine and glutamate-rich protein 1	65.31%	2	3	38	128							-0.383	-0.299	-0.150	-0.048	-0.139	4	0.107	0.079	0.119	-0.112	0.181	6	0.099	0.100	0.195	0.072	0.131	3							271	32.9	10.36
IPI00403909.2	Isoform 1 of Armadillo repeat-containing protein 6	52.35%	3	6	23	105							0.457	-0.001	0.141	-0.206	0.166	3							-0.462	0.040	-0.208	-0.003	-0.028	11							468	50.7	6.00
IPI00652358.5	Isoform 1 of Arylsulfatase B	39.14%	1	2	20	42																									0.010	0.183	0.118	0.215	0.297	3	534	59.6	7.24
IPI00121013.1	Isoform 1 of Astrocytic phosphoprotein PEA-15	88.46%	2	8	15	131	0.392	-0.085	0.514	0.080	0.423	6	-0.708	-0.150	-0.615	-0.309	-0.529	4	0.007	-0.048	-0.186	0.096	-0.102	3							0.134	0.023	0.061	0.003	0.084	26	130	15.0	5.02
IPI00474125.3	Isoform 1 of Atlastin-2	67.24%	4	6	44	244	0.404	-0.092	0.258	0.114	0.185	3							0.085	0.039	-0.042	0.162	-0.003	4	-0.368	-0.171	-0.429	-0.198	-0.390	8	0.017	-0.238	0.096	0.432	0.073	5	583	66.2	5.43
IPI00458393.3	Isoform 1 of Atlastin-3	65.43%	2	16	41	255	0.246	0.134	0.225	-0.014	0.349	23	0.214	-0.010	0.438	0.043	0.288	21	0.507	0.129	0.404	0.172	0.453	8													541	60.5	6.10
IPI00126913.5	Isoform 1 of ATPase family AAA domain-containing protein 3	86.13%	3	16	77	355	-0.236	0.161	-0.204	0.094	-0.229	8	0.315	0.141	0.255	0.081	0.102	8	0.048	0.035	-0.020	0.034	-0.109	12	-0.224	-0.048	-0.221	0.020	-0.204	26	0.059	-0.007	-0.097	-0.026	-0.152	11	591	66.7	9.29
IPI00123878.4	Isoform 1 of ATP-dependent RNA helicase DDX39A	84.78%	6	14	55	657	-0.012	0.099	-0.041	0.001	-0.142	37	-0.162	0.011	-0.213	-0.056	-0.069	62	0.102	0.042	0.061	0.013	0.013	52	0.124	0.034	0.205	-0.023	0.096	57							427	49.0	5.68
IPI00329839.7	Isoform 1 of ATP-dependent RNA helicase DDX42	72.44%	2	16	68	262	-0.025	0.036	0.073	0.017	0.077	5	0.213	0.011	0.224	0.143	0.087	12	0.004	0.039	0.007	-0.091	0.036	3	-0.134	-0.020	-0.017	0.000	-0.080	8							929	101.9	6.98
IPI00310293.3	Isoform 1 of BAG family molecular chaperone regulator 1	82.82%	2	2	41	131							0.153	-0.047	-0.300	-0.141	-0.336	6																			355	39.7	8.46
IPI00830613.1	Isoform 1 of Band 4.1-like protein 1	81.11%	3	2	90	344	-0.089	0.212	0.034	0.116	-0.072	5	-0.448	0.236	-0.262	0.365	-0.450	9	0.224	0.020	0.111	-0.009	0.134	3							0.009	0.084	0.024	0.016	0.004	60	879	98.3	5.62
IPI00415385.1	Isoform 1 of Bcl-2-associated transcription factor 1	80.09%	5	28	139	714	-0.010	0.038	0.077	-0.038	0.036	27	-0.258	0.091	-0.071	0.149	-0.196	55	0.300	0.188	0.222	0.003	0.197	30	0.037	-0.042	0.099	-0.037	-0.025	33	-0.271	0.383	0.233	-0.119	-0.170	4	919	105.9	9.99
IPI00323122.1	Isoform 1 of Beta-adducin	65.79%	3	18	62	241																									0.017	0.041	-0.006	-0.079	0.023	58	725	80.6	6.21
IPI00454118.1	Isoform 1 of Bifunctional polynucleotide phosphatase/kinase	78.35%	4	5	39	76	-0.392	-0.201	-0.154	0.103	-0.176	4																									522	57.2	7.90
IPI00222731.2	Isoform 1 of Brain-specific angiogenesis inhibitor 1-associated protein 2	97.38%	5	25	77	474	0.202	0.187	0.037	0.190	0.088	10	-0.175	0.013	-0.204	0.112	-0.193	19	-0.231	0.067	-0.208	-0.146	-0.031	9							-0.009	-0.175	-0.255	-0.216	-0.038	54	535	59.2	9.04
IPI00336713.1	Isoform 1 of Breast carcinoma-amplified sequence 3 homolog	56.90%	4	2	50	111																									0.582	0.316	0.345	-0.013	-0.088	3	928	101.0	6.70
IPI00660178.4	Isoform 1 of Bromodomain and WD repeat-containing protein 3	58.48%	2	2	132	388	-0.206	-0.324	0.066	-0.089	-0.106	3																									1799	202.8	7.42
IPI00410791.1	Isoform 1 of Bromodomain-containing protein 3	62.12%	5	5	58	169	0.238	0.249	0.300	0.161	0.273	3	0.068	0.143	0.139	-0.199	0.111	4																			726	79.7	9.36
IPI00169704.3	Isoform 1 of Calcineurin-like phosphoesterase domain-containing protein 1	64.10%	6	2	22	60							-0.165	-0.003	-0.191	-0.246	0.175	3																			312	35.2	5.34
IPI00655156.3	Isoform 1 of Calcium uniporter protein, mitochondrial	71.14%	2	6	25	95	0.020	0.123	0.054	0.092	-0.094	5																			-0.026	0.014	0.050	0.103	-0.028	10	350	39.7	8.56
IPI00404152.2	Isoform 1 of Calcium/calmodulin-dependent protein kinase kinase 2	66.50%	6	3	41	152																									-0.370	-0.041	-0.624	0.138	-0.208	3	588	64.6	5.90
IPI00112584.3	Isoform 1 of Calcium/calmodulin-dependent protein kinase type II subunit c	78.76%	11	1	60	591	0.040	-0.060	0.038	-0.026	-0.007	26	-0.245	-0.116	-0.385	-0.105	-0.302	18	0.138	0.078	0.044	0.104	0.127	28	-0.136	0.110	-0.170	0.051	-0.028	14	0.021	0.033	0.001	-0.079	-0.034	80	499	56.3	7.25
IPI00124695.1	Isoform 1 of Calcium/calmodulin-dependent protein kinase type II subunit g	64.65%	4	9	57	502	-0.139	-0.158	-0.103	-0.155	-0.152	11	-0.218	-0.162	-0.400	-0.203	-0.228	11	0.128	0.078	0.217	0.096	0.157	21							0.045	0.124	0.069	0.094	0.066	66	529	59.6	7.58
IPI00668903.2	Isoform 1 of Calcium-dependent secretion activator 1	80.07%	3	2	125	621																									0.014	0.024	-0.003	-0.026	0.004	108	1355	153.0	5.74
IPI00409176.1	Isoform 1 of Calpastatin	73.98%	9	21	65	407	0.530	0.041	0.617	-0.013	0.544	21	0.207	-0.019	0.230	-0.020	0.092	36	-0.031	-0.171	0.119	-0.082	0.179	16	0.047	0.077	0.020	-0.009	0.061	11							788	84.9	5.52
IPI00230005.3	Isoform 1 of cAMP-dependent protein kinase catalytic subunit alpha	77.49%	2	12	42	426	0.064	0.059	0.161	0.048	0.157	15	-0.056	-0.053	0.088	-0.012	-0.039	38	-0.133	-0.110	-0.262	0.061	0.003	6	0.073	0.029	0.038	0.044	-0.029	21	0.006	0.143	0.076	0.079	0.058	34	351	40.5	8.79
IPI00263822.7	Isoform 1 of cAMP-dependent protein kinase catalytic subunit beta	78.06%	4	7	37	248	-0.042	-0.038	-0.010	0.031	0.019	11	-0.085	-0.004	-0.069	-0.119	-0.072	16	-0.025	-0.050	-0.089	0.076	0.003	4	0.289	0.020	0.038	0.112	-0.012	11	-0.003	0.120	0.066	0.091	-0.029	33	351	40.7	8.56
IPI00129525.1	Isoform 1 of cAMP-regulated phosphoprotein 21	45.35%	12	2	45	101																									-0.924	-0.381	-0.139	-0.079	-0.226	3	807	88.5	7.39
IPI00467854.1	Isoform 1 of CAP-Gly domain-containing linker protein 2	72.87%	5	6	107	361																									-0.020	-0.072	-0.152	-0.260	0.026	11	1047	115.8	6.48
IPI00411109.4	Isoform 1 of Caskin-1	62.47%	6	8	103	463																									-0.178	0.295	-0.077	0.217	0.094	16	1431	150.4	9.17
IPI00403823.1	Isoform 1 of Catenin delta-1	89.45%	13	1	114	698	0.276	0.038	0.267	0.024	0.252	68	0.306	-0.013	0.288	0.014	0.104	23	0.081	0.061	0.024	-0.039	0.006	57	0.513	-0.003	0.269	-0.125	0.181	4							938	104.9	6.87
IPI00187402.1	Isoform 1 of CCA tRNA nucleotidyltransferase 1, mitochondrial	85.71%	2	9	47	254	0.087	0.172	0.036	0.072	-0.032	10	-0.218	-0.017	-0.117	-0.047	-0.053	20							-0.054	-0.114	-0.147	-0.072	-0.219	21							434	49.9	8.56
IPI00673465.2	Isoform 1 of CCR4-NOT transcription complex subunit 1	74.15%	6	30	207	1116	0.031	0.072	-0.069	0.019	0.050	24	-0.063	-0.037	-0.246	0.079	-0.163	20	-0.031	-0.019	-0.147	-0.072	-0.050	13	-0.032	0.069	0.010	0.057	-0.013	25							2375	266.6	7.11
IPI00221683.3	Isoform 1 of CCR4-NOT transcription complex subunit 10	85.89%	6	6	72	248	-0.322	-0.300	-0.077	-0.181	-0.144	4																									744	81.8	7.68
IPI00120193.1	Isoform 1 of Cell division control protein 42 homolog	76.44%	7	2	18	275	0.415	-0.028	0.256	0.045	0.368	23	0.006	0.222	0.190	0.276	0.066	18	-0.035	-0.056	-0.114	0.013	-0.119	25	0.004	0.076	-0.083	0.088	-0.069	9	-0.017	0.116	0.016	0.029	-0.032	33	191	21.3	6.04
IPI00135207.3	Isoform 1 of Cell division cycle and apoptosis regulator protein 1	61.34%	3	9	87	310	-0.130	-0.010	-0.155	-0.022	-0.171	11	0.534	0.255	0.545	0.271	0.185	6	0.233	0.106	0.127	0.271	0.091	11	-0.111	0.061	-0.086	0.073	-0.080	6							1146	132.0	5.76
IPI00461969.2	Isoform 1 of Cell division cycle protein 123 homolog	38.39%	1	3	16	57	-0.226	0.000	-0.253	-0.020	-0.047	3																									336	38.8	4.67
IPI00330767.1	Isoform 1 of Cellular nucleic acid-binding protein	79.78%	2	1	19	55	-0.100	-0.032	0.089	0.044	-0.178	3	0.020	0.084	-0.336	-0.515	-0.035	11	0.054	0.009	0.024	-0.038	-0.050	3													178	19.6	7.58
IPI00318428.4	Isoform 1 of Centromere protein V	64.29%	3	5	20	114																			-0.272	-0.054	-0.290	0.023	-0.189	11	-0.116	0.036	0.130	0.124	0.124	13	252	27.5	9.79
IPI00667973.3	Isoform 1 of Centrosomal protein of 170 kDa	77.64%	3	18	179	630							-0.162	-0.127	-0.105	-0.035	-0.111	12	0.111	0.093	0.139	0.229	0.158	20	0.205	0.038	0.381	-0.059	0.341	5	-0.260	-0.051	0.079	-0.265	-0.241	5	1588	174.9	7.17
IPI00466518.2	Isoform 1 of Centrosomal protein of 55 kDa	83.98%	3	8	71	260							0.030	-0.120	0.051	-0.042	-0.106	6	0.092	-0.004	0.200	0.041	0.055	5													462	53.9	7.94
IPI00109214.1	Isoform 1 of Ceramide synthase 5	57.97%	2	5	25	131	0.081	-0.001	-0.041	-0.020	0.068	8	0.632	0.112	0.135	0.072	-0.013	4							0.227	-0.034	0.087	-0.042	0.098	3							414	48.1	8.07
IPI00648633.2	Isoform 1 of Choline transporter-like protein 1	55.28%	6	8	42	107																									-0.125	-0.017	-0.004	-0.012	0.062	11	653	73.0	8.75
IPI00128915.4	Isoform 1 of Chondroitin sulfate proteoglycan 4	40.65%	3	8	81	252							1.054	0.309	0.584	-0.155	0.515	10																			2327	252.3	5.44
IPI00228978.2	Isoform 1 of Clathrin light chain B	53.28%	4	11	24	151							0.153	0.031	0.252	-0.219	0.484	12													0.172	-0.100	-0.053	-0.068	-0.076	34	229	25.2	4.63
IPI00225249.2	Isoform 1 of Cleavage and polyadenylation specificity factor subunit 7	63.69%	3	6	42	197	-0.047	0.037	0.021	-0.112	-0.292	4	-0.007	-0.014	0.336	-0.458	0.397	5	0.000		0.017	0.095	0.091	7													471	52.0	8.00
IPI00457575.2	Isoform 1 of Cleavage stimulation factor subunit 2	66.72%	6	6	53	279	0.001	0.044	-0.117	-0.060	-0.063	4	-0.204	-0.167	-0.062	-0.184	-0.236	10	0.162	0.045	0.034	-0.056	0.016	6	-0.157	0.079	-0.243	-0.045	-0.074	9							580	61.3	6.83
IPI00869359.1	Isoform 1 of Coiled-coil domain-containing protein 132	76.66%	5	6	93	275	-0.007	-0.038	-0.059	-0.092	-0.257	3																			0.140	0.203	0.189	0.143	0.126	7	964	111.1	6.07
IPI00406830.5	Isoform 1 of Coiled-coil domain-containing protein 18	89.42%	1	4	195	886																			0.374	0.238	0.333	0.231	-0.003	4							1455	169.6	5.49
IPI00113099.4	Isoform 1 of Coiled-coil domain-containing protein 50	58.69%	4	3	26	104													-0.031	0.065	0.040	-0.158	0.073	5							-0.392	-0.144	-0.484	0.000	-0.274	5	305	35.3	6.89
IPI00110708.3	Isoform 1 of Coiled-coil domain-containing protein 51	92.12%	2	2	50	175	0.202	0.183	0.104	0.144	0.249	3																									406	45.1	8.09
IPI00329872.1	Isoform 1 of Collagen alpha-1(I) chain	71.23%	3	37	88	385							0.640	0.103	0.475	0.135	0.274	72																			1453	137.9	5.85
IPI00138061.1	Isoform 1 of Complement regulatory protein Crry	60.04%	4	3	24	86							1.454	-0.074	0.897	-0.257	0.297	3																			483	53.7	6.65
IPI00856653.1	Isoform 1 of Condensin-2 complex subunit H2	61.12%	3	2	34	109	0.089	0.233	-0.306	0.036	-0.016	3																									607	68.9	4.67
IPI00120513.3	Isoform 1 of COP9 signalosome complex subunit 2	93.68%	3	23	60	385	-0.141	-0.069	-0.162	-0.041	-0.094	28	-0.039	0.107	-0.095	-0.045	-0.083	16	-0.240	-0.151	-0.260	0.030	-0.065	8	0.107	0.165	0.193	0.055	0.051	13	0.019	-0.013	-0.013	0.069	-0.025	26	443	51.6	5.53
IPI00762331.2	Isoform 1 of Cordon-bleu protein-like 1	73.53%	10	22	115	563	-0.034	-0.013	0.083	-0.059	0.030	38	0.100	-0.016	-0.076	-0.065	-0.100	29																			1273	137.3	8.16
IPI00270376.6	Isoform 1 of Coxsackievirus and adenovirus receptor homolog	74.25%	3	4	28	100	-0.122	0.026	-0.224	-0.050	-0.191	14																									365	39.9	6.96
IPI00112017.5	Isoform 1 of CTP synthase 2	62.29%	4	3	48	152							-0.048	0.033	-0.311	0.048	0.011	5																			586	65.5	6.49
IPI00387216.1	Isoform 1 of Cullin-2	84.16%	4	14	81	362	-0.183	0.026	-0.112	-0.034	-0.025	10	-0.204	-0.255	-0.226	0.011	-0.194	3	-0.173	-0.099	0.356	0.124	0.333	6	-0.152	0.031	-0.163	-0.023	-0.175	11	-0.014	-0.105	-0.103	0.011	0.045	10	745	86.8	7.01
IPI00224689.3	Isoform 1 of Cullin-4B	87.32%	3	19	111	586	-0.094	0.047	-0.063	0.023	-0.147	14	0.084	-0.019	0.099	0.019	0.027	36	0.126	0.000	0.104	-0.017	0.242	8							0.194	-0.031	0.103	0.081	-0.080	9	970	110.6	8.37
IPI00276112.4	Isoform 1 of CWF19-like protein 1	60.15%	2	5	40	99	-0.333	-0.119	0.092	-0.127	-0.037	3	0.483	0.016	0.513	-0.054	0.313	3																			537	60.2	7.14
IPI00119924.1	Isoform 1 of Cyclic AMP-responsive element-binding protein 1	35.19%	5	2	14	59	-0.100	-0.241	-0.059	-0.142	-0.034	5	-0.035	0.033	0.029	-0.023	-0.103	4																			341	36.7	5.57
IPI00114953.1	Isoform 1 of Cyclin-dependent kinase 9	71.77%	46	3	36	158	-0.131	0.149	-0.194	0.212	-0.274	5																									372	42.7	8.79
IPI00308149.1	Isoform 1 of Cyclin-Y	69.21%	4	3	25	54																									0.223	0.108	0.076	-0.012	-0.041	8	341	39.4	7.20
IPI00331422.4	Isoform 1 of Cysteinyl-tRNA synthetase, cytoplasmic	83.03%	2	3	98	610	-0.089	0.017	-0.017	0.010	-0.065	51	0.040	0.112	0.087	0.136	0.120	54	-0.327	-0.233	-0.229	-0.025	-0.106	13	-0.357	-0.029	-0.292	-0.009	-0.279	23							831	94.8	6.76
IPI00132728.2	Isoform 1 of Cytochrome c1, heme protein, mitochondrial	94.46%	2	11	35	386	-0.042	-0.060	-0.133	-0.079	-0.056	18	-0.142	0.001	0.092	-0.037	0.149	8	-0.114	0.038	-0.168	-0.065	-0.253	14	0.187	-0.042	0.194	-0.037	0.276	35	0.130	-0.032	0.048	-0.109	0.024	39	325	35.3	9.16
IPI00330476.3	Isoform 1 of Cytoplasmic FMR1-interacting protein 1	73.66%	4	14	135	679	0.089	-0.017	0.003	0.045	-0.083	21	0.124	0.115	0.099	0.215	0.047	30	-0.122	-0.168	-0.120	-0.056	0.011	8	-0.066	-0.028	-0.141	-0.022	0.031	10	-0.006	-0.109	-0.032	-0.072	-0.111	41	1253	145.1	6.90
IPI00337930.4	Isoform 1 of Cytoskeleton-associated protein 5	87.06%	3	49	228	1157	-0.004	-0.074	-0.042	-0.025	0.054	21	0.094	0.065	-0.054	0.091	0.006	39	0.099	-0.011	0.033	-0.032	0.064	36	0.166	0.004	0.124	0.016	0.114	30	-0.186	0.009	-0.054	-0.065	-0.142	19	2032	225.5	7.96
IPI00331436.4	Isoform 1 of Cytosol aminopeptidase	92.87%	2	23	58	560	-0.048	-0.059	0.108	0.034	-0.026	25	0.059	0.033	0.124	-0.088	0.084	53	0.118	-0.069	0.020	0.157	0.114	16	0.382	0.014	0.370	0.004	0.439	35	0.001	0.081	-0.001	-0.037	0.062	29	519	56.1	7.72
IPI00223154.2	Isoform 1 of Dehydrogenase/reductase SDR family member 13	71.01%	2	1	32	68							-0.044	-0.088	0.009	-0.006	-0.054	5							-0.134	-0.074	-0.351	-0.071	-0.085	3							376	40.7	7.93
IPI00114878.1	Isoform 1 of Dehydrogenase/reductase SDR family member 7B	73.37%	2	6	33	121																			-0.243	-0.088	-0.379	-0.026	-0.213	7							323	35.0	9.63
IPI00377609.2	Isoform 1 of Deubiquitinating protein VCIP135	77.13%	2	6	124	296																									0.026	0.076	-0.204	-0.047	0.158	3	1220	134.4	7.17
IPI00134809.2	Isoform 1 of Dihydrolipoyllysine-residue succinyltransferase component of 2	79.07%	2	11	49	551	-0.028	0.115	-0.103	-0.037	-0.048	34	0.207	-0.017	0.285	0.072	0.318	12	0.345	0.072	0.309	0.062	0.312	23	0.075	0.014	0.095	0.011	0.106	29	0.100	0.102	0.055	0.038	0.106	37	454	49.0	8.95
IPI00462461.2	Isoform 1 of Dipeptidyl peptidase 9	41.76%	3	4	32	74																			-0.204	0.024	-0.037	-0.001	0.031	4							862	97.9	6.65
IPI00331314.6	Isoform 1 of Disco-interacting protein 2 homolog B	67.34%	3	3	119	373																									-0.415	-0.342	-0.120	-0.116	-0.060	3	1574	171.0	8.09
IPI00408668.2	Isoform 1 of Disks large homolog 1	72.71%	4	1	69	306	0.019	0.118	-0.007	0.120	-0.035	7	0.376	0.143	0.419	0.363	0.480	12													0.029	0.126	0.065	0.079	0.140	29	905	100.1	5.80
IPI00853932.1	Isoform 1 of DmX-like protein 2	62.76%	4	39	194	708																									0.118	-0.162	-0.108	-0.062	-0.085	80	3032	338.0	6.42
IPI00131694.5	Isoform 1 of DNA (cytosine-5)-methyltransferase 3A	69.49%	4	2	71	169																			-0.047	-0.199	0.062	0.075	-0.037	3							908	101.6	6.65
IPI00330591.1	Isoform 1 of DNA-binding protein A	78.12%	6	2	37	315	-0.105	-0.109	-0.095	-0.117	-0.167	23	0.193	0.040	0.050	0.026	-0.031	19	-0.053	0.050	0.102	-0.056	-0.007	29	0.280	0.225	0.233	0.019	0.197	23							361	38.8	9.69
IPI00347634.2	Isoform 1 of DnaJ homolog subfamily A member 3, mitochondrial	77.92%	2	3	43	154							-0.302	-0.340	-0.069	-0.189	-0.392	9							-0.188	-0.130	-0.193	-0.133	-0.082	7							480	52.4	9.22
**IPI00118853.1**	**Isoform 1 of Double-strand break repair protein MRE11A**	**57.37%**	**5**	**4**	**60**	**123**	-0.117	-0.133	-0.219	0.141	-0.066	**4**																									**706**	**80.2**	**6.01**
IPI00133713.5	Isoform 1 of D-tyrosyl-tRNA(Tyr) deacylase 1	60.77%	2	3	16	50																									-1.006	-0.025	-0.655	-0.214	-0.531	4	209	23.4	7.87
IPI00228989.2	Isoform 1 of Dual specificity mitogen-activated protein kinase kinase 7	66.92%	8	2	42	97																			0.310	0.088	0.300	0.099	0.308	3							535	59.3	9.11
IPI00556688.1	Isoform 1 of Dynactin subunit 4	76.66%	2	11	41	271	-0.201	0.031	-0.012	-0.045	-0.109	6	-0.238	0.111	-0.032	0.503	0.057	7							-0.272	0.112	-0.141	0.011	-0.149	8	0.007	0.059	-0.094	-0.091	0.068	10	467	53.0	7.72
IPI00556723.1	Isoform 1 of Dynamin-1-like protein	96.50%	3	2	96	652	-0.349	-0.117	-0.324	-0.028	-0.068	4	-0.060	0.001	-0.028	-0.037	0.044	16	-0.171	-0.194	-0.306	-0.276	-0.170	4	-0.097	0.068	-0.028	-0.071	-0.082	32	-0.014	-0.109	-0.120	-0.181	-0.102	99	742	82.6	7.05
IPI00227432.7	Isoform 1 of Dynamin-3	85.40%	8	15	109	573							0.429	0.193	0.791	0.344	0.441	4							-0.116	0.398	0.269	0.149	0.103	5	0.026	0.036	0.010	-0.020	-0.026	84	863	97.1	8.35
IPI00380766.5	Isoform 1 of E3 ubiquitin-protein ligase BRE1A	82.32%	4	11	123	381	-0.025	-0.094	-0.039	0.006	-0.146	8	-0.346	-0.095	-0.292	-0.245	-0.120	10																			973	113.5	5.98
IPI00380162.3	Isoform 1 of E3 ubiquitin-protein ligase Itchy	64.12%	6	2	63	162							-1.029	0.126	-0.927	-0.089	-0.718	4																			864	98.9	6.28
IPI00395040.1	Isoform 1 of E3 ubiquitin-protein ligase KCMF1	58.27%	2	5	27	227	-0.315	-0.116	0.292	0.384	0.274	7													-0.141	-0.100	-0.360	-0.016	-0.095	11							381	41.8	5.76
IPI00649115.1	Isoform 1 of E3 ubiquitin-protein ligase NEDD4-like	59.36%	6	6	61	193	-0.147	-0.128	-0.100	0.016	-0.029	6	0.009	-0.001	-0.216	-0.154	0.296	3	0.267	0.216	0.006	0.288	0.124	8													1004	115.3	6.15
[illegible]	Isoform 1 of E3 UFM1-protein ligase 1	[illegible]	[illegible]	[illegible]	[illegible]	[illegible]	[illegible]	[illegible]	[illegible]	[illegible]	[illegible]	[illegible]	[illegible]	[illegible]	[illegible]	[illegible]	[illegible]	[illegible]	[illegible]	[illegible]	[illegible]	[illegible]	[illegible]	[illegible]	[illegible]	[illegible]	[illegible]	[illegible]	[illegible]	[illegible]	[illegible]	[illegible]	[illegible]	[illegible]	[illegible]	[illegible]	[illegible]	[illegible]	[illegible]	[illegible]
[illegible]	Isoform 1 of Electrogenic sodium bicarbonate cotransporter 1	[illegible]	[illegible]	[illegible]	[illegible]	[illegible]	[illegible]	[illegible]	[illegible]	[illegible]	[illegible]	[illegible]	[illegible]	[illegible]	[illegible]	[illegible]	[illegible]	[illegible]	[illegible]	[illegible]	[illegible]	[illegible]	[illegible]	[illegible]	[illegible]	[illegible]	[illegible]	[illegible]	[illegible]	[illegible]	[illegible]	[illegible]	[illegible]	[illegible]	[illegible]	[illegible]	[illegible]	[illegible]	[illegible]	[illegible]
[illegible]	Isoform 1 of Electroneutral sodium bicarbonate exchanger 1	[illegible]	[illegible]	[illegible]	[illegible]	[illegible]	[illegible]	[illegible]	[illegible]	[illegible]	[illegible]	[illegible]	[illegible]	[illegible]	[illegible]	[illegible]	[illegible]	[illegible]	[illegible]	[illegible]	[illegible]	[illegible]	[illegible]	[illegible]	[illegible]	[illegible]	[illegible]	[illegible]	[illegible]	[illegible]	[illegible]	[illegible]	[illegible]	[illegible]	[illegible]	[illegible]	[illegible]	[illegible]	[illegible]	[illegible]
[illegible]	Isoform 1 of Elongation factor Tu, mitochondrial	[illegible]	[illegible]	[illegible]	[illegible]	[illegible]	[illegible]	[illegible]	[illegible]	[illegible]	[illegible]	[illegible]	[illegible]	[illegible]	[illegible]	[illegible]	[illegible]	[illegible]	[illegible]	[illegible]	[illegible]	[illegible]	[illegible]	[illegible]	[illegible]	[illegible]	[illegible]	[illegible]	[illegible]	[illegible]	[illegible]	[illegible]	[illegible]	[illegible]	[illegible]	[illegible]	[illegible]	[illegible]	[illegible]	[illegible]
[illegible]	Isoform 1 of Elongator complex protein 2	[illegible]	[illegible]	[illegible]	[illegible]	[illegible]	[illegible]	[illegible]	[illegible]	[illegible]	[illegible]	[illegible]	[illegible]	[illegible]	[illegible]	[illegible]	[illegible]	[illegible]	[illegible]	[illegible]	[illegible]	[illegible]	[illegible]	[illegible]	[illegible]	[illegible]	[illegible]	[illegible]	[illegible]	[illegible]	[illegible]	[illegible]	[illegible]	[illegible]	[illegible]	[illegible]	[illegible]	[illegible]	[illegible]	[illegible]
[illegible]	Isoform 1 of Endophilin-B1	[illegible]	[illegible]	[illegible]	[illegible]	[illegible]	[illegible]	[illegible]	[illegible]	[illegible]	[illegible]	[illegible]	[illegible]	[illegible]	[illegible]	[illegible]	[illegible]	[illegible]	[illegible]	[illegible]	[illegible]	[illegible]	[illegible]	[illegible]	[illegible]	[illegible]	[illegible]	[illegible]	[illegible]	[illegible]	[illegible]	[illegible]	[illegible]	[illegible]	[illegible]	[illegible]	[illegible]	[illegible]	[illegible]	[illegible]
[illegible]	Isoform 1 of Endophilin-B2	[illegible]	[illegible]	[illegible]	[illegible]	[illegible]	[illegible]	[illegible]	[illegible]	[illegible]	[illegible]	[illegible]	[illegible]	[illegible]	[illegible]	[illegible]	[illegible]	[illegible]	[illegible]	[illegible]	[illegible]	[illegible]	[illegible]	[illegible]	[illegible]	[illegible]	[illegible]	[illegible]	[illegible]	[illegible]	[illegible]	[illegible]	[illegible]	[illegible]	[illegible]	[illegible]	[illegible]	[illegible]	[illegible]	[illegible]
[illegible]	Isoform 1 of Endoplasmic reticulum metallopeptidase 1	[illegible]	[illegible]	[illegible]	[illegible]	[illegible]	[illegible]	[illegible]	[illegible]	[illegible]	[illegible]	[illegible]	[illegible]	[illegible]	[illegible]	[illegible]	[illegible]	[illegible]	[illegible]	[illegible]	[illegible]	[illegible]	[illegible]	[illegible]	[illegible]	[illegible]	[illegible]	[illegible]	[illegible]	[illegible]	[illegible]	[illegible]	[illegible]	[illegible]	[illegible]	[illegible]	[illegible]	[illegible]	[illegible]	[illegible]
[illegible]	Isoform 1 of Endoplasmic reticulum-Golgi intermediate compartment protein	[illegible]	[illegible]	[illegible]	[illegible]	[illegible]	[illegible]	[illegible]	[illegible]	[illegible]	[illegible]	[illegible]	[illegible]	[illegible]	[illegible]	[illegible]	[illegible]	[illegible]	[illegible]	[illegible]	[illegible]	[illegible]	[illegible]	[illegible]	[illegible]	[illegible]	[illegible]	[illegible]	[illegible]	[illegible]	[illegible]	[illegible]	[illegible]	[illegible]	[illegible]	[illegible]	[illegible]	[illegible]	[illegible]	[illegible]
[illegible]	Isoform 1 of Enhancer of mRNA-decapping protein 4	[illegible]	[illegible]	[illegible]	[illegible]	[illegible]	[illegible]	[illegible]	[illegible]	[illegible]	[illegible]	[illegible]	[illegible]	[illegible]	[illegible]	[illegible]	[illegible]	[illegible]	[illegible]	[illegible]	[illegible]	[illegible]	[illegible]	[illegible]	[illegible]	[illegible]	[illegible]	[illegible]	[illegible]	[illegible]	[illegible]	[illegible]	[illegible]	[illegible]	[illegible]	[illegible]	[illegible]	[illegible]	[illegible]	[illegible]
[illegible]	Isoform 1 of Enolase-phosphatase E1	[illegible]	[illegible]	[illegible]	[illegible]	[illegible]	[illegible]	[illegible]	[illegible]	[illegible]	[illegible]	[illegible]	[illegible]	[illegible]	[illegible]	[illegible]	[illegible]	[illegible]	[illegible]	[illegible]	[illegible]	[illegible]	[illegible]	[illegible]	[illegible]	[illegible]	[illegible]	[illegible]	[illegible]	[illegible]	[illegible]	[illegible]	[illegible]	[illegible]	[illegible]	[illegible]	[illegible]	[illegible]	[illegible]	[illegible]
[illegible]	Isoform 1 of Enoyl-CoA delta isomerase 2, mitochondrial	[illegible]	[illegible]	[illegible]	[illegible]	[illegible]	[illegible]	[illegible]	[illegible]	[illegible]	[illegible]	[illegible]	[illegible]	[illegible]	[illegible]	[illegible]	[illegible]	[illegible]	[illegible]	[illegible]	[illegible]	[illegible]	[illegible]	[illegible]	[illegible]	[illegible]	[illegible]	[illegible]	[illegible]	[illegible]	[illegible]	[illegible]	[illegible]	[illegible]	[illegible]	[illegible]	[illegible]	[illegible]	[illegible]	[illegible]
[illegible]	Isoform 1 of Ephexin-1	[illegible]	[illegible]	[illegible]	[illegible]	[illegible]	[illegible]	[illegible]	[illegible]	[illegible]	[illegible]	[illegible]	[illegible]	[illegible]	[illegible]	[illegible]	[illegible]	[illegible]	[illegible]	[illegible]	[illegible]	[illegible]	[illegible]	[illegible]	[illegible]	[illegible]	[illegible]	[illegible]	[illegible]	[illegible]	[illegible]	[illegible]	[illegible]	[illegible]	[illegible]	[illegible]	[illegible]	[illegible]	[illegible]	[illegible]
[illegible]	Isoform 1 of Epidermal growth factor receptor kinase substrate 8-like prote	[illegible]	[illegible]	[illegible]	[illegible]	[illegible]	[illegible]	[illegible]	[illegible]	[illegible]	[illegible]	[illegible]	[illegible]	[illegible]	[illegible]	[illegible]	[illegible]	[illegible]	[illegible]	[illegible]	[illegible]	[illegible]	[illegible]	[illegible]	[illegible]	[illegible]	[illegible]	[illegible]	[illegible]	[illegible]	[illegible]	[illegible]	[illegible]	[illegible]	[illegible]	[illegible]	[illegible]	[illegible]	[illegible]	[illegible]
[illegible]	Isoform 1 of Epidermal growth factor receptor substrate 15	[illegible]	[illegible]	[illegible]	[illegible]	[illegible]	[illegible]	[illegible]	[illegible]	[illegible]	[illegible]	[illegible]	[illegible]	[illegible]	[illegible]	[illegible]	[illegible]	[illegible]	[illegible]	[illegible]	[illegible]	[illegible]	[illegible]	[illegible]	[illegible]	[illegible]	[illegible]	[illegible]	[illegible]	[illegible]	[illegible]	[illegible]	[illegible]	[illegible]	[illegible]	[illegible]	[illegible]	[illegible]	[illegible]	[illegible]
[illegible]	Isoform 1 of Epoxide hydrolase 2	[illegible]	[illegible]	[illegible]	[illegible]	[illegible]	[illegible]	[illegible]	[illegible]	[illegible]	[illegible]	[illegible]	[illegible]	[illegible]	[illegible]	[illegible]	[illegible]	[illegible]	[illegible]	[illegible]	[illegible]	[illegible]	[illegible]	[illegible]	[illegible]	[illegible]	[illegible]	[illegible]	[illegible]	[illegible]	[illegible]	[illegible]	[illegible]	[illegible]	[illegible]	[illegible]	[illegible]	[illegible]	[illegible]	[illegible]
[illegible]	Isoform 1 of Epsin-1	[illegible]	[illegible]	[illegible]	[illegible]	[illegible]	[illegible]	[illegible]	[illegible]	[illegible]	[illegible]	[illegible]	[illegible]	[illegible]	[illegible]	[illegible]	[illegible]	[illegible]	[illegible]	[illegible]	[illegible]	[illegible]	[illegible]	[illegible]	[illegible]	[illegible]	[illegible]	[illegible]	[illegible]	[illegible]	[illegible]	[illegible]	[illegible]	[illegible]	[illegible]	[illegible]	[illegible]	[illegible]	[illegible]	[illegible]
[illegible]	Isoform 1 of Estradiol 17-beta-dehydrogenase 12	[illegible]	[illegible]	[illegible]	[illegible]	[illegible]	[illegible]	[illegible]	[illegible]	[illegible]	[illegible]	[illegible]	[illegible]	[illegible]	[illegible]	[illegible]	[illegible]	[illegible]	[illegible]	[illegible]	[illegible]	[illegible]	[illegible]	[illegible]	[illegible]	[illegible]	[illegible]	[illegible]	[illegible]	[illegible]	[illegible]	[illegible]	[illegible]	[illegible]	[illegible]	[illegible]	[illegible]	[illegible]	[illegible]	[illegible]
[illegible]	Isoform 1 of Eukaryotic translation initiation factor 2A	[illegible]	[illegible]	[illegible]	[illegible]	[illegible]	[illegible]	[illegible]	[illegible]	[illegible]	[illegible]	[illegible]	[illegible]	[illegible]	[illegible]	[illegible]	[illegible]	[illegible]	[illegible]	[illegible]	[illegible]	[illegible]	[illegible]	[illegible]	[illegible]	[illegible]	[illegible]	[illegible]	[illegible]	[illegible]	[illegible]	[illegible]	[illegible]	[illegible]	[illegible]	[illegible]	[illegible]	[illegible]	[illegible]	[illegible]
[illegible]	Isoform 1 of Eukaryotic translation initiation factor 3 subunit K	[illegible]	[illegible]	[illegible]	[illegible]	[illegible]	[illegible]	[illegible]	[illegible]	[illegible]	[illegible]	[illegible]	[illegible]	[illegible]	[illegible]	[illegible]	[illegible]	[illegible]	[illegible]	[illegible]	[illegible]	[illegible]	[illegible]	[illegible]	[illegible]	[illegible]	[illegible]	[illegible]	[illegible]	[illegible]	[illegible]	[illegible]	[illegible]	[illegible]	[illegible]	[illegible]	[illegible]	[illegible]	[illegible]	[illegible]
[illegible]	Isoform 1 of Exocyst complex component 6B	[illegible]	[illegible]	[illegible]	[illegible]	[illegible]	[illegible]	[illegible]	[illegible]	[illegible]	[illegible]	[illegible]	[illegible]	[illegible]	[illegible]	[illegible]	[illegible]	[illegible]	[illegible]	[illegible]	[illegible]	[illegible]	[illegible]	[illegible]	[illegible]	[illegible]	[illegible]	[illegible]	[illegible]	[illegible]	[illegible]	[illegible]	[illegible]	[illegible]	[illegible]	[illegible]	[illegible]	[illegible]	[illegible]	[illegible]
[illegible]	Isoform 1 of Exportin-5	[illegible]	[illegible]	[illegible]	[illegible]	[illegible]	[illegible]	[illegible]	[illegible]	[illegible]	[illegible]	[illegible]	[illegible]	[illegible]	[illegible]	[illegible]	[illegible]	[illegible]	[illegible]	[illegible]	[illegible]	[illegible]	[illegible]	[illegible]	[illegible]	[illegible]	[illegible]	[illegible]	[illegible]	[illegible]	[illegible]	[illegible]	[illegible]	[illegible]	[illegible]	[illegible]	[illegible]	[illegible]	[illegible]	[illegible]
[illegible]	Isoform 1 of Exportin-7	[illegible]	[illegible]	[illegible]	[illegible]	[illegible]	[illegible]	[illegible]	[illegible]	[illegible]	[illegible]	[illegible]	[illegible]	[illegible]	[illegible]	[illegible]	[illegible]	[illegible]	[illegible]	[illegible]	[illegible]	[illegible]	[illegible]	[illegible]	[illegible]	[illegible]	[illegible]	[illegible]	[illegible]	[illegible]	[illegible]	[illegible]	[illegible]	[illegible]	[illegible]	[illegible]	[illegible]	[illegible]	[illegible]	[illegible]
[illegible]	Isoform 1 of Extended synaptotagmin-1	[illegible]	[illegible]	[illegible]	[illegible]	[illegible]	[illegible]	[illegible]	[illegible]	[illegible]	[illegible]	[illegible]	[illegible]	[illegible]	[illegible]	[illegible]	[illegible]	[illegible]	[illegible]	[illegible]	[illegible]	[illegible]	[illegible]	[illegible]	[illegible]	[illegible]	[illegible]	[illegible]	[illegible]	[illegible]	[illegible]	[illegible]	[illegible]	[illegible]	[illegible]	[illegible]	[illegible]	[illegible]	[illegible]	[illegible]
[illegible]	Isoform 1 of Extended synaptotagmin-2	[illegible]	[illegible]	[illegible]	[illegible]	[illegible]	[illegible]	[illegible]	[illegible]	[illegible]	[illegible]	[illegible]	[illegible]	[illegible]	[illegible]	[illegible]	[illegible]	[illegible]	[illegible]	[illegible]	[illegible]	[illegible]	[illegible]	[illegible]	[illegible]	[illegible]	[illegible]	[illegible]	[illegible]	[illegible]	[illegible]	[illegible]	[illegible]	[illegible]	[illegible]	[illegible]	[illegible]	[illegible]	[illegible]	[illegible]
[illegible]	Isoform 1 of Fanconi anemia group I protein homolog	[illegible]	[illegible]	[illegible]	[illegible]	[illegible]	[illegible]	[illegible]	[illegible]	[illegible]	[illegible]	[illegible]	[illegible]	[illegible]	[illegible]	[illegible]	[illegible]	[illegible]	[illegible]	[illegible]	[illegible]	[illegible]	[illegible]	[illegible]	[illegible]	[illegible]	[illegible]	[illegible]	[illegible]	[illegible]	[illegible]	[illegible]	[illegible]	[illegible]	[illegible]	[illegible]	[illegible]	[illegible]	[illegible]	[illegible]
[illegible]	Isoform 1 of Fanconi anemia group M protein homolog	[illegible]	[illegible]	[illegible]	[illegible]	[illegible]	[illegible]	[illegible]	[illegible]	[illegible]	[illegible]	[illegible]	[illegible]	[illegible]	[illegible]	[illegible]	[illegible]	[illegible]	[illegible]	[illegible]	[illegible]	[illegible]	[illegible]	[illegible]	[illegible]	[illegible]	[illegible]	[illegible]	[illegible]	[illegible]	[illegible]	[illegible]	[illegible]	[illegible]	[illegible]	[illegible]	[illegible]	[illegible]	[illegible]	[illegible]
[illegible]	Isoform 1 of FAS-associated factor 2	[illegible]	[illegible]	[illegible]	[illegible]	[illegible]	[illegible]	[illegible]	[illegible]	[illegible]	[illegible]	[illegible]	[illegible]	[illegible]	[illegible]	[illegible]	[illegible]	[illegible]	[illegible]	[illegible]	[illegible]	[illegible]	[illegible]	[illegible]	[illegible]	[illegible]	[illegible]	[illegible]	[illegible]	[illegible]	[illegible]	[illegible]	[illegible]	[illegible]	[illegible]	[illegible]	[illegible]	[illegible]	[illegible]	[illegible]
[illegible]	Isoform 1 of Fibronectin type III and SPRY domain-containing protein 1	[illegible]	[illegible]	[illegible]	[illegible]	[illegible]	[illegible]	[illegible]	[illegible]	[illegible]	[illegible]	[illegible]	[illegible]	[illegible]	[illegible]	[illegible]	[illegible]	[illegible]	[illegible]	[illegible]	[illegible]	[illegible]	[illegible]	[illegible]	[illegible]	[illegible]	[illegible]	[illegible]	[illegible]	[illegible]	[illegible]	[illegible]	[illegible]	[illegible]	[illegible]	[illegible]	[illegible]	[illegible]	[illegible]	[illegible]
[illegible]	Isoform 1 of Filamin A-interacting protein 1-like	[illegible]	[illegible]	[illegible]	[illegible]	[illegible]	[illegible]	[illegible]	[illegible]	[illegible]	[illegible]	[illegible]	[illegible]	[illegible]	[illegible]	[illegible]	[illegible]	[illegible]	[illegible]	[illegible]	[illegible]	[illegible]	[illegible]	[illegible]	[illegible]	[illegible]	[illegible]	[illegible]	[illegible]	[illegible]	[illegible]	[illegible]	[illegible]	[illegible]	[illegible]	[illegible]	[illegible]	[illegible]	[illegible]	[illegible]
[illegible]	Isoform 1 of Friend of PRMT1 protein	[illegible]	[illegible]	[illegible]	[illegible]	[illegible]	[illegible]	[illegible]	[illegible]	[illegible]	[illegible]	[illegible]	[illegible]	[illegible]	[illegible]	[illegible]	[illegible]	[illegible]	[illegible]	[illegible]	[illegible]	[illegible]	[illegible]	[illegible]	[illegible]	[illegible]	[illegible]	[illegible]	[illegible]	[illegible]	[illegible]	[illegible]	[illegible]	[illegible]	[illegible]	[illegible]	[illegible]	[illegible]	[illegible]	[illegible]
[illegible]	Isoform 1 of Ganglioside-induced differentiation-associated protein 1	[illegible]	[illegible]	[illegible]	[illegible]	[illegible]	[illegible]	[illegible]	[illegible]	[illegible]	[illegible]	[illegible]	[illegible]	[illegible]	[illegible]	[illegible]	[illegible]	[illegible]	[illegible]	[illegible]	[illegible]	[illegible]	[illegible]	[illegible]	[illegible]	[illegible]	[illegible]	[illegible]	[illegible]	[illegible]	[illegible]	[illegible]	[illegible]	[illegible]	[illegible]	[illegible]	[illegible]	[illegible]	[illegible]	[illegible]
[illegible]	Isoform 1 of General vesicular transport factor p115	[illegible]	[illegible]	[illegible]	[illegible]	[illegible]	[illegible]	[illegible]	[illegible]	[illegible]	[illegible]	[illegible]	[illegible]	[illegible]	[illegible]	[illegible]	[illegible]	[illegible]	[illegible]	[illegible]	[illegible]	[illegible]	[illegible]	[illegible]	[illegible]	[illegible]	[illegible]	[illegible]	[illegible]	[illegible]	[illegible]	[illegible]	[illegible]	[illegible]	[illegible]	[illegible]	[illegible]	[illegible]	[illegible]	[illegible]
[illegible]	Isoform 1 of Geranylgeranyl transferase type-2 subunit alpha	[illegible]	[illegible]	[illegible]	[illegible]	[illegible]	[illegible]	[illegible]	[illegible]	[illegible]	[illegible]	[illegible]	[illegible]	[illegible]	[illegible]	[illegible]	[illegible]	[illegible]	[illegible]	[illegible]	[illegible]	[illegible]	[illegible]	[illegible]	[illegible]	[illegible]	[illegible]	[illegible]	[illegible]	[illegible]	[illegible]	[illegible]	[illegible]	[illegible]	[illegible]	[illegible]	[illegible]	[illegible]	[illegible]	[illegible]
[illegible]	Isoform 1 of Glial fibrillary acidic protein	[illegible]	[illegible]	[illegible]	[illegible]	[illegible]	[illegible]	[illegible]	[illegible]	[illegible]	[illegible]	[illegible]	[illegible]	[illegible]	[illegible]	[illegible]	[illegible]	[illegible]	[illegible]	[illegible]	[illegible]	[illegible]	[illegible]	[illegible]	[illegible]	[illegible]	[illegible]	[illegible]	[illegible]	[illegible]	[illegible]	[illegible]	[illegible]	[illegible]	[illegible]	[illegible]	[illegible]	[illegible]	[illegible]	[illegible]
[illegible]	Isoform 1 of Glucosamine--fructose-6-phosphate aminotransferase [isomeri	[illegible]	[illegible]	[illegible]	[illegible]	[illegible]	[illegible]	[illegible]	[illegible]	[illegible]	[illegible]	[illegible]	[illegible]	[illegible]	[illegible]	[illegible]	[illegible]	[illegible]	[illegible]	[illegible]	[illegible]	[illegible]	[illegible]	[illegible]	[illegible]	[illegible]	[illegible]	[illegible]	[illegible]	[illegible]	[illegible]	[illegible]	[illegible]	[illegible]	[illegible]	[illegible]	[illegible]	[illegible]	[illegible]	[illegible]
[illegible]	Isoform 1 of Glyoxalase domain-containing protein 4	[illegible]	[illegible]	[illegible]	[illegible]	[illegible]	[illegible]	[illegible]	[illegible]	[illegible]	[illegible]	[illegible]	[illegible]	[illegible]	[illegible]	[illegible]	[illegible]	[illegible]	[illegible]	[illegible]	[illegible]	[illegible]	[illegible]	[illegible]	[illegible]	[illegible]	[illegible]	[illegible]	[illegible]	[illegible]	[illegible]	[illegible]	[illegible]	[illegible]	[illegible]	[illegible]	[illegible]	[illegible]	[illegible]	[illegible]
[illegible]	Isoform 1 of Golgi phosphoprotein 3	[illegible]	[illegible]	[illegible]	[illegible]	[illegible]	[illegible]	[illegible]	[illegible]	[illegible]	[illegible]	[illegible]	[illegible]	[illegible]	[illegible]	[illegible]	[illegible]	[illegible]	[illegible]	[illegible]	[illegible]	[illegible]	[illegible]	[illegible]	[illegible]	[illegible]	[illegible]	[illegible]	[illegible]	[illegible]	[illegible]	[illegible]	[illegible]	[illegible]	[illegible]	[illegible]	[illegible]	[illegible]	[illegible]	[illegible]

IPI00165716.4	Isoform 1 of Golgi reassembly-stacking protein 2	41.02%	4	6	20	139
IPI00272521.3	Isoform 1 of Golgi to ER traffic protein 4 homolog	87.77%	4	4	30	151
IPI00221745.1	Isoform 1 of Golgi-associated PDZ and coiled-coil motif-containing protein	70.63%	3	4	41	117
IPI00754998.1	Isoform 1 of G-protein coupled receptor 98	58.10%	4	3	319	1007
IPI00119058.1	Isoform 1 of Growth factor receptor-bound protein 2	78.80%	4	7	21	162
IPI01023179.1	Isoform 1 of GTPase HRas	94.71%	3	6	25	134
IPI00127930.1	Isoform 1 of Guanine nucleotide-binding protein subunit beta-5	63.29%	2	7	22	78
IPI00762316.4	Isoform 1 of Guanine nucleotide-binding protein-like 1	71.50%	3	6	49	232
IPI00222461.4	Isoform 1 of Guanine nucleotide-binding protein-like 3	87.17%	4	22	61	419
IPI00111166.7	Isoform 1 of Haloacid dehalogenase-like hydrolase domain-containing prote	77.61%	4	4	19	87
IPI00762140.1	Isoform 1 of HEAT repeat-containing protein 5B	77.15%	4	9	182	503
IPI00222937.1	Isoform 1 of Heat shock 70 kDa protein 13	56.48%	2	7	31	123
IPI00119066.2	Isoform 1 of Heat shock 70 kDa protein 14	93.52%	4	15	67	386
IPI00317710.1	Isoform 1 of Heat shock 70 kDa protein 4L	95.94%	4	41	124	830
IPI00321022.2	Isoform 1 of Hemicentin-2	69.75%	3	7	338	1122
IPI00269661.1	Isoform 1 of Heterogeneous nuclear ribonucleoprotein A3	73.35%	14	20	43	816
IPI00226073.2	Isoform 1 of Heterogeneous nuclear ribonucleoprotein F	82.65%	2	13	42	684
IPI00121760.5	Isoform 1 of Heterogeneous nuclear ribonucleoprotein L-like	82.23%	4	13	53	369
IPI00132443.3	Isoform 1 of Heterogeneous nuclear ribonucleoprotein M	94.10%	5	29	99	1220
IPI00406117.1	Isoform 1 of Heterogeneous nuclear ribonucleoprotein C	84.59%	12	31	84	972
IPI00123501.3	Isoform 1 of Heterogeneous nuclear ribonucleoprotein U-like protein 1	63.91%	3	8	76	365
IPI00828488.1	Isoform 1 of Heterogeneous nuclear ribonucleoproteins A2/B1	98.87%	3	3	49	2492
IPI00515403.1	Isoform 1 of Histone acetyltransferase MYST2	76.84%	5	6	67	203
IPI00313968.8	Isoform 1 of Histone-lysine N-methyltransferase setd3	80.13%	7	11	54	188
IPI00221566.3	Isoform 1 of HIV Tat-specific factor 1 homolog	71.07%	2	13	56	176
IPI00173157.4	Isoform 1 of Huntingtin-interacting protein K	64.34%	2	6	11	77
IPI00115866.3	Isoform 1 of Hydroxyacylglutathione hydrolase, mitochondrial	66.67%	5	6	27	103
IPI00120264.1	Isoform 1 of Immunity-related GTPase family M protein 1	77.51%	2	5	36	108
IPI00626994.3	Isoform 1 of Importin-5	88.42%	4	42	107	1309
IPI00265986.1	Isoform 1 of Inner centromere protein	73.41%	2	5	103	591
IPI00127050.1	Isoform 1 of Inorganic pyrophosphatase 2, mitochondrial	77.27%	5	11	28	97
IPI00761863.1	Isoform 1 of Insulin-like growth factor 2 mRNA-binding protein 2	70.27%	3	8	57	172
IPI00380394.6	Isoform 1 of Integrator complex subunit 3	73.29%	3	5	83	230
IPI00649251.1	Isoform 1 of Integrin beta-4	74.64%	4	13	144	429
IPI00396804.2	Isoform 1 of Integrin-linked kinase-associated serine/threonine phosphatas	68.88%	2	3	34	76
IPI00555078.1	Isoform 1 of Interleukin enhancer-binding factor 3	71.71%	4	27	78	966
IPI00129356.1	Isoform 1 of Intersectin-1	67.56%	10	5	156	538
IPI00459725.2	Isoform 1 of Isocitrate dehydrogenase [NAD] subunit alpha, mitochondrial	77.60%	2	18	42	412
IPI00459134.2	Isoform 1 of KDEL motif-containing protein 1	85.86%	4	3	50	119
IPI00330539.1	Isoform 1 of Kin of IRRE-like protein 1	64.26%	1	3	48	124
IPI00330497.4	Isoform 1 of KN motif and ankyrin repeat domain-containing protein 2	75.09%	2	7	64	186
IPI00929786.1	Isoform 1 of La-related protein 1	83.96%	2	27	112	504
IPI00620564.3	Isoform 1 of La-related protein 4B	58.57%	3	7	65	218
IPI00340860.5	Isoform 1 of La-related protein 7	79.30%	3	12	73	236
IPI00330362.5	Isoform 1 of Large subunit GTPase 1 homolog	75.93%	2	3	58	213
IPI00654388.2	Isoform 1 of Leucine-rich repeat flightless-interacting protein 1	91.91%	4	17	86	534
IPI00403079.4	Isoform 1 of Leukocyte surface antigen CD47	60.40%	1	1	22	206
IPI00111789.2	Isoform 1 of LIM domain-binding protein 1	77.86%	4	1	36	136
IPI00405437.3	Isoform 1 of Lipase maturation factor 1	40.24%	6	2	30	89
IPI00221494.7	Isoform 1 of Lipoma-preferred partner homolog	70.15%	5	12	35	396
IPI00228693.4	Isoform 1 of LisH domain and HEAT repeat-containing protein KIAA1468	62.50%	5	8	85	252
IPI00625955.1	Isoform 1 of Long-chain-fatty-acid--CoA ligase 6	66.71%	11	22	56	199
IPI00134135.1	Isoform 1 of Low molecular weight phosphotyrosine protein phosphatase	91.77%	2	1	22	124
IPI00411145.2	Isoform 1 of Low-density lipoprotein receptor-related protein 4	60.47%	2	1	98	210
IPI00649422.1	Isoform 1 of Luc7-like protein 3	67.36%	5	10	54	244
IPI00761693.5	Isoform 1 of MAGUK p55 subfamily member 7	53.65%	4	5	39	67
IPI00316989.7	Isoform 1 of Mammalian ependymin-related protein 1	72.32%	2	6	21	70
IPI00828556.1	Isoform 1 of MAP kinase-activating death domain protein	61.07%	24	13	111	304
IPI00282957.4	Isoform 1 of MAP7 domain-containing protein 1	80.38%	4	9	105	364
IPI00808096.3	Isoform 1 of MAP7 domain-containing protein 2	69.01%	9	6	75	275
IPI00988679.1	Isoform 1 of Mediator of RNA polymerase II transcription subunit 23	61.81%	4	4	84	201
IPI00222037.4	Isoform 1 of Melanoma inhibitory activity protein 3	57.56%	3	26	155	522
IPI00110402.1	Isoform 1 of Metabotropic glutamate receptor 1	52.29%	3	4	73	205
IPI00659932.4	Isoform 1 of Metabotropic glutamate receptor 5	55.94%	3	3	83	198
IPI00124900.1	Isoform 1 of Methylglutaconyl-CoA hydratase, mitochondrial	84.08%	7	10	38	152
IPI00408119.6	Isoform 1 of Microtubule-associated protein 4	85.24%	6	27	109	1560
IPI00115833.2	Isoform 1 of Microtubule-associated protein 6	93.71%	3	52	99	616
IPI00403682.2	Isoform 1 of Microtubule-associated protein RP/EB family member 2	76.07%	4	5	26	114
~~IPI00112072.1~~	~~Isoform 1 of Minor histocompatibility antigen H13~~	~~38.10%~~	~~6~~	~~7~~	~~17~~	~~174~~
IPI00875016.1	Isoform 1 of Mitochondrial glutamate carrier 2	65.63%	3	5	25	107
IPI00165694.1	Isoform 1 of Mitochondrial import receptor subunit TOM34	94.17%	2	12	43	205
IPI00312244.3	Isoform 1 of Mitofusin-2	82.03%	6	4	64	178
IPI00124052.1	Isoform 1 of Mitotic spindle assembly checkpoint protein MAD1	86.47%	2	5	92	251
IPI00115975.8	Isoform 1 of Mixed lineage kinase domain-like protein	80.93%	3	4	55	162
IPI00225912.8	Isoform 1 of MKI67 FHA domain-interacting nucleolar phosphoprotein	80.13%	3	10	35	188
IPI00108431.8	Isoform 1 of MMP37-like protein, mitochondrial	74.18%	4	3	30	63
IPI00317858.2	Isoform 1 of Motile sperm domain-containing protein 2	49.61%	4	5	36	84
IPI00761839.1	Isoform 1 of Mps one binder kinase activator-like 1B	64.81%	4	4	15	66
IPI00453849.3	Isoform 1 of mRNA cap guanine-N7 methyltransferase	93.55%	6	8	49	191
IPI00114352.1	Isoform 1 of Myc box-dependent-interacting protein 1	68.37%	2	2	52	499
IPI00115240.1	Isoform 1 of Myelin basic protein	71.20%	3	1	29	311
IPI00226891.1	Isoform 1 of Myelin expression factor 2	86.97%	7	23	68	628
IPI00263013.4	Isoform 1 of Myelin proteolipid protein	68.95%	3	10	22	306
IPI00989268.1	Isoform 1 of Myoferlin	81.98%	6	98	227	1661
IPI00410957.2	Isoform 1 of Myosin-Vb	65.24%	6	4	161	506
IPI00109311.3	Isoform 1 of Na(+)/H(+) exchange regulatory cofactor NHE-RF1	86.76%	1	12	41	275
IPI00308063.3	Isoform 1 of Na(+)/H(+) exchange regulatory cofactor NHE-RF2	78.04%	3	4	27	59
IPI00322206.2	Isoform 1 of N-acylneuraminate cytidylyltransferase	68.06%	2	6	36	94
IPI00110265.1	Isoform 1 of NAD-dependent deacetylase sirtuin-2	88.17%	3	20	36	224
IPI00169925.2	Isoform 1 of NADH dehydrogenase [ubiquinone] flavoprotein 2, mitochondr	85.89%	4	12	27	325
IPI00119131.2	Isoform 1 of NADH-cytochrome b5 reductase 1	72.13%	5	8	28	132
IPI00121079.3	Isoform 1 of NADH-cytochrome b5 reductase 3	94.68%	3	15	35	550
IPI00226576.1	Isoform 1 of N-alpha-acetyltransferase 25, NatB auxiliary subunit	77.98%	4	12	90	359
IPI00226943.2	Isoform 1 of NEDD8-activating enzyme E1 catalytic subunit	82.47%	4	8	41	191
IPI00845851.1	Isoform 1 of Nesprin-2	78.46%	7	15	696	2790
IPI00453692.4	Isoform 1 of Nestin	80.10%	7	99	171	2680
IPI00420651.3	Isoform 1 of Neurabin-2	60.10%	2	9	65	239
IPI00222149.4	Isoform 1 of Neural cell adhesion molecule L1-like protein	51.78%	5	4	65	188
IPI00469204.2	Isoform 1 of Neurobeachin	71.25%	4	9	233	981
IPI00675407.4	Isoform 1 of Neurobeachin-like protein 2	65.68%	2	4	180	527
IPI00331299.9	Isoform 1 of Neurochondrin	74.49%	2	24	53	248
IPI00119952.1	Isoform 1 of Neuronal membrane glycoprotein M6-b	28.66%	9	7	15	78
IPI00845776.1	Isoform 1 of Neuronal-specific septin-3	68.86%	3	11	27	139
IPI00403058.1	Isoform 1 of Neutral alpha-glucosidase AB	83.69%	2	1	75	671
IPI00110435.2	Isoform 1 of Nischarin	54.43%	9	6	93	282
IPI00109529.2	Isoform 1 of Nitric oxide synthase-interacting protein	77.41%	3	4	24	55
IPI00320016.7	Isoform 1 of Non-POU domain-containing octamer-binding protein	82.24%	3	23	56	928
IPI00112460.1	Isoform 1 of Nuclear distribution protein nudE homolog 1	90.99%	6	4	44	106
IPI00318238.5	Isoform 1 of Nuclear distribution protein nudE-like 1	92.46%	4	3	48	125
IPI00227669.4	Isoform 1 of Nuclear export mediator factor Nemf	58.27%	4	7	83	308
IPI00113873.2	Isoform 1 of Nuclear factor NF-kappa-B p105 subunit	59.63%	7	9	63	198
IPI00330146.6	Isoform 1 of Nuclear fragile X mental retardation-interacting protein 2	62.14%	2	14	57	374
IPI00129535.1	Isoform 1 of Nuclear pore complex protein Nup160	74.89%	2	10	101	440
IPI00229021.1	Isoform 1 of Nuclear pore complex protein Nup88	56.84%	3	5	58	189
IPI00222307.1	Isoform 1 of Nuclear pore complex protein Nup93	80.34%	2	23	99	492
IPI00465884.7	Isoform 1 of Nuclear protein localization protein 4 homolog	79.28%	2	11	52	251
IPI00465879.2	Isoform 1 of Nuclear-interacting partner of ALK	71.26%	4	3	33	90
IPI00315517.14	Isoform 1 of Nucleoporin SEH1	82.78%	2	6	36	129
IPI00124073.1	Isoform 1 of Nucleoredoxin	56.55%	2	5	23	58
IPI00268927.1	Isoform 1 of NudC domain-containing protein 3	79.34%	4	11	30	119
IPI00111981.1	Isoform 1 of Obg-like ATPase 1	65.40%	3	13	35	407
IPI00133608.3	Isoform 1 of OCIA domain-containing protein 1	80.57%	3	5	23	97
IPI00121018.3	Isoform 1 of Opioid growth factor receptor	79.94%	4	9	52	187
IPI00277552.3	Isoform 1 of Oxidation resistance protein 1	70.79%	6	30	86	371
IPI00123765.2	Isoform 1 of Oxidoreductase NAD-binding domain-containing protein 1	81.99%	2	1	26	70
IPI00469318.1	Isoform 1 of Oxysterol-binding protein-related protein 1	66.84%	8	3	72	195
IPI00318000.4	Isoform 1 of Pachytene checkpoint protein 2 homolog	59.72%	2	4	38	114
IPI00129298.1	Isoform 1 of Paralemmin-1	79.63%	2	15	45	252
IPI00229685.2	Isoform 1 of Partner of Y14 and mago	76.85%	2	5	18	110
IPI00115257.1	Isoform 1 of PC4 and SFRS1-interacting protein	87.31%	4	21	71	622
IPI00828969.2	Isoform 1 of PDZ and LIM domain protein 5	68.87%	9	14	62	377
IPI00130607.8	Isoform 1 of PDZ and LIM domain protein 7	79.43%	5	7	41	209
IPI00127764.3	Isoform 1 of Pericentriolar material 1 protein	65.53%	2	28	195	937
IPI00319131.4	Isoform 1 of Peroxisomal biogenesis factor 19	78.26%	2	6	17	84
IPI00318829.6	Isoform 1 of PHD finger protein 10	64.79%	4	2	40	148
IPI00408894.3	Isoform 1 of Phosphatidylinositol-4-phosphate 3-kinase C2 domain-containi	70.11%	4	5	154	494
IPI00224093.2	Isoform 1 of Phytanoyl-CoA hydroxylase-interacting protein-like	59.73%	5	8	31	106
IPI00473693.3	Isoform 1 of Plakophilin-4	79.50%	5	1	126	513
IPI00471475.1	Isoform 1 of Plasminogen activator inhibitor 1 RNA-binding protein	81.57%	3	2	54	750
IPI00309207.5	Isoform 1 of Platelet-activating factor acetylhydrolase IB subunit alpha	84.15%	3	16	46	281
IPI00112473.2	Isoform 1 of Poly [ADP-ribose] polymerase 1	73.84%	2	3	111	578
IPI00127707.1	Isoform 1 of Poly(rC)-binding protein 2	98.90%	4	11	41	744
IPI00652919.2	Isoform 1 of Poly(U)-binding-splicing factor PUF60	82.27%	1	1	57	631
IPI00420710.1	Isoform 1 of Polypeptide N-acetylgalactosaminyltransferase 2	75.79%	2	8	55	191
IPI00321923.5	Isoform 1 of Polyribonucleotide nucleotidyltransferase 1, mitochondria	81.23%	3	11	92	347
IPI00133589.2	Isoform 1 of Porphobilinogen deaminase	78.95%	2	6	39	152
IPI00110716.1	Isoform 1 of Pre-mRNA 3'-end-processing factor FIP1	49.40%	7	9	39	232
IPI00480507.1	Isoform 1 of Pre-mRNA-processing factor 19	84.13%	3	17	39	642
IPI00284213.3	Isoform 1 of Pre-mRNA-processing factor 40 homolog A	58.76%	8	16	74	327
IPI00130409.1	Isoform 1 of Pre-mRNA-processing factor 6	78.96%	2	21	121	492
IPI00420546.4	Isoform 1 of Pre-rRNA-processing protein TSR1 homolog	69.86%	3	9	67	254
IPI00354432.4	Isoform 1 of PRKC apoptosis WT1 regulator protein	94.59%	2	12	39	271
IPI00122038.3	Isoform 1 of Probable ATP-dependent RNA helicase DDX27	76.84%	2	9	92	312
IPI00468896.7	Isoform 1 of Probable ATP-dependent RNA helicase DDX46	72.19%	3	27	112	431
IPI00135559.3	Isoform 1 of Probable G-protein coupled receptor 37	40.17%	4	1	30	89
IPI00165762.1	Isoform 1 of Probable RNA-binding protein 19	75.21%	2	11	91	294
IPI00228645.5	Isoform 1 of Profilin-2	74.29%	4	7	15	89
IPI00453818.3	Isoform 1 of Programmed cell death 6-interacting protein	77.45%	5	37	94	690
IPI00224078.1	Isoform 1 of Prolyl endopeptidase-like	50.76%	3	3	43	76
IPI00396680.3	Isoform 1 of Proteasome activator complex subunit 4	72.00%	3	3	148	502
IPI00321646.4	Isoform 1 of Proteasome-associated protein ECM29 homolog	84.08%	6	23	193	916
IPI00395157.2	Isoform 1 of Protein 4.1	63.99%	11	1	67	172
IPI00123565.1	Isoform 1 of Protein AATF	58.37%	3	7	37	84
IPI00134093.5	Isoform 1 of Protein bassoon	78.16%	2	68	309	1349
IPI00227808.3	Isoform 1 of Protein CDV3	90.04%	5	6	36	377
IPI00756750.2	Isoform 1 of Protein FAM160B1	62.57%	3	4	45	137
IPI00222393.2	Isoform 1 of Protein FAM164A	68.52%	2	5	39	186
IPI00223670.2	Isoform 1 of Protein FAM40A	76.46%	6	1	68	198
IPI00109033.3	Isoform 1 of Protein FAM54B	57.79%	4	3	18	38
IPI00453799.3	Isoform 1 of Protein Jade-1	74.22%	2	6	72	157
IPI00647986.1	Isoform 1 of Protein KIAA1045	43.00%	3	9	25	103
IPI00125855.1	Isoform 1 of Protein kinase C delta type	72.85%	7	6	61	213
IPI00462502.3	Isoform 1 of Protein LAS1 homolog	73.71%	2	12	67	361
IPI00221690.1	Isoform 1 of Protein MAK16 homolog	55.07%	2	2	21	70
IPI00330176.4	Isoform 1 of Protein MON2 homolog	69.27%	3	7	121	480
IPI00136134.1	Isoform 1 of Protein NDRG2	69.27%	2	15	26	167
IPI00947566.1	Isoform 1 of Protein OS-9	69.20%	5	5	51	150
IPI00671847.3	Isoform 1 of Protein phosphatase 1 regulatory subunit 12A	77.65%	2	19	116	385
IPI00121349.3	Isoform 1 of Protein phosphatase 1 regulatory subunit 1B	80.93%	2	9	17	95
IPI00620040.3	Isoform 1 of Protein phosphatase 1H	63.74%	2	4	45	210
IPI00229072.2	Isoform 1 of Protein PML	62.49%	6	8	66	221
IPI00622482.2	Isoform 1 of Protein RUFY3	88.49%	9	16	66	240
IPI00313539.4	Isoform 1 of Protein SCAI	67.99%	2	8	51	112
IPI00454104.1	Isoform 1 of Protein scribble homolog	59.93%	4	10	103	310
IPI00133385.3	Isoform 1 of Protein syndesmos	57.82%	3	2	17	37
IPI00222225.2	Isoform 1 of Protein transport protein Sec24A	60.00%	4	6	58	227
IPI00938457.1	Isoform 1 of Protein transport protein Sec31A	66.34%	3	29	93	884
IPI00113244.3	Isoform 1 of Protein tweety homolog 1	58.22%	6	4	17	43
IPI00463681.2	Isoform 1 of Protein wntless homolog	55.08%	3	4	32	65
IPI00463765.1	Isoform 1 of Proto-oncogene tyrosine-protein kinase receptor Ret	62.96%	3	1	73	183
IPI00153880.4	Isoform 1 of Purine-rich element-binding protein gamma	68.29%	2	1	31	63
IPI00989798.1	Isoform 1 of Putative adenosylhomocysteinase 3	86.79%	9	2	65	330
IPI00165730.4	Isoform 1 of Putative phospholipase B-like 2	71.04%	2	4	37	109
IPI00410804.1	Isoform 1 of Putative RNA-binding protein Luc7-like 1	66.31%	4	4	41	282
IPI00380309.3	Isoform 1 of Putative RNA-binding protein Luc7-like 2	62.24%	8	8	54	350
IPI00122320.3	Isoform 1 of Quinone oxidoreductase-like protein 1	69.25%	6	5	27	87
IPI00122565.1	Isoform 1 of Rab GDP dissociation inhibitor beta	95.06%	4	24	58	977
IPI00378156.2	Isoform 1 of Rab GTPase-activating protein 1	59.77%	3	6	81	182
IPI00119795.2	Isoform 1 of RalBP1-associated Eps domain-containing protein 1	50.82%	5	3	47	124
IPI00461277.4	Isoform 1 of Rap1 GTPase-activating protein 2	58.43%	2	8	49	163
IPI00468702.1	Isoform 1 of Ras-related GTP-binding protein C	75.63%	5	10	31	144
IPI00116697.1	Isoform 1 of Ras-related protein Rab-6A	89.90%	8	1	36	285
IPI00396701.3	Isoform 1 of Ras-related protein Rap-2a	87.98%	2	2	14	68
IPI00108685.4	Isoform 1 of Receptor-type tyrosine-protein phosphatase alpha	73.82%	3	4	73	284
IPI00454123.4	Isoform 1 of Regulation of nuclear pre-mRNA domain-containing protein 2	62.70%	2	4	98	353
**IPI00221840.1**	**Isoform 1 of Regulator of differentiation 1**	**74.00%**	**8**	**8**	**46**	**521**
IPI00420949.3	Isoform 1 of Regulator of nonsense transcripts 1	77.05%	2	33	109	709
IPI00469392.2	Isoform 1 of Reticulon-4	76.25%	4	29	87	937
IPI00226384.2	Isoform 1 of Retinoblastoma-binding protein 5	75.84%	2	4	46	115
IPI00320905.7	Isoform 1 of Rho GTPase-activating protein 17	65.37%	8	6	55	167
IPI00310764.8	Isoform 1 of Rho guanine nucleotide exchange factor 2	86.19%	7	31	129	642
IPI00654133.1	Isoform 1 of Rho guanine nucleotide exchange factor 40	62.10%	6	3	111	463
IPI00108147.1	Isoform 1 of Rho-associated protein kinase 1	79.39%	2	10	174	571
IPI01027776.1	Isoform 1 of Rho-associated protein kinase 2	84.01%	4	31	188	831
IPI00719915.1	Isoform 1 of Ribonucleoprotein PTB-binding 1	73.66%	2	9	52	251
IPI00320878.5	Isoform 1 of RING finger protein 121	48.62%	2	1	10	27
IPI00223425.2	Isoform 1 of RNA binding protein fox-1 homolog 2	59.69%	25	5	25	128
IPI00338904.1	Isoform 1 of RNA polymerase II subunit A C-terminal domain phosphatase	75.73%	4	6	80	216
IPI00120919.4	Isoform 1 of RNA polymerase-associated protein CTR9 homolog	58.65%	4	6	90	226
IPI00474486.5	Isoform 1 of RNA polymerase-associated protein LEO1	68.67%	2	5	49	123
IPI00404707.1	Isoform 1 of RNA-binding protein 14	60.39%	5	11	48	445
IPI00828892.2	Isoform 1 of RNA-binding protein 27	61.13%	3	2	84	256
IPI00128377.2	Isoform 1 of RNA-binding protein 47	53.90%	7	6	32	91
IPI00308402.2	Isoform 1 of RNA-binding protein Raly	95.27%	5	16	49	583
IPI00462424.1	Isoform 1 of RNA-binding protein with serine-rich domain 1	77.70%	3	5	40	170
IPI00229917.4	Isoform 1 of Rootletin	86.91%	3	4	312	1071
IPI00224219.1	Isoform 1 of Sec1 family domain-containing protein 1	91.86%	4	15	73	485
IPI00830189.2	Isoform 1 of Selenocysteine lyase	82.64%	7	3	42	173
IPI00123219.1	Isoform 1 of Septin-4	64.64%	11	6	41	200
IPI00457611.2	Isoform 1 of Septin-9	95.71%	6	26	87	852
IPI00133374.5	Isoform 1 of Sequestosome-1	72.40%	3	2	21	232
IPI00605037.2	Isoform 1 of Serine/arginine repetitive matrix protein 1	76.74%	8	10	150	575
IPI00785240.2	Isoform 1 of Serine/arginine repetitive matrix protein 2	86.27%	3	45	390	1775
IPI00420807.3	Isoform 1 of Serine/arginine-rich splicing factor 1	94.35%	6	15	54	440
IPI00990689.1	Isoform 1 of Serine/arginine-rich splicing factor 10	73.66%	6	5	43	136
IPI00474228.2	Isoform 1 of Serine/threonine-protein kinase 3	67.40%	3	4	40	105
IPI00515701.1	Isoform 1 of Serine/threonine-protein kinase BRSK1	70.82%	6	4	52	139
IPI00468380.4	Isoform 1 of Serine/threonine-protein kinase DCLK1	78.57%	10	14	77	251
IPI00268673.6	Isoform 1 of Serine/threonine-protein kinase mTOR	78.82%	1	13	227	969
IPI00762427.1	Isoform 1 of Serine/threonine-protein kinase N1	65.01%	3	4	88	302
IPI00312414.5	Isoform 1 of Serine/threonine-protein kinase N2	72.02%	7	5	95	337
IPI00132936.1	Isoform 1 of Serine/threonine-protein kinase VRK1	82.27%	4	4	51	130
IPI00319816.3	Isoform 1 of Serine/threonine-protein kinase VRK2	69.58%	2	1	36	78
IPI00121545.1	Isoform 1 of Serine/threonine-protein phosphatase 2B catalytic subunit alpl	89.64%	4	1	49	441
IPI00169960.3	Isoform 1 of Shootin-1	85.90%	3	12	84	301
IPI00111794.3	Isoform 1 of Sialate O-acetylesterase	76.89%	3	3	38	111
IPI00126115.1	Isoform 1 of Sideroflexin-3	84.42%	3	10	36	257
IPI00387377.1	Isoform 1 of Signal recognition particle 54 kDa protein	93.65%	3	6	77	454
IPI00468418.2	Isoform 1 of Signal transducing adapter molecule 2	59.27%	2	5	29	109
IPI00317401.6	Isoform 1 of Sister chromatid cohesion protein PDS5 homolog B	79.53%	3	22	157	578
IPI00877254.1	Isoform 1 of Solute carrier family 12 member 5	62.39%	5	20	86	356
IPI00331175.5	Isoform 1 of Solute carrier family 12 member 7	64.73%	4	12	80	266
IPI00119776.1	Isoform 1 of Something about silencing protein 10	55.44%	2	7	36	268

IPI00111320.7	Isoform 1 of Sorting nexin-27	63.64%	4	8	45	192							-0.342	-0.229	0.431	0.352	-0.324	3													-0.116	0.004	0.087	0.021	-0.001	10	539	61.0	6.40
IPI00153501.5	Isoform 1 of Spartin	56.93%	5	7	51	143							0.023	-0.048	-0.150	0.099	0.471	4																			671	72.6	5.86
IPI00753815.3	Isoform 1 of Spectrin alpha chain, brain	94.70%	4	1	385	5053	0.043	0.058	0.083	0.010	0.048	234	0.450	0.029	0.474	0.051	0.367	256	-0.023	0.037	-0.044	-0.023	-0.127	219	-0.257	0.009	-0.248	0.007	-0.269	264	0.013	0.068	0.085	0.041	0.055	643	2472	284.4	5.33
IPI00319830.7	Isoform 1 of Spectrin beta chain, brain 1	93.10%	4	10	316	3955	0.011	0.023	0.009	-0.013	0.004	191	0.433	0.026	0.439	0.083	0.423	193	-0.045	0.028	-0.071	-0.028	-0.133	172	-0.272	0.031	-0.233	0.029	-0.267	178	0.045	0.120	0.084	0.100	0.092	478	2363	274.1	5.58
IPI00128254.2	Isoform 1 of Spermatid perinuclear RNA-binding protein	79.32%	3	2	69	223																			0.152	-0.006	0.111	0.001	0.135	3							672	73.7	8.72
IPI00169793.1	Isoform 1 of Spermatogenesis-associated serine-rich protein 2	83.49%	2	4	63	228							-0.213	-0.194	-0.358	0.157	-0.474	6																			545	58.9	9.00
IPI00122011.2	Isoform 1 of Splicing factor 3B subunit 3	81.68%	2	37	116	671	-0.044	0.064	-0.144	-0.038	-0.112	37	-0.105	0.084	-0.122	0.038	-0.131	43	0.047	-0.007	0.069	0.036	-0.009	32	0.321	-0.006	0.339	-0.031	0.262	48							1217	135.5	5.26
IPI00458854.2	Isoform 1 of Squamous cell carcinoma antigen recognized by T-cells 3	62.47%	2	12	84	330	0.009	0.027	-0.023	-0.063	0.068	6	0.108	0.166	-0.191	-0.020	-0.069	9	-0.241	0.084	-0.127	-0.065	-0.181	4													962	109.5	5.24
IPI00318935.2	Isoform 1 of SRA stem-loop-interacting RNA-binding protein, mitochondria	84.82%	3	3	9	40	-0.183	-0.041	0.163	0.154	-0.292	7							0.262	0.077	0.204	-0.020	0.026	4													112	12.6	9.82
IPI00331076.4	Isoform 1 of STE20-like serine/threonine-protein kinase	84.67%	7	28	129	529	0.079	-0.035	0.181	0.007	0.122	29	0.031	-0.108	-0.017	-0.029	-0.056	11	-0.281	-0.021	-0.068	0.102	-0.042	8													1233	141.4	5.14
IPI00153862.2	Isoform 1 of Sulfotransferase 4A1	43.66%	2	4	15	25																									0.013	-0.099	-0.094	-0.037	-0.032	7	284	33.0	5.53
IPI00129105.2	Isoform 1 of SUMO-activating enzyme subunit 1	84.86%	2	18	41	438	-0.034	-0.069	-0.006	0.068	-0.035	31	-0.206	-0.095	-0.181	-0.119	-0.231	37	-0.120	-0.184	-0.264	0.099	0.077	12	0.581	0.127	0.668	0.061	0.506	49	0.057	0.073	0.061	0.047	-0.054	14	350	38.6	5.36
IPI00263505.1	Isoform 1 of SUN domain-containing protein 1	73.60%	14	6	70	208							0.204	-0.023	0.102	0.280	-0.083	10																			913	101.9	6.81
IPI00460291.2	Isoform 1 of SUN domain-containing protein 2	75.24%	5	18	73	309	0.038	0.140	0.001	-0.004	0.152	16	0.246	-0.056	0.321	0.045	0.234	19	0.187	0.015	0.170	-0.060	0.001	23													731	81.6	7.02
IPI00556837.1	Isoform 1 of SWI/SNF-related matrix-associated actin-dependent regulator (	71.89%	3	2	81	218	-0.236	-0.130	-0.213	-0.189	0.045	4																									1021	116.4	5.68
IPI00649685.3	Isoform 1 of SWI/SNF-related matrix-associated actin-dependent regulator (	77.78%	3	3	42	153													0.451	0.195	0.066	-0.168	-0.150	3	-0.138	-0.327	-0.094	-0.175	-0.281	10							531	59.0	9.64
IPI00458153.2	Isoform 1 of Swi5-dependent recombination DNA repair protein 1 homolog	72.73%	3	4	27	177	0.171	0.153	0.479	0.072	-0.032	9	-0.255	-0.196	-0.141	-0.123	-0.317	13																			319	35.2	5.15
IPI00269408.1	Isoform 1 of Synaptosomal-associated protein 47	89.35%	6	9	45	208																			-0.120	-0.039	-0.324	-0.044	-0.252	8	-0.142	-0.173	-0.342	-0.278	-0.056	19	413	46.5	5.76
IPI00115450.1	Isoform 1 of Syndecan-1	62.06%	3	5	12	47	-0.053	-0.079	-0.221	-0.062	-0.131	8																									311	32.9	4.79
IPI00785417.1	Isoform 1 of Syntaxin-5	89.86%	2	3	40	122																			-0.213	-0.180	-0.271	-0.154	-0.165	5							355	39.7	8.92
IPI00415402.3	Isoform 1 of Syntaxin-binding protein 1	96.97%	2	3	87	639																									0.054	0.026	0.040	-0.032	-0.014	225	594	67.5	6.96
IPI00624098.1	Isoform 1 of Syntaxin-binding protein 3	86.66%	3	6	64	179	-0.181	-0.117	0.011	-0.059	0.108	5	-0.165	0.010	-0.189	0.034	-0.203	11																			592	67.9	8.02
IPI00555114.1	Isoform 1 of Syntaxin-binding protein 5-like	55.70%	7	5	57	133																									-0.034	-0.088	-0.150	0.337	-0.234	6	1185	131.8	6.86
IPI00380814.1	Isoform 1 of Target of Myb protein 1	70.93%	2	6	30	123																									0.048	-0.130	0.089	-0.020	-0.194	10	492	54.3	4.94
IPI00172147.2	Isoform 1 of TBC1 domain family member 23	52.49%	1	2	35	71	0.000	0.013	-0.288	-0.047	-0.139	3																									684	76.4	5.36
IPI00761259.1	Isoform 1 of TBC1 domain family member 24	63.46%	3	3	49	105																									0.309	0.157	-0.128	0.375	-0.170	5	561	63.2	7.24
IPI00459493.5	Isoform 1 of T-complex protein 1 subunit alpha	96.40%	4	35	81	1464	-0.114	-0.051	-0.125	-0.012	-0.131	100	0.140	0.029	0.169	0.033	0.128	83	-0.163	-0.107	-0.088	0.038	0.006	62	0.023	0.004	0.001	-0.014	0.098	118	-0.155	-0.082	-0.149	-0.188	-0.168	38	556	60.4	6.16
IPI00626173.3	Isoform 1 of Telomere-associated protein RIF1	81.11%	3	7	237	845							0.270	0.051	0.161	0.234	0.474	4																			2419	266.1	5.57
IPI00403938.2	Isoform 1 of Tenascin	63.32%	8	4	112	283																									0.124	-0.023	0.141	0.199	-0.083	5	2110	231.7	4.89
IPI00227126.2	Isoform 1 of Tenascin-R	67.75%	2	31	82	387																									0.089	0.088	0.038	0.087	0.111	109	1358	149.5	4.94
IPI00353237.8	Isoform 1 of Tensin-3	66.32%	2	13	96	510	-0.013	-0.079	0.061	-0.116	0.089	12	0.224	0.083	0.578	-0.155	0.157	15	-0.100	-0.156	0.190	0.165	0.293	3													1440	155.5	6.65
IPI00121364.2	Isoform 1 of Tether containing UBX domain for GLUT4	72.36%	10	6	49	162							-0.351	-0.128	-0.521	-0.223	-0.074	3																			550	59.8	6.96
IPI00341643.2	Isoform 1 of Tetratricopeptide repeat protein 39B	68.56%	5	6	46	114	-0.119	-0.025	-0.193	-0.134	-0.149	5																									617	70.2	6.64
IPI00153448.3	Isoform 1 of Thioesterase superfamily member 4	53.91%	3	4	19	68																									0.143	-0.183	0.013	-0.370	-0.224	9	230	26.0	9.64
IPI00776252.1	Isoform 1 of Thioredoxin reductase 1, cytoplasmic	88.74%	5	19	71	522	0.048	0.160	-0.069	0.006	-0.010	11	-0.218	0.075	-0.109	-0.184	-0.162	22	-0.506	-0.109	-0.388	0.140	-0.228	21	-0.152	0.031	-0.297	0.019	-0.138	33	-0.042	-0.089	-0.012	-0.003	-0.037	16	613	67.0	7.43
IPI00114407.2	Isoform 1 of THO complex subunit 4	97.25%	7	9	44	535	-0.085	0.023	-0.071	-0.016	-0.088	20	0.055	0.038	0.044	0.030	-0.045	21	0.192	0.038	0.169	0.037	0.139	34	-0.311	-0.054	-0.218	-0.026	-0.142	37	-0.188	0.118	0.011	0.088	-0.082	6	255	26.9	11.15
IPI00756257.1	Isoform 1 of Titin	72.67%	7	33	3142	10196	-0.006	0.016	0.020	-0.042	0.108	6	-0.206	-0.170	-0.458	-0.208	-0.144	6	-0.184	0.009	-0.245	-0.120	-0.141	24	0.185	0.077	0.131	0.027	0.128	6	-0.144	0.003	-0.095	-0.014	-0.031	6	35213	3904.1	6.20
IPI00322033.7	Isoform 1 of TOM1-like protein 2	71.40%	7	10	35	104																									0.055	0.108	-0.010	0.010	0.095	23	507	55.6	4.82
IPI00955124.1	Isoform 1 of Transcription activator BRG1	63.48%	7	24	145	572	-0.001	-0.017	0.112	-0.080	-0.054	16	-0.035	0.064	-0.003	0.052	0.058	16	0.187	0.079	0.207	0.034	0.102	16	-0.048	0.007	-0.014	-0.032	0.057	18							1613	181.3	8.00
IPI00656221.1	Isoform 1 of Transcription elongation factor SPT5	71.35%	2	19	92	464	-0.094	0.062	-0.097	-0.069	-0.080	11	0.220	0.013	0.163	-0.097	-0.112	23							-0.160	-0.122	-0.007	-0.025	-0.047	13							1082	120.6	5.05
IPI00312128.3	Isoform 1 of Transcription intermediary factor 1-beta	87.05%	2	29	81	849	0.026	0.052	-0.076	-0.017	-0.063	53	-0.186	-0.039	-0.127	0.016	-0.176	60	0.089	-0.002	0.029	0.041	0.085	39	-0.142	-0.138	-0.103	-0.062	-0.112	49	0.038	0.089	0.250	-0.045	0.106	9	834	88.8	5.77
IPI00128615.1	Isoform 1 of Transcriptional repressor p66-beta	84.85%	2	11	73	428	-0.022	0.007	-0.229	-0.134	-0.168	11	0.057	0.050	0.031	0.106	-0.060	7	-0.320	-0.071	-0.116	0.124	0.058	6													594	65.4	9.70
IPI00139259.1	Isoform 1 of Transformer-2 protein homolog beta	84.72%	2	6	45	168							0.118	0.066	0.448	0.397	-0.181	3	0.160	0.044	0.088	-0.099	0.048	4	-0.077	-0.255	-0.069	-0.175	-0.048	11							288	33.6	11.25
IPI00120826.1	Isoform 1 of Translocon-associated protein subunit gamma	68.11%	2	2	12	57							0.282	-0.340	0.148	0.214	0.126	5																			185	21.1	9.61
IPI00224237.6	Isoform 1 of Transmembrane and TPR repeat-containing protein 3	70.87%	2	8	65	258							-0.092	0.116	-0.193	-0.035	-0.139	5	0.271	-0.007	0.069	-0.229	0.094	9	0.424	0.068	0.374	-0.032	0.329	4							920	104.1	8.53
IPI00466570.4	Isoform 1 of Transmembrane emp24 domain-containing protein 10	65.75%	4	9	20	270	0.277	0.066	0.180	0.034	0.131	34	0.518	0.145	0.408	0.017	0.393	12	0.474	0.106	0.317	0.048	0.210	22	0.084	0.057	-0.010	-0.003	-0.006	29	-0.136	-0.009	-0.035	-0.112	-0.171	8	219	24.9	6.70
IPI00222924.3	Isoform 1 of Transmembrane protein 214	78.46%	3	14	72	384	-0.086	0.157	0.145	0.023	0.069	15	0.439	0.057	0.331	0.099	0.238	9	0.249	0.065	0.256	-0.029	0.102	10	0.299	-0.066	0.377	-0.255	-0.026	3							687	76.4	9.29
IPI00221523.4	Isoform 1 of Transportin-1	74.05%	3	19	60	551	0.076	0.061	0.030	0.059	0.026	26	-0.089	0.059	-0.012	-0.038	-0.023	28	0.029	-0.024	0.103	-0.066	-0.001	17	0.000	0.057	0.036	0.006	0.001	29							898	102.3	4.98
IPI00226311.2	Isoform 1 of Trinucleotide repeat-containing gene 6B protein	64.03%	2	6	117	516	0.027	0.167	0.016	-0.086	-0.041	5																									1810	191.8	6.30
IPI00459632.4	Isoform 1 of TRMT1-like protein	77.88%	3	5	69	201							-0.504	-0.340	-0.286	0.110	0.096	3																			728	80.8	7.50
IPI00894870.1	Isoform 1 of tRNA (cytosine(34)-C(5))-methyltransferase	88.77%	2	25	78	487	-0.044	0.081	0.054	0.057	-0.012	26	-0.041	0.112	0.102	0.059	0.023	26	0.048	0.228	0.069	0.102	0.126	10	0.161	0.027	0.120	0.048	0.102	45							757	85.4	6.58
IPI00129184.3	Isoform 1 of tRNA-dihydrouridine synthase 3-like	76.14%	3	4	51	179																			0.203	0.167	0.456	0.076	0.338	6							637	71.0	7.97
IPI00402982.2	Isoform 1 of Tropomodulin-2	91.17%	3	16	43	216																			-0.019	-0.123	0.122	0.011	0.197	3	0.000	0.029	-0.032	-0.097	0.003	48	351	39.5	5.35
IPI00278611.7	Isoform 1 of Tropomyosin alpha-3 chain	92.96%	4	2	60	777	0.013	0.059	0.103	0.062	0.050	59	0.336	0.017	0.258	-0.020	0.119	57	-0.089	-0.069	-0.029	0.030	0.051	54	0.135	0.026	0.199	0.017	0.116	29	0.058	0.100	0.131	0.143	0.152	30	284	32.8	4.72
IPI00123319.1	Isoform 1 of Tropomyosin beta chain	90.85%	4	3	51	653	-0.007	0.088	0.115	0.059	0.026	45	0.041	-0.071	-0.109	-0.056	-0.017	54	-0.281	-0.101	-0.173	-0.031	-0.076	86	0.162	0.014	0.210	0.009	0.112	14	0.003	-0.086	0.041	-0.007	0.027	22	284	32.8	4.70
IPI00923656.1	Isoform 1 of Tyrosine-protein kinase BAZ1B	74.78%	2	32	166	702	0.104	-0.001	0.011	-0.142	0.021	17	-0.114	-0.014	-0.083	-0.063	0.010	36	0.162	0.028	0.341	-0.082	0.227	8	0.120	-0.065	0.229	-0.097	0.077	17							1479	170.5	8.60
IPI00464166.2	Isoform 1 of Tyrosine-protein phosphatase non-receptor type 23	53.78%	2	6	123	475	0.001	-0.063	-0.006	-0.099	-0.045	4	0.193	-0.029	0.295	-0.010	0.102	7																			1692	185.1	6.80
IPI00625105.2	Isoform 1 of U1 small nuclear ribonucleoprotein 70 kDa	58.48%	4	14	38	388	0.059	0.058	0.076	0.023	0.027	28	0.157	0.115	0.188	0.154	0.034	30	0.385	0.168	0.277	0.061	0.188	33	0.017	0.016	0.246	0.013	0.171	17	-0.160	0.123	0.330	0.318	0.158	6	448	52.0	9.94
IPI00830853.2	Isoform 1 of U2 snRNP-associated SURP motif-containing protein	72.59%	2	1	104	300	-0.127	-0.016	0.036	-0.209	-0.136	9	-0.127	-0.042	-0.288	-0.134	-0.336	5																			1029	118.2	8.47
IPI00462762.2	Isoform 1 of U3 small nucleolar RNA-associated protein 14 homolog A	61.80%	2	12	69	237	-0.022	-0.012	-0.213	0.059	-0.109	14	0.500	0.280	0.031	-0.025	0.152	3	-0.048	0.186	0.198	-0.003	0.122	4	-0.060	0.043	-0.053	-0.091	-0.216	3							767	87.2	9.20
IPI00153642.1	Isoform 1 of Ubiquilin-1	56.70%	1	2	31	1117	0.198	0.062	0.269	0.102	0.108	34	-0.007	0.045	-0.178	-0.051	-0.105	33	-0.108	-0.131	0.116	-0.079	0.124	10	-0.240	0.052	-0.269	-0.003	-0.180	70	0.031	0.052	-0.053	-0.066	-0.134	13	582	61.9	4.94
IPI00154012.1	Isoform 1 of Ubiquitin carboxyl-terminal hydrolase 15	71.36%	5	15	76	218	0.069	0.027	-0.071	0.108	0.170	7	0.003	0.024	-0.146	-0.007	-0.199	5	-0.292	-0.019	-0.077	0.089	-0.062	4	0.037	-0.010	-0.103	0.011	0.054	7							981	112.3	5.17
IPI00608091.1	Isoform 1 of Ubiquitin carboxyl-terminal hydrolase 47	67.01%	3	16	114	403	0.135	-0.088	-0.009	-0.125	0.178	11	-0.175	-0.097	-0.056	-0.047	0.036	10							-0.006	0.110	-0.056	0.151	0.010	6							1376	157.4	5.11
IPI00124938.1	Isoform 1 of Ubiquitin carboxyl-terminal hydrolase isozyme L5	98.18%	1	1	35	223	-0.080	0.000	-0.089	-0.010	-0.142	25	0.068	-0.051	0.088	-0.050	0.055	12	-0.276	-0.089	-0.103	-0.053	0.072	11	0.080	0.055	0.006	0.059	0.017	12							329	37.6	5.33
IPI00761937.1	Isoform 1 of Ubiquitin-associated protein 2-like	63.05%	7	2	63	1313	0.014	-0.026	-0.051	-0.034	0.024	64	0.000	-0.062	-0.053	-0.029	-0.162	81	0.070	0.023	0.114	-0.028	0.218	53	0.023	-0.010	0.040	0.004	0.065	33	-0.188	-0.152	-0.216	-0.203	-0.116	8	1107	116.7	7.11
IPI00113257.3	Isoform 1 of Ubiquitin-conjugating enzyme E2 variant 1	96.60%	6	3	25	243	0.088	-0.059	0.181	0.092	0.107	10	-0.892	-0.133	-0.865	-0.278	-0.749	12	-0.335	-0.112	-0.285	0.151	-0.108	14							0.000	-0.072	0.021	0.045	0.073	20	147	16.3	7.96
IPI00402913.1	Isoform 1 of Ubiquitin-conjugating enzyme E2 variant 2	100.00%	5	5	26	250	0.048	-0.042	0.085	0.112	0.038	9	-0.938	-0.260	-0.419	-0.466	-0.444	7	-0.375	-0.216	-0.233	0.134	-0.313	3	0.051	0.098	0.158	0.127	0.193	4	-0.026	-0.032	0.052	0.048	0.057	27	145	16.4	8.09
IPI00121151.1	Isoform 1 of UBX domain-containing protein 6	82.35%	3	4	55	137																									-0.178	0.043	-0.029	-0.009	-0.029	3	442	49.8	8.54
**IPI00420870.4**	**Isoform 1 of UDP-N-acetylglucosamine--peptide N-acetylglucosaminyltransf**	**71.89%**	**2**	**17**	**64**	**332**	-0.034	-0.020	0.169	-0.019	0.011	9	-0.241	-0.131	-0.184	-0.257	-0.025	6	0.782	0.283	0.275	0.455	0.940	3							-0.131	-0.117	-0.083	-0.125	0.103	11	**1046**	**116.9**	**6.70**
IPI00462482.3	Isoform 1 of UDP-N-acetylhexosamine pyrophosphorylase-like protein 1	65.29%	3	6	37	184	0.087	-0.077	0.017	-0.091	0.271	15	-0.336	-0.299	-0.238	-0.377	-0.059	4																			507	56.6	5.43
IPI00226932.2	Isoform 1 of Uncharacterized protein KIAA0090	79.44%	2	1	80	446	-0.057	0.122	0.170	0.064	-0.059	24	-0.390	-0.029	-0.186	0.079	-0.007	11	0.040	0.072	0.081	0.031	0.024	14	-0.580	-0.060	-0.415	-0.013	-0.383	13							997	111.5	7.43
IPI00132352.2	Isoform 1 of UPF0368 protein Cxorf26 homolog	74.24%	2	9	22	84	0.131	-0.128	0.166	-0.013	0.234	9	-0.234	-0.127	-0.292	-0.243	0.135	5	-0.411	-0.097	-0.224	-0.059	-0.245	3	0.000	-0.062	-0.014	0.017	-0.074	3							198	22.2	4.55
IPI00989838.1	Isoform 1 of UPF0505 protein C16orf62 homolog	60.96%	6	4	65	140	-0.092	0.059	-0.309	-0.029	-0.130	4																									963	109.0	7.31
IPI00132883.1	Isoform 1 of UPF0534 protein C4orf43 homolog	75.11%	2	3	23	131							0.346	0.300	0.415	0.139	-0.295	4							-0.181	-0.069	-0.037	-0.117	-0.186	14							221	25.8	9.19
IPI00225796.3	Isoform 1 of UPF0554 protein C2orf43 homolog	75.46%	5	3	26	81	-0.262	0.033	-0.006	-0.037	-0.297	4							0.444	0.071	0.299	0.285	0.314	5													326	37.3	8.28
IPI00118475.1	Isoform 1 of Urokinase plasminogen activator surface receptor	60.24%	2	2	17	24							0.523	-0.102	0.251	0.040	0.440	3																			327	35.4	6.83
IPI00131204.1	Isoform 1 of UTP--glucose-1-phosphate uridylyltransferase	87.80%	2	15	60	384	-0.175	-0.154	-0.152	-0.080	-0.116	6	0.044	-0.128	0.136	0.001	0.110	8	-0.141	-0.094	-0.130	0.037	0.024	6	0.302	0.029	0.280	0.030	0.303	28	0.019	-0.034	-0.069	-0.083	-0.083	12	508	56.9	7.61
IPI00551411.5	Isoform 1 of Vacuolar protein sorting-associated protein 13C	78.34%	7	8	377	1314																									-0.065	0.208	0.279	-0.134	0.076	3	3748	419.8	6.81
IPI00223759.2	Isoform 1 of Vacuolar protein sorting-associated protein 26B	72.32%	2	11	34	141							0.100	0.167	0.228	0.119	0.229	3							-0.183	-0.199	-0.158	-0.184	-0.057	5	-0.311	-0.141	-0.042	-0.022	0.033	21	336	39.1	7.37
IPI00136936.1	Isoform 1 of Vacuolar protein sorting-associated protein 29	95.05%	5	8	19	228	0.108	-0.079	0.116	-0.004	0.134	11	0.007	0.064	-0.037	0.072	-0.041	12	-0.045	-0.054	0.037	0.041	-0.051	14	-0.204	-0.022	-0.050	-0.085	-0.068	8	-0.184	-0.014	-0.120	-0.109	-0.102	19	182	20.5	6.79
IPI00153241.1	Isoform 1 of Vacuolar protein sorting-associated protein 37C	47.73%	1	2	14	35							-0.069	0.061	0.069	0.066	0.099	4																			352	38.4	5.31
IPI00623259.1	Isoform 1 of Vacuolar protein sorting-associated protein 52 homolog	62.93%	3	3	55	167																									0.080	0.143	0.020	0.247	0.333	4	723	82.0	5.90
IPI00132167.1	Isoform 1 of Vacuolar protein-sorting-associated protein 25	64.20%	6	5	12	90																			-0.421	0.043	-0.429	-0.120	-0.338	14							176	20.7	6.34
IPI00457578.2	Isoform 1 of Villin-like protein	65.54%	5	5	66	327	0.353	-0.057	0.145	-0.037	0.236	8																									859	96.4	6.35
IPI00349521.2	Isoform 1 of Vomeromodulin	65.99%	3	2	48	170	-0.178	-0.260	-0.170	-0.097	-0.502	4																									591	62.4	5.68
IPI00407692.3	Isoform 1 of V-type proton ATPase catalytic subunit A	90.76%	4	35	70	759	0.172	0.045	0.195	0.031	0.139	30	-0.167	0.020	-0.114	0.076	-0.020	26	-0.265	-0.128	-0.276	0.135	-0.366	16	-0.342	-0.003	-0.313	-0.010	-0.214	43	0.091	-0.271	-0.231	-0.269	-0.204	175	617	68.3	5.58
IPI00336198.1	Isoform 1 of WD repeat-containing protein 48	69.23%	3	4	54	139	-0.271	-0.056	-0.255	-0.127	-0.111	3																									676	76.0	7.17
IPI00120637.5	Isoform 1 of WD repeat-containing protein 7	68.91%	2	20	109	381																									-0.020	-0.109	-0.131	-0.048	-0.062	48	1489	163.3	6.90
IPI00331342.6	Isoform 1 of WD40 repeat-containing protein SMU1	75.83%	2	8	47	219	0.122	0.290	0.070	0.044	0.031	7	-0.133	-0.054	-0.102	0.033	-0.272	5	0.070	0.033	0.108	-0.074	-0.020	13	0.116	0.167	0.376	-0.085	0.225	4							513	57.5	7.18
IPI00221632.3	Isoform 1 of Y+L amino acid transporter 2	38.64%	2	2	19	79	0.076	0.084	0.174	0.087	0.110	3	0.647	-0.193	0.269	0.095	-0.250	3																			515	56.7	7.30
IPI00461416.4	Isoform 1 of Zinc finger CCCH domain-containing protein 14	84.22%	3	8	70	273	0.040	0.134	0.014	0.011	0.010	10	0.134	0.153	-0.019	-0.069	0.007	10	0.341	0.156	0.308	0.052	0.354	4													735	82.4	7.37
IPI00336958.3	Isoform 1 of Zinc finger CCCH domain-containing protein 15	80.05%	3	16	42	358	-0.057	0.044	-0.017	0.001	0.013	31	0.073	-0.072	0.083	-0.051	0.070	21	-0.170	-0.034	-0.170	-0.103	-0.108	12	0.203	0.080	0.179	0.115	0.126	24							426	48.3	5.30
IPI00750931.1	Isoform 1 of Zinc finger CCCH domain-containing protein 4	47.16%	6	7	74	243	-0.160	-0.120	0.038	-0.047	0.058	10	-0.077	-0.059	-0.454	-0.300	-0.606	3																			1304	140.9	6.27
IPI00314507.6	Isoform 1 of Zinc finger protein 326	63.62%	4	8	38	237							-0.381	-0.138	0.023	0.033	-0.130	17	0.288	-0.034	0.059	-0.029	0.181	8	-0.218	-0.125	-0.150	-0.003	-0.336	5							580	65.2	5.19
IPI00123012.1	Isoform 1 of Zinc finger protein 330	60.13%	3	3	28	191	0.496	0.204	0.308	0.038	0.349	4	0.115	-0.272	-0.003	-0.186	-0.240	5							-0.131	-0.221	-0.229	-0.091	-0.160	6							316	35.6	6.16
IPI00134718.1	Isoform 1 of Zinc finger protein 687	55.38%	3	2	72	195																									-0.066	-0.035	0.023	0.050	-0.089	3	1237	130.3	8.12
IPI00400401.1	Isoform 1 of Zinc phosphodiesterase ELAC protein 2	78.22%	4	6	73	329	0.073	0.081	-0.102	0.059	-0.155	11	0.040	0.176	-0.001	0.215	0.383	7							-0.127	0.160	-0.191	0.019	-0.117	6							831	92.7	7.34
IPI00759913.1	Isoform 11 of Kinectin	89.11%	10	2	197	1126	-0.028	0.085	0.040	0.079	-0.054	36	0.294	-0.032	0.224	0.013	0.183	46	0.292	0.062	0.258	-0.138	0.231	47	0.148	-0.077	0.222	-0.006	0.136	52							1304	149.9	5.90
IPI00515160.2	Isoform 12 of Disintegrin and metalloproteinase domain-containing protein	76.09%	23	12	85	261																									-0.123	0.052	0.085	0.052	0.013	21	962	106.5	7.90
IPI00131084.4	Isoform 1A of Cytoplasmic dynein 1 intermediate chain 1	68.95%	6	7	33	126																									0.041	-0.133	-0.074	-0.139	-0.186	14	628	70.7	5.12
IPI00223325.5	Isoform 1A of Gamma-aminobutyric acid type B receptor subunit 1	67.92%	3	5	73	188																									0.052	-0.100	0.068	0.104	-0.001	9	960	108.1	8.21
IPI00468539.3	Isoform 1a of Neurexin-1-alpha	76.88%	8	5	121	411																									0.014	0.081	0.040	0.069	0.075	18	1514	166.1	6.09
IPI00761566.1	Isoform 1B of Beta-arrestin-1	72.93%	8	3	30	64	0.069	-0.063	0.034	0.040	0.126	4																									410	46.2	6.42
IPI00402981.2	Isoform 2 of 40S ribosomal protein S24	80.00%	5	6	21	215	0.050	-0.050	0.058	0.021	0.083	8	0.318	0.048	0.151	0.069	0.036	24	0.044	0.104	0.044	0.102	0.021	34	0.333	0.154	0.270	0.160	0.200	7	-0.068	-0.069	-0.050	0.151	0.017	13	130	15.1	10.89
IPI00668938.1	Isoform 2 of 5'-3' exoribonuclease 1	57.44%	3	6	117	380							-0.593	-0.442	-0.122	0.023	-0.038	4																			1706	192.9	7.50
IPI00762362.1	Isoform 2 of 60S ribosomal protein L22-like 1	78.51%	2	4	13	58	0.541	0.115	0.381	0.172	0.323	12							0.313	-0.062	0.414	-0.097	0.506	5													121	14.3	9.45
IPI00848443.1	Isoform 2 of Acetyl-CoA carboxylase 1	86.91%	8	22	228	832	0.070	-0.034	0.111	0.043	0.052	15	0.637	0.265	0.533	0.065	0.371	23	-0.112	0.004	0.059	-0.071	0.253	5	0.033	0.118	-0.108	0.034	-0.127	19							2383	269.8	6.51
IPI00648778.2	Isoform 2 of Activator of 90 kDa heat shock protein ATPase homolog 2	70.67%	2	1	35	127													-0.056	-0.168	-0.149	0.054	-0.079	3	-0.336	-0.010	-0.102	-0.004	-0.077	4							283	32.0	6.57
IPI00756908.2	Isoform 2 of Adenylate cyclase type 5	45.62%	4	3	68	141																									-0.029	0.096	0.230	0.138	0.100	3	1348	148.3	7.55
IPI00269076.3	Isoform 2 of Adenylate kinase 2, mitochondrial	94.40%	3	13	34	276	-0.022	0.061	-0.013	0.001	0.009	24	-0.238	-0.071	-0.281	-0.044	-0.411	14	-0.173	0.045	-0.218	-0.023	-0.146	41	0.518	-0.019	0.572	0.019	0.525	23	-0.007	0.194	0.019	0.154	-0.010	4	232	25.6	7.42
IPI00109656.2	Isoform 2 of ADP-ribosylation factor GTPase-activating protein 2	78.65%	3	13	58	209							-0.226	-0.125	-0.362	-0.051	-0.315	10	0.127	0.085	-0.117	0.099	0.077	6													534	58.1	8.28
IPI00109729.1	Isoform 2 of Alpha-crystallin A chain	61.27%	2	2	11	29							-1.130	-0.540	-0.591	-0.309	-1.065	4																			173	19.8	6.20
IPI00457597.2	Isoform 2 of Ankyrin repeat and sterile alpha motif domain-containing prote	60.09%	11	7	30	69																									0.081	0.143	0.197	0.202	0.098	16	426	48.3	7.97
IPI00227235.6	Isoform 2 of Ankyrin-2	76.95%	6	5	344	1629							0.123	-0.071	0.079	0.139	0.215	3													0.013	-0.037	-0.017	-0.051	-0.050	213	3926	428.5	5.16
IPI00356608.4	Isoform 2 of AP2-associated protein kinase 1	80.07%	4	26	76	362																									0.044	-0.111	-0.065	-0.080	-0.050	71	878	94.6	6.61
IPI00135626.2	Isoform 2 of Aquaporin-4	31.27%	4	6	11	52																									-0.041	-0.022	0.144	0.050	0.009	21	323	34.4	7.42
IPI00458001.1	Isoform 2 of Ataxin-2-like protein	59.87%	6	28	87	565	-0.016	-0.007	-0.045	0.047	-0.032	33	0.040	-0.037	-0.007	-0.106	-0.019	26	0.041	-0.002	-0.102	-0.178	0.026	26	-0.131	-0.074	-0.324	0.006	-0.155	17							1074	113.0	8.59
IPI00230633.1	Isoform 2 of Aurora kinase A	62.35%	2	3	32	62							-1.181	-0.086	-0.492	-0.515	-0.735	3																			417	47.1	9.36
IPI00113869.1	Isoform 2 of Basigin	83.15%	3	10	24	268	0.070	0.154	0.051	0.072	0.009	8	-0.396	-0.028	-0.440	0.126	-0.468	16	0.331	0.075	0.249	-0.009	0.102	32	0.558	-0.026	0.371	-0.048	0.411	11	0.011	-0.171	-0.060	0.083	0.033	11	273	29.7	5.54
IPI00128466.5	Isoform 2 of BRCA1-A complex subunit BRE	52.22%	8	3	28	82	0.283	0.094	-0.016	0.098	0.376	3													0.095	0.088	0.084	0.092	0.040	4							383	43.5	5.94
IPI00622700.2	Isoform 2 of Bromodomain-containing protein 2	65.43%	3	5	53	138	0.019	-0.013	0.010	0.108	-0.050	7																									752	83.2	9.11
IPI00845833.1	Isoform 2 of Calcineurin subunit B type 1	88.75%	2	10	21	146																									-0.050	-0.076	0.136	0.130	0.023	34	160	18.2	4.92
IPI00308813.1	Isoform 2 of Calcium uptake protein 1, mitochondrial	84.27%	4	5	65	284	0.190	0.229	0.241	0.188	0.193	9	0.367	-0.216	0.328	-0.388	0.019	8	0.642	0.226	0.315	0.092	0.614	4													483	55.1	8.32
IPI00480395.1	Isoform 2 of Calcium/calmodulin-dependent protein kinase type 1D	75.14%	4	4	42	173							-0.400	-0.065	-0.063	-0.119	-0.144	4							-0.559	0.054	-0.546	0.075	-0.529	6	0.269	0.120	0.013	-0.089	-0.057	19	362	41.1	6.74
IPI00330163.5	Isoform 2 of Calcium-dependent secretion activator 1	78.32%	2	1	124	610																									0.026	0.031	-0.007	-0.010	0.013	109	1361	153.9	5.86
IPI00410748.1	Isoform 2 of CCR4-NOT transcription complex subunit 2	92.84%	8	10	46	396	0.185	0.161	0.161	0.098	0.306	5	-0.357	0.126	-0.290	0.054	-0.035	7	0.077	-0.077	0.115	0.629	0.315	5	0.013	-0.074	-0.080	0.003	0.050	13							531	58.6	7.37
IPI00265503.5	Isoform 2 of CD44 antigen	42.26%	26	7	26	270	-0.031	-0.034	-0.142	0.080	-0.035	13	0.024	-0.138	-0.063	-0.138	-0.108	10	0.456	0.127	0.441	-0.051	0.285	47													736	81.0	4.88
IPI00808628.1	Isoform 2 of Cdc42-interacting protein 4	67.94%	4	5	64	197							-0.065	-0.364	-0.236	-0.167	-0.392	4																			602	68.4	5.90
IPI00453537.2	Isoform 2 of Cell adhesion molecule 2	60.89%	4	8	30	125																									0.085	0.112	0.062	0.077	-0.004	32	404	44.4	5.64
IPI00169896.2	Isoform 2 of Choline transporter-like protein 2	56.39%	1	1	43	97													0.030	0.031	-0.128	-0.076	-0.112	4							0.068	-0.131	-0.388	-0.063	-0.089	3	704	79.9	8.66
IPI01008121.2	Isoform 2 of Chondroitin sulfate proteoglycan 5	32.47%	4	5	13	34																									0.153	0.059	-0.069	0.000	-0.150	9	539	57.3	4.44
IPI00648479.2	Isoform 2 of Coiled-coil domain-containing protein 47	67.92%	5	15	43	321	-0.031	0.134	-0.082	0.006	-0.134	20	0.239	0.029	0.238	0.029	0.269	22	-0.028	0.063	0.145	-0.206	-0.103	12	-0.116	0.003	-0.178	-0.019	-0.054	14							477	54.8	4.72
IPI00228912.1	Isoform 2 of Collagen type IV alpha-3-binding protein	61.54%	3	5	45	109	0.436	-0.228	0.357	0.010	0.518	4							-0.112	-0.086	0.064	-0.388	-0.031	5													598	68.2	5.43
IPI00172226.3	Isoform 2 of Condensin complex subunit 1	85.83%	2	1	132	583	-0.056	0.021	-0.085	-0.026	-0.120	22	0.077	0.019	0.081	0.087	0.000	33							0.557	0.013	0.687	-0.109	0.715	5							1404	156.8	6.10
IPI00317701.2	Isoform 2 of COP9 signalosome complex subunit 7a	96.75%	5	7	33	212	0.197	-0.091	0.162	0.163	0.239	6							-0.146	0.255	0.016	-0.057	0.235	3	-0.188	-0.060	-0.246	-0.106	-0.317	12	-0.241	-0.219	-0.240	-0.326	-0.292	6	277	30.4	7.40
IPI00229487.1	Isoform 2 of Core-binding factor subunit beta	79.67%	4	3	24	90	-0.071	0.096	-0.109	-0.053	-0.074	5							0.132	0.119	-0.072	0.189	0.059	5	0.192	-0.020	0.417	-0.010	0.153	9							182	21.5	6.80
IPI00856974.1	Isoform 2 of C-terminal-binding protein 2	73.58%	4	4	78	224													-0.258	-0.155	-0.184	-0.292	0.048	3							0.003	-0.322	-0.155	0.033	-0.146	5	988	107.1	7.62
IPI00620823.2	Isoform 2 of Cyclin-G-associated kinase	58.92%	5	9	94	313	0.256	0.143	0.322	0.055	0.236	10	-0.417	-0.180	-0.157	-0.083	-0.429	6													0.194	0.296	0.128	-0.077	-0.028	9	1256	138.3	5.96
IPI00410740.1	Isoform 2 of Cysteinyl-tRNA synthetase, cytoplasmic	81.02%	2	1	91	588	-0.088	0.023	-0.012	0.010	-0.044	50	0.040	0.124	0.099	0.160	0.120	53	-0.327	-0.233	-0.229	-0.025	-0.106	13	-0.357	-0.029	-0.292	-0.009	-0.279	23							748	85.5	6.76
IPI00267596.1	Isoform 2 of Cytochrome c oxidase assembly protein COX15 homolog	92.68%	3	1	35	96	-0.076	-0.226	-0.168	0.006	-0.041	4																									396	44.0	9.70
IPI00406493.3	Isoform 2 of DAZ-associated protein 1	49.63%	4	8	23	150	0.219	-0.044	0.203	0.084	0.218	12	-0.050	-0.065	0.149	-0.045	-0.013	12	0.348	0.171	0.309	0.246	0.083	10	-0.342	-0.279	-0.019	-0.309	0.294	3							405	43.1	8.56
IPI00339428.9	Isoform 2 of Dedicator of cytokinesis protein 7	76.97%	6	11	190	571	0.001	0.256	0.119	0.213	-0.130	3							-0.016	-0.034	0.070	-0.168	-0.133	13													2128	241.0	6.73
IPI00845581.1	Isoform 2 of Dihydropyrimidinase-related protein 4	79.93%	4	12	52	301																									0.158	0.219	0.107	0.217	0.106	35	593	64.3	7.09
IPI00752220.1	Isoform 2 of Disks large-associated protein 4	73.21%	6	5	67	165																									-0.286	-0.204	-0.095	0.064	-0.023	6	989	107.5	7.30
IPI00274739.1	Isoform 2 of DNA-binding protein A	85.27%	10	2	35	325	-0.105	-0.109	-0.095	-0.117	-0.167	23	0.156	0.061	0.019	0.061	0.001	28	-0.057	0.045	0.080	-0.074	0.016	28	0.283	0.241	0.240	0.016	0.197	22							292	30.7	9.63
IPI00279858.1	Isoform 2 of DnaJ homolog subfamily A member 3, mitochondrial	80.35%	1	1	40	146							-0.134	-0.333	0.122	-0.123	-0.331	8							-0.146	-0.141	-0.193	-0.057	-0.082	12							453	49.5	9.31
IPI00308222.3	Isoform 2 of Drebrin-like protein	72.98%	3	15	54	381	0.072	-0.068	-0.041	-0.088	0.033	18	-0.009	0.085	0.047	-0.100	0.080	20	0.665	0.190	0.420	0.463	0.397	7	-0.016	0.004	0.120	0.088	0.112	16	0.126	0.043	-0.088	0.085	0.037	17	433	48.4	4.92
IPI00114245.2	Isoform 2 of Dual specificity mitogen-activated protein kinase kinase 3	86.74%	3	5	30	143	-0.116	-0.057	-0.103	-0.108	-0.240	14	0.209	-0.119	-0.034	-0.398	0.282	6	-0.515	-0.150	-0.186	0.198	-0.080	5													347	39.3	7.43
IPI00314883.4	Isoform 2 of Dynamin-2	79.33%	8	12	93	731	-0.060	0.013	-0.105	0.061	-0.065	25	0.317	0.040	0.324	-0.016	0.382	30	-0.106	-0.160	0.044	0.207	0.024	4							0.061	0.023	-0.053	-0.085	-0.106	50	866	97.7	7.62
IPI00403336.4	Isoform 2 of Dynamin-like 120 kDa protein, mitochondrial	82.45%	6	36	124	617	-0.056	0.120	-0.039	0.036	0.036	7	0.354	0.261	-0.006	-0.071	0.188	5	0.239	0.105	0.070	-0.006	-0.079	8	-0.004	-0.144	-0.054	0.029	-0.117	9	-0.065	0.011	-0.047	-0.082	-0.089	78	997	115.5	7.55
IPI00113271.2	Isoform 2 of Dysferlin	50.81%	5	1	115	402	0.116	0.023	0.132	0.128	0.336	5	0.401	0.210	0.069	0.045	-0.198	3	0.288	0.038	0.236	0.034	0.195	3													2100	239.1	5.82
IPI00648087.1	Isoform 2 of E3 ubiquitin-protein ligase UBR2	54.19%	3	8	103	319							0.004	-0.054	0.068	-0.017	0.099	12																			1755	199.1	6.28
IPI00224682.2	Isoform 2 of Elongator complex protein 3	68.20%	2	5	43	82																			-0.178	-0.056	-0.136	0.120	-0.219	5							566	64.2	9.01
IPI00109256.4	Isoform 2 of Ena/VASP-like protein	59.66%	4	2	24	88																									-0.394	-0.300	-0.531	0.375	-0.878	3	414	44.3	8.85
IPI00416751.4	Isoform 2 of Equilibrative nucleoside transporter 1	62.45%	11	5	23	73	-0.014	0.126	-0.025	0.013	-0.045	8																									458	50.0	8.32
IPI00409918.1	Isoform 2 of Eukaryotic initiation factor 4A-II	91.67%	6	8	48	563	-0.116	-0.155	-0.032	0.030	-0.032	27	-0.047	0.011	-0.034	-0.019	0.069	24	-0.209	-0.021	-0.054	0.130	-0.017	31	0.158	-0.025	0.107	-0.092	0.240	24	-0.001	-0.016	-0.026	-0.057	-0.062	47	408	46.5	5.48
IPI00269481.7	Isoform 2 of F-actin-capping protein subunit beta	81.99%	8	20	44	447	0.114	-0.068	0.179	0.010	0.160	28	0.020	0.065	0.059	0.112	-0.122	33	-0.102	-0.095	-0.042	0.119	-0.006	25	0.103	0.047	0.240	0.024	0.247	16	-0.068	-0.057	-0.109	-0.032	-0.099	36	272	30.6	6.00
IPI00222107.6	Isoform 2 of FERM domain-containing protein 4A	72.94%	3	4	102	302																			-0.082	-0.010	-0.122	0.036	-0.037	6							1042	116.4	9.03
IPI00387559.2	Isoform 2 of Gamma-adducin	80.31%	2	16	62	326							0.364	0.420	0.862	0.428	0.352	18													-0.076	-0.085	0.051	0.087	-0.028	29	706	78.7	5.95
IPI00323554.3	Isoform 2 of Gamma-aminobutyric acid receptor subunit beta-2	82.07%	3	4	39	113																									-0.086	0.197	0.258	0.200	0.134	13	474	54.6	9.32
IPI00759948.1	Isoform 2 of Gelsolin	77.84%	2	31	69	867	-0.076	-0.120	-0.048	-0.059	0.034	68	-0.586	-0.050	-0.617	-0.053	-0.599	50	0.009	0.009	-0.026	0.016	-0.100	61							-0.162	0.134	0.050	0.141	0.066	16	731	80.7	5.76
IPI00122353.1	Isoform 2 of Glucosidase 2 subunit beta	70.45%	2	19	37	318	0.111	0.098	0.027	0.135	0.024	24	0.154	0.106	0.172	0.043	0.258	47	-0.059	0.030	-0.072	-0.025	-0.065	17	-0.279	-0.022	-0.288	0.043	-0.317	12	-0.742	-0.333	-0.233	-0.111	0.021	4	528	59.5	4.48
IPI00845769.1	Isoform 2 of Glycerol-3-phosphate dehydrogenase 1-like protein	97.81%	3	12	39	150	0.419	-0.160	0.457	-0.112	0.664	8																			0.017	0.057	-0.012	0.003	0.014	23	320	34.6	6.87
IPI00406596.4	Isoform 2 of GTPase-activating protein and VPS9 domain-containing protein	62.07%	10	8	112	301	-0.141	-0.226	-0.130	-0.068	-0.116	4													0.073	-0.246	-0.102	-0.112	-0.482	4							1437	160.0	5.20
IPI00856505.1	Isoform 2 of HAUS augmin-like complex subunit 3	81.61%	2	1	55	159							0.149	0.242	0.262	0.410	-0.299	3																			533	61.8	5.10
IPI00876146.2	Isoform 2 of HAUS augmin-like complex subunit 8	80.91%	2	2	42	139							-0.639	0.003	-0.624	-0.260	0.100	3																			372	41.5	6.47
IPI00608040.1	Isoform 2 of Hepatoma-derived growth factor-related protein 3	76.59%	3	5	19	91	-0.108	-0.114	-0.079	0.001	-0.017	3	-0.336	-0.116	-0.295	0.031	-0.322	10																			205	22.8	8.54
IPI00896020.1	Isoform 2 of Heterochromatin protein 1-binding protein 3	84.10%	8	15	79	357	0.132	0.066	0.112	0.011	0.059	17	0.048	0.092	0.068	0.172	-0.141	12	0.044	0.087	0.156	-0.083	0.017	5	0.219	0.183	0.259	0.043	0.219	3	-0.037	0.062	0.239	0.114	0.062	19	541	59.5	9.79
IPI00224575.1	Isoform 2 of Heterogeneous nuclear ribonucleoprotein K	84.05%	4	10	55	1512	0.132	0.020	0.081	0.001	0.073	96	0.003	-0.016	-0.017	0.040	-0.119	82	0.192	0.029	0.072	-0.026	0.120	83	0.131	0.030	0.204	0.004	0.188	110	-0.001	0.038	0.111	0.076	0.107	59	464	51.0	5.33
IPI00754296.1	Isoform 2 of High mobility group nucleosome-binding domain-containing pr	62.34%	1	1	8	27																			-0.204	0.021	-0.102	-0.022	-0.131	3							77	8.4	10.10
IPI00761713.2	Isoform 2 of Histone H2B type 1-P	89.86%	10	1	26	1014	0.178	0.147	0.162	0.048	0.114	50	0.273	0.156	0.193	0.240	-0.056	54	0.598	0.165	0.534	0.041	0.430	89	0.494	0.019	0.582	-0.050	0.449	82	-0.086	0.054	0.318	0.073	0.089	69	138	15.6	10.24
IPI00395047.2	Isoform 2 of Homer protein homolog 3	89.14%	6	8	45	208							-0.226	-0.028	-0.262	-0.309	-0.109	7													-0.076	-0.066	0.212	0.252	0.051	10	359	40.0	5.29
IPI00355368.4	Isoform 2 of Importin-11	58.03%	3	5	54	177	0.227	-0.035	-0.123	-0.035	0.003	4																									984	113.3	5.24
IPI00798463.1	Isoform 2 of Integrator complex subunit 6	65.90%	2	4	74	229	-0.088	-0.146	-0.224	-0.094	0.091	4																									874	98.6	8.66
IPI00468674.5	Isoform 2 of Integrin alpha-3	58.61%	3	10	67	253	0.120	0.073	0.102	0.047	0.083	23	0.283	0.017	0.127	-0.116	0.279	13																			1068	118.8	6.81
IPI00867847.1	Isoform 2 of Kinesin-like protein KIF2A	76.02%	7	15	76	397	0.043	0.115	0.024	-0.019	0.112	8	-0.125	-0.054	-0.004	-0.001	-0.170	9	0.041	0.009	0.062	-0.013	0.225	11	-0.308	0.095	-0.208	0.006	-0.175	19	0.010	0.069	0.062	-0.042	0.043	25	659	74.8	6.46
IPI00322712.2	Isoform 2 of Lactadherin	75.59%	2	9	39	174	-0.158	-0.069	0.017	-0.026	0.052	9							-0.037	0.051	0.114	-0.051	-0.123	11													426	47.1	7.05
IPI00124830.3	Isoform 2 of Leukocyte surface antigen CD47	54.63%	1	1	22	207	-0.031	0.157	-0.114	0.001	-0.178	8	0.300	0.160	0.001	0.322	0.075	4	0.452	0.179	0.122	0.068	-0.250	7	-0.862	-0.029	-0.994	-0.077	-0.852	8	0.011	-0.029	-0.014	0.037	-0.076	31	324	35.3	8.25
IPI00223459.1	Isoform 2 of Lipid phosphate phosphohydrolase 1	70.77%	2	2	15	43																			0.200	0.296	0.603	0.094	0.330	5							284	32.0	7.78
IPI00136806.4	Isoform 2 of LMBR1 domain-containing protein 2	60.09%	3	4	37	86																			-0.383	-0.131	-0.515	-0.071	-0.234	6							649	75.7	7.50
IPI00808497.1	Isoform 2 of Lymphocyte-specific helicase	77.85%	3	10	87	366	-0.017	-0.029	-0.204	-0.045	-0.100	13	-0.068	-0.031	-0.116	-0.083	-0.290	9							0.204	-0.097	0.180	-0.060	0.085	6							808	93.7	8.19
IPI00756238.2	Isoform 2 of MAGUK p55 subfamily member 2	70.83%	2	9	47	146																									0.087	0.149	0.366	0.160	0.123	12	569	63.3	6.11
IPI00515213.2	Isoform 2 of Mannosyl-oligosaccharide 1,2-alpha-mannosidase IB	64.50%	2	3	41	98							0.091	0.167	0.145	0.120	0.081	3																			614	69.9	8.68
IPI00625061.2	Isoform 2 of MAP/microtubule affinity-regulating kinase 3	81.21%	4	3	94	228	-0.163	0.069	-0.209	0.116	-0.460	3																			0.165	-0.123	-0.077	-0.057	0.037	10	729	81.3	9.44
IPI00754963.2	Isoform 2 of Mesoderm-specific transcript protein	49.69%	3	3	13	51																			0.644	0.341	0.710	0.152	0.900	10							326	37.6	9.44
IPI00649402.2	Isoform 2 of Methionine adenosyltransferase 2 subunit beta	72.14%	3	5	32	173	-0.189	-0.032	0.003	-0.056	0.069	10	-0.108	-0.133	0.081	-0.120	-0.076	4							0.189	-0.041	0.100	-0.003	0.065	9							323	36.2	6.60
IPI00381412.1	Isoform 2 of Mitochondrial inner membrane protein	94.37%	7	3	111	1348	-0.053	0.057	-0.001	0.017	-0.094	69	0.043	0.019	-0.017	0.006	-0.045	46	0.418	0.099	0.363	-0.035	0.253	64	-0.171	-0.068	-0.045	-0.022	-0.139	67	0.017	-0.004	0.001	-0.056	-0.020	143	746	82.9	6.61
IPI00265705.2	Isoform 2 of Mitogen-activated protein kinase kinase kinase MLT	59.69%	2	2	25	107	0.020	-0.127	-0.206	-0.157	-0.221	4	-0.150	0.059	0.138	-0.009	0.292	7																			454	51.3	5.35
IPI00227374.1	Isoform 2 of N-acetylgalactosaminyltransferase 7	49.62%	3	2	30	62	-0.099	-0.083	-0.051	-0.114	-0.034	4																									657	75.5	7.68
IPI00753284.3	Isoform 2 of N-alpha-acetyltransferase 50, NatE catalytic subunit	98.21%	5	10	28	185	-0.152	0.006	-0.240	-0.144	-0.226	18	-0.311	-0.088	-0.088	-0.091	-0.346	5	-0.417	-0.032	-0.297	-0.010	-0.199	14	0.156	0.169	-0.009	0.347	0.204	4							168	19.3	8.81
IPI00108783.1	Isoform 2 of Neurofibromin	79.27%	5	6	247	767																									-0.236	-0.194	-0.364	-0.178	-0.295	5	2841	319.4	7.39
IPI00338880.3	Isoform 2 of Neuronal cell adhesion molecule	49.58%	6	15	52	208																									-0.079	-0.009	-0.109	-0.234	-0.007	40	1186	131.2	6.06
IPI00757771.1	Isoform 2 of Neuroplastin	78.59%	3	6	46	285	0.020	0.128	-0.080	-0.009	0.004	3	0.547	0.185	0.483	0.214	0.218	7	0.081	0.106	0.119	0.153	0.013	13							-0.020	0.000	0.091	0.031	-0.039	55	397	44.3	7.74
IPI00115679.1	Isoform 2 of Neutral alpha-glucosidase AB	84.37%	2	1	78	577	0.234	0.057	0.192	0.029	0.162	39	0.110	0.055	0.162	0.073	0.055	53	-0.037	0.034	0.128	0.027	0.059	15	-0.290	-0.032	-0.349	-0.041	-0.329	42	0.010	-0.130	-0.226	-0.295	-0.216	17	966	109.3	6.15
IPI00828368.2	Isoform 2 of NTF2-related export protein 2	52.02%	2	2	9	44							-0.565	-0.231	-0.567	-0.398	-0.597	3																			198	22.6	6.90
IPI00230656.1	Isoform 2 of Nuclear factor 1 B-type	62.83%	20	1	31	256	0.104	0.004	0.352	0.197	0.533	5													0.386	-0.026	0.204	-0.077	0.127	8							487	54.9	8.57
IPI00857897.1	Isoform 2 of Paraspeckle component 1	71.67%	2	5	49	265	-0.116	-0.163	-0.171	-0.265	-0.221	8	0.132	0.123	0.132	0.161	-0.079	6	0.011	-0.069	0.394	-0.133	0.224	5	-0.045	-0.068	0.037	-0.133	0.000	13	0.024	0.148	0.279	0.069	-0.037	10	466	53.0	6.09
IPI00881287.1	Isoform 2 of Peptidyl-prolyl cis-trans isomerase FKBP8	88.83%	2	10	36	188							-0.080	0.087	0.027	0.059	0.000	15	-0.236	-0.044	-0.112	-0.117	-0.133	9	-0.419	0.021	-0.385	0.031	-0.127	13	-0.595	-0.366	-0.260	-0.299	-0.274	3	403	43.6	5.16
IPI00130081.2	Isoform 2 of Peroxisomal targeting signal 1 receptor	78.07%	3	12	34	137							-0.402	-0.183	-0.340	-0.082	-0.272	20																			602	66.8	4.55
IPI00625118.3	Isoform 2 of Phosphatase and actin regulator 4	70.61%	4	6	55	138	0.374	-0.056	0.373	-0.246	0.292	4	0.036	-0.088	-0.147	-0.066	0.041	5																			667	73.4	6.38
IPI00336929.3	Isoform 2 of Phostensin	58.78%	2	6	52	234	-0.039	0.134	0.004	0.154	0.059	12							0.104	0.009	-0.089	-0.001	0.013	12													609	67.1	5.68
IPI00471476.1	Isoform 2 of Plasminogen activator inhibitor 1 RNA-binding protein	80.87%	3	3	52	826	0.127	0.068	0.134	0.098	0.062	64	-0.001	-0.045	-0.072	-0.034	-0.100	79	0.059	0.068	0.076	-0.037	0.123	88	0.251	0.110	0.212	0.104	0.167	52	0.044	-0.076	-0.045	0.006	0.006	13	392	42.9	8.44
IPI00403827.2	Isoform 2 of Pleckstrin homology-like domain family B member 2	80.41%	14	23	147	599	0.122	-0.026	0.130	0.050	0.088	13	0.110	-0.103	0.107	0.017	0.044	27																			1302	147.3	7.80
IPI00127174.4	Isoform 2 of Poly(U)-binding-splicing factor PUF60	81.72%	2	1	57	567	0.054	0.041	-0.029	0.030	-0.085	39	-0.139	-0.196	-0.386	-0.062	-0.201	26	0.014	-0.013	-0.059	-0.020	-0.034	35	0.104	-0.095	0.140	-0.026	0.185	34							547	58.5	5.35
IPI00136002.1	Isoform 2 of Polypyrimidine tract-binding protein 2	88.16%	3	13	49	354	0.062	0.120	0.062	0.106	0.058	10	-0.783	-0.074	-0.580	-0.007	-0.599	13	0.386	0.051	0.114	0.038	0.088	5	0.448	0.036	0.401	0.013	0.239	14	0.138	0.160	0.072	0.044	0.140	12	532	57.6	8.81
**IPI00757147.1**	**Isoform 2 of Pre-mRNA-splicing factor CWC22 homolog**	**59.87%**	**8**	**5**	**77**	**170**																															**902**	**104.1**	**8.09**
IPI00788337.1	Isoform 2 of Presequence protease, mitochondrial	85.02%	3	29	97	590	-0.004	0.062	0.065	-0.022	0.045	14	0.420	-0.059	0.465	0.123	0.425	37	0.135	-0.004	0.037	-0.131	0.051	27	-0.209	-0.039	-0.357	-0.109	-0.218	24							1035	117.1	7.12
IPI00267492.4	Isoform 2 of Protein bicaudal D homolog 2	79.55%	4	7	109	421	0.088	-0.022	0.003	-0.003	-0.255	3							-0.072	-0.085	-0.194	-0.421	-0.100	9													851	96.6	5.44
IPI00876418.2	Isoform 2 of Protein EFR3 homolog B	84.86%	2	3	78	232																									-0.260	-0.136	-0.047	-0.243	-0.206	3	773	87.5	6.68
IPI00648806.2	Isoform 2 of Protein FAM114A2	76.53%	2	9	44	225	-0.082	-0.071	0.134	0.054	0.014	9	-0.060	-0.031	0.128	0.144	0.124	7							-0.057	0.236	0.010	0.119	-0.047	8							490	53.3	4.89
IPI00653706.1	Isoform 2 of Protein FAM126B	57.85%	2	6	42	153																									-0.019	-0.144	-0.340	-0.214	-0.248	12	586	64.4	6.67
IPI00830404.1	Isoform 2 of Protein FAM63A	40.09%	2	2	20	78	1.202	-0.022	1.242	-0.031	0.930	5	1.136	0.299	1.193	0.293	1.215	8																			459	50.2	4.74
IPI00457485.3	Isoform 2 of Protein LAP2	80.90%	5	8	121	380	0.088	0.126	-0.102	-0.184	-0.196	5	-0.260	-0.241	-0.009	0.189	-0.085	3																			1450	162.1	5.86
IPI00124154.2	Isoform 2 of Protein NDRG4	61.06%	1	1	18	62																									-0.035	-0.050	-0.189	0.011	-0.080	16	339	37.1	5.90
IPI00410883.1	Isoform 2 of Protein SET	61.73%	1	4	28	612	-0.271	-0.057	-0.023	0.048	-0.201	36	-0.243	-0.023	-0.272	-0.025	-0.189	25	-0.231	-0.166	-0.368	0.077	-0.089	32	0.285	0.111	0.301	0.033	0.348	78	-0.010	0.107	0.249	0.083	0.123	11	277	32.1	4.22
IPI00828226.1	Isoform 2 of Protein TBRG4	64.50%	6	4	50	130	-0.404	0.073	-0.468	-0.146	-0.308	3													0.198	0.030	0.034	-0.122	0.003	4							600	68.1	8.32
IPI00830899.2	Isoform 2 of RAF proto-oncogene serine/threonine-protein kinase	75.30%	4	5	71	205	0.030	0.017	-0.226	-0.083	-0.250	5	0.066	0.295	-0.057	-0.243	0.013	3																			668	75.1	9.32
IPI00338228.2	Isoform 2 of RalBP1-associated Eps domain-containing protein 1	59.05%	2	1	16	39																			-0.897	-0.158	-0.578	-0.127	-0.542	3							210	23.4	4.98
IPI00230011.3	Isoform 2 of Ras-related protein Rab-6A	89.90%	9	1	35	276	0.172	0.036	0.007	0.064	0.085	13	0.115	-0.094	0.014	-0.017	0.076	12	0.124	0.078	0.092	-0.086	0.077	19	-0.260	-0.038	-0.243	-0.038	-0.146	12	-0.109	-0.149	-0.032	-0.127	-0.094	20	208	23.5	5.54
IPI00915505.1	Isoform 2 of Receptor-type tyrosine-protein phosphatase S	61.61%	8	5	116	263																									0.158	0.135	-0.066	0.031	0.157	10	1904	211.4	7.30
IPI00889254.1	Isoform 2 of RELT-like protein 1	72.28%	2	2	14	35							0.457	0.424	0.548	0.070	0.433	5																			202	22.2	8.34
IPI00480414.2	Isoform 2 of Reticulon-3	81.48%	5	20	89	479							0.149	0.102	0.203	0.148	0.115	3	0.038	0.064	0.076	-0.128	0.068	6	-0.149	0.122	-0.180	0.047	-0.320	5	0.036	0.054	0.016	0.079	0.066	41	945	101.9	4.93
IPI00468834.4	Isoform 2 of RNA-binding protein 10	73.86%	3	10	79	445	0.188	-0.108	-0.168	0.169	0.045	3	0.187	0.064	0.153	0.176	-0.676	8	0.162	0.081	0.258	0.010	0.215	10													853	94.4	6.70
IPI00928541.1	Isoform 2 of RNA-binding protein 25	59.79%	3	17	75	346	0.050	0.016	-0.010	0.021	0.020	23	0.043	-0.076	-0.241	-0.112	-0.127	21	0.255	0.074	0.430	0.070	0.282	13	0.029	0.006	0.147	-0.003	0.050	16							838	99.5	6.32
IPI00223371.3	Isoform 2 of RNA-binding protein 39	84.54%	7	14	54	499	-0.092	0.064	-0.076	-0.020	-0.019	25	-0.253	0.051	-0.100	0.048	-0.302	19	0.034	0.024	0.040	0.010	-0.009	20	-0.004	-0.034	0.023	-0.071	-0.095	28	0.372	-0.315	0.088	-0.026	0.030	3	524	58.6	10.13
IPI00798508.1	Isoform 2 of RNA-binding protein 5	61.18%	3	4	63	206	-0.203	0.062	-0.150	0.064	-0.141	6							0.134	0.073	0.371	0.095	0.241	4													814	92.2	6.21
IPI00109860.3	Isoform 2 of RNA-binding protein 8A	61.85%	3	7	9	131	0.085	0.104	0.106	0.100	0.184	8	-0.117	-0.141	-0.105	-0.034	-0.152	17	-0.026	0.011	-0.060	-0.037	0.044	11	-0.146	-0.025	0.043	-0.016	0.075	13							173	19.7	5.92
IPI00462260.1	Isoform 2 of RNA-binding protein Musashi homolog 1	79.73%	3	2	25	63																			0.068	-0.338	0.194	-0.099	0.163	9							301	34.0	9.66
IPI00556695.1	Isoform 2 of SAFB-like transcription modulator	69.69%	3	14	112	425	-0.100	0.080	-0.051	0.073	-0.094	19	0.257	-0.368	-0.127	-0.349	-0.344	3							-0.246	-0.083	-0.214	-0.059	-0.223	11							1013	115.1	8.05
IPI00474373.1	Isoform 2 of Sequestosome-1	69.80%	3	1	20	189							0.408	0.180	0.783	-0.462	0.302	8	-0.371	-0.018	-0.462	0.176	-0.392	25	0.100	0.219	-0.304	0.203	0.308	11							404	44.2	5.44
IPI00407158.2	Isoform 2 of Serine racemase	55.10%	5	5	15	45																									0.167	0.122	-0.039	0.220	0.127	10	314	34.0	6.01
IPI00972877.1	Isoform 2 of Serine/threonine-protein kinase WNK1	48.74%	6	9	140	488	-0.001	0.091	-0.293	0.079	0.062	8	-0.092	-0.589	-0.329	0.068	-0.056	5	0.376	-0.031	0.092	0.263	0.044	3													2626	279.0	6.61
IPI00756703.1	Isoform 2 of Serine/threonine-protein phosphatase 2B catalytic subunit alpl	89.43%	6	1	51	441																			-0.173	-0.191	-0.120	-0.154	0.104	3	0.007	-0.105	-0.057	-0.042	-0.072	89	511	57.6	6.30
IPI00720007.1	Isoform 2 of Serine/threonine-protein phosphatase 6 regulatory subunit 3	88.63%	5	12	62	304	-0.004	-0.045	0.134	0.092	0.003	9	0.066	0.128	0.010	0.126	0.061	20																			827	92.6	4.59
IPI00757214.2	Isoform 2 of SLAIN motif-containing protein 2	85.50%	3	4	74	281							-0.991	-0.338	-0.554	-0.569	-0.204	3	0.464	0.001	-0.012	0.198	0.257	4													607	65.2	9.76
IPI00399631.1	Isoform 2 of Small glutamine-rich tetratricopeptide repeat-containing prote	68.15%	2	14	24	226	-0.014	-0.050	0.054	-0.006	0.027	16	-0.231	-0.120	-0.368	-0.327	-0.281	9	-0.057	-0.153	-0.099	0.019	0.075	17	-0.054	0.055	0.034	0.079	-0.086	21	-0.013	-0.072	-0.016	0.034	-0.009	10	314	34.2	5.06
IPI00652964.2	Isoform 2 of Small ubiquitin-related modifier 3	91.82%	7	1	15	136	-0.102	-0.144	-0.007	-0.045	-0.054	9	-0.139	-0.006	0.001	-0.001	0.006	10							0.379	0.057	0.293	-0.029	0.374	4	0.108	0.202	0.416	0.245	0.270	7	110	12.3	5.92
IPI00555118.4	Isoform 2 of Sodium-driven chloride bicarbonate exchanger	64.43%	3	10	63	219																									0.021	-0.201	-0.163	-0.007	-0.136	35	1088	122.5	6.38
IPI00673886.5	Isoform 2 of Sorbin and SH3 domain-containing protein 2	74.52%	9	3	124	506	-0.086	0.030	-0.077	0.048	-0.056	53							0.037	0.044	-0.136	0.007	-0.178	8													1295	144.9	8.27
IPI00556765.1	Isoform 2 of Sorcin	76.50%	2	8	22	149	-0.072	-0.020	-0.051	0.016	-0.037	14	-0.206	-0.112	-0.109	-0.120	-0.014	8	0.192	-0.091	0.167	0.235	0.192	10													183	20.3	5.34
IPI00650038.2	Isoform 2 of Sortilin	44.76%	2	5	44	110																									0.023	0.001	-0.053	0.166	0.059	7	858	94.8	5.99
IPI00121892.9	Isoform 2 of Spectrin beta chain, brain 1	93.41%	4	2	288	3727	0.006	0.021	0.009	-0.016	0.006	174	0.411	0.023	0.402	0.072	0.420	182	-0.045	0.023	-0.079	-0.034	-0.138	159	-0.297	0.027	-0.257	0.027	-0.279	167	0.037	0.116	0.077	0.091	0.084	453	2154	251.0	5.54
IPI00379480.1	Isoform 2 of Splicing regulatory glutamine/lysine-rich protein 1	55.90%	2	4	48	144							0.006	-0.258	0.455	0.017	-0.117	3							-0.309	-0.050	-0.082	-0.106	-0.292	5							610	69.1	10.27
IPI01008021.2	Isoform 2 of Striatin-4	55.91%	2	2	44	135							-0.076	0.122	0.098	0.126	0.136	4													-0.142	-0.236	0.064	-0.089	-0.025	5	753	80.9	5.38
IPI00121270.5	Isoform 2 of SWI/SNF-related matrix-associated actin-dependent regulator (	77.86%	2	2	41	167																			-0.138	-0.327	-0.094	-0.175	-0.281	10							515	58.2	9.25
IPI00227035.2	Isoform 2 of Syntaphilin	64.20%	10	7	45	160																									0.135	0.072	-0.004	-0.042	0.092	8	528	57.0	6.02
IPI00415403.1	Isoform 2 of Syntaxin-binding protein 1	97.01%	1	2	84	616																									0.054	0.030	0.055	-0.029	-0.007	219	603	68.7	6.77
IPI00222197.1	Isoform 2 of Target of Myb protein 1	57.14%	1	1	11	44							0.375	-0.508	0.165	-0.133	0.224	3													0.646	0.202	0.441	0.064	-0.194	4	168	17.8	4.78
IPI00854004.1	Isoform 2 of Tetratricopeptide repeat protein 27	65.01%	2	3	71	219																			0.163	0.118	0.026	0.106	0.011	5							846	96.2	5.77
IPI00121887.5	Isoform 2 of Transcription elongation factor A protein 1	91.03%	7	16	46	417	-0.091	-0.042	-0.047	-0.079	-0.157	10	-0.283	-0.082	-0.228	0.030	-0.233	27	-0.344	-0.038	-0.274	0.104	-0.162	10	0.171	0.037	0.214	-0.044	0.217	32							301	33.9	8.38
IPI00229485.1	Isoform 2 of Transcription elongation regulator 1	42.72%	4	13	68	202	-0.095	0.100	-0.085	-0.047	0.004	4	0.016	-0.117	-0.111	0.045	-0.039	17							-0.032	0.016	0.055	0.021	0.085	3							1079	121.5	8.54
IPI00131186.2	Isoform 2 of Transcription factor BTF3	97.53%	3	13	24	908	-0.004	-0.048	0.103	0.034	0.031	61	0.111	0.020	0.058	0.029	0.059	35	-0.099	-0.001	-0.114	0.081	0.023	41	0.104	-0.122	0.050	-0.122	0.145	88	-0.181	0.038	0.180	0.058	0.335	3	162	17.7	7.50
IPI00221819.2	Isoform 2 of Translation initiation factor eIF-2B subunit delta	83.64%	4	3	55	209	0.245	-0.097	0.209	-0.127	0.081	3							0.180	0.051	0.119	-0.127	-0.048	3	0.088	0.219	-0.117	0.084	-0.119	5							544	59.6	9.25

IPI00464115.1	Isoform 2 of Transportin-3	73.30%	2	7	68	194	0.019	-0.023	-0.498	0.024	0.069	3	-0.141	0.189	0.068	-0.264	0.088	6							0.080	0.013	-0.072	0.238	0.023	4							929	104.8	5.57
IPI00227835.1	Isoform 2 of Tropomyosin alpha-1 chain	95.07%	6	0	62	1062	-0.176	0.057	-0.057	0.036	-0.178	103	0.533	-0.007	0.377	0.000	0.194	89	-0.201	-0.090	-0.119	-0.007	-0.053	91	0.176	0.026	0.290	0.004	0.153	19	0.023	-0.041	0.017	0.004	0.058	33	284	32.7	4.75
IPI00230044.5	Isoform 2 of Tropomyosin alpha-3 chain	95.97%	3	3	62	918	0.144	0.027	0.153	0.047	0.172	76	0.084	0.003	-0.007	0.021	0.016	63	-0.051	-0.065	0.024	0.076	0.148	78	0.141	0.023	0.202	0.003	0.107	52	0.023	0.114	0.114	0.045	0.144	36	248	29.0	4.78
IPI00874728.1	Isoform 2 of Tropomyosin beta chain	92.25%	5	4	54	641	-0.007	0.088	0.115	0.059	0.026	45	0.052	-0.065	-0.056	-0.016	0.000	54	-0.175	-0.119	-0.076	-0.031	-0.035	76	0.162	0.014	0.210	0.009	0.112	14	0.023	-0.086	0.017	0.003	0.045	23	284	32.9	4.67
IPI00355382.2	Isoform 2 of Tryptophanyl-tRNA synthetase, cytoplasmic	95.58%	3	19	62	415	-0.088	-0.173	0.014	-0.088	0.136	22	-0.032	-0.039	-0.045	0.044	-0.062	19	-0.158	-0.220	-0.274	0.110	-0.094	8	-0.304	0.210	-0.413	0.072	-0.364	51	-0.083	0.048	0.026	-0.004	-0.035	29	475	53.6	7.08
IPI00125270.1	Isoform 2 of TSC22 domain family protein 1	67.13%	4	1	14	134	-0.023	-0.001	-0.059	-0.165	0.020	6	-0.523	-0.105	-0.423	-0.056	-0.517	4													0.384	0.169	0.167	0.096	0.080	7	143	15.6	5.20
IPI00751146.1	Isoform 2 of Twinfilin-2	74.64%	2	8	31	106	-0.276	-0.281	-0.039	0.059	-0.183	7	0.059	-0.141	-0.007	-0.085	0.099	9													-0.060	0.027	-0.170	-0.127	0.134	6	347	39.3	6.70
IPI00322097.6	Isoform 2 of Tyrosine-protein kinase Fyn	58.61%	46	2	34	153							-0.130	0.089	0.130	0.095	-0.085	8	0.183	0.061	-0.037	0.059	-0.034	6							0.118	0.104	-0.022	-0.019	-0.163	6	534	60.0	6.11
IPI00316479.7	Isoform 2 of Tyrosine-protein phosphatase non-receptor type 11	81.96%	2	15	63	385	0.036	-0.238	0.215	0.021	0.156	16	-0.290	0.003	-0.265	-0.057	-0.158	12							-0.041	-0.079	-0.012	-0.003	0.140	11	0.070	0.019	-0.111	0.001	0.106	9	593	68.0	7.30
IPI00221791.5	Isoform 2 of Tyrosine-protein phosphatase non-receptor type substrate 1	72.69%	8	14	45	178																									0.087	-0.147	-0.142	-0.051	-0.095	36	509	56.0	8.15
IPI00467507.4	Isoform 2 of U2 snRNP-associated SURP motif-containing protein	72.76%	1	1	105	323	-0.127	-0.016	0.036	-0.209	-0.136	9	-0.127	-0.042	-0.288	-0.134	-0.336	5																			1028	118.1	8.47
IPI00280207.1	Isoform 2 of Ubiquilin-1	54.51%	1	1	30	958	0.240	0.106	0.348	0.102	0.148	28	-0.038	0.021	-0.184	-0.066	-0.157	32	-0.079	-0.131	0.157	-0.062	0.140	10	-0.238	0.051	-0.257	-0.003	-0.178	64	0.031	0.052	-0.053	-0.066	-0.134	13	554	58.6	4.93
IPI00420601.6	Isoform 2 of Ubiquitin carboxyl-terminal hydrolase 10	68.85%	3	15	56	201	-0.006	-0.028	-0.017	0.043	-0.034	14	0.024	-0.086	-0.094	0.081	0.143	12	0.170	0.155	0.020	0.062	0.031	8	0.052	-0.029	0.122	-0.060	0.127	18							793	87.0	5.17
IPI00230763.1	Isoform 2 of Ubiquitin carboxyl-terminal hydrolase isozyme L5	98.17%	1	1	37	248	-0.069	0.003	-0.077	-0.010	-0.136	26	0.068	-0.051	0.088	-0.050	0.055	12	-0.292	-0.088	-0.120	-0.056	-0.009	12	0.080	0.076	-0.038	0.084	0.017	14							328	37.5	5.40
IPI00761172.1	Isoform 2 of Ubiquitin-associated protein 2-like	67.36%	3	1	65	1306	0.023	-0.026	-0.026	-0.023	0.024	62	-0.010	-0.062	-0.053	-0.029	-0.170	77	0.072	0.031	0.087	0.013	0.151	46	0.023	-0.010	0.040	0.004	0.065	33	-0.188	-0.152	-0.216	-0.203	-0.116	8	1014	107.2	7.11
IPI00720171.2	Isoform 2 of Uncharacterized protein KIAA0090	79.28%	2	1	79	437	-0.053	0.127	0.148	0.068	-0.060	25	-0.390	-0.029	-0.186	0.079	-0.007	11	0.040	0.072	0.081	0.031	0.024	14	-0.496	-0.106	-0.338	-0.044	-0.288	11							994	111.2	7.43
IPI00420527.2	Isoform 2 of Uncharacterized protein KIAA1522	50.37%	5	5	60	197	-0.004	-0.167	0.026	-0.068	0.033	11																									1070	110.6	10.07
IPI00652728.1	Isoform 2 of Urea transporter 1	17.27%	1	2	10	22																									-0.421	-0.320	-0.089	-0.131	-0.079	3	440	48.3	7.06
IPI00329942.4	Isoform 2 of Vacuolar protein sorting-associated protein 26A	94.43%	3	10	40	174							0.064	-0.019	-0.386	-0.236	0.064	5	-0.300	-0.054	-0.114	-0.123	-0.017	13	-0.023	0.190	-0.004	-0.032	-0.265	7							359	41.5	6.96
IPI00264750.4	Isoform 2 of Vascular cell adhesion protein 1	78.26%	1	1	29	109							-0.170	0.001	0.010	0.107	-0.152	11																			345	38.3	5.44
IPI00221831.1	Isoform 2 of Vesicular inhibitory amino acid transporter	54.32%	3	10	31	142																									0.096	0.024	-0.095	-0.032	-0.133	28	521	56.8	6.90
IPI00454026.6	Isoform 2 of Zinc finger CCCH domain-containing protein 18	54.11%	1	2	20	117																			-0.149	0.016	-0.188	0.043	0.102	5							316	34.7	4.31
IPI00854952.1	Isoform 2 of Zinc transporter 9	48.82%	4	3	39	89																									-0.262	0.267	-0.019	0.179	-0.238	3	592	65.1	8.63
IPI00134941.1	Isoform 2B of GTPase KRas	83.51%	3	3	22	121	0.066	-0.028	0.120	-0.048	0.096	6	-0.059	0.154	-0.062	-0.029	0.001	7	0.361	0.000	0.323	0.107	0.143	3	-0.407	-0.219	-0.390	-0.131	-0.233	6	0.087	0.151	-0.009	0.178	0.126	18	188	21.5	8.12
IPI00407868.1	Isoform 2D of Voltage-dependent calcium channel subunit alpha-2/delta-1	66.21%	6	16	72	315																									0.059	-0.029	-0.048	-0.042	0.016	38	1086	122.6	5.25
IPI00131369.1	Isoform 2L of Gamma-aminobutyric acid receptor subunit gamma-2	60.13%	3	2	31	89																									-0.048	0.132	0.094	0.158	0.253	4	474	55.1	8.66
IPI00556820.1	Isoform 3 of ADP-dependent glucokinase	52.93%	3	4	18	75							0.554	0.020	0.695	-0.010	0.602	8							-0.383	0.023	-0.476	-0.050	-0.302	6							495	53.7	5.62
IPI00228697.4	Isoform 3 of Ankyrin-2	57.43%	1	1	70	337																									-0.026	-0.048	-0.045	-0.019	-0.062	89	1050	117.4	4.92
IPI00420171.1	Isoform 3 of Arf-GAP domain and FG repeats-containing protein 1	50.00%	8	8	33	241	0.000	-0.039	0.139	0.006	0.038	19	-0.550	-0.233	-0.624	-0.285	-0.610	6							-0.368	-0.209	-0.476	-0.050	-0.375	12	0.017	-0.136	-0.062	-0.117	-0.020	4	530	55.0	8.47
IPI00229295.1	Isoform 3 of Band 4.1-like protein 3	93.35%	9	1	108	811	-0.141	0.033	-0.071	-0.060	-0.072	3	0.270	0.193	0.193	0.267	-0.109	10	0.108	-0.042	0.083	-0.080	0.141	24	-0.427	-0.100	-0.450	-0.031	-0.434	35	0.019	0.208	0.160	0.157	0.111	152	917	101.9	5.40
IPI00406624.2	Isoform 3 of Bifunctional protein NCOAT	79.77%	3	6	87	294	0.001	-0.035	0.041	0.007	-0.035	4													-0.302	0.079	-0.144	-0.016	-0.431	9	0.009	-0.214	-0.292	-0.315	-0.311	9	954	107.3	5.12
IPI00944734.1	Isoform 3 of Catenin alpha-2	76.69%	5	13	99	537	0.231	0.081	0.210	0.054	0.265	13	0.209	-0.154	0.303	0.072	0.089	11	0.108	0.064	0.110	-0.016	0.057	22							0.096	0.116	-0.068	-0.053	-0.006	21	918	101.8	5.94
IPI00316623.4	Isoform 3 of Catenin delta-1	88.88%	5	1	108	668	0.246	0.026	0.258	0.000	0.236	71	0.262	-0.020	0.274	-0.042	0.050	22	0.083	0.068	0.037	-0.004	0.006	55	0.513	-0.003	0.269	-0.125	0.181	4							917	102.5	6.87
IPI00461967.2	Isoform 3 of Cell adhesion molecule 1	89.02%	9	9	38	257	-0.484	-0.139	-0.723	0.399	-0.704	8	-0.193	0.029	-0.125	0.231	-0.279	24													-0.022	-0.039	-0.160	-0.077	-0.045	3	428	46.9	5.08
IPI00454151.1	Isoform 3 of Cellular nucleic acid-binding protein	79.66%	2	1	19	56	-0.100	-0.032	0.089	0.044	-0.178	3	0.149	0.047	-0.370	-0.580	-0.044	12	0.054	0.009	0.024	-0.038	-0.050	3													177	19.4	7.71
IPI00408269.4	Isoform 3 of Clathrin coat assembly protein AP180	42.05%	7	24	49	383																			-0.584	0.176	-0.969	-0.056	-0.417	7	0.233	-0.233	-0.224	-0.206	-0.147	104	868	88.8	4.91
IPI00125861.2	Isoform 3 of Disks large homolog 1	79.07%	3	1	76	377	0.014	0.051	-0.012	0.098	0.000	12	0.444	0.158	0.437	0.399	0.522	13													0.029	0.128	0.073	0.081	0.140	28	927	102.9	5.96
IPI00626797.1	Isoform 3 of Disks large homolog 4	61.03%	7	14	47	227																									0.057	0.102	0.064	0.088	0.148	42	721	80.1	5.92
IPI00465648.3	Isoform 3 of Dynamin-1	95.42%	6	3	132	1051	-0.125	-0.039	-0.181	-0.045	-0.236	5	0.091	0.098	0.043	0.004	0.058	5							-0.300	0.016	-0.329	-0.188	-0.390	12	0.069	-0.047	-0.057	-0.116	-0.065	226	851	95.9	6.76
IPI00556781.1	Isoform 3 of Dynamin-1-like protein	96.14%	2	2	90	663	-0.106	-0.105	-0.193	-0.060	-0.094	7	-0.059	-0.020	-0.042	-0.141	0.037	30	-0.394	-0.220	-0.419	-0.178	-0.258	5	-0.097	0.068	-0.028	-0.017	-0.082	32	-0.013	-0.097	-0.108	-0.180	-0.102	97	699	78.0	6.81
IPI00845523.1	Isoform 3 of E3 ubiquitin-protein ligase UBR4	79.91%	5	42	441	1733	0.031	-0.012	0.036	-0.017	0.058	31	0.084	0.126	0.073	0.289	0.194	10	-0.892	-0.256	-0.546	-0.492	-0.423	3	-0.241	0.152	-0.189	0.102	-0.198	34							5156	569.9	6.06
IPI00944009.1	Isoform 3 of Elongation factor 1-delta	79.70%	5	6	67	839	-0.091	-0.014	-0.063	-0.035	-0.091	62	0.183	-0.050	0.144	-0.048	0.072	52	-0.184	-0.043	-0.134	0.065	-0.119	70	0.749	0.112	0.687	0.055	0.597	52	-0.019	-0.203	-0.144	-0.199	-0.158	13	660	72.9	6.43
IPI00468079.3	Isoform 3 of Engulfment and cell motility protein 2	82.96%	5	5	75	289																									0.072	-0.042	-0.009	-0.130	-0.260	4	798	91.2	6.57
IPI00420931.1	Isoform 3 of ERC protein 2	85.93%	11	9	143	463																									-0.066	-0.106	-0.139	-0.150	-0.138	19	1002	115.4	7.58
IPI00407312.3	Isoform 3 of Fatty acyl-CoA reductase 1	95.73%	7	12	54	325	0.108	0.031	-0.042	0.065	0.102	20	0.098	0.152	-0.076	-0.003	-0.074	12	0.180	0.070	0.200	0.001	0.070	23	-0.029	-0.157	-0.146	-0.007	-0.032	11							515	59.3	9.13
IPI00943452.1	Isoform 3 of Flotillin-2	91.82%	5	11	54	184							0.029	0.019	-0.201	-0.016	0.069	6	0.073	0.094	0.027	-0.031	-0.127	6							-0.063	0.239	0.214	0.192	0.257	9	379	41.6	5.22
IPI00404687.5	Isoform 3 of Glycerol kinase	51.34%	4	7	34	120																									0.209	-0.014	0.218	0.184	0.179	19	559	61.2	5.87
IPI00116442.1	Isoform 3 of Hepatoma-derived growth factor-related protein 2	62.83%	4	12	56	294	-0.071	-0.032	-0.079	0.001	-0.017	13	-0.108	-0.116	-0.240	0.108	-0.279	21	0.169	0.183	0.076	0.102	0.198	6	-0.032	-0.108	-0.010	-0.044	0.020	15	-0.029	0.095	0.020	0.073	-0.035	3	678	75.2	8.59
IPI00336873.1	Isoform 3 of Heterogeneous nuclear ribonucleoprotein D0	70.26%	9	13	30	457	0.115	0.057	0.104	0.036	0.014	25	-0.498	-0.038	-0.440	0.077	-0.653	30	0.184	0.062	0.215	-0.072	0.126	35	0.242	0.094	0.396	0.087	0.228	29	0.030	0.194	0.215	0.084	0.148	30	306	32.7	8.16
IPI00405058.6	Isoform 3 of Heterogeneous nuclear ribonucleoproteins A2/B1	99.34%	1	1	48	2132	-0.218	0.089	-0.109	0.091	-0.175	96	-0.056	-0.042	-0.193	-0.020	-0.152	112	0.275	0.098	0.335	-0.020	0.169	81	0.128	-0.057	0.217	-0.023	0.151	126	0.003	0.224	0.273	0.207	0.262	54	301	32.4	8.72
IPI00223444.1	Isoform 3 of Heterogeneous nuclear ribonucleoproteins C1/C2	98.29%	2	1	56	950	0.054	0.104	0.011	0.059	-0.014	89	0.212	-0.080	0.223	0.034	0.181	53	0.356	0.122	0.315	-0.020	0.208	71	0.174	-0.006	0.235	0.001	0.080	92	-0.010	0.089	0.128	0.066	0.081	25	293	32.3	5.08
IPI00896029.2	Isoform 3 of Inositol 1,4,5-trisphosphate receptor type 2	77.66%	7	4	259	954																									-0.158	-0.152	0.149	0.099	-0.001	21	2668	303.8	6.47
IPI00130390.5	Isoform 3 of Kinesin-like protein KIF1B	90.09%	4	3	154	578	0.130	-0.133	0.065	-0.329	-0.057	3	-0.142	0.148	0.276	-0.076	0.036	7													0.145	-0.114	-0.106	-0.257	-0.155	6	1150	130.0	8.34
IPI00856328.1	Isoform 3 of Kinesin-like protein KIFC1	75.12%	10	7	68	319							-0.175	-0.050	-0.150	-0.091	-0.265	5							-0.007	-0.105	0.075	-0.013	0.333	10							611	67.1	8.59
IPI00480372.3	Isoform 3 of Liprin-beta-1	88.47%	7	14	119	395	-0.206	-0.048	-0.260	-0.198	-0.255	4	0.119	0.013	0.066	0.108	-0.050	30							-0.092	-0.175	0.290	-0.041	0.362	3							980	109.8	5.60
IPI00875638.1	Isoform 3 of Mitochondrial inner membrane protein	94.08%	7	1	104	1276	-0.054	0.058	-0.006	0.013	-0.102	68	0.099	0.023	0.023	0.047	0.007	48	0.415	0.099	0.341	-0.035	0.244	66	-0.141	-0.069	-0.023	-0.009	-0.057	56	0.013	0.001	0.000	-0.056	-0.029	129	709	78.7	7.25
IPI00754649.2	Isoform 3 of Myosin-Ic	81.23%	7	37	119	812	0.030	0.024	0.055	0.003	0.080	73	0.657	0.050	0.612	0.096	0.538	86	-0.065	-0.013	-0.034	0.040	-0.130	31							0.052	-0.029	0.171	0.072	0.252	5	1044	119.8	9.39
IPI00662285.1	Isoform 3 of Neuroplastin	81.59%	1	2	29	200	0.020	0.128	-0.080	-0.009	0.004	3	0.560	0.204	0.483	0.214	0.210	5	0.103	0.119	0.119	0.167	0.014	10							-0.020	-0.012	0.091	0.152	0.066	41	277	30.8	7.49
IPI00387232.1	Isoform 3 of NSFL1 cofactor p47	87.63%	4	16	43	525	0.040	-0.114	-0.047	-0.025	-0.029	32	-0.692	-0.082	-0.655	-0.076	-0.639	18	-0.448	-0.016	-0.267	0.072	0.066	26	-0.094	0.004	-0.006	-0.072	0.029	40	0.118	0.107	0.096	-0.029	-0.056	17	372	40.9	5.20
IPI00229962.1	Isoform 3 of Protein arginine N-methyltransferase 1	86.59%	8	10	39	267	-0.269	-0.041	-0.315	0.000	-0.308	29	-0.333	-0.017	-0.265	-0.057	-0.229	12	-0.127	-0.081	-0.226	0.110	-0.080	18	0.127	0.016	-0.102	0.040	-0.128	34	0.089	0.139	0.253	0.423	0.379	4	343	39.6	5.71
IPI00620325.2	Isoform 3 of Protein NDRG4	70.05%	2	2	22	81																									-0.035	-0.123	-0.189	-0.031	0.047	18	404	44.4	6.23
IPI00882124.1	Isoform 3 of Protein rogdi homolog	84.04%	3	5	22	77																									0.083	-0.044	-0.103	-0.111	-0.181	10	213	23.8	7.11
IPI00400350.1	Isoform 3 of Pumilio homolog 1	45.88%	6	6	53	223	0.217	0.051	0.006	0.006	0.007	5	0.094	0.030	-0.105	0.064	-0.193	13	-0.017	-0.007	-0.062	-0.057	0.085	4	-0.139	-0.139	-0.083	-0.080	-0.236	7							1188	126.5	6.86
IPI00762713.1	Isoform 3 of Putative tyrosine-protein phosphatase auxilin	60.74%	5	12	60	210																									0.081	-0.144	-0.071	-0.089	-0.089	32	968	105.3	7.47
IPI00323505.5	Isoform 3 of Rap guanine nucleotide exchange factor 4	68.98%	7	5	89	215																									-0.134	-0.146	-0.194	0.021	-0.357	7	993	113.4	6.83
IPI00121149.1	Isoform 3 of Ribosome-binding protein 1	91.84%	12	84	218	2634	0.024	-0.014	0.052	-0.068	-0.012	218	0.228	0.084	0.136	0.123	-0.032	134	0.044	0.074	0.166	-0.062	-0.025	158	-0.003	-0.060	0.066	-0.042	0.057	122	0.026	0.055	0.044	0.061	0.118	6	1605	172.8	9.33
IPI00462900.1	Isoform 3 of RNA-binding protein Musashi homolog 2	69.50%	8	4	28	104																			0.040	-0.069	0.009	0.031	-0.095	3	0.414	0.058	0.544	0.156	0.343	3	282	30.9	9.10
IPI00226417.3	Isoform 3 of Serine/threonine-protein kinase DCLK2	81.06%	6	3	78	271																									-0.165	-0.290	0.068	-0.074	-0.004	10	771	84.4	8.62
**IPI00312101.1**	**Isoform 3 of SH3 domain-containing kinase-binding protein 1**	**69.90%**	**9**	**6**	**54**	**134**							-0.980	-0.181	-1.062	0.091	-0.824	**4**																			**628**	**68.8**	**7.46**
IPI00605111.1	Isoform 3 of Transcription factor Sp3	16.69%	4	1	12	36	-0.054	0.061	-0.050	0.077	-0.032	4																									719	76.7	6.33
IPI00816988.1	Isoform 3 of Transforming acidic coiled-coil-containing protein 2	59.66%	7	4	67	176	0.247	0.007	-0.116	0.062	0.065	4	0.152	-0.089	0.213	-0.041	0.010	8																			947	103.2	4.93
IPI00742413.3	Isoform 3 of U4/U6 small nuclear ribonucleoprotein Prp31	77.37%	5	8	50	270	0.068	0.043	0.052	-0.057	0.000	6	0.107	0.026	0.108	0.050	-0.117	3	-0.181	-0.136	-0.122	-0.278	0.010	7	0.052	0.006	0.075	-0.106	-0.057	4							495	55.0	5.90
IPI00120915.2	Isoform 3 of UPF0587 protein C1orf123 homolog	67.35%	7	4	20	103							-0.398	-0.338	-0.288	-0.413	-0.322	3																			196	22.0	6.57
IPI00626660.3	Isoform 3 of Voltage-dependent L-type calcium channel subunit beta-4	60.31%	17	5	47	128																									0.021	-0.019	-0.082	0.084	0.073	9	519	57.9	9.28
IPI00227149.4	Isoform 3 of YTH domain family protein 3	48.32%	4	7	35	250	-0.032	0.051	-0.016	0.044	-0.016	16	-0.158	0.027	-0.216	-0.034	-0.180	17	-0.216	-0.074	-0.127	-0.109	-0.141	11	-0.421	-0.390	-0.112	-0.103	0.027	4							596	65.4	9.23
IPI00788350.1	Isoform 3 of Zinc finger protein 598	63.31%	4	2	60	175	-0.288	0.098	0.195	-0.178	-0.246	3																									905	98.7	8.25
IPI00751423.1	Isoform 3a of C-Jun-amino-terminal kinase-interacting protein 3	69.29%	9	7	94	295																			-0.023	0.040	-0.028	0.043	-0.186	10							1322	145.7	5.49
IPI00454179.3	Isoform 4 of Abl interactor 1	75.37%	10	6	43	131							0.047	-0.037	-0.072	-0.224	-0.240	9							0.094	-0.004	0.119	-0.025	-0.083	3	-0.076	-0.091	-0.128	-0.039	-0.167	8	475	51.6	7.06
IPI00404055.5	Isoform 4 of Ankyrin-2	76.16%	13	1	350	1606							0.123	-0.071	0.079	0.139	0.215	3													-0.001	-0.026	-0.004	-0.031	-0.053	190	3959	432.6	5.16
IPI00848909.1	Isoform 4 of Anoctamin-10	65.97%	4	2	40	89							0.432	0.154	0.398	-0.233	0.708	3																			670	77.2	7.03
IPI00922962.2	Isoform 4 of Apoptotic chromatin condensation inducer in the nucleus	68.18%	13	22	142	688	-0.082	0.030	0.001	-0.004	-0.006	32	0.279	0.135	0.144	0.222	0.322	23	0.241	0.042	0.214	-0.150	0.175	21	-0.044	-0.013	0.145	-0.056	-0.032	27							1298	146.2	6.13
IPI00896657.1	Isoform 4 of Coatomer subunit gamma-2	90.34%	4	6	90	394	0.102	-0.026	0.070	-0.045	0.038	19	0.023	0.171	0.083	0.144	-0.155	12							0.167	0.122	0.190	0.054	0.052	17							870	97.5	5.86
IPI00395213.1	Isoform 4 of CUGBP Elav-like family member 1	59.26%	6	9	38	208	-0.194	-0.167	-0.196	-0.105	-0.063	12							0.019	0.120	0.188	0.038	0.157	15	0.194	0.047	0.066	0.045	0.190	7	-0.198	-0.102	0.048	0.214	0.095	7	513	55.1	8.38
IPI00381563.4	Isoform 4 of Cytospin-B	83.18%	3	19	102	494	0.104	0.195	0.069	0.103	0.122	15	0.341	-0.117	0.255	-0.034	0.220	26	0.141	0.187	-0.013	-0.023	-0.041	17													987	109.8	5.86
IPI00331293.3	Isoform 4 of Dynamin-1	95.02%	10	4	130	1065	-0.125	-0.039	-0.181	-0.045	-0.236	5	0.091	0.098	0.043	0.004	0.058	5	-0.283	-0.041	-0.031	-0.108	-0.155	3	-0.327	-0.019	-0.351	-0.188	-0.390	14	0.076	-0.047	-0.054	-0.119	-0.062	229	864	97.4	7.17
IPI00453615.4	Isoform 4 of E3 ubiquitin-protein ligase HUWE1	75.00%	5	1	327	1526	-0.288	0.009	-0.183	-0.109	-0.248	21	0.119	0.003	0.163	0.045	0.072	44							-0.089	0.031	-0.097	0.029	-0.063	31	-0.160	-0.295	-0.095	-0.154	-0.170	4	3832	419.2	5.21
IPI00608015.1	Isoform 4 of Glutamate receptor 2	72.64%	5	13	74	273																									0.085	-0.004	0.047	0.091	0.038	37	921	102.8	7.72
IPI00515349.1	Isoform 4 of Mitochondrial Rho GTPase 1	86.93%	6	4	70	156							0.596	-0.006	0.546	0.091	0.669	5													-0.184	-0.053	0.040	0.057	0.079	3	704	80.8	7.09
IPI00555136.2	Isoform 4 of Muscleblind-like protein 2	50.92%	12	6	23	90	-0.076	-0.013	0.030	-0.050	0.099	8	0.158	0.131	0.045	0.010	0.079	11	-0.044	0.063	0.009	-0.092	0.004	9	0.096	-0.269	0.034	-0.057	0.062	4							379	41.3	8.50
IPI00223377.1	Isoform 4 of Myelin basic protein	92.82%	3	0	34	377																									0.055	0.341	0.409	0.263	0.309	121	195	21.5	11.24
IPI00752192.1	Isoform 4 of Protein piccolo	65.19%	6	37	368	1418																									-0.032	-0.105	-0.106	-0.083	-0.116	73	5177	563.1	6.55
IPI00943997.1	Isoform 4 of Triple functional domain protein	56.62%	18	6	181	473																									-0.095	-0.245	-0.136	-0.083	-0.134	9	3103	347.7	6.35
IPI00755395.1	Isoform 5 of Ankyrin-1	79.30%	14	9	159	559																									0.014	-0.085	0.108	0.079	0.080	24	1744	191.9	7.87
IPI00830892.1	Isoform 5 of Collagen alpha-1(XII) chain	74.32%	7	23	268	961							-1.340	0.185	-1.466	0.301	-1.248	24																			3068	333.9	5.80
IPI00828355.1	Isoform 5 of Formin-1	63.21%	6	4	103	317							0.544	0.062	0.218	-0.402	0.957	4																			1204	133.1	5.71
IPI00776206.1	Isoform 5 of Homeobox protein cut-like 1	66.67%	9	11	144	456	-0.026	-0.088	-0.088	-0.106	-0.009	4	-0.092	-0.216	-0.077	0.070	-0.168	13																			1515	165.5	6.32
IPI00223378.1	Isoform 5 of Myelin basic protein	97.04%	4	0	32	377																									0.037	0.341	0.408	0.263	0.308	121	169	18.5	11.15
IPI00720036.1	Isoform 5 of Myosin phosphatase Rho-interacting protein	82.31%	9	1	106	351	-0.057	-0.022	0.065	-0.014	0.069	9	0.654	0.124	0.720	0.076	0.588	24	0.020	-0.017	-0.221	0.017	-0.003	7													1029	116.7	6.18
IPI00119517.3	Isoform 5 of Peripheral plasma membrane protein CASK	70.43%	10	12	81	209	0.044	0.030	-0.025	0.038	0.021	5	0.077	-0.031	0.384	-0.119	-0.041	3	0.382	0.211	0.192	-0.311	-0.194	3							0.023	-0.074	0.072	-0.006	-0.032	12	920	104.4	6.43
IPI00321378.3	Isoform 5 of Phosphatidylinositol-binding clathrin assembly protein	72.98%	3	1	59	366	0.345	0.116	0.306	0.069	0.253	14	0.080	0.011	0.147	0.209	-0.056	13	0.140	-0.043	0.036	-0.029	0.240	14	-0.193	0.051	-0.218	0.010	-0.097	16	0.072	-0.082	-0.122	-0.260	-0.120	24	655	70.9	8.37
IPI01008259.1	Isoform 5 of Protein enabled homolog	49.25%	9	5	42	144	0.001	-0.062	-0.041	-0.141	0.043	5													-0.317	-0.094	-0.480	-0.019	-0.326	5	-0.154	-0.154	-0.102	-0.077	-0.171	4	802	85.8	7.72
IPI00900398.1	Isoform 5 of Sorbin and SH3 domain-containing protein 2	82.74%	14	1	67	360	-0.068	0.050	-0.047	0.048	-0.023	57							0.079	0.059	-0.100	0.100	-0.054	10													707	78.1	9.33
IPI00856877.1	Isoform 5 of Voltage-dependent calcium channel subunit alpha-2/delta-2	59.57%	6	7	53	128																									-0.053	0.014	0.010	0.085	-0.149	7	1150	129.9	5.60
IPI00129527.3	Isoform 5g of Peripherin	96.84%	6	4	82	798	0.386	0.189	0.245	0.016	0.286	6	0.220	-0.010	0.099	-0.009	0.084	9	0.181	0.146	0.100	-0.001	-0.026	25	-0.431	0.027	-0.415	0.021	-0.377	145	0.076	-0.158	-0.160	-0.122	0.037	11	475	54.2	5.52
IPI00395163.1	Isoform 6 of General transcription factor II-I	74.51%	12	15	99	525	0.059	0.123	0.218	0.052	0.010	18	0.076	-0.066	-0.029	-0.130	-0.044	10	0.407	0.087	0.350	0.144	0.187	8	0.157	-0.019	0.194	-0.045	0.212	19							977	109.9	7.62
IPI00223379.1	Isoform 6 of Myelin basic protein	92.86%	3	2	32	342																									0.001	0.335	0.402	0.267	0.290	100	154	17.2	11.75
IPI00856302.1	Isoform 6 of Palladin	65.71%	8	18	95	453							-0.238	-0.041	-0.390	0.017	-0.419	35	-0.436	-0.110	-0.470	-0.047	-0.022	7													1391	150.5	6.24
IPI00404438.1	Isoform 6 of Phosphatidylinositol-binding clathrin assembly protein	74.08%	3	1	58	351	0.279	0.038	0.327	0.083	0.282	10	0.081	0.011	0.127	0.208	-0.062	14	0.140	-0.043	0.036	-0.029	0.240	14	-0.181	0.059	-0.196	0.066	-0.076	15	0.072	-0.082	-0.122	-0.260	-0.120	24	652	70.5	7.90
IPI00759871.1	Isoform 6 of Protein quaking	67.58%	9	7	30	202	-0.283	-0.127	-0.231	-0.077	-0.214	8	0.144	0.068	-0.019	-0.006	-0.112	14	-0.228	-0.085	-0.007	0.224	-0.054	18													330	36.6	7.91
IPI00751703.1	Isoform 9 of CUGBP Elav-like family member 2	52.47%	13	7	43	200	-0.086	-0.074	-0.189	-0.071	-0.077	7							-0.038	0.089	0.183	-0.006	-0.240	12							-0.223	-0.097	-0.032	0.165	-0.209	9	526	56.3	8.40
IPI00130045.2	Isoform A of 1-phosphatidylinositol-4,5-bisphosphate phosphodiesterase be	73.93%	5	30	97	326																									0.004	0.108	0.102	0.065	0.003	61	1216	138.3	6.13
IPI00120764.3	Isoform A of Coronin-6	48.20%	5	1	22	39	0.114	-0.085	0.234	-0.037	0.214	4																									471	52.6	5.96
IPI00119896.2	Isoform A of DnaJ homolog subfamily B member 6	63.84%	5	6	26	73	-0.100	-0.042	-0.023	-0.116	-0.146	3	0.013	-0.088	-0.213	0.045	-0.007	5	-0.635	-0.339	-0.213	-0.186	-0.108	5							-0.114	0.004	0.244	0.207	0.172	5	365	39.8	9.36
IPI00135475.3	Isoform A of Drebrin	68.13%	6	22	53	526							0.364	-0.088	0.364	-0.032	0.414	33	0.235	-0.021	0.244	-0.181	0.235	27	-0.582	-0.034	-0.662	-0.035	-0.502	44	-0.026	-0.014	-0.077	-0.074	-0.138	47	706	77.2	4.49
IPI00128522.1	Isoform A of Heat shock protein beta-1	96.17%	5	15	26	281	0.145	-0.160	0.047	-0.139	0.076	12	0.666	-0.288	0.366	-0.533	0.833	9	0.059	-0.137	0.058	0.208	0.136	61													209	23.0	6.55
IPI00131063.4	Isoform A of Methyl-CpG-binding protein 2	85.74%	4	12	63	311																									0.058	0.280	0.343	0.295	0.222	24	484	52.3	9.96
IPI00620256.3	Isoform A of Prelamin-A/C	96.39%	4	51	128	2532	0.052	0.047	0.068	-0.004	0.013	226	0.174	0.061	0.034	0.024	0.040	212	0.303	0.098	0.233	0.000	0.184	246	0.310	0.011	0.296	0.004	0.244	79	-0.048	0.054	0.034	0.065	0.070	42	665	74.2	6.98
IPI00124245.1	Isoform A of Ras GTPase-activating protein-binding protein 2	76.35%	1	1	40	265	0.092	0.128	0.108	0.051	-0.007	11	-0.188	-0.105	-0.112	0.036	-0.147	25	0.045	0.066	0.123	-0.053	0.076	19	0.193	0.047	0.127	0.135	0.081	5	-0.114	0.045	0.013	0.107	-0.056	11	482	54.1	5.62
IPI00129145.1	Isoform A of SWI/SNF-related matrix-associated actin-dependent regulator	74.03%	4	6	38	155																			-0.088	-0.037	0.045	-0.013	-0.086	6							385	44.1	6.23
IPI00228525.3	Isoform A1-III of V-type proton ATPase 116 kDa subunit a isoform 1	60.70%	9	4	61	419	0.295	0.184	0.476	0.148	0.302	6							0.193	0.026	0.122	0.055	0.054	4	-0.051	0.089	-0.178	0.132	-0.123	8	0.126	-0.147	-0.178	-0.178	-0.149	118	832	95.6	6.77
IPI00119042.2	Isoform ALF1B of Transcription factor 12	75.64%	7	1	50	177																			-0.138	-0.216	-0.031	-0.007	0.045	6							706	75.8	6.90
IPI00621806.2	Isoform Alpha CaMKII of Calcium/calmodulin-dependent protein kinase typ	70.08%	6	17	58	572	-0.139	-0.158	-0.103	-0.155	-0.152	11	-0.241	-0.162	-0.438	-0.293	-0.488	5	0.111	0.066	0.180	0.118	0.172	12	0.190	0.207	0.111	0.061	0.084	4	0.051	0.083	-0.041	-0.009	-0.014	118	478	54.1	7.08
IPI00117829.1	Isoform Alpha of Caveolin-1	68.54%	5	7	11	164	0.075	0.144	0.048	0.085	0.068	29	0.065	0.062	0.160	0.215	0.001	22	0.295	0.070	0.275	0.013	0.236	6													178	20.5	6.02
IPI00129175.1	Isoform Alpha of Disintegrin and metalloproteinase domain-containing prot	79.61%	9	6	64	151																									-0.020	0.230	0.131	0.274	0.148	10	829	91.5	7.68
IPI00126338.6	Isoform Alpha of Lamina-associated polypeptide 2, isoforms alpha/zeta	92.06%	1	20	86	898	-0.037	0.013	0.070	0.062	-0.035	47	0.052	0.066	0.108	0.134	-0.028	60	0.276	0.033	0.193	0.001	0.147	67	0.384	-0.016	0.445	-0.031	0.324	55	-0.038	-0.138	-0.324	0.199	0.171	3	693	75.1	8.05
IPI00314316.3	Isoform Alpha of MAGUK p55 subfamily member 6	76.81%	3	12	50	194																			-0.240	-0.123	-0.050	-0.116	-0.142	4	-0.012	0.079	-0.026	0.026	0.055	21	539	60.9	6.18
IPI00230192.5	Isoform Alpha-1 of Guanine nucleotide-binding protein G(o) subunit alpha	88.42%	7	4	44	397	0.160	0.026	0.112	-0.037	0.020	5							0.195	0.113	0.209	-0.039	0.183	3	-0.464	-0.035	-0.660	-0.034	-0.462	10	0.100	0.070	0.080	0.020	0.048	115	354	40.1	5.53
IPI00115546.4	Isoform Alpha-2 of Guanine nucleotide-binding protein G(o) subunit alpha	85.31%	5	1	43	392	0.160	0.026	0.001	0.009	0.048	7							0.246	0.064	0.244	-0.039	0.153	7	-0.464	-0.026	-0.664	-0.029	-0.484	11	0.116	0.110	0.112	0.033	0.061	104	354	40.0	6.01
IPI00123875.1	Isoform Alpha-2 of Mitogen-activated protein kinase 9	61.47%	12	1	33	96																									-0.100	-0.066	-0.180	-0.324	-0.120	5	423	48.2	5.97
IPI00420835.4	Isoform Alpha-6X1B of Integrin alpha-6	78.09%	4	9	117	311	-0.022	-0.109	0.040	-0.007	-0.097	8													-0.221	-0.362	-0.630	-0.146	-0.007	4							1091	122.1	7.03
IPI00114389.4	Isoform APP770 of Amyloid beta A4 protein (Fragment)	55.19%	6	7	38	162													-0.492	0.067	-0.565	-0.062	-0.711	5							0.052	0.010	-0.079	-0.131	-0.013	11	770	86.7	4.79
IPI00130961.2	Isoform ATE1-1 of Arginyl-tRNA--protein transferase 1	67.05%	3	3	33	97							-0.139	-0.283	-0.042	-0.142	-0.109	3																			516	59.1	8.19
IPI00622911.1	Isoform B of AP-2 complex subunit alpha-1	90.79%	3	21	121	1030	0.038	0.091	0.073	-0.007	0.055	20	0.169	0.112	0.214	0.084	0.131	28	0.145	0.052	0.096	-0.009	0.003	33	-0.109	0.026	0.001	0.031	0.054	13	0.073	-0.034	-0.045	-0.063	-0.047	169	955	105.4	7.66
IPI00458068.6	Isoform B of FK506-binding protein 15	59.28%	4	4	103	383	0.052	0.100	0.040	-0.044	0.100	6	-0.139	-0.276	-0.112	-0.003	-0.238	3																			1255	137.1	5.27
IPI00132819.1	Isoform B of Huntingtin-associated protein 1	61.78%	2	2	34	97																									0.424	-0.180	-0.034	-0.097	0.286	3	628	70.1	4.74
IPI00331295.4	Isoform B of Ras GTPase-activating protein-binding protein 2	74.61%	1	1	36	288	0.088	0.128	0.132	0.048	0.122	15	-0.183	-0.181	-0.150	0.030	-0.154	26	0.024	0.063	0.080	-0.035	0.059	22	0.193	0.047	0.127	0.135	0.081	5	-0.094	0.061	0.001	0.045	0.024	10	449	50.8	5.43
IPI00655136.3	Isoform B of Rho guanine nucleotide exchange factor 7	74.01%	10	8	80	220																															862	97.0	6.80
IPI00126191.5	Isoform B2 of Lamin-B2	88.76%	2	23	79	527	0.019	0.026	0.019	-0.009	0.010	17	0.037	0.111	-0.023	-0.029	-0.071	18	0.099	0.064	0.120	-0.063	0.013	47	0.076	-0.013	0.259	-0.032	0.239	25	-0.162	0.003	0.120	0.050	-0.037	29	596	67.3	5.50
IPI00133454.3	Isoform Bcl-X(L) of Bcl-2-like protein 1	77.68%	8	3	17	54							-0.916	-0.373	-1.158	-0.340	-0.613	4																			233	26.1	4.94
IPI00230471.2	Isoform Beta of DNA ligase 3	60.67%	6	7	72	155							0.045	0.058	0.365	0.241	0.370	3							-0.125	0.270	-0.029	0.122	-0.069	3							956	106.4	9.22
IPI00320399.7	Isoform Beta of Lamina-associated polypeptide 2, isoforms beta/delta/epsil	93.14%	6	3	60	955	0.026	0.085	0.119	0.044	0.010	61	0.077	0.004	0.098	0.040	-0.020	51	0.386	0.096	0.229	-0.013	0.162	70	0.349	-0.016	0.445	-0.031	0.353	59	0.021	-0.136	0.055	0.027	0.157	4	452	50.3	9.45
IPI00759925.2	Isoform Beta of LIM domain and actin-binding protein 1	82.07%	2	25	98	486	0.583	0.065	0.634	0.004	0.547	30	-0.269	0.033	-0.255	-0.051	-0.333	34	-0.099	0.076	-0.114	-0.072	-0.099	33													753	84.0	6.60
IPI00165881.3	Isoform Beta of Paxillin	55.84%	3	9	35	152	0.295	-0.060	0.245	-0.026	0.251	11	-0.342	-0.026	-0.593	-0.223	-0.329	11																			591	64.4	6.02
IPI00109375.1	Isoform Beta of Poliovirus receptor-related protein 2	76.60%	2	2	31	104							0.051	-0.026	-0.196	0.161	-0.429	3																			530	57.3	4.86
IPI00227898.3	Isoform Beta-II of Protein kinase C beta type	75.04%	2	11	52	193																									-0.007	0.120	0.076	0.141	0.073	45	673	76.8	7.02
IPI00230599.1	Isoform C of Fragile X mental retardation syndrome-related protein 1	87.38%	12	27	72	575	0.098	0.156	-0.076	0.047	0.001	31	-0.183	0.004	-0.116	0.089	-0.189	42	-0.023	0.025	-0.013	-0.089	-0.048	39	-0.346	-0.051	-0.388	0.021	-0.274	34							650	72.8	6.27
IPI00224644.1	Isoform C of Serrate RNA effector molecule homolog	65.39%	4	19	75	627	-0.331	-0.037	-0.178	0.007	-0.278	34	-0.045	0.091	0.010	0.048	-0.032	38	0.184	-0.076	-0.041	-0.079	0.185	21	0.149	0.069	0.230	0.068	0.145	27							864	99.4	5.92
IPI00223443.1	Isoform C1 of Heterogeneous nuclear ribonucleoproteins C1/C2	98.33%	2	0	57	988	0.054	0.114	0.023	0.059	-0.013	89	0.219	-0.076	0.255	0.055	0.217	55	0.363	0.116	0.323	-0.035	0.210	79	0.180	-0.016	0.241	-0.007	0.089	94	-0.007	0.099	0.132	0.068	0.083	26	300	33.0	5.03
IPI00874321.1	Isoform C2 of Heterogeneous nuclear ribonucleoproteins C1/C2	98.40%	2	1	57	989	0.054	0.112	0.017	0.059	-0.013	88	0.279	-0.068	0.267	0.088	0.222	50	0.363	0.112	0.318	-0.035	0.208	77	0.130	0.019	0.229	-0.009	0.084	100	-0.007	0.099	0.132	0.068	0.083	26	313	34.4	5.05
IPI00752710.1	Isoform CBF1 of CCAAT/enhancer-binding protein zeta	72.43%	2	6	90	260													1.112	0.510	1.471	0.639	0.617	3	0.127	-0.214	0.148	-0.139	0.089	6							1052	120.2	5.74
IPI00124240.1	Isoform CDK2-beta of Cyclin-dependent kinase 2	54.62%	45	3	23	92																			0.497	0.019	0.495	-0.003	0.282	5							346	39.0	8.54
IPI00319602.3	Isoform CNPII of 2',3'-cyclic-nucleotide 3'-phosphodiesterase	94.52%	2	38	62	683													0.236	0.065	0.310	0.048	-0.108	3							0.034	0.336	0.270	0.239	0.309	223	420	47.1	8.97
IPI00307991.1	Isoform Crk-II of Adapter molecule crk	75.66%	6	13	28	188	-0.044	-0.085	-0.072	-0.131	0.023	19	-0.071	-0.181	0.085	-0.229	0.017	13	-0.214	-0.210	-0.533	-0.103	-0.209	5							0.004	0.091	0.011	-0.004	-0.092	18	304	33.8	5.55
IPI00759940.3	Isoform Cytoplasmic of Fumarate hydratase, mitochondrial	99.14%	2	27	63	1064	-0.125	-0.012	-0.091	-0.001	-0.023	53	-0.014	-0.019	0.043	0.077	0.021	28	0.162	0.105	0.075	-0.001	0.054	28	-0.079	-0.053	-0.035	-0.041	0.038	91	0.054	0.006	0.026	-0.020	0.034	79	467	50.0	7.94
IPI00227773.1	Isoform Gamma-2 of Serine/threonine-protein phosphatase PP1-gamma cat	81.31%	2	1	28	252	0.006	0.029	-0.050	0.166	-0.063	12	-0.199	-0.037	-0.106	0.006	-0.069	22	0.000	0.029	0.031	0.006	0.051	27	0.162	0.052	0.183	0.013	0.047	7	-0.071	-0.060	-0.083	-0.023	0.006	23	337	38.5	6.13
IPI00468633.5	Isoform GlyT-1B of Sodium- and chloride-dependent glycine transporter 1	31.80%	7	7	21	107																									-0.071	0.180	0.261	0.069	0.169	17	651	72.2	7.50
IPI00119853.1	Isoform Gnas-1 of Guanine nucleotide-binding protein G(s) subunit alpha iso	82.49%	9	14	46	332	0.006	0.023	0.011	-0.019	-0.057	22	-0.196	0.157	-0.302	0.112	-0.295	12	0.419	0.056	0.259	0.016	0.235	20	-0.464	-0.003	-0.552	-0.079	-0.398	7	-0.047	0.092	0.092	0.134	0.134	26	394	45.6	5.96
IPI00128360.1	Isoform GP145-TRKB of BDNF/NT-3 growth factors receptor	56.88%	4	1	43	111																									-0.150	0.124	-0.253	-0.100	-0.097	6	821	92.1	6.55
IPI00270098.1	Isoform GP95-TRKB of BDNF/NT-3 growth factors receptor	51.26%	2	1	24	73																									-0.091	0.120	-0.082	-0.100	-0.029	8	476	53.2	6.47
IPI00283611.2	Isoform HK1-SA of Hexokinase-1	91.38%	8	48	126	986	0.123	0.044	0.130	0.017	0.178	20	-0.029	-0.010	0.047	0.073	-0.037	14	0.145	-0.032	0.131	-0.037	0.111	52	0.235	-0.019	0.302	0.033	0.225	25	0.167	-0.154	-0.149	-0.214	-0.147	174	974	108.2	6.80
IPI00314240.5	Isoform HMG-I of High mobility group protein HMG-I/HMG-Y	82.24%	4	3	18	206	-0.257	0.036	-0.318	0.055	-0.388	16							0.277	0.121	0.277	-0.057	0.163	39	-0.173	0.102	-0.099	0.072	-0.152	10	-0.082	0.158	0.349	0.270	0.228	11	107	11.6	10.32
IPI00624711.3	Isoform HMG-Y of High mobility group protein HMG-I/HMG-Y	80.21%	2	1	13	94	-0.196	0.116	-0.279	0.050	-0.250	8							0.283	0.126	0.280	-0.039	0.208	24	0.114	0.038	-0.044	0.041	-0.203	3	-0.134	0.189	0.363	0.405	0.241	6	96	10.6	10.32
IPI00123802.5	Isoform HSP105-alpha of Heat shock protein 105 kDa	92.89%	4	42	108	1043	-0.031	-0.029	-0.022	-0.068	-0.038	45	-0.409	0.029	-0.490	-0.042	-0.385	47	-0.264	-0.147	-0.271	-0.004	-0.097	20	0.209	0.119	0.308	0.033	0.265	125	0.006	-0.253	-0.272	-0.392	-0.246	81	858	96.3	5.53
IPI00122451.1	Isoform HuC-L of ELAV-like protein 3	79.56%	2	4	36	158																			-0.257	0.088	-0.214	-0.039	-0.160	10	0.235	0.140	0.102	0.230	0.014	8	367	39.5	9.28
IPI00473707.3	Isoform I of Septin-6	76.11%	5	8	45	292	-0.091	0.188	0.068	0.095	-0.023	7	0.058	-0.031	-0.013	0.045	-0.173	9	-0.186	-0.109	-0.065	-0.053	0.076	8	0.091	0.108	0.052	0.051	-0.025	9	0.045	0.162	0.099	0.140	0.094	41	427	48.7	6.55
IPI00649886.1	Isoform Ia of Synapsin-1	83.29%	3	31	53	632																									0.247	-0.066	-0.136	-0.128	-0.076	213	706	74.1	9.80
IPI00222968.2	Isoform LAMP-2C of Lysosome-associated membrane glycoprotein 2	85.78%	3	7	33	246	-0.063	0.100	-0.162	-0.054	-0.152	8	-0.278	-0.045	0.016	-0.074	-0.053	9	0.180	0.105	0.054	-0.065	0.001	18							0.069	0.382	0.290	-0.109	0.636	4	415	45.6	7.84
IPI00129225.1	Isoform LMW of Kininogen-1	58.10%	7	4	31	134																									-0.099	0.135	0.095	-0.062	0.401	7	432	47.9	6.09
IPI00230682.6	Isoform Long of 14-3-3 protein beta/alpha	95.12%	4	10	35	1106	-0.006	-0.072	0.051	0.011	0.003	71	-0.739	-0.080	-0.786	-0.119	-0.561	60	-0.245	-0.125	-0.074	0.037	-0.042	63	0.288	0.085	0.346	0.062	0.265	47	0.104	0.011	0.014	-0.007	0.058	111	246	28.1	4.83
IPI00126940.1	Isoform Long of Adenosine kinase	76.45%	2	17	33	212	-0.248	-0.152	-0.158	-0.102	-0.125	13	0.189	0.091	0.176	0.001	0.072	16							0.325	0.044	0.367	0.024	0.234	12	0.055	0.066	0.044	0.100	0.144	8	361	40.1	6.21
IPI00129350.2	Isoform Long of Delta-1-pyrroline-5-carboxylate synthase	85.16%	1	1	98	804	0.019	0.061	0.083	0.029	0.055	33	0.469	0.126	0.523	0.148	0.336	69	0.045	0.066	0.036	0.036	-0.080	20	-0.146	-0.034	-0.211	-0.014	-0.131	77							795	87.2	7.55
IPI00129198.2	Isoform Long of Ephrin type-A receptor 4	63.18%	9	3	59	112													-0.069	0.014	0.064	-0.091	0.038	3							0.062	0.165	-0.158	0.029	0.048	8	986	109.7	6.51
IPI00124742.3	Isoform Long of Eukaryotic translation initiation factor 4H	89.92%	2	15	36	383	-0.041	0.092	0.263	0.044	0.143	9	-0.262	0.048	-0.191	-0.039	-0.257	16	0.016	-0.034	0.003	0.153	0.061	18	0.151	0.089	0.217	0.029	0.175	22	-0.083	-0.142	-0.022	-0.100	-0.146	16	248	27.3	7.23
IPI00133257.1	Isoform Long of Hematopoietic progenitor cell antigen CD34	49.48%	2	5	21	67							0.658	-0.010	0.619	0.107	0.419	8																			382	41.0	5.30
IPI00817004.1	Isoform Long of Heterogeneous nuclear ribonucleoprotein A1	98.13%	2	2	46	1997	0.126	0.036	0.075	0.019	0.092	79	-0.203	-0.028	-0.128	0.010	-0.183	58	0.120	0.068	0.118	-0.017	0.122	71	0.009	-0.054	0.070	0.016	0.104	115	0.072	0.256	0.233	0.258	0.301	17	320	34.2	9.23
IPI00131701.2	Isoform Long of Long-chain-fatty-acid--CoA ligase 4	89.03%	2	17	71	389	0.103	0.162	0.037	0.054	0.033	11	0.261	0.259	0.229	0.413	-0.097	15	0.318	0.059	0.066	-0.026	0.172	25	-0.381	0.004	-0.515	0.003	-0.411	27							711	79.0	8.28
IPI00556692.2	Isoform Long of Mothers against decapentaplegic homolog 2	64.03%	6	2	33	129																			-0.191	0.068	-0.193	-0.108	-0.213	7							467	52.2	6.58
IPI00127556.1	Isoform Long of Neural cell adhesion molecule 2	77.06%	4	16	86	270																									0.065	-0.023	-0.138	-0.150	-0.072	26	837	93.1	6.07
IPI00129323.1	Isoform Long of Serine/arginine-rich splicing factor 3	78.66%	4	7	23	481	0.006	0.011	-0.041	-0.032	-0.123	27	-0.076	0.214	0.021	0.134	-0.062	16	0.305	0.076	0.220	-0.100	0.240	37	-0.072	-0.147	-0.079	-0.079	-0.089	29	0.050	0.165	0.124	0.083	0.156	19	164	19.3	11.65
IPI00130997.2	Isoform Long of Thymidylate kinase	70.28%	3	3	17	60	-0.194	0.061	-0.059	0.031	-0.083	5																									212	23.9	7.90
IPI00323644.5	Isoform Long of Trifunctional purine biosynthetic protein adenosine-3	84.06%	3	33	107	722	-0.211	-0.080	-0.208	-0.012	-0.183	39	-0.053	0.036	0.036	-0.117	0.143	46	-0.604	-0.223	-0.434	0.134	-0.272	16	0.057	0.088	0.153	0.019	0.070	43	-0.154	0.200	0.151	0.147	-0.001	11	1010	107.4	6.68
IPI00469194.2	Isoform Long of Tripeptidyl-peptidase 2	78.45%	2	43	110	1187	-0.068	0.016	-0.163	-0.028	-0.102	69	-0.155	0.017	-0.211	0.016	-0.236	55	-0.262	-0.261	-0.379	-0.065	-0.472	10	-0.092	0.033	-0.039	0.045	-0.038	60	-0.077	-0.048	-0.063	0.033	0.017	20	1262	139.8	6.58
IPI00845840.1	Isoform M1 of Pyruvate kinase isozymes M1/M2	98.31%	1	4	73	2205	-0.103	-0.112	-0.072	-0.025	-0.062	185	-0.214	0.043	-0.066	-0.035	-0.062	116	-0.373	-0.193	-0.327	0.094	-0.158	177	-0.023	-0.020	0.061	-0.068	0.096	147	0.064	-0.050	-0.072	-0.082	0.011	261	531	57.9	7.14
IPI00407130.4	Isoform M2 of Pyruvate kinase isozymes M1/M2	98.31%	1	5	74	2269	-0.111	-0.114	-0.072	-0.023	-0.063	196	-0.226	0.038	-0.072	-0.035	-0.062	129	-0.373	-0.193	-0.322	0.100	-0.147	180	0.016	0.010	0.116	-0.072	0.124	147	0.070	-0.035	-0.063	-0.066	0.016	243	531	57.8	7.47
IPI00129517.1	Isoform Mitochondrial of Peroxiredoxin-5, mitochondria	88.57%	5	12	33	456	0.192	-0.133	0.222	-0.034	0.262	37	-0.523	-0.045	-0.315	-0.178	-0.262	21	0.174	-0.049	0.072	0.083	0.108	33	-0.095	-0.106	-0.077	-0.026	-0.125	4	0.188	-0.054	-0.004	-0.035	0.017	80	210	21.9	8.85
IPI00112822.2	Isoform Mitochondrial of Transcription factor A, mitochondrial	83.54%	4	10	31	188	0.029	0.070	0.013	0.040	-0.029	5							0.180	0.102	-0.122	-0.074	-0.152	13	0.210	-0.020	0.344	-0.060	0.370	9	0.210	-0.100	-0.012	0.151	0.088	21	243	28.0	9.69
IPI00319666.1	Isoform NFIX2 of Nuclear factor 1 X-type	62.25%	22	3	18	58							0.416	-0.208	0.153	0.064	0.058	5																			400	44.6	7.94
IPI00229030.1	Isoform NNOS Mu of Nitric oxide synthase, brain	71.57%	8	8	131	391																									0.006	-0.265	-0.010	-0.029	-0.001	8	1463	164.2	7.24
IPI00129487.5	Isoform p59-HCK of Tyrosine-protein kinase HCK	59.16%	45	1	37	109							-0.288	-0.065	-0.778	0.020	-0.340	3	0.098	-0.002	0.003	-0.039	-0.091	3													524	59.1	7.24
IPI00134188.3	Isoform p65 of Transcription factor p65	48.45%	3	3	30	66							-0.311	0.132	-0.180	-0.138	-0.019	6																			549	60.2	5.76
IPI00125298.2	Isoform p66Shc of SHC-transforming protein 1	68.05%	5	3	42	170							-0.193	0.123	0.055	-0.308	0.100	6																			579	62.6	6.54
IPI00308852.4	Isoform p96 of Disabled homolog 2	58.22%	8	8	49	200							-0.278	-0.035	-0.492	-0.069	-0.466	14																			766	82.3	6.10
IPI00121336.2	Isoform PDE1A2 of Calcium/calmodulin-dependent 3',5'-cyclic nucleotide pl	72.39%	6	4	44	123																									0.277	-0.250	-0.347	-0.404	-0.125	18	565	64.5	5.97
IPI00122549.1	Isoform Pl-VDAC1 of Voltage-dependent anion-selective channel protein 1	92.91%	3	18	35	1478	-0.112	0.102	-0.133	0.031	-0.103	81	0.441	0.038	0.396	0.104	0.318	68	0.441	0.147	0.331	0.010	0.154	77	0.135	-0.050	0.152	-0.013	0.197	101	0.172	-0.102	-0.074	-0.186	-0.114	150	296	32.3	8.43
IPI00623994.2	Isoform PTPA of Tyrosine-protein phosphatase non-receptor type 2	79.06%	3	2	41	108							-0.257	-0.176	-0.253	-0.056	0.047	5																			382	44.5	8.24
IPI01008405.1	Isoform RF1/RF2 of Retrotransposon-derived protein PEG10	43.53%	2	3	39	110																			1.605	0.016	1.558	-0.097	1.524	8							958	109.8	6.38
IPI00120107.2	Isoform RHAMM1V4 of Hyaluronan mediated motility receptor	88.41%	3	5	111	351	-0.122	-0.077	-0.154	-0.042	-0.080	15																									794	91.7	5.52
IPI00381291.1	Isoform Rpn10B of 26S proteasome non-ATPase regulatory subunit 4	63.59%	4	3	34	355	-0.016	-0.037	0.027	0.019	0.051	24	-0.025	-0.004	-0.016	0.017	0.051	19	-0.257	-0.128	-0.144	0.034	-0.082	15	0.103	0.123	0.122	0.062	-0.068	13	-0.042	-0.026	-0.057	0.059	0.138	5	379	41.0	4.79
IPI00230570.1	Isoform Rpn10D of 26S proteasome non-ATPase regulatory subunit 4	78.22%	1	2	31	319	-0.026	-0.035	0.024	0.024	0.019	19	-0.025	0.021	-0.006	-0.017	0.064	17	-0.193	-0.103	-0.123	0.037	-0.057	14	0.103	0.110	0.122	0.030	-0.071	11	-0.042	-0.026	-0.057	0.059	0.138	5	349	37.9	5.36
IPI00134131.2	Isoform SCPx of Non-specific lipid-transfer protein	64.72%	3	10	47	226	0.290	0.103	0.296	-0.006	0.185	16	-0.676	-0.211	-0.550	-0.010	-0.295	6	0.034	0.075	-0.102	-0.105	-0.221	13							0.021	0.169	0.058	0.076	0.208	9	547	59.1	7.44
IPI00338964.3	Isoform SERCA2B of Sarcoplasmic/endoplasmic reticulum calcium ATPase 2	70.31%	3	38	111	1287	0.107	0.087	0.052	0.013	0.034	91	0.099	0.014	-0.020	0.011	0.027	72	0.151	0.077	0.037	-0.097	-0.029	49	-0.026	-0.035	-0.019	-0.031	-0.065	71	-0.071	0.004	0.120	0.123	0.058	106	1044	114.8	5.34
IPI00336889.5	Isoform SERCA3B of Sarcoplasmic/endoplasmic reticulum calcium ATPase 3	64.16%	3	3	88	447	0.076	0.194	0.084	0.013	-0.147	7	0.072	-0.032	-0.116	-0.037	0.068	20	-0.044	0.035	-0.032	-0.184	-0.047	9	0.148	0.169	0.038	0.189	-0.054	3	-0.109	-0.038	0.132	0.110	0.076	20	1038	113.6	5.85
IPI00760067.1	Isoform Short of Beta-1,4-galactosyltransferase 1	56.22%	4	3	22	72	-0.039	0.058	-0.103	0.011	0.021	5																									386	42.9	9.31
IPI00221398.2	Isoform Short of Delta-1-pyrroline-5-carboxylate synthase	85.12%	1	1	98	734	-0.003	0.088	0.055	-0.009	0.044	28	0.454	0.119	0.452	0.156	0.286	67	-0.014	0.066	0.027	0.052	-0.080	18	-0.165	-0.041	-0.201	-0.012	-0.128	72							793	87.0	7.55
IPI00227467.1	Isoform Short of Transcription factor COE3	55.35%	15	2	36	70																			0.024	0.047	0.037	-0.130	0.047	3							551	60.4	8.81
**IPI00230151.1**	**Isoform S-MAG of Myelin-associated glycoprotein**	**50.69%**	**1**	**1**	**33**	**147**																									0.037	0.320	0.271	0.230	0.240	**37**	**582**	**64.2**	**5.08**
IPI00354819.5	Isoform Smooth muscle of Myosin light polypeptide 6	88.08%	9	10	19	603	0.229	-0.006	0.218	0.024	0.249	49	0.503	0.055	0.382	-0.059	0.318	33	-0.162	-0.112	-0.214	0.158	-0.034	53	0.207	0.073	0.139	0.010	0.210	42	-0.117	-0.123	-0.120	-0.141	-0.065	35	151	17.0	4.55
IPI00225389.1	Isoform SNAP-25a of Synaptosomal-associated protein 25	78.64%	1	2	29	241																									0.031	-0.009	0.019	0.016	-0.045	77	206	23.3	4.86
IPI00125635.1	Isoform SNAP-25b of Synaptosomal-associated protein 25	85.92%	1	2	36	278																									0.026	0.004	0.068	0.047	0.003	84	206	23.3	4.77
IPI00331265.8	Isoform Tau-D of Microtubule-associated protein tau	96.51%	7	1	60	441																			-0.673	-0.020	-0.727	-0.028	-0.711	26	0.136	0.123	0.051	0.149	0.111	81	372	38.9	9.50
IPI00225201.6	Isoleucyl-tRNA synthetase, cytoplasmic	74.96%	3	39	127	774	0.190	0.016	0.102	-0.010	0.112	41	0.276	0.134	0.381	0.054	0.401	46	0.087	-0.040	-0.077	0.262	0.114	15	-0.165	0.091	-0.198	0.036	-0.204	74							1262	144.2	6.55
IPI00453499.3	Isoleucyl-tRNA synthetase, mitochondrial	74.01%	4	25	92	369	-0.051	0.050	-0.117	-0.100	-0.160	8	0.122	0.065	0.102	-0.029	-0.035	22	-0.094	-0.050	-0.158	-0.053	-0.194	7	-0.085	0.098	-0.053	-0.063	0.054	4	0.122	-0.034	-0.032	-0.086	-0.038	26	1012	112.7	6.81
IPI00115850.2	isopentenyl-diphosphate Delta-isomerase 1	78.80%	2	5	28	104	0.553	0.092	0.629	0.040	0.631	5													0.212	-0.158	0.268	-0.141	0.474	3							283	32.5	7.02
IPI00471246.2	Isovaleryl-CoA dehydrogenase, mitochondrial	78.07%	1	7	37	127													0.220	0.144	0.147	-0.267	0.140	3							0.655	0.091	0.605	0.070	0.711	10	424	46.3	8.29
IPI00109389.1	IST1 homolog	64.09%	2	9	30	153	0.271	0.026	0.238	-0.022	0.208	6	-0.238	0.031	-0.045	0.036	-0.133	14							-0.012	-0.206	0.327	-0.223	0.379	6	0.200	0.354	0.188	0.013	-0.080	4	362	39.4	5.44
IPI00229475.1	Junction plakoglobin	84.70%	1	23	86	501	0.256	0.061	0.210	-0.029	0.222	40	0.194	0.000	0.059	-0.077	0.293	9	-0.042	0.103	-0.082	-0.089	-0.272	21							-0.103	-0.054	0.040	0.040	0.126	4	745	81.7	6.14
IPI00121362.2	junctional adhesion molecule A precursor	63.00%	2	3	19	73							-0.267	-0.063	0.059	0.021	0.131	4																			300	32.4	6.77
IPI00121859.4	Kelch domain-containing protein 4	55.14%	4	2	33	86	-0.066	0.088	-0.112	0.318	0.178	5																									584	64.8	5.73
IPI00672611.2	Kelch repeat and BTB domain-containing protein 11	55.13%	1	3	34	76																									-0.108	0.040	-0.006	-0.051	0.183	4	633	67.9	5.39
IPI00348328.4	keratin Kb40	98.50%	3	2	134	504							0.515	0.051	0.122	-0.035	0.127	6	0.143	0.093	-0.009	-0.082	-0.069	7							0.080	-0.327	-0.381	-0.371	-0.001	5	1068	112.2	7.97
IPI00227140.1	Keratin, type I cytoskeletal 14	82.64%	6	17	70	493	0.552	0.030	0.643	0.050	0.490	26	1.437	-0.012	1.422	0.084	1.370	46																			484	52.8	5.17
IPI00874585.1	Keratin, type I cytoskeletal 16	87.85%	4	3	59	166	0.190	-0.253	0.143	-0.006	0.019	3	1.478	0.183	1.443	0.349	1.641	9																			469	51.6	5.20
IPI00311493.5	Keratin, type I cytoskeletal 18	96.93%	1	36	72	1119	0.338	0.070	0.324	0.045	0.285	245	-0.781	-0.188	-0.626	-0.250	-0.529	25																			423	47.5	5.33
IPI00112947.1	Keratin, type I cytoskeletal 19	99.01%	4	28	61	663	1.174	0.037	1.038	0.001	1.170	131	0.141	-0.082	0.219	0.080	-0.003	31																			403	44.5	5.39
IPI00462986.2	Keratin, type II cuticular Hb3	93.54%	7	1	58	292							0.523	0.100	0.153	-0.025	0.148	5	0.143	0.093	-0.009	-0.082	-0.069	7							0.080	-0.327	-0.381	-0.371	-0.001	5	495	54.6	6.06
IPI00625729.2	Keratin, type II cytoskeletal 1	87.44%	1	1	82	315	0.384	0.080	0.484	0.061	0.306	4	0.585	-0.211	0.592	-0.006	0.605	7	0.729	-0.161	0.261	-0.080	0.088	6													637	65.6	8.15
IPI00462140.1	Keratin, type II cytoskeletal 1b	78.15%	1	3	72	363	0.846	0.207	0.935	0.080	0.762	20							0.841	-0.107	0.285	-0.523	0.026	3													572	61.3	8.02
IPI00622240.4	Keratin, type II cytoskeletal 2 epidermal	84.30%	2	1	69	241							0.681	-0.139	0.594	-0.006	0.605	5													0.057	0.154	0.138	0.094	0.131	6	707	70.9	8.06
IPI00346834.1	Keratin, type II cytoskeletal 2 oral	83.00%	4	2	75	376	0.751	0.238	0.758	0.112	0.685	6	0.552	0.003	0.246	-0.006	0.215	9	0.198	0.044	0.047	-0.160	-0.086	10							0.065	-0.019	0.003	-0.053	0.131	12	594	62.8	8.43
IPI00420312.4	Keratin, type II cytoskeletal 4	95.43%	1	1	85	318							0.543	0.003	0.225	-0.006	0.366	10	0.143	0.093	-0.009	-0.082	-0.069	7							0.075	0.072	0.017	-0.056	0.131	10	525	56.2	8.15
IPI00406377.3	Keratin, type II cytoskeletal 7	96.50%	4	13	69	545	1.122	0.083	1.187	0.023	0.965	47	0.385	-0.007	0.153	-0.009	0.169	18	0.219	0.134	0.066	-0.032	-0.023	19	-0.327	-0.016	-0.360	0.011	-0.375	11	0.076	-0.188	-0.163	-0.122	-0.001	9	457	50.7	5.87
IPI00468956.4	Keratin, type II cytoskeletal 71	81.30%	1	1	57	213	0.912	0.089	0.887	0.040	0.893	7	0.633	-0.175	0.593	0.011	0.590	6	0.841	-0.107	0.285	-0.523	0.026	3													524	57.3	6.99
IPI00420970.3	Keratin, type II cytoskeletal 74	85.25%	1	1	48	245	0.861	0.057	0.836	-0.006	0.834	8	0.633	-0.175	0.593	0.011	0.590	6	0.841	-0.107	0.285	-0.523	0.026	3							0.098	-0.317	-0.329	-0.326	-0.095	7	495	54.7	5.72
IPI00124499.2	Keratin, type II cytoskeletal 79	96.23%	1	1	82	312	1.043	0.310	1.069	0.081	0.949	13	0.534	0.003	0.204	-0.025	0.346	11	0.143	0.093	-0.009	-0.082	-0.069	7													531	57.5	7.69
IPI00322209.5	Keratin, type II cytoskeletal 8	95.51%	4	34	84	1527	0.641	0.070	0.643	0.052	0.619	228	0.572	-0.022	0.552	0.011	0.503	103	0.219	0.134	0.066	-0.032	-0.023	19	-0.327	-0.016	-0.360	0.011	-0.375	11	0.076	-0.188	-0.163	-0.122	-0.001	9	490	54.5	5.82
IPI00278366.4	Keratinocyte-associated protein 2	46.36%	1	1	12	43	-0.076	-0.039	-0.017	0.026	-0.125	4																									220	24.0	9.38
IPI00340836.1	Keratinocyte-associated transmembrane protein 2	52.12%	1	1	17	80	0.128	0.200	0.007	0.143	-0.136	3							-0.167	0.127	-0.279	-0.194	-0.448	5													259	28.0	5.07
IPI00458765.2	KH domain-containing, RNA-binding, signal transduction-associated protein	72.01%	3	11	47	444	0.047	0.070	0.052	0.041	0.073	18	0.029	-0.053	-0.083	-0.041	-0.162	18	0.219	0.074	0.217	0.013	0.194	23	0.051	0.151	0.102	0.044	-0.028	25	-0.089	0.126	0.040	0.122	0.036	9	443	48.3	8.72
IPI00314520.3	KH domain-containing, RNA-binding, signal transduction-associated protein	60.12%	1	2	40	187	0.167	0.277	0.011	0.073	-0.016	3							0.114	0.030	0.102	-0.146	0.014	5													346	38.8	8.10
IPI00760003.2	kinectin	88.77%	4	4	195	1193	-0.066	0.083	-0.023	0.070	-0.071	43	0.275	-0.032	0.234	0.013	0.181	55	0.236	0.046	0.238	-0.125	0.204	54	0.136	-0.029	0.198	-0.019	0.126	60							1327	152.4	5.90
IPI00407864.3	kinesin family member 23	81.64%	3	15	116	521	-0.105	0.120	-0.170	0.048	0.010	10	-0.009	-0.292	-0.016	-0.056	0.147	9	-0.144	-0.049	-0.009	-0.066	-0.094	12													953	108.7	8.66
IPI00109420.2	Kinesin heavy chain isoform 5A	84.13%	1	2	142	668	0.016	-0.281	-0.114	-0.134	-0.219	6	-0.039	-0.120	0.098	-0.039	-0.013	17	-0.116	-0.004	-0.500	-0.019	0.144	3	-0.128	-0.004	-0.059	0.043	-0.079	15	-0.048	-0.051	-0.050	0.073	-0.007	13	1027	116.9	5.94
IPI00421137.4	Kinesin heavy chain isoform 5C	88.39%	2	18	140	723	-0.037	-0.271	-0.167	-0.163	-0.265	15	-0.044	-0.146	-0.013	-0.004	-0.057	22	-0.122	-0.100	-0.573	-0.106	0.131	4	-0.292	-0.066	-0.085	0.034	-0.214	25	-0.048	-0.029	-0.010	-0.044	-0.031	57	956	109.2	6.19
IPI00753928.1	kinesin light chain 2	80.94%	4	2	77	293	0.175	-0.116	0.091	-0.103	0.149	7	0.218	0.138	0.264	0.069	0.016	4	-0.313	-0.255	-0.320	-0.013	-0.116	4	-0.226	0.139	-0.201	0.068	-0.265	7	0.128	0.059	-0.014	-0.001	-0.035	8	619	68.4	7.06
IPI00844651.1	Kinesin light chain 2	79.97%	3	1	75	281	0.019	-0.119	0.089	-0.099	0.116	6	-0.020	0.134	0.095	0.069	-0.112	4	-0.415	-0.255	-0.320	0.041	-0.116	3	-0.226	0.139	-0.201	0.068	-0.265	7	0.041	0.059	-0.014	-0.001	-0.035	7	599	66.6	7.21
IPI00120089.1	Kinesin light chain 4	82.55%	4	3	67	235	-0.065	-0.094	-0.117	0.009	0.110	4	0.081	0.114	0.229	0.004	-0.007	3							-0.062	0.172	-0.003	0.138	-0.031	4							619	68.6	6.09
IPI00124954.2	Kinesin-1 heavy chain	95.43%	2	41	158	1232	0.006	-0.045	0.047	-0.054	0.045	72	0.062	-0.028	0.152	-0.035	-0.013	78	-0.181	-0.124	-0.276	0.045	-0.053	43	-0.125	0.062	-0.051	0.076	-0.167	49	-0.085	0.014	-0.029	0.036	0.014	25	963	109.5	6.44
IPI00130218.2	Kinesin-like protein KIF11	92.87%	2	14	138	472	-0.111	0.052	-0.292	-0.017	-0.214	14	-0.112	-0.037	-0.213	-0.080	-0.012	9							0.406	0.077	0.596	0.095	0.621	12							1052	118.0	6.00
IPI00123947.1	Kinesin-like protein KIF20A	72.04%	1	9	83	270							-0.066	-0.023	-0.127	-0.041	0.127	9	0.466	0.173	0.625	-0.542	0.303	4													887	99.8	6.99
IPI00223570.2	KRR1 small subunit processome component homolog	81.58%	3	7	54	158	-0.097	-0.102	-0.398	-0.147	-0.255	6													0.031	0.148	0.115	0.081	0.202	8							380	43.5	9.79
IPI00321734.7	Lactoylglutathione lyase	89.67%	1	11	20	249	0.019	-0.057	0.183	0.006	0.198	13	-0.278	0.079	-0.154	0.016	-0.157	29	-0.353	-0.169	-0.311	0.190	-0.203	8	1.341	0.370	1.283	0.193	1.118	5	0.021	0.013	0.085	-0.060	-0.014	35	184	20.8	5.47
IPI00319558.1	Ladinin-1	89.39%	1	19	95	596	0.381	-0.019	0.345	-0.038	0.348	57																									528	58.8	9.73
IPI00331173.8	Lamin-B receptor	55.91%	1	17	45	338	-0.083	0.073	-0.019	0.017	-0.089	30	-0.028	0.108	0.013	-0.041	-0.111	16	0.087	0.034	0.069	0.000	0.030	17	0.219	-0.048	0.138	-0.010	0.197	20							626	71.4	9.36

IPI00230394.5	Lamin-B1	94.56%	1	33	90	1245	0.059	0.061	0.123	0.070	0.104	70	-0.057	0.070	-0.035	0.107	-0.228	83	0.280	0.098	0.209	-0.066	0.116	109	0.009	-0.083	0.033	-0.044	0.020	66	-0.086	0.118	0.204	0.103	0.070	54	588	66.7	5.16
IPI01027599.1	laminin subunit alpha-1 precursor	67.43%	2	3	237	792	0.491	0.217	0.156	-0.109	0.368	3																									3083	337.9	6.71
IPI00116913.4	Laminin subunit alpha-5	73.05%	2	16	266	995							-0.720	-0.042	-0.644	0.026	-0.617	15	-0.139	0.217	0.013	-0.111	-0.047	9													3718	403.8	6.73
IPI00119065.2	Laminin subunit beta-2	74.15%	1	19	144	567							0.045	0.170	0.020	0.167	-0.149	24													-0.111	-0.041	-0.037	-0.069	-0.057	6	1799	196.5	6.67
IPI01027808.1	laminin subunit gamma-1 precursor	71.50%	3	21	138	703							-0.265	0.016	-0.144	0.092	-0.333	30	-0.097	0.011	-0.128	-0.484	-0.335	9							-0.050	0.205	0.059	0.277	0.174	9	1607	177.1	5.19
IPI00894786.1	LanC (Bacterial lantibiotic synthetase component C)-like 1, isoform CRA_a	74.44%	3	5	26	96							-0.085	-0.077	0.147	0.104	-0.077	3							0.111	0.010	-0.181	0.099	-0.198	4	0.138	0.017	-0.105	-0.051	-0.038	12	399	45.3	7.77
IPI00121949.1	LanC-like protein 2	68.89%	2	8	35	151																									0.170	-0.160	-0.178	0.004	-0.123	23	450	50.7	7.28
IPI00458711.2	Lanosterol 14-alpha demethylase	83.30%	1	12	37	209	0.059	0.040	0.138	0.083	0.115	29	0.163	0.141	0.467	0.372	-0.123	9	0.441	0.277	0.315	0.171	0.215	7													503	56.7	8.41
IPI00169958.3	Lanosterol synthase	62.48%	1	7	43	134	-0.042	0.324	-0.162	0.050	-0.069	5	0.264	0.048	-0.034	0.300	-0.080	3	0.013	0.170	-0.054	-0.041	-0.366	4	0.095	-0.069	-0.001	0.036	0.052	6							733	83.1	6.40
IPI00828610.1	la-related protein 4 isoform 2	69.64%	3	10	60	304	-0.065	0.004	-0.106	-0.059	-0.131	22	-0.095	-0.097	-0.203	-0.074	-0.155	11	-0.050	0.159	-0.088	-0.131	-0.080	18	0.306	0.128	0.265	0.010	0.205	7							718	79.6	6.51
IPI00129395.2	Large neutral amino acids transporter small subunit 1	49.61%	2	6	22	210	0.229	0.072	0.111	0.036	0.084	8	0.572	0.126	0.505	0.192	0.553	18	0.050	0.041	0.111	-0.119	-0.089	8	-0.346	0.020	-0.525	0.023	-0.385	18	0.106	-0.155	-0.009	0.099	0.230	10	512	55.8	7.90
IPI00130381.1	Large proline-rich protein BAG6	56.93%	10	20	69	429	0.000	0.044	0.148	-0.020	0.009	22	-0.038	-0.050	-0.062	-0.142	-0.138	21	-0.079	-0.013	0.050	0.011	0.141	8	0.068	-0.007	0.011	-0.003	0.092	23	-0.206	-0.214	-0.138	-0.193	-0.139	4	1154	121.0	5.71
IPI00223875.1	L-asparaginase	75.15%	1	9	28	121																									-0.095	-0.048	0.043	-0.001	-0.079	36	326	33.9	7.65
IPI00331745.2	Latexin	50.45%	1	4	9	62							-1.111	-0.257	-0.554	0.014	-0.181	6													-0.196	0.154	0.340	-0.122	0.158	7	222	25.5	5.74
IPI00349285.2	LDLR chaperone MESD	70.54%	2	5	20	118	-0.114	-0.016	-0.045	0.092	0.041	5	0.314	0.009	0.282	0.194	0.208	15	0.180	0.017	-0.010	-0.035	-0.059	5													224	25.2	6.34
IPI00130627.1	Legumain	66.21%	1	6	28	63																			-0.274	0.151	-0.165	0.003	-0.257	4							435	49.3	6.39
IPI00465876.1	LEM domain-containing protein 2	56.36%	1	3	36	81							-0.170	0.181	-0.003	-0.322	-0.133	4																			511	57.5	9.01
IPI00380354.1	lethal(2) giant larvae protein homolog 1 isoform 1	49.15%	3	2	44	106																									0.095	0.084	0.250	0.169	0.190	3	1062	115.5	6.39
IPI00131177.1	LETM1 and EF-hand domain-containing protein 1, mitochondrial	86.04%	1	18	87	447	0.043	0.195	0.023	0.057	0.176	5	0.282	0.148	0.296	0.285	0.019	7	0.149	0.072	-0.105	-0.004	0.024	6	0.253	-0.085	0.238	-0.003	0.073	17	0.000	-0.063	-0.022	-0.178	-0.019	24	738	82.9	6.52
IPI00322204.4	Leucine zipper protein 1	87.08%	3	14	130	448	0.010	-0.016	0.134	-0.120	0.081	6	-0.290	-0.162	-0.209	-0.147	-0.072	14	0.057	0.110	-0.038	0.233	-0.020	7													1068	119.2	7.99
IPI00120302.1	Leucine-rich glioma-inactivated protein 1	55.12%	2	9	47	140																									-0.105	-0.133	-0.114	-0.128	-0.047	25	557	63.6	8.02
IPI00420706.4	Leucine-rich PPR motif-containing protein, mitochondrial	86.06%	3	60	172	1264	-0.181	0.055	-0.171	0.001	-0.184	73	-0.077	0.094	-0.086	0.069	-0.069	43	0.051	0.059	-0.041	-0.072	-0.077	81	-0.122	0.038	-0.095	0.026	-0.163	60	-0.003	-0.054	-0.057	-0.173	-0.086	30	1392	156.5	6.83
IPI00225459.1	Leucine-rich repeat and WD repeat-containing protein 1	79.01%	1	2	39	93																			-0.160	0.084	-0.068	-0.240	0.000	3							648	71.5	7.87
IPI01008615.1	leucine-rich repeat flightless-interacting protein 2 isoform 1	80.48%	4	3	52	165	0.079	0.136	0.264	0.119	-0.025	4	-0.091	0.044	-0.013	0.014	-0.076	4	-0.103	0.000	-0.246	-0.175	-0.411	4													415	47.1	5.68
IPI00170121.1	Leucine-rich repeat LGI family member 3	46.35%	1	3	29	84																									-0.228	-0.119	0.167	0.208	0.024	5	548	61.8	8.16
IPI00131754.2	Leucine-rich repeat protein SHOC-2	82.30%	1	5	57	188							-0.262	0.313	0.229	0.181	-0.184	3																			582	64.9	8.46
IPI00470138.4	Leucine-rich repeat-containing protein 40	87.04%	4	8	71	256							0.001	0.233	-0.180	0.185	-0.209	6							0.122	-0.007	0.130	-0.071	0.077	8							602	68.0	6.89
IPI00929831.1	leucine-rich repeat-containing protein 57 isoform a	90.75%	4	2	40	140													-0.154	-0.021	-0.336	-0.088	-0.065	3							0.052	-0.028	-0.054	0.023	-0.062	13	281	31.4	9.14
IPI00123281.1	Leucine-rich repeat-containing protein 59	90.23%	2	18	44	658	0.120	0.098	0.088	0.036	-0.051	51	0.584	0.043	0.445	0.106	0.348	48	0.244	0.105	0.180	-0.057	0.080	62	-0.423	-0.017	-0.404	-0.035	-0.379	52	-0.175	-0.042	-0.091	-0.158	-0.154	20	307	34.9	9.52
IPI00223987.1	Leucyl-cystinyl aminopeptidase	80.39%	2	11	94	395	-0.191	0.037	-0.130	-0.286	-0.452	5	-0.181	-0.016	-0.035	0.224	-0.226	19	0.183	0.132	-0.013	0.084	-0.059	8													1025	117.2	5.96
IPI00457659.2	Leukocyte elastase inhibitor A	84.96%	4	10	44	181													-0.272	-0.221	-0.149	0.091	-0.180	3							-0.004	0.297	0.279	0.308	0.267	26	379	42.5	6.21
IPI00229527.8	Leukotriene A-4 hydrolase	74.80%	1	23	58	460	0.041	-0.128	0.009	-0.068	0.066	35	0.154	-0.105	0.132	-0.068	0.420	12	-0.171	-0.260	-0.318	0.123	-0.082	21	0.088	0.021	-0.025	0.031	-0.097	22	-0.007	0.037	-0.012	0.102	0.118	47	611	69.0	6.42
IPI00153364.1	Leydig cell tumor 10 kDa protein homolog	87.23%	1	4	18	48							-0.053	0.064	-0.131	0.200	-0.147	9																			94	10.2	11.63
IPI00125091.1	LIM and SH3 domain protein 1	93.54%	12	16	32	617	0.161	-0.097	0.235	-0.031	0.138	43	-0.336	-0.102	-0.320	-0.191	-0.245	17	-0.138	-0.095	-0.108	0.076	0.091	21	-0.116	-0.089	-0.253	-0.014	-0.186	16	-0.032	0.153	0.200	0.222	0.161	27	263	30.0	7.05
IPI00659447.3	LIM domain only 7	80.22%	5	1	184	775	-0.139	0.027	-0.157	0.004	-0.170	74	0.088	0.084	0.162	0.057	0.126	19	0.315	0.113	0.204	0.016	0.154	21													1699	192.4	6.25
IPI00134881.2	LIM domain-containing protein 1	64.67%	1	5	40	141	0.355	-0.038	0.197	-0.068	0.227	3																									668	71.4	6.32
IPI00222833.1	Limbic system-associated membrane protein	82.70%	3	10	31	149																									0.009	0.099	0.052	0.131	0.092	32	341	38.1	6.65
IPI00310701.4	Lipase maturation factor 2	50.43%	1	8	33	117	-0.141	0.091	-0.119	0.036	-0.054	3	0.163	-0.183	0.318	0.296	0.386	5	0.107	0.067	0.037	0.207	-0.004	16													702	79.9	9.99
IPI00115626.1	Lipid phosphate phosphohydrolase 3	49.68%	1	5	21	79																									-0.050	0.112	0.181	0.160	0.166	12	312	35.2	9.07
IPI00555143.5	liprin-alpha-2	79.94%	5	2	139	473																									0.070	-0.007	0.034	-0.057	-0.109	3	1256	143.2	6.11
IPI00399905.5	Liprin-alpha-3	68.84%	1	8	101	349																									0.019	-0.186	-0.100	-0.059	-0.448	15	1043	116.2	5.96
IPI00751369.1	L-lactate dehydrogenase A chain isoform 2	87.53%	8	24	54	1172	-0.072	-0.120	-0.010	-0.007	0.021	97	-0.498	0.112	-0.454	0.004	-0.454	74	-0.366	-0.199	-0.300	0.144	-0.083	89	0.367	0.174	0.352	0.058	0.395	63	0.014	-0.342	-0.353	-0.364	-0.342	96	361	39.7	8.35
IPI00229510.5	L-lactate dehydrogenase B chain	82.63%	3	22	48	511	-0.139	-0.139	-0.196	-0.065	-0.149	7	-0.490	0.068	-0.571	-0.009	-0.381	7	-0.599	-0.224	-0.375	-0.034	-0.223	10							-0.003	0.123	0.175	0.102	0.065	156	334	36.5	6.05
IPI00319518.4	Lon protease homolog	89.09%	2	35	115	1011	-0.089	0.045	-0.109	-0.060	-0.138	55	-0.278	-0.023	-0.269	0.098	-0.444	35	0.030	0.106	-0.039	0.061	-0.116	19	0.010	-0.019	0.006	-0.044	0.020	119	0.009	0.076	-0.014	0.007	0.038	16	953	106.4	6.71
IPI00119114.2	Long-chain specific acyl-CoA dehydrogenase, mitochondrial	80.23%	2	17	53	353	0.099	0.044	0.185	0.052	0.070	31	-0.188	-0.035	-0.125	-0.057	-0.141	21	-0.069	0.092	-0.138	-0.041	-0.240	23							-0.099	0.098	0.181	0.069	0.102	28	430	47.9	8.31
IPI00169772.3	Long-chain-fatty-acid--CoA ligase 3	80.42%	3	12	60	375	-0.013	0.102	-0.051	0.077	-0.016	30	0.095	-0.004	0.375	0.374	-0.031	14	0.416	0.104	0.320	-0.039	0.157	5	0.054	-0.136	0.011	-0.045	-0.076	4							720	80.4	8.54
IPI00170363.1	Long-chain-fatty-acid--CoA ligase 5	69.55%	3	8	55	176	-0.032	-0.048	0.014	0.052	0.013	6	0.305	0.395	-0.048	0.343	0.549	5							-0.224	0.092	-0.178	-0.034	-0.139	3							683	76.2	7.09
IPI00453834.2	Long-chain-fatty-acid--CoA ligase ACSBG1	72.54%	3	16	63	194																									-0.051	0.033	0.123	0.158	0.156	27	721	80.4	5.94
IPI00556850.1	low molecular weight phosphotyrosine protein phosphatase isoform 1	76.58%	2	1	18	69	-0.198	-0.069	0.021	-0.082	0.020	6	-0.390	-0.131	-0.281	-0.147	-0.028	9													0.360	0.250	0.043	0.081	0.116	5	158	17.9	6.54
IPI00849664.3	LOW QUALITY PROTEIN: dynein heavy chain 7, axonemal	76.13%	4	1	318	935	0.000	0.076	-0.105	-0.063	-0.091	3																									4068	465.3	6.20
IPI00889205.3	LOW QUALITY PROTEIN: RING finger protein 213	72.64%	2	10	435	1373	-0.160	0.073	0.007	-0.009	-0.264	6													-0.163	0.007	-0.138	0.004	-0.102	3							5223	592.1	6.57
IPI00785217.1	low-density lipoprotein receptor	79.12%	3	6	58	183	0.072	0.185	0.116	-0.255	0.020	4	0.100	-0.191	0.141	-0.053	0.210	7	-0.355	-0.212	-0.633	-0.878	-0.478	3													862	94.9	5.02
IPI00855165.1	Lrrfip1 protein	83.56%	3	1	90	295	0.007	0.132	0.040	0.027	-0.041	11	-0.054	0.001	-0.013	0.014	-0.071	9	-0.373	-0.049	-0.326	-0.175	-0.255	10													663	75.1	5.85
IPI00134300.1	Lupus La protein homolog	83.61%	5	23	69	633	-0.114	0.029	-0.170	0.029	-0.167	42	-0.109	0.007	-0.041	0.033	-0.123	44	0.007	0.037	0.123	0.037	0.091	44	0.084	0.009	0.193	0.064	0.087	33	-0.128	0.006	0.127	0.041	-0.077	8	415	47.7	9.77
IPI00129253.3	Lymphocyte antigen 75	58.27%	2	17	96	291							-0.276	0.069	-0.349	-0.014	-0.442	34	0.519	-0.075	0.435	0.112	0.009	3													1723	197.2	6.39
IPI00467350.2	Lymphokine-activated killer T-cell-originated protein kinase	48.18%	1	4	18	36																			0.743	0.017	0.881	0.087	0.721	3							330	36.7	5.12
IPI00453837.2	Lysine-specific histone demethylase 1A	62.72%	5	11	68	252	-0.054	-0.017	-0.150	0.045	-0.028	5	-0.042	-0.255	0.110	-0.066	0.066	5							-0.257	-0.089	-0.199	-0.076	-0.038	10							853	92.8	6.52
IPI00380082.1	Lysophospholipid acyltransferase 5	43.33%	2	2	24	63	0.112	-0.010	-0.019	-0.213	0.052	6																									487	56.1	8.56
IPI00319646.3	Lysophospholipid acyltransferase 7	33.83%	2	3	21	52	-0.053	-0.035	0.065	-0.001	-0.111	5																									473	53.4	8.69
IPI00344734.3	Lysophospholipid acyltransferase LPCAT4	68.13%	1	2	32	66																									-0.204	0.396	0.126	-0.219	-0.028	3	524	57.1	8.75
IPI00111960.2	Lysosomal alpha-glucosidase	62.43%	5	13	60	371	-0.315	0.157	-0.023	-0.031	-0.245	4	-0.407	-0.324	-0.366	0.040	-0.633	5							-0.664	0.001	-0.478	-0.053	-0.513	34	0.085	0.138	-0.131	-0.216	0.127	6	953	106.2	5.83
IPI00381303.6	Lysosomal alpha-mannosidase	64.96%	1	4	53	140							0.136	0.065	0.496	0.305	0.439	8																			1013	114.6	8.13
IPI00658539.1	lysosomal protective protein isoform a	64.43%	2	3	32	139	0.259	0.179	0.224	0.085	0.259	4	-1.041	0.021	-1.056	0.003	-0.673	6																			492	55.7	5.87
IPI00122648.2	lysosomal-associated transmembrane protein 4A	88.96%	2	1	20	80																			1.527	-0.245	1.144	-0.004	1.961	8							308	35.0	8.50
IPI00127447.3	Lysosome membrane protein 2	64.02%	1	6	33	188	-0.086	0.094	-0.014	0.084	-0.048	8	-0.165	-0.022	0.051	-0.218	-0.214	6	0.241	0.151	0.099	0.031	-0.088	11	0.262	-0.028	0.310	-0.035	0.274	7							478	54.0	5.10
IPI00469103.1	lysyl-tRNA synthetase isoform 1	88.14%	3	24	65	957	0.080	0.099	0.054	0.051	0.040	45	-0.139	-0.031	-0.136	0.059	-0.199	38	-0.250	-0.117	-0.243	0.001	-0.105	24	0.239	0.075	0.283	0.016	0.197	84	-0.062	-0.056	-0.083	0.044	-0.099	14	624	71.2	6.43
IPI00122740.2	MACRO domain-containing protein 1	80.19%	1	2	25	50																									0.302	0.297	0.393	0.360	0.070	3	323	35.3	8.85
IPI00230427.5	Macrophage migration inhibitory factor	52.17%	1	5	9	112	-0.191	-0.077	-0.438	-0.150	-0.037	11							-0.057	-0.064	-0.183	0.178	-0.069	3	0.281	0.011	0.397	0.045	0.511	3	0.241	-0.079	-0.095	-0.167	-0.031	12	115	12.5	7.34
**IPI00120800.2**	**mage-k1**	**72.50%**	**5**	**10**	**26**	**159**																			-0.342	0.033	-0.510	0.009	-0.349	**42**							**320**	**35.0**	**5.14**
IPI00120793.1	Major prion protein	53.94%	1	5	11	44																									1.834	0.270	2.022	-0.116	1.873	5	254	28.0	9.33
IPI00111258.4	major vault protein	85.63%	2	23	83	429	0.274	-0.056	0.275	0.009	0.270	53	0.089	0.027	0.020	0.073	0.043	32	0.087	-0.045	-0.221	0.114	0.088	17													870	96.8	5.64
IPI00336324.11	Malate dehydrogenase, cytoplasmic	96.11%	2	21	47	609	0.006	-0.097	0.047	-0.023	0.145	20	0.020	-0.007	0.123	-0.133	-0.003	13	-0.320	-0.179	-0.209	0.057	-0.094	14	0.184	-0.031	0.197	0.007	0.253	26	0.023	0.044	0.037	-0.013	0.004	114	334	36.5	6.58
IPI00323592.2	Malate dehydrogenase, mitochondrial	96.15%	1	20	45	1003	0.001	0.023	-0.071	-0.001	-0.026	76	-0.034	-0.009	0.026	0.030	-0.009	65	0.308	0.108	0.323	-0.014	0.174	89	0.059	-0.089	0.084	-0.053	0.114	47	0.212	-0.003	0.006	-0.112	-0.001	114	338	35.6	8.68
IPI00126120.1	Maleylacetoacetate isomerase	90.74%	1	6	20	119																			-0.320	-0.102	-0.482	-0.001	-0.065	12	-0.097	0.029	-0.252	-0.016	0.011	9	216	24.3	7.85
IPI00342938.1	Malonyl-CoA-acyl carrier protein transacylase, mitochondrial	79.00%	2	4	32	114	-0.154	0.070	-0.051	0.084	-0.281	4																									381	41.9	8.10
IPI00125807.3	Mannose-6-phosphate isomerase	66.19%	2	7	29	92																									0.281	0.079	0.010	0.013	-0.023	15	423	46.5	5.95
IPI00330323.1	Mannosyl-oligosaccharide glucosidase	46.88%	1	5	34	129	-0.026	0.050	0.052	-0.091	0.068	9	-0.060	0.124	0.084	0.047	0.154	5							-0.097	0.064	-0.152	-0.029	-0.088	5							834	91.8	9.00
IPI00928064.2	MAP/microtubule affinity-regulating kinase	73.37%	1	2	73	179																									0.122	-0.123	-0.080	-0.044	0.076	10	796	88.3	9.36
IPI00816921.2	MAP6 domain-containing protein 1	79.06%	1	1	14	38																									0.092	0.016	0.403	0.258	0.161	4	191	20.4	9.88
IPI00281011.7	MARCKS-related protein	99.00%	1	8	23	211	-0.340	-0.001	-0.375	-0.047	-0.257	4	-1.083	-0.219	-0.842	-0.160	-1.020	16	0.188	0.146	0.179	0.033	0.057	11	-0.152	-0.088	-0.250	0.034	-0.144	17	-0.050	0.256	0.338	0.031	0.183	7	200	20.2	4.61
IPI00754025.1	mast/stem cell growth factor receptor isoform 1	69.77%	9	1	78	259													-0.171	-0.258	-0.957	-0.884	-0.464	3							-0.199	-0.116	-0.155	-0.128	-0.022	4	979	109.3	7.21
IPI00323045.4	Maternal embryonic leucine zipper kinase	62.99%	1	2	52	113	-0.074	0.069	-0.390	-0.086	-0.160	3																									643	72.7	8.72
IPI00453826.2	Matrin-3	78.25%	2	39	90	1219	-0.025	-0.038	0.011	0.019	-0.041	52	-0.142	-0.029	-0.184	0.064	-0.170	85	0.166	0.073	0.127	0.058	0.048	55	-0.196	-0.045	-0.122	-0.026	-0.095	85	-0.014	0.079	0.140	0.110	0.127	66	846	94.6	6.25
IPI00226263.5	Matrix-remodeling-associated protein 7	55.06%	2	5	7	45							-0.089	0.348	0.335	0.076	0.416	4	0.081	0.025	0.026	-0.338	-0.103	9													178	19.4	4.26
IPI00111353.3	Mediator of RNA polymerase II transcription subunit 11	78.63%	2	1	12	57							0.122	0.141	-0.295	0.119	0.068	3																			117	13.1	5.96
IPI00125670.1	Mediator of RNA polymerase II transcription subunit 15	39.65%	5	2	30	75							0.323	0.139	0.048	-0.163	0.029	4																			792	87.0	9.42
IPI00459787.2	Mediator of RNA polymerase II transcription subunit 17	63.33%	1	1	54	167							-0.100	-0.434	0.487	-0.175	-0.125	3																			649	72.4	7.30
IPI00134961.1	Medium-chain specific acyl-CoA dehydrogenase, mitochondrial	72.45%	2	14	40	241	-0.007	-0.014	-0.170	-0.050	-0.020	10	-0.413	0.033	-0.231	0.287	-0.448	19	0.103	0.151	-0.009	-0.102	-0.076	14													421	46.5	8.37
IPI00136178.1	Melanoma-associated antigen D1	55.48%	2	5	45	221																			0.089	-0.250	0.007	-0.089	0.169	4							775	85.6	7.50
IPI00169845.2	Membrane magnesium transporter 1	99.24%	1	2	13	53	0.052	0.115	-0.059	0.036	-0.105	5	0.398	0.026	0.134	-0.114	0.225	3	0.240	0.114	0.402	0.126	0.194	4													131	14.7	9.16
IPI00351206.5	Membrane-associated progesterone receptor component 2	98.16%	1	5	26	154	-0.243	-0.082	-0.068	-0.057	-0.144	6	0.019	0.152	-0.117	-0.388	-0.158	4	0.346	0.150	0.087	-0.189	-0.012	14	-0.211	-0.117	-0.216	-0.108	-0.193	11							217	23.3	5.15
IPI00121279.1	Mesothelin	80.00%	1	9	51	237	0.763	0.062	0.750	0.094	0.659	16	0.584	0.069	0.748	0.027	0.516	22																			625	69.4	7.59
IPI00762862.3	Metabotropic glutamate receptor 2	52.87%	1	1	52	147																									0.044	-0.106	0.077	0.048	0.061	9	872	95.8	8.06
IPI00136716.1	Metabotropic glutamate receptor 3	69.06%	2	5	67	241																									-0.119	-0.034	0.100	0.069	-0.001	11	879	99.0	7.75
IPI00471150.3	Metalloreductase STEAP1	62.83%	2	2	21	106	-0.134	0.202	-0.109	0.061	-0.208	4	-0.226	0.069	-0.279	0.080	-0.311	6																			339	39.3	9.19
IPI00128230.1	Metastasis-associated protein MTA2	82.78%	1	18	91	523	0.158	0.051	0.103	0.016	0.100	26	-0.076	-0.042	-0.022	-0.007	-0.117	40	0.062	-0.005	-0.048	-0.094	0.031	25	0.026	-0.088	0.061	-0.144	0.065	13							668	75.0	9.67
IPI00112327.2	metaxin-1 isoform 1	83.73%	8	10	55	326	0.227	0.192	0.089	0.145	-0.109	11							0.277	0.115	0.084	0.021	0.132	12							-0.007	-0.381	0.083	0.127	-0.060	8	461	51.3	9.60
IPI00225254.6	Metaxin-2	77.95%	1	7	16	121							-0.032	0.023	-0.056	0.085	0.016	8	0.256	0.061	0.158	-0.072	0.001	8							0.019	0.036	-0.077	-0.010	-0.004	23	263	29.7	5.63
IPI00272238.5	Methionine aminopeptidase	82.99%	1	13	44	259	-0.050	-0.160	-0.175	-0.157	0.019	14	0.102	0.020	-0.042	-0.158	0.112	14	-0.149	-0.053	-0.025	-0.059	-0.094	13	0.286	-0.048	0.107	0.064	0.187	21							488	54.1	5.81
IPI00272622.1	Methionine aminopeptidase 1	90.93%	1	9	29	86							-0.535	0.068	-0.492	-0.099	-0.615	17																			386	43.2	7.17
IPI00225312.2	Methionine-R-sulfoxide reductase B3, mitochondrial	70.75%	2	1	21	89							-0.130	-0.053	-0.400	-0.139	-0.255	4																			253	26.8	7.99
IPI00461469.3	Methionyl-tRNA synthetase, cytoplasmic	78.27%	3	26	86	677	0.072	0.014	0.044	-0.032	0.020	49	0.242	-0.112	0.224	-0.290	0.295	23	0.024	-0.125	-0.022	-0.031	0.140	22	-0.204	0.048	-0.260	-0.012	-0.219	37	0.210	-0.150	-0.221	0.048	-0.589	4	902	101.4	7.14
IPI00553717.6	Methylcrotonoyl-CoA carboxylase beta chain, mitochondrial	77.44%	3	4	39	89																									0.087	-0.112	0.165	0.348	0.175	3	563	61.3	8.00
IPI00461964.3	Methylmalonate-semialdehyde dehydrogenase [acylating], mitochondria	89.53%	2	17	55	172																									-0.059	0.085	0.130	0.065	0.126	33	535	57.9	8.07
IPI00133553.2	Methylmalonyl-CoA mutase, mitochondrial	78.34%	1	9	63	275	-0.025	-0.233	-0.221	-0.105	-0.109	3																			-0.229	-0.039	-0.170	-0.016	-0.130	9	748	82.8	6.89
IPI00885509.1	Methylthioribose-1-phosphate isomerase	84.55%	3	9	35	131	-0.062	0.001	-0.133	-0.042	-0.072	4	0.007	0.174	0.154	0.236	0.145	10																			369	39.4	5.91
IPI00556810.1	MHC class I like protein GS10	78.25%	42	1	30	122	0.271	0.185	0.240	0.197	0.157	4	-0.136	0.029	-0.038	0.100	-0.072	19													0.050	-0.072	0.354	-0.157	0.100	3	354	39.6	6.62
IPI00228590.2	Microfibrillar-associated protein 1	80.87%	1	7	43	233	0.041	-0.079	-0.091	-0.083	0.019	12	0.057	0.273	0.333	0.061	-0.083	9							0.047	-0.022	0.158	0.013	-0.042	5							439	51.9	4.98
IPI00884482.4	microtubule-actin cross-linking factor 1 isoform 1	76.48%	14	67	780	2875	-0.013	-0.044	-0.034	-0.066	0.037	23	0.021	-0.047	-0.026	-0.086	-0.123	39	-0.109	0.009	-0.026	-0.038	-0.060	21							0.024	-0.020	-0.006	0.094	-0.042	31	7355	831.4	5.41
IPI00473477.2	Microtubule-associated protein	84.66%	2	2	173	1183	0.161	0.026	0.189	0.014	0.187	53	0.612	0.112	0.628	0.079	0.445	51	-0.183	-0.126	-0.209	0.013	0.016	49	-0.788	0.114	-0.776	0.070	-0.655	18	-0.133	-0.082	0.014	-0.035	-0.065	25	1441	152.7	9.28
IPI00895965.1	Microtubule-associated protein	90.97%	20	83	207	1274	0.370	-0.080	0.538	-0.010	0.227	3													-0.189	-0.020	0.017	-0.083	-0.276	7	-0.028	0.001	-0.105	-0.057	-0.072	327	1828	198.8	4.88
IPI00986743.1	Microtubule-associated protein	85.32%	4	6	117	823	0.172	0.010	0.180	-0.007	0.188	42	0.585	0.116	0.623	0.079	0.432	34	-0.167	-0.115	-0.204	0.024	0.014	44	-0.492	-0.039	-0.559	-0.062	-0.550	4	-0.139	-0.060	0.021	-0.029	-0.080	38	933	97.7	9.09
IPI00676243.2	microtubule-associated protein 1A isoform 1	61.98%	2	90	217	1145																			-1.006	0.128	-1.162	0.128	-1.026	7	0.037	0.148	0.153	0.104	0.134	291	3014	325.7	5.01
IPI00130920.2	Microtubule-associated protein 1B	75.77%	3	113	232	3401	0.322	0.062	0.123	-0.094	0.296	18	0.754	0.096	0.430	-0.136	0.438	9	0.119	-0.061	0.259	0.029	0.324	64	-0.639	0.045	-0.727	0.045	-0.630	601	0.024	0.044	-0.054	-0.085	-0.032	227	2464	270.1	4.83
IPI00223621.2	Microtubule-associated protein 1S	63.72%	1	8	64	192							0.100	-0.141	-0.035	0.106	-0.191	12							0.041	0.044	0.213	0.108	0.123	8							973	102.9	7.02
IPI00117896.3	Microtubule-associated protein RP/EB family member 1	94.78%	1	13	32	464	-0.037	-0.035	0.036	0.024	-0.059	39	-0.826	-0.076	-0.788	-0.091	-0.881	36	-0.204	-0.108	-0.117	0.189	-0.127	33	0.069	0.120	0.031	0.124	-0.047	11	0.111	0.066	0.069	-0.060	0.051	14	268	30.0	5.22
IPI00343557.1	Microtubule-associated protein RP/EB family member 3	67.26%	3	12	31	178	0.066	0.128	0.127	0.131	-0.122	5	-0.739	-0.071	-0.375	-0.077	-0.344	3	-0.381	-0.233	-0.211	-0.360	-0.189	3							-0.053	0.024	-0.031	-0.060	0.033	23	281	31.9	5.54
IPI00137594.2	Microtubule-associated proteins 1A/1B light chain 3A	34.71%	1	2	6	42																									-0.017	-0.051	0.118	-0.006	-0.119	8	121	14.3	8.68
IPI00230349.9	Microtubule-associated proteins 1A/1B light chain 3B	74.40%	2	2	10	69																			0.124	0.138	-0.123	0.080	-0.116	7	-0.157	-0.234	-0.050	-0.146	-0.357	6	125	14.6	8.43
IPI00458039.3	Midasin	72.57%	2	15	454	1583	-0.233	-0.201	-0.178	0.036	-0.141	3	0.296	0.027	0.727	0.535	0.729	5	-0.381	0.016	-0.258	-0.105	-0.147	3	-0.103	-0.048	-0.204	0.075	-0.189	16							5589	629.9	5.72
IPI00330551.2	Mimitin, mitochondrial	76.79%	1	5	17	94							0.027	-0.184	-0.125	-0.154	-0.344	5	0.166	-0.105	0.072	-0.216	-0.130	6							-0.228	-0.155	-0.163	-0.111	0.103	7	168	19.6	8.25
IPI00153724.1	Mini-chromosome maintenance complex-binding protein	77.41%	2	9	48	301	-0.080	-0.053	0.000	0.064	0.058	12	-0.003	0.122	0.011	0.084	0.033	14																			642	72.8	5.66
IPI00230754.5	Mitochondrial 2-oxoglutarate/malate carrier protein	93.63%	3	14	46	310	-0.097	0.057	-0.034	0.009	-0.063	11							0.240	0.024	0.287	0.048	0.027	10	-0.196	0.088	-0.085	0.034	-0.233	7	-0.004	-0.010	-0.059	-0.125	-0.100	31	314	34.1	9.94
IPI00122075.1	Mitochondrial antiviral-signaling protein	84.29%	2	8	30	296	0.062	0.009	0.006	0.084	0.034	8	-0.091	-0.353	-0.322	-0.158	-0.292	3	0.478	0.267	0.305	-0.390	0.172	7													503	53.4	6.37
IPI00132039.2	Mitochondrial carrier homolog 2	77.23%	4	8	27	264	-0.262	-0.047	-0.183	-0.019	-0.415	9	0.777	0.152	0.819	0.458	0.606	3	0.031	0.134	0.006	-0.017	-0.198	5	0.294	-0.026	0.156	0.116	0.128	13	-0.089	-0.213	-0.136	-0.167	-0.028	20	303	33.5	8.25
IPI00317074.3	Mitochondrial dicarboxylate carrier	95.12%	1	6	35	165	0.010	-0.206	-0.076	-0.082	0.123	5	0.052	-0.176	0.043	-0.063	-0.062	9	0.345	0.059	0.116	0.026	-0.041	9	0.569	0.153	0.518	-0.173	0.448	8							287	31.7	9.32
IPI00132217.1	Mitochondrial fission 1 protein	91.45%	3	5	18	185	0.358	0.043	0.185	0.050	0.316	14	0.029	-0.069	0.236	-0.120	0.372	12	0.285	0.015	0.244	-0.023	0.116	14	0.325	-0.025	0.269	-0.007	0.268	7	-0.054	-0.004	0.024	-0.003	0.055	14	152	17.0	8.53
IPI00315908.1	Mitochondrial fission process protein 1	43.37%	1	1	6	11																									0.163	0.353	-0.136	-0.109	0.010	3	166	18.3	8.68
IPI00109275.1	Mitochondrial glutamate carrier 1	97.83%	5	14	38	273																									0.126	-0.317	-0.388	-0.394	-0.329	69	323	34.6	9.09
IPI00165902.1	Mitochondrial import inner membrane translocase subunit Tim10	80.00%	1	5	11	42																									-0.001	-0.105	-0.198	0.054	-0.007	9	90	10.3	6.29
IPI00134484.1	Mitochondrial import inner membrane translocase subunit Tim13	87.37%	1	4	12	141	0.010	0.036	-0.006	-0.031	-0.019	14	0.089	0.077	-0.105	-0.012	0.019	12	0.020	0.075	-0.016	-0.019	-0.031	9	-0.026	0.068	-0.003	0.019	0.057	11	-0.019	0.041	0.076	0.031	0.094	15	95	10.5	8.18
IPI00133167.1	Mitochondrial import inner membrane translocase subunit TIM16	83.20%	1	3	16	194	-0.071	-0.014	-0.228	-0.016	-0.383	8	-0.203	0.119	-0.816	0.141	-0.234	4	1.613	0.270	0.054	-0.114	1.009	3	-0.089	-0.013	-0.199	-0.048	-0.102	9							125	13.8	9.64
IPI00129506.1	Mitochondrial import inner membrane translocase subunit Tim17-A	91.23%	2	2	10	148	0.020	-0.123	0.038	0.134	-0.163	10																									171	18.1	7.90
IPI00129504.1	Mitochondrial import inner membrane translocase subunit Tim17-B	90.70%	2	2	15	62							0.030	0.315	0.185	-0.103	0.641	3																			172	18.3	9.03
IPI00849271.1	mitochondrial import inner membrane translocase subunit Tim23	100.00%	3	4	22	109	-0.326	0.131	-0.311	-0.026	-0.258	8													0.045	-0.095	0.114	-0.020	0.166	8							209	21.9	8.60
IPI00135068.2	Mitochondrial import inner membrane translocase subunit TIM44	85.40%	2	15	62	295	0.047	0.011	0.044	0.075	-0.004	8							0.073	0.037	-0.109	-0.014	-0.038	21	-0.007	-0.039	0.014	-0.056	-0.042	21	-0.421	-0.102	-0.183	-0.029	-0.138	10	452	51.1	8.13
IPI00111045.1	Mitochondrial import inner membrane translocase subunit TIM50	76.49%	1	8	38	229	-0.009	0.072	-0.086	0.054	-0.068	9	0.122	0.003	0.091	0.045	0.115	21	0.267	0.126	0.172	-0.054	-0.072	10	0.119	0.004	0.152	-0.022	0.111	12	0.153	0.070	0.205	0.395	0.376	9	353	39.8	8.13
IPI00125776.1	Mitochondrial import inner membrane translocase subunit Tim8 A	97.94%	2	4	11	36	-0.009	0.195	-0.331	-0.191	-0.165	3	0.269	0.070	0.132	-0.048	0.095	6							-0.476	-0.258	-0.304	-0.142	-0.398	4							97	11.0	5.16
IPI00132169.1	Mitochondrial import inner membrane translocase subunit Tim8 B	48.19%	1	1	6	23	-0.454	-0.119	-0.425	-0.048	-0.550	6																									83	9.3	5.12
IPI00125513.1	Mitochondrial import inner membrane translocase subunit Tim9	100.00%	1	3	13	61													0.285	0.030	-0.014	-0.041	-0.111	7							0.135	-0.318	-0.274	-0.173	-0.100	5	89	10.3	7.21
IPI00315135.3	Mitochondrial import receptor subunit TOM22 homolog	84.51%	1	4	12	251	-0.010	0.000	-0.125	0.016	-0.059	16	-0.044	-0.109	-0.233	-0.209	-0.069	12	0.193	0.085	-0.053	0.011	-0.150	17	0.411	-0.007	0.376	0.013	0.323	22	0.030	0.001	-0.117	0.075	-0.054	9	142	15.5	4.34
IPI00474157.4	Mitochondrial import receptor subunit TOM40 homolog	86.70%	2	7	24	547	-0.201	0.014	-0.219	-0.007	-0.285	40	0.108	-0.009	-0.029	0.273	0.123	13	0.010	0.026	-0.012	-0.089	0.014	15	0.111	-0.086	0.176	0.027	0.251	30	-0.023	-0.039	-0.025	-0.134	0.037	9	361	37.9	7.74
IPI00113043.1	Mitochondrial import receptor subunit TOM40B	36.69%	4	1	12	30																									0.162	0.126	-0.136	-0.099	-0.017	3	308	34.0	7.27
IPI00133289.1	Mitochondrial import receptor subunit TOM7 homolog	69.09%	1	1	5	18																									0.123	0.075	0.027	0.089	0.171	3	55	6.2	10.17
IPI00377728.3	Mitochondrial import receptor subunit TOM70	88.38%	2	24	71	667	-0.340	0.118	-0.309	0.076	-0.371	38	0.043	-0.122	0.161	0.004	0.317	22	0.126	0.087	0.001	0.027	0.038	41	-0.006	0.000	-0.007	0.047	-0.109	33	0.122	-0.025	-0.069	-0.063	-0.066	66	611	67.5	7.53
IPI00124389.1	Mitochondrial intermembrane space import and assembly protein 40	51.80%	1	4	9	81	-0.208	0.128	-0.168	-0.184	-0.155	6	-0.007	-0.056	-0.189	-0.069	-0.065	15																			139	15.5	4.32
IPI00471368.3	Mitochondrial ribonuclease P protein 1	68.84%	1	5	39	129	-0.290	0.040	-0.258	-0.128	-0.309	7																									414	48.4	9.38
IPI00120199.1	Mitochondrial-processing peptidase subunit alpha	80.15%	2	11	47	171	-0.091	0.047	-0.152	0.051	-0.091	10							-0.144	0.025	-0.017	-0.028	-0.056	6	-0.213	-0.141	-0.236	-0.100	-0.060	8							524	58.2	6.83
IPI00274656.6	Mitochondrial-processing peptidase subunit beta	68.10%	1	8	41	191							-0.016	-0.056	-0.062	0.200	-0.035	6	-0.077	0.076	-0.009	-0.117	-0.149	9	-0.009	0.045	0.106	0.043	-0.003	9	-0.053	-0.111	-0.066	-0.034	-0.085	3	489	54.6	6.99
IPI00119663.3	Mitogen-activated protein kinase 1	91.06%	15	18	42	397	0.016	-0.163	0.006	-0.117	0.135	16	-0.281	-0.029	-0.079	-0.041	-0.194	21	-0.264	-0.145	-0.267	-0.089	-0.155	5	-0.025	-0.001	-0.079	0.051	0.064	20	0.096	0.004	-0.048	-0.083	0.041	50	358	41.2	6.98
IPI00230277.3	Mitogen-activated protein kinase 3	70.00%	15	5	31	276	0.176	-0.155	0.096	-0.108	0.373	16	-0.119	0.059	-0.057	0.277	-0.068	16							-0.062	0.011	-0.099	0.110	0.040	10	-0.069	-0.077	-0.082	0.024	0.131	22	380	43.0	6.61
IPI00626752.2	Mitotic checkpoint protein BUB3	71.47%	1	11	28	216	-0.072	0.024	-0.022	0.020	-0.109	19	-0.292	-0.160	-0.133	-0.147	-0.214	16	-0.019	0.006	-0.034	0.009	0.057	20	0.135	-0.004	0.064	0.026	0.011	13							326	36.9	6.84
IPI00850385.1	mitotic spindle assembly checkpoint protein MAD2A-like	74.63%	2	4	16	53	-0.346	-0.105	-0.355	0.066	-0.299	8																									205	23.6	5.20
IPI00110588.4	Moesin	93.07%	1	40	100	1834	0.199	0.000	0.230	0.013	0.195	173	0.116	-0.019	0.236	-0.069	0.202	119	-0.023	-0.024	-0.004	0.026	-0.012	197	0.072	0.064	0.062	0.033	0.075	86	0.027	-0.077	-0.079	-0.080	0.055	21	577	67.7	6.60
IPI00165731.3	Monoacylglycerol lipase ABHD12	61.31%	4	6	26	82													0.043	0.121	-0.155	0.560	0.192	3							-0.117	0.062	0.285	0.033	0.268	9	398	45.2	8.72
IPI00137194.1	Monocarboxylate transporter 1	59.03%	1	8	28	249	-0.150	0.085	-0.264	0.033	-0.221	25	-0.292	-0.068	-0.240	0.026	-0.144	15	0.073	0.109	-0.071	-0.054	-0.194	20	0.320	-0.120	0.163	-0.114	0.328	17	-0.045	0.043	0.088	0.151	0.006	7	493	53.2	7.47
IPI00118910.1	Monocarboxylate transporter 4	38.94%	3	3	12	35																			0.346	-0.007	0.350	-0.031	0.428	7							470	50.3	7.96
IPI00308990.3	Monocyte differentiation antigen CD14	69.67%	1	7	29	98	0.010	-0.025	0.003	0.023	-0.125	11																									366	39.2	5.21
IPI00875833.2	Monofunctional C1-tetrahydrofolate synthase, mitochondrial	94.47%	1	47	129	1230	-0.108	0.111	-0.131	0.037	-0.155	59	0.565	0.085	0.450	0.192	0.340	78	0.139	0.059	0.149	-0.022	0.052	66	0.296	0.001	0.332	-0.020	0.318	90	0.011	-0.122	-0.016	-0.038	-0.234	12	977	105.7	7.02
IPI00132703.1	MORF4 family-associated protein 1	88.80%	1	3	13	31	-0.014	-0.213	-0.023	-0.095	0.070	3													0.495	0.307	0.376	0.156	0.477	5							125	14.2	4.73
IPI00463666.4	Mortality factor 4-like protein 2	60.07%	5	5	26	74							0.323	-0.001	0.299	-0.240	0.305	3	-0.194	-0.017	-0.012	0.027	0.100	8													288	32.2	9.72
IPI00123276.1	MOSC domain-containing protein 2, mitochondrial	92.60%	6	7	38	212	0.307	0.165	0.249	0.092	0.312	11	0.256	0.108	0.264	0.289	0.119	13	0.391	0.197	0.238	-0.189	0.094	6							0.023	-0.123	-0.160	-0.119	-0.228	15	338	38.2	8.68
IPI00314870.1	Mothers against decapentaplegic homolog 3	61.88%	5	2	32	97	0.130	-0.004	-0.069	0.131	-0.125	7							0.160	0.098	0.375	0.373	0.103	3													425	48.0	7.15
IPI00973614.1	Mothers against decapentaplegic-like protein 4 splice variant 2	62.94%	4	3	37	191	0.103	0.058	-0.260	-0.029	-0.139	3	0.007	-0.208	0.856	0.404	-0.022	3																			402	44.7	7.80
IPI00134044.1	M-phase phosphoprotein 6	54.66%	1	2	10	28							0.231	0.139	-0.010	0.000	0.059	4																			161	19.1	5.30
IPI00420832.1	Mps one binder kinase activator-like 3	74.22%	3	3	16	57							-0.213	-0.076	0.096	-0.223	0.006	4																			225	26.0	5.78
IPI00225719.3	mRNA export factor	80.16%	2	10	26	138							-0.346	-0.085	-0.646	-0.082	-0.409	6	0.172	0.087	-0.105	0.181	0.006	9	0.068	-0.022	-0.006	-0.092	-0.037	3							368	40.9	7.83
IPI00132578.1	mRNA turnover protein 4 homolog	81.59%	5	9	31	114	-0.119	0.057	-0.004	0.100	-0.109	4							-0.103	0.087	-0.116	-0.085	-0.085	6	-0.022	-0.074	0.001	-0.042	0.000	15							239	27.5	8.54
IPI00120030.1	Mu-crystallin homolog	45.69%	1	10	18	115																									0.037	0.170	-0.062	-0.010	-0.054	37	313	33.5	5.67
IPI00128152.1	Multidrug resistance protein 1	62.07%	3	11	89	239							0.070	-0.224	-0.158	-0.147	-0.022	13																			1276	140.9	8.34
IPI00129915.3	Multidrug resistance-associated protein 1	72.97%	4	9	121	331	-0.069	0.234	-0.338	0.050	-0.324	5	0.038	0.073	0.033	-0.051	0.118	3	0.262	0.099	0.424	0.253	0.134	3													1528	171.1	7.36
IPI00129205.2	Multidrug resistance-associated protein 5	58.01%	1	2	95	296							-0.271	-0.050	-0.525	-0.023	-0.279	3																			1436	161.0	8.60
IPI00322096.8	Multifunctional protein ADE2	96.94%	3	23	56	573	0.065	-0.068	0.122	-0.001	0.124	64	0.034	-0.007	0.003	0.013	0.153	53	-0.019	-0.151	-0.034	0.030	0.034	25	0.402	0.077	0.307	0.058	0.293	18	-0.136	0.110	0.180	0.188	0.099	12	425	47.0	7.23
IPI00170316.1	Multiple coagulation factor deficiency protein 2 homolog	64.14%	1	2	8	53	-0.144	-0.047	0.213	0.275	-0.183	3	0.073	-0.246	0.037	-0.286	0.087	9							-0.006	-0.216	0.147	-0.068	0.139	8							145	16.2	4.72
IPI00123223.2	Murinoglobulin-1	75.95%	3	8	111	442	0.016	0.036	-0.010	0.031	-0.069	5	0.415	0.292	0.313	0.244	-0.028	4	-0.340	-0.075	-0.353	0.108	-0.150	12	-0.102	0.119	-0.069	0.030	-0.133	3	0.294	0.187	0.165	0.305	0.309	7	1476	165.2	6.42
IPI00331361.2	Myb-binding protein 1A	92.56%	6	68	164	2542	-0.245	0.088	-0.286	0.036	-0.300	196	0.364	0.123	0.229	0.092	0.231	144	-0.059	0.046	-0.071	-0.038	-0.062	156	-0.100	0.029	-0.076	0.020	-0.181	187							1344	151.9	8.95
IPI00458777.2	myc box-dependent-interacting protein 1 isoform 2	77.78%	3	1	49	492	-0.201	-0.026	-0.050	0.017	-0.062	9	-0.932	-0.074	-0.671	-0.123	-0.796	15	-0.286	-0.121	-0.241	0.059	-0.117	55	0.244	0.209	0.202	0.031	0.207	7	0.070	0.041	0.044	0.021	0.058	86	477	52.7	5.24
IPI00885879.4	myelin gene regulatory factor	57.19%	3	7	58	229	0.695	-0.573	0.411	-0.057	0.562	6																									1112	120.6	7.33
IPI00117735.1	Myelin protein P0	48.79%	2	3	20	59																									0.616	-0.048	0.516	0.001	0.132	6	248	27.6	9.47
IPI00785314.1	myelin-oligodendrocyte glycoprotein	77.33%	3	11	21	169																									0.024	0.414	0.331	0.358	0.446	60	247	28.4	7.96
IPI00116372.1	Myeloid leukemia factor 2	49.80%	1	2	18	77							-0.010	-0.183	-0.271	-0.117	0.038	7													-0.260	0.057	0.014	-0.157	-0.057	3	247	28.0	6.98
IPI00132938.2	Myeloid-associated differentiation marker	43.44%	1	5	16	119	0.347	-0.114	0.316	0.029	0.262	11	0.249	0.051	0.271	0.430	0.464	7	0.710	0.235	0.576	0.192	0.202	7													320	35.3	8.31
IPI00330649.4	myosin IE	77.51%	4	1	111	307	-0.186	-0.105	0.014	-0.023	-0.026	19																									1107	126.7	9.07
IPI00109044.8	myosin light chain, regulatory B-like	92.44%	5	3	19	387	0.189	-0.035	0.217	-0.029	0.212	23	0.705	0.077	0.594	-0.094	0.719	9	-0.226	-0.157	-0.293	0.047	-0.122	19	0.215	-0.009	0.267	-0.057	0.279	43	-0.029	-0.152	-0.109	-0.142	-0.158	21	172	19.9	4.81
IPI00132705.1	Myosin regulatory light chain 12B	83.72%	2	1	18	352	0.356	-0.035	0.287	-0.039	0.396	27	0.807	0.079	0.767	-0.028	0.736	6	-0.228	-0.156	-0.288	0.062	-0.120	18	0.131	-0.019	-0.077	-0.057	0.141	25	-0.029	-0.152	-0.109	-0.142	-0.158	21	172	19.8	4.84
IPI00750595.2	Myosin regulatory light polypeptide 9	78.49%	2	1	13	331	0.323	-0.034	0.235	-0.007	0.323	12	0.880	0.077	0.824	-0.068	0.751	5	-0.050	-0.152	-0.253	0.047	0.033	14	0.162	-0.012	0.058	-0.085	0.197	20	-0.038	-0.048	-0.099	-0.122	-0.045	18	172	19.8	4.92
IPI00466187.5	myosin X	71.14%	5	2	179	627	-0.056	-0.060	0.152	0.013	0.179	9																									2062	237.2	6.25
IPI00605455.5	myosin XVIIIb	79.85%	1	2	273	872																									-0.571	-0.388	-0.194	0.094	0.031	4	2605	288.7	6.67
IPI00380896.1	Myosin-1	77.14%	11	1	236	871							0.171	0.179	0.224	0.026	0.204	6	-0.013	0.031	0.034	0.016	-0.134	10													1942	223.2	5.76
IPI00938530.1	myosin-11 isoform 1	82.92%	9	2	245	1534	0.261	0.021	0.325	0.003	0.297	44	0.635	0.083	0.552	-0.039	0.539	62	-0.292	-0.151	-0.308	0.041	-0.116	107	0.395	-0.003	0.292	0.009	0.287	41	-0.009	-0.083	-0.057	-0.158	-0.032	46	1938	223.2	5.50
IPI00380895.7	Myosin-3	75.93%	6	1	215	674							0.171	0.179	0.224	0.026	0.204	6	-0.013	0.031	0.034	0.016	-0.134	10													1940	223.7	5.81
IPI00130653.1	Myosin-7	76.54%	6	2	236	809							0.171	0.179	0.224	0.026	0.204	6	-0.013	0.031	0.034	0.016	-0.134	10													1935	222.7	5.76
IPI00755200.3	Myosin-7B	78.36%	6	2	222	760							0.115	0.118	0.210	0.151	0.233	7	-0.035	0.024	-0.017	0.036	-0.144	11													1941	221.4	5.88
IPI00123181.4	Myosin-9	90.26%	14	123	308	5119	0.264	0.000	0.279	-0.001	0.273	317	0.669	0.044	0.618	0.011	0.548	334	-0.281	-0.139	-0.281	0.077	-0.095	631	0.307	-0.003	0.280	-0.004	0.285	329	-0.048	-0.181	-0.147	-0.173	-0.047	125	1960	226.2	5.66
IPI00408215.1	myosin-Ib isoform 1	73.50%	6	9	127	422	0.000	-0.038	-0.116	-0.103	-0.091	14																									1136	132.0	9.33
IPI00988257.1	myosin-Ie-like	77.35%	4	1	106	317	-0.056	-0.054	-0.051	-0.029	-0.029	22																									1051	120.4	9.14
IPI00228583.5	Myotrophin	75.42%	1	5	10	134	-0.163	-0.054	-0.059	-0.028	-0.045	12	-1.003	-0.231	-0.991	-0.385	-0.624	7	-0.108	-0.219	-0.360	0.099	0.184	12	0.390	0.158	0.462	0.100	0.199	8	0.114	-0.117	-0.141	-0.035	0.003	15	118	12.9	5.52
IPI00752390.4	myotubularin-related protein 5 isoform 1	53.14%	4	8	110	239																									-0.131	-0.001	-0.082	-0.149	-0.089	7	1893	211.3	7.33
IPI00123689.1	Myotubularin-related protein 6	73.91%	1	3	62	150	0.043	0.069	0.299	-0.034	0.099	5																									617	70.9	7.83
IPI00131035.2	Myotubularin-related protein 7	74.85%	5	4	48	96																			-0.633	-0.103	-0.278	0.041	-0.474	4							660	75.6	6.43
IPI00229534.5	Myristoylated alanine-rich C-kinase substrate	69.26%	1	11	24	744	0.316	0.122	0.340	0.055	0.174	76	2.250	0.126	2.487	0.261	2.163	6	0.187	-0.054	0.343	-0.128	0.185	44	0.741	-0.050	0.678	0.038	0.675	49	-0.006	0.194	0.166	0.234	0.141	42	309	29.6	4.34
IPI00830442.2	N(2),N(2)-dimethylguanosine tRNA methyltransferase isoform 2	74.39%	5	5	50	155							0.152	-0.123	0.318	0.023	0.139	13							0.007	-0.019	0.103	-0.241	0.217	3							656	71.6	7.83
IPI00131231.3	N(4)-(beta-N-acetylglucosaminyl)-L-asparaginase	69.65%	2	2	17	53							-0.400	-0.111	-0.134	0.437	-0.198	3																			346	37.0	6.44
IPI00109482.3	N(G),N(G)-dimethylarginine dimethylaminohydrolase 1	76.49%	2	14	30	206	0.094	-0.189	0.235	-0.047	0.169	11	-0.751	-0.246	-0.761	-0.213	-0.515	4							-0.028	0.013	-0.162	0.014	-0.209	4	0.024	0.000	-0.042	-0.125	-0.042	47	285	31.4	5.97
IPI00336881.1	N(G),N(G)-dimethylarginine dimethylaminohydrolase 2	55.44%	3	5	19	119													0.036	0.029	0.037	0.144	-0.065	12	0.347	0.034	0.500	-0.035	0.612	10	-0.048	0.021	0.010	-0.044	0.075	6	285	29.6	6.01
**IPI00470995.1**	**N-acetylgalactosamine kinase**	**64.41%**	**3**	**2**	**30**	**88**	-0.245	0.009	-0.492	0.110	-0.346	3																									**458**	**50.5**	**6.90**
IPI00221426.3	N-acetylglucosamine-6-sulfatase	63.24%	1	4	39	158	0.294	0.058	0.263	0.163	0.372	3																									544	61.1	8.24
IPI00276866.1	N-acetyltransferase 10	71.97%	2	20	96	518	-0.035	0.044	-0.031	0.112	-0.123	13	0.052	0.001	0.014	0.017	-0.034	11	-0.082	0.050	-0.106	0.066	-0.120	7	0.151	-0.082	0.068	-0.034	0.016	25							1024	115.3	8.32
IPI00118121.3	NACHT, LRR and PYD domains-containing protein 4C	77.90%	1	3	99	319																			-0.292	0.017	-0.283	0.143	-0.224	5							982	112.6	6.92
IPI00309964.3	NAD(P) transhydrogenase, mitochondrial precursor	64.27%	6	23	74	578	-0.023	-0.037	-0.032	-0.131	-0.080	11	0.161	-0.032	0.309	-0.048	0.192	11	0.722	0.143	0.499	-0.016	0.581	18	-0.065	0.096	-0.063	0.023	-0.105	27	0.045	0.112	0.072	-0.007	0.040	39	1086	113.8	7.64
IPI00263899.6	NAD(P)H dehydrogenase [quinone] 1	74.82%	1	10	20	126							-0.608	-0.233	-0.331	-0.338	-0.013	17	-0.531	-0.191	-0.591	0.059	-0.358	7													274	30.9	8.72
IPI00115977.1	NAD-dependent malic enzyme, mitochondrial	88.62%	2	13	55	371	0.010	0.034	-0.044	0.009	-0.060	19	0.057	-0.038	0.102	0.100	0.043	20	0.225	0.087	0.132	0.098	0.054	18	-0.006	-0.009	-0.020	0.036	-0.099	18	0.114	-0.165	-0.100	-0.176	-0.053	4	589	65.8	7.61
IPI00344004.3	NADH dehydrogenase (Ubiquinone) 1 alpha subcomplex, 12	93.96%	1	10	19	135	0.037	-0.248	0.029	-0.013	-0.213	5							-0.769	0.089	-0.436	-0.184	-0.308	4							-0.044	0.048	0.024	-0.038	-0.041	37	149	17.6	9.50
IPI00132940.1	NADH dehydrogenase [ubiquinone] 1 alpha subcomplex subunit 1	70.00%	1	2	4	16																									0.084	-0.079	-0.253	0.084	0.092	4	70	8.1	9.48
IPI00116748.1	NADH dehydrogenase [ubiquinone] 1 alpha subcomplex subunit 10, mitoch	95.49%	1	14	42	258	0.024	0.070	-0.004	-0.037	-0.048	3	-0.285	-0.112	-0.181	0.026	-0.088	12	-0.034	0.027	0.265	0.209	-0.065	7	0.187	0.288	0.294	0.076	0.236	4	-0.128	-0.051	0.044	0.003	0.089	38	355	40.6	7.78
IPI00850427.1	NADH dehydrogenase [ubiquinone] 1 alpha subcomplex subunit 11-like	94.41%	2	2	9	45																									-0.019	-0.038	0.100	0.057	0.068	9	143	15.2	8.46
IPI00230715.5	NADH dehydrogenase [ubiquinone] 1 alpha subcomplex subunit 13	97.92%	1	8	19	129	-0.020	0.059	-0.072	0.011	0.041	7													0.088	-0.362	-0.203	-0.072	-0.189	5	0.041	0.092	-0.023	-0.034	-0.080	27	144	16.8	9.48
IPI00315302.5	NADH dehydrogenase [ubiquinone] 1 alpha subcomplex subunit 2	100.00%	1	7	19	133													-0.299	-0.070	-0.108	0.023	-0.100	10							-0.022	0.044	0.134	0.077	0.021	24	99	10.9	9.99
IPI00132216.1	NADH dehydrogenase [ubiquinone] 1 alpha subcomplex subunit 3	57.14%	2	5	5	36																									-0.152	0.047	0.033	-0.116	0.014	11	84	9.3	8.47
IPI00125929.2	NADH dehydrogenase [ubiquinone] 1 alpha subcomplex subunit 4	92.68%	1	6	11	147	0.188	0.128	-0.191	-0.029	0.014	5	-0.234	0.157	0.057	0.294	-0.226	7	0.346	0.137	0.167	0.057	0.072	11	-0.120	-0.139	-0.149	-0.025	-0.059	9	0.036	0.069	0.081	-0.010	0.033	31	82	9.3	9.52
IPI00331332.6	NADH dehydrogenase [ubiquinone] 1 alpha subcomplex subunit 5	88.79%	2	9	14	91							0.653	0.087	0.735	0.422	0.003	4													-0.007	0.019	0.047	-0.013	0.011	28	116	13.4	8.10
IPI00133399.1	NADH dehydrogenase [ubiquinone] 1 alpha subcomplex subunit 6	77.86%	1	5	16	125	0.050	0.145	0.003	0.108	-0.026	4	-0.181	0.007	-0.147	0.014	-0.034	4	-0.028	0.087	0.004	0.073	-0.175	6	-0.069	-0.072	-0.167	-0.095	0.026	7	-0.044	-0.114	-0.031	-0.116	-0.063	23	131	15.3	10.11
IPI00130322.5	NADH dehydrogenase [ubiquinone] 1 alpha subcomplex subunit 7	100.00%	1	8	28	224	0.130	0.064	0.064	0.017	0.081	6	-0.057	-0.152	-0.204	0.258	-0.484	3	-0.047	-0.069	-0.146	0.070	-0.224	11	-0.112	-0.066	-0.028	0.010	-0.065	4	0.040	0.029	-0.089	-0.065	-0.012	25	113	12.6	10.17
IPI00120984.5	NADH dehydrogenase [ubiquinone] 1 alpha subcomplex subunit 8	68.02%	1	7	16	96	-0.028	-0.012	0.055	0.034	-0.069	6	-0.006	0.036	0.188	0.034	0.031	5	0.256	0.084	0.047	-0.006	-0.103	9	-0.003	-0.079	-0.016	-0.063	-0.168	4	-0.023	-0.133	-0.056	-0.060	0.023	21	172	20.0	8.46
IPI00120212.2	NADH dehydrogenase [ubiquinone] 1 alpha subcomplex subunit 9, mitocho	94.96%	1	23	48	391	-0.204	0.138	0.059	-0.017	-0.059	11	0.140	0.016	0.576	0.316	0.602	5	0.110	0.105	0.006	-0.079	-0.120	11	-0.193	-0.037	-0.238	0.003	-0.180	20	-0.041	0.004	0.017	-0.050	-0.016	84	377	42.5	9.74
IPI00121288.5	NADH dehydrogenase [ubiquinone] 1 beta subcomplex subunit 10	75.57%	2	6	20	145							-0.105	-0.038	-0.089	-0.062	0.119	7	0.029	0.101	0.257	-0.045	-0.114	6							-0.181	-0.086	-0.094	-0.165	-0.062	27	176	21.0	8.03
IPI00114246.2	NADH dehydrogenase [ubiquinone] 1 beta subcomplex subunit 11, mitocho	82.12%	1	6	20	204	-0.071	0.038	0.031	0.130	-0.085	15	0.072	0.181	0.708	0.416	-0.026	5	0.102	0.137	0.001	-0.013	-0.186	8	0.140	0.092	0.081	-0.059	0.166	4	-0.059	0.034	-0.037	-0.007	-0.047	29	151	17.4	5.22
IPI00133403.1	NADH dehydrogenase [ubiquinone] 1 beta subcomplex subunit 3	76.92%	1	2	11	45	-0.519	0.013	-0.318	0.199	-0.502	3																			0.027	0.024	0.007	-0.111	0.084	9	104	11.7	9.04
IPI00132390.5	NADH dehydrogenase [ubiquinone] 1 beta subcomplex subunit 4	91.47%	2	5	16	97													0.122	0.185	0.116	0.357	-0.134	7							-0.088	-0.029	-0.100	-0.032	0.037	19	129	15.1	9.89
IPI00132531.1	NADH dehydrogenase [ubiquinone] 1 beta subcomplex subunit 5, mitochon	66.67%	5	6	19	117	-0.309	0.033	-0.402	0.001	-0.591	4							0.011	0.118	-0.006	-0.092	-0.155	9							0.047	0.062	0.079	0.157	0.098	22	189	21.7	9.41
IPI00133215.3	NADH dehydrogenase [ubiquinone] 1 beta subcomplex subunit 7	74.45%	1	5	16	85	-0.003	-0.077	-0.274	-0.020	-0.168	5	0.613	0.047	0.092	0.245	0.246	4													0.027	-0.035	0.104	0.187	0.092	13	137	16.3	8.18
IPI00387430.1	NADH dehydrogenase [ubiquinone] 1 beta subcomplex subunit 8, mitochon	80.65%	4	5	19	88													-0.028	0.048	-0.366	0.029	-0.010	5							-0.062	-0.053	-0.032	-0.083	-0.045	18	186	21.9	6.64
IPI00132623.3	NADH dehydrogenase [ubiquinone] 1 beta subcomplex subunit 9	79.33%	1	6	15	100													-0.122	0.065	-0.285	-0.167	-0.379	8							-0.131	-0.042	-0.035	0.143	-0.053	17	179	22.0	7.80
IPI00132050.1	NADH dehydrogenase [ubiquinone] 1 subunit C2	94.17%	3	7	17	99																									-0.085	0.007	-0.050	-0.037	-0.069	21	120	14.2	9.20
IPI00130460.1	NADH dehydrogenase [ubiquinone] flavoprotein 1, mitochondria	82.54%	6	18	43	199							0.132	0.132	0.009	0.004	0.098	5	0.036	0.085	-0.094	-0.017	-0.045	7							0.013	0.037	0.089	0.057	0.047	53	464	50.8	8.21
IPI00403381.1	NADH dehydrogenase [ubiquinone] flavoprotein 3, mitochondria	85.58%	2	2	16	93	0.112	0.047	0.163	-0.060	0.006	9							0.245	0.119	0.013	-0.201	0.027	9							0.054	-0.082	0.020	-0.050	-0.007	12	104	11.8	9.35
IPI00128023.3	NADH dehydrogenase [ubiquinone] iron-sulfur protein 2, mitochondria	94.38%	3	14	44	228	-0.004	-0.160	-0.150	-0.097	0.011	7							0.194	0.068	0.123	-0.010	0.027	5	-0.112	0.040	0.076	-0.103	0.091	6	-0.044	-0.028	-0.038	-0.037	-0.083	49	463	52.6	6.99
IPI00121309.2	NADH dehydrogenase [ubiquinone] iron-sulfur protein 3, mitochondria	87.45%	3	13	28	161	0.098	0.126	0.036	0.166	0.069	7	-0.035	-0.092	0.009	0.128	-0.423	5	0.198	0.009	0.081	0.114	-0.022	6							-0.038	0.023	-0.038	-0.059	-0.051	37	263	30.1	7.17
IPI00229008.2	NADH dehydrogenase [ubiquinone] iron-sulfur protein 4, mitochondria	94.29%	2	9	30	241	-0.016	0.000	-0.007	0.095	-0.003	3	0.026	0.064	0.027	-0.102	0.031	3	-0.136	0.058	-0.004	-0.105	-0.198	11							-0.077	0.095	-0.007	-0.109	0.124	26	175	19.8	9.99
IPI00128345.2	NADH dehydrogenase [ubiquinone] iron-sulfur protein 6, mitochondria	93.97%	5	6	19	117	0.156	0.110	0.130	0.092	-0.003	5							0.000	0.091	-0.016	0.297	0.041	3							-0.004	0.009	-0.042	0.034	-0.004	22	116	13.0	8.65
IPI00120232.1	NADH dehydrogenase [ubiquinone] iron-sulfur protein 7, mitochondria	93.30%	1	7	30	164	0.076	0.044	-0.009	-0.035	-0.111	6	-0.347	-0.038	-0.229	-0.031	-0.231	4	-0.576	-0.154	-0.315	0.014	-0.318	6	0.019	-0.077	0.030	-0.144	0.052	3	-0.128	0.083	0.006	-0.060	0.062	16	224	24.7	9.92
IPI00170093.3	NADH dehydrogenase [ubiquinone] iron-sulfur protein 8, mitochondria	76.42%	1	8	27	158	0.108	0.229	0.126	0.111	0.073	5							-0.108	-0.026	-0.279	-0.068	-0.216	8	0.065	-0.037	-0.060	-0.001	-0.038	3	-0.044	-0.019	-0.001	-0.076	-0.069	27	212	24.0	6.21
IPI00355228.3	NADH dehydrogenase subunit 2	45.80%	2	3	11	36																									-0.088	0.207	0.144	0.059	0.138	6	345	38.7	9.92
IPI00480233.1	NADH dehydrogenase subunit 5	57.17%	2	4	28	145																									-0.149	0.235	0.280	0.184	0.115	13	607	68.4	9.03
IPI00308882.5	NADH-ubiquinone oxidoreductase 75 kDa subunit, mitochondrial	91.06%	1	33	83	759	-0.128	-0.019	-0.071	-0.006	-0.012	39	-0.173	-0.069	-0.131	0.057	-0.236	10	-0.039	0.103	-0.014	-0.026	-0.218	36	-0.083	-0.130	-0.147	-0.059	-0.057	21	-0.062	-0.065	-0.028	-0.065	-0.042	102	727	79.7	5.72
IPI00341550.1	NADH-ubiquinone oxidoreductase chain 1	25.16%	1	3	8	24																									0.239	0.280	0.163	0.297	0.407	9	318	36.0	6.93
IPI00116843.1	NADH-ubiquinone oxidoreductase chain 4	41.83%	1	3	18	75	-0.606	-0.198	-0.839	0.014	-0.881	3																									459	51.8	9.38
IPI00128857.2	NADP-dependent malic enzyme	96.33%	3	26	65	518	0.230	-0.062	0.307	-0.045	0.275	53	-0.037	0.001	0.124	0.104	0.076	39	-0.450	-0.139	-0.494	0.054	-0.114	4	-0.059	0.084	-0.076	0.021	-0.125	26	0.027	-0.142	-0.139	-0.114	-0.076	37	572	63.9	7.44
IPI00308976.2	NADP-dependent malic enzyme, mitochondrial	90.23%	3	16	60	219																									0.187	-0.245	-0.224	-0.349	-0.231	41	604	67.1	7.83
IPI00621548.2	NADPH--cytochrome P450 reductase	78.32%	5	24	60	543	0.047	0.000	0.048	-0.019	0.069	36	-0.664	-0.004	-0.576	0.013	-0.567	50	0.497	0.126	0.417	-0.031	0.241	29	-0.095	0.030	-0.209	0.013	-0.201	40	-0.109	0.003	-0.095	-0.017	0.007	12	678	77.0	5.53
IPI00465765.2	N-alpha-acetyltransferase 10, NatA catalytic subunit	77.45%	9	1	23	152	-0.167	-0.072	0.017	0.034	-0.068	9	-0.221	-0.065	-0.206	0.020	-0.163	8	-0.072	-0.055	0.061	0.158	0.044	12													235	26.5	5.64
IPI00758413.2	N-alpha-acetyltransferase 10, NatA catalytic subunit isoform 2	65.33%	7	1	22	209	-0.068	-0.069	-0.097	0.055	-0.144	12	-0.221	-0.071	-0.206	0.020	-0.163	8	-0.162	-0.047	0.084	0.162	0.088	13	-0.342	-0.421	-0.808	-0.188	-0.394	6							225	24.8	6.61
IPI00387212.1	N-alpha-acetyltransferase 15, NatA auxiliary subunit	80.12%	2	26	111	603	-0.170	0.030	-0.144	0.050	-0.165	18	-0.255	0.076	-0.209	0.043	-0.144	37	-0.167	-0.004	-0.122	0.077	-0.175	29	-0.209	0.210	0.026	0.052	-0.214	10							865	100.9	7.62

IPI00119509.1	N-alpha-acetyltransferase 16, NatA auxiliary subunit	68.98%	3	1	66	183
IPI00119162.1	N-alpha-acetyltransferase 20, NatB catalytic subunit isoform 2	47.87%	4	2	9	29
IPI00263042.3	N-alpha-acetyltransferase 38, NatC auxiliary subunit	100.00%	1	3	13	100
IPI00111831.2	Nascent polypeptide-associated complex subunit alpha, muscle-specific forr	79.93%	2	10	206	1103
IPI00467223.1	NCK-interacting protein with SH3 domain	78.99%	1	3	63	185
IPI00127021.1	NEDD8	90.12%	1	6	13	114
IPI00404981.1	NEDD8-activating enzyme E1 regulatory subunit	88.39%	3	12	53	289
IPI00169448.1	NEDD8-conjugating enzyme Ubc12	81.42%	4	9	23	155
IPI00221446.1	Negative elongation factor A	60.19%	1	3	44	104
IPI00277028.4	Negative elongation factor B	76.38%	4	4	48	110
IPI00117416.1	Neighbor of COX4	74.88%	1	7	18	92
IPI00132005.1	Neudesin	95.32%	1	6	15	48
IPI00230665.3	neural cell adhesion molecule 1 isoform 3	87.17%	6	8	98	1021
IPI00785371.2	neural cell adhesion molecule L1	57.27%	8	12	69	295
IPI00468996.2	Neural Wiskott-Aldrich syndrome protein	60.48%	1	2	35	102
IPI00460715.4	neurexin III isoform 1	73.09%	7	10	141	700
IPI00263638.8	Neurocalcin-delta	92.23%	4	7	24	159
IPI00135563.1	Neurocan core protein	38.88%	2	11	60	207
IPI00123059.1	Neuroendocrine protein 7B2	64.62%	1	4	14	53
IPI00329927.4	Neurofascin	48.71%	1	3	84	361
IPI00930898.1	neurofascin isoform 2 precursor	58.28%	3	3	94	371
IPI00114241.2	Neurofilament heavy polypeptide	77.25%	2	50	117	608
IPI00554928.3	Neurofilament light polypeptide	87.11%	1	36	73	650
IPI00323800.6	Neurofilament medium polypeptide	87.03%	4	49	119	760
IPI00380227.1	Neurogranin	39.74%	1	3	4	39
IPI00119201.1	Neuroguidin	65.08%	1	2	26	98
IPI00858277.1	Neuroligin 4*	35.56%	4	1	21	39
IPI00314829.5	Neurolysin, mitochondrial	68.04%	2	12	68	245
IPI00128973.1	Neuromodulin	91.63%	1	19	35	453
IPI00466984.3	neuron navigator 2 isoform 1	80.39%	6	6	263	960
IPI00113521.5	Neuronal calcium sensor 1	91.05%	1	4	18	41
IPI00122974.1	Neuronal membrane glycoprotein M6-a	31.29%	1	6	10	74
IPI00125992.1	Neuronal pentraxin-1	71.06%	1	7	55	269
IPI00222801.6	Neuronal proto-oncogene tyrosine-protein kinase Src	78.56%	2	6	47	154
IPI00230310.5	Neuron-specific calcium-binding protein hippocalcin	98.45%	5	4	26	275
IPI00123996.2	Neuropilin-1	62.08%	1	5	54	132
IPI00135130.3	Neutral amino acid transporter A	67.29%	1	7	31	228
IPI00403586.3	Neutral cholesterol ester hydrolase 1	60.78%	1	5	21	99
IPI00400168.3	Nexilin	71.17%	1	2	57	133
IPI00881509.2	NFU1 iron-sulfur cluster scaffold homolog, mitochondrial	71.37%	2	2	18	133
IPI00227730.1	NHL repeat-containing protein 2	66.62%	2	5	51	217
IPI00621272.3	NHP2-like protein 1	75.00%	5	8	20	152
IPI00330695.5	Niban-like protein 1	76.64%	2	24	76	507
IPI00226958.3	Nicalin	59.68%	2	11	40	164
IPI00118674.8	Nicastrin	75.00%	1	7	58	213
IPI00320188.5	Nicotinamide phosphoribosyltransferase	72.91%	1	10	48	208
IPI00132600.1	Niemann-Pick C1 protein	55.32%	3	3	50	116
IPI00875732.2	NIF3-like protein 1	77.66%	2	4	31	66
IPI00222429.6	Nodal modulator 1	86.33%	1	22	111	568
IPI00720005.1	noelin isoform a	62.06%	3	5	29	67
IPI00114295.5	Noelin-2	64.29%	1	2	26	36
IPI00338745.4	Non-histone chromosomal protein HMG-14	78.13%	3	6	10	534
IPI00990954.1	non-histone chromosomal protein HMG-17-like	55.56%	2	1	13	109
IPI00124677.6	Notchless protein homolog 1	83.09%	1	4	46	173
IPI00462855.3	N-terminal kinase-like protein	54.47%	1	7	42	85
IPI00138158.1	NTF2-related export protein 1	24.29%	1	1	3	8
IPI00830976.1	nuclear autoantigenic sperm protein isoform 2	76.46%	4	21	72	440
IPI00458056.3	Nuclear cap-binding protein subunit 1	63.04%	1	13	56	229
IPI00132019.1	Nuclear cap-binding protein subunit 2	76.92%	2	3	15	70
IPI00380737.1	Nuclear envelope pore membrane protein POM 121	35.33%	2	5	59	192
IPI00123474.11	Nuclear factor NF-kappa-B p100 subunit	60.62%	3	12	65	224
IPI00132942.1	Nuclear migration protein nudC	95.18%	1	25	56	796
IPI00263048.2	nuclear mitotic apparatus protein 1	95.03%	4	30	321	2337
IPI00221767.1	Nuclear pore complex protein Nup107	81.21%	3	17	91	349
IPI00320303.2	Nuclear pore complex protein Nup133	81.73%	1	15	98	333
IPI00453821.2	Nuclear pore complex protein Nup155	75.84%	7	31	120	711
IPI00229722.3	Nuclear pore complex protein Nup214	48.25%	2	18	98	538
IPI00120572.5	Nuclear pore complex protein Nup50	85.41%	1	11	43	331
**IPI00225192.1**	**Nuclear pore complex protein Nup54**	**61.18%**	**1**	**8**	**42**	**166**
IPI00461532.4	Nuclear pore complex protein Nup85	65.85%	3	6	59	214
IPI00139994.1	Nuclear pore glycoprotein p62	37.07%	2	9	20	116
IPI00313525.5	Nuclear receptor coactivator 5	70.29%	3	9	55	306
IPI00115588.4	Nuclear RNA export factor 1	85.11%	1	19	76	419
IPI00989983.1	nuclear transport factor 2-like	85.23%	1	4	19	186
IPI00341869.5	Nuclear ubiquitous casein and cyclin-dependent kinases substrate	80.77%	2	9	27	417
IPI00321884.1	Nuclear valosin-containing protein-like	80.94%	1	9	87	385
IPI00955735.1	nuclease EXOG, mitochondrial isoform 1 precursor	59.18%	6	3	23	48
IPI00120886.3	Nuclease-sensitive element-binding protein 1	88.20%	7	10	38	2156
IPI00132314.1	Nucleobindin-1	75.16%	4	19	52	342
IPI00911151.1	nucleobindin-2 isoform 2	87.62%	2	11	43	145
IPI00761417.1	nucleolar and coiled-body phosphoprotein 1 isoform B	70.14%	4	28	93	888
IPI00828463.1	nucleolar complex protein 2 homolog	77.07%	1	6	61	191
IPI00128918.2	Nucleolar complex protein 3 homolog	65.30%	1	7	68	192
IPI00117642.3	Nucleolar GTP-binding protein 1	75.08%	1	13	82	317
IPI00116898.4	Nucleolar GTP-binding protein 2	80.77%	3	5	79	241
IPI00119760.4	Nucleolar protein 10	67.10%	1	4	62	244
IPI00353010.2	Nucleolar protein 14	78.26%	1	11	90	308
IPI00458704.2	Nucleolar protein 16	70.79%	2	6	19	85
IPI00134353.3	Nucleolar protein 3	53.18%	1	6	14	41
IPI00318048.5	Nucleolar protein 56	83.79%	8	25	73	548
IPI00463468.3	Nucleolar protein 58	84.33%	1	18	61	420
IPI00120691.4	Nucleolar RNA helicase 2	91.30%	3	51	122	1381
IPI00317794.5	Nucleolin	78.93%	2	63	111	2362
IPI00132462.1	Nucleolysin TIA-1	58.55%	6	3	31	128
IPI00108085.1	Nucleolysin TIAR	73.98%	3	5	43	178
IPI00127415.1	Nucleophosmin	96.92%	6	24	45	1269
IPI00131725.3	Nucleoplasmin-3	48.00%	1	2	10	47
IPI00330624.2	nucleoporin 153	63.54%	1	21	93	435
IPI00165794.1	Nucleoporin NDC1	56.76%	1	8	46	171
IPI00420602.3	Nucleoporin NUP188 homolog	68.22%	1	8	126	472
IPI00109615.2	Nucleoporin Nup37	72.70%	2	7	23	94
IPI00177202.2	Nucleoporin Nup43	49.47%	2	2	17	57
IPI00469331.2	Nucleoporin NUP53	77.85%	3	9	29	283
IPI00990246.1	Nucleoside diphosphate kinase	98.88%	5	7	30	815
IPI00131459.1	Nucleoside diphosphate kinase A	100.00%	3	2	24	517
IPI00929813.1	nucleosome assembly protein 1-like 1 isoform 1	70.33%	3	14	29	594
IPI00378026.4	Nucleus accumbens-associated protein 1	67.70%	1	3	34	116
IPI00342259.7	NudC domain-containing protein 1	79.38%	4	10	44	201
IPI00461281.2	NudC domain-containing protein 2	80.25%	2	2	11	170
IPI00280984.6	Numb-like protein	47.68%	1	3	41	112
IPI00135659.2	oligodendrocyte-myelin glycoprotein	69.75%	2	9	34	144
IPI00173343.3	Oligoribonuclease, mitochondrial (Fragment)	91.56%	2	9	28	146
IPI00119945.1	Omega-amidase NIT2	93.12%	1	11	33	178
IPI00227678.5	O-phosphoseryl-tRNA(Sec) selenium transferase	71.03%	2	1	45	148
IPI00170101.1	Optineurin	90.92%	1	9	76	242
IPI00129178.1	Ornithine aminotransferase, mitochondrial	84.05%	1	17	50	613
IPI00125923.1	Osteoclast-stimulating factor 1	68.84%	1	5	20	136
IPI00137459.6	OTU domain-containing protein 4	53.84%	5	4	69	197
IPI00119756.1	OX-2 membrane glycoprotein	71.22%	5	4	22	84
IPI00320165.11	oxidoreductase HTATIP2 isoform a	84.36%	3	6	26	122
IPI00462728.3	Oxysterol-binding protein	72.23%	4	4	73	183
IPI00755161.2	oxysterol-binding protein 1	78.76%	7	12	76	242
IPI00120790.2	p21-activated protein kinase-interacting protein 1	73.56%	1	3	38	129
IPI00132995.1	Paired mesoderm homeobox protein 2B	35.99%	2	1	15	28
IPI00129215.3	Pantetheinase	66.02%	1	10	30	165
IPI00648755.1	Paralemmin-2	76.33%	4	3	34	96
IPI00316088.3	Paralemmin-3	59.26%	1	15	39	170
IPI00895414.1	Parkinson disease (Autosomal recessive, early onset) 7	96.81%	11	14	22	587
IPI00230766.4	Parvalbumin alpha	90.91%	1	5	9	44
IPI00989436.1	PCNA-associated factor	96.25%	3	4	30	91
IPI00309768.7	PDZ and LIM domain protein 1	96.64%	1	14	33	316
IPI01007944.1	PDZ and LIM domain protein 5 isoform ENH3b	96.65%	4	1	40	210
IPI00273491.7	PDZ domain containing 8	67.04%	1	5	100	281
IPI00129107.1	PDZ domain-containing protein GIPC1	71.47%	2	4	25	90
IPI00554989.3	Peptidyl-prolyl cis-trans isomerase	86.23%	2	4	22	968
IPI00987580.1	Peptidyl-prolyl cis-trans isomerase	96.95%	1	2	21	468
IPI00135686.2	Peptidyl-prolyl cis-trans isomerase B	92.13%	2	14	29	444
IPI00130240.1	Peptidyl-prolyl cis-trans isomerase C	90.57%	1	5	14	94
IPI00659713.1	Peptidyl-prolyl cis-trans isomerase CWC27 homolog	52.03%	1	3	27	65
IPI00132966.3	Peptidyl-prolyl cis-trans isomerase D	87.30%	2	23	43	954
IPI00111343.1	Peptidyl-prolyl cis-trans isomerase E	87.71%	1	5	31	59
IPI00116228.1	Peptidyl-prolyl cis-trans isomerase F, mitochondrial	82.04%	1	4	23	134
IPI00944068.1	peptidyl-prolyl cis-trans isomerase FKBP10 isoform 2	78.79%	4	23	50	258
IPI00133931.1	Peptidyl-prolyl cis-trans isomerase FKBP11	49.75%	1	2	7	21
IPI00266899.6	Peptidyl-prolyl cis-trans isomerase FKBP1A	54.63%	1	2	12	55
IPI00133948.1	Peptidyl-prolyl cis-trans isomerase FKBP2	90.71%	1	7	12	118
IPI00126000.1	Peptidyl-prolyl cis-trans isomerase FKBP3	79.46%	1	12	34	206
IPI00230139.5	Peptidyl-prolyl cis-trans isomerase FKBP4	83.62%	4	21	58	705
IPI00338963.3	Peptidyl-prolyl cis-trans isomerase FKBP5	74.34%	2	10	51	175
IPI00120073.1	Peptidyl-prolyl cis-trans isomerase FKBP7	83.94%	1	7	25	73
IPI00130486.1	Peptidyl-prolyl cis-trans isomerase FKBP9	63.33%	1	9	30	79
IPI00349306.1	Peptidyl-prolyl cis-trans isomerase G	62.63%	1	4	69	183
IPI00848926.2	peptidyl-prolyl cis-trans isomerase H-like	87.94%	5	3	24	166
IPI00132093.1	Peptidyl-prolyl cis-trans isomerase NIMA-interacting 1	81.21%	2	5	16	121
IPI00109696.1	Peptidyl-prolyl cis-trans isomerase NIMA-interacting 4	87.02%	2	5	13	98
IPI00133229.1	Peptidyl-prolyl cis-trans isomerase-like 1	89.16%	1	5	15	72
IPI00229025.2	Peptidylprolyl isomerase domain and WD repeat-containing protein 1	65.79%	2	6	52	135
IPI00153702.2	peptidyl-tRNA hydrolase 2, mitochondrial isoform a	97.80%	2	8	20	140
IPI00319270.2	Perilipin-3	97.25%	2	13	56	524
IPI00320422.3	Periodic tryptophan protein 1 homolog	56.69%	2	5	30	70
IPI00944023.1	peripherin isoform 2	97.04%	4	1	83	783
IPI00129193.1	Periplakin	85.01%	1	48	243	922
IPI00121788.1	Peroxiredoxin-1	98.99%	5	14	28	595
IPI00117910.3	Peroxiredoxin-2	78.28%	3	10	20	377
IPI00116254.1	Peroxiredoxin-4	79.56%	3	9	25	244
IPI00555059.2	Peroxiredoxin-6	92.86%	3	1	24	571
IPI00127558.4	Peroxisomal acyl-coenzyme A oxidase 1	65.36%	3	5	45	173
IPI00134870.4	Peroxisomal acyl-coenzyme A oxidase 2	56.24%	2	3	38	90
IPI01026884.1	Peroxisomal biogenesis factor 5-like protein transcript variant 1	50.00%	6	3	44	98
IPI00127237.1	Peroxisomal membrane protein PEX14	67.02%	2	7	34	256
IPI00331628.5	Peroxisomal multifunctional enzyme type 2	77.55%	1	20	73	484
IPI01026792.1	PERQ amino acid-rich with GYF domain-containing protein 2 isoform b	69.65%	7	3	128	614
IPI00553784.1	PEST proteolytic signal-containing nuclear protein	94.94%	4	6	26	200
IPI00881789.1	PH and SEC7 domain-containing protein 3 isoform 3	92.02%	6	17	55	199
IPI00399702.6	PHD finger protein 6	72.95%	4	4	36	141
IPI00263874.3	Phenylalanyl-tRNA synthetase alpha chain	79.33%	4	20	56	401
IPI00322828.2	Phenylalanyl-tRNA synthetase beta chain	92.02%	2	21	79	440
IPI00311476.1	Phosducin-like protein 3	81.25%	1	4	21	77
IPI00850430.1	phosphate carrier protein, mitochondrial-like	93.28%	3	18	40	546
IPI00468999.4	Phosphatidate cytidylyltransferase 2	66.67%	4	6	29	108
IPI00137730.7	Phosphatidylethanolamine-binding protein 1	84.49%	7	10	15	356
IPI00109221.3	Phosphatidylinositide phosphatase SAC1	73.94%	1	13	53	262
IPI00115875.8	phosphatidylinositol 4-kinase type 3 alpha	66.39%	3	19	163	488
IPI00230003.7	Phosphatidylinositol transfer protein alpha isoform	93.36%	2	15	43	249
IPI00312067.4	Phosphatidylinositol-3,4,5-trisphosphate 5-phosphatase 2	76.85%	1	1	120	372
IPI00115708.1	Phosphatidylinositol-5-phosphate 4-kinase type-2 alpha	63.70%	3	3	35	147
IPI00336318.1	Phosphatidylinositol-5-phosphate 4-kinase type-2 beta	88.70%	2	4	42	169
IPI00129479.3	Phosphatidylinositol-5-phosphate 4-kinase type-2 gamma	46.56%	1	5	23	68
IPI00223060.1	phosphoenolpyruvate carboxykinase [GTP], mitochondria	83.51%	3	27	65	617
IPI00321922.2	Phosphofurin acidic cluster sorting protein 1	65.87%	4	11	81	262
IPI00555140.3	Phosphoglucomutase-1	90.75%	4	28	67	579
IPI00338302.1	Phosphoglucomutase-2	80.32%	1	7	58	296
IPI00555069.3	Phosphoglycerate kinase 1	98.08%	4	34	59	1566
IPI00457898.3	Phosphoglycerate mutase 1	96.85%	1	15	34	1189
IPI00230706.5	Phosphoglycerate mutase 2	94.47%	1	2	28	378
IPI00380195.1	Phosphoglycolate phosphatase	69.47%	2	13	35	203
IPI00226234.6	Phospholipase A-2-activating protein	83.00%	2	23	77	549
IPI00130624.1	Phospholipase D3	50.41%	1	3	20	35
IPI00660262.4	phospholipid hydroperoxide glutathione peroxidase, nuclear isoform 1 prec	89.33%	4	2	31	120
IPI00755389.2	phosphoribosyl pyrophosphate synthase-associated protein 1	89.61%	3	9	42	273
IPI00153950.1	Phosphoribosyl pyrophosphate synthase-associated protein 2	83.74%	2	6	38	125
IPI00265406.3	Phosphoribosylformylglycinamidine synthase	68.29%	2	19	75	413
IPI00331075.4	Phosphorylated adapter RNA export protein	81.30%	3	7	43	125
IPI00849478.1	phosphoserine aminotransferase-like isoform 2	92.16%	4	24	59	929
IPI00117146.1	Phosphoserine phosphatase	81.33%	2	9	19	233
**IPI00169622.3**	**Phytanoyl-CoA hydroxylase-interacting protein**	**58.48%**	**2**	**7**	**23**	**90**
IPI00331088.1	Pigment epithelium-derived factor	70.26%	4	4	27	65
IPI00153088.1	PIN2/TERF1-interacting telomerase inhibitor 1	56.33%	1	2	13	33
IPI00317891.7	Pinin	66.48%	2	14	83	348
IPI00308851.3	PI-PLC X domain-containing protein 3	69.16%	3	5	31	109
IPI00109437.1	Pirin	53.45%	2	3	20	127
IPI00461359.2	Piwi-like protein 2	83.63%	1	2	108	439
IPI00556827.1	plasma membrane calcium ATPase 1	74.18%	3	21	136	897
IPI00265299.3	plasma membrane calcium ATPase 3	69.92%	5	15	116	677
IPI00751474.1	plasma membrane calcium ATPase 4 isoform x/e	66.47%	4	1	112	633
IPI00121350.1	Plasmolipin	25.27%	1	1	3	13
IPI00344681.2	Plastin-1	76.51%	2	2	55	186
IPI00118821.2	Platelet-activating factor acetylhydrolase IB subunit beta	54.15%	2	6	15	142
IPI00118819.1	Platelet-activating factor acetylhydrolase IB subunit gamma	66.38%	4	4	17	47
IPI00626385.4	plectin isoform 12alpha	93.82%	24	4	763	8676
IPI01008579.1	plectin isoform 1a	95.31%	23	2	750	8599
IPI00331172.1	Pleiotropic regulator 1	69.59%	3	11	50	223
IPI00405742.6	plexin B2	68.84%	4	21	131	553
IPI00137311.1	Plexin-A1	67.00%	2	2	146	403
IPI00187545.5	Plexin-A4	65.77%	2	4	127	377
IPI00671477.2	plexin-B3	63.77%	1	1	109	429
IPI00473988.1	Pola2 protein	72.69%	3	1	38	108
IPI01023295.1	Polr1c protein	51.01%	6	5	17	65
IPI00120954.2	poly A binding protein, cytoplasmic 2	91.08%	3	1	74	451
IPI00137145.1	poly(A) binding protein, cytoplasmic 3	91.76%	1	1	73	801
IPI00459344.2	Poly(A) RNA polymerase, mitochondrial	76.92%	1	6	52	183
IPI00128904.1	Poly(rC)-binding protein 1	99.16%	1	15	48	985
IPI00118718.1	Poly(rC)-binding protein 4	71.96%	1	2	30	137
IPI00124287.2	Polyadenylate-binding protein 1	99.06%	3	13	90	1519
IPI00396676.1	Polycomb protein Suz12	87.58%	2	6	72	215
IPI00457950.2	Polyglutamine-binding protein 1	52.09%	4	5	14	60
IPI00858215.1	polyhomeotic-like protein 3 isoform 3	36.50%	7	1	34	118
IPI00459273.1	Polymerase (DNA directed), alpha 2, isoform CRA_a	95.56%	3	1	37	126
IPI00229721.3	Polymerase delta-interacting protein 3	93.10%	3	12	62	270
IPI00117689.1	Polymerase I and transcript release factor	82.65%	3	15	47	517
IPI00133719.1	Potassium voltage-gated channel subfamily A member 1	46.46%	1	3	21	53
IPI00129774.1	Potassium voltage-gated channel subfamily A member 2	44.09%	1	3	25	100
IPI00133980.1	Potassium/sodium hyperpolarization-activated cyclic nucleotide-gated chan	47.16%	2	4	46	141
IPI00120546.1	PRA1 family protein 2	35.96%	1	3	8	30
IPI00119024.3	PRA1 family protein 3	28.72%	1	4	9	118
IPI01019264.1	Predicted	88.78%	71	2	28	105
IPI00915480.1	predicted gene, ENSMUSG00000061104	94.77%	4	9	31	207
IPI00990671.1	prefoldin 4 isoform 3	100.00%	4	6	19	82
IPI00132485.2	Prefoldin subunit 1	86.89%	2	6	22	134
IPI00119545.2	Prefoldin subunit 2	90.26%	2	9	24	261
IPI00121851.2	Prefoldin subunit 3	84.69%	2	14	32	245
IPI00480275.1	Prefoldin subunit 5	96.75%	7	6	24	333
IPI00138238.1	Prefoldin subunit 6	99.21%	2	9	24	245

IPI00117687.1	Pre-mRNA branch site protein p14	96.00%	1	5	21	51							0.265	-0.050	-0.004	0.154	-0.032	3	-0.077	-0.037	-0.245	-0.013	-0.347	3													125	14.6	9.38
IPI00120145.1	Pre-mRNA-processing factor 17	70.64%	2	6	44	142							-0.188	0.153	-0.035	-0.203	-0.038	5	0.251	0.100	0.224	0.114	0.198	3													579	65.4	7.06
IPI00121596.4	Pre-mRNA-processing-splicing factor 8	80.21%	8	80	256	1635	-0.025	0.026	-0.006	0.033	-0.074	86	0.058	0.040	0.041	0.010	-0.001	87	0.103	0.052	0.052	-0.041	0.011	115	0.079	-0.051	0.076	-0.025	0.052	56	-0.201	0.026	0.185	0.258	-0.123	5	2335	273.4	8.84
IPI00320636.3	Pre-mRNA-splicing factor 38B	50.92%	2	4	39	185	-0.436	0.116	-0.327	0.085	-0.464	7							-0.466	-0.109	0.564	-1.053	0.864	3													542	63.7	10.54
IPI00469994.2	Pre-mRNA-splicing factor ISY1 homolog	56.49%	1	1	24	55	0.019	0.030	0.179	0.050	-0.120	3																									285	33.0	5.17
IPI00221951.5	Pre-mRNA-splicing factor RBM22	63.33%	1	7	44	159	-0.168	-0.010	0.029	0.004	-0.248	3	-0.142	-0.037	-0.141	0.081	0.019	6	-0.038	-0.028	0.154	0.044	0.200	6													420	46.9	8.54
IPI00316184.1	Pre-mRNA-splicing factor SPF27	76.89%	1	6	32	173	0.081	0.123	0.136	0.065	0.084	13	-0.173	-0.102	-0.189	-0.114	-0.023	9	-0.009	-0.009	-0.025	-0.076	-0.083	4	-0.028	0.034	-0.059	0.036	0.147	13							225	26.1	5.66
IPI00120744.1	Pre-mRNA-splicing factor SYF1	62.46%	2	6	68	221							0.303	0.257	-0.034	0.384	0.332	11																			855	99.9	6.23
IPI00970229.1	pre-mRNA-splicing regulator WTAP isoform a	96.21%	6	7	47	185							0.305	0.108	0.068	0.222	-0.051	6	0.245	0.218	0.318	-0.123	-0.293	4													396	44.2	5.25
IPI00460063.2	Prenylcysteine oxidase	75.05%	2	5	42	187	-0.209	-0.009	-0.228	-0.429	-0.173	9	-0.125	-0.089	-0.020	0.157	0.000	12	1.069	0.111	1.145	-0.006	1.079	4							-0.102	0.080	0.019	-0.149	-0.063	9	505	56.5	6.92
IPI00226726.1	Prenylcysteine oxidase-like	69.29%	1	4	28	76	0.041	0.103	0.065	0.070	0.092	5																									495	54.8	7.65
IPI00944702.1	pre-rRNA-processing protein TSR2 homolog isoform 1	48.21%	2	2	10	62																									-0.083	0.188	0.079	0.395	-0.108	3	195	21.4	4.26
IPI00124015.2	Presenilins-associated rhomboid-like protein, mitochondria	59.42%	3	5	27	102																			-0.175	0.034	0.003	-0.080	-0.139	4							377	41.9	9.92
IPI00110761.3	PRKR-interacting protein 1	76.88%	2	2	18	59	0.077	0.170	0.047	0.030	-0.020	5																									186	21.5	9.42
IPI00416303.1	Probable aminopeptidase NPEPL1	58.21%	2	4	26	83	0.010	-0.103	0.003	0.066	0.026	6	-0.751	-0.014	-0.723	0.023	-0.586	5																			524	55.9	6.84
IPI00653307.1	probable ATP-dependent RNA helicase DDX17 isoform 1	75.46%	4	16	65	598	0.139	0.102	0.073	0.068	0.094	20	-0.122	0.001	-0.123	-0.032	-0.157	56	0.198	0.103	0.203	-0.141	0.095	52	0.024	-0.026	0.324	-0.041	0.203	24	0.011	-0.009	0.080	0.061	-0.012	21	652	72.5	8.68
IPI00122400.8	Probable ATP-dependent RNA helicase DDX20	90.67%	2	8	73	269							-0.219	-0.009	0.171	0.445	0.377	6	0.276	0.397	-0.060	0.299	0.026	4	-0.203	0.038	0.163	0.055	-0.051	5							825	91.7	6.74
IPI00651980.1	probable ATP-dependent RNA helicase DDX4 isoform 1	72.66%	2	1	70	223							0.047	-0.097	0.036	-0.009	0.003	8	0.123	0.082	0.076	-0.031	0.024	10													728	79.2	5.99
IPI00187240.1	Probable ATP-dependent RNA helicase DDX47	86.15%	2	6	44	141							0.073	0.199	-0.364	0.026	0.014	5							-0.138	0.037	0.037	-0.069	0.080	4							455	50.6	9.10
IPI00420363.3	Probable ATP-dependent RNA helicase DDX5	87.30%	4	25	76	1169	0.011	0.083	-0.045	0.061	-0.022	63	-0.019	-0.028	-0.076	-0.022	-0.109	88	0.152	0.061	0.208	-0.074	0.092	101	0.019	-0.059	0.044	-0.034	0.099	71	-0.006	0.100	0.122	0.057	0.026	39	614	69.2	8.92
IPI00336965.2	Probable ATP-dependent RNA helicase DDX52	58.36%	1	2	38	75	-0.293	0.187	-0.155	-0.069	-0.229	3																									598	67.4	9.57
IPI00109932.1	Probable ATP-dependent RNA helicase DDX6	85.09%	3	18	50	517	-0.016	0.019	-0.054	-0.076	-0.029	39	0.047	-0.029	0.089	-0.056	0.047	23	-0.057	-0.073	-0.072	-0.079	0.010	25	0.092	0.040	0.066	0.024	-0.004	24	-0.125	0.045	0.285	0.011	0.050	5	483	54.2	8.66
IPI00351266.2	Probable ATP-dependent RNA helicase DHX36	87.11%	1	13	102	380							-0.069	-0.034	0.128	-0.006	0.079	14							0.107	0.033	0.231	-0.082	0.335	6							1001	113.8	8.41
IPI00226807.3	Probable cationic amino acid transporter	38.13%	2	4	20	55																			-1.127	-0.194	-1.162	0.013	-1.224	6	0.223	0.099	0.041	0.059	-0.063	4	771	83.9	5.35
IPI00109891.2	Probable cation-transporting ATPase 13A1	72.33%	2	8	95	380	-0.063	-0.062	-0.133	-0.048	-0.139	4	-0.134	-0.074	-0.083	0.080	-0.094	6	-0.094	-0.054	-0.074	-0.146	-0.069	6	0.557	0.225	0.620	0.004	0.500	4							1200	132.3	8.03
IPI00132415.1	Probable dimethyladenosine transferase	66.77%	1	3	31	88																			-0.086	-0.183	-0.042	-0.127	-0.127	5							313	35.3	9.99
IPI00465871.1	Probable G-protein coupled receptor 158	61.92%	2	6	89	243																									-0.285	0.011	0.009	-0.351	-0.170	12	1200	134.3	8.09
IPI00116138.1	Probable histidyl-tRNA synthetase, mitochondrial	78.42%	2	2	60	203							-0.111	-0.331	-0.054	-0.111	-0.083	3																			505	56.9	8.32
IPI00330933.6	Probable methylthioribulose-1-phosphate dehydratase	76.76%	1	5	20	92	0.047	-0.279	-0.281	-0.176	0.160	11	-0.474	-0.173	-0.106	-0.299	-0.274	9																			241	26.9	6.90
IPI00831373.1	probable phospholipid-transporting ATPase IA isoform a	57.90%	7	16	94	359																									0.153	-0.271	-0.375	-0.276	-0.243	31	1164	131.3	6.84
IPI00127464.1	Probable phospholipid-transporting ATPase IB	72.39%	2	1	86	255																									-0.014	-0.141	-0.353	-0.306	-0.438	6	1148	129.3	7.33
IPI00111829.1	Probable rRNA-processing protein EBP2	74.51%	2	12	42	293	-0.088	0.034	-0.109	-0.023	-0.060	7	0.064	0.140	-0.025	-0.071	0.037	9	0.045	-0.004	0.077	-0.082	-0.038	30	-0.044	-0.001	-0.069	-0.065	-0.003	13							306	34.7	10.08
IPI00229726.2	Probable threonyl-tRNA synthetase 2, cytoplasmic	63.67%	1	4	69	248	0.043	-0.120	0.023	-0.119	0.070	12	-0.464	0.026	-0.402	-0.025	-0.313	5							0.051	0.072	0.107	0.077	0.141	3							790	91.3	7.53
IPI00112763.1	Probable U3 small nucleolar RNA-associated protein 11	53.75%	1	2	26	55																			-0.178	-0.204	-0.074	-0.106	-0.163	3	-0.076	0.058	0.123	0.222	-0.183	3	253	30.5	10.26
IPI00798468.2	probable ubiquitin carboxyl-terminal hydrolase FAF-X	73.45%	10	36	203	881	-0.035	-0.048	-0.050	0.020	-0.086	17	-0.045	-0.041	-0.041	-0.057	0.044	21	-0.054	-0.163	-0.204	-0.050	-0.178	7	-0.221	0.044	-0.221	-0.013	-0.276	15	-0.102	-0.048	-0.131	-0.180	-0.163	18	2554	290.0	5.83
IPI00118987.2	Pro-cathepsin H	84.38%	2	5	27	94	0.233	-0.066	0.126	-0.063	0.215	12																									333	37.1	8.40
IPI00172251.1	processing of precursor 1 isoform 2	84.04%	2	7	111	374	-0.271	-0.019	-0.236	-0.229	-0.243	5	0.116	-0.105	-0.295	-0.062	-0.146	4	-0.326	-0.030	-0.599	-0.092	-0.576	3													1015	114.0	9.33
IPI00169870.6	Procollagen galactosyltransferase 1	71.80%	1	17	51	226	0.068	-0.038	-0.065	0.010	0.079	13	0.044	-0.006	0.029	-0.009	0.016	22	-0.025	-0.020	0.020	-0.144	-0.054	8	-0.048	-0.001	-0.144	-0.048	-0.103	5							617	71.0	7.28
IPI00127407.1	Procollagen-lysine,2-oxoglutarate 5-dioxygenase 1	76.10%	4	10	58	201							-0.057	-0.017	0.089	0.094	-0.170	26							0.091	-0.026	0.152	-0.063	0.014	6							728	83.5	6.54
IPI00123758.3	procollagen-lysine,2-oxoglutarate 5-dioxygenase 2 isoform 1	83.25%	2	11	67	213							-0.095	0.024	0.161	0.023	0.026	12	-0.109	0.018	0.339	0.123	0.642	4	-0.037	-0.257	0.083	-0.204	0.171	3							758	86.8	6.89
IPI00127406.1	Procollagen-lysine,2-oxoglutarate 5-dioxygenase 3	76.79%	2	16	54	260	-0.176	0.014	-0.080	-0.099	-0.108	8	-0.117	-0.108	-0.181	-0.149	-0.271	16	0.197	0.126	0.353	-0.092	0.144	9	-0.080	-0.037	-0.231	0.011	-0.180	19							741	84.9	6.23
IPI00224740.6	Profilin-1	93.57%	2	11	16	619	-0.103	-0.128	0.033	-0.031	0.001	63	-0.181	-0.083	-0.125	-0.168	0.027	46	-0.272	-0.179	-0.231	0.130	-0.076	66	0.128	0.075	-0.003	0.011	0.108	48	0.051	-0.099	-0.072	-0.150	0.114	41	140	14.9	8.28
IPI00331188.1	Programmed cell death protein 10	68.40%	2	2	20	89							-0.576	-0.517	-0.523	-0.425	-0.371	3																			212	24.7	8.19
IPI00323064.3	Programmed cell death protein 4	66.10%	1	6	48	139	-0.147	-0.157	-0.233	-0.112	0.118	5																									469	51.7	5.16
IPI00116120.4	Programmed cell death protein 5	92.86%	2	8	28	169	-0.038	0.000	0.055	0.038	-0.071	10	-0.260	0.026	-0.186	0.050	-0.147	18	-0.091	-0.105	-0.186	0.085	-0.099	13	-0.134	-0.245	-0.016	-0.228	0.140	12	0.094	0.092	-0.013	0.084	0.193	10	126	14.3	5.68
IPI00121736.1	Programmed cell death protein 6	100.00%	1	6	23	85	-0.417	-0.293	-0.407	0.031	-0.309	3	-0.371	0.103	0.048	-0.017	-0.004	6													0.070	-0.026	0.160	0.040	0.095	5	191	21.9	5.40
IPI00133440.1	Prohibitin	98.16%	4	18	35	491	-0.048	-0.019	-0.074	-0.016	-0.091	34	0.184	-0.092	0.131	-0.114	0.199	20	0.481	0.114	0.383	0.038	0.267	39	0.308	0.009	0.385	-0.032	0.419	39	0.141	-0.013	-0.060	-0.085	-0.053	48	272	29.8	5.76
IPI00321718.4	Prohibitin-2	90.30%	3	12	49	756	-0.054	0.019	0.013	0.048	-0.122	36	0.365	0.077	0.189	0.138	0.171	22	0.381	0.078	0.354	-0.016	0.220	49	0.445	-0.039	0.393	-0.009	0.430	38	0.135	-0.097	-0.016	-0.088	-0.047	38	299	33.3	9.83
IPI00124980.1	Prolactin regulatory element-binding protein	66.67%	2	3	27	45	0.181	0.157	0.009	0.014	0.178	3																									417	45.4	8.76
IPI00113870.1	Proliferating cell nuclear antigen	98.47%	2	4	35	544	-0.065	-0.032	-0.034	-0.082	-0.017	32	-0.016	0.040	0.075	0.031	-0.050	32	-0.020	-0.129	-0.009	0.095	0.107	37	0.594	0.029	0.807	-0.047	0.652	31							261	28.8	4.77
IPI00473170.1	Proliferating cell nuclear antigen	100.00%	1	1	38	454	-0.072	-0.062	-0.050	-0.112	-0.045	28	-0.023	0.064	0.004	0.026	-0.056	19	0.010	-0.126	-0.003	0.088	0.123	30	0.774	0.001	0.778	-0.056	0.723	28							262	29.0	4.81
IPI00119305.3	Proliferation-associated protein 2G4	87.31%	3	26	49	840	-0.069	0.014	-0.059	-0.016	-0.066	59	-0.063	-0.108	-0.100	-0.133	-0.048	66	-0.059	0.035	-0.059	0.026	-0.060	79	-0.044	0.070	0.062	-0.025	0.017	72	-0.130	-0.003	-0.012	-0.080	0.014	22	394	43.7	6.86
IPI00321597.6	Proline-, glutamic acid- and leucine-rich protein 1	54.76%	2	9	53	228	0.023	0.104	-0.082	0.076	-0.014	6							0.188	0.128	0.021	-0.333	-0.022	3	0.175	0.112	0.171	0.038	0.169	4							1123	118.0	4.36
IPI00281761.3	proline-rich transmembrane protein 2	54.62%	1	6	19	84																									0.048	-0.112	-0.016	-0.054	-0.080	17	346	35.9	4.63
IPI00119063.2	Prolow-density lipoprotein receptor-related protein 1	77.01%	3	62	381	1622	0.088	-0.060	-0.031	-0.029	0.128	25	0.294	0.076	0.445	0.149	0.147	75	0.292	0.024	0.193	-0.014	0.157	77							-0.231	-0.114	-0.114	-0.026	0.001	11	4545	504.4	5.36
IPI00929859.1	prolyl 4-hydroxylase subunit alpha-2 isoform 2	78.96%	7	9	46	203							-0.003	0.139	-0.054	0.043	-0.154	13	0.347	-0.119	0.402	-0.097	0.069	9	-0.022	-0.122	-0.035	-0.053	-0.184	16							537	61.0	5.90
IPI00989777.1	Prolyl endopeptidase	81.55%	2	36	63	728	-0.152	-0.106	-0.053	-0.066	-0.077	58	-0.013	0.009	0.001	-0.003	0.033	50	-0.466	-0.157	-0.336	0.153	-0.246	55	0.076	0.065	0.001	0.050	0.026	61	0.079	0.048	0.160	0.001	0.079	16	710	80.7	5.73
IPI00330523.1	Propionyl-CoA carboxylase alpha chain, mitochondrial	72.65%	3	9	68	255	0.072	0.162	0.214	-0.048	0.003	3																			0.089	0.059	-0.047	0.077	0.066	16	724	79.9	7.25
IPI00606510.2	Propionyl-CoA carboxylase beta chain, mitochondrial	74.86%	2	2	55	233	0.026	0.170	0.089	0.076	0.083	3																			0.014	-0.031	0.065	0.024	0.114	22	541	58.4	7.66
IPI00135604.5	ProSAAS	57.75%	1	6	19	138																			-2.047	-0.265	-3.573	-0.103	-1.662	3	0.202	0.059	-0.092	-0.013	0.124	26	258	27.3	5.85
IPI00312174.7	Prostaglandin E synthase 2	97.66%	1	6	48	178	-0.198	0.072	-0.264	0.011	-0.318	3	0.919	0.198	1.020	0.144	0.416	3							-0.333	0.066	-0.315	-0.082	-0.529	3	-0.031	0.085	0.167	0.239	0.085	4	384	43.3	9.00
IPI00127989.1	Prostaglandin E synthase 3	98.75%	3	8	18	237	0.172	0.108	0.290	0.273	0.100	14	-0.390	0.048	-0.063	-0.047	-0.176	30	-0.226	-0.127	-0.209	-0.003	-0.088	21	-0.255	-0.141	-0.079	0.107	-0.191	7	-0.236	0.160	0.132	-0.054	-0.114	11	160	18.7	4.55
IPI00125497.4	Prostaglandin F2 receptor negative regulator	65.64%	1	5	56	123							0.385	0.152	0.548	0.269	0.199	7	-0.175	-0.129	0.062	0.041	-0.065	4													879	98.7	6.61
IPI00128389.3	Prostaglandin G/H synthase 1	70.43%	2	10	38	259	-0.170	0.127	-0.216	0.048	-0.250	18	0.374	0.128	0.375	0.207	0.160	15	0.041	0.010	0.144	-0.117	-0.060	31	0.282	0.006	0.210	-0.009	0.327	9							602	69.0	6.83
IPI00308785.1	Prostaglandin G/H synthase 2	51.66%	2	4	31	96	-0.683	0.011	-0.704	-0.003	-0.615	11							0.092	-0.010	-0.097	-0.295	-0.060	3													604	69.0	7.37
IPI00131887.3	Prostaglandin reductase 1	75.68%	1	3	24	76							-0.243	-0.183	-0.111	-0.317	-0.019	3	-0.496	-0.296	-0.576	0.189	-0.344	5													329	35.5	7.96
IPI00119094.1	Prostamide/prostaglandin F synthase	75.62%	1	2	16	45																									0.379	0.293	0.437	0.220	0.242	3	201	21.7	6.74
IPI00311947.7	Prostate androgen-regulated mucin-like protein 1 homolog	60.14%	1	3	14	103	0.057	0.083	0.209	-0.063	-0.023	12																									296	30.6	5.49
**IPI00331155.4**	**Proteasomal ubiquitin receptor ADRM1**	40.54%	3	5	28	195	0.143	0.100	0.158	0.102	0.041	8	-0.116	-0.013	0.029	-0.105	-0.007	7	-0.264	-0.139	-0.149	-0.017	-0.042	20	-0.226	0.295	-0.092	0.374	-0.013	6	0.144	0.224	0.004	0.225	0.523	5	407	42.0	5.07
IPI00124223.3	Proteasome activator complex subunit 1	94.78%	5	14	36	257	-0.327	-0.060	-0.211	-0.017	-0.158	19	-0.276	-0.176	-0.038	-0.038	-0.128	22							-0.086	0.072	-0.162	0.026	-0.059	29	0.192	-0.026	-0.158	-0.089	0.170	4	249	28.7	5.97
IPI00124225.3	Proteasome activator complex subunit 2	89.96%	2	11	27	151	-0.114	-0.012	-0.199	0.044	-0.074	14	-0.213	0.169	-0.001	0.040	-0.001	15	0.147	0.071	0.151	-0.017	0.065	3	-0.032	0.072	0.037	0.081	0.151	16							239	27.0	5.76
IPI00113660.1	Proteasome activator complex subunit 3	83.86%	3	10	34	179	-0.103	-0.045	-0.099	-0.016	-0.076	20	-0.012	-0.203	-0.031	-0.208	0.141	13	0.030	-0.105	-0.163	0.076	-0.068	6	0.048	0.108	0.034	0.092	-0.082	13							254	29.5	5.95
IPI00676631.1	Proteasome assembly chaperone 4	61.79%	2	3	5	10	-0.034	-0.029	0.048	-0.083	0.057	5																									123	14.0	6.15
IPI00420745.7	Proteasome subunit alpha type	78.95%	2	10	25	514	0.051	0.003	0.119	0.084	0.140	25	0.134	-0.041	-0.053	-0.186	0.092	14	-0.219	-0.044	-0.146	-0.076	-0.053	14	0.079	0.249	0.147	0.085	0.193	41	0.119	0.024	-0.029	-0.063	0.041	18	247	27.5	8.22
IPI00283862.6	Proteasome subunit alpha type-1	100.00%	2	20	43	562	0.021	-0.019	0.084	-0.012	-0.022	42	0.120	-0.004	0.234	0.021	0.038	32	-0.085	-0.056	-0.103	0.023	-0.066	40	0.017	-0.028	0.034	-0.022	0.050	48	-0.051	0.044	-0.029	0.026	-0.047	35	263	29.5	6.46
IPI00331644.5	Proteasome subunit alpha type-3	68.24%	1	15	28	316	0.116	-0.044	0.143	-0.053	0.171	13	0.124	-0.023	0.085	0.010	0.092	10	-0.155	-0.168	-0.173	-0.123	-0.080	16	0.076	0.064	0.152	0.041	0.152	19	-0.080	-0.105	-0.009	0.034	-0.037	10	255	28.4	5.44
IPI00277001.4	Proteasome subunit alpha type-4	93.49%	3	12	37	267	0.019	0.023	0.066	-0.016	-0.012	22	-0.009	-0.028	0.192	0.001	0.156	11	-0.050	-0.082	-0.020	0.091	0.029	10	0.088	0.107	0.048	-0.013	0.001	8	-0.023	-0.139	-0.112	-0.099	-0.122	9	261	29.5	7.72
IPI00131407.1	Proteasome subunit alpha type-5	73.86%	2	11	24	375	0.118	0.011	0.188	0.069	0.119	12	-0.292	-0.071	-0.360	-0.171	-0.241	20	-0.163	-0.084	-0.103	0.079	-0.026	22	-0.009	0.108	0.048	0.024	0.080	23	-0.026	-0.012	-0.054	0.024	-0.089	10	241	26.4	4.79
IPI00131845.1	Proteasome subunit alpha type-6	79.67%	3	13	26	240	0.102	-0.019	0.099	0.069	0.127	12	-0.063	-0.099	-0.028	-0.057	0.030	29	-0.245	-0.091	-0.216	-0.042	-0.097	22	-0.194	-0.031	-0.050	-0.050	-0.133	7	0.026	-0.114	-0.050	-0.083	-0.108	27	246	27.4	6.76
IPI00131406.1	Proteasome subunit alpha type-7	94.76%	4	15	38	371	-0.136	-0.168	0.069	0.026	-0.035	26	0.079	-0.019	0.144	0.058	0.163	15	-0.218	-0.200	-0.125	0.136	-0.065	18	0.098	0.103	0.024	0.033	0.006	36	0.203	0.066	0.044	-0.022	0.007	17	248	27.8	8.46
IPI00113845.1	Proteasome subunit beta type-1	90.83%	1	5	29	262	0.114	-0.007	0.143	0.057	0.207	11	0.066	0.031	0.013	0.007	0.305	18	-0.065	-0.026	0.061	0.077	-0.216	6	-0.313	-0.228	-0.188	-0.142	-0.003	16	-0.063	-0.086	0.031	0.188	-0.048	21	240	26.4	7.81
IPI00316736.1	Proteasome subunit beta type-10	77.29%	1	2	20	53							-0.353	-0.271	-0.211	0.194	0.055	4																			273	29.0	6.87
IPI00128945.1	Proteasome subunit beta type-2	77.11%	1	8	23	193	-0.099	-0.010	-0.065	0.013	-0.056	16	-0.016	-0.020	-0.051	0.075	0.004	5	-0.213	-0.159	-0.186	-0.142	0.021	6	0.075	-0.006	-0.032	-0.013	-0.088	19	-0.069	-0.050	-0.091	-0.133	-0.117	7	201	22.9	7.02
IPI00314467.3	Proteasome subunit beta type-3	68.78%	5	7	26	201	-0.029	-0.184	-0.204	-0.071	-0.149	8							-0.306	-0.232	-0.188	0.194	-0.048	9	0.107	-0.031	0.065	-0.112	0.103	14	0.135	0.153	0.027	0.100	0.233	7	205	22.9	6.55
IPI00129512.3	Proteasome subunit beta type-4	95.83%	1	8	22	400	0.180	-0.086	0.043	-0.039	0.167	35	-0.125	-0.091	0.016	-0.214	0.057	12	-0.120	0.006	-0.060	0.041	-0.114	21	0.007	0.050	-0.050	-0.016	0.153	27	-0.016	-0.020	-0.060	-0.213	-0.025	33	264	29.1	5.64
IPI00317902.3	Proteasome subunit beta type-5	79.55%	3	12	29	208	0.087	-0.042	0.070	0.004	0.004	20	0.066	-0.079	0.343	-0.265	0.205	11	-0.050	-0.021	-0.059	0.064	-0.085	17	-0.099	-0.206	-0.092	-0.134	0.013	9	-0.142	-0.150	-0.088	-0.080	0.044	12	264	28.5	7.02
IPI00119239.2	Proteasome subunit beta type-6	100.00%	1	7	22	217	0.102	-0.016	0.103	0.006	0.068	16	0.140	0.038	0.057	-0.114	0.163	16	-0.221	-0.090	-0.138	0.085	-0.077	16	0.057	0.083	0.103	0.013	0.157	13	0.038	0.038	-0.006	0.077	0.081	11	238	25.4	5.11
IPI00136483.1	Proteasome subunit beta type-7	93.14%	1	10	28	211	0.041	-0.048	-0.065	0.029	-0.054	16	0.110	-0.054	0.198	-0.076	0.006	17	-0.176	-0.081	-0.109	0.052	0.009	7	-0.097	0.073	0.020	-0.004	0.026	13	0.000	-0.050	-0.004	-0.054	-0.014	11	277	29.9	7.99
IPI00116712.1	Proteasome subunit beta type-8	95.29%	2	5	30	127							-0.531	0.041	-0.324	0.198	-0.318	11																			276	30.2	6.68
IPI01026793.1	Protein	81.09%	4	8	49	251	-0.095	0.094	-0.208	0.100	-0.117	8	0.448	0.151	0.405	0.277	0.345	7							0.076	0.062	0.124	0.011	0.174	40							439	48.4	7.01
IPI01027090.1	Protein	77.13%	3	5	20	86	0.052	-0.035	-0.025	-0.128	0.034	6	-0.221	0.007	-0.074	0.165	-0.120	4																			223	24.6	6.02
IPI01027185.1	Protein	81.54%	2	4	10	179																			-0.016	-0.022	-0.031	0.001	-0.154	5							130	14.2	5.81
IPI00118763.2	Protein AF-10	38.30%	3	3	41	90							-0.088	0.054	0.094	0.127	-0.318	3																			1068	113.0	8.19
IPI00229845.3	Protein arginine N-methyltransferase 5	80.38%	2	10	56	287	-0.057	0.036	0.026	-0.013	-0.105	17	0.114	-0.034	0.020	0.218	-0.083	9							-0.007	0.092	0.009	0.033	0.098	21							637	72.6	6.42
IPI00420678.2	Protein arginine N-methyltransferase 8	72.84%	1	1	27	157	-0.458	-0.028	-0.525	-0.029	-0.409	10	-0.066	0.045	-0.059	0.140	-0.368	3	-0.127	-0.047	-0.226	0.095	-0.099	8	0.267	0.099	0.110	0.004	-0.149	9	0.066	0.006	0.247	0.360	0.332	5	394	45.2	6.93
IPI00127176.3	Protein BRICK1	89.33%	1	5	12	53							-0.599	-0.178	-0.231	-0.144	-0.138	6													-0.032	-0.199	-0.160	0.131	-0.060	10	75	8.8	5.45
IPI00849741.1	protein BUD31 homolog isoform 2	77.78%	3	8	18	52	0.098	0.047	0.116	0.062	0.251	4	0.057	0.112	-0.051	0.087	0.208	6																			144	16.9	8.51
IPI00132184.1	Protein C17orf37 homolog	49.57%	1	3	6	24							-0.013	0.054	-0.031	-0.304	0.066	4													-0.125	-0.327	-0.060	0.190	0.166	6	115	12.3	4.51
IPI00110487.1	Protein C20orf11 homolog	89.04%	1	6	25	66																			-0.125	0.011	-0.272	-0.141	-0.035	5							228	26.8	4.97
IPI00135512.1	Protein canopy homolog 2	58.24%	1	7	15	104	-0.100	0.062	0.066	0.095	-0.001	4							0.050	0.126	-0.134	0.054	0.139	6	-0.099	-0.188	-0.114	-0.012	-0.204	9							182	20.8	5.07
IPI00875961.1	Protein canopy homolog 4	53.06%	2	5	21	74							0.076	-0.068	0.127	0.048	0.026	9																			245	28.1	4.77
IPI00226564.3	Protein CIP2A	77.84%	1	6	109	299	-0.019	0.194	-0.039	0.030	-0.079	7	0.596	0.080	0.346	-0.037	0.301	7																			907	102.0	6.28
IPI00119814.3	Protein CWC15 homolog	84.28%	1	6	25	244	0.108	0.111	0.171	0.016	0.080	11	-0.355	-0.051	-0.329	-0.260	-0.189	6	-0.199	0.041	-0.029	-0.176	0.069	5	0.038	0.132	0.242	0.115	-0.031	19							229	26.6	5.71
IPI00113117.1	Protein CYR61	87.60%	1	3	39	84							0.010	-0.138	-0.671	0.089	-0.057	4																			379	41.7	8.12
IPI00608021.1	Protein DDI1 homolog 2	82.21%	1	6	36	180																			-0.165	-0.020	-0.165	-0.065	-0.175	14							399	44.6	5.05
IPI00227720.3	Protein DEK	84.47%	4	12	51	282	0.143	-0.082	0.199	-0.016	0.157	22	0.095	0.065	-0.041	0.069	-0.001	18	0.429	0.020	0.228	-0.028	0.116	9	-0.116	-0.128	0.184	-0.029	0.240	19							380	43.1	6.86
IPI00808370.2	Protein diaphanous homolog 1	66.61%	4	17	107	356	0.190	-0.012	0.095	0.050	0.132	30	0.197	0.064	0.241	0.128	0.355	5																			1255	139.3	5.57
IPI00133522.2	Protein disulfide-isomerase	92.53%	2	42	73	1457	0.273	0.073	0.249	0.041	0.162	129	0.661	0.141	0.600	0.126	0.473	122	0.329	0.073	0.245	0.034	0.139	106	-0.189	-0.060	-0.224	-0.017	-0.178	139	-0.050	0.006	-0.013	-0.062	-0.112	53	509	57.0	4.88
IPI00230108.6	Protein disulfide-isomerase A3	89.31%	2	40	74	1465	0.020	0.026	0.031	0.017	-0.017	135	0.200	0.065	0.181	0.045	0.081	110	0.299	0.076	0.255	0.031	0.175	131	0.084	-0.012	0.029	-0.004	0.070	142	0.027	-0.051	-0.026	-0.127	-0.076	67	505	56.6	6.21
IPI00271951.5	protein disulfide-isomerase A4	75.20%	1	41	74	1095	0.111	0.024	0.139	0.007	0.079	77	0.100	0.014	0.110	0.079	-0.009	98	0.532	0.050	0.479	-0.035	0.384	67	-0.004	-0.062	-0.111	-0.031	-0.006	108	-0.117	-0.109	-0.131	-0.095	-0.147	19	641	72.3	5.22
IPI00986015.1	protein disulfide-isomerase A6-like	80.32%	2	1	53	669	-0.102	0.064	-0.100	0.017	-0.068	50	-0.059	-0.009	-0.133	0.061	-0.149	43	0.450	0.102	0.390	-0.010	0.288	31	0.163	-0.108	-0.006	-0.047	0.165	78	-0.234	-0.072	0.055	-0.094	-0.057	15	432	47.2	6.71
IPI00453798.3	Protein disulfide-isomerase TMX3	84.87%	1	13	39	264	0.136	0.066	0.043	-0.025	0.153	11	0.341	-0.059	0.349	0.050	0.269	20	0.574	0.089	0.549	-0.014	0.314	8	-0.535	-0.133	-0.531	-0.035	-0.573	10	0.135	0.108	0.132	0.153	-0.004	3	456	51.8	5.16
IPI00117168.3	Protein dpy-30 homolog	97.98%	1	3	9	60	0.050	-0.012	0.148	0.069	0.050	6	-0.047	-0.037	-0.167	0.108	-0.139	12	0.072	0.042	0.273	0.110	0.041	5													99	11.2	4.88
IPI00169684.2	protein ECT2 isoform 1	86.20%	3	6	92	283	-0.394	0.084	-0.383	0.051	-0.360	4	0.445	-0.010	0.107	0.124	0.065	3																			913	103.1	7.44
IPI00122594.4	Protein ELYS	70.49%	1	10	185	658							-0.279	0.040	-0.241	0.019	-0.173	11																			2243	247.5	6.55
IPI00113386.1	Protein ETHE1, mitochondrial	90.55%	1	5	24	84							-0.117	-0.109	-0.010	-0.020	-0.138	4	0.096	0.048	-0.120	0.244	-0.063	9													254	27.7	7.23
IPI00133338.1	Protein FAM107B	73.28%	1	3	13	112	-0.271	-0.236	-0.071	-0.154	-0.131	10							-0.300	-0.350	-0.014	0.282	-0.163	3													131	15.6	8.31
IPI00134994.3	Protein FAM111A	66.88%	3	2	48	130							0.077	-0.333	0.110	-0.285	-0.072	3																			613	69.9	8.44
IPI00133411.1	Protein FAM136A	100.00%	5	1	23	52																									0.167	-0.357	-0.123	-0.142	-0.236	3	138	15.7	7.61
IPI00109611.1	Protein FAM162A	97.42%	1	6	31	140	-0.739	-0.044	-0.644	-0.001	-0.706	6	-0.304	-0.039	0.044	-0.077	0.091	7	0.118	0.099	-0.003	-0.034	-0.016	11	-0.170	-0.041	0.061	0.079	-0.047	3	0.103	0.087	0.179	0.037	0.197	8	155	17.7	9.88
IPI00808550.2	Protein FAM169A	46.17%	1	3	26	84																			-2.211	-0.063	-2.293	-0.054	-2.184	5							665	73.2	4.68
IPI00224068.1	Protein FAM177A1	76.33%	1	8	18	60							-0.007	-0.221	-0.167	-0.184	0.166	7	0.250	-0.089	0.189	-0.149	0.144	3													207	23.6	4.59
IPI00331260.1	Protein FAM192A	54.33%	1	3	22	69																			-0.171	-0.250	-0.324	-0.109	-0.175	7							254	28.7	5.14
IPI00114944.3	Protein FAM195B	97.94%	1	4	14	44							0.364	-0.097	0.360	0.038	0.151	3																			97	11.1	9.14
IPI00222044.1	Protein FAM49A	78.95%	2	5	25	74																									-0.044	0.157	0.106	0.083	0.104	11	323	37.3	6.01
IPI00122015.1	Protein FAM49B	87.35%	1	14	36	383	-0.057	0.023	0.003	0.023	0.058	27	-0.236	-0.031	-0.123	-0.009	0.003	4	-0.405	-0.112	-0.241	0.059	-0.085	8	0.194	0.120	0.145	0.141	0.268	15	0.029	0.084	0.114	0.163	0.075	38	324	36.8	6.06
IPI00322922.4	Protein FAM50A	58.11%	1	4	29	93							-0.300	-0.119	-0.072	-0.122	0.188	4	-0.023	0.019	0.138	0.089	0.014	6													339	40.2	6.83
IPI00377732.3	Protein FAM91A1	66.79%	1	5	66	244	-0.009	0.139	-0.057	0.089	-0.079	10																									837	93.4	6.35
IPI00457756.2	Protein FAM98A	84.47%	1	5	45	221	-0.014	-0.240	-0.068	-0.203	-0.211	3	0.417	-0.162	0.736	0.124	0.079	6	0.207	0.037	0.152	-0.396	0.275	6	0.122	-0.221	-0.020	-0.010	0.202	5							515	55.0	8.95
IPI00465946.1	Protein FAM98B	84.15%	1	6	46	178	-0.163	-0.142	-0.193	-0.206	-0.076	10													0.091	-0.091	0.172	0.023	0.165	5							429	45.3	8.50
IPI00118904.1	Protein farnesyltransferase/geranylgeranyltransferase type-1 subunit alpha	53.58%	1	5	25	111	-0.252	0.006	-0.327	-0.103	-0.320	3	-0.421	-0.338	-0.405	0.081	-0.106	6							-0.056	0.198	-0.112	0.094	-0.044	6							377	44.0	4.93
IPI00467104.2	Protein flightless-1 homolog	75.61%	1	21	123	584	0.148	0.026	0.141	0.066	0.172	19	-0.013	0.052	0.094	-0.068	-0.023	19	-0.146	-0.077	-0.223	-0.057	-0.168	30													1271	144.7	6.06
IPI00311968.1	Protein FRG1	61.63%	1	5	19	44							-0.304	-0.496	0.058	0.104	-0.486	4							-0.007	-0.165	-0.012	-0.068	0.052	3							258	29.1	8.92
IPI00153798.1	Protein HEXIM1	55.90%	1	4	19	88	0.027	-0.045	0.352	0.047	0.096	7																									356	40.2	5.38
IPI00319956.1	Protein IMPACT	90.25%	1	7	32	128	-0.521	0.019	-0.423	-0.134	-0.147	3	-0.554	-0.189	-0.434	-0.178	-0.523	9													0.167	0.273	0.130	0.118	0.037	9	318	36.3	5.05
IPI00123624.8	Protein KIAA1967 homolog	63.99%	1	14	71	226	-0.056	0.089	0.016	-0.023	-0.085	12							0.116	-0.027	0.209	0.190	0.234	3	-0.496	-0.279	-0.373	-0.045	-0.250	7	0.203	0.295	0.075	-0.191	0.282	6	922	102.9	5.25
IPI00884460.1	protein kinase C alpha type	66.22%	2	7	53	203	-0.029	-0.142	-0.014	-0.056	-0.186	5																			0.011	0.154	0.130	0.084	0.094	43	672	76.8	7.05
IPI00123613.1	Protein kinase C and casein kinase substrate in neurons protein 1	80.73%	2	27	55	351													-0.537	-0.190	-0.515	-0.019	-0.417	3							0.024	-0.019	0.009	-0.079	-0.041	121	441	50.5	5.24
IPI00125880.1	Protein kinase C and casein kinase substrate in neurons protein 2	78.19%	1	19	51	506	0.303	-0.031	0.264	-0.041	0.228	22	0.393	-0.010	0.331	-0.092	0.328	23	-0.635	-0.138	-0.531	0.058	-0.519	58	0.209	0.003	0.099	-0.091	0.208	16	-0.139	-0.007	-0.054	-0.086	-0.163	23	486	55.8	5.20
IPI00108978.4	Protein kinase C binding protein 1	75.14%	7	6	112	470	0.040	-0.201	0.156	-0.221	0.122	3																									1255	139.1	7.12
IPI00126939.1	Protein kinase C delta-binding protein	86.15%	1	5	25	94							0.452	0.004	0.762	0.241	0.422	12																			260	27.8	5.57
IPI00130419.1	Protein kinase C epsilon type	70.96%	1	11	75	229																									-0.020	-0.160	-0.308	-0.141	-0.214	22	737	83.5	7.03
IPI00122069.1	Protein kinase C gamma type	74.03%	3	17	52	199																									-0.003	0.116	0.179	0.140	0.094	55	697	78.3	7.46
IPI00121084.4	Protein kinase C iota type	62.18%	2	4	33	71																															595	68.2	5.85
IPI00311761.3	Protein KRI1 homolog	43.66%	1	8	47	125	-0.234	0.118	-0.147	0.043	-0.109	3																									710	82.6	5.12
IPI00344142.4	Protein lin-7 homolog A	89.27%	3	2	25	103																			0.037	-0.029	-0.189	-0.047	0.037	5	0.048	0.094	0.027	0.030	-0.051	12	233	26.0	8.72
IPI00136496.1	Protein lin-7 homolog B	82.13%	2	1	24	72																			0.069	0.116	0.241	-0.047	0.057	3	0.103	0.245	0.202	0.283	0.043	7	207	22.9	8.68
IPI00136498.1	Protein lin-7 homolog C	81.22%	3	3	24	125	-0.189	0.052	-0.149	-0.050	-0.221	6													-0.094	0.116	-0.189	0.058	-0.141	7	0.103	0.209	0.157	0.244	0.027	9	197	21.8	8.43
IPI00132973.1	Protein LSM12 homolog	74.87%	2	5	24	152	-0.123	-0.106	-0.063	0.114	-0.163	16	0.210	-0.102	0.258	0.031	0.317	7	0.119	0.032	0.059	0.029	-0.146	8													195	21.7	7.74
IPI00172202.1	Protein LSM14 homolog A	67.97%	2	4	48	273							-0.116	0.037	-0.106	-0.054	-0.199	14																			462	50.5	9.52
IPI00469947.2	Protein lunapark	73.88%	3	5	32	145							0.198	-0.224	0.247	0.217	0.153	12	0.081	0.019	0.132	-0.360	0.126	5	-0.160	0.167	-0.123	-0.077	-0.114	3							425	47.5	5.27
IPI00330599.1	Protein LYRIC	89.64%	7	24	72	664	0.009	0.057	0.050	0.044	-0.105	49	0.225	0.062	0.103	0.072	0.141	26	0.016	0.077	0.026	-0.039	-0.068	44	0.040	0.044	-0.041	0.111	-0.001	13	-0.125	-0.260	-0.415	-0.377	-0.355	10	579	63.8	9.33
IPI00173156.1	Protein LZIC	82.63%	1	5	21	123	-0.109	-0.119	-0.111	0.043	-0.082	5	-0.368	0.004	-0.419	-0.272	-0.069	5							-0.764	0.128	-0.813	0.073	-0.783	4							190	21.5	4.96
IPI00551412.1	Protein mago nashi homolog	83.56%	5	6	20	92	0.283	-0.045	0.103	-0.060	0.054	8	-0.108	0.070	-0.292	-0.034	-0.094	6	-0.336	-0.155	-0.229	-0.088	-0.057	4	0.030	0.052	0.069	0.076	-0.057	3							146	17.2	6.11
IPI01008209.1	protein misato homolog 1	65.47%	4	8	42	100							-0.329	-0.042	-0.468	-0.053	-0.216	9																			556	61.2	6.46
IPI00223886.2	Protein monoglycylase TTLL8	68.63%	2	2	67	179							0.087	0.138	0.491	0.202	0.124	3																			832	94.9	8.98
IPI00125960.1	Protein NDRG1	74.37%	6	10	31	250	0.268	0.106	0.527	0.192	0.502	10	0.427	0.143	0.457	0.241	0.120	21													0.158	0.373	0.377	0.253	0.295	27	394	43.0	6.10
IPI00136107.1	Protein NDRG3	49.87%	3	7	18	86																									-0.034	0.081	0.037	0.091	0.043	11	375	41.5	5.25
IPI00113389.5	Protein Niban	64.47%	4	26	59	493	0.466	-0.003	0.688	0.034	0.729	19	0.238	0.033	0.422	0.123	0.244	35							-0.510	0.064	-0.513	-0.029	-0.415	52							926	102.6	4.78
IPI00115824.1	Protein NipSnap homolog 1	76.06%	3	5	31	112																									0.048	-0.060	0.021	-0.109	-0.089	18	284	33.3	9.44
IPI00115827.1	Protein NipSnap homolog 2	83.27%	2	9	26	100																									-0.082	0.027	0.044	0.026	0.021	24	281	32.9	9.26
IPI00132444.1	Protein NipSnap homolog 3A	47.77%	2	3	15	34																									-0.240	-0.158	-0.299	-0.131	-0.147	4	247	28.3	9.48
IPI00331605.5	Protein Noxp20	57.29%	2	9	43	168							-0.226	-0.045	-0.147	0.043	-0.150	20																			569	61.0	4.56
IPI00457959.3	Protein pelota homolog	74.55%	1	6	31	84																			-0.749	-0.246	-0.016	-0.142	0.371	4							385	43.3	5.99
IPI00623545.2	Protein phosphatase 1 regulatory subunit 14B	80.27%	1	3	12	28	-0.304	-0.025	-0.211	-0.271	-0.023	8													0.444	0.328	0.157	0.224	0.072	3							147	15.9	4.86
IPI00129319.3	Protein phosphatase 1 regulatory subunit 7	88.37%	2	19	51	381	-0.019	-0.025	-0.123	0.096	-0.056	18	-0.383	-0.342	-0.226	-0.198	-0.293	8	-0.304	-0.118	0.347	0.198	-0.152	4	-0.112	-0.199	-0.170	-0.088	-0.231	19	-0.022	0.102	0.027	0.050	0.058	44	361	41.3	4.92
IPI00114802.1	Protein phosphatase 1A	54.45%	3	4	27	102																									-0.007	-0.029	-0.001	0.059	0.124	12	382	42.4	5.36
IPI00118736.3	protein phosphatase 1B isoform 1	72.96%	8	3	41	193	0.000	-0.077	-0.150	-0.188	-0.062	5																									477	52.1	5.11
IPI00461286.1	Protein phosphatase 1E	49.40%	1	5	51	195																									0.041	-0.056	-0.037	-0.138	0.009	12	749	83.4	4.97
IPI00117072.1	Protein phosphatase 1G	62.18%	1	14	43	276	0.001	0.268	0.103	0.114	0.152	11	-0.267	0.011	-0.188	0.059	-0.245	29	-0.053	0.040	0.010	0.188	0.095	9	0.392	0.064	0.484	0.023	0.346	10							542	58.7	4.39
IPI00380331.1	Protein phosphatase 2A B56 delta subunit	81.68%	2	14	68	371	-0.007	-0.012	0.069	0.026	0.083	20	-0.209	0.033	-0.245	-0.108	-0.199	28	-0.500	-0.228	-0.265	-0.133	-0.072	10	0.421	-0.060	0.420	-0.013	0.380	4	0.033	-0.079	-0.139	-0.022	-0.109	18	595	69.1	7.96
IPI00986790.1	protein phosphatase inhibitor 2-like	75.73%	3	1	20	227	0.089	-0.019	-0.038	0.017	0.223	11																									206	23.2	4.75
IPI00415908.4	Protein phosphatase methylesterase 1	77.98%	1	13	38	232	-0.133	-0.203	-0.383	-0.031	-0.189	6	-0.535	-0.039	-0.329	-0.240	-0.255	13							0.138	0.236	0.181	0.081	-0.041	13	-0.065	-0.116	-0.122	-0.178	-0.127	25	386	42.2	5.97
IPI00130462.2	Protein PRRC1	63.43%	1	11	26	141							0.019	-0.056	0.076	0.021	0.139	16	0.091	-0.095	0.080	-0.016	0.228	6	-0.229	-0.076	-0.194	-0.032	-0.260	3							443	46.3	5.95
IPI00380722.1	Protein PRRC2A	70.62%	2	18	165	619	-0.218	0.023	-0.194	-0.023	-0.108	8	-0.189	-0.211	-0.264	-0.130	-0.456	14	-0.062	0.113	-0.231	-0.019	0.001	16	0.072	0.047	0.128	0.058	0.103	14							2158	229.1	9.39
IPI00754134.3	protein PRRC2B isoform 1	69.78%	5	7	184	465	-0.456	0.094	0.405	0.273	0.443	5													0.092	0.153	-0.102	0.224	-0.157	3							2230	243.0	8.47
IPI00222509.1	Protein RCC2	89.23%	2	26	56	664	0.019	0.007	0.038	-0.051	-0.012	32	-0.091	0.062	-0.019	0.081	-0.102	33	-0.336	-0.144	-0.317	-0.006	-0.095	43	0.280	0.037	0.451	-0.007	0.460	88							520	55.9	8.72
IPI00130277.2	Protein Red	77.38%	1	15	57	259	-0.131	0.030	-0.044	-0.051	-0.034	14	-0.216	-0.063	-0.188	-0.198	-0.106	17	0.170	0.060	0.165	-0.076	0.176	10	-0.062	-0.176	0.144	-0.050	0.312	5							557	65.6	6.64
IPI00133119.1	Protein RER1	50.00%	1	4	9	49	0.009	0.088	-0.442	0.251	-0.203	3	0.522	0.148	0.409	0.104	0.482	11	0.122	0.061	0.123	-0.066	-0.116	9													196	23.0	9.51
IPI00551454.3	Protein RRP5 homolog	74.92%	1	28	183	762	-0.095	0.024	-0.068	0.040	-0.183	41	0.122	0.037	0.057	0.118	0.209	19	0.285	0.176	0.234	-0.056	0.044	17	0.239	0.156	0.210	0.069	0.061	31							1862	207.6	8.75
IPI00126175.2	protein S100-A1	91.49%	2	3	8	53	0.123	-0.094	0.179	0.031	0.088	9																			-0.017	0.054	0.261	0.379	0.339	12	94	10.5	4.59
IPI00222555.5	Protein S100-A10	83.51%	1	4	9	163	0.176	0.026	0.337	0.076	0.322	16	0.116	-0.037	0.134	0.036	0.185	11	0.303	0.198	0.218	0.052	0.019	32													97	11.2	6.77
IPI00987238.1	protein S100-A11-like	88.54%	3	3	10	178	0.051	-0.224	0.256	-0.105	0.476	15	-0.573	0.021	-0.561	-0.186	-0.264	16	-0.184	-0.109	-0.016	0.148	-0.120	27													96	10.8	5.14
IPI00988603.1	protein S100-A11-like	93.88%	2	1	12	97	0.048	-0.224	0.081	-0.023	0.310	7							-0.183	-0.095	-0.059	0.147	-0.130	14													98	10.9	5.45
IPI00124115.1	Protein S100-A13	69.39%	1	4	13	86	0.178	0.014	0.099	-0.082	0.073	14	-0.364	-0.130	-0.392	-0.165	-0.186	8	-0.267	-0.052	-0.020	-0.288	-0.130	4													98	11.2	6.13
IPI00124096.1	Protein S100-A4	95.05%	1	5	12	101	-0.226	-0.184	-0.112	-0.063	0.030	12	-0.713	0.004	-0.651	-0.181	-0.392	10	-0.458	-0.158	-0.186	0.017	-0.203	12													101	11.7	5.31
IPI00121427.1	Protein S100-A6	95.51%	1	9	11	138	0.296	-0.272	-0.112	-0.173	0.374	16	-0.563	0.009	-0.550	-0.229	-0.288	3	-0.529	-0.081	-0.255	0.238	-0.211	7	0.261	0.006	0.715	-0.026	0.346	3							89	10.0	5.48
IPI00222557.5	Protein S100-B	44.57%	1	1	3	8																									0.178	-0.139	0.473	0.475	0.385	5	92	10.7	4.55
IPI00405108.1	Protein SCO2 homolog, mitochondrial	67.06%	1	5	22	60	-0.086	0.017	-0.158	0.020	-0.074	6																									255	28.9	8.29
IPI00133920.3	Protein SEC13 homolog	75.16%	1	9	20	114							-0.083	-0.079	-0.114	-0.089	0.079	20	0.267	-0.136	-0.088	0.112	0.027	3													322	35.5	5.38
IPI00380894.4	Protein shisa-6 homolog	75.62%	1	1	48	175																									-0.056	0.192	0.256	0.223	0.119	3	525	58.4	9.48
IPI00330780.1	Protein Smaug homolog 2	73.07%	1	4	43	91							0.011	-0.029	-0.245	0.153	0.047	3																			687	75.0	6.74
IPI00123349.2	Protein transport protein Sec23A	89.67%	2	11	70	446	0.115	0.020	-0.044	0.180	-0.147	12	0.126	-0.112	0.006	-0.178	0.031	26	0.013	-0.085	-0.012	0.065	0.073	6	0.007	-0.080	-0.147	-0.016	-0.103	19	0.141	0.070	-0.001	-0.014	0.013	7	765	86.1	7.08
IPI00317604.1	Protein transport protein Sec23B	86.18%	3	10	74	464	-0.063	0.014	0.017	-0.013	-0.108	19	-0.208	-0.007	-0.116	-0.042	-0.063	16	-0.478	-0.146	-0.255	-0.038	-0.191	13	-0.100	-0.069	-0.044	-0.022	-0.111	26							767	86.4	6.96
IPI00226882.7	Protein transport protein Sec61 subunit alpha isoform 1	57.56%	2	6	23	290	-0.206	-0.010	-0.300	-0.041	-0.209	24	0.259	0.131	0.126	0.048	0.210	19	0.189	0.128	0.194	0.047	0.122	21	0.099	0.070	0.070	0.070	0.127	21							476	52.2	8.06
IPI00227631.5	Protein transport protein Sec61 subunit alpha isoform 2	51.47%	2	2	21	180	-0.186	0.031	-0.329	-0.066	-0.385	13	0.315	0.169	0.166	0.100	0.231	9	0.139	0.098	0.103	0.024	0.061	10	0.095	0.171	0.070	0.033	0.127	15							476	52.2	8.06
IPI00133030.3	Protein transport protein Sec61 subunit beta	98.96%	3	6	14	114	-0.083	-0.014	-0.168	-0.023	-0.094	8	0.404	0.030	0.054	0.050	-0.048	6	0.514	0.154	0.024	-0.072	0.037	11	-0.012	-0.383	-0.004	-0.013	-0.362	3							96	10.0	11.56
IPI00405121.7	Protein unc-13 homolog A	68.81%	2	6	125	368																									0.088	-0.105	0.100	-0.004	-0.233	4	1712	193.7	5.25
IPI00309724.6	Protein unc-45 homolog A	77.44%	3	10	92	380	-0.060	-0.048	0.135	-0.032	-0.028	11	0.001	-0.293	-0.086	-0.535	-0.295	5																			944	103.4	6.34
IPI00330619.3	Protein VAC14 homolog	80.43%	2	6	67	238																			-0.226	-0.079	-0.165	-0.173	-0.262	4							782	88.0	6.13
IPI00121100.4	Protein WWC2	70.35%	1	5	101	260																			-0.513	-0.069	-0.144	-0.155	-0.366	4							1187	132.5	5.71
IPI00122491.2	Protein YIPF3	70.61%	1	2	21	83	0.033	-0.125	-0.026	0.104	-0.076	4																									347	38.0	5.94
IPI00989281.1	Protein zwilch homolog	79.46%	3	6	47	147							-0.224	-0.045	-0.480	0.112	0.184	4																			589	66.8	6.02
IPI00123014.2	Protein-arginine deiminase type-2	64.04%	1	6	51	126																									-0.231	0.061	0.094	0.034	0.107	9	673	76.2	5.53
IPI00126861.3	Protein-glutamine gamma-glutamyltransferase 2	70.85%	2	12	50	197	0.080	-0.074	0.228	-0.051	0.119	29	-1.404	-0.125	-1.300	-0.255	-1.262	5																			686	77.0	5.10
**IPI00970652.1**	**protein-L-isoaspartate(D-aspartate) O-methyltransferase**	86.32%	5	11	33	230							-0.128	0.064	-0.267	0.034	-0.083	5	-0.260	-0.047	-0.065	-0.097	-0.173	3	-0.047	-0.022	0.034	-0.105	0.104	16	-0.066	-0.105	-0.009	-0.056	-0.125	29	285	30.4	8.35
IPI00310056.3	Protein-lysine 6-oxidase	65.69%	1	3	24	69							0.854	0.262	0.429	0.119	0.145	6																			411	46.7	8.43
IPI00133132.1	Protein-tyrosine kinase 2-beta	74.03%	4	6	87	208																									-0.004	0.135	-0.092	0.081	-0.076	5	1009	115.7	6.35
IPI00128982.1	Proteolipid protein 2	65.79%	1	1	8	35	-0.105	0.066	0.156	0.122	-0.037	5	0.204	-0.326	0.095	-0.218	-0.001	6							-0.099	0.072	-0.109	-0.095	-0.264	5							152	16.6	7.14
IPI00224784.4	Prothymosin alpha	45.95%	4	6	10	449	0.123	-0.039	0.172	0.001	0.165	53	-0.238	0.058	-0.057	0.085	-0.464	39	-0.544	-0.226	-0.411	0.144	-0.223	40	0.352	0.145	0.339	0.070	0.253	67	-0.091	-0.102	0.081	0.163	0.016	19	111	12.2	3.79
IPI00343214.5	Protocadherin Fat 2	62.63%	1	7	235	729																									0.010	-0.233	0.010	-0.199	-0.358	4	4351	479.8	5.12
IPI00120751.8	Proton myo-inositol cotransporter	52.90%	3	1	32	88																									0.087	-0.016	-0.253	0.154	0.014	4	637	69.0	6.86
IPI00471441.1	Ptms protein	69.19%	2	5	22	419	0.153	-0.069	0.181	0.003	0.259	43	-0.001	0.021	0.055	-0.053	0.004	26	0.004	-0.138	0.058	0.073	0.200	32	0.183	0.016	0.183	0.061	0.148	29	-0.085	0.033	0.181	0.163	0.134	21	211	23.1	4.79
IPI00858111.1	PTPRF interacting protein, binding protein 1	94.30%	1	1	28	66							0.358	-0.290	0.322	0.268	0.386	5																			193	20.5	10.42
IPI00330623.3	Pumilio domain-containing protein C14orf21 homolog	66.04%	1	5	49	125													0.665	0.055	0.446	0.415	0.650	4	0.289	0.301	0.134	0.122	-0.007	3							636	70.0	7.27
IPI00930762.1	pumilio homolog 2 isoform 2	44.36%	8	4	63	207							-0.063	-0.322	-0.080	-0.066	-0.306	7	0.316	0.003	0.140	-0.269	0.152	5	-0.193	-0.016	-0.163	0.063	-0.152	6							1064	114.0	7.08
IPI00315452.5	Purine nucleoside phosphorylase	94.81%	2	11	32	234	-0.010	-0.198	0.083	-0.158	0.120	18	0.242	-0.342	0.153	0.023	0.424	8	0.000	0.077	0.050	0.252	0.242	8	0.195	0.100	0.145	-0.022	0.184	30							289	32.3	6.16
IPI00130000.2	Puromycin-sensitive aminopeptidase	76.85%	6	31	77	514	-0.072	-0.106	-0.013	-0.031	-0.017	39	-0.513	0.066	-0.308	-0.066	-0.371	21	-0.326	-0.057	-0.122	0.038	-0.178	16	-0.297	0.047	-0.309	0.007	-0.315	12	0.027	0.083	0.017	0.044	0.057	59	920	103.3	5.90
IPI00162781.2	Putative adenosylhomocysteinase 2	93.96%	3	11	72	418	-0.231	-0.029	-0.077	-0.006	-0.168	6	-0.535	0.017	-0.610	-0.194	-0.409	11													-0.022	0.110	0.160	0.110	0.062	66	530	58.9	6.89
IPI00133270.1	Putative ATP-dependent Clp protease proteolytic subunit, mitochondria	78.31%	1	7	29	168	-0.086	0.199	-0.134	0.063	-0.112	9	0.467	-0.076	0.140	0.029	0.200	6	0.111	0.098	0.204	-0.076	-0.069	12	0.230	0.036	0.170	0.077	0.084	9							272	29.8	7.47
IPI00457961.1	Putative cyclooxygenase 3	71.65%	1	1	3	34																			1.048	0.297	-0.001	0.055	0.037	4							127	13.2	10.18
IPI00225151.1	Putative E3 ubiquitin-protein ligase UBR7	73.18%	1	2	32	82																			0.000	-0.226	-0.106	-0.094	-0.108	6							425	48.0	4.75
IPI00420814.3	Putative gag-pol protein	78.73%	5	18	100	707	0.079	0.120	-0.012	0.091	0.224	9	0.415	-0.155	-0.304	-0.111	-0.028	7							-0.219	-0.034	-0.311	-0.063	-0.146	143							1067	119.7	9.26
IPI00349069.4	Putative GTP-binding protein Parf	42.48%	1	6	35	68																									-0.333	0.096	0.075	-0.116	-0.025	7	725	79.8	5.53
IPI00830528.2	putative helicase MOV-10 isoform a	70.84%	3	9	95	301							-0.063	0.072	-0.188	0.116	-0.141	13							-0.116	-0.088	-0.158	-0.020	-0.315	5							1077	120.9	9.13
IPI00127495.3	Putative hexokinase HKDC1	77.60%	1	1	102	304	0.127	0.017	0.200	-0.083	0.262	4													-0.189	-0.405	-0.086	0.160	0.170	3	0.200	-0.157	-0.178	-0.122	-0.072	9	915	102.2	7.09
IPI00135277.1	Putative hydrolase RBBP9	66.67%	1	2	10	25																									0.175	0.052	0.147	0.240	0.329	5	186	20.9	5.97
IPI00111821.6	Putative oxidoreductase GLYR1	71.98%	1	8	54	171	0.014	0.021	-0.072	-0.173	-0.173	5							0.246	0.153	0.195	0.084	0.112	5	0.111	0.076	-0.001	0.139	0.192	3							546	59.7	9.22
IPI00128818.2	Putative pre-mRNA-splicing factor ATP-dependent RNA helicase DHX15	83.77%	2	25	99	680	-0.016	0.132	-0.086	0.038	-0.056	25	-0.086	0.069	-0.165	0.055	-0.102	41	0.001	-0.021	0.084	0.030	0.122	18	0.010	-0.042	0.141	-0.006	0.069	40	-0.318	-0.241	-0.097	0.009	0.004	6	795	90.9	7.46
IPI00311453.5	putative ribosomal RNA methyltransferase NOP2	80.35%	1	25	84	666	-0.188	0.076	-0.231	0.051	-0.183	45	0.004	-0.029	-0.154	0.017	0.062	30	0.152	0.057	0.070	-0.003	0.124	26	0.048	0.009	-0.085	-0.016	-0.044	21							794	86.9	9.19
IPI00130883.1	Putative RNA-binding protein 3	98.69%	1	1	24	293	-0.088	-0.079	0.161	0.061	0.027	22	-0.199	-0.088	-0.112	0.020	-0.099	16	0.262	0.060	0.250	0.054	0.188	29	0.737	0.249	0.479	0.057	0.476	8	-0.189	-0.233	-0.100	0.104	-0.243	5	153	16.6	7.50
IPI00378187.2	putative RNA-binding protein 3 isoform 1	95.45%	2	1	24	269	-0.103	-0.089	0.163	0.156	0.016	13	-0.199	-0.088	-0.112	0.020	-0.099	16	0.253	0.049	0.214	0.019	0.140	26	0.629	0.045	0.364	0.057	0.409	4	-0.317	-0.233	-0.100	0.104	-0.243	3	154	16.8	8.38
IPI00119632.3	Putative rRNA methyltransferase 3	74.94%	1	18	80	551	-0.155	-0.026	-0.150	-0.045	-0.123	28	0.234	-0.117	0.153	0.091	0.124	29	0.126	0.040	0.031	-0.056	0.050	18	0.061	-0.016	-0.009	0.003	-0.023	34							838	95.5	8.38
IPI00988413.1	putative transposase element L1Md-A101/L1Md-A102/L1Md-A2-like	74.59%	51	1	44	146																			-0.133	0.009	0.349	-0.183	0.247	4							429	49.1	8.54
IPI00109108.2	Putative uncharacterized protein	69.59%	2	15	61	389	-0.060	0.064	-0.072	0.027	-0.089	27	0.089	0.092	-0.109	0.066	0.136	25	0.287	0.085	0.277	0.033	0.058	20	0.021	-0.044	-0.026	0.000	0.043	15							753	85.9	7.97
IPI00113287.1	Putative uncharacterized protein	41.77%	2	3	15	94																									-0.083	-0.013	-0.122	0.011	-0.054	6	249	27.9	6.54
IPI00115231.2	Putative uncharacterized protein	53.17%	3	6	65	199													0.157	0.042	0.107	0.073	0.128	7							0.000	0.149	0.112	0.102	0.062	15	1087	120.7	6.60
IPI00115644.2	Putative uncharacterized protein	77.27%	2	5	19	245	-0.003	-0.091	-0.038	-0.007	0.100	11	0.208	-0.028	0.021	-0.112	0.024	12	-0.025	0.004	0.017	0.037	-0.004	13	0.276	0.151	0.287	0.031	0.325	23	0.135	0.136	0.030	0.058	0.043	13	198	22.3	7.03
IPI00117569.3	Putative uncharacterized protein	96.39%	3	11	30	390	0.144	0.026	0.187	0.038	0.110	30	0.033	0.009	-0.022	-0.006	0.152	33	-0.004	0.052	-0.065	0.069	-0.007	43	0.104	0.076	0.154	0.051	0.108	33	-0.304	-0.091	-0.035	-0.136	-0.119	12	166	19.4	10.14
IPI00119079.6	Putative uncharacterized protein	79.05%	1	8	43	212	0.014	-0.116	-0.004	-0.041	0.102	23	-0.144	0.021	0.403	-0.523	0.696	3	0.507	-0.027	0.681	-0.211	0.943	4													377	42.7	6.67
IPI00123099.2	Putative uncharacterized protein	46.03%	1	3	9	28																									-0.517	-0.271	-0.290	-0.411	-0.521	3	252	28.3	5.43
IPI00123292.5	Putative uncharacterized protein	69.00%	5	19	56	490	-0.071	0.007	-0.102	-0.094	-0.076	28	-0.357	-0.057	-0.718	-0.077	-0.615	43	-0.111	0.028	0.020	-0.025	0.050	18	0.050	-0.053	0.024	-0.053	0.031	26							700	74.4	7.74
IPI00126396.2	Putative uncharacterized protein	90.56%	5	31	88	629	-0.044	-0.009	-0.071	0.006	-0.031	45	-0.222	0.010	0.110	0.083	-0.039	49	0.158	-0.108	0.114	-0.122	0.202	15	0.222	-0.001	0.347	-0.017	0.297	51							720	81.3	6.37
IPI00126892.12	Putative uncharacterized protein	95.03%	1	8	31	163	-0.138	-0.034	-0.304	-0.069	-0.184	9	-0.604	-0.278	-0.432	-0.269	-0.355	8							0.131	0.081	0.124	0.127	0.210	7							302	34.3	7.14
IPI00128441.3	Putative uncharacterized protein	87.34%	4	24	76	754	-0.051	0.100	-0.050	0.088	-0.171	16	-0.150	0.040	-0.178	0.098	-0.175	44	0.128	0.072	0.027	-0.089	-0.045	34	-0.023	-0.044	0.079	-0.047	-0.001	73	-0.053	0.267	0.123	0.180	0.089	16	632	70.8	8.13
IPI00132134.3	Putative uncharacterized protein	69.94%	7	2	88	357							-0.265	-0.006	-0.193	-0.456	-0.168	3																			795	88.0	9.33
IPI00132475.2	Putative uncharacterized protein	83.00%	2	17	53	517	0.073	0.099	-0.045	0.066	-0.031	13	0.274	-0.012	0.220	0.100	0.189	39	0.267	0.087	0.162	-0.006	0.037	27	-0.074	-0.053	-0.167	-0.057	-0.105	21							547	61.2	6.44
IPI00133342.1	Putative uncharacterized protein	96.90%	2	5	12	108	-0.053	-0.048	-0.131	-0.014	-0.171	17							0.347	0.254	0.202	0.070	0.037	4	-0.077	-0.048	0.246	0.014	0.184	6	0.208	-0.057	0.124	-0.088	0.091	21	129	14.2	8.63
IPI00133977.3	Putative uncharacterized protein	73.22%	3	9	37	438	-0.111	-0.085	0.009	0.004	-0.023	34	-0.009	0.016	-0.012	-0.130	-0.026	19	-0.136	-0.181	-0.196	-0.059	-0.035	10	0.052	-0.079	0.000	-0.026	0.040	41	0.030	0.051	0.118	-0.050	0.027	24	422	47.3	4.73
IPI00134344.6	Putative uncharacterized protein	85.80%	2	71	273	1327	0.029	0.004	-0.071	-0.038	-0.006	11	0.457	0.160	0.414	0.130	0.432	22	-0.023	0.034	0.001	0.072	-0.092	11	-0.347	0.034	-0.267	0.016	-0.240	7	-0.013	0.000	0.038	0.057	0.033	232	2388	270.8	5.86
IPI00134373.1	Putative uncharacterized protein	72.86%	3	3	18	47																									0.153	0.085	-0.050	0.145	-0.136	4	199	21.4	9.88
IPI00134476.2	Putative uncharacterized protein	68.89%	3	4	20	54																									-0.146	-0.062	0.203	0.045	0.156	6	315	35.1	7.81
IPI00139168.2	Putative uncharacterized protein	84.02%	1	7	118	642	-0.114	-0.031	-0.086	-0.014	-0.131	37	-0.039	-0.019	-0.071	0.083	-0.127	34	0.436	0.096	0.273	-0.068	0.094	9	0.179	0.003	0.136	0.016	0.114	42							1014	112.6	8.98

IPI00222496.3	Putative uncharacterized protein	81.12%	1	2	52	656	-0.091	0.076	-0.106	0.001	-0.068	49	-0.097	-0.009	-0.123	0.043	-0.203	41	0.474	0.102	0.458	-0.010	0.292	29	0.112	-0.114	-0.050	-0.047	0.166	77	-0.271	-0.127	0.064	-0.105	-0.031	13	445	48.7	5.19
IPI00222550.8	Putative uncharacterized protein	82.47%	1	1	18	95	0.275	0.089	-0.031	0.021	-0.042	7	0.158	0.171	0.066	-0.025	0.057	5	0.416	0.134	0.011	0.227	0.051	9													97	10.7	10.67
IPI00229911.2	Putative uncharacterized protein	75.88%	2	5	15	85							-0.029	-0.128	-0.206	0.024	0.139	7	0.064	0.093	0.343	-0.206	0.107	5													228	24.8	5.12
IPI00272381.5	Putative uncharacterized protein	85.56%	3	17	53	370	0.130	0.112	0.092	0.027	0.119	9	0.108	0.023	0.122	0.043	-0.007	22	0.720	0.059	0.567	-0.080	0.517	30	0.312	0.026	0.502	-0.047	0.464	14							561	63.8	5.90
IPI00277930.7	Putative uncharacterized protein	69.91%	6	11	30	252	-0.358	-0.059	-0.476	0.000	-0.480	15	-0.894	-0.243	-0.484	-0.563	-0.243	19	0.257	-0.115	0.265	0.045	0.448	24													349	38.7	6.95
IPI00320462.4	Putative uncharacterized protein	78.93%	2	9	33	124	0.185	0.029	0.301	0.107	0.135	6																			-0.134	0.026	0.066	-0.072	-0.060	15	299	33.7	8.94
IPI00322095.4	Putative uncharacterized protein	54.29%	2	1	6	190	-0.079	-0.019	-0.269	-0.353	-0.201	5																									210	23.7	4.83
IPI00323820.4	Putative uncharacterized protein	78.20%	1	33	102	822	-0.028	0.000	-0.054	-0.031	-0.057	62	-0.086	0.050	0.084	0.103	-0.004	57	-0.092	-0.161	-0.014	-0.012	0.193	15	0.294	0.010	0.257	-0.013	0.257	49							913	103.1	5.76
IPI00338458.2	Putative uncharacterized protein	59.68%	1	8	54	152	-0.023	0.100	-0.196	0.091	-0.034	7							0.262	0.134	0.104	-0.116	-0.025	6	0.029	-0.204	-0.147	-0.157	-0.404	3	-0.054	0.143	0.050	0.058	0.140	3	687	77.9	5.67
IPI00352163.3	Putative uncharacterized protein	80.05%	1	1	175	1331							-0.569	0.239	0.051	0.557	-0.525	49	0.683	0.096	0.690	-0.050	0.470	292	0.744	0.084	0.522	0.029	0.566	4							2296	252.8	6.07
IPI00356872.3	Putative uncharacterized protein	57.75%	1	1	26	76																									0.416	0.156	0.236	0.408	0.457	3	497	57.3	6.84
IPI00399961.2	Putative uncharacterized protein	87.98%	1	15	62	387	-0.056	0.130	-0.022	0.027	-0.111	25	0.003	0.076	-0.035	-0.144	-0.042	12	0.006	-0.013	-0.103	0.016	0.003	18	-0.026	-0.091	0.009	-0.065	0.011	27	0.198	0.327	0.128	0.247	0.347	4	524	59.0	7.34
IPI00407543.2	Putative uncharacterized protein	82.53%	1	3	34	817	0.286	-0.154	0.325	-0.025	0.392	66	-0.470	0.019	-0.306	-0.144	-0.272	49	-0.194	-0.159	-0.183	0.143	-0.057	84	-0.106	0.010	-0.181	0.020	-0.243	62	0.027	-0.042	-0.034	-0.194	-0.044	62	229	24.8	8.34
IPI00410836.1	Putative uncharacterized protein	82.97%	2	15	47	384	0.270	0.004	0.294	-0.031	0.283	34	-0.221	-0.089	-0.142	-0.255	-0.149	20	0.006	-0.045	-0.117	-0.029	0.103	16	0.094	0.009	0.134	0.040	0.079	23							411	45.8	8.24
IPI00457731.1	Putative uncharacterized protein	69.86%	1	1	26	331	0.161	0.242	0.167	0.103	0.094	14	-0.218	-0.152	-0.265	-0.007	-0.071	15	-0.072	-0.039	-0.141	-0.157	-0.127	19	-0.039	-0.077	0.059	-0.025	-0.065	19							282	32.1	8.94
IPI00458795.1	Putative uncharacterized protein	66.10%	5	7	30	145	-0.128	0.112	0.033	0.031	-0.146	4																			0.089	-0.025	-0.283	-0.236	-0.189	16	236	25.4	8.24
IPI00468653.4	Putative uncharacterized protein	77.43%	1	1	53	223	0.026	0.170	0.089	0.076	0.083	3																			0.011	0.100	0.092	0.021	0.153	19	505	55.0	8.16
IPI00469218.1	Putative uncharacterized protein	82.06%	1	6	43	412	-0.131	0.041	-0.112	0.009	-0.170	8	-0.106	0.234	-0.076	0.163	-0.119	15	0.072	0.130	0.030	0.043	-0.130	31	0.059	-0.034	-0.131	-0.020	-0.083	12	0.048	0.112	0.190	0.227	0.077	7	407	43.9	8.40
IPI00473667.1	Putative uncharacterized protein	89.11%	1	2	36	1281	-0.014	-0.189	-0.023	-0.023	-0.012	51	-0.233	-0.051	-0.211	-0.085	-0.074	37	-0.211	-0.138	-0.188	0.130	-0.025	69	-0.155	0.001	-0.127	-0.097	-0.112	62	0.122	0.102	0.061	0.069	0.112	184	395	44.0	6.07
IPI00473680.2	Putative uncharacterized protein	84.76%	2	10	35	300	0.098	0.080	0.135	0.030	0.033	21	0.257	-0.274	0.198	-0.236	0.143	15	0.433	0.068	0.406	-0.120	0.318	17	-0.014	-0.139	-0.127	-0.007	-0.089	13	-0.381	-0.146	0.059	-0.125	0.083	6	269	31.2	9.58
IPI00474820.1	Putative uncharacterized protein	80.11%	2	3	19	80	-0.191	0.262	-0.111	0.011	-0.035	4													-0.088	0.069	0.016	0.062	-0.037	5							186	21.1	8.13
IPI00474959.2	Putative uncharacterized protein	84.79%	2	17	35	427	-0.074	0.059	-0.001	0.118	-0.201	22	0.228	-0.047	0.244	-0.050	0.130	50	0.530	0.181	0.375	0.079	0.327	7	-0.122	-0.039	-0.099	0.021	0.064	13	0.092	0.084	-0.035	-0.146	0.088	5	355	41.0	4.59
IPI00553777.2	Putative uncharacterized protein	80.97%	1	1	46	1041	0.107	0.010	0.076	0.014	0.123	68	-0.193	-0.013	-0.146	-0.013	-0.191	39	0.126	0.084	0.131	-0.012	0.098	66	0.009	-0.041	0.076	0.006	0.088	93	0.072	0.256	0.233	0.258	0.301	17	373	38.8	9.13
IPI00626139.2	Putative uncharacterized protein	79.91%	3	1	37	349	0.108	0.061	0.085	0.009	0.043	10	0.073	0.118	0.119	-0.004	0.112	22	-0.007	0.046	-0.003	-0.038	-0.114	20	0.169	0.135	0.055	0.069	0.116	11	0.073	0.204	0.054	0.212	0.212	6	219	24.0	9.99
IPI00649300.2	Putative uncharacterized protein	45.53%	2	10	34	172	-0.228	0.065	-0.141	-0.054	-0.265	6	-0.016	0.069	-0.271	-0.144	-0.357	3	0.023	0.086	0.143	0.026	-0.120	9							0.038	0.024	0.118	0.084	-0.026	12	492	54.0	8.87
IPI00651875.3	Putative uncharacterized protein	98.02%	4	3	37	69	0.069	0.070	0.069	-0.035	0.051	3	-0.130	-0.131	0.745	-0.236	-0.260	3																			253	26.4	9.67
IPI00652445.1	Putative uncharacterized protein	83.91%	1	1	45	291	-0.211	-0.050	0.048	-0.034	0.021	10	0.697	0.141	0.438	0.305	0.598	9	-0.183	-0.065	-0.092	-0.023	-0.026	10	-0.038	-0.056	-0.088	-0.209	-0.069	4	0.038	0.017	0.203	-0.074	-0.189	5	379	43.0	6.23
IPI00652722.1	Putative uncharacterized protein	48.33%	2	1	24	43																									0.145	0.280	0.228	-0.154	-0.006	3	629	69.4	7.74
IPI00652778.1	Putative uncharacterized protein	64.91%	3	2	29	78							0.335	0.386	0.309	0.080	0.525	3																			399	46.3	7.87
IPI00652813.1	Putative uncharacterized protein	78.23%	3	4	178	1361							-0.569	0.239	0.051	0.557	-0.525	49	0.689	0.097	0.695	-0.051	0.470	307	0.744	0.084	0.522	0.029	0.566	4							2361	260.0	5.88
IPI00652998.1	Putative uncharacterized protein	90.52%	2	1	77	1353	0.033	0.092	0.024	0.020	-0.065	77	0.132	-0.004	0.107	0.096	0.004	88	0.257	0.052	0.169	-0.013	0.064	144	0.224	-0.053	0.187	-0.022	0.368	134	-0.057	-0.059	-0.103	-0.168	-0.111	66	612	70.9	5.38
IPI00653300.1	Putative uncharacterized protein	41.64%	4	4	31	144	0.269	0.050	0.020	0.104	0.179	10	1.089	0.123	1.185	0.519	0.742	9	0.136	0.044	0.294	0.088	0.335	5													670	76.0	7.30
IPI00654420.1	Putative uncharacterized protein	91.10%	4	7	40	193	0.122	-0.066	0.091	-0.028	0.162	5	0.115	0.115	0.178	0.003	0.163	7							-0.112	0.087	-0.035	0.085	0.057	6	-0.004	-0.050	-0.173	-0.128	-0.042	13	326	35.9	8.51
IPI00656182.1	Putative uncharacterized protein	65.17%	1	1	23	105	-0.026	0.160	-0.173	-0.079	-0.315	3							0.498	0.093	0.314	0.084	0.120	7	0.179	0.323	0.185	0.171	-0.258	4	0.007	0.066	-0.004	0.193	0.023	3	267	30.1	6.70
IPI00749720.1	Putative uncharacterized protein	52.34%	1	6	47	106																									-0.020	-0.056	-0.152	-0.223	-0.313	3	982	110.1	5.81
IPI00749735.1	Putative uncharacterized protein	44.25%	3	3	20	94																									0.026	0.036	-0.160	-0.340	0.020	8	348	37.2	9.33
IPI00757186.1	Putative uncharacterized protein	59.63%	3	8	30	126													-0.178	0.016	-0.097	-0.523	0.552	4	0.037	0.212	0.087	0.075	0.027	5	0.222	0.296	0.188	0.111	0.064	6	374	39.7	8.09
IPI00761938.3	Putative uncharacterized protein	54.37%	1	1	19	55																									-0.085	0.001	0.058	0.242	0.009	7	355	39.3	9.25
IPI00830623.1	Putative uncharacterized protein	50.48%	4	10	45	725	0.143	0.077	0.187	0.065	0.048	32	-0.407	0.004	-0.404	-0.076	-0.494	45	0.204	0.084	0.194	-0.095	0.157	37	0.116	0.045	0.199	0.076	0.139	54	-0.017	0.111	0.069	0.051	0.151	8	517	52.6	9.36
IPI00856220.1	Putative uncharacterized protein	62.81%	3	4	96	288																			0.162	0.273	0.692	0.140	0.456	5							1842	199.4	6.60
IPI00876001.21	Putative uncharacterized protein	73.75%	3	11	64	261	0.111	-0.035	0.236	-0.014	0.202	7							-0.209	0.028	0.006	0.143	0.077	5	-0.170	-0.167	-0.160	-0.157	-0.089	4	0.030	-0.152	0.073	-0.044	0.080	9	522	59.0	5.22
IPI00889878.1	Putative uncharacterized protein	91.04%	1	1	66	355	0.003	0.037	0.241	0.108	0.064	4	0.084	-0.023	-0.017	0.112	0.115	11	0.134	-0.072	0.081	0.051	0.087	12	-0.060	-0.031	-0.109	0.077	-0.044	10							502	55.5	8.10
IPI00974711.2	Putative uncharacterized protein	70.56%	31	2	32	161							-0.250	0.044	-0.176	0.059	-0.267	20	0.048	0.026	0.185	-0.183	0.048	4							0.050	-0.072	0.354	-0.157	0.100	3	360	40.3	6.37
IPI00986198.1	Putative uncharacterized protein	82.04%	8	4	36	101	0.207	-0.009	0.126	-0.023	0.107	4							-0.069	-0.010	-0.128	-0.111	-0.155	5													334	38.5	9.41
IPI01026702.1	Putative uncharacterized protein	72.19%	5	1	112	544	0.087	-0.085	-0.088	-0.077	-0.004	6	-0.494	-0.016	-0.383	-0.039	-0.500	11	-0.168	0.052	-0.108	-0.180	0.140	9							-0.333	0.075	0.069	-0.088	-0.271	3	1122	129.6	5.43
IPI01027221.1	Putative uncharacterized protein	62.47%	1	9	26	203	0.077	0.055	0.038	0.050	0.021	16	0.181	0.083	0.051	-0.051	0.070	12																			397	42.8	4.87
IPI01027577.1	Putative uncharacterized protein	86.17%	4	4	65	337	-0.047	-0.123	-0.047	-0.100	0.009	17	-0.109	0.037	-0.108	0.011	-0.003	9	-0.318	-0.178	-0.102	0.055	0.041	8	0.240	0.167	0.147	0.158	0.124	12	0.079	-0.026	0.007	0.050	-0.004	22	694	76.0	5.19
IPI00118995.3	Putative uncharacterized protein (Fragment)	71.63%	2	4	31	95	-0.209	-0.221	0.038	-0.168	-0.079	3	0.366	-0.136	0.430	0.208	0.380	7	0.360	-0.237	0.388	-0.519	0.106	3													356	40.3	8.53
IPI00268553.4	Putative uncharacterized protein (Fragment)	70.79%	3	4	19	77	0.200	-0.099	0.222	0.019	0.387	8																									267	30.5	8.97
IPI00459253.1	Putative uncharacterized protein (Fragment)	62.93%	1	1	42	109	0.098	-0.063	0.180	-0.014	0.116	6	-0.421	-0.147	-0.155	-0.028	-0.281	4																			491	56.9	5.10
IPI00473453.2	Putative uncharacterized protein (Fragment)	90.91%	1	1	35	220													-0.154	-0.021	-0.336	-0.088	-0.065	3							0.143	-0.028	-0.240	-0.122	-0.062	9	264	28.8	8.63
IPI00474254.1	Putative uncharacterized protein (Fragment)	55.85%	1	1	30	80	-0.246	-0.231	0.068	-0.123	0.031	4																									410	42.4	7.94
IPI00652944.1	Putative uncharacterized protein (Fragment)	80.76%	1	1	69	299	0.010	-0.031	-0.386	-0.127	-0.168	5	0.073	0.003	0.112	-0.017	0.176	19	0.170	0.083	0.132	-0.136	-0.054	12	-0.580	-0.083	-0.191	0.047	-0.224	3							655	70.5	6.90
IPI00653573.1	Putative uncharacterized protein (Fragment)	94.62%	1	1	67	575	0.147	0.020	0.108	-0.003	0.149	34	-0.128	-0.006	-0.099	-0.130	-0.029	37	-0.265	-0.074	-0.241	-0.004	-0.141	36	-0.243	-0.186	-0.085	-0.245	-0.155	10	0.036	0.006	0.030	-0.053	-0.099	22	502	56.3	9.36
IPI00654263.1	Putative uncharacterized protein (Fragment)	52.05%	1	2	14	80	-0.095	-0.149	-0.123	0.024	0.036	8	-0.446	-0.091	-0.152	-0.194	-0.170	10																			244	24.8	4.56
IPI00756279.1	Putative uncharacterized protein (Fragment)	68.27%	2	4	30	78							0.336	-0.034	0.354	0.052	0.290	12																			457	51.4	5.33
IPI00989752.1	Putative uncharacterized protein (Fragment)	78.88%	3	15	43	396	0.358	-0.131	0.526	0.021	0.396	24	0.068	-0.056	0.171	-0.045	0.204	19	-0.181	-0.024	0.045	0.079	-0.106	8	-0.072	-0.072	-0.100	-0.001	-0.013	29	-0.095	0.026	-0.080	-0.029	0.057	17	483	52.5	8.60
IPI00283511.1	Pyridoxal kinase	85.26%	2	14	29	154																			0.197	0.199	0.273	0.321	0.364	5	0.040	0.140	-0.031	-0.045	0.107	38	312	35.0	6.29
IPI00118654.2	Pyridoxal phosphate phosphatase	77.05%	1	6	23	105																									-0.022	-0.105	-0.102	-0.281	-0.203	13	292	31.5	5.74
IPI00123510.1	Pyrroline-5-carboxylate reductase 1, mitochondrial	75.73%	4	7	31	126							0.271	0.104	0.419	0.033	0.495	23	0.158	0.078	-0.157	-0.069	-0.218	4													309	32.4	6.86
IPI00123278.1	Pyrroline-5-carboxylate reductase 2	89.06%	1	8	38	285	0.009	0.068	0.135	0.021	0.029	18	0.115	-0.006	0.081	-0.012	0.119	27	0.076	0.070	-0.133	-0.020	-0.165	17	0.286	-0.117	0.403	-0.028	0.295	9							320	33.6	7.77
IPI00153234.2	Pyrroline-5-carboxylate reductase 3	52.92%	1	4	22	112							-0.041	0.048	0.047	0.029	0.000	5							-0.041	-0.066	0.009	-0.062	-0.035	4	0.383	0.174	0.293	0.408	0.178	3	274	28.7	7.27
IPI00337893.2	Pyruvate dehydrogenase E1 component subunit alpha, somatic form, mitoc	96.15%	1	22	59	449	-0.050	0.026	-0.051	0.065	0.058	8	0.576	0.044	0.508	-0.006	0.336	16	0.203	0.046	0.165	0.108	0.027	13	0.033	-0.088	0.034	-0.094	0.123	22	0.054	0.076	0.061	0.026	0.064	65	390	43.2	8.19
IPI00132042.4	Pyruvate dehydrogenase E1 component subunit beta, mitochondria	82.73%	1	14	28	445	0.110	0.089	0.103	0.020	0.091	10	0.428	-0.026	0.495	0.023	0.395	23	0.279	0.098	0.153	-0.082	0.131	19	0.178	-0.028	0.207	0.013	0.151	27	0.081	0.001	0.026	-0.063	-0.003	89	359	38.9	6.87
IPI00453792.2	Pyruvate dehydrogenase phosphatase regulatory subunit, mitochondria	60.25%	2	1	59	163																									-0.198	0.175	0.143	0.425	-0.032	3	878	99.2	6.35
IPI00222767.1	Pyruvate dehydrogenase protein X component, mitochondria	83.23%	2	9	52	327	-0.020	0.019	-0.079	0.095	0.029	16	0.303	-0.167	0.297	0.013	0.119	9							-0.022	-0.290	-0.250	-0.272	0.195	3	0.044	-0.062	0.001	-0.100	0.016	29	501	54.0	7.75
IPI00133605.2	Pyruvate kinase isozymes R/L	72.47%	2	2	50	294	-0.095	0.029	0.054	-0.012	0.212	3	-0.226	-0.063	-0.175	-0.039	-0.066	6	-0.344	-0.207	-0.402	0.103	-0.189	14	0.387	0.218	0.297	0.083	0.169	8	0.208	0.070	-0.047	-0.050	-0.020	10	574	62.3	7.09
IPI00134704.1	Quinone oxidoreductase	82.78%	5	8	36	90	0.069	0.106	0.183	-0.047	0.145	7																			-0.260	0.034	0.058	0.088	-0.007	8	331	35.2	8.07
**IPI00323179.3**	**Rab GDP dissociation inhibitor alpha**	**97.32%**	**2**	**20**	**53**	**979**	0.079	-0.149	0.160	-0.111	0.048	**21**	-0.278	-0.013	-0.122	-0.059	-0.056	**28**	-0.260	-0.139	-0.194	0.038	-0.048	**31**	0.076	0.227	0.119	0.069	0.098	**71**	-0.013	-0.013	-0.019	-0.066	-0.038	**159**	**447**	**50.5**	**5.08**
IPI00134979.1	Rab proteins geranylgeranyltransferase component A 1	54.74%	3	3	39	129	-0.086	0.007	-0.308	-0.206	-0.065	4																									665	73.9	4.68
IPI00480432.2	rab11 family-interacting protein 5 isoform 1	57.97%	3	7	72	240																									-0.252	0.010	0.000	0.075	-0.029	3	1318	139.5	5.12
IPI00555130.2	rab3 GTPase-activating protein non-catalytic subunit	55.08%	2	13	84	277							0.330	-0.201	-0.320	-0.128	0.000	5													-0.051	-0.108	-0.119	0.041	-0.050	13	1387	154.5	6.07
IPI00116563.2	Rab5B	92.58%	3	6	25	227	-0.060	0.147	-0.014	0.051	0.034	4	0.147	-0.009	0.119	-0.053	0.250	4	-0.074	0.013	-0.150	0.020	0.007	13	-0.176	0.073	-0.150	-0.196	-0.224	5	0.009	-0.032	0.081	0.006	0.044	29	229	25.3	6.92
IPI00111151.2	Rabphilin-3A	71.95%	1	14	53	205																									-0.127	-0.009	-0.014	-0.060	-0.014	42	681	75.4	8.27
IPI00126176.3	Rac GTPase-activating protein 1	83.92%	4	8	69	283	-0.318	0.051	-0.229	0.068	-0.189	8							0.492	0.068	0.167	0.241	0.061	10	0.123	0.045	0.058	-0.122	0.162	7							628	70.1	8.51
IPI00315187.5	Ragulator complex protein LAMTOR1	97.52%	1	8	16	175	0.222	0.080	0.238	-0.017	0.309	12	-0.083	0.051	-0.100	0.040	-0.320	9							-0.025	-0.142	-0.149	-0.106	-0.149	15							161	17.7	5.15
IPI00119807.1	Ragulator complex protein LAMTOR2	65.60%	2	3	9	60	-0.168	-0.035	0.014	0.065	-0.180	10	-0.786	-0.358	-0.764	-0.519	-0.474	4																			125	13.5	5.40
IPI00467338.5	Ran GTPase-activating protein 1	75.89%	3	2	65	366	-0.012	-0.010	-0.025	-0.010	-0.131	27	-0.138	-0.088	-0.176	-0.204	0.081	17	-0.241	-0.022	-0.165	0.119	-0.175	18	0.207	0.021	0.163	-0.042	0.122	17							589	63.5	4.68
IPI00135190.3	Ran-binding protein 3	73.12%	1	15	49	581	-0.010	0.040	0.031	0.066	0.017	26	-0.149	-0.042	-0.189	0.020	-0.139	17	-0.331	-0.160	-0.248	0.092	-0.031	14	0.444	0.027	0.274	0.034	0.316	24							491	52.5	5.12
IPI00471224.1	Ran-binding protein 6	83.89%	8	2	97	472							0.263	0.102	-0.009	0.174	0.080	5	-0.241	0.008	0.151	-0.206	0.053	4	-0.138	-0.080	-0.023	-0.131	-0.188	3							1105	124.5	5.03
IPI01023308.1	Rangap1 protein	76.06%	1	1	65	349	-0.097	-0.034	-0.038	0.003	-0.216	25	-0.175	-0.092	-0.199	-0.211	0.048	16	-0.173	-0.013	-0.094	0.149	-0.173	16	0.207	0.021	0.163	-0.042	0.122	17							589	63.5	4.68
IPI00321978.3	Ran-specific GTPase-activating protein (Fragment)	87.19%	4	8	22	334	-0.168	-0.012	0.061	0.038	-0.111	18	-0.274	-0.045	-0.329	-0.123	-0.409	18	-0.531	-0.151	-0.204	0.136	0.054	12	0.068	0.029	0.041	-0.017	0.154	36	0.167	0.057	-0.034	0.123	0.003	9	203	23.6	5.22
IPI00653794.2	RAP1, GTP-GDP dissociation stimulator 1 isoform a	98.52%	4	29	70	613	-0.136	-0.170	-0.069	-0.167	-0.100	5	-0.613	-0.077	-0.559	-0.149	-0.531	20	0.061	0.106	0.044	0.341	-0.044	3	-0.102	0.038	-0.142	0.000	-0.013	12	-0.045	-0.059	-0.095	-0.112	-0.088	89	607	66.0	5.35
IPI00648543.5	Ras association (RalGDS/AF-6) and pleckstrin homology domains 1	58.45%	4	4	82	272	-0.041	0.052	-0.417	-0.062	0.122	3																									1266	136.7	8.98
IPI00663736.3	ras GTPase-activating protein SynGAP isoform 3	72.24%	5	22	131	384																									-0.014	0.135	0.099	0.130	0.081	51	1340	148.1	8.98
IPI00130095.1	Ras GTPase-activating protein-binding protein 1	78.06%	2	16	39	957	-0.086	0.029	0.041	0.061	0.013	65	0.172	-0.086	0.050	-0.056	0.066	58	0.009	0.080	-0.069	0.047	-0.092	64	0.136	-0.035	0.096	-0.004	0.145	76							465	51.8	5.59
IPI00989933.2	ras GTPase-activating-like protein IQGAP1	86.42%	4	78	205	2457	0.207	-0.004	0.197	-0.016	0.179	196	0.023	0.044	-0.086	0.024	-0.059	138	0.075	-0.037	0.066	-0.006	0.072	140	-0.099	0.013	-0.117	0.007	-0.100	97							1657	188.6	6.48
IPI00406193.4	Ras GTPase-activating-like protein IQGAP2	80.13%	2	4	173	749	0.253	-0.009	0.192	0.004	0.184	9	-0.117	0.011	-0.063	-0.010	-0.265	3	-0.016	0.096	0.010	-0.053	0.301	7	0.033	0.103	0.014	0.038	0.031	6							1575	180.4	5.64
IPI00130621.4	RAS p21 protein activator 1	58.19%	2	3	75	193																			-0.147	0.072	-0.097	-0.029	-0.142	10							1038	115.4	6.60
IPI00555071.2	Ras suppressor protein 1	90.61%	6	9	34	367	-0.095	-0.001	-0.154	0.034	-0.092	17	0.001	-0.079	-0.072	-0.105	-0.122	19	-0.120	-0.121	-0.037	0.087	-0.048	12	0.193	-0.034	0.240	-0.048	0.231	23							277	31.5	8.88
IPI00130555.2	RasGAP-activating-like protein 1	63.95%	1	10	71	225																									0.156	0.030	-0.208	-0.130	-0.106	13	799	89.3	6.37
IPI00157462.1	Ras-related C3 botulinum toxin substrate 3	63.54%	9	1	16	192	0.303	-0.054	0.247	-0.136	0.374	13	0.034	0.188	0.235	0.251	0.112	20	-0.031	-0.053	-0.050	0.033	-0.014	20							-0.173	0.107	0.040	0.084	0.016	23	192	21.4	8.15
IPI00117357.1	Ras-related protein M-Ras	59.13%	2	3	19	49																									-0.208	-0.324	-0.170	0.151	0.160	4	208	23.9	8.78
IPI00130118.1	Ras-related protein Rab-10	95.50%	8	11	34	407	0.143	0.106	0.176	0.041	0.087	21	-0.019	-0.031	-0.023	0.154	0.021	18	0.262	0.133	0.176	-0.042	0.061	23	-0.265	0.038	-0.324	0.011	-0.278	15	0.089	0.048	0.020	0.073	-0.023	30	200	22.5	8.38
IPI00323897.3	Ras-related protein Rab-11A	93.98%	9	1	28	291	0.390	-0.041	0.320	0.044	0.268	20	0.534	0.107	0.434	0.034	0.449	5	0.215	0.109	0.151	-0.042	-0.032	21	0.108	0.006	-0.125	0.044	-0.029	20	0.062	-0.020	0.026	-0.031	0.024	24	216	24.4	6.57
IPI00135869.3	Ras-related protein Rab-11B	94.95%	10	2	28	333	0.185	-0.047	0.145	-0.022	0.212	18	0.149	0.065	0.064	0.036	0.085	8	0.189	0.076	0.072	-0.048	-0.069	24	0.096	-0.004	-0.035	0.059	0.024	28	0.076	-0.026	0.051	-0.017	0.027	29	218	24.5	5.94
IPI00121183.1	Ras-related protein Rab-13	90.10%	2	2	31	210	0.214	0.130	0.130	0.029	0.087	3	0.048	-0.028	0.061	0.001	0.062	3	0.141	0.089	0.176	-0.042	-0.003	5	0.184	0.210	-0.071	0.103	-0.123	5	0.033	0.138	0.036	0.144	-0.007	6	202	22.8	9.50
IPI00126042.3	Ras-related protein Rab-14	93.95%	9	14	26	401	-0.013	-0.038	-0.025	-0.042	0.030	32	-0.068	-0.020	-0.141	-0.077	0.122	15	0.031	0.123	0.048	-0.026	-0.112	14	-0.133	-0.003	-0.146	-0.041	-0.128	16	-0.069	-0.053	-0.119	-0.059	-0.074	37	215	23.9	6.21
IPI00170032.1	Ras-related protein Rab-15	81.60%	14	1	25	199	0.209	0.088	0.080	0.026	0.026	4	0.050	-0.023	-0.019	0.043	0.051	4	0.281	0.119	0.193	-0.013	0.234	3	0.184	0.210	-0.071	0.103	-0.123	5	0.107	-0.260	-0.320	-0.062	-0.203	8	212	24.3	5.71
IPI00116770.1	Ras-related protein Rab-18	90.78%	1	9	30	215	-0.097	-0.186	-0.041	-0.091	-0.150	9	0.123	0.167	0.188	0.040	0.354	11	0.306	0.042	0.214	-0.014	0.045	13	-0.083	-0.080	-0.216	0.007	-0.023	5	-0.149	0.001	0.045	0.059	-0.062	9	206	23.0	5.36
IPI00133706.1	Ras-related protein Rab-1B	92.04%	8	7	27	504	0.165	-0.035	0.110	0.009	0.103	32	0.058	-0.028	0.043	0.011	0.073	24	0.203	0.072	0.163	0.007	0.088	34	-0.035	0.041	-0.089	0.041	-0.116	19	0.019	-0.074	-0.069	-0.127	-0.086	41	201	22.2	5.73
IPI00337980.5	Ras-related protein Rab-21	83.33%	2	9	31	203							0.205	-0.219	-0.427	-0.260	0.244	3	0.522	0.164	0.386	0.084	0.288	3	-0.029	0.043	-0.349	0.176	-0.855	5	0.128	0.089	0.029	0.095	0.051	7	222	24.1	7.94
IPI00621963.2	ras-related protein Rab-23	75.11%	2	4	27	164	-0.224	0.112	-0.180	0.036	-0.201	5	-0.175	-0.085	-0.452	-0.165	-0.292	5	0.205	0.151	0.079	0.023	0.058	4													237	26.8	7.30
IPI00120346.3	Ras-related protein Rab-27B	82.11%	2	3	22	75	0.720	0.360	0.414	-0.204	0.404	3																			0.481	0.256	0.165	-0.013	0.014	6	218	24.5	5.54
IPI00137227.1	Ras-related protein Rab-2A	98.11%	6	14	26	489	0.085	0.131	0.119	0.026	0.080	41	0.123	-0.016	0.037	-0.020	-0.059	18	0.156	0.108	-0.066	0.114	-0.071	32	0.156	0.077	0.096	0.020	0.051	27	0.066	-0.072	-0.080	-0.077	-0.039	38	212	23.5	6.54
IPI00331205.1	Ras-related protein Rab-30	91.13%	5	5	28	170																									-0.167	-0.276	-0.252	-0.057	-0.163	7	203	23.0	4.97
IPI00222632.3	ras-related protein Rab-31	82.56%	3	3	22	60	0.130	0.126	-0.006	0.026	-0.017	4																									195	21.4	7.40
IPI00556875.1	Ras-related protein Rab-34	69.11%	5	5	20	86													-0.167	-0.118	0.073	-0.233	-0.039	8	-0.051	-0.032	-0.044	0.043	-0.149	4							259	29.1	8.27
IPI00130489.1	Ras-related protein Rab-35	95.02%	8	8	29	387	0.128	0.092	0.134	0.131	0.321	13	0.004	0.004	0.089	0.021	0.016	12	0.365	0.094	0.144	-0.048	0.037	14	0.003	0.174	-0.048	0.081	-0.083	10	0.020	-0.017	-0.091	0.003	-0.048	22	201	23.0	8.29
IPI00122965.1	Ras-related protein Rab-3A	95.00%	11	4	26	264																			-0.523	-0.056	-0.621	0.013	-0.567	6	0.087	-0.105	-0.133	-0.108	-0.141	75	220	25.0	5.03
IPI00113112.3	Ras-related protein Rab-3B	89.95%	9	4	24	120																									0.016	0.047	-0.077	0.068	0.045	14	219	24.7	5.11
IPI00316495.3	Ras-related protein Rab-3C	90.31%	10	5	28	217																			-1.340	0.047	-1.673	0.027	-1.326	6	0.062	-0.136	-0.201	-0.122	-0.176	44	227	25.9	5.24
IPI00116688.1	Ras-related protein Rab-3D	86.76%	10	1	20	177																									0.011	-0.009	-0.201	-0.057	-0.184	17	219	24.4	4.93
IPI00271059.2	Ras-related protein Rab-4B	84.04%	9	3	28	102																									-0.167	-0.288	-0.432	0.044	-0.293	5	213	23.6	6.06
IPI00132410.1	Ras-related protein Rab-5A	98.14%	1	6	28	288	-0.158	-0.032	-0.178	0.014	-0.119	11	0.098	0.023	0.004	-0.134	0.077	8	-0.074	0.011	-0.188	-0.009	-0.022	13	-0.176	-0.022	-0.260	-0.293	-0.234	13	-0.072	-0.031	0.009	-0.097	-0.022	32	215	23.6	8.15
IPI00224518.2	Ras-related protein Rab-5C	96.76%	4	6	27	334	0.014	0.026	0.009	-0.032	-0.017	18	0.084	-0.016	0.280	-0.001	0.132	19	-0.050	0.096	0.010	0.020	0.037	23	-0.173	-0.092	-0.446	-0.029	-0.238	16	0.017	-0.074	0.061	-0.022	0.026	29	216	23.4	8.41
IPI00378145.1	Ras-related protein Rab-6B	92.31%	8	1	37	221	0.175	0.050	0.079	0.099	0.087	9	0.241	-0.057	0.070	0.019	0.141	8	0.165	0.056	0.090	-0.089	0.047	12	-0.154	-0.255	-0.407	-0.038	-0.184	4	-0.010	-0.150	-0.038	-0.139	-0.082	21	208	23.4	5.53
IPI00408892.2	Ras-related protein Rab-7a	90.82%	6	20	32	488	0.043	0.013	-0.041	0.007	0.021	32	0.370	0.096	0.306	0.083	0.106	32	0.327	0.055	0.322	0.024	0.102	38	0.279	0.077	0.166	0.055	0.174	21	-0.072	-0.044	-0.079	-0.059	-0.029	39	207	23.5	6.70
IPI00331128.3	Ras-related protein Rab-8A	87.92%	9	5	32	257	0.189	0.091	0.115	0.027	0.052	8	-0.010	-0.160	0.050	0.059	0.030	6	0.185	0.096	0.161	0.048	0.091	10	0.162	0.038	-0.071	0.061	-0.120	9	0.021	0.118	0.038	0.014	0.016	12	207	23.7	9.07
IPI00411115.1	Ras-related protein Rab-8B	90.34%	8	3	32	251	0.255	0.169	0.153	0.055	0.103	4	-0.059	-0.309	0.014	-0.091	0.006	4	0.207	0.096	0.147	0.033	0.038	7	0.184	0.210	-0.071	0.103	-0.123	5	0.021	-0.106	-0.160	0.014	-0.128	10	207	23.6	9.07
IPI00124282.1	Ras-related protein Ral-A	75.24%	1	6	20	225	0.089	-0.010	0.010	0.052	0.154	14	0.280	0.070	0.241	0.036	0.204	10	0.391	0.158	0.323	-0.020	0.262	19	-0.542	0.037	-0.720	0.040	-0.419	4	0.099	0.110	0.040	0.027	0.021	28	206	23.5	7.11
IPI00121110.1	Ras-related protein Ral-B	54.85%	2	3	20	126	-0.059	0.020	-0.133	-0.139	-0.085	10	-0.010	0.040	0.140	0.033	-0.123	6	0.096	0.092	0.212	0.119	-0.066	9							0.096	0.204	0.104	0.089	0.139	5	206	23.3	6.62
IPI00138406.1	Ras-related protein Rap-1A	96.20%	1	2	23	264	0.111	0.052	0.050	0.003	0.085	13	-0.347	-0.023	-0.154	-0.080	-0.253	10	0.229	0.106	0.262	0.013	0.140	12	-0.155	0.072	-0.167	0.037	-0.180	12	0.010	0.069	0.059	0.167	0.140	17	184	21.0	6.67
IPI00407954.2	Ras-related protein Rap-1b	96.74%	1	5	24	335	0.123	0.043	0.079	0.010	0.080	14	0.183	0.026	0.108	0.065	0.011	13	0.380	0.091	0.380	0.014	0.235	28	-0.252	0.081	-0.264	0.036	-0.290	23	0.058	0.051	0.064	0.058	0.021	20	184	20.8	5.78
IPI00138716.1	Ras-related protein Rap-2b	96.72%	2	4	16	121													0.380	0.111	0.344	0.041	0.102	5							0.119	0.083	-0.068	0.045	0.062	25	183	20.5	4.81
IPI00466588.3	Ras-related protein Rap-2c	91.80%	2	3	14	71													0.380	0.111	0.434	0.108	0.135	3							0.052	0.163	-0.068	0.045	0.050	15	183	20.7	4.94
IPI00323822.1	Ras-related protein R-Ras2	68.14%	2	9	20	177	0.024	0.079	0.017	-0.037	-0.020	16	-0.111	-0.213	-0.234	-0.186	-0.229	6	0.184	0.068	0.027	-0.025	-0.013	26													204	23.4	6.01
IPI00315463.4	receptor expression-enhancing protein 5	72.49%	1	8	15	204	0.200	0.339	0.203	0.195	0.276	4	0.244	0.061	0.238	0.036	0.264	16	0.535	0.060	0.361	-0.044	0.281	24	-0.496	-0.138	-0.602	0.013	-0.571	16	0.036	0.212	0.073	0.061	-0.003	15	189	21.4	8.12
IPI00944099.2	receptor-interacting serine/threonine-protein kinase 3 isoform 1	77.57%	3	7	31	102							0.336	-0.108	0.238	-0.147	0.578	17																			486	53.3	7.53
IPI00229935.3	Receptor-type tyrosine-protein phosphatase beta	75.93%	1	2	183	702							0.100	0.170	-0.142	0.095	0.202	4	0.238	0.086	0.089	-0.063	0.126	8													1998	224.4	7.18
IPI00750262.1	regulation of nuclear pre-mRNA domain-containing protein 1B	79.69%	6	6	35	244	-0.071	-0.006	0.031	0.292	0.390	4	-0.816	-0.311	-0.697	0.084	-1.224	9	-0.088	0.144	0.043	0.099	-0.071	3	-0.038	-0.029	0.214	-0.265	0.271	7							325	36.8	5.97
IPI00123762.2	regulator of chromosome condensation isoform 1	86.64%	3	11	42	353	0.219	0.099	0.210	0.073	0.099	23	0.099	-0.004	0.108	0.043	0.156	27	0.241	0.091	-0.086	0.170	0.034	11	-0.063	0.000	0.043	-0.038	-0.026	23							434	46.3	8.35
IPI00132450.1	Regulator of G-protein signaling 10	75.14%	1	2	20	68							-0.963	-0.293	-0.276	-0.053	-0.236	5																			181	21.1	6.81
IPI00265467.3	Regulator of G-protein signaling 6	65.89%	2	5	43	116																									0.048	0.048	0.157	0.098	0.066	10	472	54.5	7.42
IPI00118769.2	regulator of G-protein signaling 7 isoform 2	56.38%	5	5	36	95																									-0.176	-0.069	-0.029	-0.175	-0.120	8	470	55.0	7.85
IPI00678532.3	Regulator of microtubule dynamics protein 3	74.68%	1	6	41	88																			0.084	0.236	0.234	0.112	0.014	4							470	52.0	5.21
IPI00122845.11	remodeling and spacing factor 1	60.31%	1	5	118	304							-0.606	-0.358	-0.375	0.047	0.163	3																			1441	161.7	5.02
IPI00459331.2	Renin receptor	52.00%	1	3	20	100													-0.042	-0.368	-0.458	-0.819	-0.163	5	0.225	0.151	0.374	-0.094	0.316	4							350	39.1	5.54
IPI00124744.1	Replication factor C subunit 2	90.83%	1	8	42	131							-0.022	-0.006	-0.082	0.066	0.013	10	0.433	0.025	0.577	0.103	0.073	3													349	38.7	6.44
IPI00665571.2	Replication factor C subunit 3	86.24%	1	3	43	164	-0.051	-0.199	0.044	0.080	-0.189	3	-0.022	-0.265	0.131	0.337	-0.013	4																			356	40.5	8.38
IPI00319874.3	Replication factor C subunit 4	85.71%	4	4	37	79							-0.088	-0.245	0.263	0.073	-0.211	5																			364	39.8	6.70
IPI00132481.3	Replication factor C subunit 5	92.04%	2	5	40	134	0.065	-0.026	-0.042	0.126	-0.062	6	0.019	0.003	-0.056	-0.130	-0.165	9																			339	38.1	7.81
IPI00132128.1	Replication protein A 14 kDa subunit	86.78%	3	6	8	48	-0.152	-0.123	-0.183	-0.152	-0.340	4	-0.260	-0.128	-0.189	0.077	-0.276	8																			121	13.6	4.84
IPI00311131.1	Replication protein A 32 kDa subunit	65.19%	2	5	18	82							-0.604	-0.181	-0.322	0.038	-0.571	8																			270	29.7	6.15
IPI00124520.1	Replication protein A 70 kDa DNA-binding subunit	83.15%	2	11	62	215	-0.031	-0.025	-0.077	-0.007	0.229	3	-0.464	-0.038	-0.308	-0.060	-0.271	17	-0.276	-0.111	-0.274	-0.162	-0.020	5	0.138	0.212	0.241	0.024	0.176	6							623	69.0	7.91
IPI00462957.5	reproductive homeobox 2D	73.82%	2	2	10	19																			-0.739	0.135	-0.599	0.262	-0.108	4							191	21.1	4.70
IPI00137831.1	Reticulocalbin-1	80.92%	1	11	27	197							0.312	0.020	0.242	0.084	0.178	19	0.188	0.011	0.145	-0.196	0.047	25	0.473	-0.050	0.363	0.040	0.276	11							325	38.1	4.84
IPI00221798.1	Reticulocalbin-3	63.41%	4	10	15	105							-0.440	0.062	-0.351	0.195	-0.436	22							2.543	-0.213	2.326	-0.120	2.528	4							328	38.0	4.88
IPI00395193.1	Reticulon-1	62.18%	2	8	39	281	0.415	0.356	0.321	0.041	0.338	3																			-0.006	0.087	0.099	0.124	0.007	51	780	83.5	4.58
IPI00459442.1	reticulon-1 isoform RTN1-C	93.75%	2	2	23	181	0.415	0.356	0.321	0.041	0.338	3																			0.095	0.122	0.089	0.128	0.076	25	208	23.5	8.91
IPI00470167.3	reticulon-4 isoform B1	74.72%	3	1	23	412	-0.060	0.088	-0.050	0.020	-0.167	36	0.610	0.045	0.468	0.154	0.592	16	0.230	0.040	0.171	-0.168	0.088	53	-0.199	-0.031	-0.240	0.030	-0.201	44	-0.006	0.036	0.023	0.154	0.048	15	356	38.4	4.78
IPI00626662.3	Retinal dehydrogenase 1	71.26%	4	11	41	140	0.565	0.009	0.733	-0.065	0.824	11																			-0.051	0.058	0.289	0.044	0.147	12	501	54.4	7.80
IPI00321753.7	Retinoic acid-induced protein 3	41.29%	1	5	17	108	0.007	-0.057	-0.080	-0.028	0.061	11	-0.097	-0.031	-0.048	-0.007	0.072	8	0.530	0.087	0.452	0.099	0.474	12													356	40.1	7.62
IPI00310323.2	Retinoid-inducible serine carboxypeptidase	46.90%	3	9	26	155	0.112	0.124	0.010	-0.038	0.207	9	-0.069	-0.047	0.120	0.099	0.188	9	0.257	0.050	0.163	0.160	0.171	10													452	50.9	5.66
IPI00896013.2	Rfc1 protein	81.80%	5	11	139	421	0.036	-0.012	-0.060	-0.031	-0.074	7	0.051	0.103	0.072	0.047	0.041	12																			1132	126.0	9.35
IPI00322312.3	Rho GDP-dissociation inhibitor 1	96.57%	1	14	32	507	0.103	-0.186	0.246	-0.032	0.280	38	-0.136	-0.079	0.004	-0.125	0.077	40	-0.357	-0.253	-0.390	0.127	-0.165	59	-0.112	0.084	-0.267	0.037	-0.221	35	0.030	0.059	-0.009	-0.042	-0.032	54	204	23.4	5.20
IPI00122568.3	Rho GDP-dissociation inhibitor 2	80.00%	2	6	17	93							0.157	0.007	0.296	-0.086	0.505	20																			200	22.8	5.11
IPI00404970.2	Rho GTPase-activating protein 1	65.83%	3	8	33	174	0.252	-0.065	0.547	0.418	0.879	4	0.087	-0.050	0.092	-0.057	0.252	12	-0.504	-0.130	-0.413	0.135	-0.099	7	-0.125	0.174	-0.300	-0.228	-0.431	5	0.241	-0.320	-0.436	-0.434	-0.388	16	439	50.4	6.44
IPI00480321.2	Rho GTPase-activating protein 35	59.04%	2	6	104	237																									-0.029	0.197	0.085	-0.095	0.017	5	1499	170.3	6.61
IPI00881406.1	rho guanine nucleotide exchange factor 1 isoform b	75.61%	7	8	97	326	0.120	-0.120	0.066	-0.050	0.169	10	-0.106	0.072	0.437	0.466	0.084	3																			976	108.6	6.09
IPI00678935.4	Rho guanine nucleotide exchange factor 16	63.81%	5	6	53	135	-0.068	-0.062	-0.091	-0.038	0.026	10																									713	80.3	7.49
IPI00319986.2	Rhomboid domain-containing protein 1	62.54%	1	1	13	46							0.427	0.050	-0.072	0.099	0.591	3																			315	35.8	9.09
IPI00404182.2	Rho-related GTP-binding protein RhoB	95.41%	1	4	22	149	-0.094	-0.154	0.001	-0.044	0.017	12	0.061	-0.208	-0.150	-0.189	0.145	5	0.023	0.074	-0.006	0.111	-0.149	9							0.040	-0.042	-0.083	-0.097	0.014	27	196	22.1	5.24
IPI00122245.2	Rho-related GTP-binding protein RhoC	69.95%	1	3	18	185	-0.089	-0.120	0.075	-0.003	0.007	23	0.246	-0.066	0.047	-0.004	0.234	12	0.023	0.067	-0.171	0.040	-0.059	15							0.033	0.050	-0.152	-0.100	-0.092	19	193	22.0	6.58
IPI00116558.1	Rho-related GTP-binding protein RhoG	89.01%	7	8	26	333	0.153	-0.004	0.166	-0.080	0.200	30	0.038	0.287	0.251	0.279	0.047	19	0.044	-0.053	-0.044	0.024	-0.020	17							-0.068	0.281	0.157	0.349	0.092	21	191	21.3	8.12
IPI00229787.3	Ribonuclease H2 subunit A	82.39%	3	5	22	66							0.332	-0.065	0.597	-0.329	0.456	5																			301	33.5	4.98
IPI00458223.2	Ribonuclease H2 subunit B	74.03%	2	4	34	119							0.961	-0.095	0.952	0.280	-0.001	3																			308	34.7	9.45
IPI00955744.1	ribonuclease inhibitor isoform b	91.26%	2	22	63	658	0.208	-0.066	0.281	0.019	0.305	50	0.059	0.059	0.231	-0.007	0.140	46	-0.320	-0.166	-0.311	0.136	-0.173	64	-0.059	0.070	0.111	-0.038	0.010	6	-0.059	-0.047	-0.112	-0.211	0.003	18	492	53.9	5.01
IPI00134346.1	Ribonuclease P protein subunit p30	87.31%	1	2	28	66																			0.308	-0.034	0.107	0.099	0.055	4							268	29.5	9.06
IPI00625328.2	Ribonuclease T2	69.50%	1	2	15	50							0.199	0.064	-0.346	-0.094	-0.029	4																			259	29.6	6.37
IPI00130640.5	Ribonuclease UK114	76.30%	1	7	11	69	0.346	-0.071	0.409	-0.086	0.348	3																			0.044	0.054	-0.004	0.017	-0.025	24	135	14.2	8.68
IPI00315127.2	Ribonucleoside-diphosphate reductase large subunit	94.44%	1	32	82	1014	-0.045	-0.023	0.091	0.037	0.103	43	-0.513	0.088	-0.468	0.089	-0.525	67	-0.265	0.067	-0.080	-0.099	-0.109	9	0.441	0.045	0.590	0.070	0.441	33							792	90.2	6.70
IPI00112645.1	Ribonucleoside-diphosphate reductase subunit M2	76.67%	2	3	35	138	-0.498	-0.074	-0.529	-0.138	-0.597	5													0.445	0.161	0.525	0.055	0.258	3							390	45.1	5.45
IPI00653091.1	Ribose-phosphate pyrophosphokinase	90.25%	6	10	32	161	-0.206	-0.057	-0.209	-0.057	-0.265	4													-0.276	0.163	-0.478	0.095	-0.383	13	-0.047	-0.042	-0.039	-0.103	0.115	12	318	34.8	6.98
IPI00473532.1	Ribosomal protein L15	94.12%	10	10	41	463	0.027	-0.034	0.161	0.108	0.077	43	0.343	0.143	0.264	0.153	0.058	24	-0.065	0.040	-0.057	0.004	-0.062	49	-0.068	-0.059	-0.038	-0.041	0.222	30	-0.224	0.031	-0.079	0.000	-0.019	13	204	24.1	11.58
IPI00125145.3	Ribosomal protein S6 kinase alpha-2	62.62%	15	2	63	164	0.091	-0.320	-0.023	0.007	0.174	5	0.087	-0.007	0.161	0.123	-0.063	3																			733	83.1	8.50
IPI00114333.2	Ribosomal protein S6 kinase alpha-3	82.57%	15	5	69	234	-0.035	0.031	0.006	-0.032	0.087	9	0.070	-0.010	0.161	0.147	-0.063	11							0.073	0.217	0.075	0.247	-0.130	5							740	83.6	6.89
IPI00131006.2	Ribosomal protein S6 kinase alpha-4	65.59%	3	7	62	138	-0.023	0.075	0.006	0.066	-0.163	7																									773	85.6	8.27
IPI00127554.1	Ribosomal RNA small subunit methyltransferase NEP1	89.34%	1	4	25	55	-0.032	-0.097	0.014	0.080	0.108	3																									244	27.0	8.91
IPI00133594.3	Ribosomal RNA-processing protein 7 homolog A	66.07%	4	2	22	48	-0.042	-0.022	-0.007	-0.057	-0.103	6																									280	32.4	9.48
IPI00653834.2	ribosomal RNA-processing protein 8 isoform 1	66.00%	2	5	40	108							-0.781	-0.281	-0.454	-0.472	-0.335	5																			503	56.5	9.95
IPI00387354.5	Ribosome biogenesis protein BOP1	67.76%	2	13	54	170	-0.454	-0.218	-0.209	-0.139	-0.099	8							0.062	0.028	0.103	0.051	0.136	13	-0.062	0.079	0.055	-0.035	0.147	11							732	82.5	6.28
IPI00130436.5	Ribosome biogenesis protein BRX1 homolog	66.57%	4	7	37	145	-0.082	0.069	-0.031	0.041	-0.219	5							-0.031	-0.008	-0.194	-0.039	-0.119	5	0.106	-0.028	-0.029	-0.022	0.003	13							353	41.2	10.02
IPI00121562.1	Ribosome biogenesis protein WDR12	79.91%	2	9	37	157	-0.250	0.044	-0.089	0.045	-0.189	12							-0.306	-0.160	-0.302	-0.044	-0.155	6	0.073	-0.006	-0.141	0.016	0.051	6							423	47.3	5.60
IPI00318725.4	Ribosome biogenesis regulatory protein homolog	80.27%	1	15	51	239	-0.150	-0.203	-0.243	-0.038	-0.051	11	-0.130	-0.057	-0.175	-0.146	0.027	13	-0.176	0.078	-0.080	-0.175	-0.020	13	0.061	-0.031	0.120	-0.045	0.013	24							365	41.5	10.77
IPI00136151.4	Ribosome maturation protein SBDS	91.20%	2	11	36	148							-0.136	-0.003	-0.100	-0.108	0.153	15	-0.088	0.057	0.158	0.007	-0.092	12	0.316	0.111	-0.039	0.069	-0.130	7							250	28.8	8.76
IPI00121159.3	Ribosome production factor 2 homolog	71.90%	2	7	36	105																			0.123	-0.139	0.116	0.024	0.077	13							306	35.3	10.04
IPI00624030.3	Ribulose-phosphate 3-epimerase	54.89%	3	3	17	73	-0.112	-0.012	-0.154	0.037	0.233	4	-0.201	-0.139	-0.353	-0.167	-0.231	5																			235	25.6	5.81
IPI00110315.2	RNA 3'-terminal phosphate cyclase	90.98%	3	7	33	122	0.019	-0.079	0.099	0.033	0.016	4	-0.355	0.085	-0.415	0.066	-0.128	9																			366	39.2	7.90
IPI00136573.1	RNA binding motif 31, Y-linked	72.57%	2	1	43	205							-0.664	-0.178	-1.165	-0.004	-1.396	3							0.381	0.212	0.541	0.127	0.348	6	0.081	0.215	0.214	-0.167	0.135	5	565	62.5	8.31
IPI00124979.2	RNA binding motif protein, X-linked	93.61%	6	2	71	741	0.069	0.076	0.130	0.007	0.154	46	0.225	0.123	0.209	0.044	0.233	24	0.240	0.094	0.179	-0.047	0.144	48	0.119	0.065	0.172	0.043	0.057	50	0.006	0.123	0.131	0.138	0.096	35	391	42.3	10.05
IPI00331654.1	RNA polymerase II-associated factor 1 homolog	65.79%	1	5	43	131	-0.082	-0.056	-0.183	0.041	-0.157	6																									535	60.5	4.65
IPI00109401.1	RNA polymerase II-associated protein 3	74.39%	1	12	69	298	-0.308	-0.114	-0.283	-0.086	-0.311	14	-0.180	-0.009	-0.438	0.375	-0.730	3							-0.141	-0.044	0.077	-0.006	0.052	13							660	74.1	7.99
IPI00480480.2	RNA-binding motif, single-stranded-interacting protein 1 isoform 1	73.38%	17	9	36	310	-0.194	0.007	-0.092	0.021	-0.123	8	0.202	0.016	0.014	0.066	0.066	15	0.104	0.016	0.029	-0.094	-0.012	14	-0.038	0.108	-0.088	0.020	0.001	7							417	45.0	8.97
IPI00322263.2	RNA-binding protein 12	43.65%	3	5	44	157	0.065	0.171	0.209	-0.016	0.124	9	-0.116	-0.076	-0.292	-0.176	-0.204	6							0.481	0.165	0.480	0.066	0.497	3							992	102.7	8.32
IPI00229472.6	RNA-binding protein 28	79.87%	2	10	105	365	0.061	0.043	-0.257	0.009	-0.091	5	0.073	0.079	-0.079	0.089	-0.281	8	0.083	0.054	0.229	0.175	0.192	8	0.007	-0.051	0.013	-0.062	0.062	7							750	84.2	9.54
IPI00225915.2	RNA-binding protein 34	75.11%	2	7	46	219							-0.243	-0.269	-0.238	-0.170	-0.111	3							-0.022	-0.056	0.044	0.034	-0.116	8							442	49.1	10.01
IPI00133061.3	RNA-binding protein 7	79.25%	2	4	29	91							0.178	-0.246	-0.065	-0.204	-0.228	4																			265	30.1	9.38
IPI00225974.1	RNA-binding protein NOB1	65.51%	3	4	39	119	0.516	0.152	0.175	0.084	0.435	3													0.170	0.149	0.100	-0.017	-0.056	7							403	45.4	7.31
IPI00131909.1	RNA-binding protein PNO1	85.89%	1	4	38	155	0.163	-0.060	0.188	0.001	0.103	7													0.118	-0.086	0.054	-0.050	0.172	4							248	27.4	9.79
IPI00464101.2	Roquin	59.73%	1	1	71	146	0.058	0.023	-0.056	0.038	-0.028	3																									1130	125.3	7.27
IPI00119581.2	rRNA 2'-O-methyltransferase fibrillarin	85.32%	2	13	45	505	-0.103	0.110	-0.125	0.033	-0.139	27	0.148	0.007	0.073	0.010	0.020	23	0.119	0.077	0.118	-0.095	0.038	30	0.166	0.020	0.230	0.020	0.184	45							327	34.3	10.24
IPI00420344.1	RRP12-like protein	78.22%	1	18	112	472	0.065	-0.027	0.098	-0.105	0.102	5	0.038	0.000	-0.238	-0.127	-0.013	15	0.115	0.044	0.500	0.361	0.070	5	-0.317	-0.053	-0.470	0.050	-0.293	27							1295	143.0	8.91
IPI00458958.2	RRP15-like protein	57.65%	1	4	33	94													-0.088	-0.131	-0.181	0.030	-0.122	3	-0.077	0.040	-0.039	-0.020	-0.248	3							281	31.0	8.76
IPI00133985.1	RuvB-like 1	86.40%	3	21	56	487	-0.152	0.048	-0.111	-0.004	-0.184	37	-0.106	-0.038	-0.223	-0.026	-0.108	55	0.136	-0.024	0.099	-0.009	0.088	27	0.303	0.080	0.316	0.007	0.335	38							456	50.2	6.42
**IPI00123557.3**	**RuvB-like 2**	**85.96%**	**2**	**17**	**61**	**532**	-0.125	0.010	-0.136	0.011	-0.077	**27**	-0.100	0.037	0.017	-0.039	0.003	**28**	0.154	0.035	0.132	-0.111	0.099	**26**	0.043	0.019	0.179	-0.016	0.156	**33**	-0.088	0.095	0.124	-0.198	-0.105	**4**	**463**	**51.1**	**5.64**
IPI00132659.1	RWD domain-containing protein 1	45.68%	3	5	10	66	-0.226	-0.152	-0.300	-0.042	-0.144	6													0.282	-0.054	0.016	-0.037	0.044	11							243	27.8	4.26
IPI00131834.1	RWD domain-containing protein 4	71.81%	1	3	11	49							-0.133	-0.074	-0.427	-0.194	-0.136	6																			188	21.1	5.63
IPI00454016.5	S-adenosylmethionine synthase isoform type-2	83.80%	5	15	39	293	-0.086	-0.076	-0.066	-0.037	0.089	12	-0.089	-0.037	-0.103	0.004	-0.014	24	-0.317	-0.171	-0.062	0.104	0.017	14	0.179	-0.082	0.095	0.037	0.027	18	-0.091	0.127	0.068	0.001	0.077	6	395	43.7	6.48
IPI00875198.1	SAM domain and HD domain-containing protein 1 isoform 1	74.01%	6	18	57	228							-1.032	0.006	-0.919	0.054	-0.977	46																			658	75.8	7.93
IPI00133801.3	SAP domain-containing ribonucleoprotein	89.52%	1	8	35	212	-0.097	0.080	-0.181	-0.056	-0.219	10	-0.189	-0.012	-0.128	-0.100	-0.208	8	0.325	0.104	0.094	0.044	0.185	16	-0.095	-0.231	-0.045	-0.112	-0.063	18							210	23.5	6.65
IPI00944159.1	scaffold attachment factor B	78.12%	5	14	106	705	-0.007	0.006	-0.039	-0.047	-0.059	22	-0.216	0.036	-0.128	0.066	-0.191	52	0.061	0.060	0.045	-0.136	-0.060	34	0.110	0.003	0.143	0.017	0.007	42	-0.407	0.001	0.075	-0.034	-0.213	11	937	105.0	5.35
IPI00857736.1	Scaffold attachment factor B2	76.08%	7	3	96	475	-0.077	0.027	-0.032	0.016	0.009	17	-0.186	0.176	-0.109	0.190	-0.131	19	0.102	0.063	0.118	-0.106	0.001	19	0.112	0.011	0.190	0.021	0.061	18	-0.521	0.001	0.087	-0.034	-0.136	7	991	111.8	6.38
IPI00407413.2	SCAN domain containing 3	79.63%	5	2	72	303	0.352	0.010	0.306	0.011	0.272	8	0.136	-0.095	0.308	0.072	0.233	9	-0.236	-0.074	0.007	-0.060	-0.069	3	0.076	0.157	0.273	-0.039	0.264	7							756	84.2	8.44
IPI00459280.2	Scavenger mRNA-decapping enzyme DcpS	72.49%	3	9	37	238	-0.184	-0.007	-0.017	0.075	-0.106	16	0.021	-0.065	0.244	-0.001	0.089	17							0.017	0.103	-0.006	0.051	0.033	24	-0.271	0.072	0.095	-0.288	-0.037	5	338	39.0	6.48
IPI00129174.6	schlafen 4	58.31%	2	2	44	113																			0.214	-0.042	0.079	-0.039	0.089	3							602	67.9	7.97
IPI00275753.1	Scr3	73.88%	3	9	41	323	-0.184	0.044	-0.233	0.081	-0.209	11	-0.026	0.021	0.081	-0.279	-0.299	8	0.058	0.056	-0.050	-0.109	-0.094	23													356	37.9	8.85
IPI00114818.1	SEC14-like protein 2	75.93%	1	5	30	79																									-0.142	-0.060	-0.100	-0.068	-0.154	9	403	46.3	7.11
IPI00648142.2	SEC23-interacting protein	50.10%	2	16	72	320	0.030	-0.014	0.045	-0.014	0.024	24	-0.020	-0.037	0.023	0.073	0.009	16	-0.236	-0.136	-0.315	0.003	0.057	4	0.190	-0.038	0.098	0.062	-0.035	7							998	110.7	5.72
IPI00626620.2	Sec24 related gene family, member C isoform 2	63.73%	2	9	80	310	0.214	0.120	0.242	-0.133	0.091	4	-0.213	0.072	0.055	-0.069	-0.097	3	0.066	0.092	0.124	-0.039	0.023	3	-0.062	-0.092	0.141	-0.094	-0.050	8							1020	111.5	6.74
IPI00284595.8	SEC24 related gene family, member D	46.12%	1	10	52	240							-0.304	0.166	-0.311	-0.034	-0.224	27																			1032	112.6	7.18
IPI00112536.1	Secernin-1	53.14%	2	9	31	168							1.040	0.100	1.195	0.147	0.832	6							-0.125	0.076	-0.302	0.041	-0.051	12	0.036	0.228	0.069	0.040	0.119	24	414	46.3	4.79
IPI00137758.5	Secernin-3	55.74%	1	4	25	81																									0.433	0.153	0.061	0.099	0.459	6	418	47.6	5.66
IPI00133418.1	Secretogranin-2	87.03%	2	20	75	440																			-0.617	-0.028	-0.690	-0.059	-0.687	41	0.136	-0.336	-0.240	-0.297	-0.240	51	617	70.6	4.75
IPI00944768.1	secretogranin-3 isoform 2 precursor	82.62%	2	5	35	101																			-0.902	-0.125	-1.059	0.007	-0.974	4							466	52.8	4.91
IPI00278804.1	Secretory carrier-associated membrane protein 1	67.75%	3	8	24	159							-0.189	-0.031	-0.304	0.023	-0.405	3							-0.362	0.102	-0.317	-0.051	-0.421	6	0.036	-0.186	-0.125	-0.226	-0.168	20	338	38.0	7.71
IPI00122865.1	Secretory carrier-associated membrane protein 5	31.06%	1	4	6	49																									0.118	-0.173	-0.138	-0.080	-0.120	13	235	26.1	8.54
IPI00121386.2	Segment polarity protein dishevelled homolog DVL-2	59.10%	4	2	36	108																			0.139	0.094	0.318	-0.014	0.072	3							736	78.8	5.96
IPI00221759.1	Selenide, water dikinase 1	67.35%	1	7	27	182	0.034	0.004	0.245	-0.206	-0.034	6	-0.238	-0.035	-0.285	-0.044	-0.155	7	-1.146	-0.693	-1.020	0.031	0.181	3	0.244	-0.010	0.103	-0.038	0.158	13							392	42.9	5.97
IPI00623845.3	Selenium-binding protein 1	74.58%	6	4	35	79																									-0.204	-0.122	-0.157	0.156	0.048	6	472	52.5	6.29
IPI00310205.2	Selenocysteine-specific elongation factor	84.22%	2	4	52	226							-0.432	0.573	0.016	0.276	-0.158	3							-0.103	0.088	0.134	-0.095	0.392	4							583	63.5	8.29
IPI00222578.6	Selenoprotein W	92.05%	1	3	9	15																									0.107	0.138	0.092	0.271	0.225	4	88	9.6	8.57
IPI00129164.1	sepiapterin reductase	93.51%	4	12	34	299	-0.123	-0.138	-0.059	-0.111	-0.108	23	-0.083	-0.196	-0.276	-0.214	0.094	5	0.147	-0.293	-0.233	0.061	0.089	5	-0.066	-0.048	0.114	0.148	0.119	3	-0.019	0.102	0.081	-0.051	0.085	41	262	27.9	6.21
IPI00114945.1	Septin-2	78.67%	9	18	37	647	0.048	0.016	0.076	-0.006	-0.009	56	0.029	-0.003	0.065	-0.029	-0.022	50	0.036	-0.087	0.009	0.139	0.065	22	0.244	0.095	0.299	0.011	0.189	40	-0.079	-0.047	-0.155	-0.111	-0.091	27	361	41.5	6.55
IPI00224626.3	septin-7	85.13%	5	28	59	930	0.000	0.036	0.033	0.006	-0.032	42	0.099	0.037	0.153	0.007	0.037	61	0.120	-0.034	0.144	0.050	0.100	35	-0.053	0.014	-0.063	0.041	-0.094	44	0.089	0.178	0.153	0.132	0.034	115	437	50.6	8.57
IPI00115682.1	serine (or cysteine) proteinase inhibitor, clade B, member 9	57.49%	6	4	26	86							-0.091	0.305	0.038	0.157	0.064	4																			374	42.2	5.40
IPI00454008.1	Serine hydroxymethyltransferase	95.24%	1	28	66	939	-0.085	-0.014	-0.165	-0.035	-0.138	45	0.544	0.143	0.582	0.212	0.346	59	0.239	0.126	0.174	0.004	0.033	56	0.066	-0.009	-0.010	-0.053	0.066	69							504	55.7	8.46
IPI00137465.1	Serine incorporator 1	49.89%	2	5	21	114	-0.077	0.026	-0.059	-0.097	-0.146	6							-0.608	0.033	-0.267	0.026	-0.425	6													453	50.5	6.28
IPI00315983.2	Serine palmitoyltransferase 1	63.00%	2	4	28	60	0.521	-0.082	0.301	0.029	0.526	4																									473	52.5	6.40
IPI00128040.2	Serine protease HTRA1	83.54%	1	4	44	177							-1.012	-0.229	-0.438	-0.201	-0.302	3																			480	51.2	7.65
IPI00275992.3	Serine protease HTRA2, mitochondrial	68.12%	5	6	33	103							0.014	0.208	0.175	-0.112	-0.278	5							-0.252	-0.170	-0.476	0.011	-0.454	5							458	49.3	9.60
IPI00131830.1	Serine protease inhibitor A3K	75.36%	7	11	29	114																									0.034	0.050	-0.083	0.057	0.163	30	418	46.8	5.16
IPI00139364.1	Serine/arginine-rich splicing factor 4	77.10%	3	2	78	212	-0.001	0.013	0.122	-0.074	0.163	10							0.100	0.065	-0.014	-0.012	0.029	8	-0.007	0.003	0.123	0.004	0.271	13							489	55.9	11.40
IPI00988192.1	serine/arginine-rich splicing factor 7 isoform 2	90.64%	7	8	47	242	-0.035	0.043	-0.051	-0.007	-0.176	10	-0.133	0.072	-0.094	0.140	-0.163	11	0.112	0.074	0.103	-0.157	0.111	15	-0.160	-0.041	0.079	-0.100	0.006	6	-0.074	0.066	0.019	0.066	-0.083	11	235	26.9	11.85
IPI00132340.1	Serine/arginine-rich splicing factor 9	70.72%	2	6	22	104	0.199	0.068	0.198	0.010	0.194	3	0.066	0.134	0.010	0.003	-0.089	14							-0.706	-0.394	-0.112	-0.072	-0.031	6							222	25.6	8.65
IPI00115321.4	Serine/threonine-protein kinase 10	76.50%	3	4	98	299							0.249	0.084	0.299	-0.219	0.158	4	-0.366	-0.130	-0.077	0.267	-0.042	3													966	111.8	7.15
IPI00116065.3	Serine/threonine-protein kinase 24	80.28%	3	7	37	191	0.154	0.016	0.179	0.027	0.267	15	0.084	0.138	0.114	0.052	0.023	8	0.014	-0.086	-0.051	-0.193	-0.106	3													431	47.9	5.43
IPI00118122.2	Serine/threonine-protein kinase 31	76.42%	4	4	97	310							-0.773	0.131	-0.468	0.004	-0.602	3																			1018	114.9	5.39
IPI00126943.1	Serine/threonine-protein kinase 38	58.92%	1	4	30	56	-0.083	-0.086	-0.123	-0.162	0.011	3																									465	54.1	7.34
IPI00114613.4	Serine/threonine-protein kinase MRCK beta	71.04%	7	12	146	419	0.292	0.163	0.262	0.114	0.333	18	0.376	-0.290	0.147	-0.218	0.455	5																			1713	194.6	6.46
IPI00115494.1	Serine/threonine-protein kinase MST4	73.08%	2	1	33	179	0.095	-0.085	0.077	-0.060	0.119	4	0.084	0.111	0.122	0.151	0.066	5	0.014	-0.076	-0.082	0.040	0.103	3													416	46.6	5.24
IPI00169493.1	Serine/threonine-protein kinase N3	51.82%	2	1	57	131							-0.063	0.081	0.242	0.026	0.089	3																			878	97.8	8.79
IPI00132993.1	Serine/threonine-protein kinase PAK 1	66.24%	2	8	54	451	0.047	-0.163	0.239	-0.039	0.165	17	-0.517	-0.165	-0.299	-0.048	-0.502	9	-0.436	-0.145	-0.409	-0.074	-0.311	19	0.143	0.004	0.140	-0.019	-0.086	4	0.001	-0.007	0.017	-0.133	-0.063	42	545	60.7	5.74

IPI00229884.1	Serine/threonine-protein kinase PAK 2	80.15%	1	8	56	460	-0.088
IPI00830990.2	serine/threonine-protein kinase PAK 3 isoform A	66.21%	6	1	52	380	-0.077
IPI00467379.2	Serine/threonine-protein kinase PAK 4	74.20%	1	4	46	136	-0.189
IPI00320690.2	Serine/threonine-protein kinase PRP4 homolog	63.65%	2	12	120	409	
IPI00387234.2	Serine/threonine-protein kinase SRPK1	74.85%	2	8	43	219	-0.231
IPI00124856.5	Serine/threonine-protein kinase TBK1	77.50%	1	1	53	119	
IPI00970148.1	Serine/threonine-protein phosphatase	58.40%	5	4	25	136	
IPI00667471.1	Serine/threonine-protein phosphatase 2A 55 kDa regulatory subunit B alpha	83.89%	10	12	44	200	0.054
IPI00224697.1	Serine/threonine-protein phosphatase 2A 56 kDa regulatory subunit epsilon	80.30%	2	7	41	112	0.320
IPI00408059.5	serine/threonine-protein phosphatase 2A 56 kDa regulatory subunit gamma	75.05%	5	4	52	220	0.120
IPI00310091.8	Serine/threonine-protein phosphatase 2A 65 kDa regulatory subunit A alpha	76.06%	3	22	59	851	0.043
IPI00118723.3	Serine/threonine-protein phosphatase 2A activator	91.33%	5	10	29	210	-0.080
IPI00120374.1	Serine/threonine-protein phosphatase 2A catalytic subunit alpha isoform	76.05%	2	1	28	321	-0.012
IPI00111556.1	Serine/threonine-protein phosphatase 2A catalytic subunit beta isoform	75.08%	3	2	26	340	-0.012
IPI00109415.1	Serine/threonine-protein phosphatase 4 catalytic subunit	66.78%	1	3	23	73	
IPI00969824.1	serine/threonine-protein phosphatase 4 regulatory subunit 1 isoform b	67.45%	7	6	63	135	
IPI00756696.2	Serine/threonine-protein phosphatase 4 regulatory subunit 2	84.89%	2	10	39	193	0.026
IPI00930798.1	serine/threonine-protein phosphatase 4 regulatory subunit 3A isoform 2	83.91%	5	9	75	231	-0.127
IPI00119180.1	Serine/threonine-protein phosphatase 5	73.95%	2	14	47	345	-0.068
IPI00132965.1	Serine/threonine-protein phosphatase 6 catalytic subunit	65.25%	1	3	21	51	
IPI00380742.3	Serine/threonine-protein phosphatase 6 regulatory subunit 1	55.02%	2	3	34	97	
IPI00130185.1	Serine/threonine-protein phosphatase PP1-alpha catalytic subunit	85.45%	2	4	30	320	-0.017
IPI00311873.5	Serine/threonine-protein phosphatase PP1-beta catalytic subunit	90.52%	1	3	30	244	0.021
IPI00918973.1	Serine-threonine kinase receptor-associated protein	91.43%	1	16	29	370	-0.012
IPI00139788.2	Serotransferrin	79.05%	7	2	63	220	
IPI00944143.1	serpin B6 isoform a	96.24%	11	23	47	619	0.166
IPI00114733.2	Serpin H1	87.29%	1	26	52	823	0.098
IPI00969867.1	Serpina1a protein	57.63%	4	5	25	129	
IPI00131695.3	Serum albumin	80.26%	4	34	65	477	
IPI00135660.5	Serum deprivation-response protein	97.85%	1	11	54	548	0.305
IPI00310567.2	Serum paraoxonase/arylesterase 2	50.56%	2	3	16	51	
IPI00121114.2	Serum paraoxonase/lactonase 3	77.68%	1	4	22	82	
IPI00395196.5	Serum response factor-binding protein 1	80.05%	2	3	59	191	-0.170
IPI00225307.1	seryl-tRNA synthetase, cytoplasmic isoform 1	85.45%	4	28	71	908	0.038
IPI00828824.1	set1/Ash2 histone methyltransferase complex subunit ASH2 isoform b	83.90%	5	6	50	176	
IPI00109142.4	S-formylglutathione hydrolase	96.45%	3	12	36	397	-0.214
IPI00465999.5	SH3 and multiple ankyrin repeat domains protein 1	42.09%	3	11	121	354	
IPI00351827.6	SH3 and multiple ankyrin repeat domains protein 3	57.12%	4	6	97	216	
IPI00122265.1	SH3 domain-binding glutamic acid-rich-like protein	100.00%	1	8	16	226	0.193
IPI00127358.1	SH3 domain-binding glutamic acid-rich-like protein 3	62.37%	2	5	18	193	0.034
IPI00405836.2	SH3 domain-binding protein 4	69.33%	1	2	79	189	0.076
IPI00623241.3	SH3-domain GRB2-like interacting protein 1 alpha	62.76%	8	14	53	169	
IPI00119842.1	Short/branched chain specific acyl-CoA dehydrogenase, mitochondria	94.68%	2	7	44	200	0.083
IPI00116591.2	Short-chain specific acyl-CoA dehydrogenase, mitochondrial	86.65%	2	12	43	242	0.279
IPI00380793.1	Shugoshin-like 2	77.58%	7	2	143	349	
IPI00411164.4	sickle tail protein isoform c	82.17%	11	8	227	844	-0.119
IPI00115454.3	Sideroflexin-1	94.72%	1	10	38	358	0.444
IPI00221602.1	Sideroflexin-5	69.30%	3	11	23	121	
IPI00228236.6	Signal peptidase complex subunit 2	72.12%	1	10	27	205	-0.204
IPI00420727.5	signal peptidase complex subunit 3	71.11%	1	3	13	81	0.151
IPI00132418.1	Signal recognition particle 14 kDa protein	78.18%	2	4	16	52	-0.019
IPI00877257.1	signal recognition particle 19 kDa protein	87.50%	3	5	18	71	
IPI00222977.2	Signal recognition particle 68 kDa protein	80.80%	2	20	85	552	0.019
IPI00316133.7	signal recognition particle 72	93.44%	2	20	80	448	0.245
IPI00119695.1	Signal recognition particle receptor subunit alpha	86.79%	2	17	75	377	-0.111
IPI00323360.2	Signal transducer and transcription activator 6	54.24%	1	2	58	151	0.019
IPI00408243.1	single-stranded DNA-binding protein, mitochondrial isoform 1	94.59%	3	6	21	129	0.034
IPI00669709.3	Sister chromatid cohesion protein PDS5 homolog A	76.95%	2	13	141	504	0.106
IPI00319160.6	Sjoegren syndrome/scleroderma autoantigen 1 homolog	83.92%	2	3	15	144	0.037
IPI00128415.4	Slit homolog 2 protein	74.16%	8	5	133	512	
IPI00676293.3	SLIT-ROBO Rho GTPase-activating protein 2	72.46%	2	8	97	342	-0.127
IPI00330039.3	SLIT-ROBO Rho GTPase-activating protein 3	65.61%	4	9	91	246	
IPI00127941.1	Small acidic protein	63.54%	3	5	15	159	-0.048
IPI00654219.2	small G protein signaling modulator 1 isoform b	52.39%	4	3	66	159	
IPI00943994.1	Small nuclear ribonucleoprotein F	97.67%	1	4	8	76	0.147
IPI00322749.3	Small nuclear ribonucleoprotein Sm D1	86.55%	2	5	16	224	-0.147
IPI00119220.1	Small nuclear ribonucleoprotein Sm D2	94.07%	5	10	25	342	-0.091
IPI00119224.1	Small nuclear ribonucleoprotein Sm D3	98.41%	1	5	21	132	0.092
IPI00114052.1	Small nuclear ribonucleoprotein-associated protein B	96.10%	2	7	28	212	-0.047
IPI00850851.1	small subunit processome component 20 homolog	69.52%	1	14	257	811	-0.108
IPI00988313.1	small ubiquitin-related modifier 2-like	42.42%	5	1	10	183	0.029
IPI00132096.3	S-methyl-5'-thioadenosine phosphorylase	97.88%	1	14	34	273	-0.264
IPI00317298.2	SNW domain-containing protein 1	84.70%	2	10	71	265	-0.074
IPI00227928.3	Sodium- and chloride-dependent GABA transporter 1	33.89%	3	8	13	74	
IPI00314054.3	Sodium- and chloride-dependent taurine transporter	28.99%	7	5	18	60	0.156
IPI00664442.3	sodium bicarbonate cotransporter 3	52.70%	4	1	63	183	-0.142
IPI00624932.4	sodium channel protein type 5 subunit alpha	50.64%	5	1	107	315	
IPI00671181.3	sodium channel protein type 9 subunit alpha	51.14%	6	3	134	380	
IPI00395089.3	Sodium channel subunit beta-4	82.46%	1	2	22	116	
IPI00761641.1	sodium channel, voltage-gated, type II, alpha 1	53.64%	4	6	123	342	
IPI00311682.5	Sodium/potassium-transporting ATPase subunit alpha-1	68.04%	2	29	98	1828	0.210
IPI00420569.2	Sodium/potassium-transporting ATPase subunit alpha-2	71.96%	3	37	119	1475	0.244
IPI00122048.2	Sodium/potassium-transporting ATPase subunit alpha-3	78.18%	2	40	101	1667	0.244
IPI00378485.3	Sodium/potassium-transporting ATPase subunit alpha-4	67.73%	2	1	84	648	0.157
IPI00121550.3	Sodium/potassium-transporting ATPase subunit beta-1	71.71%	1	21	33	358	0.854
IPI00123704.1	Sodium/potassium-transporting ATPase subunit beta-2	62.41%	2	9	24	175	
IPI00124221.1	Sodium/potassium-transporting ATPase subunit beta-3	78.78%	2	8	24	185	-0.213
IPI00453817.3	Sodium-coupled neutral amino acid transporter 2	44.44%	2	6	19	237	-0.171
IPI00322156.1	Sodium-coupled neutral amino acid transporter 3	46.73%	1	4	16	74	
IPI00135324.2	solute carrier family 12 member 2	56.22%	2	27	73	370	-0.069
IPI00134191.3	Solute carrier family 2, facilitated glucose transporter member 3	36.11%	2	9	23	89	
IPI00225530.1	solute carrier family 24 (sodium/potassium/calcium exchanger), member 2	45.20%	7	3	27	99	
IPI00276926.3	solute carrier family 25, member 1	75.24%	1	3	40	105	
IPI00117662.3	solute carrier family 4 (anion exchanger), member 1, adaptor protein	75.66%	4	5	64	147	
IPI00973628.1	Solute carrier family 4 sodium bicarbonate cotransporter member 7 variant	53.75%	2	1	67	209	-0.163
IPI00753038.1	Sorbitol dehydrogenase	65.55%	1	3	29	115	0.178
IPI00221608.3	Sorting and assembly machinery component 50 homolog	76.55%	1	13	43	196	0.130
IPI00468902.2	sorting nexin-12 isoform 2	87.06%	4	6	23	258	-0.009
IPI00109212.3	Sorting nexin-2	77.65%	1	13	56	358	-0.088
IPI00420136.2	sorting nexin-3	85.19%	4	8	18	303	-0.068
IPI00130225.1	Sorting nexin-4	56.67%	1	3	33	81	
IPI00112053.1	Sorting nexin-5	77.97%	1	8	40	209	-0.042
IPI00111827.5	Sorting nexin-6	74.88%	2	8	50	192	-0.031
IPI00313275.7	Sorting nexin-9	70.59%	2	5	46	216	-0.037
IPI00308484.4	SPARC-like protein 1	44.00%	1	8	26	75	
IPI00757353.2	spectrin alpha chain, brain isoform 2	94.71%	4	0	386	5026	0.038
IPI00968403.1	spectrin alpha chain, brain isoform 3	94.67%	5	1	379	5011	0.034
IPI00323230.6	Spectrin alpha chain, erythrocyte	69.90%	3	2	220	915	
IPI00856469.1	spectrin beta 4 isoform sigma1	75.05%	6	3	246	990	0.020
IPI00131376.5	spectrin beta chain, erythrocyte	76.47%	5	17	229	812	0.051
IPI00421240.5	Sperm-associated antigen 17	64.46%	2	1	164	587	
IPI00273232.6	Sperm-associated antigen 7	82.38%	2	4	22	87	0.080
IPI00136912.1	Spermidine synthase	86.09%	1	11	25	485	-0.344
IPI00124127.1	Spermine synthase	78.14%	2	3	27	83	
IPI00331163.9	S-phase kinase-associated protein 1	96.93%	4	12	23	332	-0.095
IPI00122396.1	Sphingomyelin phosphodiesterase 3	62.29%	1	4	44	143	0.481
IPI00320480.3	Sphingosine-1-phosphate lyase 1	73.59%	4	15	58	345	0.016
IPI00409462.2	Spliceosome RNA helicase Ddx39b	84.58%	2	16	51	565	0.004
IPI00408796.3	Splicing factor 3A subunit 1	69.28%	1	16	68	738	0.100
IPI00380281.4	splicing factor 3A subunit 2	42.06%	3	7	30	203	-0.102
IPI00623284.4	Splicing factor 3B subunit 1	77.45%	3	40	127	960	0.013
IPI00154082.2	Splicing factor 3B subunit 4	38.68%	1	1	17	144	-0.170
IPI00311948.3	Splicing factor 3B subunit 5	44.19%	1	3	5	19	
IPI00349401.5	splicing factor 3b, subunit 2	63.21%	1	25	78	595	0.011
IPI00170394.1	Splicing factor 45	83.21%	2	10	45	255	0.006
IPI00318548.3	Splicing factor U2AF 35 kDa subunit	71.13%	6	7	21	213	-0.111
IPI00990842.2	splicing factor U2AF 65 kDa subunit isoform 2	75.37%	2	13	36	738	-0.042
IPI00986609.1	splicing factor U2AF 65 kDa subunit-like	86.16%	2	2	78	522	0.052
IPI00848672.1	splicing factor, arginine/serine-rich 11 isoform 2	66.14%	12	8	43	168	-0.004
IPI00129430.1	Splicing factor, proline- and glutamine-rich	61.95%	1	29	68	853	0.019
IPI00889824.1	SRC kinase signaling inhibitor 1	78.88%	3	1	131	508	
IPI00118143.2	Src substrate cortactin	78.94%	2	21	72	649	0.249
IPI00989397.1	src substrate cortactin	71.82%	2	7	41	798	0.124
IPI00123129.1	Staphylococcal nuclease domain-containing protein 1	92.31%	3	46	107	1469	0.004
IPI00551236.3	Stathmin	87.92%	6	3	29	413	0.058
IPI00649157.1	Stathmin	88.44%	6	1	29	377	0.043
IPI00309223.4	Stathmin-3	71.67%	3	2	23	61	
IPI00622783.2	STE20/SPS1-related proline-alanine-rich protein kinase	69.96%	2	3	44	125	
IPI00128692.1	Sterol-4-alpha-carboxylate 3-dehydrogenase, decarboxylating	67.13%	1	8	25	97	0.051
IPI00471361.3	STIP1 homology and U box-containing protein 1	72.04%	1	8	32	101	
IPI00115117.1	Stomatin-like protein 2	93.48%	4	14	47	508	-0.010
IPI00133903.1	Stress-70 protein, mitochondrial	88.22%	2	53	107	2766	-0.146
IPI00121514.3	Stress-induced-phosphoprotein 1	92.08%	1	50	94	1433	0.134
IPI00352986.3	Striatin	72.18%	3	5	57	154	
IPI00955742.1	striatin-3 isoform 2	65.73%	4	9	48	190	-0.059
IPI00123852.7	stromal cell-derived factor 2	66.21%	3	3	20	124	0.250
IPI00227657.6	Stromal cell-derived factor 2-like protein 1	81.45%	1	3	21	65	0.126
IPI00108041.2	Stromal interaction molecule 1	58.39%	1	7	49	172	-0.012
IPI00127492.1	Stromal membrane-associated protein 1	35.00%	2	5	22	141	
IPI00137433.3	Structural maintenance of chromosomes flexible hinge domain-containing p	79.27%	2	24	203	896	-0.009
IPI00123870.2	Structural maintenance of chromosomes protein 1A	84.35%	2	33	188	987	-0.003
IPI00987134.1	Structural maintenance of chromosomes protein 2	85.47%	4	40	164	814	-0.001
IPI00132122.3	Structural maintenance of chromosomes protein 3	89.81%	2	37	192	1085	-0.017
IPI00229397.1	Structural maintenance of chromosomes protein 4	87.01%	6	31	179	765	-0.128
IPI00230351.1	Succinate dehydrogenase [ubiquinone] flavoprotein subunit, mitochondria	85.54%	1	25	71	771	0.065
IPI00338536.1	Succinate dehydrogenase [ubiquinone] iron-sulfur subunit, mitochondria	84.04%	1	15	55	407	0.327
IPI00319111.3	Succinate dehydrogenase cytochrome b560 subunit, mitochondria	83.43%	2	3	11	33	
IPI00273164.1	Succinate-semialdehyde dehydrogenase, mitochondria	89.67%	1	24	47	278	
IPI00261627.1	Succinyl-CoA ligase [ADP-forming] subunit beta, mitochondrial	98.49%	1	24	68	596	0.034
IPI00132653.1	Succinyl-CoA:3-ketoacid-coenzyme A transferase 1, mitochondrial	87.69%	2	18	58	608	-0.076
IPI00928320.1	sulfated glycoprotein 1 isoform F preproprotein	79.67%	6	14	53	420	0.033
IPI00153144.3	Sulfite oxidase, mitochondrial	68.32%	1	6	37	110	
IPI00130173.1	SUMO-activating enzyme subunit 2	81.19%	2	23	59	502	-0.141
IPI00119227.1	SUMO-conjugating enzyme UBC9	72.78%	5	9	17	120	0.050
IPI00454140.5	superkiller viralicidic activity 2-like	58.12%	2	3	69	157	
IPI00113134.1	Superkiller viralicidic activity 2-like 2	73.37%	3	21	94	427	0.013
IPI00130589.8	Superoxide dismutase [Cu-Zn]	70.13%	2	12	20	415	-0.074
IPI00109109.1	Superoxide dismutase [Mn], mitochondrial	99.10%	1	8	28	260	-0.150
IPI00408957.3	Suppressor of G2 allele of SKP1 homolog	90.18%	1	11	45	258	0.085
IPI00130510.2	Suppressor of SWI4 1 homolog	84.47%	1	10	52	336	-0.317
IPI00923639.17	Suprabasin	73.00%	3	4	32	106	
IPI00137612.1	Surfeit locus protein 6	77.75%	2	6	54	170	
IPI00454015.2	SURP and G-patch domain-containing protein 1	78.54%	2	4	62	182	-0.131
IPI00221714.1	Survival of motor neuron-related-splicing factor 30	68.07%	1	3	21	133	-0.183
IPI00651846.1	SWI/SNF complex subunit SMARCC2 isoform 2	68.94%	3	13	98	298	0.004
IPI00119892.1	SWI/SNF-related matrix-associated actin-dependent regulator chromatin su	95.62%	2	10	44	183	-0.029
IPI00396739.1	SWI/SNF-related matrix-associated actin-dependent regulator of chromatin	87.25%	7	47	144	815	0.100
IPI00467181.2	SWI/SNF-related matrix-associated actin-dependent regulator of chromatin	71.01%	1	3	46	139	
IPI00130948.3	Switch-associated protein 70	67.69%	1	5	58	156	
IPI00317599.3	Synapse-associated protein 1	82.74%	1	9	30	260	-0.054
IPI00465810.3	Synaptic vesicle glycoprotein 2A	60.51%	1	18	42	170	
IPI00221456.1	Synaptic vesicle glycoprotein 2B	50.81%	2	15	37	163	
IPI00755859.1	Synaptic vesicle glycoprotein 2C	33.43%	2	4	27	71	
IPI00126072.2	Synaptic vesicle membrane protein VAT-1 homolog	74.63%	1	18	30	392	0.052
IPI00222759.3	Synaptic vesicle membrane protein VAT-1 homolog-like	87.05%	2	18	35	323	0.092
IPI00453771.2	Synaptobrevin homolog YKT6	92.42%	1	10	28	162	-0.209
IPI00331579.1	Synaptogyrin-3	43.67%	1	2	13	49	
IPI00989529.1	synaptojanin-1 isoform b	79.53%	6	37	119	558	
IPI00123505.2	Synaptophysin	70.38%	4	13	24	155	
IPI00113798.2	synaptosomal-associated protein 23 isoform a	92.76%	5	7	34	275	-0.089
IPI00129618.1	Synaptotagmin-1	84.56%	2	15	44	406	
IPI00129622.3	Synaptotagmin-2	58.53%	1	10	33	213	
IPI00400053.1	synaptotagmin-7 beta isoform	72.26%	3	1	39	155	
IPI00229256.5	synaptotagmin-7 gamma isoform	70.37%	3	1	49	227	
IPI00136382.1	Syndecan-4	38.38%	1	2	7	29	0.040
IPI00111416.1	Syntaxin-12	91.97%	1	11	36	421	-0.111
IPI00131618.1	Syntaxin-1A	86.81%	4	1	39	157	
IPI00113149.1	Syntaxin-1B	95.49%	4	19	45	356	
IPI00109335.1	Syntaxin-4	88.59%	1	4	33	164	
IPI00118217.3	Syntaxin-7	71.26%	1	8	31	256	0.361
IPI00133317.1	Syntaxin-binding protein 2	60.20%	2	2	45	105	
IPI00656317.2	syntaxin-binding protein 5	54.21%	5	5	52	111	
IPI00465786.4	Talin-1	91.50%	5	113	306	3909	0.041
IPI00229647.6	talin-2	92.33%	3	8	300	2003	0.066
IPI00121842.2	TAP2	60.88%	1	3	46	152	
IPI00122523.2	Tapasin-related protein	93.79%	1	2	36	135	
IPI00121758.1	TAR DNA-binding protein 43	75.60%	7	13	39	803	0.132
IPI00420481.5	Targeting protein for Xklp2	75.70%	1	9	91	292	0.310
IPI00665510.1	tax1-binding protein 3-like isoform 2	93.55%	2	2	14	84	0.020
IPI00469012.3	TBC1 domain family member 10B	45.61%	2	3	37	82	
IPI00110247.7	TBC1 domain family member 15	52.31%	1	6	39	132	-0.231
IPI00676513.5	TBC1 domain family member 8B	68.22%	1	5	83	255	-0.116
IPI00320217.9	T-complex protein 1 subunit beta	97.01%	2	40	81	1164	-0.085
IPI00116277.3	T-complex protein 1 subunit delta	97.77%	6	35	90	1255	-0.152
IPI00116279.3	T-complex protein 1 subunit epsilon	94.27%	2	35	73	1102	-0.180
IPI00331174.5	T-complex protein 1 subunit eta	94.49%	1	35	69	1027	-0.059
IPI00116283.1	T-complex protein 1 subunit gamma	93.58%	4	37	90	1339	-0.114
IPI00469268.5	T-complex protein 1 subunit theta	94.53%	1	40	71	1146	-0.116
IPI00116281.3	T-complex protein 1 subunit zeta	89.45%	1	23	69	973	-0.082
IPI00321816.6	T-complex protein 1 subunit zeta-2	76.27%	2	1	47	310	-0.125
IPI00850595.1	TCTEX5 isoform 3	68.25%	5	2	11	42	
IPI00378438.6	tensin 1	61.44%	3	9	119	447	
IPI00344307.2	testin	79.00%	5	15	49	328	0.079
IPI00349443.5	testis expressed gene 264	62.14%	2	2	19	40	
IPI00458057.3	Testis-expressed sequence 10 protein	81.68%	2	7	95	276	
IPI00153756.1	tetraspanin 8	52.34%	1	3	10	83	0.563
IPI00757517.1	tetratricopeptide repeat domain 7B	58.72%	1	8	66	228	
IPI00133612.1	Tetratricopeptide repeat protein 35	70.03%	1	7	32	91	-0.155
IPI00322150.8	Thimet oligopeptidase	73.36%	1	21	78	473	-0.034
IPI00226993.5	Thioredoxin	88.57%	1	7	9	90	
IPI00133110.1	Thioredoxin domain-containing protein 12	89.41%	1	5	14	92	0.397
IPI00471077.3	Thioredoxin domain-containing protein 17	60.16%	1	3	9	37	0.041
IPI00163011.2	Thioredoxin domain-containing protein 5	92.09%	3	17	36	268	0.111
IPI00132151.1	Thioredoxin domain-containing protein 9	79.20%	1	6	23	100	-0.122
IPI00116192.1	Thioredoxin-dependent peroxide reductase, mitochondria	96.89%	1	10	31	308	0.021
IPI00266281.4	Thioredoxin-like protein 1	93.43%	2	13	28	590	-0.162
IPI00121341.1	Thioredoxin-related transmembrane protein 1	54.32%	2	11	27	133	-0.050
IPI00459309.2	Thioredoxin-related transmembrane protein 2	56.61%	3	5	19	99	-0.034
IPI00453829.3	Thioredoxin-related transmembrane protein 4	61.49%	2	5	19	94	
IPI00223216.5	Thiosulfate sulfurtransferase	51.18%	2	6	23	103	0.081
IPI00153778.5	THO complex subunit 1	71.08%	1	8	52	113	-0.183
IPI00664886.2	THO complex subunit 2	71.46%	1	4	154	455	-0.288
IPI00222687.2	THO complex subunit 5 homolog	71.60%	3	9	59	263	0.051
IPI00468688.4	Threonyl-tRNA synthetase, cytoplasmic	76.59%	1	29	86	726	-0.017
IPI00109727.1	Thy-1 membrane glycoprotein	98.15%	1	6	19	127	
IPI00648094.3	Thymidine kinase, cytosolic	78.97%	1	5	26	106	-0.548
IPI00130734.1	Thymidylate synthase	79.15%	2	6	28	97	0.152
IPI00130226.1	Thymocyte nuclear protein 1	79.20%	2	3	27	65	
IPI00117583.3	Thymosin beta-10	47.73%	1	2	7	160	0.330
IPI00380829.4	thyroid hormone receptor interactor 11	83.70%	1	7	247	1067	-0.076
IPI00623570.3	thyroid hormone receptor interactor 12	82.86%	3	19	205	1047	0.242
IPI00556768.1	Thyroid hormone receptor-associated protein 3	76.55%	5	21	128	507	0.123
IPI00135971.2	Tight junction protein ZO-1	77.94%	1	1	143	973	0.135

IPI00944062.1	tight junction protein ZO-1 isoform 2	82.02%	1	1	146	1004	0.128	0.027	0.073	-0.019	0.050	60	0.465	0.038	0.335	0.006	0.233	75	-0.099	0.037	-0.088	0.017	-0.116	26													1685	188.7	6.67
IPI00323349.2	Tight junction protein ZO-2	83.38%	2	31	138	772	0.350	-0.010	0.403	-0.035	0.288	64	0.240	-0.006	0.013	-0.034	0.062	24	0.153	-0.074	-0.045	-0.031	0.128	9													1167	131.2	6.79
IPI00221741.1	TIP41-like protein	60.52%	1	5	15	55	-0.150	-0.204	-0.157	-0.017	-0.082	3	-0.351	0.048	-0.396	-0.120	-0.029	4							-0.079	-0.198	0.031	0.006	0.149	9							271	31.2	5.63
IPI00136618.1	Toll-interacting protein	73.72%	4	10	24	94							0.717	-0.510	0.815	0.280	0.855	4													0.081	0.114	0.214	0.140	0.190	15	274	30.3	5.17
IPI00975188.1	torsin-1A-interacting protein 1 isoform 1	70.14%	5	14	53	367	0.188	0.195	-0.108	0.057	0.006	6	0.126	-0.012	0.136	0.041	0.021	29	0.102	-0.017	0.047	-0.116	0.084	11													576	64.8	7.14
IPI00421157.4	Torsin-1A-interacting protein 2	57.97%	1	4	29	77	-0.149	-0.095	-0.269	-0.097	0.026	3	0.190	0.157	0.031	0.036	0.059	7																			502	54.5	4.86
IPI00460669.3	Torsin-3A	54.81%	1	2	25	76							0.358	0.111	1.138	-0.150	0.437	4																			385	43.8	6.28
IPI00121251.8	TOX high mobility group box family member 4	38.61%	3	2	28	172	0.044	0.023	-0.201	-0.181	-0.246	5																									619	65.9	5.01
IPI00277184.1	TraB domain-containing protein	67.02%	3	2	22	69																			-0.063	-0.233	0.199	-0.066	0.152	3							376	42.2	8.41
IPI00115056.1	Trafficking protein particle complex subunit 3	68.33%	1	3	22	126	0.044	0.139	0.205	-0.108	0.115	7	-0.274	-0.092	-0.383	0.000	-0.120	4	-0.342	-0.170	-0.342	0.130	-0.119	4							-0.108	0.029	-0.020	0.111	0.316	4	180	20.3	4.96
IPI00121276.4	Trans-2-enoyl-CoA reductase, mitochondrial	89.81%	2	8	32	188	-0.176	-0.016	-0.308	-0.004	-0.404	5	-0.513	-0.099	-0.144	-0.095	-0.071	4													0.017	-0.134	-0.085	-0.311	-0.125	6	373	40.3	9.07
IPI00124692.1	Transaldolase	70.92%	1	21	46	513	0.149	-0.082	0.156	-0.056	0.178	43	-0.292	-0.037	-0.112	-0.091	-0.173	29	-0.184	-0.187	-0.255	0.112	-0.077	18	0.132	-0.072	-0.013	0.041	0.136	26	-0.048	0.059	0.045	0.038	0.006	45	337	37.4	7.03
IPI00757153.2	Transcription elongation factor A protein-like 3	61.00%	3	1	19	94																									-0.012	0.127	0.204	0.193	0.179	32	200	22.5	5.44
IPI00228674.3	Transcription elongation factor A protein-like 5	69.50%	1	3	20	116																									-0.012	0.152	0.170	0.197	0.220	40	200	22.0	6.20
IPI00323130.3	Transcription elongation factor B polypeptide 1	92.86%	3	4	10	217	-0.047	-0.047	-0.042	0.016	-0.048	15	-0.370	-0.127	-0.318	-0.238	-0.219	11	-0.123	-0.050	-0.109	0.051	-0.050	5	0.043	0.007	0.207	0.031	0.185	22	-0.183	-0.130	-0.228	-0.077	-0.050	16	112	12.5	4.78
IPI00131224.1	Transcription elongation factor B polypeptide 2	98.31%	1	6	13	295	-0.035	-0.088	0.069	0.131	0.122	23	-0.119	-0.042	-0.108	-0.080	-0.152	26	-0.191	-0.122	-0.228	0.029	-0.039	9	0.360	0.156	0.293	0.135	0.184	18	-0.039	-0.085	-0.114	-0.053	-0.076	13	118	13.2	5.01
IPI00317167.3	Transcription elongation factor B polypeptide 3	60.93%	1	9	77	195	0.072	-0.006	-0.072	-0.157	-0.228	3	-0.262	-0.038	-0.357	-0.134	-0.425	10																			773	87.1	9.61
IPI00454050.1	Transcription elongation factor SPT6	67.38%	1	16	137	475	-0.010	0.064	-0.020	0.024	-0.016	15	-0.100	0.029	0.263	0.127	-0.088	11	-0.022	-0.136	-0.149	-0.597	-0.007	3	-0.114	0.036	0.001	0.037	0.091	11							1726	199.0	4.93
IPI00132539.3	Transcription factor BTF3 homolog 4	97.47%	1	3	21	367	0.187	-0.029	0.391	-0.001	0.323	33	0.095	-0.106	-0.001	0.013	-0.286	10	-0.216	-0.096	-0.300	-0.001	-0.091	14	0.052	0.054	-0.051	0.044	0.030	26	-0.181	0.038	0.180	0.058	0.335	3	158	17.3	6.35
IPI00137166.1	Transcription factor jun-B	70.93%	3	5	33	97	0.175	0.052	-0.071	-0.037	0.120	6	-0.336	-0.178	-0.478	-0.211	-0.012	4	0.323	0.067	0.249	0.048	0.115	4													344	35.7	9.22
IPI00153874.1	Transcription initiation factor IIB	90.51%	1	7	47	194	-0.050	-0.017	0.076	-0.076	0.038	4	-0.370	-0.029	-0.213	0.004	-0.221	8	-0.250	0.009	-0.139	0.306	0.075	5													316	34.8	8.35
IPI00118447.1	Transcriptional activator protein Pur-alpha	94.39%	1	16	37	472	0.176	0.079	0.103	0.130	0.064	22	0.353	0.099	0.361	0.269	0.038	9	-0.276	0.029	-0.128	-0.180	-0.565	4	-0.219	0.041	-0.119	0.026	-0.163	33	0.038	0.079	0.136	0.085	0.019	43	321	34.9	6.44
IPI00128867.3	Transcriptional activator protein Pur-beta	89.81%	2	10	37	362	0.162	-0.034	0.119	-0.044	0.138	30	0.075	-0.028	0.198	-0.490	0.212	6	-0.427	-0.054	-0.193	-0.178	-0.265	23	-0.060	0.110	-0.060	0.014	0.162	19	0.205	0.010	0.026	-0.003	0.085	31	324	33.9	5.43
IPI00322707.6	Transcriptional regulator ATRX	69.47%	9	17	235	922	0.190	0.114	0.088	0.057	0.119	14	0.172	-0.006	0.174	-0.128	0.181	12	-0.211	-0.007	-0.022	-0.051	0.122	8	0.118	-0.029	-0.150	0.016	-0.223	8							2476	278.4	6.68
IPI00320741.1	Transcriptional repressor CTCF	54.89%	1	8	40	188	0.229	0.033	0.321	0.194	0.170	3	-0.022	0.068	0.330	0.233	-0.144	19																			736	83.7	6.90
IPI00229784.2	Transcriptional repressor p66 alpha	85.69%	2	10	71	414	-0.089	0.038	0.107	0.151	0.050	9	0.162	0.017	0.299	-0.333	0.072	3	-0.034	-0.114	-0.390	-0.309	0.084	3	0.044	0.083	0.069	0.083	0.193	6							629	67.3	9.89
IPI00127026.2	Transducin beta-like protein 2	75.11%	3	5	37	110							0.229	-0.155	0.246	0.215	0.200	9																			442	49.6	9.04
IPI00124057.2	Transducin beta-like protein 3	87.02%	2	9	73	303	-0.057	-0.039	-0.186	-0.157	-0.119	8													-0.183	-0.035	-0.358	-0.221	-0.228	6							801	88.2	6.81
IPI00124700.1	Transferrin receptor protein 1	74.44%	2	30	74	631	-0.204	0.045	-0.183	-0.009	-0.260	40	0.366	0.083	0.027	-0.068	0.110	20	-0.010	0.063	-0.041	0.017	-0.127	61	0.084	-0.128	0.016	-0.094	-0.029	33							763	85.7	6.57
IPI00315100.1	Transforming protein RhoA	88.60%	3	6	24	368	-0.019	-0.103	0.026	-0.038	0.059	39	0.190	0.036	0.135	-0.048	0.195	25	0.072	0.011	-0.059	0.020	-0.035	27	-0.160	0.072	-0.130	0.048	-0.060	12	0.092	0.045	-0.060	-0.039	0.007	32	193	21.8	6.10
IPI00226515.5	Transgelin	96.52%	1	14	28	187							0.766	-0.054	0.427	-0.313	0.611	33	0.070	-0.084	0.152	0.184	0.246	7							0.119	0.045	-0.063	0.054	0.104	9	201	22.6	8.81
IPI00125778.4	Transgelin-2	96.98%	1	13	35	767	0.222	-0.071	0.324	-0.013	0.320	54	0.136	-0.025	0.166	-0.130	0.218	48	-0.216	-0.156	-0.186	0.106	-0.023	82	-0.456	0.058	-0.678	0.016	-0.379	51	0.029	-0.157	-0.038	-0.095	0.084	16	199	22.4	8.24
IPI00128986.1	Transgelin-3	95.48%	2	9	30	361	0.207	0.000	0.276	0.029	0.175	7	0.094	0.085	-0.116	-0.165	-0.004	3	-0.248	-0.190	-0.032	0.031	-0.108	9	-0.571	-0.009	-0.653	0.027	-0.318	12	0.010	-0.221	-0.131	-0.133	-0.123	40	199	22.5	7.33
IPI00622235.5	Transitional endoplasmic reticulum ATPase	88.83%	9	53	102	2288	0.165	-0.010	0.149	-0.003	0.139	168	0.026	0.007	0.040	-0.026	-0.007	158	-0.228	-0.069	-0.229	-0.020	-0.147	167	0.081	0.054	0.175	0.023	0.116	164	-0.004	-0.114	-0.139	-0.138	-0.071	122	806	89.3	5.26
IPI00137409.3	Transketolase	98.23%	1	35	77	1292	0.212	-0.065	0.352	-0.042	0.295	134	0.172	-0.013	0.285	0.087	0.151	42	-0.134	-0.145	0.165	-0.010	0.122	9	0.444	0.131	0.482	0.044	0.429	97	0.019	0.154	0.119	0.047	0.079	79	623	67.6	7.50
IPI00116761.1	Translation initiation factor eIF-2B subunit alpha	77.70%	3	4	28	95	-0.264	-0.077	-0.751	0.059	-0.476	3													0.141	0.009	0.100	0.021	0.126	4							305	33.8	8.32
IPI00116804.1	Translation initiation factor eIF-2B subunit beta	79.77%	3	5	28	134	0.076	-0.099	0.079	-0.028	0.111	10	-0.333	0.148	0.132	-0.271	0.203	6	-0.034	-0.468	-0.980	0.029	-0.032	3	-0.020	0.081	-0.014	0.087	-0.193	5							351	38.9	6.24
IPI00261239.6	Translation initiation factor eIF-2B subunit epsilon	68.34%	3	7	56	178	0.285	0.188	0.193	0.156	0.231	9	-0.146	-0.026	-0.028	-0.130	-0.023	10	-0.236	-0.132	-0.142	-0.175	-0.109	3	-0.144	0.180	-0.306	0.091	-0.285	5							717	80.0	5.07
IPI00129685.3	Translationally-controlled tumor protein	94.19%	2	9	14	478	-0.167	-0.120	0.120	0.038	-0.022	45	0.011	-0.028	-0.050	-0.077	0.119	24	-0.128	-0.109	-0.108	0.167	0.065	30	0.722	0.222	0.816	0.202	0.919	43	0.023	0.019	0.070	0.010	0.054	12	172	19.4	4.86
IPI00124684.1	Translin	66.23%	1	6	24	114	-0.338	-0.065	-0.482	-0.100	-0.243	3	-0.010	0.169	0.059	0.098	-0.014	4	-0.624	-0.226	-0.462	0.088	-0.379	6	-0.103	0.058	-0.009	-0.050	0.066	12	0.244	0.130	0.107	0.314	0.083	5	228	26.2	6.44
IPI00125907.3	Translocating chain-associated membrane protein 1	54.28%	1	4	17	72							0.132	-0.006	-0.097	0.174	0.036	7	0.305	0.088	0.167	0.124	0.116	3													374	43.0	9.69
IPI00134398.6	Translocation protein SEC62	37.94%	1	8	23	113	0.030	0.107	-0.238	0.017	-0.139	4	-0.004	0.123	0.026	0.052	0.026	20	0.231	0.050	0.213	-0.023	0.058	3													398	45.6	7.31
IPI00330857.6	Translocation protein SEC63 homolog	75.26%	2	18	79	387	-0.279	0.100	-0.257	0.006	-0.313	12	0.252	0.151	0.199	0.271	0.020	13	-0.003	0.095	0.026	-0.082	-0.155	25	-0.099	0.050	-0.209	0.007	-0.201	29							760	87.8	5.38
IPI00755329.1	Translocon-associated protein subunit alpha	70.98%	2	5	12	325	0.517	0.054	0.597	0.141	0.450	13	0.262	0.023	0.301	-0.039	0.350	18	0.619	0.098	0.356	-0.029	0.261	10	0.112	-0.042	-0.013	0.068	-0.038	8							286	32.0	4.45
IPI00122346.3	Translocon-associated protein subunit delta	47.09%	2	4	11	100	-0.105	0.077	-0.086	0.098	-0.163	12	-0.262	0.106	0.169	0.045	0.033	5	0.161	0.004	0.224	0.107	0.148	8													172	18.9	5.78
IPI00113528.1	Transmembrane 9 superfamily member 3	62.69%	3	7	33	131	0.127	0.163	0.034	0.075	-0.048	6	-0.020	0.092	0.114	0.079	0.065	8							0.057	0.096	-0.051	0.038	0.107	5							587	67.5	7.21
IPI00626537.2	Transmembrane 9 superfamily member 4	60.50%	2	7	42	128							-0.508	0.234	-0.271	0.240	-0.708	5	0.126	0.061	0.257	0.092	-0.037	6													643	74.6	7.23
IPI00121974.1	Transmembrane and coiled-coil domain-containing protein 1	91.49%	2	5	27	106	-0.353	0.068	0.066	-0.022	-0.248	4	-0.464	-0.047	-0.163	0.447	-0.248	6	0.318	-0.065	0.270	0.076	-0.029	5	0.037	-0.054	-0.041	-0.022	-0.097	3							188	21.2	9.74
IPI00127983.1	Transmembrane emp24 domain-containing protein 2	78.11%	3	9	20	184	0.123	0.120	0.059	0.064	0.006	8	0.321	0.027	0.147	0.069	0.323	15	0.134	0.050	0.132	-0.099	0.131	20	0.055	0.020	-0.095	0.001	-0.009	8	-0.026	0.023	0.160	-0.114	-0.128	3	201	22.7	5.17
IPI00153468.1	Transmembrane emp24 domain-containing protein 4	93.39%	3	5	31	208	0.009	0.108	-0.048	0.010	-0.039	10							0.476	-0.028	0.285	-0.313	0.301	6	-0.234	-0.163	-0.171	-0.091	-0.176	6	-0.347	-0.131	-0.047	-0.125	-0.003	5	227	26.0	8.18
IPI00466640.2	Transmembrane emp24 domain-containing protein 5	53.28%	2	3	17	69	0.143	0.054	-0.019	-0.060	0.070	4	0.077	-0.092	-0.074	-0.144	0.092	3																			229	26.2	4.93
IPI00134378.9	transmembrane emp24 protein transport domain containing 7	87.05%	2	5	21	77	0.047	0.077	0.034	0.033	-0.009	9	0.421	0.144	0.402	0.256	0.387	4	0.310	0.087	0.234	0.004	0.099	7													224	25.2	6.89
IPI00330680.5	Transmembrane protein 106B	69.82%	2	3	14	28							-0.960	0.048	-0.444	-0.111	-0.347	3																			275	31.2	6.68
IPI00153101.3	Transmembrane protein 109	55.56%	5	3	21	81													0.393	0.153	0.218	0.016	0.259	6	0.003	-0.017	-0.146	0.030	-0.267	5							243	26.3	9.89
IPI00222447.1	Transmembrane protein 11, mitochondrial	46.84%	2	2	13	35	-0.265	-0.088	-0.171	0.054	-0.099	3																			0.077	-0.141	-0.044	-0.228	0.038	5	190	21.3	7.36
IPI00116077.3	Transmembrane protein 111	85.44%	1	7	27	145	-0.041	-0.076	-0.309	-0.127	-0.082	8	0.161	0.123	0.016	0.162	0.281	5	0.085	0.023	0.076	-0.102	-0.189	12													261	30.0	6.81
IPI00395051.2	Transmembrane protein 119	35.00%	1	4	7	45							-0.582	0.163	-0.732	0.088	-0.791	12																			280	29.4	4.51
IPI00224209.1	Transmembrane protein 120A	81.92%	2	2	35	119	-0.141	-0.020	-0.209	-0.103	-0.218	3	-0.057	0.308	0.156	-0.068	-0.047	5																			343	40.7	8.88
IPI00118333.2	Transmembrane protein 131	52.32%	1	3	112	430																															1877	204.5	8.54
IPI00131606.2	Transmembrane protein 165	73.37%	2	3	23	151	-0.229	0.120	-0.114	-0.167	0.010	8	-0.490	0.118	-0.144	0.122	-0.300	10							0.179	0.014	0.231	-0.097	0.225	7							323	34.8	7.46
IPI00387361.1	Transmembrane protein 176B	71.86%	1	3	17	75	-0.138	-0.001	-0.044	-0.012	-0.209	5																									263	28.3	7.84
IPI00133234.1	Transmembrane protein 33	72.06%	3	5	22	259	0.016	0.041	-0.108	-0.065	-0.071	9	-0.133	0.124	-0.085	-0.009	-0.025	5	0.460	0.045	0.140	-0.057	0.066	5	0.055	-0.066	0.007	0.006	0.011	11	0.006	0.087	0.034	-0.035	-0.088	7	247	28.0	9.66
IPI00120083.1	Transmembrane protein 43	67.50%	1	10	31	102	-0.186	-0.009	-0.221	-0.069	-0.226	5							0.345	0.027	0.309	-0.077	0.215	11													400	44.8	7.36
IPI00626928.1	Transmembrane protein 65	52.14%	1	5	14	28																									0.144	0.017	-0.009	0.054	0.050	4	234	24.9	7.78
IPI00625262.4	transmembrane protein 87A isoform 1	62.50%	4	4	35	98							0.943	-0.214	1.123	0.217	0.183	3																			560	63.8	7.12
IPI00133012.1	Transmembrane protein 9	62.84%	3	2	14	28							-0.221	-0.050	-0.271	-0.191	-0.208	3																			183	20.6	6.95
IPI00928105.1	transmembrane protein C20orf108 homolog	76.84%	2	2	14	38	0.452	-0.017	0.288	0.094	0.437	4																									190	20.3	10.51
IPI00136293.1	Transmembrane protein C9orf46 homolog	78.23%	4	3	17	51							0.014	0.200	-0.150	-0.370	0.331	3																			147	17.2	9.50
**IPI00116877.5**	**transportin-2**	**74.36%**	**1**	**10**	**61**	**299**	0.103	-0.013	0.180	-0.045	0.189	5	-0.278	-0.051	-0.014	-0.050	0.180	**28**							0.145	0.057	0.085	0.034	0.001	**11**							**897**	**101.4**	**5.01**
IPI00127560.1	Transthyretin	78.91%	1	6	11	33																									0.258	0.157	0.222	0.431	0.580	12	147	15.8	6.16
IPI00988759.1	treacle protein isoform 1	84.81%	5	32	156	1015	-0.131	-0.001	-0.196	0.004	-0.283	15	0.367	0.091	0.428	0.094	0.225	58	0.045	-0.041	-0.112	-0.019	0.040	28	-0.032	-0.119	-0.032	-0.029	-0.072	17							1356	138.5	9.26
IPI00223092.5	Trifunctional enzyme subunit alpha, mitochondrial	91.22%	1	35	97	840	0.115	0.054	0.036	0.019	0.064	59	0.179	0.033	0.245	0.136	0.061	52	0.255	0.066	0.225	-0.050	0.094	39	0.187	0.040	0.169	-0.063	0.197	32	-0.099	0.118	0.138	0.079	0.108	67	763	82.6	9.14
IPI00115607.3	Trifunctional enzyme subunit beta, mitochondrial	92.84%	7	17	62	467	0.154	0.111	0.158	0.091	0.149	24	0.273	-0.035	0.315	0.122	0.172	21	0.280	0.107	0.189	-0.054	0.111	30	0.185	-0.060	0.217	-0.041	0.280	17	0.107	0.208	0.223	0.140	0.076	33	475	51.4	9.38
IPI00467833.6	triosephosphate isomerase	94.31%	2	19	31	1040	-0.001	-0.125	0.069	-0.041	0.099	77	-0.694	-0.004	-0.513	-0.119	-0.496	68	0.135	-0.173	0.127	0.145	0.274	70	0.430	0.132	0.541	0.057	0.469	65	0.123	0.004	0.011	-0.068	-0.009	126	299	32.2	5.74
IPI00895985.1	Tripartite motif protein 25	84.86%	4	1	61	171	0.023	-0.054	0.136	-0.074	0.110	6	0.115	-0.088	0.163	-0.007	-0.014	7																			634	71.8	8.29
IPI00130661.1	Tripeptidyl-peptidase 1	68.68%	1	5	29	90	0.188	-0.088	-0.028	0.066	0.152	3													-0.209	0.152	-0.769	-0.082	-0.063	3	0.007	0.118	-0.025	0.210	0.301	9	562	61.3	6.57
IPI00109290.3	tRNA (guanine-N(7)-)-methyltransferase subunit WDR4	70.61%	5	2	34	73	0.161	0.262	0.517	0.364	0.455	3																									456	50.4	7.96
IPI00120871.1	tRNA methyltransferase 112 homolog	48.80%	3	4	8	47							-0.080	-0.248	-0.248	-0.313	-0.120	3							0.041	0.016	0.087	-0.088	0.054	7							125	14.1	5.27
IPI00116850.3	tRNA-splicing ligase RtcB homolog	83.76%	1	18	53	447	-0.082	-0.017	-0.063	0.027	-0.112	31	-0.012	0.069	0.036	0.064	0.098	12	0.003	0.000	0.016	-0.035	0.066	30	0.103	0.054	0.116	0.011	0.010	50	0.007	-0.125	0.095	0.166	0.210	9	505	55.2	7.23
IPI00309658.2	Tropomodulin-1	64.35%	1	1	37	116																									0.079	0.070	0.080	0.051	0.085	6	359	40.4	5.10
IPI00119478.1	Tropomodulin-3	77.56%	1	14	46	393	0.120	0.029	0.072	0.033	0.081	31	0.160	-0.042	0.148	-0.119	0.094	26	0.124	-0.090	0.034	-0.152	0.024	10	0.136	0.010	0.041	-0.037	-0.032	11	0.079	0.070	0.080	0.051	0.085	6	352	39.5	5.14
IPI00830223.1	tropomyosin alpha-1 chain isoform 10	93.88%	5	0	55	816	-0.042	0.081	0.040	0.048	-0.001	70	0.452	0.006	0.384	-0.003	0.210	64	-0.116	-0.073	-0.050	-0.023	0.014	74	0.162	0.045	0.240	0.000	0.116	20	0.057	-0.034	0.017	0.075	0.058	43	245	28.3	4.77
IPI00830604.1	tropomyosin alpha-1 chain isoform 2	95.07%	4	1	57	922	-0.109	0.073	0.030	0.045	-0.114	87	0.452	0.003	0.384	0.004	0.192	74	-0.186	-0.092	-0.103	-0.016	-0.042	78	0.176	0.034	0.275	0.000	0.171	18	0.040	-0.037	0.017	0.003	0.083	32	284	32.6	4.74
IPI00830884.1	tropomyosin alpha-1 chain isoform 4	95.07%	4	2	63	985	-0.176	0.051	-0.051	0.029	-0.178	96	0.464	-0.023	0.355	0.006	0.190	77	-0.186	-0.079	-0.106	-0.009	-0.042	78	0.176	0.026	0.290	0.004	0.153	19	-0.042	-0.041	0.014	0.003	0.058	27	284	32.8	4.78
IPI00831330.1	tropomyosin alpha-1 chain isoform 6	96.77%	5	0	56	935	-0.102	0.050	0.031	0.047	-0.057	84	0.498	0.004	0.404	0.016	0.210	68	-0.088	-0.069	-0.039	-0.016	0.036	76	0.162	0.045	0.240	0.000	0.116	20	0.047	-0.037	0.016	0.055	0.083	40	248	28.5	4.77
IPI00944126.1	tropomyosin alpha-1 chain isoform 8	88.71%	5	0	54	820	-0.042	0.081	0.040	0.048	-0.001	70	0.452	0.006	0.384	-0.003	0.210	64	-0.122	-0.069	-0.059	-0.023	0.000	75	0.162	0.045	0.240	0.000	0.116	20	0.057	-0.041	0.014	0.075	0.108	41	248	28.5	4.74
IPI00830427.1	tropomyosin alpha-1 chain isoform 9	92.53%	5	0	61	943	-0.114	0.080	-0.045	0.040	-0.133	89	0.530	0.003	0.370	-0.012	0.194	85	-0.231	-0.091	-0.122	-0.009	-0.056	89	0.176	0.026	0.290	0.004	0.153	19	0.040	-0.037	0.017	0.019	0.054	36	281	32.5	4.77
IPI00421223.3	Tropomyosin alpha-4 chain	98.39%	1	21	53	802	0.149	0.054	0.228	0.058	0.209	74	0.265	-0.025	0.174	-0.056	0.108	73	-0.071	-0.128	-0.032	-0.020	0.095	72	0.075	-0.079	0.126	0.001	-0.007	46	-0.032	0.006	-0.071	0.034	0.118	18	248	28.5	4.68
IPI00130828.1	Tubby-related protein 3	62.17%	1	3	35	86							-0.340	0.041	-0.001	-0.023	-0.267	4																			460	51.2	6.24
IPI00121558.5	Tubulin alpha chain-like 3	77.35%	1	1	33	141	-0.324	0.030	-0.219	0.195	-0.183	3	-0.490	-0.163	-0.519	-0.299	-0.074	5	-0.139	-0.095	-0.274	0.140	-0.023	7	0.112	0.295	0.126	-0.031	-0.022	7							446	50.0	5.58
IPI00117348.4	Tubulin alpha-1B chain	96.90%	3	2	41	1879	-0.031	-0.154	-0.022	-0.009	0.034	101	-0.229	-0.020	-0.188	-0.032	-0.086	71	-0.223	-0.182	-0.193	0.098	-0.032	117	-0.123	0.073	-0.131	-0.019	-0.066	128	0.114	0.054	0.010	0.057	0.087	261	451	50.1	5.06
IPI00403810.2	Tubulin alpha-1C chain	96.44%	1	3	42	1671	0.004	-0.127	-0.022	-0.012	0.030	94	-0.228	-0.020	-0.186	-0.065	-0.065	68	-0.224	-0.180	-0.191	0.104	-0.035	110	-0.053	0.084	-0.103	-0.003	-0.069	111	0.122	0.062	0.004	0.057	0.070	240	449	49.9	5.10
IPI00117350.1	Tubulin alpha-4A chain	92.86%	1	5	40	1611	-0.014	-0.117	0.052	-0.004	0.021	73	-0.248	-0.016	-0.186	-0.003	-0.068	58	-0.223	-0.173	-0.186	0.099	-0.025	89	-0.071	0.075	-0.095	-0.035	-0.066	100	0.104	0.052	0.017	0.036	0.085	241	448	49.9	5.06
IPI00311175.5	Tubulin alpha-8 chain	70.82%	1	4	34	1240	-0.014	-0.136	-0.042	-0.014	0.013	57	-0.265	-0.017	-0.238	-0.066	-0.095	44	-0.226	-0.173	-0.191	0.091	-0.037	77	-0.119	0.068	-0.106	-0.041	-0.079	86	0.130	0.054	-0.025	0.047	0.072	174	449	50.0	5.10
IPI00338039.1	Tubulin beta-2A chain	87.42%	3	2	48	2158	0.066	-0.076	0.202	0.027	0.175	94	-0.278	0.006	-0.091	-0.006	-0.079	63	-0.170	-0.171	-0.194	0.131	-0.016	120	-0.285	-0.007	-0.218	-0.004	-0.080	107	0.091	0.069	0.047	-0.065	0.014	239	445	49.9	4.89
IPI00109061.1	Tubulin beta-2B chain	78.65%	3	1	47	2111	0.072	-0.076	0.207	0.027	0.179	92	-0.285	-0.004	-0.091	-0.006	-0.097	59	-0.142	-0.174	-0.188	0.120	-0.004	120	-0.292	-0.007	-0.255	-0.004	-0.100	107	0.079	0.061	0.038	-0.065	0.011	227	445	49.9	4.89
IPI00169463.1	Tubulin beta-2C chain	78.65%	3	1	47	2065	0.003	-0.117	0.156	-0.016	0.141	76	-0.279	-0.048	-0.095	-0.079	-0.077	69	-0.221	-0.183	-0.219	0.115	-0.031	110	-0.094	0.009	-0.056	0.029	0.065	91	0.065	0.070	0.051	-0.022	0.010	240	445	49.8	4.89
IPI00112251.1	Tubulin beta-3 chain	86.44%	2	4	43	1516	-0.009	-0.119	0.143	-0.012	0.135	50	-0.265	0.006	-0.120	-0.048	-0.125	37	-0.253	-0.199	-0.257	0.152	-0.071	79	-0.492	-0.010	-0.630	-0.016	-0.576	117	0.041	0.092	0.058	0.007	0.040	174	450	50.4	4.93
IPI00988328.1	tubulin beta-3 chain-like	69.30%	1	1	38	982	0.212	-0.106	0.215	0.000	0.335	23	-0.279	0.037	-0.127	-0.056	-0.193	25	-0.297	-0.214	-0.317	0.143	-0.068	28	-0.633	0.041	-0.921	0.031	-0.701	42	0.003	0.059	0.040	-0.028	0.000	69	443	50.0	4.98
IPI00109073.5	Tubulin beta-4 chain	78.83%	1	6	41	1603	0.014	-0.119	0.134	-0.054	0.172	61	-0.248	-0.080	-0.007	0.009	-0.069	53	-0.252	-0.182	-0.252	0.139	-0.059	73	-0.155	-0.048	-0.162	-0.010	0.044	54	0.048	0.102	0.115	-0.001	0.051	227	444	49.6	4.88
IPI00117352.1	Tubulin beta-5 chain	78.83%	2	5	43	1984	-0.023	-0.122	0.122	0.000	0.100	105	-0.279	0.000	-0.099	0.037	-0.106	88	-0.199	-0.183	-0.219	0.141	-0.026	126	-0.085	-0.025	-0.041	-0.074	0.100	143	0.081	0.062	0.040	-0.023	0.011	205	444	49.6	4.89
IPI00122928.1	Tubulin beta-6 chain	78.75%	1	9	43	1323	-0.012	-0.054	0.122	-0.006	0.061	69	-0.091	0.048	0.091	-0.083	-0.038	49	-0.127	-0.176	-0.196	0.169	-0.016	72	-0.142	0.054	-0.295	0.061	-0.167	84	0.048	0.106	0.041	-0.039	0.027	139	447	50.1	4.89
IPI00131309.1	Tubulin gamma-1 chain	96.67%	2	9	46	237	-0.155	-0.208	-0.095	0.315	0.130	3	0.241	0.259	-0.079	0.158	0.167	4							-0.203	0.246	-0.180	0.075	-0.106	13							451	51.1	6.02
IPI00119067.3	Tubulin polymerization-promoting protein	76.15%	1	13	27	159																									0.154	0.176	0.070	0.096	0.092	60	218	23.6	9.42
IPI00133557.1	Tubulin polymerization-promoting protein family member 3	76.14%	1	10	21	211													-0.849	-0.171	-0.884	0.119	-0.685	31	-0.340	-0.047	-0.358	0.004	-0.193	11	0.089	0.171	0.193	0.192	0.294	31	176	19.0	9.11
IPI00315948.3	Tubulin-folding cofactor B	99.59%	1	7	37	167	0.029	-0.047	-0.004	-0.001	-0.012	7	-0.434	-0.057	-0.293	0.107	-0.234	8	-0.584	-0.300	-0.816	0.166	-0.171	5	-0.083	-0.050	-0.176	0.017	-0.180	6	0.037	-0.037	0.258	0.249	0.163	7	244	27.4	5.24
IPI00222972.5	Tubulin-specific chaperone A	85.19%	1	11	17	127	0.192	0.024	0.227	0.072	0.181	7	-0.299	-0.039	-0.308	-0.130	-0.201	13	-0.252	-0.141	-0.134	0.036	-0.001	5	-0.020	0.041	-0.059	0.011	0.020	18							108	12.7	5.27
IPI00461857.2	Tubulin-specific chaperone D	75.50%	4	19	104	396	-0.074	-0.295	-0.167	-0.147	-0.150	17	0.000	-0.257	0.070	-0.060	-0.173	16	-0.127	-0.081	-0.379	0.102	-0.063	4	-0.386	-0.001	-0.299	0.065	-0.322	17							1196	133.2	6.51
IPI00379876.2	Tubulin--tyrosine ligase-like protein 12	65.57%	2	17	48	273	-0.155	-0.042	-0.112	-0.094	-0.020	34	-0.053	0.033	0.064	0.111	-0.050	15	-0.431	-0.212	-0.537	0.158	-0.250	6	-0.028	0.190	0.052	0.004	0.136	15							639	74.0	5.63
IPI00320916.2	Tudor domain-containing protein 1	80.63%	1	4	102	512	0.112	-0.095	0.222	0.030	-0.037	7																									1172	129.6	5.81
IPI00112101.1	Tuftelin-interacting protein 11	78.04%	1	7	73	176							0.399	-0.001	0.179	0.108	-0.082	7																			838	96.2	5.90
IPI00153113.4	Tumor necrosis factor receptor type 1-associated DEATH domain protein	87.74%	3	4	30	96	0.069	-0.152	0.059	0.033	0.152	4																									310	34.6	5.20
IPI00625441.3	tumor protein D52 isoform 1	89.88%	9	10	26	256	0.120	-0.091	0.162	-0.105	0.163	27	-0.271	-0.013	-0.373	-0.208	-0.204	12	-0.175	-0.196	-0.199	0.001	-0.150	10	0.158	-0.083	-0.077	-0.007	0.036	7	0.241	-0.080	0.118	-0.272	-0.311	12	247	26.9	4.96
IPI00319046.1	Tumor protein D54	86.36%	6	7	30	249	-0.032	0.115	-0.065	0.116	-0.076	16	0.251	-0.267	0.283	0.050	0.305	8	-0.233	-0.062	-0.279	0.073	-0.146	12	-0.116	-0.131	0.476	0.153	0.441	3	-0.012	-0.025	-0.134	0.001	-0.102	14	220	24.0	6.15
IPI00120085.1	Tumor protein p63-regulated gene 1-like protein	60.53%	2	4	11	30																									-0.122	-0.405	-0.329	-0.456	-0.276	5	266	29.8	7.37
IPI00117944.1	Tumor susceptibility gene 101 protein	49.87%	3	8	29	132	-0.069	0.080	0.055	-0.010	0.091	9	-0.211	-0.260	-0.167	-0.082	-0.047	3	0.270	0.213	0.277	0.258	0.156	3							0.249	-0.279	0.119	0.152	0.315	5	391	44.1	6.71
IPI00314844.2	Twinfilin-1	58.00%	3	9	33	230	-0.176	-0.083	0.064	0.064	-0.127	18	0.026	0.083	-0.186	-0.194	-0.246	4	0.282	0.226	-0.048	0.433	0.094	5	0.031	0.043	-0.144	0.019	-0.236	13							350	40.1	6.67
IPI00956858.1	type I inositol-3,4-bisphosphate 4-phosphatase isoform 1	76.46%	8	5	76	212																									0.114	0.123	0.165	0.483	0.014	5	939	105.5	6.98
IPI00124800.2	type II hair keratin	84.13%	7	1	44	216							0.523	0.100	0.153	-0.025	0.148	5	0.143	0.093	-0.009	-0.082	-0.069	7							0.080	-0.327	-0.381	-0.371	-0.001	5	479	52.8	5.74
IPI00112648.2	Tyrosine-protein kinase CSK	64.00%	1	5	35	105	-0.076	-0.149	-0.111	-0.047	0.030	13																									450	50.7	7.06
IPI00830433.1	tyrosine-protein kinase receptor UFO isoform 2 precursor	40.50%	4	7	32	81	0.212	-0.071	0.029	0.045	0.310	9																									879	97.2	5.64
IPI00123039.1	Tyrosine-protein phosphatase non-receptor type 1	74.07%	1	4	34	143	0.223	-0.329	0.295	-0.051	0.366	7							0.259	0.139	0.065	0.062	0.141	4	-0.068	-0.238	-0.016	-0.176	-0.057	3							432	49.6	6.16
IPI00337948.3	Tyrosine-protein phosphatase non-receptor type 12	66.19%	1	3	52	104	-0.133	-0.252	0.054	-0.037	-0.072	4																									775	86.5	5.99
IPI00396833.1	Tyrosyl-tRNA synthetase, mitochondrial	69.07%	2	6	44	133																			-0.240	-0.402	-0.472	-0.109	-0.219	7							472	52.6	9.16
IPI00122350.4	U1 small nuclear ribonucleoprotein A	60.98%	7	7	24	337	0.030	0.048	0.070	0.020	0.047	34	0.083	-0.045	-0.025	-0.175	0.003	21	0.134	0.055	0.156	-0.120	0.075	19	0.055	0.016	0.152	0.007	0.079	17							287	31.8	9.80
IPI00170008.1	U2 small nuclear ribonucleoprotein A'	84.71%	2	10	34	242	-0.016	0.065	0.066	0.041	0.026	10	0.151	0.130	-0.142	-0.091	0.099	12	0.024	0.101	0.103	0.020	0.044	16	-0.016	0.089	0.047	0.062	0.045	11							255	28.3	8.62
IPI00132576.1	U2 small nuclear ribonucleoprotein B''	65.33%	2	6	19	126	-0.051	0.009	0.027	0.029	-0.191	9	0.104	0.010	-0.136	0.059	0.023	7	0.080	-0.073	-0.035	-0.144	0.017	3	0.152	0.016	0.043	0.023	0.023	10	0.292	-0.050	0.220	0.337	0.136	3	225	25.3	9.72
IPI00402911.2	U3 small nucleolar ribonucleoprotein protein MPP10	55.65%	3	9	53	182	0.110	0.185	0.027	0.024	0.073	4	0.180	-0.020	0.110	0.003	0.114	5	0.077	-0.073	-0.292	0.054	0.007	5	0.013	-0.127	-0.017	-0.150	-0.044	4							681	78.7	4.87
IPI00226889.1	U3 small nucleolar RNA-associated protein 15 homolog	87.12%	1	8	58	161	-0.152	0.308	-0.102	0.328	-0.109	4	-0.155	-0.013	-0.398	-0.099	-0.306	3	-0.086	0.038	0.123	0.048	0.116	5	0.073	-0.097	0.198	-0.091	0.147	5							528	59.3	8.79
IPI00353579.1	U3 small nucleolar RNA-associated protein 18 homolog	92.39%	2	9	63	243	-0.253	-0.051	-0.125	0.058	-0.122	4							-0.017	0.115	0.087	-0.022	-0.267	3	-0.167	-0.404	-0.170	-0.176	-0.191	3							552	61.2	8.78
IPI00128256.1	U3 small nucleolar RNA-interacting protein 2	90.32%	2	6	48	155	-0.258	0.023	-0.388	-0.100	-0.208	4							-0.083	-0.023	-0.029	-0.133	-0.039	4	-0.139	0.108	0.047	0.036	-0.059	7							475	52.1	7.88
IPI00331170.1	U4/U6 small nuclear ribonucleoprotein Prp3	73.94%	2	8	84	298	-0.180	0.041	-0.083	0.007	-0.149	3	0.222	0.171	0.062	0.208	0.230	5	-0.017	-0.095	0.058	-0.194	-0.044	5													683	77.4	9.50
IPI00458908.2	U4/U6 small nuclear ribonucleoprotein Prp4	79.85%	1	9	45	145	-0.060	-0.313	-0.066	-0.173	-0.004	4	-0.154	-0.122	-0.228	-0.134	-0.175	13	0.038	-0.004	-0.044	0.009	0.047	8													521	58.3	7.28
IPI00323674.6	U4/U6.U5 tri-snRNP-associated protein 1	77.42%	1	19	97	382	-0.010	-0.028	-0.083	-0.013	0.085	21	0.088	0.083	0.066	-0.069	0.048	16	0.013	0.009	0.072	0.010	-0.079	18	-0.092	0.134	-0.199	0.276	-0.133	3							806	90.8	5.82
IPI00457815.3	U4/U6.U5 tri-snRNP-associated protein 2	72.87%	1	4	44	94	-0.329	0.043	0.037	0.024	-0.165	4													-0.089	-0.102	0.040	-0.103	-0.031	7							564	65.1	8.90
IPI00461621.1	U5 small nuclear ribonucleoprotein 40 kDa protein	88.55%	2	12	28	326	0.029	-0.125	0.006	0.021	0.066	18	-0.050	-0.057	-0.023	-0.004	-0.010	14	0.108	0.033	0.088	0.029	0.103	13	0.104	-0.051	0.119	-0.089	0.050	26							358	39.3	8.10
IPI00876050.1	U6 snRNA-associated Sm-like protein LSm2	93.68%	3	3	12	40							-0.604	0.040	-0.346	-0.176	-0.038	5	0.136	0.089	0.616	0.332	0.424	3													95	10.8	6.52
IPI00877286.1	U6 snRNA-associated Sm-like protein LSm4	83.33%	2	4	17	70							-0.020	-0.029	-0.016	0.083	-0.092	4																			138	15.2	10.05
IPI00129873.1	U6 snRNA-associated Sm-like protein LSm6	92.50%	1	1	12	61							0.595	-0.257	0.156	-0.029	-0.038	3	-0.119	-0.126	0.003	0.111	-0.054	5	0.010	-0.157	0.092	-0.103	0.112	4							80	9.1	9.58
IPI00132901.1	U6 snRNA-associated Sm-like protein LSm7	82.52%	1	4	11	22													-0.390	-0.172	-0.283	-0.017	-0.175	3	0.091	0.079	0.019	0.027	0.033	3							103	11.6	5.27
IPI00387416.2	Ubiquilin-2	39.81%	1	9	34	989	0.246	0.076	0.353	0.023	0.360	20	-0.229	0.016	-0.208	0.036	-0.288	34	-0.020	-0.036	0.070	0.073	0.178	8	-0.529	0.127	-0.500	0.079	-0.355	67	0.007	-0.007	-0.023	-0.223	-0.009	20	638	67.3	5.22
IPI00118575.1	Ubiquilin-4	59.23%	1	9	37	323							-0.069	-0.020	-0.171	-0.059	-0.057	20							-0.193	0.190	-0.047	0.175	-0.158	10							596	63.5	5.03
IPI00169862.1	Ubiquinone biosynthesis protein COQ9, mitochondrial	76.68%	2	8	30	222	0.163	0.338	-0.322	0.004	0.296	4													-0.385	-0.304	-0.383	-0.183	-0.320	12	-0.069	-0.302	-0.134	-0.218	-0.016	8	313	35.1	5.92
IPI00881918.1	Ubiquitin carboxyl-terminal hydrolase	82.28%	4	32	71	765	-0.136	-0.130	-0.012	-0.079	-0.017	56	-0.246	-0.028	-0.094	0.020	-0.094	56	-0.006	-0.139	0.023	0.085	0.195	12	0.003	0.087	0.021	0.079	-0.010	52	0.003	-0.059	-0.004	-0.080	-0.092	53	835	93.3	5.06
IPI01007734.1	Ubiquitin carboxyl-terminal hydrolase	78.13%	5	23	92	291	0.006	-0.168	0.010	-0.022	0.000	7	-0.293	0.079	-0.278	0.119	-0.262	18	0.007	0.023	0.010	0.057	0.010	4	-0.109	-0.130	0.003	-0.086	0.104	7							1143	132.8	5.66
IPI00270877.5	Ubiquitin carboxyl-terminal hydrolase 14	94.52%	2	27	66	603	-0.006	-0.037	0.000	-0.050	0.004	48	-0.149	-0.041	-0.144	-0.068	-0.102	52	-0.181	-0.148	-0.146	0.184	0.024	24	-0.241	0.031	-0.346	-0.010	-0.344	24	0.088	-0.086	-0.047	0.038	0.013	11	493	56.0	5.24
IPI00115701.2	Ubiquitin carboxyl-terminal hydrolase 4	71.10%	2	11	63	207	-0.163	-0.082	-0.077	-0.086	-0.120	16	0.144	0.122	-0.077	0.047	-0.112	8																			962	108.3	5.64
IPI00313962.3	Ubiquitin carboxyl-terminal hydrolase isozyme L1	83.41%	2	13	22	375																			0.114	0.183	0.019	0.062	0.044	41	0.045	-0.208	-0.245	-0.452	-0.253	83	223	24.8	5.24
IPI00311369.1	Ubiquitin carboxyl-terminal hydrolase isozyme L3	69.13%	4	8	20	177	-0.458	-0.162	-0.051	0.041	-0.066	14													-0.045	-0.014	-0.088	-0.025	0.021	14	0.130	-0.206	-0.108	-0.066	-0.023	16	230	26.1	5.05
IPI00112597.11	Ubiquitin conjugation factor E4 B	86.70%	6	22	123	597	0.123	0.083	0.415	0.020	0.336	10	0.065	0.207	0.050	0.026	0.000	17							-0.051	0.001	-0.094	0.065	-0.086	18							1173	133.2	6.07
IPI00131915.2	Ubiquitin domain-containing protein UBFD1	55.43%	2	5	27	87																									-0.168	-0.112	-0.130	-0.173	0.081	5	368	40.1	8.85
IPI00656165.2	Ubiquitin fusion degradation protein 1 homolog	60.91%	1	2	25	110	0.145	0.158	-0.020	-0.079	0.003	5							0.498	0.093	0.314	0.084	0.120	7	0.179	0.323	0.185	0.171	-0.258	4	0.007	0.066	-0.051	0.346	-0.308	3	307	34.5	6.70
IPI00154004.1	Ubiquitin thioesterase OTUB1	91.51%	3	11	23	407	0.045	-0.006	0.077	-0.044	0.091	26	-0.519	-0.092	-0.452	-0.102	-0.214	29	-0.318	-0.122	-0.274	0.148	-0.243	20	-0.269	0.111	-0.349	0.048	-0.253	22	0.010	0.153	0.116	0.165	0.154	37	271	31.3	4.94
IPI00470152.2	Ubiquitin-40S ribosomal protein S27a	87.82%	12	6	22	674	0.041	-0.105	0.085	-0.038	0.185	44	-0.105	0.100	-0.066	0.104	-0.083	45	0.194	0.046	0.052	0.051	0.062	46	0.313	0.163	0.362	0.081	0.091	43	0.058	-0.069	0.011	-0.117	-0.160	35	156	17.9	9.64
IPI00457533.2	Ubiquitin-associated protein 2	50.27%	4	21	66	738	0.019	-0.037	0.084	0.001	0.083	43	0.031	0.052	-0.189	-0.074	0.075	48	-0.181	0.001	-0.201	0.073	-0.069	39	0.209	0.034	0.214	0.077	0.192	15							1132	117.9	7.72
IPI00125135.1	Ubiquitin-conjugating enzyme E2 D2	56.46%	4	3	12	61							-0.771	0.110	-0.595	0.187	-0.494	8	-0.162	-0.086	-0.357	0.088	0.255	3							0.258	0.127	0.061	-0.020	0.104	4	147	16.7	7.83
IPI00321895.1	Ubiquitin-conjugating enzyme E2 J1	85.85%	3	7	33	148	0.228	0.083	0.172	0.136	0.068	6	-0.360	-0.252	-0.540	0.184	-0.201	4	-0.072	-0.040	-0.196	-0.170	-0.105	10													318	35.0	6.99
IPI00322440.5	Ubiquitin-conjugating enzyme E2 K	96.50%	3	8	25	152	-0.168	-0.086	-0.157	-0.063	-0.122	16	-0.542	-0.095	-0.413	-0.317	-0.208	15	-0.417	-0.348	0.050	0.021	-0.119	4	0.527	0.100	0.395	0.092	0.413	5	-0.004	-0.051	0.031	-0.004	0.099	8	200	22.4	5.44
IPI00128760.1	Ubiquitin-conjugating enzyme E2 L3	86.36%	9	8	16	220	-0.062	-0.112	-0.004	-0.022	0.016	24	-0.776	0.112	-0.573	0.030	-0.371	16	-0.405	-0.182	-0.400	0.136	-0.173	7	0.321	0.096	0.303	0.024	0.268	17	-0.125	-0.013	-0.010	-0.023	-0.091	26	154	17.9	8.51
IPI00165854.3	Ubiquitin-conjugating enzyme E2 N	98.68%	5	7	21	324	0.011	-0.112	0.151	-0.025	0.187	25	-0.366	0.061	-0.221	0.013	-0.257	11	-0.351	-0.133	-0.283	0.148	-0.125	14	0.070	-0.023	0.062	0.043	0.185	25	0.037	0.057	0.026	-0.042	-0.001	37	152	17.1	6.57
IPI00459054.6	ubiquitin-conjugating enzyme E2 N-like	75.14%	1	1	9	123	-0.051	-0.139	0.218	0.003	0.187	10	-0.776	0.167	-0.221	0.179	-0.250	3	-0.368	-0.220	-0.394	-0.059	-0.170	4	0.089	-0.035	0.110	-0.004	0.253	9	0.095	0.021	0.061	-0.054	-0.004	14	173	19.3	4.92
IPI00453803.6	Ubiquitin-conjugating enzyme E2 O	66.69%	1	11	91	273							0.088	-0.010	0.029	0.094	-0.274	6							-0.498	0.050	-0.427	0.024	-0.496	4	0.017	-0.181	0.045	-0.083	-0.139	10	1288	140.7	5.06
IPI00229310.1	Ubiquitin-conjugating enzyme E2 R1	81.28%	1	1	14	22	-0.079	-0.158	-0.199	-0.082	-0.162	5																									235	26.6	4.56
IPI00311591.3	Ubiquitin-conjugating enzyme E2 Z	43.54%	1	4	23	113	-0.228	-0.083	-0.147	-0.082	0.009	4	-0.178	-0.106	-0.219	-0.317	-0.167	4																			356	38.3	5.62
IPI00988395.1	ubiquitin-like domain-containing CTD phosphatase 1-like isoform 1	70.97%	3	3	28	116	-0.173	0.076	-0.213	0.000	-0.065	3	0.069	0.141	0.360	-0.117	0.600	5																			310	35.9	6.01
IPI00123313.1	Ubiquitin-like modifier-activating enzyme 1	95.65%	1	43	99	2215	0.006	-0.082	0.079	-0.026	0.057	177	-0.028	-0.013	0.087	0.011	0.040	114	-0.333	-0.173	-0.269	0.122	-0.119	92	0.084	0.069	0.134	0.031	0.115	188	0.029	-0.037	-0.020	-0.085	-0.044	160	1058	117.7	5.66
IPI00337053.3	Ubiquitin-like modifier-activating enzyme 1 Y	69.66%	1	1	66	462	0.026	-0.083	0.092	-0.050	0.094	25	-0.097	-0.054	0.000	-0.099	-0.048	13	-0.510	-0.268	-0.349	0.099	-0.123	10	0.156	0.065	0.128	0.010	0.108	22	0.081	-0.023	0.030	-0.010	-0.019	27	1058	118.0	5.55
IPI00311833.6	Ubiquitin-like modifier-activating enzyme 5	81.39%	3	4	32	105	0.128	0.077	0.065	-0.626	-0.117	3																			-0.056	-0.127	-0.228	-0.142	-0.158	6	403	44.8	4.96
IPI00226815.1	Ubiquitin-like modifier-activating enzyme 6	64.29%	2	12	79	251							-0.510	-0.091	-0.398	-0.525	-0.044	8	0.286	0.059	1.096	-0.127	0.563	5													1053	117.9	6.11
IPI00987739.1	ubiquitin-like modifier-activating enzyme ATG7-like	65.83%	4	1	52	175													0.308	-0.036	-0.020	0.302	0.095	5							0.295	-0.032	-0.091	0.024	0.041	4	638	70.8	7.78
IPI00471341.2	Ubiquitin-like protein 4A	90.45%	1	6	20	108	0.033	0.030	-0.056	-0.056	0.007	6	-0.035	0.011	-0.068	0.011	-0.181	8																			157	17.8	8.44
IPI00399869.2	Ubiquitin-like protein 7	49.47%	1	4	13	26							-0.114	-0.059	0.016	-0.012	-0.092	3							-0.092	0.085	0.014	0.083	-0.060	3							380	40.4	5.01
IPI00987848.1	ubiquitin-like protein ISG15-like	92.61%	2	2	36	544	0.017	-0.086	0.084	-0.047	0.176	33	-0.238	0.100	-0.092	0.085	-0.139	21	0.170	-0.057	-0.038	0.066	0.061	24	0.313	0.017	0.308	0.055	0.041	29	0.058	-0.020	0.089	0.001	-0.041	26	203	22.9	9.82
IPI00132142.1	Ubiquitin-like-conjugating enzyme ATG3	55.41%	1	4	20	62							0.084	0.230	0.287	-0.034	0.474	4																			314	35.8	4.72
IPI00115595.1	Ubiquitin-protein ligase E3A	78.19%	1	1	77	279	-0.290	0.003	-0.379	-0.304	-0.245	10	-0.035	-0.010	-0.031	-0.162	0.087	12							-0.127	0.001	-0.167	-0.127	-0.063	6							885	101.1	5.07
IPI00228830.3	Ubiquitin-protein ligase E3C	61.77%	1	3	67	218																			0.158	-0.199	0.030	-0.100	0.190	4							1083	123.9	6.39
IPI00123589.1	UBX domain-containing protein 1	87.88%	1	9	28	184	0.108	-0.074	0.286	0.026	0.270	12	-0.431	-0.062	-0.335	0.009	-0.206	12							-0.313	0.069	-0.194	-0.117	-0.188	11							297	33.6	5.26
IPI00165799.1	UBX domain-containing protein 4	69.17%	1	7	39	298	0.080	-0.056	0.120	0.048	0.107	14	-0.283	-0.231	-0.246	-0.248	-0.335	13	0.069	0.189	0.239	-0.012	0.019	13	-0.482	0.019	-0.786	-0.234	-0.630	5							506	56.4	6.61
IPI00751399.2	UBX domain-containing protein 7	62.17%	2	2	30	70																			1.218	0.187	1.375	0.055	1.190	4							489	54.8	5.16
IPI00417182.2	UDP glucuronosyltransferase 1 family, polypeptide A5	73.35%	5	1	37	136	0.152	0.000	0.036	0.033	0.463	6																									529	60.0	8.09
IPI00416861.2	UDP glucuronosyltransferase 1 family, polypeptide A6B	72.88%	5	1	40	156	-0.032	-0.025	0.000	0.000	0.257	9																									531	60.5	8.69
IPI00153129.1	UDP-glucose 4-epimerase	63.11%	1	7	20	90	0.094	-0.231	-0.048	-0.119	0.044	13													-0.404	-0.074	-0.432	0.016	-0.012	4							347	38.2	6.74
IPI00118344.1	UDP-glucose 6-dehydrogenase	84.79%	3	24	49	508	0.408	-0.065	0.447	-0.068	0.457	46	-0.651	-0.050	-0.496	-0.082	-0.466	41	-0.048	-0.143	0.205	0.052	0.100	8													493	54.8	7.56
IPI00762897.3	UDP-glucose:glycoprotein glucosyltransferase 1	82.85%	7	45	161	1511	-0.085	0.072	-0.150	0.080	-0.106	48	0.268	0.104	0.381	0.147	0.149	63	0.023	0.044	0.019	0.091	-0.013	35	-0.173	-0.054	-0.218	-0.014	-0.216	98							1551	176.3	5.62
IPI00129252.3	UDP-glucuronic acid decarboxylase 1	55.95%	1	1	28	95							-0.114	-0.076	0.165	0.220	0.062	7																			420	47.5	8.94
IPI00134432.3	UDP-glucuronosyltransferase 1-6	72.32%	7	2	43	171	-0.032	-0.025	-0.003	0.000	0.103	11																									531	60.4	8.62
IPI00417181.3	UDP-glucuronosyltransferase 1-7C	71.75%	11	7	35	123	0.329	0.024	0.183	-0.006	0.353	21																									531	59.7	8.35
IPI00762371.3	UHRF1-binding protein 1-like	69.18%	2	3	108	263	-0.134	-0.048	0.024	0.148	0.062	4																									1457	161.8	6.61
IPI00331146.5	UMP-CMP kinase	74.01%	1	9	27	299	0.220	-0.150	0.250	-0.086	0.465	24	-0.373	-0.013	-0.198	-0.233	-0.134	13	-0.464	-0.145	-0.488	0.169	-0.381	9	-0.082	0.091	-0.171	0.068	-0.178	6	0.110	0.006	-0.065	-0.099	-0.127	28	227	25.7	7.97
IPI00112004.1	Uncharacterized protein	86.18%	1	4	21	83	-0.041	-0.025	-0.160	0.009	-0.057	7																									123	14.1	11.47
IPI00112190.5	Uncharacterized protein	58.68%	2	1	23	77													0.777	0.143	0.766	-0.136	0.350	3							-0.157	0.367	0.084	0.282	0.192	3	317	35.2	9.16
IPI00112407.3	Uncharacterized protein	92.72%	1	2	27	168	0.092	0.106	0.077	0.138	-0.001	5	0.019	-0.423	-0.392	-0.184	0.013	5	-0.003	0.113	0.004	0.075	-0.004	3	-0.162	0.098	-0.111	0.108	-0.141	13							151	16.3	10.11
IPI00114925.1	Uncharacterized protein	71.31%	1	11	30	173	0.001	-0.128	0.085	-0.120	0.207	8	-0.396	0.045	-0.402	-0.204	-0.181	11	-0.271	-0.130	-0.472	0.106	-0.105	5	0.003	0.120	0.145	0.027	0.057	8	-0.265	-0.163	0.075	0.247	-0.311	3	359	40.0	7.06
IPI00116247.1	Uncharacterized protein	90.19%	4	10	37	164	-0.069	-0.165	0.062	-0.152	0.083	13	0.325	-0.112	0.809	0.057	0.571	5																			377	42.5	5.26
IPI00117932.3	Uncharacterized protein	75.82%	8	13	113	420	0.199	0.084	-0.171	0.062	-0.059	6	0.047	0.102	0.009	0.162	0.023	10	0.014	-0.015	0.170	-0.149	0.199	4	-0.264	-0.072	-0.299	-0.035	-0.245	8							1282	146.0	7.12
IPI00118413.2	Uncharacterized protein	71.31%	1	17	97	396							-0.116	0.314	-0.139	0.179	-0.302	24	0.140	-0.037	0.448	0.004	0.001	4													1171	129.6	4.94
IPI00118590.1	Uncharacterized protein	79.79%	1	4	37	208	0.030	0.058	0.092	-0.029	0.055	3	0.623	-0.044	0.336	-0.240	0.497	3	0.944	0.287	0.646	0.106	0.638	11	-0.231	0.020	-0.131	-0.051	-0.128	9							188	20.1	11.22
IPI00119575.3	Uncharacterized protein	67.45%	5	8	58	356	0.112	-0.013	0.036	-0.080	0.212	11	0.092	-0.029	0.081	-0.092	0.062	40	-0.621	-0.105	-0.468	-0.109	-0.250	6	0.041	-0.127	-0.114	-0.071	0.024	19	-0.026	0.348	0.149	0.156	0.175	13	510	57.2	6.89
IPI00120023.1	Uncharacterized protein	72.96%	3	12	28	133	0.072	0.081	0.036	0.131	-0.053	5																			0.040	-0.080	0.019	-0.051	0.016	27	270	31.1	8.31
IPI00120674.2	Uncharacterized protein	56.66%	7	1	82	217	0.178	0.289	0.335	0.019	0.239	4																									1232	135.9	8.21
IPI00121338.1	Uncharacterized protein	60.94%	5	4	45	127																			0.587	0.017	0.552	-0.022	0.650	5							763	86.7	4.75
IPI00121827.4	Uncharacterized protein	58.30%	10	2	66	224	0.026	-0.327	-0.188	-0.111	0.187	5																			-0.199	-0.116	-0.155	-0.128	-0.022	4	1103	123.3	5.08
IPI00122202.5	Uncharacterized protein	71.22%	2	17	93	466	-0.037	0.096	-0.072	-0.034	-0.108	21	-0.162	-0.013	0.112	0.011	-0.183	21							0.185	-0.029	0.230	-0.068	0.140	11							1004	112.8	5.55
IPI00122696.6	Uncharacterized protein	62.91%	2	6	33	248	0.041	-0.037	0.089	0.054	0.026	13	-0.508	-0.108	-0.458	-0.085	-0.492	11	0.122	0.004	-0.007	0.085	-0.004	29	0.350	0.116	0.157	0.068	0.241	15	-0.032	0.068	0.154	0.077	0.128	6	461	51.7	5.05
IPI00124248.1	Uncharacterized protein	69.29%	2	6	14	150	-0.209	-0.076	-0.386	-0.186	-0.368	10	-0.001	0.003	-0.233	-0.089	-0.150	15	-0.429	-0.138	-0.655	0.043	-0.257	3	-0.171	-0.003	-0.100	-0.071	0.051	10							241	26.5	4.12
**IPI00124959.2**	**Uncharacterized protein**	**91.50%**	**1**	**78**	**439**	**2332**	-0.029	0.084	-0.051	0.024	-0.072	**143**	0.235	0.000	0.239	-0.003	0.181	**84**	0.374	0.073	0.384	0.095	0.331	**21**	0.228	-0.079	0.229	-0.035	0.212	**69**							**3177**	**350.7**	**9.72**
IPI00126389.2	Uncharacterized protein	72.85%	2	3	67	297	0.003	0.019	-0.271	-0.112	-0.189	8	0.020	-0.102	0.112	-0.048	0.176	19	0.107	0.072	0.119	-0.248	-0.060	13	-0.580	-0.083	-0.191	0.047	-0.224	3							674	74.2	6.23
IPI00127109.4	Uncharacterized protein	76.39%	1	1	18	278	-0.063	-0.119	0.126	0.020	-0.054	16	-0.274	0.120	-0.123	0.031	-0.252	31	-0.108	-0.090	-0.001	0.179	0.083	25	0.222	-0.106	0.062	-0.031	0.410	20	-0.057	0.091	0.062	0.010	0.019	21	216	24.3	7.97
IPI00127408.3	Uncharacterized protein	61.61%	9	5	20	384	0.151	-0.056	0.198	-0.102	0.322	28	0.034	0.085	0.197	0.107	0.098	41	-0.042	-0.077	-0.048	0.034	0.006	36	-0.088	0.184	-0.071	0.066	-0.097	20	-0.088	0.108	0.048	0.210	0.037	43	211	23.4	8.69
IPI00130013.4	Uncharacterized protein	55.01%	7	10	25	410	0.069	-0.116	0.149	-0.072	0.077	31	0.208	-0.133	0.194	-0.032	0.246	26	-0.448	-0.075	-0.409	0.120	-0.415	7	-0.060	0.122	0.055	0.059	-0.012	18	0.030	-0.062	0.286	0.154	0.322	9	389	42.2	5.10
IPI00131692.8	Uncharacterized protein	86.11%	9	4	116	357							-0.108	0.081	-0.240	0.132	0.153	4													-0.201	0.167	-0.039	-0.138	0.145	3	864	99.7	5.01
IPI00132347.1	Uncharacterized protein	94.59%	4	10	19	200	-0.085	-0.041	0.030	-0.020	-0.022	8	0.087	-0.003	-0.054	-0.120	-0.022	6	0.080	0.118	-0.062	-0.048	-0.045	15	-0.012	-0.089	-0.076	-0.006	0.058	14	-0.047	-0.243	-0.004	-0.203	-0.039	27	111	13.6	9.11
IPI00132604.3	Uncharacterized protein	54.00%	3	1	20	103	0.249	0.095	0.261	-0.094	0.263	4																									350	38.6	7.64
IPI00134920.3	Uncharacterized protein	96.49%	5	1	9	204	0.373	0.257	-0.105	0.151	0.365	3	-0.108	-0.086	-0.074	0.119	-0.086	11	0.512	0.205	0.460	0.131	0.210	13	0.001	0.061	-0.531	0.064	0.120	5							114	11.4	4.36
IPI00136459.1	Uncharacterized protein	81.85%	3	1	29	60	-0.431	-0.086	-0.375	0.204	-0.311	3							-0.054	-0.316	-0.088	-0.619	-0.504	3													248	29.1	8.21
IPI00136572.4	Uncharacterized protein	86.04%	3	13	110	462	-0.188	-0.047	-0.293	-0.025	-0.318	3	-0.004	-0.257	0.029	0.001	-0.216	4	0.379	0.076	0.472	0.047	0.184	6	-0.206	-0.091	-0.095	-0.141	-0.071	8							996	112.5	8.07
IPI00138089.5	Uncharacterized protein	78.94%	2	14	71	400	0.000	0.062	-0.218	-0.199	-0.082	8	0.124	0.036	0.122	0.014	0.138	11	-0.088	0.038	-0.041	-0.083	-0.068	6	-0.138	0.171	-0.048	0.094	-0.028	14							603	65.9	8.76
IPI00154057.3	Uncharacterized protein	59.34%	3	1	50	230													0.227	0.095	0.066	0.387	0.274	3	-0.269	-0.077	-0.482	-0.012	-0.262	13	-0.272	-0.158	-0.134	-0.139	-0.320	16	1038	112.3	5.02
IPI00169699.4	Uncharacterized protein	84.88%	8	3	36	141																									0.068	-0.039	-0.100	0.038	-0.082	4	291	32.2	8.84
IPI00170310.1	Uncharacterized protein	39.52%	3	11	42	142																									0.247	0.174	0.077	0.023	0.218	26	921	100.6	5.12
IPI00221501.3	Uncharacterized protein	86.67%	3	3	10	21																									0.059	-0.127	0.041	0.000	-0.180	5	120	13.5	8.43
IPI00224729.1	Uncharacterized protein	81.99%	3	7	45	817	0.103	0.013	0.050	-0.031	0.163	49	0.134	0.004	0.190	0.107	-0.013	41	0.195	0.100	0.099	-0.034	0.047	55	-0.089	-0.139	-0.106	-0.032	-0.060	66	-0.120	-0.063	0.111	0.031	0.045	22	472	51.2	6.80
IPI00226149.6	Uncharacterized protein	85.81%	2	18	63	606	-0.109	0.104	-0.130	0.072	-0.281	33	0.174	0.011	-0.050	-0.057	0.116	41	-0.054	0.027	0.010	-0.100	0.003	39	0.095	0.026	0.108	0.006	0.026	31							465	51.9	9.98
IPI00226205.3	Uncharacterized protein	50.69%	4	5	22	71							-0.065	0.001	0.010	0.107	-0.053	13																			436	46.7	8.68
IPI00226872.1	Uncharacterized protein	91.67%	2	10	36	245	-0.116	0.103	0.115	-0.037	0.029	5	-0.697	-0.044	-0.385	-0.238	-0.429	3	-0.324	-0.122	-0.364	-0.204	-0.317	8							0.175	-0.308	-0.373	-0.250	-0.204	41	240	26.8	5.14
IPI00226930.2	Uncharacterized protein	72.34%	1	8	35	109	0.023	0.045	0.011	0.087	-0.037	9	-0.176	0.151	0.066	0.033	-0.056	8							0.355	0.047	0.195	0.041	0.195	5							423	50.2	6.42
IPI00229300.3	Uncharacterized protein	92.89%	4	1	102	748	-0.141	0.033	-0.071	-0.060	-0.072	3	0.270	0.193	0.193	0.267	-0.109	10	0.131	-0.040	0.098	-0.020	0.149	25	-0.427	-0.100	-0.450	-0.031	-0.434	35	-0.003	0.180	0.162	0.157	0.106	131	858	95.6	5.45
IPI00230021.5	Uncharacterized protein	71.79%	16	35	241	925																									-0.258	-0.272	0.249	0.096	-0.060	97	2730	310.9	6.21
IPI00230680.7	Uncharacterized protein	69.34%	2	2	85	514	0.315	0.114	0.183	0.088	0.255	18	0.122	0.131	-0.045	-0.092	-0.038	16	0.256	0.085	0.128	-0.088	0.095	16	-0.131	-0.133	-0.322	-0.007	-0.116	9	0.044	0.151	0.161	0.083	0.094	51	1034	114.0	5.81
IPI00264652.3	Uncharacterized protein	71.27%	2	9	48	880	0.132	0.044	0.054	0.017	0.138	36	0.161	-0.012	0.347	0.038	0.204	30	0.088	0.048	0.104	-0.123	0.119	17	-0.510	0.045	-0.236	0.023	-0.271	30	-0.017	0.111	0.069	0.040	0.151	4	557	58.6	8.40
IPI00265358.1	Uncharacterized protein	65.07%	1	3	106	338							-0.241	0.415	-0.304	-0.180	-0.037	5																			1228	140.5	6.49
IPI00268433.3	Uncharacterized protein	40.96%	5	5	41	93																									0.000	-0.074	-0.223	-0.100	-0.162	7	664	74.3	5.06
IPI00270326.1	Uncharacterized protein	92.00%	1	26	65	749	0.076	-0.035	0.037	0.000	0.027	53	0.073	-0.059	0.134	-0.037	0.183	36	-0.112	-0.092	-0.133	-0.035	-0.048	51	0.055	0.079	0.066	0.020	0.055	44	-0.032	0.052	0.001	0.043	0.026	36	475	52.8	6.28
IPI00270737.5	Uncharacterized protein	75.73%	16	11	63	356	-0.216	-0.068	-0.092	-0.032	-0.094	14	-0.142	-0.089	-0.028	0.040	0.034	29	-0.089	0.006	-0.060	-0.221	0.052	10	-0.597	-0.083	-0.589	-0.023	-0.454	7							614	68.8	7.42
IPI00272854.2	Uncharacterized protein	61.95%	3	2	41	158																									-0.127	0.165	0.225	0.357	-0.016	3	523	58.3	8.85
IPI00276515.4	Uncharacterized protein	51.83%	2	1	39	146																									0.024	0.274	0.258	0.214	0.257	34	627	69.3	5.10
IPI00281164.5	Uncharacterized protein	55.19%	1	3	45	137																									-0.079	-0.194	-0.167	-0.004	-0.028	4	752	87.2	6.77
IPI00281245.2	Uncharacterized protein	81.71%	1	1	17	42	-0.218	0.057	-0.091	-0.013	-0.117	3	0.286	0.104	0.140	0.147	0.107	3																			175	20.2	9.91
IPI00283755.6	Uncharacterized protein	74.95%	6	3	330	864	0.000	0.076	-0.105	-0.063	-0.091	3																									4068	466.4	6.28
IPI00311344.4	Uncharacterized protein	81.25%	6	4	113	1353	-0.283	-0.023	-0.226	-0.014	-0.288	89	0.061	-0.001	-0.013	0.026	-0.183	146	0.148	-0.058	0.227	-0.077	0.280	77	0.202	0.058	0.115	-0.006	0.202	30							768	89.2	5.50
IPI00312018.6	Uncharacterized protein	83.22%	1	14	37	237	0.061	0.023	0.084	0.020	0.084	23	-0.106	0.030	-0.123	0.095	0.016	33	0.210	-0.030	0.160	-0.117	0.065	8	-0.103	-0.076	-0.086	0.017	0.124	3							292	32.4	6.05
IPI00312527.4	Uncharacterized protein	77.11%	2	2	60	800	0.301	-0.091	0.317	-0.111	0.355	16	0.170	0.076	0.569	0.193	0.197	11	0.219	-0.123	0.069	0.010	0.421	15	-0.576	0.066	-0.653	0.057	-0.624	62	0.080	0.102	0.062	0.016	0.052	145	686	74.2	6.81
IPI00314153.4	Uncharacterized protein	82.45%	2	30	76	557	-0.133	0.003	-0.076	-0.003	-0.175	32	-0.304	-0.042	-0.267	-0.144	-0.240	46	-0.458	-0.129	-0.444	0.061	-0.218	22	-0.042	0.066	-0.023	0.031	-0.083	55	-0.079	0.102	-0.059	0.000	0.024	21	564	63.0	8.44
IPI00323927.3	Uncharacterized protein	70.82%	5	6	41	88	0.253	0.153	0.303	0.193	0.425	3	-0.563	-0.108	-0.421	-0.377	-0.285	3																			634	69.8	7.81
IPI00330406.2	Uncharacterized protein	73.38%	1	17	93	388	0.070	0.149	0.089	0.027	0.087	25	0.172	0.061	-0.071	0.152	-0.074	8	0.080	0.060	-0.056	-0.029	0.004	14	0.016	-0.297	0.111	-0.131	0.224	3							819	95.4	9.58
IPI00331016.1	Uncharacterized protein	60.19%	2	13	63	315	-0.006	-0.106	-0.685	-0.204	-0.342	6	-0.478	0.062	-0.423	-0.229	-0.329	10	0.184	-0.023	0.098	-0.072	0.033	3	-0.092	0.069	-0.154	0.016	-0.438	7							1251	135.5	6.96
IPI00331182.4	Uncharacterized protein	93.42%	1	34	90	690	0.124	0.100	0.041	-0.031	0.009	38	0.007	0.047	-0.006	0.116	-0.133	9	0.179	0.096	0.193	0.023	0.023	53	0.135	0.061	0.276	0.079	0.242	7	-0.050	-0.004	-0.038	-0.017	0.045	76	745	82.8	6.42
IPI00336281.2	Uncharacterized protein	91.39%	2	5	189	964							-0.358	-0.297	-0.531	-0.371	-0.260	4							-0.076	-0.079	0.148	-0.133	0.126	3							1487	167.2	5.38
IPI00336313.2	Uncharacterized protein	67.58%	4	7	91	259																									0.132	-0.045	-0.117	-0.188	-0.013	10	1095	122.9	5.14
IPI00336503.1	Uncharacterized protein	76.43%	7	16	83	406	0.153	0.045	0.000	-0.072	0.030	24	0.096	-0.060	0.040	0.024	0.026	27	-0.050	-0.026	-0.297	-0.147	-0.158	9	-0.028	-0.047	-0.025	0.013	0.014	10							789	87.4	5.67
IPI00339468.4	Uncharacterized protein	77.67%	3	47	139	1410	-0.044	0.040	-0.162	0.010	-0.053	77	-0.041	0.029	-0.120	0.064	-0.035	91	0.149	0.064	0.114	0.011	0.029	102	-0.013	-0.042	0.027	-0.028	-0.059	80	-0.079	0.088	0.058	0.135	0.174	17	1384	149.6	6.83
IPI00340815.3	Uncharacterized protein	81.08%	2	9	116	433	-0.167	-0.056	-0.173	0.041	-0.196	8	-0.404	-0.062	-0.155	0.112	-0.680	5							-0.025	-0.139	0.214	-0.079	0.174	10							962	105.7	10.08
IPI00342603.7	Uncharacterized protein	70.65%	1	23	98	533	0.174	0.140	0.076	-0.032	0.130	10	0.614	0.192	0.753	0.077	0.941	5	0.143	-0.002	-0.047	-0.071	0.040	12	-0.269	-0.062	-0.383	-0.007	-0.299	22	-0.014	-0.080	-0.022	-0.017	-0.069	72	1029	116.5	6.84

IPI00345097.5	Uncharacterized protein	58.87%	1	3	100	267	-0.029
IPI00345552.5	Uncharacterized protein	73.60%	1	2	39	94	
IPI00348094.4	Uncharacterized protein	77.83%	1	2	43	470	0.131
IPI00353927.16	Uncharacterized protein	84.58%	3	11	79	406	
IPI00355250.6	Uncharacterized protein	46.05%	1	2	24	59	
IPI00377615.2	Uncharacterized protein	65.48%	2	4	160	499	-0.106
IPI00379625.2	Uncharacterized protein	57.29%	3	6	42	186	-0.077
IPI00380738.2	Uncharacterized protein	31.68%	3	2	56	123	0.161
IPI00381407.3	Uncharacterized protein	60.58%	20	2	35	77	
IPI00381495.5	Uncharacterized protein	81.88%	1	7	47	135	0.092
IPI00387489.2	Uncharacterized protein	58.08%	2	3	13	28	
IPI00396784.1	Uncharacterized protein	80.65%	2	1	48	145	
IPI00403564.3	Uncharacterized protein	82.35%	4	3	18	80	0.247
IPI00404662.2	Uncharacterized protein	93.66%	6	1	61	952	-0.114
IPI00405986.3	Uncharacterized protein	66.82%	1	1	90	241	-0.089
IPI00406030.2	Uncharacterized protein	80.83%	7	3	247	1057	0.040
IPI00406447.3	Uncharacterized protein	81.84%	5	22	72	1044	0.116
IPI00406762.5	Uncharacterized protein	76.21%	9	9	226	845	
IPI00406790.9	Uncharacterized protein	80.11%	13	2	64	607	0.045
IPI00417154.3	Uncharacterized protein	69.92%	7	6	139	557	-0.045
IPI00420329.1	Uncharacterized protein	82.02%	4	63	203	1117	-0.038
IPI00420950.3	Uncharacterized protein	96.82%	9	15	91	705	-0.010
IPI00421070.3	Uncharacterized protein	71.07%	40	1	68	184	0.171
IPI00453860.2	Uncharacterized protein	59.81%	1	8	84	243	0.111
IPI00458008.4	Uncharacterized protein	68.66%	1	2	14	43	0.804
IPI00458851.3	Uncharacterized protein	73.95%	3	1	26	643	0.140
IPI00459570.1	Uncharacterized protein	95.56%	3	0	60	875	0.144
IPI00459745.3	Uncharacterized protein	68.72%	4	5	56	192	
IPI00460042.4	Uncharacterized protein	63.27%	7	4	153	532	
IPI00460298.3	Uncharacterized protein	96.43%	5	3	16	88	0.004
IPI00460717.3	Uncharacterized protein	78.79%	1	2	179	621	-0.089
IPI00461407.1	Uncharacterized protein	96.71%	3	1	36	466	-0.119
IPI00463493.1	Uncharacterized protein	71.47%	2	21	146	565	0.371
IPI00463589.2	Uncharacterized protein	70.56%	3	3	118	639	0.069
IPI00464141.2	Uncharacterized protein	52.82%	3	6	62	388	-0.017
IPI00464280.2	Uncharacterized protein	44.01%	6	5	38	178	-0.183
IPI00464296.5	Uncharacterized protein	80.86%	4	1	110	808	-0.141
IPI00467006.2	Uncharacterized protein	47.49%	2	1	14	33	-0.007
IPI00467747.2	Uncharacterized protein	79.94%	3	8	20	100	
IPI00467841.6	Uncharacterized protein	99.49%	4	13	22	1538	0.236
IPI00468665.2	Uncharacterized protein	77.40%	6	1	218	760	
IPI00469618.2	Uncharacterized protein	53.27%	2	2	60	126	
IPI00471047.2	Uncharacterized protein	74.05%	2	1	37	437	0.143
IPI00474002.1	Uncharacterized protein	71.77%	6	1	26	192	
IPI00474193.2	Uncharacterized protein	30.86%	3	2	12	75	
IPI00474430.2	Uncharacterized protein	76.77%	3	5	35	285	0.006
IPI00474558.7	Uncharacterized protein	70.37%	3	9	82	428	0.017
IPI00480250.2	Uncharacterized protein	85.47%	5	1	90	328	-0.047
IPI00515199.1	Uncharacterized protein	46.44%	4	9	44	341	-0.031
IPI00515343.3	Uncharacterized protein	65.37%	1	1	24	67	
IPI00551435.7	Uncharacterized protein	59.01%	3	2	137	543	-0.158
IPI00553355.1	Uncharacterized protein	75.05%	3	1	36	113	
IPI00553379.3	Uncharacterized protein	44.72%	4	2	12	39	0.346
IPI00556699.1	Uncharacterized protein	75.29%	1	1	14	68	-0.062
IPI00606638.6	Uncharacterized protein	75.88%	2	6	406	1359	
IPI00620167.2	Uncharacterized protein	66.33%	1	13	48	203	-0.144
IPI00622850.2	Uncharacterized protein	82.15%	4	1	213	727	
IPI00623214.2	Uncharacterized protein	80.88%	2	8	33	165	0.010
IPI00623629.2	Uncharacterized protein	80.77%	13	2	42	155	
IPI00623834.1	Uncharacterized protein	58.86%	4	7	62	190	0.091
IPI00624138.3	Uncharacterized protein	73.15%	43	3	34	158	0.213
IPI00624663.3	Uncharacterized protein	55.54%	2	16	72	212	
IPI00624735.4	Uncharacterized protein	96.07%	9	4	29	238	-0.032
IPI00625263.1	Uncharacterized protein	73.73%	4	3	44	173	-0.065
IPI00625462.4	Uncharacterized protein	86.93%	1	1	80	342	
IPI00625848.2	Uncharacterized protein	70.04%	2	5	101	223	
IPI00625954.4	Uncharacterized protein	37.69%	1	5	17	54	
IPI00626132.3	Uncharacterized protein	98.15%	3	10	31	268	-0.221
**IPI00626366.3**	**Uncharacterized protein**	**83.28%**	**3**	**1**	**38**	**719**	0.169
IPI00626501.4	Uncharacterized protein	74.03%	5	2	149	378	
IPI00627008.2	Uncharacterized protein	44.51%	2	17	92	263	
IPI00648401.1	Uncharacterized protein	70.39%	2	6	64	194	
IPI00648408.1	Uncharacterized protein	66.97%	4	17	77	367	-0.083
IPI00648499.1	Uncharacterized protein	70.82%	6	2	89	265	-0.480
IPI00648567.1	Uncharacterized protein	76.10%	6	5	50	314	0.282
IPI00648589.1	Uncharacterized protein	70.09%	7	2	19	117	
IPI00648683.3	Uncharacterized protein	78.80%	7	1	101	1181	0.029
IPI00648685.1	Uncharacterized protein	53.14%	2	7	16	115	0.070
IPI00648743.1	Uncharacterized protein	77.32%	2	5	9	49	
IPI00648762.1	Uncharacterized protein	78.07%	11	2	44	333	
IPI00648810.1	Uncharacterized protein	73.32%	3	3	38	147	0.204
IPI00648915.1	Uncharacterized protein	62.80%	3	3	39	142	
IPI00648959.1	Uncharacterized protein	80.56%	5	4	73	306	-0.150
IPI00649019.1	Uncharacterized protein	61.54%	2	2	8	15	
IPI00649060.1	Uncharacterized protein	56.82%	9	7	78	243	
IPI00649424.2	Uncharacterized protein	61.07%	2	3	62	149	-0.199
IPI00649577.2	Uncharacterized protein	77.19%	20	2	64	189	
IPI00649682.1	Uncharacterized protein	45.53%	3	1	8	47	
IPI00649708.1	Uncharacterized protein	78.13%	3	2	135	508	
IPI00649778.1	Uncharacterized protein	79.88%	4	15	65	720	-0.139
IPI00649926.1	Uncharacterized protein	91.10%	4	3	14	58	
IPI00650029.1	Uncharacterized protein	47.51%	3	3	13	39	
IPI00652110.1	Uncharacterized protein	48.89%	5	4	80	350	0.149
IPI00652159.2	Uncharacterized protein	63.29%	6	1	33	91	
IPI00652845.3	Uncharacterized protein	98.85%	2	2	28	98	
IPI00653062.1	Uncharacterized protein	69.14%	2	2	23	75	
IPI00653599.1	Uncharacterized protein	52.14%	3	4	27	65	
IPI00653643.4	Uncharacterized protein	85.47%	7	27	55	1635	0.041
IPI00654008.2	Uncharacterized protein	78.97%	1	1	59	199	
IPI00654038.3	Uncharacterized protein	70.72%	1	1	48	107	
IPI00654377.4	Uncharacterized protein	80.98%	3	1	58	149	
IPI00662157.3	Uncharacterized protein	56.91%	4	11	63	203	
IPI00662244.2	Uncharacterized protein	73.50%	3	6	103	245	
IPI00663323.2	Uncharacterized protein	55.53%	4	2	60	136	
IPI00664246.4	Uncharacterized protein	68.69%	4	4	32	139	
IPI00664670.4	Uncharacterized protein	84.29%	2	84	259	1866	0.058
IPI00665996.4	Uncharacterized protein	84.30%	2	13	49	362	-0.029
IPI00673613.1	Uncharacterized protein	59.16%	6	2	139	429	
IPI00673669.2	Uncharacterized protein	81.85%	6	8	39	178	0.068
IPI00676717.3	Uncharacterized protein	65.96%	1	5	119	459	-0.083
IPI00677756.1	Uncharacterized protein	62.21%	2	7	49	120	-0.240
IPI00678265.2	Uncharacterized protein	88.86%	2	1	43	186	-0.331
IPI00678657.3	Uncharacterized protein	38.35%	1	1	16	152	-0.189
IPI00719927.2	Uncharacterized protein	70.19%	2	1	62	259	
IPI00749697.1	Uncharacterized protein	68.83%	3	7	35	213	-0.007
IPI00749713.3	Uncharacterized protein	80.00%	3	2	19	731	0.380
IPI00749991.7	Uncharacterized protein	99.05%	7	5	59	1497	0.033
IPI00750473.1	Uncharacterized protein	70.16%	2	3	45	107	
IPI00751223.1	Uncharacterized protein	82.81%	2	1	29	263	-0.193
IPI00751303.2	Uncharacterized protein	74.87%	8	1	21	192	0.255
IPI00751834.1	Uncharacterized protein	63.71%	8	5	78	233	
IPI00752080.1	Uncharacterized protein	70.83%	4	15	85	423	-0.283
IPI00752419.5	Uncharacterized protein	95.04%	2	1	221	1548	0.066
IPI00753072.3	Uncharacterized protein	72.02%	1	1	15	52	-0.201
IPI00754929.3	Uncharacterized protein	75.33%	1	3	60	145	
IPI00754992.1	Uncharacterized protein	92.85%	2	20	57	744	-0.017
IPI00755241.1	Uncharacterized protein	80.60%	4	28	101	562	-0.065
IPI00755843.1	Uncharacterized protein	84.65%	7	4	30	619	-0.295
IPI00755905.1	Uncharacterized protein	51.62%	3	7	117	392	-0.223
IPI00755907.1	Uncharacterized protein	59.29%	2	7	85	218	
IPI00756485.3	Uncharacterized protein	85.15%	4	6	46	167	-0.007
IPI00757097.2	Uncharacterized protein	56.55%	3	4	119	297	
IPI00757312.1	Uncharacterized protein	86.04%	10	63	273	2172	0.152
IPI00758024.1	Uncharacterized protein	92.86%	4	2	26	566	-0.396
IPI00758248.3	Uncharacterized protein	56.18%	3	2	108	246	
IPI00761402.2	Uncharacterized protein	65.80%	2	1	38	133	
IPI00761442.2	Uncharacterized protein	100.00%	1	1	15	118	0.048
IPI00761607.2	Uncharacterized protein	77.63%	1	1	7	43	0.219
IPI00761751.2	Uncharacterized protein	72.76%	1	2	184	593	0.167
IPI00762808.4	Uncharacterized protein	81.01%	2	2	21	76	-0.095
IPI00775790.1	Uncharacterized protein	83.82%	5	1	34	135	-0.238
IPI00775791.1	Uncharacterized protein	79.89%	7	9	22	143	0.244
IPI00775798.1	Uncharacterized protein	74.63%	8	15	47	378	0.149
IPI00775849.2	Uncharacterized protein	95.17%	2	1	70	1164	0.318
IPI00775863.1	Uncharacterized protein	84.82%	7	8	38	446	0.120
IPI00776023.1	Uncharacterized protein	96.87%	3	27	73	525	-0.184
IPI00776055.1	Uncharacterized protein	92.41%	3	10	93	440	-0.054
IPI00776104.1	Uncharacterized protein	69.56%	2	11	101	488	0.084
IPI00776145.1	Uncharacterized protein	76.70%	5	18	136	439	0.505
IPI00776164.1	Uncharacterized protein	67.50%	3	5	33	201	-0.085
IPI00776310.2	Uncharacterized protein	52.78%	2	8	58	184	0.099
IPI00776324.1	Uncharacterized protein	63.77%	4	6	55	155	
IPI00808277.2	Uncharacterized protein	64.89%	2	2	52	133	
IPI00828266.2	Uncharacterized protein	96.10%	1	1	30	517	-0.053
IPI00830152.1	Uncharacterized protein	65.08%	8	2	37	99	
IPI00830159.1	Uncharacterized protein	93.09%	3	10	30	581	0.024
IPI00830222.1	Uncharacterized protein	53.61%	2	3	16	31	
IPI00830254.1	Uncharacterized protein	89.95%	2	2	62	321	0.352
IPI00830282.1	Uncharacterized protein	58.73%	2	1	5	25	-0.042
IPI00830393.1	Uncharacterized protein	58.92%	3	6	19	80	-0.335
IPI00830443.1	Uncharacterized protein	82.18%	2	3	83	372	-0.146
IPI00830625.1	Uncharacterized protein	65.67%	7	1	19	55	
IPI00830703.1	Uncharacterized protein	35.00%	1	1	3	19	
IPI00830719.1	Uncharacterized protein	72.27%	11	1	76	213	
IPI00830734.1	Uncharacterized protein	93.78%	4	13	38	262	-0.120
IPI00830807.1	Uncharacterized protein	72.62%	5	10	45	214	
IPI00830857.1	Uncharacterized protein	76.26%	3	1	41	92	0.361
IPI00830910.1	Uncharacterized protein	100.00%	1	1	14	692	-0.106
IPI00830923.1	Uncharacterized protein	68.21%	5	21	100	402	0.040
IPI00830957.1	Uncharacterized protein	77.22%	6	7	26	98	
IPI00830989.2	Uncharacterized protein	82.79%	13	9	46	160	
IPI00831080.1	Uncharacterized protein	62.43%	6	4	42	108	
IPI00831082.1	Uncharacterized protein	89.05%	2	1	31	606	-0.048
IPI00831180.1	Uncharacterized protein	75.86%	4	32	133	932	
IPI00831305.1	Uncharacterized protein	35.57%	2	8	17	175	
IPI00831479.1	Uncharacterized protein	54.10%	8	3	32	72	
IPI00831499.1	Uncharacterized protein	61.38%	11	1	57	110	
IPI00831604.1	Uncharacterized protein	91.09%	4	6	15	244	0.024
IPI00848478.1	Uncharacterized protein	69.07%	3	25	136	712	0.001
IPI00849953.1	Uncharacterized protein	72.93%	1	4	415	1148	
IPI00850698.3	Uncharacterized protein	47.19%	2	2	42	73	
IPI00850843.2	Uncharacterized protein	79.07%	2	4	84	455	0.070
IPI00853924.1	Uncharacterized protein	94.06%	4	2	52	1269	0.069
IPI00853964.2	Uncharacterized protein	93.12%	5	1	21	305	-0.017
IPI00856140.1	Uncharacterized protein	79.49%	2	1	12	250	-0.007
IPI00856350.1	Uncharacterized protein	90.95%	5	1	198	787	
IPI00856352.1	Uncharacterized protein	94.92%	3	5	38	548	0.188
IPI00856475.1	Uncharacterized protein	58.41%	12	11	118	347	
IPI00856488.1	Uncharacterized protein	53.24%	10	13	49	362	-0.028
IPI00856823.1	Uncharacterized protein	86.80%	1	1	22	91	-0.178
IPI00856902.1	Uncharacterized protein	75.70%	1	1	17	202	-0.047
IPI00857073.1	Uncharacterized protein	49.09%	4	2	15	52	
IPI00857151.1	Uncharacterized protein	78.36%	3	6	59	144	-0.413
IPI00857226.1	Uncharacterized protein	60.94%	3	4	39	135	
IPI00857239.1	Uncharacterized protein	60.00%	3	3	4	43	
IPI00857329.1	Uncharacterized protein	57.59%	6	6	23	164	
IPI00857598.1	Uncharacterized protein	43.48%	7	3	24	112	
IPI00857685.1	Uncharacterized protein	85.83%	8	18	201	769	0.138
IPI00857771.1	Uncharacterized protein	78.88%	9	37	178	1041	-0.045
IPI00858255.2	Uncharacterized protein	62.19%	6	7	25	122	
IPI00874408.1	Uncharacterized protein	67.60%	4	24	65	457	0.011
IPI00874542.1	Uncharacterized protein	68.66%	3	4	72	212	
IPI00874566.3	Uncharacterized protein	95.77%	11	18	89	421	0.070
IPI00874587.2	Uncharacterized protein	81.62%	6	5	253	1091	-0.125
IPI00874710.1	Uncharacterized protein	96.49%	1	2	92	669	-0.106
**IPI00875068.1**	**Uncharacterized protein**	**61.05%**	**3**	**11**	**26**	**181**	0.269
IPI00875279.1	Uncharacterized protein	98.98%	3	2	54	1438	-0.028
IPI00875340.1	Uncharacterized protein	62.93%	4	9	35	288	-0.388
IPI00875419.1	Uncharacterized protein	78.88%	8	25	55	503	
IPI00875420.1	Uncharacterized protein	79.73%	3	1	55	881	0.128
IPI00875583.1	Uncharacterized protein	69.45%	2	5	45	103	
IPI00875652.1	Uncharacterized protein	71.93%	3	1	19	112	
IPI00875717.2	Uncharacterized protein	82.64%	6	16	57	486	-0.189
IPI00875815.1	Uncharacterized protein	66.91%	3	1	118	371	
IPI00876206.1	Uncharacterized protein	65.51%	1	1	44	78	
IPI00876248.2	Uncharacterized protein	61.59%	9	1	69	259	
IPI00876257.2	Uncharacterized protein	40.43%	5	1	23	222	0.065
IPI00877229.2	Uncharacterized protein	79.53%	1	1	44	279	0.048
IPI00880373.3	Uncharacterized protein	86.73%	4	4	73	178	
IPI00880407.1	Uncharacterized protein	89.01%	4	3	12	200	
IPI00880426.1	Uncharacterized protein	69.82%	18	6	111	291	
IPI00880432.1	Uncharacterized protein	72.22%	4	2	19	40	
IPI00880644.3	Uncharacterized protein	79.85%	6	75	331	2239	0.043
IPI00880741.1	Uncharacterized protein	82.30%	9	2	14	37	
IPI00880977.1	Uncharacterized protein	95.54%	1	1	32	442	-0.120
IPI00881058.1	Uncharacterized protein	60.20%	1	1	11	78	
IPI00881361.1	Uncharacterized protein	33.33%	2	1	15	78	
IPI00881499.1	Uncharacterized protein	70.13%	3	10	65	521	-0.106
IPI00881858.1	Uncharacterized protein	63.14%	6	11	61	285	
IPI00881945.1	Uncharacterized protein	95.56%	5	0	57	857	0.144
IPI00882020.1	Uncharacterized protein	66.17%	4	5	25	61	-0.079
IPI00885254.1	Uncharacterized protein	73.60%	1	1	22	66	
IPI00885322.1	Uncharacterized protein	78.38%	4	1	174	842	0.029
IPI00885369.1	Uncharacterized protein	100.00%	1	2	6	346	
IPI00885385.1	Uncharacterized protein	90.23%	4	1	35	140	
IPI00885404.1	Uncharacterized protein	69.42%	1	1	52	194	-0.144
IPI00885524.1	Uncharacterized protein	87.69%	3	3	27	98	
IPI00885527.1	Uncharacterized protein	74.89%	3	1	25	152	
IPI00885811.1	Uncharacterized protein	91.52%	2	2	20	126	0.006
IPI00885883.1	Uncharacterized protein	59.04%	4	3	14	53	-0.013
IPI00886029.1	Uncharacterized protein	86.90%	2	1	28	142	0.088
IPI00889281.1	Uncharacterized protein	88.39%	3	3	31	117	0.013
IPI00890291.1	Uncharacterized protein	94.64%	5	2	80	395	
IPI00894613.1	Uncharacterized protein	40.65%	4	3	4	8	
IPI00894660.1	Uncharacterized protein	35.33%	1	1	13	30	0.156
IPI00894741.1	Uncharacterized protein	64.42%	3	1	86	383	0.038
IPI00894766.1	Uncharacterized protein	82.11%	6	5	20	47	-0.149
IPI00894832.4	Uncharacterized protein	67.90%	2	3	35	114	
IPI00895208.2	Uncharacterized protein	60.99%	7	5	77	187	
IPI00895370.1	Uncharacterized protein	47.52%	3	3	41	119	
IPI00896614.1	Uncharacterized protein	59.86%	4	1	52	166	-0.252
IPI00900442.1	Uncharacterized protein	83.28%	8	6	273	1477	0.223
IPI00918041.1	Uncharacterized protein	95.06%	1	1	23	346	-0.095

IPI00918064.1	Uncharacterized protein	81.93%	7	11	241	939	0.161	0.073	0.016	-0.057	-0.039	10	-0.103	0.044	-0.238	-0.278	0.104	8																			2031	226.7	7.94
IPI00918148.1	Uncharacterized protein	49.12%	6	1	90	264																									-0.085	0.040	0.148	0.033	0.050	4	1710	184.8	5.90
IPI00918301.1	Uncharacterized protein	72.66%	4	6	12	158	0.069	0.145	0.130	0.165	0.010	6	0.104	0.073	0.108	-0.044	-0.059	8	0.119	0.107	-0.034	0.104	-0.028	6	-0.142	-0.044	-0.231	-0.060	-0.288	5	-0.051	-0.160	0.058	-0.050	-0.012	26	128	14.4	4.77
IPI00918305.1	Uncharacterized protein	82.95%	1	2	24	272	0.091	0.017	0.045	0.020	0.110	57	0.801	0.031	0.676	0.104	0.656	25													-0.299	-0.136	-0.155	-0.333	-0.311	8	1126	115.2	5.59
IPI00918347.1	Uncharacterized protein	56.48%	9	11	40	140																									-0.057	-0.283	-0.228	-0.193	-0.231	38	687	75.5	6.14
IPI00922955.1	Uncharacterized protein	85.76%	5	1	140	645	-0.149	0.050	-0.157	-0.004	-0.173	71	0.115	0.092	0.104	0.066	0.127	20	0.296	0.112	0.346	0.017	0.161	24													1159	130.6	6.60
IPI00922961.1	Uncharacterized protein	74.95%	2	2	51	179							-0.669	-0.250	-0.335	-0.175	-0.498	3																			531	57.4	9.33
IPI00922963.1	Uncharacterized protein	72.65%	7	8	113	501	-0.100	0.080	-0.095	-0.028	0.085	3							-0.141	-0.167	-0.331	-0.109	0.132	7													1221	139.0	7.37
IPI00923056.1	Uncharacterized protein	84.25%	8	16	42	242																			-0.379	-0.370	-0.407	-0.071	-0.218	5	0.099	0.062	0.100	0.151	0.052	54	381	44.1	6.80
IPI00927957.1	Uncharacterized protein	96.04%	1	1	23	358	0.280	-0.048	0.341	0.058	0.281	23	-0.009	-0.173	-0.139	-0.154	0.151	9	0.262	0.035	0.202	0.143	0.080	15	-0.660	0.143	-0.908	0.036	-0.749	20	0.089	-0.026	-0.039	-0.016	-0.050	29	278	29.9	6.67
IPI00927975.1	Uncharacterized protein	89.80%	5	23	97	756	-0.184	0.000	-0.111	-0.127	-0.013	13	0.724	-0.065	0.723	-0.010	0.594	29	-0.099	-0.121	-0.095	0.130	-0.026	37	-0.279	0.017	-0.396	-0.023	-0.360	43	-0.010	-0.189	-0.165	-0.194	-0.150	80	784	85.5	6.89
IPI00927986.1	Uncharacterized protein	70.91%	4	8	111	383																															1186	132.9	8.59
IPI00928007.1	Uncharacterized protein	82.88%	11	38	243	1088	0.123	-0.014	0.030	-0.201	-0.083	6	-0.236	0.204	0.286	-0.057	0.068	9							-0.309	-0.091	-0.304	-0.076	-0.272	8	-0.020	-0.100	-0.045	-0.038	-0.076	114	1828	212.2	8.62
IPI00928150.1	Uncharacterized protein	78.27%	11	13	36	120																									-0.076	0.107	0.011	0.014	-0.037	29	382	42.7	8.98
IPI00928301.1	Uncharacterized protein	65.43%	2	1	12	241	-0.163	-0.025	-0.127	-0.221	-0.146	4	-0.606	0.030	-0.162	-0.071	-0.063	15							-0.246	-0.023	-0.108	0.065	0.123	14	-0.012	0.197	0.075	0.282	-0.083	3	188	20.1	5.69
IPI00956775.1	Uncharacterized protein	78.05%	13	10	157	564							0.307	-0.041	0.261	-0.056	0.087	20																			1449	158.6	9.11
IPI00956893.1	Uncharacterized protein	79.28%	6	4	151	643	0.156	-0.007	0.365	-0.109	0.176	5																									1226	139.8	6.04
IPI00969847.1	Uncharacterized protein	95.07%	2	1	13	120	0.073	-0.060	0.217	0.184	-0.048	6	-0.007	0.009	-0.026	-0.003	-0.095	7							-0.257	0.104	-0.196	0.041	-0.229	14	0.116	-0.026	-0.001	-0.082	-0.020	5	142	16.0	5.27
IPI00969919.1	Uncharacterized protein	61.00%	3	7	32	173	0.145	0.198	0.212	0.010	0.106	5	0.486	0.013	0.520	0.261	0.465	4	0.372	0.109	0.539	0.175	0.500	4	0.000	-0.173	0.019	-0.083	-0.031	10							300	33.2	8.79
IPI00970519.1	Uncharacterized protein	56.56%	2	1	17	29																									-0.123	0.091	0.045	0.081	0.157	3	244	26.8	5.44
IPI00970589.1	Uncharacterized protein	9.36%	2	1	4	17	0.467	0.138	0.365	-0.031	0.328	5																									235	26.2	8.34
IPI00970622.1	Uncharacterized protein	100.00%	2	1	11	67	0.208	-0.001	0.106	0.020	0.034	5													-0.010	-0.223	-0.338	-0.079	-0.053	3							71	8.1	4.61
IPI00971213.2	Uncharacterized protein	58.72%	5	3	19	30							-0.783	0.300	-0.338	-0.366	-0.844	4																			298	33.7	8.60
IPI00973613.1	Uncharacterized protein	78.51%	2	3	319	922	-0.045	0.009	-0.094	-0.019	-0.114	3	0.242	0.222	0.203	0.312	-0.019	5																			2611	301.6	6.57
IPI00974614.1	Uncharacterized protein	25.83%	7	1	26	218	-0.026	-0.051	0.084	-0.141	-0.039	5	-0.523	-0.105	-0.423	-0.056	-0.517	4	0.062	-0.392	-0.199	-0.034	-0.001	6							0.384	0.169	0.167	0.096	0.080	7	995	102.0	5.77
IPI00974743.1	Uncharacterized protein	93.21%	4	3	22	61																									0.230	0.179	-0.026	0.433	-0.138	3	221	25.3	6.35
IPI00974777.1	Uncharacterized protein	81.17%	5	11	157	539	0.010	-0.009	-0.076	-0.085	-0.047	11	0.223	0.030	-0.031	-0.117	0.183	3																			1184	135.7	5.86
IPI00974830.1	Uncharacterized protein	56.38%	1	7	51	124																									-0.163	-0.094	-0.257	-0.181	0.038	13	752	80.2	4.77
IPI00974963.1	Uncharacterized protein	60.25%	4	17	133	1000	0.104	0.000	-0.001	-0.083	0.038	17	-0.105	-0.056	0.055	-0.108	-0.152	15	-0.386	-0.081	-0.349	0.010	-0.274	7	-0.003	-0.184	-0.209	0.076	0.013	11							2287	242.5	6.71
IPI00975117.1	Uncharacterized protein	75.06%	8	19	148	391																									0.029	0.062	0.075	0.153	0.102	25	1672	186.6	6.30
IPI00975173.1	Uncharacterized protein	64.75%	4	2	35	64							-0.664	0.136	-0.349	-0.120	-0.313	4																			451	52.4	6.30
IPI00975225.1	Uncharacterized protein	57.24%	12	7	45	101																									-0.450	0.172	-0.234	-0.042	0.052	9	615	71.2	5.67
IPI00985532.1	Uncharacterized protein	79.79%	2	2	198	836	0.290	0.139	0.332	-0.050	0.469	6																									1865	205.6	7.47
IPI00985874.1	Uncharacterized protein	80.03%	16	6	97	460	-0.053	-0.165	-0.498	-0.233	-0.498	3	-0.181	0.014	-0.446	0.031	-0.360	7							0.019	-0.157	0.096	0.091	-0.288	3	-0.072	-0.039	-0.111	-0.063	-0.112	5	776	86.5	9.72
IPI00986036.1	Uncharacterized protein	82.03%	1	1	41	239							-0.306	-0.155	-0.304	0.016	-0.042	3							-0.278	-0.025	-0.347	-0.026	-0.183	48							462	51.6	8.95
IPI00986204.1	Uncharacterized protein	93.94%	3	1	177	1366	0.214	0.050	0.180	0.017	0.220	59	0.079	0.013	0.091	0.111	-0.047	62	0.069	-0.028	0.057	-0.142	-0.047	17	0.184	-0.120	0.279	-0.059	0.119	13							1090	121.9	8.34
IPI00986206.1	Uncharacterized protein	93.14%	2	8	17	304	-0.160	0.020	-0.299	-0.045	-0.255	18	0.282	0.000	0.233	-0.047	0.449	20	0.312	0.086	0.220	-0.188	0.062	11	-0.521	-0.411	-0.448	0.016	-0.317	3	-0.001	0.038	-0.144	-0.051	0.095	13	175	19.7	4.84
IPI00986276.1	Uncharacterized protein	94.56%	1	3	16	314	0.279	-0.016	0.222	0.036	0.333	35	-0.037	0.088	0.059	0.083	0.004	19	-0.062	-0.064	-0.085	0.054	-0.119	32	-0.029	0.072	-0.193	0.084	-0.119	20	-0.017	0.115	0.041	0.021	-0.016	35	147	16.5	6.55
IPI00986301.1	Uncharacterized protein	86.59%	2	1	25	213	-0.204	-0.045	-0.189	-0.050	-0.149	12	0.432	0.189	0.668	0.167	0.449	11							0.050	0.047	-0.042	0.043	-0.041	15	-0.175	-0.079	0.048	-0.183	-0.048	12	179	20.9	8.51
IPI00986360.1	Uncharacterized protein	92.52%	2	1	12	805	0.052	0.098	0.089	0.098	0.033	15	0.061	0.160	0.075	0.290	-0.105	9	0.123	-0.006	0.194	-0.041	0.175	9	-0.014	0.004	0.145	-0.014	0.123	28							107	11.5	9.39
IPI00986443.1	Uncharacterized protein	83.26%	4	1	22	121																									-0.003	0.167	0.127	0.265	0.202	38	215	25.5	6.23
IPI00986866.1	Uncharacterized protein	65.79%	5	7	43	104	-0.260	-0.035	-0.317	-0.019	0.055	4																			0.156	0.135	0.165	-0.066	-0.045	8	570	63.6	7.27
IPI00986992.1	Uncharacterized protein	56.74%	5	1	51	368	0.295	0.184	0.476	0.148	0.302	6							0.193	0.026	0.122	0.055	0.054	4	-0.007	0.152	-0.196	0.178	-0.125	7	0.124	-0.097	-0.144	-0.147	-0.127	99	779	89.4	6.86
IPI00987055.1	Uncharacterized protein	72.61%	4	10	113	443	-0.133	0.016	-0.019	-0.007	-0.152	12	0.128	-0.285	-0.400	0.307	0.094	5							-0.276	0.167	-0.311	0.238	-0.324	5							1285	142.2	6.00
IPI00987231.1	Uncharacterized protein	76.89%	3	1	33	145																			0.280	0.251	-0.004	0.202	-0.041	4							450	50.8	6.07
IPI00987239.1	Uncharacterized protein	56.91%	2	8	68	320	-0.214	0.085	-0.311	0.075	-0.423	6	-0.292	-0.068	0.152	-0.051	-0.186	4	0.577	-0.156	-0.411	-0.673	-0.144	5	0.361	0.024	0.099	-0.050	0.166	4							847	96.1	6.55
IPI00987412.1	Uncharacterized protein	78.31%	1	2	26	553	0.300	-0.131	0.295	-0.026	0.430	49	-0.470	0.010	-0.360	-0.189	-0.253	45	-0.229	-0.173	-0.178	0.134	-0.056	58	-0.100	0.037	-0.154	0.029	-0.131	30	0.034	-0.004	0.020	-0.299	0.017	53	166	18.5	7.25
IPI00987453.2	Uncharacterized protein	69.44%	4	1	21	49																									0.387	0.079	0.052	-0.216	0.468	5	373	42.1	5.05
IPI00987510.1	Uncharacterized protein	76.24%	13	23	253	949	-0.001	0.026	0.161	0.003	0.077	33							-0.969	-0.311	-0.390	-0.476	-0.448	4	-0.194	-0.088	0.047	-0.029	-0.025	3	-0.074	-0.285	-0.299	-0.114	-0.150	8	2083	235.7	6.07
IPI00987702.1	Uncharacterized protein	91.71%	1	1	53	562	-0.114	0.026	-0.072	0.011	-0.048	40	0.157	0.075	0.087	-0.003	0.135	32	-0.007	-0.052	-0.074	-0.035	-0.088	17	-0.022	-0.009	-0.042	-0.016	0.068	42	0.081	0.038	0.013	0.081	0.073	10	615	66.8	5.50
IPI00987951.1	Uncharacterized protein	88.45%	7	11	119	565	-0.117	0.011	-0.201	-0.065	-0.569	17	0.492	-0.017	0.323	0.080	0.448	15	0.358	0.177	0.209	0.033	0.057	22	0.069	-0.037	0.024	-0.038	0.087	18	-0.007	-0.062	-0.186	-0.044	0.484	68	978	107.7	7.77
IPI00988302.1	Uncharacterized protein	74.40%	3	1	43	259							-0.306	-0.155	-0.304	0.016	-0.042	3							-0.250	-0.006	-0.344	-0.026	-0.183	50							461	51.4	8.76
IPI00988656.1	Uncharacterized protein	69.41%	1	1	10	150	0.044	0.061	-0.094	0.043	-0.092	7	0.188	0.000	0.088	-0.193	0.059	7	-0.285	-0.184	-0.146	0.036	-0.007	6	-0.228	-0.311	-0.271	-0.111	0.148	9							85	9.4	5.91
IPI00988842.1	Uncharacterized protein	66.58%	2	12	35	549	-0.180	0.010	-0.276	0.037	-0.255	23	0.467	0.054	0.545	0.138	0.581	13	0.188	0.069	0.366	0.170	-0.012	5	-0.019	-0.092	0.058	-0.193	0.072	23	0.016	-0.131	-0.038	-0.198	-0.100	28	389	42.3	9.01
IPI00989075.1	Uncharacterized protein	85.14%	3	7	13	88	0.343	0.282	0.273	0.088	0.184	4																			-0.029	-0.116	-0.253	-0.131	-0.019	10	74	8.8	9.31
IPI00989102.1	Uncharacterized protein	51.56%	2	2	59	122	0.308	0.072	0.215	0.079	0.274	3																									1445	162.9	6.01
IPI00989327.1	Uncharacterized protein	65.24%	4	2	14	38	-0.302	0.149	-0.063	0.061	-0.297	4													-0.193	-0.168	-0.150	-0.206	-0.290	3							164	19.5	9.94
IPI00989391.1	Uncharacterized protein	67.19%	1	1	27	418	0.065	0.131	0.073	0.011	0.070	11	-0.158	-0.109	-0.286	-0.111	-0.123	6	0.132	0.054	0.144	-0.004	-0.125	14	-0.147	0.026	-0.139	0.031	-0.139	9	0.073	-0.009	-0.042	-0.047	-0.019	59	317	34.2	5.97
IPI00990411.1	Uncharacterized protein	60.81%	1	1	24	67																									0.188	-0.031	-0.116	0.038	-0.016	8	444	49.9	6.21
IPI00990657.1	Uncharacterized protein	97.57%	1	1	260	3017	0.051	0.052	0.027	0.004	0.000	254	0.614	0.030	0.498	0.041	0.455	162	-0.032	0.098	-0.072	-0.026	-0.189	397	0.068	-0.065	0.051	-0.123	-0.057	14	-0.083	-0.083	-0.066	-0.136	-0.077	65	1646	186.4	6.16
IPI01007925.1	Uncharacterized protein	59.49%	5	36	227	1247	-0.022	0.052	0.016	0.135	-0.066	43	-0.085	-0.068	-0.080	-0.114	-0.128	62	-0.108	-0.098	0.016	0.000	0.037	35	0.047	-0.006	0.106	-0.007	0.007	18							2846	310.7	9.10
IPI01007958.1	Uncharacterized protein	40.29%	11	12	137	474																									0.038	0.157	0.106	0.055	0.050	36	3353	366.3	4.64
IPI01008123.1	Uncharacterized protein	61.63%	6	9	40	212	0.131	0.161	0.183	0.099	0.057	4	-0.069	0.181	0.064	0.433	-0.279	8	0.112	0.076	0.441	0.001	-0.063	10	0.210	0.153	0.174	0.115	0.118	9							443	49.9	9.04
IPI01008181.1	Uncharacterized protein	43.91%	3	3	113	262							0.651	0.332	0.566	0.290	0.335	3																			2546	291.8	7.55
IPI01008362.3	Uncharacterized protein	70.71%	8	11	184	689	-0.474	-0.178	-0.213	0.007	-0.344	3	-0.142	-0.381	-0.246	-0.032	-0.065	3	-0.404	-0.206	-0.038	-0.094	-0.100	4	0.000	-0.035	0.068	-0.194	-0.154	10							1960	218.0	6.89
IPI01008564.1	Uncharacterized protein	69.85%	5	3	49	132							0.379	-0.086	0.497	0.198	0.383	4																			461	49.4	5.02
IPI01008598.1	Uncharacterized protein	23.54%	4	2	20	44							-0.229	-0.009	-0.176	0.001	-0.194	3													0.384	0.306	0.245	0.088	0.115	3	769	78.1	4.89
IPI01008670.1	Uncharacterized protein	53.02%	3	3	24	199	0.075	-0.117	-0.025	-0.063	0.058	11	-0.152	-0.004	0.158	0.158	-0.133	18	-0.260	-0.144	-0.167	0.147	-0.007	14	0.440	0.099	0.482	-0.010	0.407	11	-0.080	-0.042	0.058	-0.014	0.057	4	315	35.8	4.16
**IPI01016197.1**	**Uncharacterized protein**	**78.36%**	**4**	**3**	**15**	**71**																									**-0.206**	**0.104**	**0.189**	**0.257**	**0.238**	**21**	**171**	**18.7**	**9.22**
IPI01023254.1	Uncharacterized protein	30.18%	6	1	26	71																									0.061	0.256	-0.025	0.415	-0.258	5	845	93.4	6.28
IPI01023311.1	Uncharacterized protein	54.76%	6	2	40	80							0.197	-0.155	0.140	-0.122	-0.020	4																			820	94.1	7.55
IPI01026773.1	Uncharacterized protein	38.79%	5	3	46	136																									0.240	-0.099	-0.175	-0.060	-0.060	4	763	83.0	6.61
IPI01027105.1	Uncharacterized protein	73.84%	5	7	25	108							-0.196	-0.041	-0.094	0.094	-0.163	3	-0.694	0.076	-0.017	-0.017	-0.158	4							0.134	0.054	0.346	-0.102	-0.042	12	302	34.1	7.96
IPI01027122.1	Uncharacterized protein	62.17%	7	4	56	175																									-0.302	0.013	0.170	0.106	-0.474	3	801	90.1	7.93
IPI01027807.1	Uncharacterized protein	65.64%	3	29	80	603	0.267	0.000	0.283	0.036	0.324	39	0.139	0.066	0.161	0.023	-0.082	38	0.601	0.044	0.428	-0.252	0.447	10	-0.119	0.021	-0.120	0.038	-0.088	28	-0.082	0.066	0.037	0.029	0.069	32	745	84.9	4.89
IPI00271430.4	Uncharacterized protein (Fragment)	74.80%	2	2	13	81																			0.068	-0.155	0.057	0.006	-0.060	5							123	14.1	8.46
IPI00458337.5	Uncharacterized protein (Fragment)	84.30%	2	1	32	325	0.094	0.061	0.132	0.077	-0.003	20	0.175	0.140	0.419	0.229	0.094	17	0.134	0.119	0.085	0.123	0.070	30	0.098	0.013	0.091	0.017	0.099	22	-0.001	0.189	-0.035	0.122	0.131	11	223	26.2	11.02
IPI00464348.2	Uncharacterized protein (Fragment)	85.97%	1	1	37	578	0.055	0.026	0.062	0.013	0.024	32	-0.105	-0.009	0.043	0.019	-0.026	31	0.388	0.112	0.218	-0.032	0.141	31	0.102	0.004	0.128	-0.037	0.162	30							278	30.6	9.95
IPI00650014.4	Uncharacterized protein (Fragment)	50.72%	3	1	46	150	-0.031	-0.116	-0.175	-0.586	-0.019	4							-0.199	-0.037	-0.012	0.029	0.084	3													759	86.8	4.94
IPI00652424.2	Uncharacterized protein (Fragment)	48.03%	2	4	24	57																									0.114	-0.221	-0.224	-0.201	-0.234	8	356	40.2	9.09
IPI00874991.1	Uncharacterized protein (Fragment)	69.21%	2	14	135	493	-0.023	-0.029	0.017	0.001	0.013	17	-0.044	-0.138	-0.119	0.156	-0.206	15	0.003	0.011	0.079	-0.318	0.026	14													1468	163.2	6.76
IPI00986111.1	Uncharacterized protein (Fragment)	68.56%	9	9	109	403																			-0.248	-0.020	-0.155	-0.062	0.057	3	-0.112	-0.050	-0.111	-0.180	-0.234	9	1339	150.7	8.21
IPI00986163.1	Uncharacterized protein (Fragment)	78.86%	3	28	174	744	-0.051	0.110	-0.106	0.065	-0.144	18	0.213	0.233	0.132	0.289	0.140	33	0.411	0.039	0.026	-0.141	0.183	12	0.198	-0.079	0.100	-0.035	0.234	17							2067	233.7	6.60
IPI00986693.1	Uncharacterized protein (Fragment)	62.27%	3	7	35	148	0.203	-0.053	0.132	-0.136	0.153	6																			-0.001	0.140	0.006	0.026	-0.017	10	591	64.6	6.54
IPI00988866.1	Uncharacterized protein (Fragment)	78.55%	3	7	56	264							0.044	-0.198	0.110	0.130	-0.257	8													-0.080	-0.037	-0.116	-0.095	-0.069	17	676	74.4	8.50
IPI00989044.1	Uncharacterized protein (Fragment)	79.91%	7	24	81	384	-0.063	0.085	-0.086	0.162	0.103	6	-0.176	-0.357	-0.262	-0.245	-0.219	16	0.019	0.101	-0.127	-0.413	-0.022	4	-0.097	0.038	0.162	-0.080	0.199	7	0.076	0.052	0.103	0.110	0.087	20	906	99.1	5.02
IPI00989517.1	Uncharacterized protein (Fragment)	94.76%	6	1	83	852	0.055	0.034	0.102	0.001	0.014	22	0.209	0.076	0.276	0.395	-0.025	15	0.052	0.132	-0.053	0.075	-0.181	28	-0.699	-0.108	-0.808	-0.045	-0.727	33	0.136	0.068	0.020	-0.039	0.043	149	839	92.3	4.88
IPI00989544.2	Uncharacterized protein (Fragment)	68.36%	4	12	20	391	0.013	-0.025	0.059	0.009	0.087	68	-0.450	-0.042	-0.427	-0.128	-0.311	22	0.249	0.034	0.156	-0.007	0.087	60													177	19.2	9.31
IPI00989713.1	Uncharacterized protein (Fragment)	80.99%	3	3	43	239	-0.103	-0.100	0.041	0.111	-0.044	11	0.152	0.004	0.068	-0.042	0.000	5	0.156	-0.067	0.099	0.061	0.233	5							-0.415	-0.404	-0.257	-0.083	-0.044	6	426	49.7	6.84
IPI00990372.1	Uncharacterized protein (Fragment)	62.68%	4	4	12	58							-1.155	-0.006	-0.808	-0.340	-0.278	3																			209	22.4	6.79
IPI00990529.1	Uncharacterized protein (Fragment)	98.56%	5	13	20	603	-0.138	0.059	0.122	-0.053	0.051	41	-0.213	-0.069	-0.112	-0.123	0.094	24	-0.388	-0.151	-0.394	0.073	-0.306	30	-0.396	0.080	-0.371	0.083	-0.271	52	-0.045	0.037	-0.047	-0.051	-0.001	41	209	23.5	7.87
IPI01008188.1	Uncharacterized protein (Fragment)	77.70%	4	1	76	265	-0.262	0.068	-0.243	-0.281	-0.219	9	-0.053	-0.035	-0.032	-0.138	0.085	13							-0.127	0.001	-0.167	-0.127	-0.063	6							906	103.3	5.15
IPI01008343.1	Uncharacterized protein (Fragment)	47.47%	2	1	7	15							-0.431	0.124	-0.193	0.104	0.194	3																			257	28.5	5.01
IPI01023194.1	Uncharacterized protein (Fragment)	70.22%	16	8	104	309													0.582	0.044	-0.009	0.193	0.061	3							0.033	0.302	0.054	0.016	-0.136	9	1286	142.6	8.37
IPI01023300.1	Uncharacterized protein (Fragment)	76.95%	12	18	76	226																									0.061	0.050	0.112	0.124	0.085	53	976	109.1	6.19
IPI01023315.1	Uncharacterized protein (Fragment)	82.18%	3	21	99	1026	0.102	-0.031	0.135	-0.006	0.156	104	0.064	0.010	0.158	-0.105	0.219	49	-0.020	-0.025	-0.044	0.016	-0.019	74	0.157	0.089	0.193	0.010	0.183	72	0.041	-0.031	0.001	-0.009	0.034	19	662	77.4	8.94
IPI01027757.1	Uncharacterized protein (Fragment)	76.28%	6	9	52	527	0.014	0.007	0.069	-0.012	0.011	22	0.058	0.017	0.016	0.027	-0.109	29	0.108	-0.056	-0.057	0.070	0.174	22	0.160	0.095	0.197	0.036	0.172	23	0.091	0.058	0.095	0.075	0.024	42	430	49.6	6.44
IPI00169584.4	Uncharacterized protein C12orf43 homolog	76.56%	1	3	15	44	0.265	-0.193	0.103	-0.091	0.112	3																									256	27.6	8.70
IPI00378513.3	Uncharacterized protein C13orf27 homolog	54.67%	2	3	16	31							-0.240	-0.083	-0.271	-0.160	-0.183	4																			225	25.2	8.75
IPI00133958.2	Uncharacterized protein C19orf43 homolog	95.38%	1	8	22	183							-0.017	-0.162	-0.120	-0.111	-0.076	25	-0.905	-0.624	-1.595	-0.667	-1.074	4													173	18.4	9.67
IPI00187478.1	Uncharacterized protein C1orf96 homolog	55.56%	1	4	17	61																									-0.109	0.095	-0.112	0.027	0.147	5	252	28.4	9.22
IPI00113109.3	Uncharacterized protein C3orf26 homolog	70.29%	1	3	29	85	-0.029	0.019	0.007	-0.034	-0.077	3													0.104	0.054	0.183	-0.045	0.023	7							276	31.6	8.46
IPI00761985.1	uncharacterized protein C4orf3 homolog	61.54%	2	1	9	40							0.044	0.122	-0.474	-0.054	0.106	3																			65	7.4	5.24
IPI00133350.1	Uncharacterized protein C6orf125 homolog	69.12%	3	4	9	31							-0.742	0.593	-0.786	0.073	-0.685	3																			136	16.3	9.19
IPI00403618.4	Uncharacterized protein C6orf174 homolog	73.76%	4	7	96	245																									-0.106	-0.044	-0.116	0.050	-0.152	13	945	103.4	6.14
IPI00110456.2	Uncharacterized protein C7orf50 homolog	65.13%	1	3	15	44							-0.723	0.099	-0.884	-0.082	-0.216	3							-0.715	-0.001	-0.567	-0.095	-0.655	4							195	22.3	9.50
IPI00989332.1	uncharacterized protein CXorf49 homolog	77.68%	2	1	38	110																															475	50.4	9.13
IPI00377731.2	Uncharacterized protein KIAA0513	51.35%	4	13	31	105																									-0.063	-0.228	-0.285	-0.318	-0.218	29	407	46.3	5.02
IPI00330169.11	Uncharacterized protein KIAA1107	74.80%	4	4	110	325																									-0.089	-0.131	0.031	-0.133	0.055	3	1369	148.6	6.51
IPI00224491.1	Uncharacterized protein KIAA1671	52.92%	1	1	19	36	0.333	0.209	0.538	-0.039	0.487	3																									308	34.6	6.09
IPI00122392.1	UPF0160 protein MYG1, mitochondrial	91.84%	3	7	37	212	-0.074	0.070	-0.120	0.047	0.344	11	-0.257	-0.484	-0.288	-0.262	-0.111	9	-0.007	0.061	0.055	-0.103	0.175	3	-0.109	0.231	0.009	0.001	-0.086	10							380	42.7	7.02
IPI00270788.1	UPF0327 protein C1orf151 homolog	93.42%	1	1	5	13																									0.034	0.059	-0.062	0.208	0.029	3	76	8.6	8.94
IPI00118827.1	UPF0444 transmembrane protein C12orf23 homolog	95.65%	1	3	13	100	-0.077	-0.020	-0.072	-0.007	-0.199	3	0.024	0.255	0.126	0.236	0.044	3													-0.193	-0.201	-0.072	0.290	-0.160	3	115	11.5	9.32
IPI00109253.1	UPF0480 protein C15orf24 homolog	69.29%	1	6	25	55													0.174	0.154	0.085	-0.099	-0.134	4													241	26.3	9.23
IPI00461011.3	UPF0552 protein C15orf38 homolog	74.34%	1	6	29	109	0.200	-0.003	0.143	-0.150	0.059	3	-0.176	-0.146	-0.147	-0.233	0.144	7	-0.022	-0.092	-0.355	0.287	0.276	4													226	25.2	5.19
IPI00131954.1	UPF0556 protein C19orf10 homolog	90.96%	1	5	10	75	-0.042	0.069	-0.016	-0.020	-0.068	8	-0.149	-0.176	-0.044	0.051	-0.203	11							0.111	-0.020	0.047	-0.010	-0.017	8							166	18.0	6.79
IPI00132456.1	UPF0568 protein C14orf166 homolog	87.70%	2	13	29	363	0.043	-0.013	-0.117	-0.017	-0.053	32	0.047	-0.099	0.057	-0.042	0.119	18	0.126	0.056	0.092	0.052	0.081	20	0.091	-0.026	0.180	0.026	0.143	37	-0.082	-0.060	-0.003	0.099	-0.020	13	244	28.1	6.89
IPI00121271.1	UPF0585 protein C16orf13 homolog	94.61%	5	4	18	55																									-0.116	0.055	0.108	0.116	0.050	4	204	22.7	6.52
IPI00229103.1	UPF0608 protein C19orf42 homolog	54.67%	1	2	3	10	0.136	-0.201	-0.056	-0.053	-0.001	3																									75	8.5	8.16
IPI00229286.5	UPF0609 protein C4orf27 homolog	67.34%	1	4	29	69							-0.482	-0.010	0.184	-0.041	-0.128	3							-0.006	0.073	0.218	0.110	0.213	5							346	39.3	7.69
IPI00331014.1	UPF0670 protein C8orf55 homolog	86.96%	1	4	28	78	0.443	-0.068	0.564	-0.105	0.374	3	0.393	-0.077	0.297	-0.080	0.256	4							0.438	0.007	0.246	0.030	0.312	3							207	23.8	9.11
IPI00109313.1	UPF0760 protein C2orf29 homolog	69.31%	1	5	37	163							-0.072	-0.025	0.007	-0.106	-0.279	5	-0.392	-0.269	-0.085	0.058	-0.253	3													505	54.9	6.55
IPI00187272.3	UPF0765 protein C10orf58 homolog	86.24%	7	9	19	81	-0.167	-0.149	-0.028	-0.050	-0.154	4																			-0.012	0.135	-0.003	0.054	-0.047	21	218	24.4	9.17
IPI00119714.2	Upstream binding transcription factor, RNA polymerase I, isoform CRA_b	85.37%	7	17	112	392	0.111	0.009	0.116	0.051	0.126	13	0.069	0.096	0.212	0.106	-0.019	17	0.004	-0.007	-0.056	-0.099	-0.042	16	0.134	0.209	-0.013	0.141	0.089	9							752	88.3	9.89
IPI00121552.2	Uridine 5'-monophosphate synthase	90.85%	1	13	50	257	-0.170	-0.010	-0.146	-0.077	-0.141	12	0.166	-0.123	0.244	-0.013	0.285	6	-0.349	-0.356	-0.396	-0.059	-0.186	5	0.045	0.135	0.048	-0.041	0.112	13							481	52.3	6.61
IPI00121203.1	Uridine-cytidine kinase 2	83.14%	1	2	26	88																			0.270	0.249	0.287	0.141	0.302	5							261	29.4	6.55
IPI00131031.3	Uroplakin-1b	46.15%	1	2	12	24							2.354	-0.001	2.038	-0.038	1.906	3																			260	29.7	5.82
IPI00111958.2	Uroporphyrinogen decarboxylase	40.60%	1	4	14	28	0.289	-0.130	0.239	-0.047	0.330	4	-0.176	0.024	-0.048	0.187	-0.062	4																			367	40.7	6.65
IPI00353420.7	utrophin	81.17%	5	50	379	1585	-0.216	0.033	-0.302	-0.035	-0.130	14	-0.042	0.099	-0.086	0.157	-0.181	33	0.043	0.062	-0.013	-0.038	-0.079	44	-0.025	0.072	-0.103	-0.105	-0.007	10							3430	392.5	5.33
IPI00108774.2	UV excision repair protein RAD23 homolog B	59.62%	3	11	25	457	0.096	-0.099	-0.097	-0.100	0.011	19	0.087	0.017	0.122	-0.023	0.027	28	-0.278	-0.140	-0.223	-0.056	-0.163	21	-0.094	-0.048	-0.072	-0.022	-0.100	23	0.026	-0.134	-0.044	0.040	-0.012	22	416	43.5	4.83
IPI00111181.1	Vacuolar protein sorting-associated protein 35	79.27%	1	30	82	763	-0.020	-0.006	-0.020	0.023	0.065	32	0.001	0.051	0.066	0.114	0.054	39	-0.109	-0.077	-0.072	-0.013	-0.077	28	-0.048	-0.041	-0.079	0.017	0.048	41	-0.094	-0.017	-0.045	-0.042	0.081	56	796	91.7	5.44
IPI00153207.3	Vacuolar protein sorting-associated protein 37B	98.60%	1	6	29	180	0.145	-0.074	0.205	0.094	0.027	6	-0.576	-0.019	-0.419	-0.112	-0.240	7																			285	31.0	7.05
IPI00312172.5	Vacuolar protein sorting-associated protein 4A	80.55%	1	2	44	195	0.068	-0.114	0.120	-0.012	0.122	14	-0.295	-0.010	-0.326	-0.144	-0.125	4							0.180	0.193	0.095	0.096	0.124	9	0.096	-0.029	0.084	0.198	0.095	6	437	48.9	7.80
IPI00133700.2	Vacuolar protein sorting-associated protein 4B	89.41%	2	11	46	260	0.055	-0.114	0.120	-0.025	0.083	20	-0.223	-0.094	-0.285	-0.136	-0.150	9							0.183	0.207	0.087	0.126	0.167	10	0.134	0.055	0.085	0.183	0.178	5	444	49.4	7.11
IPI00133024.1	Vacuolar protein sorting-associated protein VTA1 homolog	56.96%	3	3	22	91	-0.224	-0.071	0.085	-0.057	0.102	5													0.124	0.061	-0.044	-0.088	0.144	8	0.009	0.198	0.061	-0.114	0.047	9	309	33.9	6.13
IPI00129014.1	Vacuolar protein sorting-associated protein 36	75.65%	1	3	34	95	-0.112	-0.154	-0.034	-0.079	0.036	4																									386	43.7	7.15
IPI00387192.4	Vacuole membrane protein 1	55.17%	1	1	24	155	-0.231	0.000	-0.405	0.094	-0.065	9													-0.265	-0.088	-0.208	-0.162	0.062	19							406	45.9	6.95
IPI00130353.5	Valyl-tRNA synthetase	81.24%	22	50	121	1136	-0.076	-0.029	-0.114	0.004	-0.152	99	0.055	0.047	0.033	-0.029	0.055	91	-0.057	-0.065	-0.038	0.073	-0.013	70	0.306	0.110	0.352	0.033	0.262	73	0.068	0.130	0.119	-0.111	-0.072	11	1263	140.1	7.77
IPI00653571.2	vascular cell adhesion protein 1 precursor	83.36%	2	10	70	328							0.087	0.106	0.293	0.122	0.062	24	-0.243	0.051	-0.128	-0.293	-0.063	7													739	81.3	5.34
IPI00124497.3	Vascular endothelial growth factor receptor 1	81.17%	10	1	135	688	0.148	-0.327	-0.188	-0.111	0.187	5																			-0.199	-0.116	-0.155	-0.128	-0.022	4	1333	149.8	8.31
IPI00624876.3	Vasodilator-stimulated phosphoprotein	63.47%	1	10	33	142	-0.044	-0.022	0.077	-0.079	-0.035	8	-0.074	-0.292	-0.130	-0.105	-0.286	17	0.487	-0.064	0.596	0.503	0.622	3	0.205	0.169	0.148	0.062	0.007	5							375	39.6	8.53
IPI00119203.4	Very long-chain specific acyl-CoA dehydrogenase, mitochondrial	92.68%	2	21	81	474	-0.072	-0.131	-0.094	-0.059	-0.082	20	0.371	0.130	0.296	-0.072	0.309	14	0.068	0.036	0.050	-0.106	0.016	23	-0.523	0.179	-0.438	0.185	-0.375	15	-0.248	0.027	0.072	-0.019	0.000	9	656	70.8	8.75
IPI00130648.1	very low-density lipoprotein receptor isoform b precursor	75.15%	6	5	59	167							0.207	0.355	0.100	0.192	0.151	13																			845	93.5	4.79
IPI00133212.1	Vesicle transport protein GOT1B	30.43%	1	3	6	25																			0.192	-0.111	0.539	-0.184	0.218	3							138	15.4	10.58
IPI00880852.1	Vesicle-associated membrane protein 2	76.07%	2	2	14	249	0.011	-0.184	0.020	0.085	0.235	6	0.393	0.026	0.387	0.044	0.331	6	0.099	0.114	0.051	-0.193	0.089	13	-0.517	0.006	-0.472	0.029	-0.621	17	0.045	-0.130	-0.189	-0.180	-0.175	36	163	17.9	9.38
IPI00132276.1	Vesicle-associated membrane protein 3	95.15%	1	1	15	238	0.091	0.029	0.127	0.098	0.225	12							-0.086	0.113	-0.019	-0.186	-0.231	15	-0.442	-0.074	-0.379	0.023	-0.517	18	0.116	-0.083	-0.211	-0.176	-0.065	23	103	11.5	8.50
IPI00404693.1	Vesicle-associated membrane protein 4, isoform CRA_c	63.83%	2	3	12	45							0.217	0.166	0.124	0.004	0.151	3							-0.442	0.065	-0.238	0.009	-0.228	6							141	16.3	7.44
IPI00453589.1	Vesicle-associated membrane protein 8	96.04%	1	3	13	26							0.209	-0.311	0.250	-0.076	0.092	3	0.198	0.031	0.141	0.009	0.040	4													101	11.4	8.19
IPI00125267.4	Vesicle-associated membrane protein-associated protein A	95.18%	1	15	35	532	0.047	0.106	-0.019	0.014	0.034	39	0.288	-0.003	0.160	0.062	0.128	30	0.317	0.137	0.195	0.010	0.095	51	-0.029	0.036	0.044	0.010	-0.031	41	0.023	0.031	0.020	-0.111	-0.053	48	249	27.8	8.40
IPI00308187.1	vesicle-associated membrane protein-associated protein B	97.94%	3	12	35	415	-0.034	0.100	-0.034	0.077	-0.134	27	-0.260	0.055	-0.525	0.021	-0.576	21	0.128	0.105	0.110	0.013	0.098	30	-0.059	0.066	-0.031	0.031	-0.038	13	-0.034	0.024	0.034	-0.091	0.024	42	243	26.9	7.78
IPI00656325.2	Vesicle-fusing ATPase	90.46%	3	52	107	1082	-0.117	-0.030	-0.085	-0.004	-0.123	26	0.229	0.115	0.134	0.104	0.076	21	-0.037	0.014	0.038	-0.077	-0.030	23	-0.288	0.059	-0.390	0.027	-0.304	26	0.103	-0.068	-0.088	-0.085	-0.063	204	744	82.6	6.95
IPI00114368.3	Vesicle-trafficking protein SEC22b	76.28%	2	7	31	316	0.021	-0.065	0.043	-0.060	0.024	19	0.070	-0.026	-0.028	-0.004	0.081	13	-0.017	0.112	0.279	-0.122	0.108	17	-0.039	-0.083	0.021	-0.025	-0.009	14	0.246	0.179	0.227	0.066	0.104	9	215	24.7	8.51
IPI00109153.7	Vesicular glutamate transporter 1	40.54%	4	9	25	108																									0.256	-0.288	-0.258	-0.203	-0.248	29	560	61.6	7.34
IPI00321634.2	Vesicular integral-membrane protein VIP36	68.16%	1	10	26	128	-0.344	0.088	-0.108	0.036	-0.223	12	0.388	-0.094	0.438	-0.094	0.349	16	0.534	0.106	0.282	-0.103	0.358	4	-0.042	0.030	-0.221	0.068	-0.080	7							358	40.4	6.95
IPI00123379.1	Vigilin	90.06%	2	57	175	1291	0.094	0.013	0.072	0.023	0.041	95	0.120	-0.023	0.045	0.029	0.016	96	-0.157	0.035	-0.085	0.045	-0.094	94	-0.029	-0.060	-0.045	-0.041	-0.053	34	-0.062	-0.041	-0.109	-0.077	-0.157	8	1268	141.7	6.87
IPI00227299.6	Vimentin	98.93%	3	42	85	3110	-0.128	0.050	-0.127	0.011	-0.213	173	0.285	0.061	0.100	0.036	0.115	174	0.197	0.134	0.091	0.021	-0.010	430	-0.170	0.001	-0.134	-0.035	-0.139	191	0.076	-0.068	-0.092	-0.191	0.112	85	466	53.7	5.12
IPI00405227.3	Vinculin	98.22%	3	60	179	2142	-0.216	-0.050	-0.168	-0.023	-0.157	202	0.576	0.019	0.544	0.014	0.467	87	-0.054	-0.116	-0.080	0.052	0.044	154	0.339	0.004	0.297	-0.044	0.315	41	0.131	0.070	0.020	0.047	0.138	36	1066	116.6	6.00
IPI00230418.5	Visinin-like protein 1	90.58%	1	13	28	159																									-0.016	-0.082	0.031	-0.001	-0.009	60	191	22.1	5.15
IPI00421220.1	Vitamin K epoxide reductase complex subunit 1-like protein 1	52.27%	1	1	14	27																			0.242	0.072	0.089	0.077	0.227	3							176	19.8	8.95
IPI00136012.1	Vitamin K-dependent gamma-carboxylase	42.01%	1	3	38	79	0.332	0.184	0.433	0.552	0.225	3																									757	87.1	8.02
IPI00122547.1	Voltage-dependent anion-selective channel protein 2	94.58%	4	15	28	857	-0.048	0.065	-0.019	0.026	-0.077	50	0.252	-0.088	0.181	-0.009	0.193	39	0.308	0.069	0.361	0.016	0.179	55	0.072	-0.028	0.098	-0.022	0.131	61	-0.072	-0.138	-0.059	-0.127	-0.119	86	295	31.7	7.49
IPI00122548.4	Voltage-dependent anion-selective channel protein 3	76.68%	2	13	29	708	0.089	-0.048	-0.034	0.024	-0.080	24	0.314	0.083	0.261	0.178	0.309	43	0.300	0.118	0.244	-0.019	0.061	33	0.096	0.095	0.038	0.051	0.126	45	0.013	-0.099	-0.119	-0.125	-0.102	55	283	30.7	8.79
IPI00128826.1	Voltage-dependent calcium channel gamma-8 subunit	52.72%	1	4	17	80																									0.256	0.233	-0.042	-0.150	0.131	6	423	43.4	9.20
IPI00408646.5	Voltage-dependent P/Q-type calcium channel subunit alpha-1A	66.47%	3	3	192	563							-0.510	-0.059	-0.561	0.055	-0.490	4																			2368	267.5	8.85
IPI00221817.5	von Willebrand factor A domain-containing protein 5A	68.10%	3	8	71	268							0.190	-0.134	0.881	-0.112	0.335	4													0.181	0.287	0.094	0.047	0.108	8	793	87.1	6.58
IPI00923083.1	Vsig1 protein	39.05%	3	3	14	95	1.163	0.116	1.009	0.009	0.993	19																									443	48.1	4.78
IPI00119113.3	V-type proton ATPase subunit B, brain isoform	92.76%	6	29	64	879	-0.080	-0.044	0.085	-0.095	-0.060	20	0.036	-0.056	0.023	0.174	-0.010	19	-0.233	-0.125	-0.094	-0.116	-0.125	15	-0.388	-0.120	-0.278	-0.091	-0.160	55	0.099	-0.184	-0.150	-0.218	-0.184	150	511	56.5	5.81
IPI00130186.4	V-type proton ATPase subunit C 1	97.91%	1	22	69	456	0.050	0.061	0.293	0.262	-0.068	4	-0.042	0.050	0.059	-0.154	0.069	5	-0.375	-0.116	-0.320	0.145	-0.228	8	-0.119	-0.053	-0.111	0.095	-0.223	7	0.183	-0.097	-0.117	-0.102	-0.114	60	382	43.9	7.46
IPI00118787.1	V-type proton ATPase subunit D	87.04%	3	10	34	245	-0.009	-0.063	0.081	-0.006	-0.069	5													-0.525	-0.130	-0.442	-0.028	-0.178	6	0.108	-0.199	-0.199	-0.234	-0.133	40	247	28.4	9.45
IPI00313841.1	V-type proton ATPase subunit d 1	68.66%	1	12	23	264							0.503	0.315	0.322	0.315	0.273	5	0.280	0.123	0.222	-0.022	0.013	11	-0.335	0.197	0.149	0.050	0.045	10	0.227	-0.071	-0.088	-0.076	-0.017	44	351	40.3	5.00
IPI00119115.2	V-type proton ATPase subunit E 1	78.76%	2	18	39	404	0.267	0.029	0.309	-0.012	0.296	3	0.107	0.052	0.045	0.333	0.223	3	-0.265	-0.090	-0.306	0.004	-0.189	12	-0.306	0.096	-0.304	0.047	-0.349	17	0.016	-0.191	-0.199	-0.250	-0.178	74	226	26.1	8.43
IPI00315999.4	V-type proton ATPase subunit F	81.51%	2	7	9	42	0.095	-0.214	0.062	-0.050	0.118	3	-0.460	-0.278	-0.488	-0.245	-0.150	3													0.077	-0.114	-0.120	-0.138	-0.160	16	119	13.4	5.82
IPI00133163.3	V-type proton ATPase subunit G 1	61.02%	1	2	7	33							-0.066	-0.293	-0.364	0.052	-0.353	6							0.579	0.268	0.212	0.484	0.107	3							118	13.7	7.97
IPI00123817.1	V-type proton ATPase subunit G 2	93.22%	2	6	20	130																									0.100	-0.165	-0.032	-0.234	-0.097	34	118	13.6	10.26
IPI00311461.1	V-type proton ATPase subunit H	94.20%	1	22	62	657	0.095	-0.146	0.193	0.062	0.081	30	0.276	-0.085	0.001	0.234	-0.039	8	-0.109	-0.204	-0.394	0.047	-0.198	7	-0.183	0.065	-0.184	0.051	-0.088	35	0.130	-0.252	-0.257	-0.252	-0.196	96	483	55.8	6.61
IPI00128989.1	V-type proton ATPase subunit S1	50.54%	7	6	22	87																									-0.051	-0.278	-0.141	0.016	-0.167	4	463	51.0	5.92
IPI00890049.1	WAS protein family homolog 1	63.16%	1	4	42	112	-0.112	-0.278	-0.157	-0.482	-0.034	5																									475	51.6	5.44
IPI00957001.1	WAS/WASL-interacting protein family member 3 isoform b	56.35%	4	3	33	92																									-0.032	0.128	-0.472	-0.173	-0.091	4	449	45.1	10.84
IPI00108450.3	WD and tetratricopeptide repeats protein 1	79.32%	2	3	50	90							0.321	0.273	0.171	0.489	0.398	3																			677	75.7	7.15
IPI00754723.2	WD repeat and FYVE domain containing 1 isoform 1	55.37%	3	1	24	81																									0.158	-0.032	0.112	0.004	-0.076	4	410	46.2	7.34
IPI00466978.3	WD repeat and HMG-box DNA-binding protein 1	58.83%	3	6	69	159	-0.130	0.010	-0.170	-0.080	-0.059	3	-0.091	0.124	0.271	-0.186	0.127	5																			1081	120.3	5.83
IPI00170242.1	WD repeat domain 33 isoform 1	63.23%	5	8	101	303	-0.056	0.024	-0.160	-0.076	-0.092	4	-0.060	-0.019	-0.086	0.225	0.021	4	0.455	0.125	0.363	0.065	0.409	4	-0.123	-0.336	0.023	-0.109	-0.158	3							1330	145.2	9.13
IPI00876121.1	WD repeat domain 36 isoform 1	78.75%	3	15	72	231	-0.094	-0.016	0.038	0.023	0.102	7	-0.223	-0.133	-0.029	0.088	-0.108	6	0.118	0.044	0.085	-0.142	-0.133	5	0.102	0.045	-0.074	0.003	0.033	13							899	99.7	7.11
IPI00314748.7	WD repeat-containing protein 1	92.90%	2	27	63	752	0.139	-0.120	0.309	0.045	0.312	51	-0.245	-0.079	-0.150	-0.017	-0.133	64	0.029	-0.062	0.088	0.176	0.160	36	0.210	-0.041	0.228	-0.056	0.331	11	0.068	0.066	0.020	-0.117	0.004	59	606	66.4	6.60
IPI00136252.3	WD repeat-containing protein 18	52.20%	2	6	27	131	-0.146	-0.120	-0.138	-0.059	-0.173	8	0.525	-0.189	0.475	0.220	0.141	3	-0.016	0.036	0.026	-0.191	-0.045	4	-0.014	0.144	0.006	0.076	0.023	5							431	47.2	6.89
IPI00226275.3	WD repeat-containing protein 26	57.72%	3	5	38	122	-0.213	0.034	-0.229	-0.133	-0.155	3																									641	70.5	6.16
IPI00221822.1	WD repeat-containing protein 3	74.63%	1	8	83	250							0.033	-0.138	-0.144	0.087	-0.065	9	-0.100	-0.004	0.166	0.038	-0.013	4													942	105.7	6.64
IPI00387421.1	WD repeat-containing protein 37	82.46%	3	8	53	152																									0.076	-0.047	0.009	-0.077	-0.028	12	496	55.0	7.23
IPI00938465.1	WD repeat-containing protein 43	76.22%	1	12	74	279	-0.245	0.084	-0.142	0.102	-0.271	17							0.047	0.021	0.034	-0.183	-0.031	3	0.276	0.043	0.275	0.016	0.041	13							677	75.3	5.38
IPI00129141.1	WD repeat-containing protein 46	78.46%	1	5	67	253	-0.326	-0.014	-0.160	-0.003	-0.178	6	0.157	0.258	-0.045	0.140	-0.194	4							-0.086	-0.028	0.034	-0.112	-0.085	7							622	69.0	9.77
IPI00330193.4	WD repeat-containing protein 47	55.76%	2	5	48	109																									-0.044	-0.085	-0.085	-0.105	-0.034	9	920	102.2	5.97
IPI00139957.1	WD repeat-containing protein 5	76.05%	2	6	28	135	0.081	0.194	0.204	0.041	0.017	6	-0.020	0.128	0.178	0.172	0.270	5	-0.047	-0.020	0.209	0.064	0.127	10	-0.082	-0.127	-0.231	-0.029	-0.038	6							334	36.6	8.27
IPI00112320.4	WD repeat-containing protein 61	67.87%	4	6	19	103	-0.105	-0.001	0.092	-0.068	0.099	9	-0.088	0.036	-0.120	-0.127	0.104	10							0.380	-0.175	0.081	0.003	0.029	5							305	33.8	5.36
IPI00221414.1	WD repeat-containing protein 82	57.19%	1	3	23	71							-0.290	-0.283	-0.117	-0.125	-0.053	5							-0.112	-0.265	-0.238	-0.068	-0.142	3							313	35.1	7.69
IPI00420597.3	Wings apart-like protein homolog	83.08%	3	8	127	417	-0.133	-0.241	-0.074	-0.243	-0.082	6	-0.322	0.110	-0.331	-0.506	-0.211	5	-0.149	-0.215	0.332	-0.014	0.994	3													1200	134.0	5.41
IPI00471372.2	Wiskott-Aldrich syndrome protein family member 1	34.35%	1	5	27	96																									0.096	-0.071	0.007	-0.054	-0.083	13	559	61.5	6.37
IPI00221723.1	Wiskott-Aldrich syndrome protein family member 2	38.43%	2	5	30	74	0.194	0.070	-0.062	-0.031	0.088	5	0.424	0.148	0.158	0.253	-0.496	3																			497	54.0	5.53
IPI00128341.1	Wiskott-Aldrich syndrome protein family member 3	38.32%	1	2	24	61																									0.106	0.048	0.181	0.195	-0.054	4	501	55.2	6.37
IPI00123333.5	WW domain-binding protein 11	59.13%	2	7	58	272	0.050	-0.023	-0.142	0.014	-0.062	10							0.239	0.031	0.072	0.034	0.269	7	0.295	-0.004	0.213	-0.007	0.222	10							641	69.8	8.40
IPI00117019.7	WW domain-binding protein 4	47.87%	1	1	24	69	-0.039	-0.095	-0.074	-0.013	0.031	4													0.136	0.126	0.219	0.068	0.059	4							376	42.1	7.36
IPI00406419.4	Xaa-Pro dipeptidase	52.13%	1	3	34	129							0.040	-0.025	-0.128	-0.114	0.052	3							0.139	-0.044	-0.014	0.004	0.419	6							493	55.0	5.78
IPI00420231.4	X-prolyl aminopeptidase (Aminopeptidase P) 1, soluble, isoform CRA_b	73.42%	2	22	51	595	0.020	-0.020	0.080	-0.032	0.136	44	-0.119	-0.020	0.047	-0.060	0.069	30	-0.022	-0.184	-0.165	0.235	-0.211	6	-0.527	0.098	-0.504	0.059	-0.494	29							666	74.5	5.83
IPI00321154.4	X-ray repair cross-complementing protein 5	88.11%	1	13	72	223	0.392	-0.149	0.198	0.059	-0.054	5	-0.013	-0.041	-0.074	-0.231	-0.178	7							0.381	0.107	0.419	0.092	0.385	4							732	83.0	5.16
IPI00132424.4	X-ray repair cross-complementing protein 6	75.82%	7	13	61	248	0.007	-0.051	0.085	-0.109	0.091	10	0.274	0.030	0.448	-0.007	0.167	7	-0.379	-0.093	-0.373	-0.080	-0.146	15	0.095	0.149	0.268	0.055	0.161	13							608	69.4	6.79
IPI00775820.1	Yip1 domain family, member 6	26.77%	2	1	3	8	0.365	0.085	0.348	0.048	0.391	3																									198	21.8	5.99
**IPI00621973.4**	**YLP motif-containing protein 1**	**53.95%**	**6**	**15**	**141**	**401**	**-0.175**	**-0.060**	**-0.109**	**-0.150**	**-0.257**	**6**	**0.130**	**0.023**	**0.051**	**0.183**	**0.112**	**10**	**0.085**	**-0.008**	**-0.117**	**-0.245**	**0.271**	**3**	**-0.130**	**0.181**	**-0.089**	**-0.088**	**-0.233**	**5**							**2139**	**240.9**	**6.68**
IPI00187444.1	YTH domain family protein 1	43.65%	2	3	33	208	-0.119	0.047	-0.020	-0.020	-0.016	12	-0.158	-0.109	-0.204	-0.211	-0.065	9	-0.255	-0.100	-0.162	-0.102	-0.188	8	0.230	-0.014	0.210	0.023	0.279	7							559	60.8	8.95
IPI00314865.10	YTH domain family protein 2	49.22%	2	6	41	366	0.006	-0.017	-0.003	-0.029	-0.048	8	-0.142	-0.048	-0.189	-0.211	-0.065	15	0.038	0.066	-0.057	-0.094	-0.019	9	0.148	0.023	0.162	0.033	0.204	10							579	62.2	8.79
IPI00421162.4	Zinc finger CCCH domain-containing protein 11A	72.60%	6	9	84	323	-0.141	0.144	-0.150	0.013	-0.163	15							-0.102	0.090	0.058	0.024	-0.054	4	-0.053	-0.105	-0.257	-0.060	-0.020	5							792	86.4	8.13
IPI00673693.1	zinc finger CCCH domain-containing protein 18 isoform a	59.88%	2	10	83	378	0.171	0.230	0.198	0.126	0.119	17	-0.076	-0.194	-0.229	-0.173	0.158	6	0.205	-0.035	0.023	-0.245	0.036	15	0.050	0.052	-0.086	0.047	0.001	16							972	108.2	8.00
IPI00187270.8	Zinc finger CCHC domain-containing protein 8	67.56%	1	11	52	318							-0.037	-0.149	-0.119	-0.071	-0.158	19																			709	78.0	4.73
IPI00911179.1	zinc finger protein 207 isoform 4	19.57%	6	4	11	53	0.484	0.047	0.356	-0.039	0.348	4							-0.150	-0.103	-0.127	0.057	0.059	5													460	49.4	9.10
IPI00222382.5	Zinc finger protein 512	80.07%	1	6	57	216							-0.333	-0.517	-0.327	-0.318	-0.525	6																			562	63.9	9.51
IPI00321357.2	Zinc finger protein 593	61.19%	1	3	10	46	0.170	-0.071	0.392	-0.056	0.394	9													-0.054	-0.072	0.013	-0.095	0.017	6							134	15.1	9.55
IPI00127449.1	Zinc finger protein 622	62.39%	1	8	50	119	0.014	0.003	0.037	0.010	-0.010	6													0.314	0.138	0.287	0.116	0.604	3							476	53.4	6.10
IPI00132963.1	Zinc finger protein 706	78.95%	2	4	12	31	0.110	-0.119	0.147	-0.041	0.178	6							-0.057	0.001	-0.023	0.341	0.075	3	0.169	-0.186	0.225	-0.092	0.366	3							76	8.5	10.01
IPI00131810.2	Zinc finger RNA-binding protein	62.29%	3	15	84	467	-0.196	0.103	0.054	0.024	0.130	13	0.000	-0.077	0.088	0.064	0.144	7	0.352	0.130	0.293	0.111	-0.020	6	-0.423	-0.108	-0.155	-0.056	-0.279	9							1074	116.8	9.04
IPI00605489.3	zinc finger, A20 domain containing 1	70.71%	3	4	56	170																															840	91.9	6.86
IPI00118834.1	Zinc transporter SLC39A7	27.94%	1	3	15	60	-0.048	0.167	-0.240	-0.001	-0.208	6	-0.047	-0.056	-0.229	-0.216	0.069	5							-0.103	-0.050	-0.071	-0.069	0.037	3	-0.131	0.054	-0.019	0.021	0.135	3	476	50.6	6.87
IPI00273801.3	Zinc transporter ZIP10	28.45%	2	5	25	78	0.004	-0.010	-0.470	-0.189	-0.086	4																			-0.063	-0.264	0.048	-0.171	-0.396	4	833	94.3	6.71
IPI00133126.1	ZW10 interactor	76.19%	1	3	28	172	-0.599	-0.094	-0.085	-0.141	-0.299	4							-0.158	0.054	-0.188	0.107	0.360	5													252	28.7	8.43
IPI00228360.4	Zyxin	53.19%	3	14	34	289	0.130	-0.026	0.214	0.047	0.135	18	-0.102	0.136	-0.208	-0.264	-0.006	23	0.217	-0.098	0.194	0.034	0.292	41													564	60.5	6.40
